# A 16 × 8 Focused Compound Library Comprising All Configurational Isomers of the Archetypal Iminosugar, 1‑Deoxynojirimycin

**DOI:** 10.1021/acs.jmedchem.6c01210

**Published:** 2026-08-10

**Authors:** Richard J. B. H. N. van den Berg, Adrianus M. C. H. van den Nieuwendijk, Bing Liu, Maria J. Ferraz, Cecile M. J. Ouairy, Amar T. Ghisaidoobe, Qiang Ma, Hans van den Elst, Anneke Strijland, Wilma E. Donker-Koopman, Marri Verhoek, Koen J. Rijpkema, Rolf G. Boot, Tom Wennekes, Gijsbert A. van der Marel, Constant A. A. van Boeckel, Marta Artola, Johannes M. F. G. Aerts, Herman S. Overkleeft

**Affiliations:** Gorlaeus Laboratories, 201229 Leiden Institute of Chemistry, Einsteinweg 55, Leiden 2333 CC, The Netherlands

## Abstract

Deoxynojirimycin is the archetypal iminosugar, from which many structural and configurational isosteres have been derived and studied in past decades. In total, 16 configurational isomers of deoxynojirimycin exist, but these have not yet been collected in a single library. This paper describes the assembly of such a library with each configurational isomer present in eight *N*-substituted forms. Screening of this library against the three glucosylceramide processing enzymes, glucosylceramide synthase (GCS), lysosomal glucosylceramidase (GBA1), and nonlysosomal glucosylceramidase (GBA2), confirms earlier data that appropriately substituted D-*Glc*, D-*Gal*, and L-*Ido* deoxynojirimycins are most effective in dually inhibiting GCS and GBA2. In contrast to our expectations, we found that the same holds true for the L-*alt* isomer. We believe that our comprehensive library, as reported here, could prove valuable in drug discovery endeavors targeting cancer, infectious diseases, and inherited glycosphingolipidoses.

## Graphical abstract

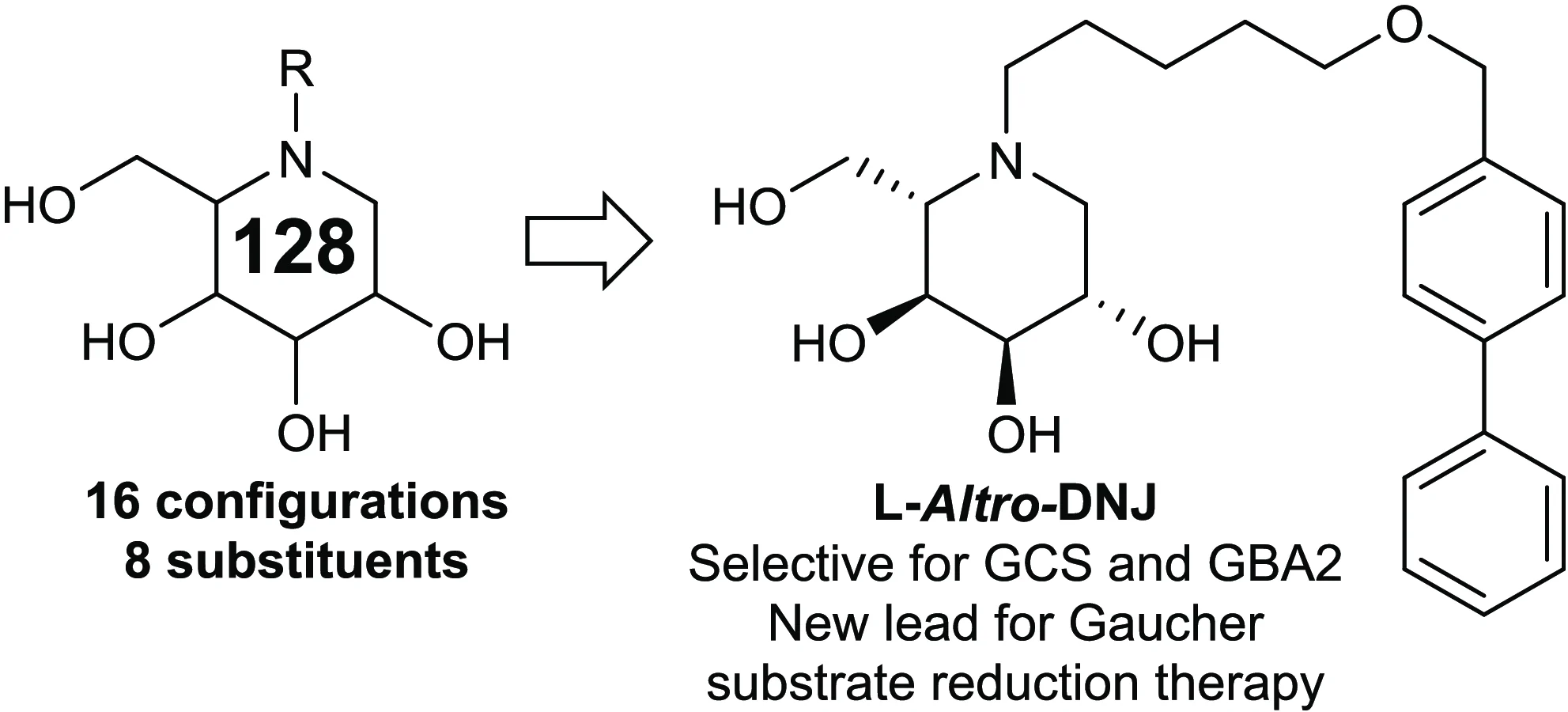

## Introduction

Iminosugars comprise an important class of glycomimetics and are pursued widely as modulators of glycoprocessing enzymes in the context of medicinal chemistry and drug research.^1^ Iminosugars currently in the clinic include *N*-hydroxyethyl-deoxynojirimycin (Miglitol, **1**, Figure 1), a digestive α-glucosidase inhibitor used for the treatment of type 2 diabetes,^2^ and *N-*butyl-deoxynojirimycin (Miglustat, Zavesca, **2**), a glucosylceramide synthase (GCS) inhibitor used for the treatment of non-neuronopathic Gaucher disease^3^ (substrate reduction therapy). Over the past decades, numerous natural product iminosugars have been discovered, and massive synthesis efforts over the same period have led to the generation of numerous structurally and functionally different iminosugars.^4^ Among others, these studies led to the development of 1-deoxygalactonojirimycin (D-*Gal*-DNM) as a clinical drug for the treatment of Fabry disease, where it acts as a pharmacological chaperone to stabilize mutant lysosomal α-galactosidase (α-GalA).^5^ Research efforts on deoxynojirimycin isosters have been scattered over many research laboratories, and therefore, comprehensive iminosugar compound libraries for medicinal chemistry studies and glycoprocessing enzyme inhibitor screening campaigns are scarce.

**Figure 1:**
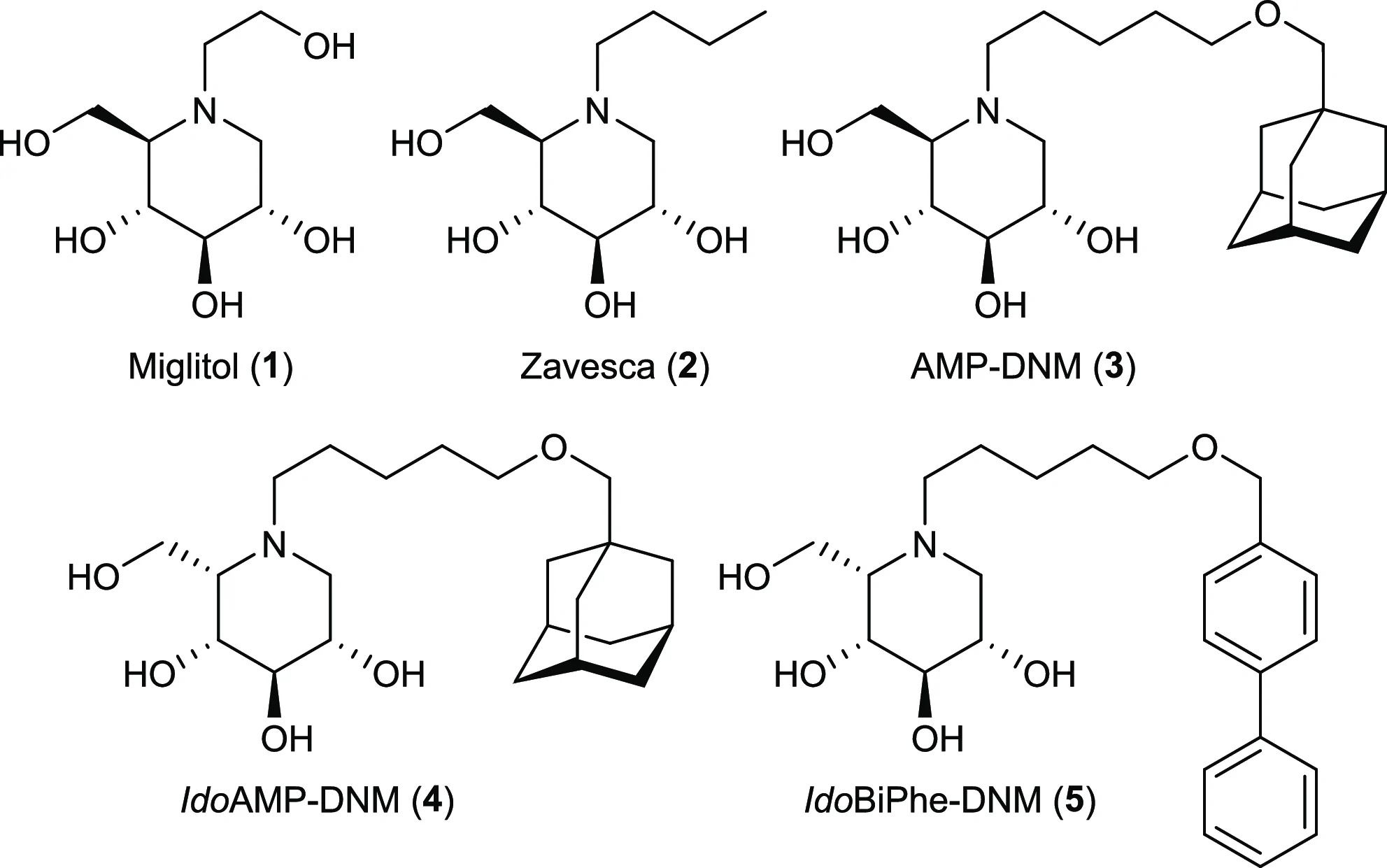
Deoxynojirimycin derivatives in clinical use for the treatment of type 1 diabetes (Miglitol, **1**) and Gaucher disease (Zavesca, **2**) and *N*-alkyl iminosugars **3–5** from our previous studies on glucosylceramide metabolism modulation.

Our research in the past two decades^6^ has focused on the design of dual GCS/nonlysosomal glucosylceramidase (GBA2) inhibitors with the aim of correcting aberrant glucosylceramide levels that are the consequence of genetically impaired lysosomal glucosylceramidase (glucocerebrosidase, GBA1) activity that characterizes Gaucher disease; both non-neuronopathic (type 1) and neuronopathic (type 2/3) disease variants. Our first report^6a^ in this context detailed the discovery of *N*-(adamantanemethyloxypentyl)-deoxynojirimycin (AMP-DNM, **3**), followed by a report^6b^ on the discovery of *N*-(adamantanemethyloxypentyl)-L-*Ido*-deoxynojirimycin (*Ido*AMP-DNM, **4**), and most recently^6d^ on *N*-(biphenylmethyloxypentyl)-L-*Ido*-deoxynojirimycin (*Ido*BiPhe-DNM, **5**). Our work on *N*-alkyl-*DNM* derivatives is complemented by a large body of work from other groups^7^ that have pursued related compounds in the context of a variety of human pathologies that involve glycoprocessing enzymes. These disease areas include, besides Gaucher disease and type 2 diabetes, a range of inherited glycosphingolipidoses, cancer, and (viral) infectious diseases.

Compounds **3–5** outperform Zavesca (**2**) in potency as GCS inhibitors and are also very potent GBA2 inhibitors.^6^ L-*Ido*-configured iminosugars **4** and **5** moreover do not inhibit intestinal digestive glucosidases and neither ER α-glucosidase II, whereas AMP-DNM **3**, as well as Zavesca **2**, have these enzymes as (biomedically unfavorable) off-targets. Compounds **1–3** are *N*-alkyl derivatives of the archetypal iminosugar, deoxynojirimycin (DNM), whereas compounds **4** and **5** are derived from the C5-epimeric, L-*ido*-congener, L-*ido*-DNM. Compounds **4** and **5** are, in contrast to **1–3**, relatively poor inhibitors of the Gaucher enzyme, GBA1, and are therefore, in our view, the more attractive compounds for correcting glucosylceramide levels in Gaucher patients.

DNM features four chiral centers and lacks a symmetry plane, resulting in 16 possible stereoisomeric forms of DNM. These stereoisomers emulate the configurations of the eight classical D-aldoses and L-aldoses combined. All these DNM stereoisomers have in the past decades been procured, as natural products, by chemical synthesis, or both, and through the work of many research groups worldwide. However, they have not been assembled in a single, comprehensive physical library for use in screening campaigns—the work of Asano and colleagues^8^ on the construction of 14 stereoisomers and their evaluation on a panel of glycohydrolases comes closest. We decided to make the effort to synthesize all 16 DNM stereoisomers.^9^ Also, and in line with the general observation that *N*-alkyl-DNM derivatives feature beneficial properties (enzyme inhibition activity, bioavailability) compared to their unsubstituted counterparts, we prepared seven *N*-alkyl derivatives for each configurational DNM analogue. As alkyl substituents, we selected those already part of clinical drugs (hydroxyethyl, as in Miglitol **1** ^2^; butyl, as in Zavesca **2** ^3^), along with those favored in our earlier studies (adamantanemethyloxypentyl, as in **3** and **4**;^6a,6b^ biphenylmethyloxypentyl, as in **5** ^6d^). Additionally, we included some linear and branched alkyl chains, some of which have been utilized in pharmacological chaperones designed to stabilize the folds of genetically disabled glycohydrolases. As part of the larger *N*-alkyl chains, we included an oxygen (an ether, five carbons away from the iminosugar nitrogen), as unmodified large linear alkyl substituents tend to be detrimental to solubility and enzyme inhibition activity. In total, our DNM library encompasses the 16 DNM configurations bearing eight (proton or one of seven alkyl chains) substituents on the nitrogen, thus 128 entries. Data on their synthesis and evaluation as inhibitors of the glucosylceramide processing enzymes, GCS, GBA1 and GBA2, are presented in this report.

## Results

The preparation of the configurational DNM isomers and their *N*-alkylation follows routes of synthesis previously developed by us^6,9^ or others,^7,8,10^ and details on syntheses not previously reported are given in the Supporting Information. The preparation of L-*Alt*-DNM requires some extra attention, since we had reported on this entry in one of our previous report,^6c^ where we showed no activity of *N*-alkyl-L-*Alt*-DNM derivatives against either of the three glucosylceramide processing enzymes, GCS, GBA1 and GBA2. After scrutinizing our results and developing an alternative route of synthesis^9a^ for L-*Alt*-DNM (a route we also applied for the synthesis of enantiomeric D-*Alt*-DNM) we had to conclude that we originally prepared D-*gluco*-pyrrolidine **12** (Scheme 1). In our earlier reported work,^6c^ we started out with galactose, and Fischer glycosylation with methanol should have yielded α-D-galactopyranoside. Perbenzylation and hydrolysis of the anomeric acetal should then return tetra-*O*-benzylgalactopyranose **7** that in four steps (reduction of the hemiacetal to the diol, mesylation of the primary and secondary alcohols to the bismesylate that subsequently should have been displaced by allylamine to afford fully protected piperidine **8** and subsequent two-step global deprotection) should have yielded L-*Alt*-DNM **9** (Scheme 1A). Instead, we likely misapplied conditions and did not obtain the thermodynamic Fischer product **7** but instead the kinetic product, D-galactofuranoside. From here, the same sequence of steps (perbenzylation and acetal hydrolysis to give **10**, followed by hemiacetal reduction, bismesylation, substitution and deprotection) gave the pyrrolizidine iminosugar with inversion of configuration at C4 of the parent sugar (galactose), thus D-*Glc*-pyrrolidine **12** (Scheme 1A). We previously^9b^ utilized hydroxynitrilase chemistry to prepare all eight 3,4-*cis*-DNM derivatives, thus half of the complete set reported here, and that included L-*Alt*-DNM **9**, and comparison of the analytical data with that of the earlier publication made us realize we evaluated *N*-alkyl-D-*Glc*-pyrrolidine iminosugars on their GCS/GBA1/GBA2 inhibitory potential. For clarity, our route to L-*Alt*-DNM **9** is recapitulated in Scheme 1B.^9b^ It commences with partial, DIBAL-H-mediated reduction of protected hydroxynitrile **13**, followed by transimination using **14** and subsequent NaBH_4_ reduction to afford secondary amine **15**. *N*-Boc-protection, followed by ring-closing metathesis and dihydroxylation of the resultant dehydropiperidine **16** gave in good yield L-*Alt*-piperidine **17**, which upon global deprotection afforded L-*Alt*-DNM **9**.

**Scheme 1:**
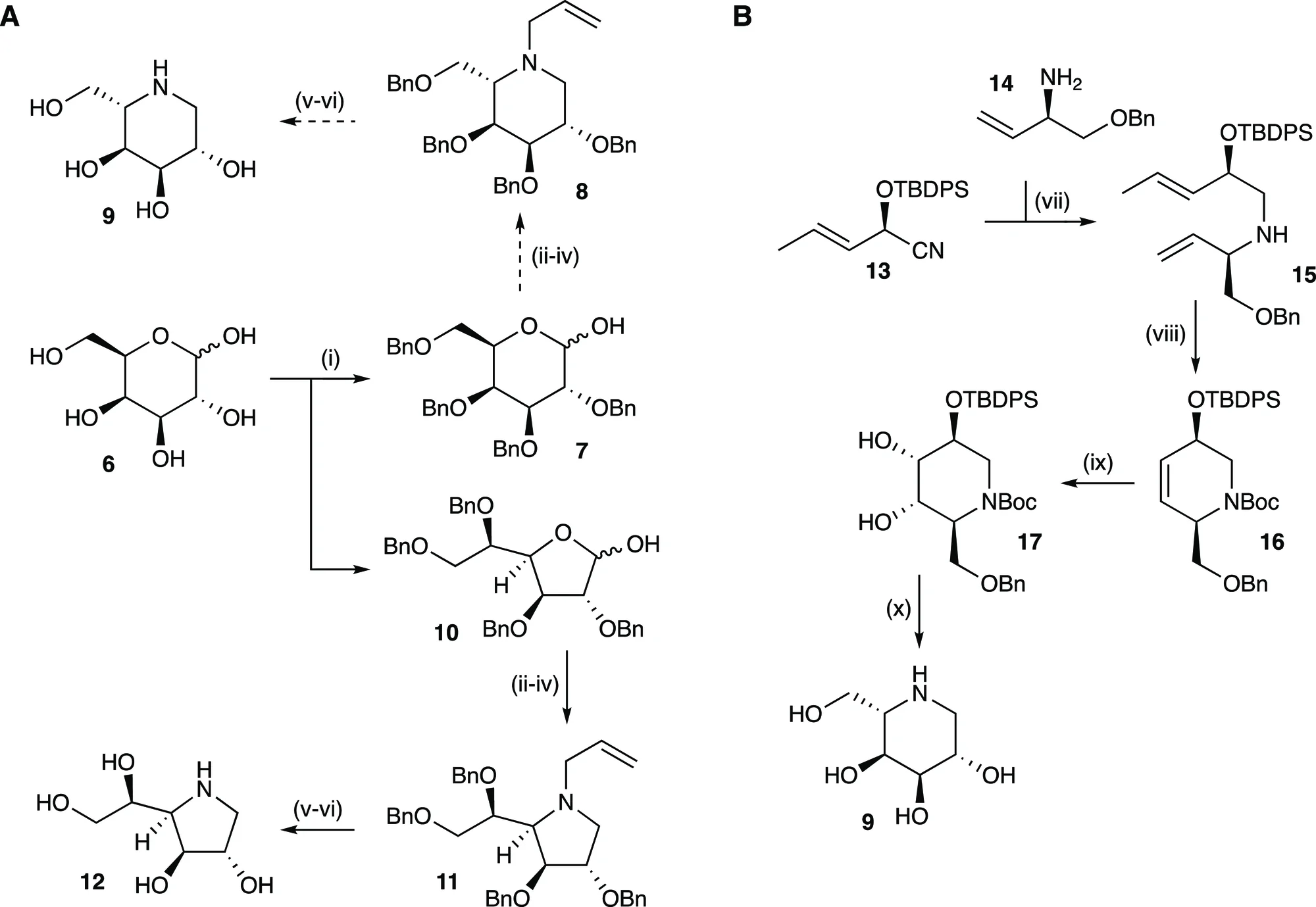
Reagents and Conditions: (i) MeOH, pTsOH, then BnBr, NaH, DMF, then Aqueous HCl; (ii) LiAlH_4_, THF; (iii) MsCl, Et_3_N, CH_2_Cl_2_; (iv) Allylamine; (v) KO^*t*^Bu, DMSO, 100 °C; (vi) BCl_3_, CH_2_Cl_2_; (vii) DiBAL-H, MeOH, then **14**, NaBH_4_; (viii) Boc_2_O, Et_3_N, CH_2_Cl_2_; then Grubbs I; CH_2_Cl_2_; (ix) K_2_OsO_4_; (x) TBAF, THF, then H_2_, Pd/C, HCl

The half-maximal inhibitory concentration (IC_50_) values of the 128-compound library as inhibitors of GCS, GBA1, and GBA2 were then determined and are presented in Tables 1 (D-DNM series) and 2 (L-DNM series). Enzyme inhibition assays were performed as reported previously, in which we assessed GCS inhibition potency up to 50 μM final compound concentration, GBA1 to 1000 μM, and GBA2 up to 100 μM.^11^ These final inhibition concentrations were chosen based on the differences in limitations in assay formats (GCS and GBA2 inhibition assays are done on cell lysates, as opposed to GBA1 assays, which are performed on recombinant, pure enzyme) and also general activity windows, with GBA2 inhibition often in the (sub)­nanomolar range. From these data, it can be concluded that the intrinsic potential to inhibit one of the three glycoprocessing enzymes is determined by the configuration of the DNM derivative.

**Table 1:**
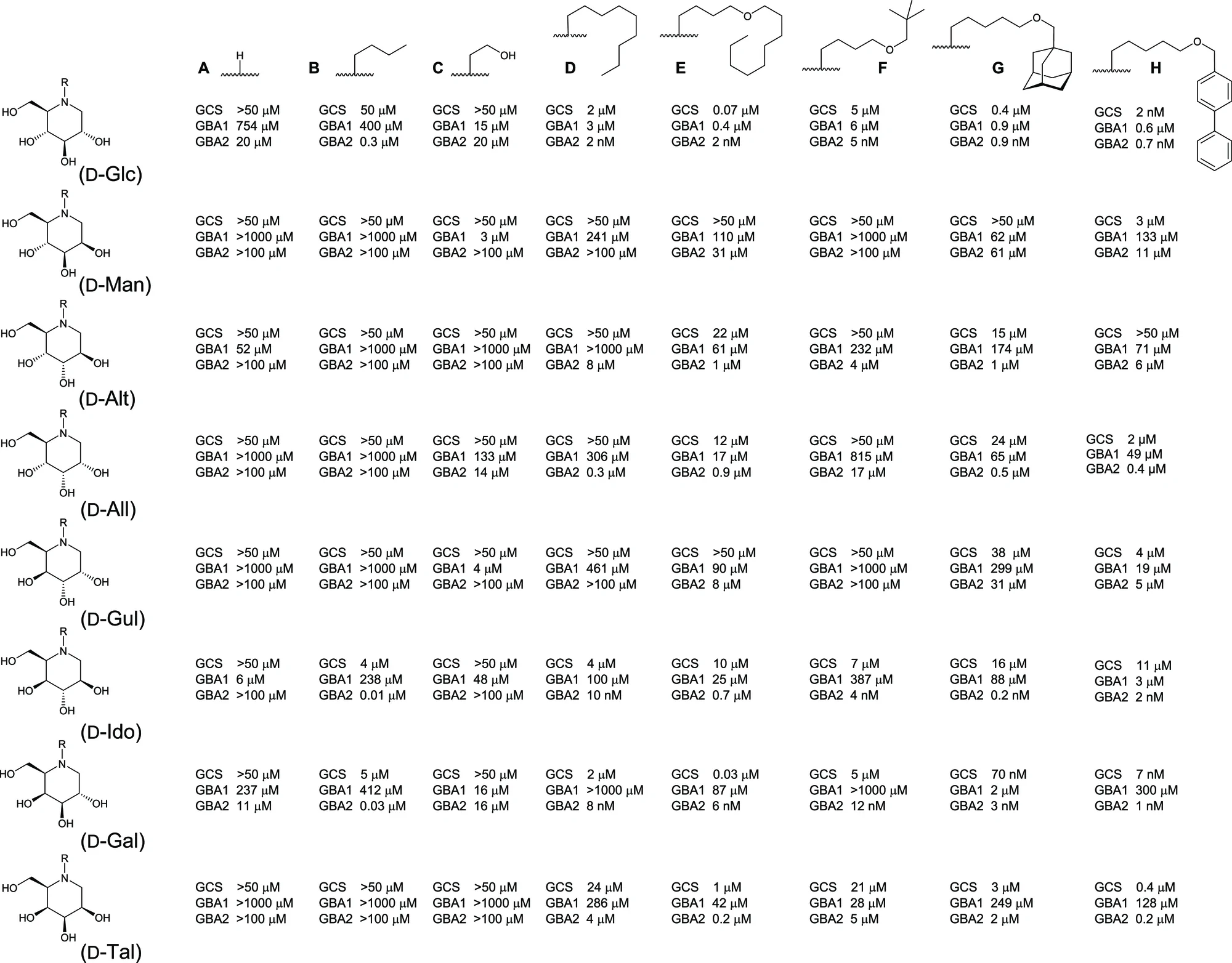
Inhibition of GCS, GBA1, and GBA2—the D-DNM Series

**Table 2:**
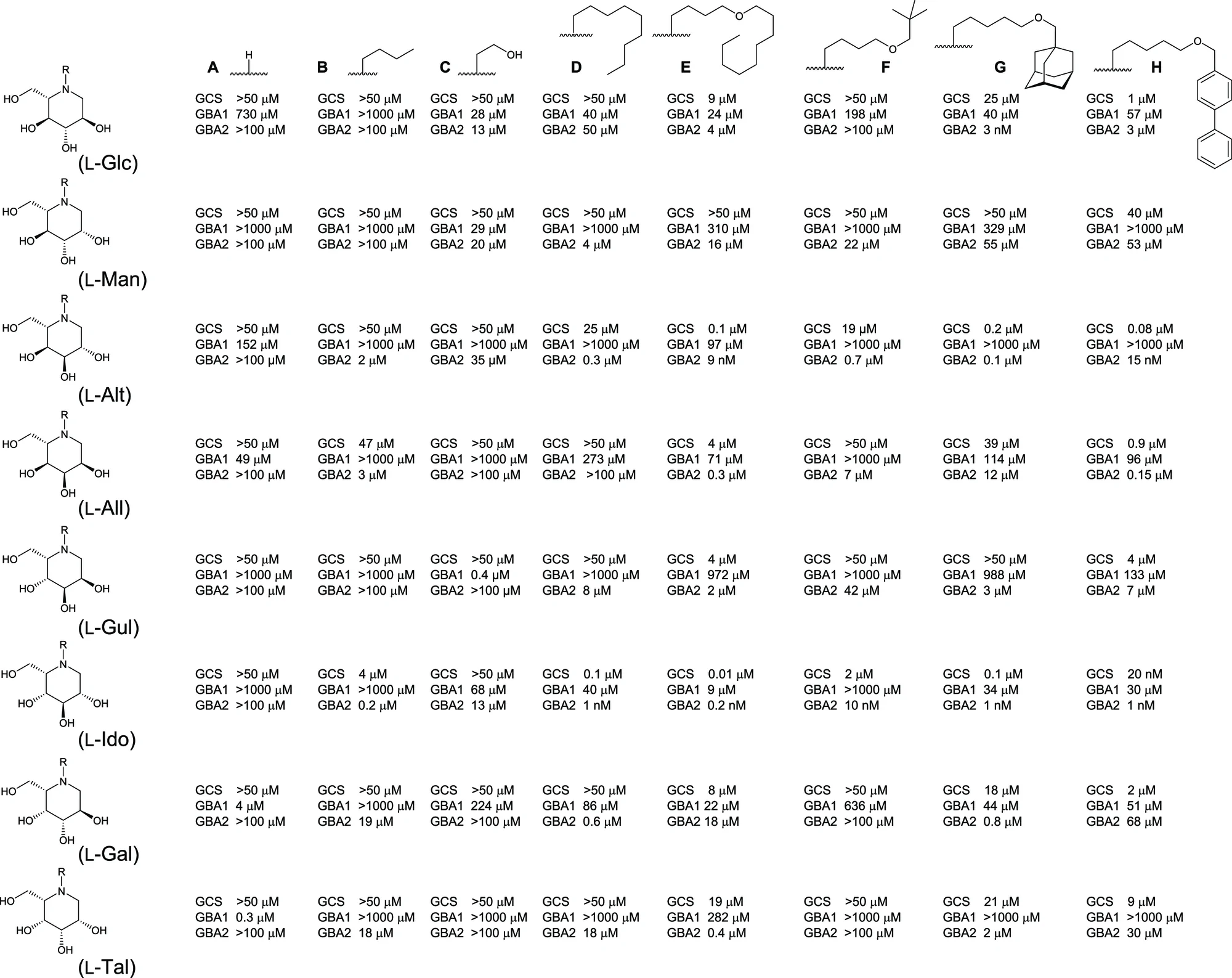
Inhibition of GCS, GBA1, and GBA2—the L-DNM Series

Potency is modulated by the nature of the *N*-alkyl chain, aligning with our general observation^6^ that the bulkier the substituents, the more potent the inhibitors for these enzymes. Among the three enzymes, GBA2 appears to be least sensitive toward DNM configuration, and the most easily inhibited. In contrast, GBA1 seems the pickiest of the three, with only the D-*Glc*-DNM and D-*Gal*-DNM series yielding single-digit micromolar inhibitors among the 16 configurations. GCS selectivity lies somewhat between that of GBA1 and GBA2. Besides D-*Glc*-DNM, D-*Gal*-DNM and L-*Ido*-DNM, L-*Alt*-DNM derivatives are among the most potent GCS inhibitors. The latter two also inhibit GBA2, although they are less effective against GBA1. This behavior, previously reported for L-*Ido*-DNM derivatives,^6^ highlights their potential as leads for further development toward therapeutics for neuronopathic lysosomal storage disorders including type 2/3 Gaucher disease. Although L-*Alt*-DNM derivatives are slightly less potent GCS and GBA2 inhibitors than their L-*Ido*-counterparts (with the same *N*-alkyl substituent), their negligible activity against GBA1—an enzyme to avoid targeting in a clinical setting—makes them particularly promising leads for further development.

## Discussion and Conclusions

At the onset of the work described in this paper, we had compiled around 12 configurational DNM isomers, equipped with a variety (but not a comprehensive set) of *N-*alkyl substituents.^6^ We realized that the construction of all 16 configurational analogues was within reach, by the preparation of the missing ones using our own synthesis methodologies (for instance, application of a route toward a D-DNM configurational isomer to prepare the corresponding l-isomer) or alternatively through existing literature procedures.^7,10^ We also realized that such a comprehensive DNM library did not exist, at least in the public domain. The closest work was the 14 configurational isomers reported^8c^ by Asano in 2005, but this study did not include *N*-alkyl diversification. The result of our efforts is the preparation of such a comprehensive library, having in total 128 entries. These were assessed as potential inhibitors of the three glucosylceramide metabolizing enzymes, GCS, GBA1 and GBA2. In doing so, we found that our earlier report^6c^ on *N*-alkyl-L-*Alt*-DNMs was at fault, and we set the record straight here. We also found that *N*-alkyl-L-*Alt*-DNMs are almost as good as the corresponding *N*-alkyl-L-*Ido*-DNMs as selective, dual GCS/GBA2 inhibitors, which may be of value for the development of new therapeutics for the treatment of neuronopathic (type 2/3) Gaucher disease. One of the driving forces behind our decision to assemble the here-presented library, encompassing all 16 DNM configurations, was our analysis that the rational design of configurational DNM isosteres targeting GCS, GBA1 and/or GBA2 selectively is currently hampered by the lack of structural data. Although X-ray structures of human GBA1 exist, also complexed to the clinical drug, miglustat,^12^ human GBA2 has to date defied structural studies, though the structure of a bacterial homologue (*Tx*GH116) has been published.^13^ In contrast, no X-ray structure of human GCS and neither that of a homologue has been reported, and no information on ligand/inhibitor binding to this glycosyltransferase is available. Perusal of the inhibitory potency and selectivity of our 128-compound library allows us to draw some general conclusions. Within each configurational series, compounds bearing large hydrophobic *N*-substituents outcompete their parent compound as well as short (butyl, hydroxyethyl) congeners in potency toward all three enzymes. This is in line with the amphiphilic nature, featuring a hydrophobic aglycon, of the substrate (glucosylceramide) that is produced (GCS) or processed (GBA1, GBA2) by these enzymes. Also apparent is the general trend that GBA1 is much more sensitive to DNM configuration than GBA2 and (to a lesser extent) GCS. This is fortunate in terms of designing effective substrate reduction therapies, in which ideally residual GBA1 activity as caused by genetic mutations is left unhampered—a finding that is at the basis of our previous designs of L-*Ido*-configured DNMs and that also makes the L-*Alt*-DNMs reported here attractive new candidates for the development of such therapies. Further research into this, besides obtaining X-ray (or AlphaFold) structures of human GCS and GBA2 complexed to our inhibitors, may involve calculating the conformational space of the individual piperidines, also taking into consideration the recent finding^14^ that *Tx*GH116, and by extension likely also human GBA2, features an active site architecture different from that of GBA1 with the catalytic acid–base residue positioned perpendicular to the aglycon, instead of in an antiarrangement. Beyond the glycoprocessing enzymes of our own interest, which are in the context of Gaucher disease and related lysosomal storage disorders, we believe that our comprehensive iminosugar library, which complements compound series we had prepared and reported previously (eight configurational fagomine isosters,^15^ among others) may hold attractive starting points for the development of inhibitors or pharmacological chaperones for other glycoprocessing enzymes, featuring in other human pathologies; besides lysosomal storage disorders also cancer and infectious diseases. The series of *N*-alkyl derivatives allows for exploring inhibitory potency for such enzymes. We realize that not all glycoprocessing enzymes favor bulky, apolar *N*-alkyl chains, and our series also encompasses smaller and more hydrophilic ones. We believe that screening of our compound collection as inhibitors of glycoprocessing enzymes other than the ones presented here may yield valuable lead inhibitors in the context of a variety of human diseases.^16^

## Experimental Section

### General Experimental Details

All reagents were of experimental grade and were used without further purification unless stated otherwise. Dichloromethane (DCM) and tetrahydrofuran (THF) were stored over 3 Å molecular sieves, and *N,N*′-dimethylformamide (DMF) was stored over 4 Å molecular sieves. All reactions were performed under an argon or N_2_ atmosphere unless stated otherwise. Reactions were monitored by reversed phase LC-MS (details below) or analytical thin layer chromatography (TLC) using Merck aluminum sheets precoated with silica gel 60 with detection by UV-absorption (254 nm) and by spraying with a solution of (NH_4_)_6_Mo_7_O_24_·H_2_O (25 g/L) and (NH_4_)_4_Ce­(SO_4_)_4_·H_2_O (10 g/mL) in 10% sulfuric acid followed by charring at 150 °C or by spraying with an aqueous solution of KMnO_4_ (7%) and K_2_CO_3_ (2%) followed by charring at 150 °C. Column chromatography was performed manually using Screening Device b.v. silica gel 60 (0.04–0.063 mm) using the indicated solvents. LC-MS analysis was performed on a LCQ Advantage Max (Thermo Finnigan) ion-trap spectrometer (ESI+) coupled to a Surveyor HPLC system (Thermo Finnigan) equipped with a C18 column (Gemini, 4.6 × 50 mm, 5 μM particle size, Phenomenex). The applied buffers were H_2_O, acetonitrile (MeCN), and 1% aqueous trifluoroacetic acid (TFA). ^1^H NMR and ^13^C NMR spectra were recorded on Bruker AV-400 (400/101 MHz), Bruker AV-500 (500/126 MHz), and Bruker AV-600 (600/151 MHz) spectrometers in the given solvent. Chemical shifts (δ) are given in ppm relative to tetramethylsilane (TMS) as internal standard (^1^H NMR in CDCl_3_) or the residual signal of the deuterated solvent. Coupling constants (*J*) are given in Hz. All given ^13^C NMR spectra are proton-decoupled. High-resolution mass spectrometry (HRMS) analysis was performed with a LTQ Orbitrap mass spectrometer (Thermo Finnigan), equipped with an electronspray ion source in positive mode (source voltage 3.5 kV, sheath gas flow 10 mL/min, capillary temperature 250 °C) with resolution *R* = 60,000 at *m*/*z* 400 (mass range *m*/*z* = 150–2000) and dioctyl phthalate (*m*/*z* = 391.28428) as a “lock mass”. The high-resolution mass spectrometer was calibrated prior to measurements with a calibration mixture (Thermo Finnigan).

### Iminosugar Synthesis

The iminosugars were synthesized, as reported in the literature. For D-*Glc*-DNM and L-*Ido-*DNM, see ref 6c; for D-*All-*DNM, D-*Gal-*DNM, and L-*Alt-*DNM, see ref 9a; for D-*Alt-*DNM, D-*Tal-*DNM, L-*All-*DNM, L-*Gal-*DNM, and L-*Tal-*DNM, see ref 9b; for D-*Man-*DNM, D-*Gul-*DNM, D-*Ido-*DNM, L-*Man-*DNM, and L-*Gul-*DNM, see ref 8; for L-*Glc-*DNM, see ref 6d.^6d^

### General Procedure A: Iminosugar *N*-alkylation

This method was used for all *N*-alkyl-1-deoxynojirimycins except for the *N*-hydroxyethyl derivatives (the miglitol analogues; these were synthesized using the General Procedure B). The appropriate 1-deoxynojirimycin hydrochloride (1 equiv) was dissolved in DMF (0.2 M), and after addition of the base (3 equiv; DIPEA or K_2_CO_3_) and the appropriate alkyl bromide^6b,6d,6e^ (2 equiv), the mixture was stirred at 80 °C overnight. After cooling to room temperature and filtration, the reaction mixture was concentrated and purified by RP-HPLC to afford the products.

### General Procedure B: Synthesis of *N*-Hydroxyethyl-1-deoxynojirimycins

Step one: The appropriate iminosugar as the hydrochloride salt (1 equiv) was dissolved in MeOH (0.15 M), and after addition of triethylamine (2 equiv), acetic acid (3 equiv), and benzyloxyacetaldehyde (5 equiv), the reaction mixture was stirred for 20 min at room temperature. Subsequently, NaCNBH_3_ (4 equiv) was added, and the purple/black reaction mixture was stirred until it was almost colorless, and LC-MS analysis showed complete conversion. Next, Celite (roughly 15 mg per mg iminosugar) was added, and the volatiles were evaporated *in vacuo*, followed by silica gel column chromatography (CHCl_3_:MeOH:0.5 M NH_3_ in MeOH 1:0:0 → 95:5:0 → 80:0:20) to afford the semipure *N*-benzyloxyethyl iminosugar, which was further purified by RP-HPLC. Step two: The product from step one was dissolved in MeOH (0.1 M), and trifluoroacetic acid (TFA, 2 equiv) was added. The mixture was purged with nitrogen for 5 min, after which Pd/C-10% (25% w/w with respect to the substrate) was added. The flask was sealed with a septum, and a balloon of hydrogen was placed on top. The mixture was stirred vigorously for 24 to 48 h, and after filtration over a Whatman filter, the solvent was evaporated. Purification by RP-HPLC afforded the target compounds.

### HPLC Conditions to Purify the Final Products

(Buffer A: 0.2% TFA in water, Buffer B: acetonitrile). *N*-butyl-deoxynojirimycins: Gemini C18 column (5 mm, 110A, 10 × 250 mm), 5 mL/min, 0–8% B in A; 12 min. *N*-2′-benzyloxyethyl-deoxynojirimycins: Gemini C18 column (5 mm, 110A, 10 × 250 mm), 5 mL/min, 8–12% B in A; 12 min. *N*-2′-hydroxyethyl-deoxynojirimycins: Gemini C18 column (5 mm, 110A, 10 × 250 mm), 5 mL/min, 0–5% B in A; 12 min. *N*-nonyl-deoxynojirimycins: Gemini C18 column (5 mm, 110A, 21.2 × 250 mm), 25 mL/min, 25–40% B in A; 10 min. *N*-5′-nonyloxypentyl-deoxynojirimycins: Gemini C18 column (5 mm, 110A, 10 × 250 mm), 5 mL/min, 30–45% B in A; 12 min. *N*-5′-neopentyloxypentyl-deoxynojirimycins: Gemini C18 column (5 mm, 110A, 21.2 × 250 mm), 25 mL/min, 20–30% B in A; 10 min. *N*-5′-adamantanemethoxypentyl-deoxynojirimycins: Gemini C18 column (5 mm, 110A, 21.2 × 250 mm), 25 mL/min, 30–40% B in A; 10 min. *N*-5′-(1,1′-biphenyl-4-ylmethoxy)­pentyl-l-deoxynojirimycins: Gemini C18 column (5 mm, 110A, 21.2 × 250 mm), 25 mL/min, 30–40% B in A; 10 min. All compounds were obtained in ≥95% (unless stated otherwise) purity after HPLC purification as determined by HPLC-MS using UV- and/or TIC-intensity.

### For NMR Characterization, Carbohydrate Numbering is Used

#### *N*-(5-([1,1′-Biphenyl]-4-ylmethoxy)­pentyl)-D-*Man*-DNM Trifluoroacetic Acid Salt

Prepared in 57% yield following general procedure A (0.391 mmol scale). ^1^H NMR (400 MHz, D_2_O) δ 7.14–7.02 (m, 4 H, Ar), 6.93 (d, *J* = 8.0 Hz, 2H, Ar), 6.87 (t, *J* = 7.5 Hz, 2H, Ar), 6.79 (t, *J* = 7.3 Hz, 1H, Ar), 4.08 (s, 2H, CH_2_–6′), 3.99 (s, 1H, H-2), 3.88 (d, *J* = 12.8 Hz, 1H, H-6a), 3.81 (t, *J* = 9.9 Hz, 1H, H-4), 3.68 (d, *J* = 12.8 Hz, 1H, H-6b), 3.44 (d, *J* = 8.6 Hz, 1H, H-3), 3.17 (d, *J* = 11.2 Hz, 1H, H-1a), 3.07 (t, *J* = 6.3 Hz, 2H, H-5′), 3.02–2.91 (m, 3H, CH_2_–1′, H-1b), 2.77 (d, *J* = 9.8 Hz, 1H, H-5), 1.37–1.22 (m, 4H, CH_2_–2′, CH_2_–4′), 1.07–0.96 (m, 2H, CH_2_–3′). ^13^C NMR (101 MHz, D_2_O) δ 139.9, 139.6, 137.0 (*C*_q_ Ar), 128.7, 128.4, 127.2, 126.52, 126.48 (Ar), 72.1 (C-3), 71.8 (C-6′), 69.5 (C-5′), 65.6 (C-5), 65.4 (C-2), 64.9 (C-4), 54.6 (C-1), 54.1 (C-6), 52.7 (C-1′), 28.2 (C-4′), 22.5 (C-3′), 21.6 (C-2′). HRMS calcd for [C_24_H_34_NO_5_]^+^ 416.24315, found 416.24312.

#### *N*-2-(Benzyloxy)­ethyl-D-*Alt*-DNM Trifluoroacetic Acid Salt

Prepared in 49% yield following general procedure B, step one (0.375 mmol scale). ^1^H NMR (400 MHz, D_2_O) δ 7.99–7.10 (m, 5H, Ar), 4.60 (s, 2H, CH_2_–3′), 4.14 (d, *J* = 7.9 Hz, 1H, H-2), 4.12–4.05 (m, 1H, H-3), 4.05–3.94 (m, 3H, H-4, CH_2_–6), 3.91 (m, 2H, CH_2_–2′), 3.67–3.40 (m, 4H, H-1a, H-5, CH_2_–1′), 3.33 (d, *J* = 14.1 Hz, 1H, H-1b). ^13^C NMR (101 MHz, D_2_O) δ 136.8 (*C*_q_ Ar), 128.8, 128.5 (Ar), 73.0 (C-3′), 67.9 (C-4), 65.8 (C-2), 63.0 (C-3), 62.7 (C-2′), 62.1 (C-5), 54.1 (C-6), 52.3 (C-1′), 52.0 (C-1). HRMS calcd for [C_15_H_24_NO_5_]^+^ 298.16490, found 298.16491.

#### *N*-(2′-Hydroxyethyl)-D-*Alt*-DNM Trifluoroacetic Acid Salt

Prepared in 52% yield following general procedure B, step two (0.109 mmol scale). ^1^H NMR (850 MHz, D_2_O, 333 K) δ 4.20 (dd, *J* = 8.8, 2.7 Hz, 1H, H-4), 4.17 (td, *J* = 4.9, 2.7 Hz, 1H, H-3), 4.06 (d, *J* = 3.1 Hz, 2H, CH_2_–6), 4.02–3.95 (m, 3H, H-2, CH_2_–1′), 3.63–3.53 (m, 2H, H-1a, CH_2_–2′a), 3.51 (broad s, 1H, H-5), 4.11–3.92 (m, 2H, H-1b, CH_2_–2′b). ^13^C NMR (214 MHz, D_2_O, @333 K) δ 68.7 (C-2), 65.9 (C-3), 63.2 (C-4, C-5), 55.4 (C-1′), 54.9 (C-6), 54.7 (C-2′), 51.9 (C-1). HRMS calcd for [C_8_H_18_NO_5_]^+^ 208.11795, found 208.11787.

#### *N*-(5-(Nonyloxy)­pentyl)-D-*Alt*-DNM Trifluoroacetic Acid Salt

Prepared in 56% yield following general procedure A (0.391 mmol scale). ^1^H NMR (400 MHz, D_2_O) δ 4.18–4.06 (m, 2H, H-2, H-4), 4.06–3.88 (m, 3H, H-3, CH_2_–6), 3.51–3.29 (m, 6H, H-1a, H-5, CH_2_–5′, CH_2_–6′), 3.28–3.15 (m, 3H, H-1b, CH_2_–1′), 1.81–1.64 (m, 2H, CH_2_–2′), 1.63–1.46 (m, 4H, CH_2_–4′, CH_2_–7′), 1.43–1.32 (m, 2H, CH_2_–3′), 1.31–1.15 (m, 12H, CH_2_–8′–13′), 0.84 (t, *J* = 6.3 Hz, 3H, CH_3_-14′). ^13^C NMR (101 MHz, D_2_O) δ 70.8, 70.4 (C-5′, C-6′), 68.4 (C-2), 66.0 (C-4), 63.6 (C-3), 61.8 (C-5), 54.5 (C-6), 52.9 (C-1′), 51.3 (C-1), 31.9, 29.7, 29.5, 29.3, 29.3, 28.6, 26.0, 22.8, 22.6 (9 × CH_2_), 22.3 (C-2′), 13.8 (C-14′). HRMS calcd for [C_20_H_42_NO_5_]^+^ 376.30575, found 376.30580.

#### *N*-(5-(Neopentyloxy)­pentyl)-D-*Alt*-DNM Trifluoroacetic Acid Salt

Prepared in 60% yield following general procedure A (0.333 mmol scale). ^1^H NMR (400 MHz, D_2_O) δ 4.26–4.05 (m, 2H, H-2, H-4), 4.04–3.89 (m, 3H, H-3, CH_2_–6), 3.52–3.43 (m, 3H, H-1a, CH_2_–5′), 3.34–3.20 (m, 4H, H-1b, H-5, CH_2_–1′), 3.13 (s, 2H, CH_2_–6′), 1.82–1.64 (m, 2H, CH_2_–2′), 1.63–1.51 (m, 2H, CH_2_–4′), 1.45–1.31 (m, 2H, CH_2_–3′), 0.83 (s, 9H, C­(CH_3_)_3_). ^13^C NMR (101 MHz, D_2_O) δ 81.1 (C-6′), 71.0 (C-5′), 68.0 (C-2), 66.0 (C-4), 62.8 (C-3), 61.1 (C-5), 54.0 (C-6), 53.0 (C-1′), 51.3 (C-1), 31.2 (*C*_q_ *t*Bu), 27.8 (C-4′), 25.9 (CH_3_)_3_, 22.5 (C-3′), 21.6 (C-2′). HRMS calcd for [C_16_H_34_NO_5_]^+^ 320.24315, found 320.24332.

#### *N*-2-(Benzyloxy)­ethyl-D-*All*-DNM Trifluoroacetic Acid Salt

Prepared in 43% yield following general procedure B, step one (0.250 mmol scale). ^1^H NMR (400 MHz, MeOD) δ 7.54–7.17 (m, 5H, Ar), 4.53 (s, 2H, CH_2_–3′), 4.07–3.99 (m, 2H, H-3, H-6a), 3.93 (dd, *J* = 10.6, 2.0 Hz, 1H, H-6b), 3.88–3.78 (m, 3H, H-2, CH_2_–2′), 3.75 (d, *J* = 10.4 Hz, 1H, H-4), 3.58 (d, *J* = 14.0 Hz, 1H, H-1′a), 3.46 (d, *J* = 14.0 Hz, 1H, H-1′b), 3.37–3.18 (m, 3H, CH_2_–1, H-5). ^13^C NMR (101 MHz, MeOD) δ 139.9 (*C*_q_ Ar), 130.9, 130.4, 130.4 (Ar), 75.7 (CH_2_Ph), 72.6 (C-3), 67.8 (C-4), 66.9 (C-2), 65.9 (C-6), 64.3 (C-5), 56.2 (C-2′), 55.2 (C-1′), 52.6 (C-1). HRMS calcd for [C_15_H_24_NO_5_]^+^ 298.16490, found 298.16517.

#### *N*-(2′-Hydroxyethyl)-D-*All*-DNM Trifluoroacetic Acid Salt

Prepared in 66% yield following general procedure B, step two (0.332 mmol scale). ^1^H NMR (400 MHz, D_2_O) δ 4.12 (t, 1H, *J* = 2.7 Hz, H-3), 4.06–3.90 (m, 5H, CH_2_–6, H-2, CH_2_–2′), 3.87 (d, *J* = 11.3 Hz, 1H, H-4), 3.65–3.47 (m, 1H, CH_2_–1′a), 3.41 (dd, *J* = 11.7, 4.7 Hz, 1H, H-1a), 3.38–3.28 (m, 2H, H-5, H-1′b), 3.24 (t, *J* = 11.7 Hz, 1H, H-1b). ^13^C NMR (101 MHz, D_2_O) δ 69.4 (C-3), 64.7 (C-4), 63.7 (C-2), 61.4 (C-5), 55.2 (C-1′ & C-2′), 53.8 (C-6), 49.2 (C-1). HRMS calcd for [C_8_H_18_NO_5_]^+^ 208.1180, found 208.1182.

#### *N*-2-(Benzyloxy)­ethyl-D-*Gul*-DNM Trifluoroacetic Acid Salt

Prepared in 13% yield following general procedure B, step one (0.240 mmol scale). ^1^H NMR (600 MHz, MeOD) δ 7.48–7.18 (m, 5H, Ar), 4.59 (s, 2H, CH_2_–3′), 4.24–4.14 (m, 1H, H-2), 4.09 (dd, *J* = 4.3, 1.9 Hz, 1H, H-4), 4.01 (dd, *J* = 12.8, 4.2 Hz, 1H, H-6a), 3.93 (dd, *J* = 12.6, 4.2 Hz, 1H, H-6b), 3.91–3.86 (m, 2H, CH_2_–2′), 3.85–3.73 (m, 1H, H-3), 3.65–3.45 (m, 3H, H-5, CH_2_–1′), 3.40 (t, *J* = 11.6 Hz, 1H, H-1a), 3.26 (dd, *J* = 11.8, 4.8 Hz, 1H, H-1b). ^13^C NMR (151 MHz, MeOD) δ 138.3 (*C*_q_ Ar), 129.2, 128.7 (Ar), 74.0 (C-3′), 72.1 (C-4), 69.7 (C-3), 64.2 (C-2′), 63.8 (C-2), 62.0 (C-5), 60.5 (C-6), 54.2 (C-1′), 51.9 (C-1). HRMS calcd for [C_15_H_24_NO_5_]^+^ 298.1654, found 298.1650.

#### *N*-(2′-Hydroxyethyl)-D-*Gul*-DNM Trifluoroacetic Acid Salt

Prepared in 43% yield following general procedure B, step two (0.030 mmol scale). ^1^H NMR (600 MHz, MeOD) δ 4.22 (m, 1H, H-2), 4.09–4.04 (m, 1H, H-4), 4.04–3.82 (m, 5H, H-3, CH_2_–6, CH_2_–2′), 3.62–3.54 (m, 1H, H-5), 3.50–3.48 (m, 1H, H-1a), 3.42–3.27 (m, 3H, H-1b, CH_2_–1′). ^13^C NMR (151 MHz, MeOD) δ 72.3 (C-4), 70.0 (C-3), 64.1 (C-2), 62.6 (C-5), 61.0 (C-6), 56.8 (C-2′), 56.6 (C-1′), 52.1 (C-1). HRMS calcd for [C_8_H_18_NO_5_]^+^ 208.1185, found 208.1179.

#### *N*-(2′-Hydroxyethyl)-D-*Ido*-DNM Trifluoroacetic Acid Salt

Prepared in 30% yield from *N*-2-(benzyloxy)­ethyl-D-*ido*-DNM^6e^ following general procedure B, step two (0.060 mmol scale). ^1^H NMR (400 MHz, MeOD) δ 4.15 (br s, 1H, H-3), 4.13–3.95 (m, 5H, CH_2_–6, CH_2_–2′a, H-2, H-4), 3.91 (dd, *J* = 11.9, 5.1 Hz, 1H, CH_2_–2′b), 3.78 (t, *J* = 5.0 Hz, 1H, H-5), 3.72 (d, *J* = 13.5 Hz, 1H, H-1a), 3.58–3.49 (m, 1H, H-1b), 3.46 (d, *J* = 13.3 Hz, 1H, H-1′a), 3.34 (dd, *J* = 13.4, 2.4 Hz, 1H, H-1′b). ^13^C NMR (101 MHz, MeOD) δ 71.9 (C-3), 69.1 and 68.6 (C-4), 68.2 and 67.8 (C-2), 64.7 (C-5), 60.8 and 60.6 (C-6), 58.1 (C-5), 56.5 (C-2′), 55.0 (C-1), 46.9 (C-1′). HRMS calcd for [C_8_H_18_NO_5_]^+^ 208.11795, found 208.11790.

#### *N*-(5-(Nonyloxy)­pentyl)-D-*Ido*-DNM Trifluoroacetic Acid Salt

Prepared in 20% yield following general procedure A (0.500 mmol scale). ^1^H NMR (400 MHz, MeOD) δ 3.85 (dd, *J* = 11.5, 4.8 Hz, 1H, H-6a), 3.81 (dd, *J* = 11.4, 6.4 Hz, 1H, H-6b), 3.69 (dd, *J* = 8.8, 5.2 Hz, 1H, H-4), 3.53 (ddd, *J* = 9.4, 8.0, 4.9 Hz, 1H, H-2), 3.43 (t, *J* = 6.5 Hz, 2H, CH_2_–5′), 3.40 (t, *J* = 6.6 Hz, 2H, CH_2_–6′), 3.38 (t, *J* = 8.4 Hz, 1H, H-3), 3.08–2.99 (m, 1H, H-5), 2.80 (dd, *J* = 12.4, 4.8 Hz, 1H, H-1a), 2.79–2.73 (m, 1H, H-1′a), 2.70–2.61 (m, 1H, H-1′b), 2.58 (dd, *J* = 12.4, 9.5 Hz, 1H, H-1b), 1.63–1.49 (m, 6H, CH_2_–2′, CH_2_–4′, CH_2_–7′), 1.43–1.24 (m, 14H, CH_2_–3′, CH_2_–8′–13′), 0.90 (t, *J* = 7.0 Hz, 3H, CH_3_-14′). ^13^C NMR (100 MHz, MeOD) δ 75.8 (C-3), 72.8 (C-4), 72.0 (C-6′), 71.8 (C-5′), 71.2 (C-2), 64.3 (C-5), 57.6 (C-6), 55.5 (C-1′), 52.9 (C-1), 33.1, 30.8, 30.7, 30.6, 30.4, 28.4, 27.3, 25.0, 23.7 (9 × CH_2_), 14.4 (C-14′). HRMS: calcd for [C_20_H_42_NO_5_]^+^ 376.30575, found 376.30605.

#### *N*-(2′-Hydroxyethyl)-D-*Gal*-DNM Trifluoroacetic Acid Salt

*N*-Boc-2-*O*-TBDPS-3,4-*O*-isopropylidene-6*-O*-benzyl-D-*Gal*-DNM^9b^ (198 mg, 0.335 mmol) was dissolved in dry DCM (5 mL), and TFA (3 mL) was added dropwise in 2 min at 4 °C. The mixture was stirred for 60 min and subsequently diluted with toluene and concentrated. The residue was dissolved in DCM, washed with aqueous 10% w/v Na_2_CO_3_ solution (2 × 20 mL) and brine (10 mL), dried over MgSO_4_, filtered, and concentrated to give a colorless oil (175 mg). The mixture was dissolved in THF (10 mL), and benzyloxyacetaldehyde (75 mg, 0.50 mmol) and acetic acid (100 μL, 1.65 mmol) were added. After 5 min, NaBH­(OAc)_3_ (144 mg, 0.678 mmol) was added, and the reaction was left stirring overnight until LCMS confirmed full conversion of the secondary amine to the tertiary and partial removal of the acetonide. The reaction was diluted with ethyl acetate (50 mL), washed with aqueous 10% w/v Na_2_CO_3_ solution (2 × 20 mL) and brine (20 mL), dried over MgSO_4_, filtered, and concentrated. The residue was dissolved in a mixture of acetone (10 mL) and 2,2-dimethoxypropane (5 mL), and *p*-toluenesulfonic acid (150 mg) was added, and the reaction was stirred at room temperature. After LCMS confirmed complete reinstallation of the 2,3-isopropylidene (1 h), the mixture was diluted with ethyl acetate (50 mL) and washed with aqueous 10% w/v Na_2_CO_3_ solution (2 × 20 mL) and brine (20 mL), dried over MgSO_4_, filtered, and concentrated. Purification by silica gel column chromatography using pentane:EtOAc 95:5 → 9:1 → 3:1 afforded 325 mg of impure material that was dissolved in THF (10 mL) and, after addition of 1 M TBAF (0.9 mL), left stirring over the weekend. The reaction mixture was diluted with ethyl acetate, washed with water (5 mL) and brine (5 mL), dried over MgSO_4_, filtered, and concentrated. The crude material was purified by silica gel column chromatography using pentane:EtOAc 9:1 → 3:1 → 1:1 → 0:1 to afford *N*-2′-benzyloxyethyl-3,4-*O*-isopropylidene-6*-O*-benzyl-D-*Gal*-DNM (120 mg) in 84% overall yield. ^1^H NMR (400 MHz, CDCl_3_) δ 7.36–7.21 (m, 10H, Ar), 4.50 (s, 2H, CH_2_Ph), 4.47 (s, 2H, CH_2_Ph), 4.31–4.24 (m, 1H, H-4), 3.80 (m, 3H, H-2, H-3, H-6a), 3.72 (dd, *J* = 9.7, 5.7 Hz, 1H, H-6b), 3.65–3.49 (m, 2H, CH_2_–2′), 3.24–2.92 (m, 4H, OH, H-5, H-1a, H-1′a), 2.86 (dt, *J* = 14.2, 5.6 Hz, 1H, H-1′b), 2.36–2.24 (m, 1H, H-1b), 1.50 (s, 3H, CH_3_), 1.33 (s, 3H, CH_3_). ^13^C NMR (101 MHz, CDCl_3_) δ 138.2, 138.2 (*C*_q_ Ar), 128.4, 128.4, 127.7, 127.6 (Ar), 109.0 (*C*_q_ acetonide), 79.9 (C-3), 75.1 (C-4), 73.3 (CH_2_Ph), 73.2 (CH_2_Ph), 70.2 (C-6), 69.8 (C-2), 67.7 (C-2′), 59.7 (C-5), 55.2 (C-1), 52.3 (C-1′), 28.3 (CH_3_), 26.05 (CH_3_). HRMS calcd for [C_25_H_34_NO_5_]^+^ 428.24315, found 428.24231. This product (120 mg, 0.281 mmol) was dissolved in methanol (15 mL) and, after addition of 6 M HCl (2 mL) and Pd/C-10% (50% w/w water, 60 mg), hydrogenated under a balloon of hydrogen for 48 h. The mixture was filtered over a Whatman and concentrated to give a colorless foam in quantitative yield. Purification by RP-HPLC afforded the target compound (70 mg) in 78% yield. ^1^H NMR (400 MHz, D_2_O) δ 4.24 (dd, *J* = 2.9, 1.7 Hz, 1H, H-4), 4.09 (ddd, *J* = 11.0, 9.9, 4.9 Hz, 1H, H-2), 4.01 (dd, *J* = 12.9, 5.1 Hz, 1H, H-6a), 3.97 (dd, *J* = 12.9, 5.1 Hz, 1H, H-6b), 3.95 (t, *J* = 5.2 Hz, 2H, CH_2_–2′), 3.65 (dd, *J* = 12.5, 4.9 Hz, 1H, H-1a), 3.61 (dd, *J* = 9.6, 3.1 Hz, 1H, H-3), 3.58–3.50 (m, 2H, H-5, H-1′a), 3.38 (dt, *J* = 14.0, 5.0 Hz, 1H, H-1′b), 3.11–2.99 (m, 1H, H-1b). ^13^C NMR (101 MHz, D_2_O) δ 72.5 (C-3), 69.4 (C-4), 65.0 (C-5), 64.0 (C-2), 58.5 (C-6), 55.2 (C-2′), 54.1 (C-1′), 53.5 (C-1). HRMS calcd for [C_8_H_18_NO_5_]^+^ 208.1180, found 208.1183.

#### *N*-(5-(Nonyloxy)­pentyl)-D-*Gal*-DNM Trifluoroacetic Acid Salt

Prepared in 46% yield following general procedure A (0.265 mmol scale). ^1^H NMR (400 MHz, D_2_O) δ 4.24 (d, *J* = 2.7 Hz, 1H, H-4), 4.08 (ddd, *J* = 10.6, 10.5, 4.9 Hz, 1H, H-2), 4.02–3.88 (m, 2H, CH_2_–6), 3.61 (dd, *J* = 9.5, 2.6 Hz, 1H, H-3), 3.48 (dd, *J* = 12.1, 4.8 Hz, 1H, H-1a), 3.45–3.33 (m, 5H, H-5, CH_2_–5′, CH_2_–6′), 3.34–3.12 (m, 2H, CH_2_–1′), 2.94 (app t, *J* = 11.6 Hz, 1H, H-1b), 1.84–1.65 (m, 2H, CH_2_–2′), 1.64–1.44 (m, 4H, CH_2_–4′, CH_2_–7′), 1.41–1.32 (m, 2H, CH_2_–3′), 1.31–1.17 (m, 12H, CH_2_–8′– 13′), 0.85 (t, *J* = 6.8 Hz, 3H, CH_3_-14′). ^13^C NMR (101 MHz, D_2_O) δ 72.7 (C-3), 70.8, 70.4 (C-5′, C-6′), 69.6 (C-4), 64.5 (C-2), 64.3 (C-5), 59.1 (C-6), 53.3 (C-1), 52.9 (C-1′), 31.9, 29.7, 29.5, 29.4, 29.3, 28.6, 25.9, 22.9 (C-3′), 22.6, 22.2 (C-2′), 13.8 (C-14′). HRMS calcd for [C_20_H_42_NO_5_]^+^ 376.3058, found 376.3060.

#### *N*-(5-(Neopentyloxy)­pentyl)-D-*Gal*-DNM Trifluoroacetic Acid Salt

Prepared in 52% yield following general procedure A (0.336 mmol scale). ^1^H NMR (400 MHz, D_2_O) δ 4.23 (s, 1H, H-4), 4.05 (td, *J* = 10.6, 5.1 Hz, 1H, H-2), 3.95 (d, *J* = 3.8 Hz, 2H, CH_2_–6), 3.57 (d, *J* = 8.4 Hz, 1H, H-3), 3.49 (m, 3H, H-1a, CH_2_–5′), 3.42 (broad s, 1H, H-5), 3.26 (m, 2H, CH_2_–1′), 3.14 (s, 2H, CH_2_–6′), 2.95 (app t, *J* = 11.8 Hz, 1H, H-1b), 1.81–1.65 (m, 2H, CH_2_–2′), 1.59 (m, 2H, CH_2_–4′), 1.38 (m, 2H, CH_2_–3′), 0.84 (s, 9H, C­(CH_3_)_3_). ^13^C NMR (101 MHz, D_2_O) δ 81.1 (C-6′), 72.6 (C-3), 71.0 (C-5′), 69.8 (C-4), 64.5 (C-2), 63.9 (C-5), 59.1 (C-6), 53.3 (C-1), 53.2 (C-1′), 31.2 (*C*_q_ *t*Bu), 27.8 (C-4′), 25.9 ((CH_3_)_3_), 22.4 (C-3′), 21.5 (C-2′). HRMS calcd for [C_16_H_34_NO_5_]^+^ 320.24315, found 320.24305.

#### *N*-(2-Benzyloxyethyl)-D-*Tal*-DNM Trifluoroacetic Acid Salt

Prepared in 47% yield following general procedure A (0.260 mmol scale). ^1^H NMR (500 MHz, MeOD) δ 7.41–7.32 (m, 4H, Ar), 7.30 (t, *J* = 6.8 Hz, 1H, Ar), 4.58 (s, 2H, CH_2_–3′), 4.26–3.94 (m, 4H, H-4, H-2, CH_2_–2′), 3.92–3.82 (m, 2H, CH_2_–6), 3.80–3.67 (m, 2H, H-3, CH_2_–1′a), 3.65–3.45 (m, 3H, H-5, H-1a, CH_2_–1′b), 3.40 (d, *J* = 11.3 Hz, 1H, H-1b). ^13^C NMR (126 MHz, MeOD) δ 138.7 (*C*_q_ Ar), 129.6, 129.1, 129.1 (Ar), 74.3 (C-3′), 71.6 (C-4), 68.8 (C-3), 68.1 (C-2), 68.0 (C-5), 64.6 (C-6), 61.1 (C-1), 57.5 (C-2′), 54.4 (C-1′). HRMS calcd for [C_15_H_24_NO_5_]^+^ 298.16490, found 298.16461.

#### *N*-(2′-Hydroxyethyl)-D-*Tal*-DNM Trifluoroacetic Acid Salt

Prepared in 74% yield following general procedure B, step two (0.105 mmol scale). ^1^H NMR (400 MHz, MeOD) δ 3.95–3.87 (m, 2H, H-4, H-6a), 3.84 (dd, *J* = 11.7, 4.6 Hz, 1H, H-6b), 3.80 (dt, *J* = 5.1, 2.6 Hz, 1H, H-2), 3.73–3.59 (m, 3H, H-3, CH_2_–2′), 3.05 (dd, *J* = 12.5, 6.1 Hz, 1H, H-1a), 2.98 (dt, *J* = 12.2, 6.0 Hz, 1H, CH_2_–1′a), 2.74 (dt, *J* = 13.6, 5.3 Hz, 1H, CH2–1′b), 2.63 (m, 1H, H-5), 2.55 (dd, *J* = 12.5, 2.9 Hz, 1H, H-1b). ^13^C NMR (101 MHz, MeOD) δ 72.0 (C-4), 71.9 (C-3), 69.7 (C-2), 65.7 (C-5), 61.6 (C-6), 60.1 (C-2′), 56.0 (C-1′), 54.8 (C-1). HRMS calcd for [C_8_H_18_NO_5_]^+^ 208.11795, found 208.11805.

#### *N*-(5-(Nonyloxy)­pentyl)-D-*Tal*-DNM Trifluoroacetic Acid Salt

Prepared in 29% yield following general procedure A (0.355 mmol scale). ^1^H NMR (400 MHz, D_2_O) δ 4.21 (s, 1H, H-4), 4.17 (s, 1H, H-2), 4.07–3.89 (m, 2H, CH_2_–6), 3.83 (s, 1H, H-3), 3.58–3.12 (m, 9H, CH_2_–1, CH_2_–1′, H-5, CH_2_–5′, CH_2_–6′), 1.86–1.64 (m, 2H, CH_2_–2′), 1.64–1.46 (m, 4H, CH_2_–4′, CH_2_–7′), 1.42–1.12 (m, 14H, CH_2_–3′, CH_2_–8′–13′), 0.83 (t, *J* = 6.6 Hz, 3H, CH_3_-14′). ^13^C NMR (101 MHz, D_2_O) δ 70.7 (C-5′), 70.4 (C-6′), 69.5 (C-4), 67.2 (C-3), 66.6 (C-2), 64.4 (C-5), 58.9 (C-1), 53.1 (C-1′), 31.9, 29.7, 29.5, 29.3, 29.2, 28.6, 25.9, 22.8, 22.5, 22.2 (10 × CH_2_), 13.7 (C-14′). HRMS calcd for [C_20_H_42_NO_5_]^+^ 376.30575, found 376.30577.

#### *N*-(5-(Neopentyloxy)­pentyl)-D-*Tal*-DNM Trifluoroacetic Acid Salt

Prepared in 21% yield following general procedure A (0.333 mmol scale). ^1^H NMR (400 MHz, D_2_O) δ 4.26 (s, 1H, H-4), 4.22 (s, 1H, H-2), 4.02 (dd, *J* = 13.0, 5.3 Hz, 1H, H-6a), 3.96 (dd, *J* = 13.0, 4.5 Hz, 1H, H-6b), 3.83 (t, *J* = 3.2 Hz, 1H, H-3), 3.58–3.47 (m, 3H, H-1a, CH_2_–5′), 3.45 (s, 1H, H-5), 3.38 (d, *J* = 13.1 Hz, 1H, H-1b), 3.34–3.21 (m, 2H, CH_2_–1′), 3.16 (s, 2H, CH_2_–6′), 1.86–1.66 (m, 2H, CH_2_–2′), 1.66–1.55 (m, 2H, CH_2_–4′), 1.47–1.32 (m, 2H, CH_2_–3′), 0.85 (s, 9H, C­(CH_3_)_3_). ^13^C NMR (101 MHz, D_2_O) δ 81.2 (C-6′), 71.1 (C-5′), 70.0 (C-4), 66.90 (C-3), 66.85 (C-2), 64.5 (C-5), 59.3 (C-6), 55.5 (C-1), 53.2 (C-1′), 31.3 (*C*_q_ *t*Bu), 27.9 (C-4′), 25.9 ((CH_3_)_3_), 22.5 (C-3′), 21.7 (C-2′). HRMS calcd for [C_16_H_34_NO_5_]^+^ 320.2432, found 320.2437.

#### *N*-(5-((Adamantan-1-yl)­methoxy)­pentyl)-D-*Tal*-DNM Trifluoroacetic Acid Salt

Prepared in 34% yield following general procedure A (0.333 mmol scale). ^1^H NMR (400 MHz, D_2_O) δ 4.25 (s, 1H, H-4), 4.20 (s, 1H, H-2), 4.00 (dd, *J* = 12.8, 4.9 Hz, 1H, H-6a), 3.94 (dd, *J* = 12.9, 3.6 Hz, 1H, H-6b), 3.82 (s, 1H, H-3), 3.50 (d, *J* = 11.4 Hz, 1H, H-1a), 3.45–3.15 (m, 6H, H-1b, H-5, CH_2_–1′, CH_2_–5′), 2.97 (s, 2H, CH_2_–6′), 1.91 (br s, 3H, 3 × CH Ada), 1.82–1.42 (m, 16H, CH_2_–2′, CH_2_–4′ 6 × CH_2_ Ada), 1.40–1.28 (m, 2H, CH_2_–3′). ^13^C NMR (101 MHz, D_2_O) δ 81.7 (C-6′), 71.5 (C-5′), 69.9 (C-4), 66.9 (C-3), 66.8 (C-2), 64.8 (C-5), 59.4 (C-6), 55.5 (C-1), 53.2 (C-1′), 39.4 (CH_2_ Ada), 37.0 (CH_2_ Ada), 33.8 (*C*_q_ Ada), 28.3 (C-4′), 28.2 (CH Ada), 22.7 (C-3′), 21.8 (C-2′). HRMS calcd for [C_22_H_40_NO_5_]^+^ 398.2901, found 398.2900.

#### *N*-(5-([1,1′-Biphenyl]-4-ylmethoxy)­pentyl)-D-*Tal*-DNM Trifluoroacetic Acid Salt

Prepared in 30% yield following general procedure A (0.360 mmol scale). ^1^H NMR (400 MHz, D_2_O) δ 7.13 (s, 2H, Ar), 7.11 (s, 2H, Ar), 6.98 (d, *J* = 7.3 Hz, 2H, Ar), 6.90 (t, *J* = 7.2 Hz, 2H, Ar), 6.83 (t, *J* = 7.1 Hz, 1H, Ar), 4.17 (s, 1H, H-4), 4.14 (s, 2H, CH_2_–6′), 4.06 (s, 1H, H-2), 3.97–3.82 (m, 2H, CH_2_–6), 3.66 (s, 1H, H-3), 3.30 (d, *J* = 12.0 Hz, 1H, H-1a), 3.24–2.96 (m, 6H, H-1b, H-5, CH_2_–1′, CH_2_–5′), 1.63–1.24 (m, 4H, CH_2_–2′, CH_2_–4′), 1.19–0.98 (m, 2H, CH_2_–3′). ^13^C NMR (101 MHz, D_2_O) δ 139.9, 139.5, 137.2 (*C*_q_ Ar), 128.7, 128.3, 127.2, 126.5 (Ar), 71.8 (C-6′), 70.0 (C-4), 69.7 (C-5′), 66.9 (C-3), 66.8 (C-2), 64.8 (C-5), 59.4 (C-6), 55.5 (C-1), 53.0 (C-1′), 28.4 (C-4′), 22.6 (C-3′), 21.7 (C-2′). HRMS calcd for [C_24_H_34_NO_5_]^+^ 416.24315, found 416.24126.

#### *N*-Butyl-L-*Glc*-DNM Trifluoroacetic Acid Salt

Prepared in 40% yield following general procedure A (0.250 mmol scale). ^1^H NMR (400 MHz, MeOD) δ 4.09 (d, *J* = 12.5 Hz, 1H, H-6a), 3.92 (dd, *J* = 12.5, 3.0 Hz, 1H, H-6b), 3.71 (ddd, *J* = 11.0, 9.2, 4.9 Hz, 1H, H-2), 3.60 (dd, *J* = 10.3, 9.2 Hz, 1H, H-4), 3.44 (dd, *J* = 12.1, 4.9 Hz, 1H, H-1a), 3.37 (t, *J* = 9.2 Hz, 1H, H-3), 3.33 (m, 1H, H-1′a), 3.19 (dt, *J* = 12.8, 6.5 Hz, 1H, H-1′b), 3.04 (d, *J* = 9.2 Hz, 1H, H-5), 2.96 (t, *J* = 11.7 Hz, 1H, H-1b), 1.82–1.48 (m, 2H, CH_2_–2′), 1.50–1.35 (m, 2H, CH_2_–3′), 1.01 (t, *J* = 7.4 Hz, 3H, CH_3_-4′). ^13^C NMR (101 MHz, MeOD) δ 78.2 (C-3), 69.0 (C-4), 67.9 (C-2), 67.4 (C-5), 55.2 (C-6), 54.8 (C-1), 54.1 (C-1′), 26.2 (C-2′), 21.0 (C-3′), 13.9 (C-4′). HRMS calcd for [C_10_H_22_NO_4_]^+^ 220.15433, found 220.15428.

#### *N*-(2-Hydroxyethyl)-L-*Glc*-DNM Trifluoroacetic Acid Salt

Prepared in 47% yield following general procedure B (0.220 mmol scale). ^1^H NMR (400 MHz, MeOD, mixture of conformers) δ 4.17–4.11 (m, 1H, H-3), 4.11–3.98 (m, 3H, CH_2_–6, H-2′a), 4.07 (ddd, *J* = 10.4, 6.7, 3.6 Hz, 1H, H-2), 3.99–3.93 (m, 1H, H-4), 3.88 (dd, *J* = 11.7, 4.8 Hz, 1H, H-2′b), 3.75–3.66 (m, 1H, H-1a), 3.58–3.48 (m, 2H, H-5, H-1b), 3.45 (dd, *J* = 13.1, 2.1 Hz, 1H, H-1′a), 3.32 (dd, *J* = 13.4, 3.3 Hz, 1H, H-1′b). ^13^C NMR (100 MHz, MeOD) δ 73.1 (C-3), 70.1 (C-4), 69.3 (C-2), 61.6 (C-2′), 59.2 (C-5), 57.5 (C-6), 57.4 (C-1), 47.8 (C-1′). HRMS calcd for [C_8_H_18_NO_5_]^+^ 208.11795, found 208.11797.

#### *N*-Nonyl-L-*Glc*-DNM Trifluoroacetic Acid Salt

Prepared in 37% yield following general procedure A (0.220 mmol scale). ^1^H NMR (400 MHz, MeOD) δ 4.07 (d, *J* = 12.5 Hz, 1H, H-6a), 3.90 (dd, *J* = 12.4, 3.0 Hz, 1H, H-6b), 3.67 (dt, *J* = 10.3, 4.7 Hz, 1H, H-2), 3.57 (t, *J* = 9.7 Hz, 1H, H-4), 3.40 (dd, *J* = 12.1, 4.7 Hz, 1H, H-1a), 3.35 (t, *J* = 9.6 Hz, 1H, H-3), 3.30–3.21 (m, 1H, H-1′a), 3.18–3.04 (m, 1H, H-1′b), 3.02–2.79 (m, 2H, H-5, H-1b), 1.83–1.60 (m, 2H, CH_2_–2′), 1.45–1.22 (m, 12H, CH_2_–3′– 8′), 0.91 (t, *J* = 7.0 Hz, 3H, CH_3_-9′). ^13^C NMR (100 MHz, MeOD) 78.5 (C-3), 71.2 (C-4), 68.3 (C-2), 67.4 (C-5), 55.5 (C-6), 55.1 (C-1), 54.3 (C-1′), 33.0, 30.5, 30.3, 30.3, 27.8, 24.4, 23.7 (7 × CH_2_), 14.4 (C-9′). HRMS calcd for [C_15_H_32_NO_4_]^+^ 290.23258, found 290.23260.

#### *N*-(5-(Nonyloxy)­pentyl)-L-*Glc*-DNM Trifluoroacetic Acid Salt

Prepared in 26% yield following general procedure A (0.244 mmol scale). ^1^H NMR (400 MHz, MeOD) δ 4.11 (d, *J* = 12.5 Hz, 1H, H-6a), 3.91 (dd, *J* = 12.5, 3.2 Hz, 1H, H-6b), 3.71 (ddd, *J* = 11.2, 9.3, 4.9 Hz, 1H, H-2), 3.60 (dd, *J* = 10.5 Hz, 1H, H-4), 3.46 (t, *J* = 12.7 Hz, 2H, H-5′), 3.43 (t, *J* = 12.6 Hz, 2H, H2–6′), 3.50–3.33 (m, 3H, H-1a, H-1′a, H-3), 3.22 (td, *J* = 12.6, 5.3 Hz, 1H, H-1′b), 3.08 (dd, *J* = 10.3, 2.8 Hz, 1H, H-5), 3.01 (dd, *J* = 12.2, 11.2 Hz, 1H, H-1b), 1.89–1.71 (m, 2H, CH_2_–2′), 1.70–1.59 (m, 2H, CH_2_–7′), 1.60–1.52 (m, 2H, CH_2_–4′), 1.49–1.40 (m, 2H, CH_2_–3′), 1.40–1.23 (m, 12H, CH_2_–8′–13′), 0.90 (t, *J* = 7.0 Hz, 3H, CH_3_-14′). ^13^C NMR (100 MHz, MeOD) δ 78.1 (C-3), 72.1 (C-6′), 71.4 (C-5′), 68.8 (C-4), 67.8 (C-2), 67.4 (C-5), 55.7 (C-6), 54.9 (C-1), 54.3 (C-1′), 33.0, 30.8, 30.7, 30.6, 30.4, 30.1, 27.3, 24.5, 24.0, 23.7 (10 × CH_2_), 14.3 (C-14′). HRMS calcd for [C_20_H_42_NO_5_]^+^ 376.30575, found 376.30587.

#### *N*-(5-(Neopentyloxy)­pentyl)-L-*Glc*-DNM Trifluoroacetic Acid Salt

Prepared in 33% yield following general procedure A (0.190 mmol scale). ^1^H NMR (400 MHz, MeOD) δ 3.94 (dd, *J* = 12.2, 2.2 Hz, 1H, H-6a), 3.88 (dd, *J* = 12.2, 3.0 Hz, 1H, H-6b), 3.56 (td, *J* = 10.5, 9.9, 4.8 Hz, 1H, H-2), 3.45 (t, *J* = 9.7 Hz, 1H, H-4), 3.43 (t, *J* = 6.3 Hz, 2H, CH_2_–5′), 3.22 (t, *J* = 9.1 Hz, 1H, H-3), 3.20–3.11 (m, 1H, H-1a), 3.07 (s, 2H, CH_2_–6′), 3.06–2.96 (m, 1H, CH_2_–1′a), 2.89–2.75 (m, 1H, CH_2_–1′b), 2.58–2.36 (m, 2H, H-1b, H-5), 1.85–1.66 (m, 4H, CH_2_–2′, CH_2_–4′), 1.59–1.43 (m, 2H, CH_2_–3′), 0.93 (s, 9H, (CH_3_)_3_). ^13^C NMR (100 MHz, MeOD) δ 82.5 (C-6′), 79.6 (C-3), 72.3 (C-5′), 70.8 (C-4), 69.6 (C-2), 67.4 (C-5), 55.5 (C-6), 57.8 (C-1), 53.9 (C-1′), 32.9 (*C*_q_ *t*Bu), 30.5 (C-4′), 27.2 ((CH_3_)_3_), 25.0 (C-3′), 24.7 (C-2′). HRMS calcd for [C_16_H_34_NO_5_]^+^ 320.24315, found 320.24339.

#### *N*-(5-((Adamantan-1-yl)­methoxy)­pentyl)-L-*Glc*-DNM Trifluoroacetic Acid Salt

Prepared in 14% yield following general procedure A (0.214 mmol scale). ^1^H NMR (400 MHz, MeOD) δ 3.87 (d, *J* = 2.7 Hz, 2H, H_2_-6), 3.49 (ddd, *J* = 10.6, 9.1, 4.9 Hz, 1H, H-2), 3.39 (t, *J* = 6.4 Hz, 2H, CH_2_–5′), 3.38 (t, *J* = 9.2 Hz, 1H, H-4), 3.15 (t, *J* = 9.1 Hz, 1H, H-3), 3.03 (dd, *J* = 11.3, 4.9 Hz, 1H, H-1a), 2.97 (s, 2H, CH_2_–6′), 2.85 (ddd, *J* = 13.4, 9.6, 6.7 Hz, 1H, H-1′a), 2.65 (ddd, *J* = 13.3, 9.4, 6.1 Hz, 1H, H-1′b), 2.26 (t, *J* = 11.0 Hz, 1H, H-1b), 2.21 (dt, *J* = 9.6, 2.8 Hz, 1H, H-5), 1.98–1.91 (m, 3H, 3 × CH Ada), 1.80–1.65 (m, 6H, 3 × CH_2_ Ada), 1.63–1.49 (m, 10H, 3 × CH_2_ Ada, CH_2_–2′, CH_2_–4′), 1.42–1.31 (m, 2H, CH_2_–3′). ^13^C NMR (101 MHz, MeOD) δ 83.1 (C-6′), 80.3 (C-3), 72.5 (C-5′), 71.7 (C-4), 70.5 (C-2), 67.3 (C-5), 59.0 (C-6), 57.4 (C-1), 53.8 (C-1′), 40.9 (3 × CH_2_ Ada), 38.3 (3 × CH_2_ Ada), 35.2 (*C*_q_ Ada), 30.5 (C-4′), 29.8 (3 × CH Ada), 25.3 (C-3′), 24.9 (C-2′). HRMS calcd for [C_22_H_40_NO_5_]^+^ 398.29010, found 398.28980.

#### *N*-(5-([1,1′-Biphenyl]-4-ylmethoxy)­pentyl)-L-*Glc*-DNM Trifluoroacetic Acid Salt

Prepared in 22% yield following general procedure A (0.318 mmol scale). ^1^H NMR (400 MHz, MeOD) δ 7.53–7.45 (m, 1H, Ar), 7.44–7.30 (m, 7H, Ar), 7.29–7.21 (m, 1H, Ar), 4.35 (s, 2H, CH_2_–6′), 3.90–3.78 (m, 2H, CH_2_–6), 3.48 (td, *J* = 10.2, 4.9 Hz, 1H, H-2), 3.40–3.32 (m, 3H, CH_2_–5′, H-4), 3.14 (t, *J* = 9.1 Hz, 1H, H-3), 2.98 (dd, *J* = 11.1, 4.9 Hz, 1H, H-1a), 2.83–2.71 (m, 1H, H-1′a), 2.62–2.50 (m, 1H, H-1′b), 2.17 (t, *J* = 10.9 Hz, 1H, H-1b), 2.10 (dt, *J* = 9.6, 2.6 Hz, 1H, H-5), 1.59–1.38 (m, 4H, CH_2_–4′, CH_2_–2′), 1.37–1.21 (m, 2H, CH_2_–3′). ^13^C NMR (101 MHz, MeOD) δ 143.5, 142.3, 136.6 (*C*_q_ Ar), 130.9, 130.7, 130.2, 129.2, 128.7, 128.4, 128.2 (Ar), 80.5 (C-3), 72.0 (C-4), 71.8 (C-6′), 71.4 (C-5′), 70.7 (C-2), 67.3 (C-5), 59.4 (C-6), 57.7 (C-1), 53.7 (C-1′), 30.5 (C-4′), 25.2 (C-3′), 25.0 (C-2′). HRMS calcd for [C_24_H_34_NO_5_]^+^ 416.24315, found 416.24299.

#### *N*-Butyl-L-*Man*-DNM Trifluoroacetic Acid Salt

Prepared in 42% yield following general procedure A (0.101 mmol scale). ^1^H NMR (400 MHz, MeOD) δ 4.12 (s, 1H, H-2), 4.08 (dd, *J* = 12.7, 2.9 Hz, 1H, H-6a), 4.01–3.88 (m, 2H, H-4, H-6b), 3.57 (d, *J* = 7.3 Hz, 1H, H-3), 3.46 (dd, *J* = 12.9, 3.6 Hz, 1H, H-1a), 3.40 (d, *J* = 13.0 Hz, 1H, H-1b), 3.38–3.24 (m, 2H, CH_2_–1′), 3.05 (d, *J* = 9.8 Hz, 1H, H-5), 1.82–1.66 (m, 2H, CH_2_–2′), 1.49–1.35 (m, 2H, CH_2_–3′), 1.01 (t, *J* = 7.4 Hz, 3H, CH_3_-4′). ^13^C NMR (100 MHz, MeOD) δ 74.2 (C-3), 67.8 (C-5), 67.2 (C-2), 67.0 (C-4), 56.0 (C-6), 56.0 (C-1), 54.3 (C-1′), 25.7 (C-2′), 20.9 (C-3′), 13.9 (C-4′). HRMS calcd for [C_10_H_22_NO_4_]^+^ 220.15433, found 220.15434.

#### *N*-(2-Hydroxyethyl)-L-*Man*-DNM Trifluoroacetic Acid Salt

Prepared in 40% yield following general procedure B, step two. (0.05 mmol scale). ^1^H NMR (400 MHz, MeOD) δ 4.06–3.92 (m, 1H, H-2), 3.9–3.85 (m, 1H, H-6a), 3.85–3.66 (m, 4H, H-4, H-6b, CH_2_–2′), 3.52–3.33 (m, 3H, H-3, H-1a, CH_2_–1′a), 3.32–3.23 (m, 1H, CH_2_–1′b), 3.22–3.11 (m, 1H, H-1b), 3.08–2.95 (m, 1H, H-5). ^13^C NMR (101 MHz, MeOD) δ 73.7 (C-3), 68.2 (C-2), 66.7 (C-4), 66.6 (C-5), 56.3 (C-6), 56.1 (C-2′), 55.6 (C-1), 55.3 (C-1′). HRMS calcd for [C_8_H_18_NO_5_]^+^ 208.11795, found 208.11799.

#### *N*-Nonyl-L-*Man*-DNM Trifluoroacetic Acid Salt

Prepared in 17% yield following general procedure A (0.176 mmol scale). ^1^H NMR (400 MHz, MeOD) δ 3.91 (dd, *J* = 11.8, 2.6 Hz, 1H, H-6a), 3.86–3.80 (m, 2H, H-2, H-6b), 3.69 (t, *J* = 9.1 Hz, 1H, H-4), 3.34 (dd, *J* = 9.0, 3.4 Hz, 1H, H-3), 3.03 (dd, *J* = 12.3, 3.4 Hz, 1H, H-1a), 2.86–2.73 (m, 1H, H-1′a), 2.72–2.61 (m, 1H, H-1′b), 2.57 (d, *J* = 12.3 Hz, 1H, H-1b), 2.27–2.13 (m, 1H, H-5), 1.64–1.44 (m, 2H, CH_2_–2′), 1.38–1.19 (m, 12H, CH_2_–3′–8′), 0.90 (t, *J* = 7.1 Hz, 3H, CH_3_-9′). ^13^C NMR (100 MHz, MeOD) δ 76.3 (C-3), 69.4 (C-4), 69.4 (C-2), 67.1 (C-5), 58.8 (C-6), 56.4 (C-1), 54.0 (C-1′), 33.1, 30.7, 30.7, 30.4, 28.5, 25.1, 23.7 (7 × CH_2_), 14.4 (C-9′). HRMS calcd for [C_15_H_32_NO_4_]^+^ 290.23258, found 290.23291.

#### *N*-(5-(Nonyloxy)­pentyl)-L-*Man*-DNM Trifluoroacetic Acid Salt

Prepared in 29% yield following general procedure A (0.207 mmol scale). ^1^H NMR (400 MHz, MeOD) δ 4.13 (br. s, 1H, H-2), 4.08 (dd, *J* = 12.7, 2.4 Hz, 1H, H-6a) 3.99–3.90 (m, 2H, H-4, H-6b), 3.58 (dd, *J* = 9.2, 2.6 Hz, 1H, H-3), 3.50–3.39 (m, 6H, CH_2_–1, CH_2_–5′, CH_2_–6′), 3.40–3.26 (m, 2H, CH_2_–1′), 3.07 (d, *J* = 7.4 Hz, 1H, H-5), 1.87–1.70 (m, 2H, CH_2_–2′), 1.69–1.61 (m, 2H, CH_2_–4′), 1.60–1.51 (m, 2H, CH_2_–7′), 1.50–1.41 (m, 2H, CH_2_–3′), 1.39–1.23 (m, 12H, CH_2_–8′–13′), 0.90 (t, *J* = 7.1 Hz, 3H, CH_3_-14′). ^13^C NMR (100 MHz, MeOD) δ 74.1 (C-3), 72.1 (C-6′), 71.4 (C-5′), 67.8 (C-5), 67.2 (C-2), 67.0 (C-4), 56.0­(C-6), 56.0 (C-1), 54.5 (C-1′), 33.0, 30.8, 30.7, 30.6, 30.4, 30.2, 27.3, 24.5, 23.7, 23.6 (10 × CH_2_), 14.4 (C-14′). HRMS calcd for [C_17_H_36_NO_5_]^+^ 376.30575, found 376.30575.

#### *N*-(5-(Neopentyloxy)­pentyl)-L-*Man*-DNM Trifluoroacetic Acid Salt

Prepared in 24% yield following general procedure A (0.208 mmol scale). ^1^H NMR (400 MHz, MeOD) δ 3.91 (dd, *J* = 11.8, 2.8 Hz, 1H, H-6a), 3.87 (dd, *J* = 11.8, 2.8 Hz, 1H, H-6b), 3.86–3.92 (m, 1H, H-2), 3.69 (t, *J* = 9.1 Hz, 1H, H-4), 3.42 (t, *J* = 6.4 Hz, 2H, H-5′), 3.32 (dd, *J* = 8.5, 3.4 Hz, 1H, H-3), 3.07 (s, 2H, CH_2_–6′), 3.02 (dd, *J* = 12.4, 3.9 Hz, 1H, H-1a), 2.89–2.72 (m, 1H, H-1′a), 2.71–2.60 (m, 1H, H-1′b), 2.56 (d, *J* = 12.3 Hz, 1H, H-1b), 2.20 (d, *J* = 8.9 Hz, 1H, H-5), 1.64–1.57 (m, 2H, CH_2_–4′), 1.56–1.47 (m, 2H, CH_2_–2′), 1.42–1.28 (m, 2H, CH_2_–3′), 0.90 (s, 9H, (CH_3_)_3_). ^13^C NMR (100 MHz, MeOD) δ 82.5 (C-6′), 76.4 (C-3), 72.4 (C-5′), 69.5 (C-2), 69.3 (C-4), 67.0 (C-5), 58.9 (C-6), 56.4 (C-1), 53.9 (C-1′), 32.9 (C-4′), 30.6 (*C*_q_ *t*Bu), 27.2 ((CH_3_)_3_), 25.2­(C-3′), 24.9 (C-2′). HRMS calcd for [C_16_H_34_NO_5_]^+^ 320.24315, found 320.24339.

#### *N*-(5-((Adamantan-1-yl)­methoxy)­pentyl)-L-*Man*-DNM Trifluoroacetic Acid Salt

Prepared in 18% yield with a purity of ≥94% following general procedure A (0.222 mmol scale). ^1^H NMR (600 MHz, MeOD) δ 4.16–4.10 (m, 1H, H-2), 4.07 (dd, *J* = 12.7, 3.0 Hz, 1H, H-6a), 3.96 (dd, *J* = 12.7, 2.9 Hz, 1H, H-6b), 3.94 (t, *J* = 9.3 Hz, 1H, H-4), 3.62–3.54 (m, 1H, H-3), 3.51 (t, *J* = 7.4 Hz, 2H, CH_2_–5′), 3.48 (t, *J* = 6.0 Hz, 2H, CH_2_–6′), 3.44 (dd, *J* = 12.8, 3.8 Hz, 1H, H-1a), 3.40–3.28 (m, 3H, CH_2_–1′, H-1b), 3.12 (br s, 1H, H-5), 1.95–1.91 (m, 3H, 3 × CH Ada), 1.89–1.78 (m, 2H, CH_2_–2′), 1.77–1.72 (m, 3H, 3 × CH_2_ Ada), 1.70– 1.60 (m, 5H, 3 × CH_2_ Ada, CH_2_–4′), 1.65 (d, *J* = 2.8 Hz, 6H, 3 × CH_2_ Ada), 1.46 (t, *J* = 7.4 Hz, 2H, CH_2_–3′). ^13^C NMR (150 MHz, MeOD) δ 74.2 (C-3), 70.9 (C-6′), 68.8 (C-5′), 67.8 (C-5), 67.2 (C-2), 67.2­(C-4), 56.7 (C-6), 56.6 (C-1), 54.5 (C-1′), 44.8 (C-3′), 43.9 (CH_2_ Ada), 38.2 (CH_2_ Ada), 32.8 (*C*_q_ Ada), 30.2 (CH Ada), 27.9 (C-2′). HRMS calcd for [C_22_H_40_NO_5_]^+^ 398.29010, found 398.28949.

#### *N*-(5-([1,1′-Biphenyl]-4-ylmethoxy)­pentyl)-L-*Man*-DNM Trifluoroacetic Acid Salt

Prepared in 18% yield following general procedure A (0.222 mmol scale). ^1^H NMR (400 MHz, MeOD) δ 7.53–7.47 (m, 1H, Ar), 7.45–7.39 (m, 2H, Ar), 7.36 (m, 5H, Ar), 7.30–7.22 (m, 1H, Ar), 4.36 (s, 2H, CH_2_–6′), 3.89 (dd, *J* = 11.7, 2.7 Hz, 1H, H-6a), 3.86 (dd, *J* = 11.7, 2.9 Hz, 1H, H-6b), 3.84 (dd, *J* = 3.7, 2.0 Hz, 1H, H-2), 3.66 (t, *J* = 9.1 Hz, 1H, H-4), 3.39 (t, *J* = 6.4 Hz, 2H, CH_2_–5′), 3.29 (dd, *J* = 7.3, 3.4 Hz, 1H, H-3), 2.96 (dd, *J* = 12.3, 3.9 Hz, 1H, H-1a), 2.75 (dt, *J* = 13.5, 8.3 Hz, 1H, CH_2_–1′a), 2.59 (dt, *J* = 13.6, 8.2 Hz, 1H, CH_2_–1′b), 2.48 (dd, *J* = 12.3, 1.8 Hz, 1H, H-1b), 2.13 (dt, *J* = 9.1, 2.7 Hz, 1H, H-5), 1.60–1.51 (m, 2H, CH_2_–4’), 1.51–1.41 (m, 2H, CH_2_–2′), 1.37–1.24 (m, 2H, CH_2_–3′). ^13^C NMR (100 MHz, MeOD) δ 143.6, 142.3, 136.7 (*C*_q_ Ar), 130.9, 130.8, 130.3 (2×), 129.3 (2×), 128.9, 128.4, 128.2 (Ar), 76.5 (C-3), 71.8 (C-6′), 71.5 (C-5′), 69.6 (C-2), 69.6 (C-4), 67.0 (C-5), 59.1 (C-6), 56.5 (C-1), 53.8 (C-1′), 30.5 (C-4′), 25.2 (C-3′), 25.0 (C-2′). HRMS calcd for [C_24_H_34_NO_5_]^+^ 416.24315, found 416.24303.

#### *N*-Butyl-L-*Alt*-DNM Trifluoroacetic Acid Salt

Prepared in 43% yield following general procedure A (0.383 mmol scale). ^1^H NMR (400 MHz, D_2_O) δ 4.12 (dd, *J* = 10.6, 2.8 Hz, 1H, H-4), 4.12–4.07 (m, 1H, H-2), 4.07–3.89 (m, 3H, H-3, CH_2_–6), 3.48 (d, *J* = 12.6 Hz, 1H, H-1a), 3.35–3.19 (m, 4H, H-1b, H-5, CH_2_–1′), 1.68 (dq, *J* = 14.9, 7.1 Hz, 2H, CH_2_–2′), 1.34 (dt, *J* = 14.2, 7.2 Hz, 2H, CH_2_–3′), 0.90 (t, *J* = 7.4 Hz, 3H, CH_3_-4′). ^13^C NMR (101 MHz, D_2_O) δ 68.1 (C-3), 66.0 (C-2), 62.9 (C-4), 61.1 (C-5), 54.0 (C-6), 53.0 (C-1′), 51.2 (C-1), 23.8 (C-2′), 19.2 (C-3′), 12.7 (C-4′). HRMS calcd for [C_10_H_22_NO_4_]^+^ 220.1546, found 220.1543.

#### *N*-(2-Benzyloxyethyl)-L-*Alt*-DNM Trifluoroacetic Acid Salt

Prepared in 29% yield following general procedure B, step one (0.383 mmol scale). ^1^H NMR (400 MHz, D_2_O) δ 7.71–7.16 (m, 5H, Ar), 4.59 (s, 2H, CH_2_Ph), 4.16 (d, *J* = 8.9 Hz, 1H, H-4), 4.13–4.08 (m, 1H, H-2), 4.07–3.96 (m, 3H, H-3, CH_2_–6), 4.96–3.86 (m, 2H, CH_2_–2′), 3.70–3.40 (m, 4H, CH_2_–1′, H-1a, H-5), 3.34 (d, *J* = 13.0 Hz, 1H, H-1b). ^13^C NMR (101 MHz, D_2_O) δ 136.8 (*C*_q_ Ar), 128.8, 128.4 (Ar), 73.0 (C-3′), 67.9 (C-3), 65.7 (C-2), 63.0 (C-4), 62.8 (C-2′), 62.2 (C-5), 54.1 (C-6), 52.3 (C-1′), 51.9 (C-1). HRMS calcd for [C_15_H_24_NO_5_]^+^ 298.1649, found 298.1653.

#### *N*-(2-Hydroxyethyl)-L-*Alt*-DNM Trifluoroacetic Acid Salt

Prepared in 42% yield following general procedure B, step two (0.383 mmol scale). ^1^H NMR (400 MHz, D_2_O) δ 4.12 (dd, *J* = 8.2, 2.5 Hz, 1H, H-4), 4.09–4.04 (m, 1H, H-2), 4.03–3.95 (m, 2H, CH_2_–6), 3.94–3.85 (m, 3H, H-3, CH_2_–2′), 3.45–3.33 (m, 3H, H-1a, H-5, H-1′a), 3.33–3.25 (m, 1H, H-1′b), 3.21 (d, *J* = 12.1 Hz, 1H, H-1b). ^13^C NMR (101 MHz, D_2_O) δ 69.0 (C-3), 66.2 (C-2), 64.9 (C-4), 63.0 (C-5), 55.8 (C-2′), 54.9 (C-6), 54.4 (C-1′), 51.6 (C-1). HRMS calcd for [C_8_H_18_NO_5_]^+^ 208.11795, found 208.11795.

#### *N*-Nonyl-L-*Alt*-DNM Trifluoroacetic Acid Salt

Prepared in 51% yield following general procedure A (0.383 mmol scale). ^1^H NMR (400 MHz, D_2_O) δ 4.19 (dd, *J* = 9.5, 2.6 Hz, 1H, H-4), 4.14 (s, 1H, H-2), 4.07 (d, *J* = 11.4 Hz, 1H, H-6a), 4.02 (s, 1H, H-3), 3.96 (d, *J* = 11.4 Hz, 1H, H-6b), 3.48 (d, *J* = 11.5 Hz, 1H, H-1a), 3.37 (d, *J* = 8.9 Hz, 1H, H-5), 3.33–3.20 (m, 3H, H-1b, CH_2_–1′), 1.72 (m, 2H, CH_2_–2′), 3.46–1.17 (m, 12H, CH_2_–3′–8′), 0.88 (t, *J* = 6.4 Hz, 3H, CH_3_-9′). ^13^C NMR (101 MHz, D_2_O) δ 68.4 (C-3), 66.1 (C-2), 63.7 (C-4), 61.7 (C-5), 54.5 (C-6), 53.2 (C-1′), 51.4 (C-1), 31.7, 29.8, 29.0, 28.9, 26.3 (5 × CH_2_′), 22.5 (CH_2_–2′, CH_2_′) 13.7 (C-9′). HRMS calcd for [C_15_H_32_NO_4_]^+^ 290.23258, found 290.23246.

#### *N*-(5-(Nonyloxy)­pentyl)-L-*Alt*-DNM Trifluoroacetic Acid Salt

Prepared in 22% yield following general procedure A (0.383 mmol scale). ^1^H NMR (400 MHz, D_2_O) δ 4.14 (d, *J* = 9.2 Hz, 1H, H-4), 4.09 (s, 1H, H-2), 4.06–3.84 (m, 3H, H-3, CH_2_–6), 3.54–3.36 (m, 5H, H-1a, CH_2_–5′, CH_2_–6′), 3.33 (d, *J* = 8.9 Hz, 1H, H-5), 3.28–3.14 (m, 3H, H-1b, CH_2_–1′), 1.83–1.65 (m, 2H, CH_2_–2′), 1.64–1.47 (m, 4H, CH_2_–4′, CH_2_–7′), 1.42–1.33 (m, 2H, CH_2_–3′), 1.27 (m, 12H, CH_2_–8′–13′), 0.84 (m, 3H, CH_3_-14′). ^13^C NMR (101 MHz, D_2_O) δ 70.8, 70.4 (CH_2_–5′, CH_2_–6′), 68.4 (C-2), 66.0 (C-4), 63.8 (C-3), 61.8 (C-5), 54.5 (C-6), 52.9 (C-1′), 51.3 (C-1), 31.9, 29.6, 29.4, 29.3, 29.2, 28.6, 25.9, 22.8, 22.5 (9 × CH_2_), 22.4 (C-2′), 13.7 (C-14′). HRMS calcd for [C_20_H_42_NO_5_]^+^ 376.3058, found 376.3062.

#### *N*-(5-(Neopentyloxy)­pentyl)-L-*Alt*-DNM Trifluoroacetic Acid Salt

Prepared in 41% yield following general procedure A (0.383 mmol scale). ^1^H NMR (400 MHz, D_2_O) δ 4.26–4.08 (m, 2H, H-2, H-4), 4.08–3.91 (m, 3H, H-3, CH_2_–6), 3.56–3.45 (m, 3H, H-1a, CH_2_–5′), 3.42–3.21 (m, 4H, H-1b, H-5, CH_2_–1′), 3.16 (s, 2H, CH_2_–6′), 1.75 (m, 2H, CH_2_–2′), 1.68–1.56 (m, 2H, CH_2_–4′), 1.48–1.34 (m, 2H, CH_2_–3′), 0.86 (s, 9H, (CH_3_)_3_). ^13^C NMR (101 MHz, D_2_O) δ 81.3 (C-6′), 71.1 (C-5′), 68.1 (C-3), 66.1 (C-2), 63.0 (C-4), 61.2 (C-5), 54.2 (C-6), 53.1 (C-1′), 51.4 (C-1), 31.3 (*C*_q_ *t*Bu), 27.9 (C-4′), 26.0 ((CH_3_)_3_), 22.6 (C-3′), 21.7 (C-2′). HRMS calcd for [C_16_H_34_NO_5_]^+^ 320.24315, found 320.24306.

#### *N*-(5-((Adamantan-1-yl)­methoxy)­pentyl)-L-*Alt*-DNM Trifluoroacetic Acid Salt

Prepared in 23% yield following general procedure A (0.292 mmol scale). ^1^H NMR (400 MHz, D_2_O) δ 4.12 (dd, *J* = 2.4, 10.4 Hz, 1H, H-4), 4.12–4.06 (m, 1H, H-2), 4.02 (d, *J* = 11.8 Hz, 1H, H-6a), 4.00 (s, 1H, H-3) 3.93 (d, *J* = 11.8 Hz, 1H, H-6b), 3.46 (d, *J* = 12.5 Hz, 1H, H-1a), 3.41 (t, *J* = 6.2 Hz, 2H, CH_2_–5′), 3.34–3.15 (m, 4H, H-1b, H-5, CH_2_–1′), 2.99 (s, 2H, CH_2_–6′), 1.90 (s, 3H, 3 × CH Ada), 1.79–1.52 (m, 10H, CH_2_–2′, CH_2_–4′, 3 × CH_2_ Ada), 1.48 (m, 6H, 3 × CH_2_ Ada), 1.43–1.27 (m, 2H, CH_2_–3′). ^13^C NMR (101 MHz, D_2_O) δ 81.7 (C-6′), 71.3 (C-5′), 68.0 (C-3), 66.1 (C-2), 62.9 (C-4), 61.2 (C-5), 54.1 (C-6), 52.9 (C-1′), 51.3 (C-1), 39.3 (3 × CH_2_ Ada), 36.9 (3 × CH_2_ Ada), 33.7 (*C*_q_ Ada), 28.2 (CH_2_–4′), 28.1 (3 × CH Ada), 22.7 (CH_2_–2′), 21.7 (CH_2_–3′). HRMS calcd for [C_22_H_40_NO_5_]^+^ 398.2901, found 398.2903.

#### *N*-(5-([1,1′-Biphenyl]-4-ylmethoxy)­pentyl)-L-*Alt*-DNM Trifluoroacetic Acid Salt

Prepared in 17% yield following general procedure A (0.383 mmol scale). ^1^H NMR (400 MHz, D_2_O) δ 7.24–7.06 (m, 4H, Ar), 7.05–6.78 (m, 5H, Ar), 4.24–4.05 (m, 3H, H-4, CH_2_–6′), 4.04–3.97 (m, 2H, H-2, H-3), 3.94 (d, 1H, *J* = 12.8 Hz, H-6a), 3.77 (d, *J* = 12.8 Hz, 1H, H-6b), 3.22 (d, *J* = 12.4 Hz, 1H, H-1a), 3.35–2.86 (m, 6H, H-1b, H-5, CH_2_–1′, CH_2_–5′), 1.64–1.24 (m, 4H, CH_2_–2′, CH_2_–4′), 1.14–0.94 (m, 2H, CH_2_–3′).^13^C NMR (101 MHz, D_2_O) δ 140.0, 139.6, 137.1 (*C*_q_ Ar), 128.7, 128.4, 127.2, 126.6 (Ar), 71.8 (C-6′), 69.6 (C-5′), 68.0 (C-3), 66.1 (C-2), 62.9 (C-4), 61.4, (C-5), 54.1 (C-6), 52.8 (C-1′), 51.3 (C-1), 28.4 (C-4′), 22.6 (C-3′), 21.7 (C-2′). HRMS calcd for [C_24_H_34_NO_5_]^+^ 416.2432, found 416.2427.

#### *N*-(2-Benzyloxyethyl)-L-*All*-DNM Trifluoroacetic Acid Salt

Prepared in 57% yield following general procedure B, step one. (0.752 mmol scale). ^1^H NMR (400 MHz, D_2_O) δ 7.62–7.20 (m, 5H, Ar), 4.55 (d, *J* = 11.6 Hz, 1H, H-3′a), 4.51 (d, *J* = 11.6 Hz, 1H, H-3′b), 4.10 (t, *J* = 2.7 Hz, 1H, H-4), 4.03–3.89 (m, 3H, H-2, CH_2_–6), 3.84 (m, 3H, H-3, CH_2_–2′), 3.68–3.50 (m, 1H, H-1′a), 3.45–3.15 (m, 4H, CH_2_–1, H-5, CH_2_–1′b). ^13^C NMR (101 MHz, D_2_O) δ 136.7 (*C*_q_ Ar), 128.8, 128.52, 128.47 (Ar), 73.0 (C-3′), 69.4 (C-4), 64.6 (C-3), 63.7 (C-2), 62.9 (C-2′), 61.2 (C-5), 53.5 (C-6), 52.6 (C-1′), 49.2 (C-1). HRMS calcd for [C_15_H_24_NO_5_]^+^ 298.1649, found 298.1654.

#### *N*-(2-Hydroxyethyl)-L-*All*-DNM Trifluoroacetic Acid Salt

Prepared in quantitative yield following general procedure B, step two (0.282 mmol scale). ^1^H NMR (400 MHz, D_2_O) δ 4.16 (t, *J* = 2.6 Hz,1H, H-4), 4.08–3.94 (m, 5H, H-2, CH_2_–H6, CH_2_–2′), 3.90 (dd, *J* = 10.6, 2.6 Hz, 1H, H-3), 3.67–3.50 (m, 1H, CH_2_–1′a), 3.43 (dd, *J* = 11.7, 4.8 Hz, 1H, H-1a), 3.34 (m, 2H, H-5, CH_2_–1′b), 3.26 (app t, *J* = 11.7 Hz, 1H, H-1b). ^13^C NMR (101 MHz, D_2_O) δ 69.5 (C-4), 64.8 (C-3), 63.8 (C-2), 61.4 (C-5), 55.3 (C-2′), 54.6 (C-1′), 54.0 (C-6), 49.3 (C-1). HRMS calcd for [C_8_H_18_NO_5_]^+^ 208.1180, found 208.1183.

#### *N*-(5-(Nonyloxy)­pentyl)-L-*All*-DNM Trifluoroacetic Acid Salt

Prepared in 31% yield following general procedure A (0.250 mmol scale). ^1^H NMR (400 MHz, D_2_O) δ 4.17 (t, *J* = 2.6 Hz, 1H, H-4), 4.08–3.91 (m, 3H, H-2, CH_2_–6), 3.88 (dd, *J* = 10.5, 2.5 Hz, 1H, H-3), 3.50–3.35 (m, 4H, CH_2_–5′, CH_2_–6′), 3.33–3.07 (m, 5H, CH_2_–1, H-5, CH_2_–1′), 1.85–1.68 (m, 2H, CH_2_–2′), 1.67–1.50 (m, 4H, CH_2_–4′, CH_2_–7′), 1.47–1.19 (m, 14H, CH_2_–8′–13′), 0.88 (t, *J* = 6.8 Hz, 3H, CH_3_-14′). ^13^C NMR (101 MHz, D_2_O) δ 70.9 (CH_2_–5′), 70.5 (CH_2_–6′), 69.6 (C-4), 65.2 (C-3), 64.2 (C-2), 60.5 (C-5), 54.3 (C-6), 52.8 (C-1′), 49.2 (C-1), 31.9, 29.7, 29.5, 29.3, 29.3, 28.7, 26.0, 23.0, 22.7 (9 × CH_2_), 22.6 (C-2′), 13.8 (C-14′). HRMS calcd for [C_20_H_42_NO_5_]^+^ 376.3058, found 376.3058.

#### *N*-(5-(Neopentyloxy)­pentyl)-L-*All*-DNM Trifluoroacetic Acid Salt

Prepared in 25% yield following general procedure A (0.250 mmol scale). ^1^H NMR (400 MHz, D_2_O) δ 4.15 (t, *J* = 2.6 Hz, 1H, H-4), 4.07–3.94 (m, 3H, H-2, CH_2_–6), 3.86 (dd, *J* = 10.6, 2.5 Hz, 1H, H-3), 3.52 (t, *J* = 6.5 Hz, 2H, CH_2_–5′), 3.37–3.08 (m, 7H, CH_2_–1, H-5, CH_2_–1′, CH_2_–6′), 1.87–1.67 (m, 2H, CH_2_–2′), 1.66–1.56 (m, 2H, CH_2_–4′), 1.47–1.35 (m, 2H, CH_2_–3′), 0.87 (s, 9H, (CH_3_)_3_). ^13^C NMR (101 MHz, D_2_O) δ 81.3 (C-6′), 71.2 (C-5′), 69.6 (C-4), 65.0 (C-3), 64.0 (C-2), 60.2 (C-5), 53.9 (C-6), 52.9 (C-1′), 48.9 (C-1), 31.3 (*C*_q_ *t*Bu), 27.9 (C-4′), 26.0 (CH_3_)_3_, 22.6 (C-3′), 22.3 (C-2′). HRMS calcd for [C_16_H_34_NO_5_]^+^ 320.2432, found 320.2437.

#### *N*-(5-([1,1′-Biphenyl]-4-ylmethoxy)­pentyl)-L-*All*-DNM Trifluoroacetic Acid Salt

Prepared in 12% yield following general procedure A (0.250 mmol scale). ^1^H NMR (400 MHz, D_2_O) δ 7.39 (s, 2H, Ar), 7.37 (s, 2H, Ar), 7.19 (t, *J* = 7.9 Hz, 4H, Ar), 7.11 (t, *J* = 7.1 Hz, 1H, Ar), 4.32 (s, 2H, CH_2_–6′), 4.15 (s, 1H, H-4), 4.01–3.91 (m, 2H, H-2, H-6a), 3.90–3.76 (m, 2H, H-3, H-6b), 3.30 (t, *J* = 6.2 Hz, 2H, CH_2_–5′), 3.24–2.94 (m, 5H, CH_2_–1, H-5, CH_2_–1′), 1.64–1.51 (m, 2H, CH_2_–2′), 1.51–1.41 (m, 2H, CH_2_–4′), 1.27–1.12 (m, 2H, CH_2_–3′). ^13^C NMR (101 MHz, D_2_O) δ 140.0, 139.9, 137.0 (*C*_q_ Ar), 128.9, 128.8, 127.5, 126.8, 126.7 (Ar), 72.0 (C-6′), 69.7 (C-5′), 69.4 (C-4), 64.8 (C-3), 63.8 (C-2), 60.3 (C-5), 53.9 (C-6), 52.7 (C-1′), 48.8 (C-1), 28.3 (C-4′), 22.6 (C-3′), 22.2 (C-2′). HRMS calcd for [C_24_H_34_NO_5_]^+^ 416.2432, found 416.2425.

#### *N*-Butyl-L-*Gul*-DNM Trifluoroacetic Acid Salt

Prepared in 35% yield following general procedure A (0.086 mmol scale). ^1^H NMR (600 MHz, MeOD) δ 4.07–4.01 (m, 1H, H-2), 3.97 (dd, *J* = 4.4, 2.1 Hz, 1H, H-4), 3.88 (dd, *J* = 11.9, 5.0 Hz, 1H, H-6a), 3.84 (dd, *J* = 11.9, 4.9 Hz, 1H, H-6b), 3.82–3.78 (m, 1H, H-3), 3.04–2.71 (m, 5H, CH_2_–1, H-5, CH_2_–1′), 1.66–1.51 (m, 2H, CH_2_–2′), 1.41–1.29 (m, 2H, CH_2_–3′), 0.97 (t, *J* = 7.4 Hz, 3H, CH_3_-4′). ^13^C NMR (151 MHz, MeOD) δ 72.6 (C-4), 71.5 (C-3), 66.3 (C-2), 61.3 (C-6), 61.1 (C-5), 54.5 (C-1′), 52.4 (C-1), 26.9 (C-2′), 21.5 (C-3′), 14.2 (C-4′). HRMS calcd for [C_15_H_32_NO_4_]^+^ 290.23258, found 290.23272.

#### *N*-(2′-Hydroxyethyl)-L-*Gul*-DNM HCl Salt

2,3,4,6-Di*-O*-isopropylidene-L-*Gul*-DNM (255 mg, 1.05 mmol) was dissolved in THF (15 mL), and subsequently, a solution of benzyloxyacetaldehyde (268 mg, 1.79 mmol) in THF (5 mL) and acetic acid (200 μL) were added. The mixture was stirred for 20 min before NaBH­(OAc)_3_ (300 mg, 1.42 mmol) was added as a solid. After 16 h, LC-MS analysis proved complete conversion of the starting material, and the mixture was diluted with ethyl acetate (120 mL), washed with aqueous 10% w/v Na_2_CO_3_ solution (2 × 20 mL) and brine (20 mL), dried over MgSO_4_, filtered, and concentrated. The resulting brownish oil was purified by silica gel column chromatography, using pentane:EtOAc 9:1 → 4:1 to afford *N*-2′-benzyloxyethyl-2,3,4,6-di*-O*-isopropylidene-L-*Gul*-DNM as a colorless oil (312 mg) in 79% yield. ^1^H NMR (400 MHz, CDCl_3_) δ 7.37–7.21 (m, 5H, Ar), 4.50 (s, 2H, CH_2_Ph), 4.28 (td, *J* = 6.2, 4.4 Hz, 1H, H-2), 4.21–4.15 (m, 1H, H-4), 4.11 (dd, *J* = 6.2, 2.0 Hz, 1H, H-3), 3.92 (dd, *J* = 12.5, 2.7 Hz, 1H, CH_2_–6a), 3.86 (dd, *J* = 12.5, 3.0 Hz, 1H, CH_2_–6b), 3.63–3.48 (m, 2H, CH_2_–2′), 3.24 (dd, *J* = 13.3, 4.2 Hz, 1H, H-1a), 3.12–2.92 (m, 2H, CH_2_–1′), 2.67 (dd, *J* = 13.3, 6.2 Hz, 1H, H-1b), 2.65–2.61 (m, 1H, H-5), 1.48 (s, 3H, CH_3_), 1.43 (s, 3H, CH_3_), 1.40 (s, 3H, CH_3_), 1.33 (s, 3H, CH_3_). ^13^C NMR (101 MHz, CDCl_3_) δ 138.3 (*C*_q_ Ar), 128.3, 127.6, 127.5 (Ar), 108.2, 98.9 (OCO), 74.3 (C-3), 73.1 (CH_2_Ph), 71.8 (C-2), 69.0 (C-2′), 67.1 (C-4), 62.8 (C-6) 55.4 (C-1′), 53.4 (C-5), 51.8 (C-1), 28.6, 27.3, 24.9, 19.5 (CH_3_). This compound was dissolved in a mixture of MeOH (20 mL) and 8 M aqueous HCl (3 mL). After 5 min, Pd/C-10% (70 mg) was added and the flask sealed with a septum. A balloon filled with H_2_ was placed on top of the flask and stirring was continued over the weekend. After filtration over a Whatman filter, the solvents were evaporated to give the target iminosugar (135 mg) in 98% yield as a colorless foam. ^1^H NMR (400 MHz, D_2_O) δ 4.34–4.25 (m, 1H, H-2), 4.21 (s, 1H, H-4), 4.10–3.88 (m, 5H, H-3, CH_2_–6, CH_2_–2′), 3.64 (m, 1H, H-5), 3.57 (dt, *J* = 13.8, 5.3 Hz, 1H, H-1′a), 3.52–3.37 (m, 2H, N-1′b, H-1a), 3.32 (t, *J* = 10.7 Hz, 1H, H-1b). ^13^C NMR (101 MHz, D_2_O) δ 70.2 (C-4), 68.2 (C-3), 62.7 (C-2), 60.7 (C-5), 58.7 (C-6), 55.2 (C-2′, C-1′), 50.0 (C-1). HRMS calcd for [C_8_H_18_NO_5_]^+^ 208.11795, found 208.11788.

#### *N*-Nonyl-L-*Gul*-DNM Trifluoroacetic Acid Salt

Prepared in 31% yield following general procedure A (0.097 mmol scale). ^1^H NMR (400 MHz, MeOD) δ 4.13 (ddd, *J* = 9.0, 6.3, 2.9 Hz, 1H, H-2), 4.02 (dd, *J* = 4.7, 2.1 Hz, 1H, H-4), 3.99–3.88 (m, 2H, CH_2_–6), 3.84 (dd, *J* = 4.6, 2.9 Hz, 1H, H-3), 3.25 (td, *J* = 4.8, 2.2 Hz, 1H, H-5), 3.12–2.99 (m, 4H, CH_2_–1, CH_2_–1′), 1.81–1.59 (m, 2H, CH_2_–2′), 1.42–1.23 (m, 12H, CH_2_–3′–8′), 0.91 (t, *J* = 6.8 Hz, 3H, CH_3_-9′). ^13^C NMR (100 MHz, MeOD) δ 72.2 (C-4), 70.8 (C-3), 65.0 (C-2), 61.5 (C-5), 60.9 (C-6), 54.7 (C-1′), 51.6 (C-1), 33.0, 30.6, 30.4, 30.4, 28.0, 24.2, 23.7 (7 × CH_2_), 14.4 (C-9′). HRMS calcd for [C_15_H_32_NO_4_]^+^ 290.23258, found 290.23272.

#### *N*-(5′-(Nonyloxy)­pentyl)-L-*Gul*-DNM Trifluoroacetic Acid Salt

Prepared in 30% yield following general procedure A (0.100 mmol scale). ^1^H NMR (500 MHz, MeOD) δ 4.20 (ddd, *J* = 11.0, 5.2, 2.8 Hz, 1H, H-2), 4.11–3.87 (m, 1H, H-4), 3.97 (dd, *J* = 12.9, 4.0 Hz, 1H, CH_2_–6a), 3.92 (dd, *J* = 12.9, 4.8 Hz, 1H, CH_2_–6b), 3.90–3.87 (m, 1H, H-3), 3.45 (t, *J* = 6.6 Hz, 2H, CH_2_–5′), 3.47–3.43 (m, 1H, H-5), 3.42 (t, *J* = 6.6 Hz, 2H, CH_2_–6′), 3.31–3.22 (m, 2H, CH_2_–1′), 3.23–3.12 (m, 2H, CH_2_–1), 1.90–1.79 (m, 1H, CH_2_–2′a), 1.78–1.70 (m, 1H, CH_2_–2′b), 1.67–1.60 (m, 2H, CH_2_–4′), 1.56 (p, *J* = 6.7 Hz, 2H, CH_2_–7′), 1.49–1.40 (m, 2H, CH_2_–3′), 1.39–1.22 (m, 12H, CH_2_–8′–13′), 0.90 (t, *J* = 6.9 Hz, 3H, CH_3_–14′). ^13^C NMR (126 MHz, MeOD) δ 72.3 (C-4), 72.0 (C-6′), 71.4 (C-5′), 70.0 (C-3), 64.1 (C-2), 61.7 (C-5), 61.1 (C-6), 55.0 (C-1′), 51.2 (C-1), 33.0 (CH_2_), 30.7 (CH_2_), 30.7 (CH_2_), 30.6 (C-5′), 30.4 (CH_2_), 30.1 (C-4′), 27.3 (CH_2_), 24.5 (C-3′), 23.7 (CH_2_), 23.0 (C-2′), 14.4 (C-14′). HRMS calcd for [C_20_H_42_NO_5_]^+^ 376.30575, found 376.30576.

#### *N*-(5-(Neopentyloxy)­pentyl)-L-*Gul*-DNM Trifluoroacetic Acid Salt

Prepared in 35% yield following general procedure A (0.105 mmol scale). ^1^H NMR (300 MHz, MeOD) δ 4.26–4.14 (m, 1H, H-2), 4.06 (s, 1H, H-4), 3.99–3.91 (m, 2H, CH_2_–6), 3.88 (dd, *J* = 4.4, 2.8 Hz, 1H, H-3), 3.52–3.40 (m, 1H, H-5), 3.45 (t, *J* = 6.1 Hz, 2H, CH_2_–5′), 3.34–3.14 (m, 4H, CH_2_–1′, CH_2_–1), 3.08 (s, 2H, CH_2_–6′), 1.93–1.70 (m, 2H, CH_2_–2′), 1.64 (dt, *J* = 12.0, 6.2 Hz, 2H, CH_2_–4′), 1.46 (p, *J* = 6.9, 6.4 Hz, 2H, CH_2_–3′), 0.91 (s, 9H, (CH_3_)_3_). ^13^C NMR (75 MHz, MeOD) δ 82.5 (C-6′), 72.1 (C-4), 70.1 (C-5′), 64.1 (C-3), 61.8 (C-2), 61.1 (C-5), 55.0 (C-6), 51.2 (C-1′), 49.9 (C-1), 32.9 (*C*_q_ *t*Bu), 30.2 (C-4′), 27.1 ((CH_3_)_3_), 24.6 (C-3′), 23.1 (C-2′). HRMS calcd for [C_16_H_34_NO_5_]^+^ 320.24315, found 320.24299.

#### *N*-(5-((Adamantan-1-yl)­methoxy)­pentyl)-L-*Gul*-DNM Trifluoroacetic Acid Salt

Prepared in 75% yield following general procedure A (0.105 mmol scale). ^1^H NMR (500 MHz, MeOD) δ 4.20 (ddd, *J* = 10.6, 4.9, 2.7 Hz, 1H, H-2), 4.08 (d, *J* = 3.1 Hz, 1H, H-4), 3.97 (dd, *J* = 12.9, 3.8 Hz, 1H, CH_2_–6a), 3.93 (dd, *J* = 12.9, 4.7 Hz, 1H, CH_2_–6b), 3.89 (dd, *J* = 4.2, 3.0 Hz, 1H, H-3), 3.46 (br s, 1H, H-5), 3.41 (t, *J* = 6.2 Hz, 2H, CH_2_–5′), 3.32–3.12 (m, 4H, CH_2_–1′, CH_2_–1), 2.98 (s, 2H, CH_2_–6′), 1.95 (br s, 3H, 3 × CH Ada), 1.90–1.79 (m, 1H, CH_2_–2a′), 1.76 (m, 4H, CH_2_–2b′, 3 × CH_2_ Ada), 1.71–1.59 (m, 5H, 3 × CH_2_ Ada, CH_2_–4′), 1.59–1.52 (m, 6H, 3 × CH_2_ Ada), 1.45 (p, *J* = 7.3, 6.9 Hz, 2H, CH_2_–3′). ^13^C NMR (126 MHz, MeOD) δ 83.1 (C-6′), 72.3 (C-4), 72.1 (C-5′), 70.0 (C-3), 64.1 (C-2), 61.7 (C-5), 61.1 (C-6), 55.0 (C-1′), 51.2 (C-1), 40.8 (CH_2_ Ada), 38.3 (CH_2_ Ada), 35.1 (*C*_q_ Ada), 30.1 (C-4′), 29.7 (CH Ada), 24.6 (C-3′), 23.0 (C-2′). HRMS calcd for [C_22_H_40_NO_5_]^+^ 398.29010, found 398.29004.

#### *N*-(5-([1,1′-Biphenyl]-4-ylmethoxy)­pentyl)-L-*Gul*-DNM Trifluoroacetic Acid Salt

Prepared in 38% yield following general procedure A (0.421 mmol scale). ^1^H NMR (400 MHz, MeOD) δ 7.65–7.51 (m, 4H, Ar), 7.46–7.37 (m, 4H, Ar), 7.37–7.28 (m, 1H, Ar), 4.53 (s, 2H, CH_2_–6′), 4.20 (ddd, *J* = 10.6, 5.2, 2.7 Hz, 1H, H-2), 4.11–4.05 (m, 1H, H-4), 4.01–3.82 (m, 3H, CH_2_–6, H-3), 3.54 (t, *J* = 6.2 Hz, 2H, CH_2_–5′), 3.44 (br s, 1H, H-5), 3.37–3.08 (m, 4H, CH_2_–1′, CH_2_–1), 1.93–1.61 (m, 4H, CH_2_–2′, CH_2_–4′), 1.53–1.41 (m, 2H, CH_2_–3′). ^13^C NMR (101 MHz, MeOD) δ 142.0, 141.8, 138.8 (*C*_q_ Ar), 130.0, 129.8, 129.5, 129.3, 128.5, 128.0, 127.9 (Ar), 73.6 (C-6′), 72.2 (C-4), 71.0 (C-5′), 70.0 (C-3), 64.0 (C-2), 61.7 (C-5), 61.1 (C-6), 54.9 (C-1′), 51.2 (C-1), 30.1 (C-4′), 24.5 (C-2′), 23.0 (C-3′). HRMS calcd for [C_24_H_34_NO_5_]^+^ 416.24315, found 416.24312.

#### *N*-(2-Hydroxyethyl)-L-*Gal*-DNM Trifluoroacetic Acid Salt

*N*-Boc-2-*O*-TBDPS-3,4-*O*-isopropylidene-6*O*-benzyl-L-*Gal*-DNM^9b^ (307 mg, 0.486 mmol) was dissolved in dry DCM (5 mL), and TFA (2 mL) was added dropwise in 2 min. The mixture was stirred for 25 min and subsequently diluted with toluene and concentrated. The residue was dissolved in diethyl ether, washed with 0.3 M NaOH (15 mL) and brine (10 mL), dried over MgSO_4_, filtered, and concentrated to give a pale brown oil (235 mg). NMR and LCMS analysis indicated full removal of the Boc group and 40% loss of the acetonide. The mixture was dissolved in THF (10 mL), and benzyloxyacetic aldehyde (85 mg, 0.570 mmol) and acetic acid (100 μL, 1.65 mmol) were added. After 5 min, NaBH­(OAc)_3_ (150 mg, 0.707 mmol) was added, and the reaction was left stirring. After 4 h, LC-MS confirmed full conversion, and the reaction was diluted with ethyl acetate (50 mL) and washed with aqueous 10% w/v Na_2_CO_3_ solution (2 × 20 mL) and brine (20 mL), dried over MgSO_4_, filtered, and concentrated. The residue was dissolved in a mixture of acetone (7 mL) and 2,2-dimethoxypropane (3 mL), and *p*-toluenesulfonic acid (140 mg) was added; the reaction was stirred overnight. LCMS confirmed complete reinstallation of the acetonide, and the mixture was diluted with ethyl acetate (50 mL) and washed with aqueous 10% w/v Na_2_CO_3_ solution (2 × 20 mL) and brine (20 mL), dried over MgSO_4_, filtered, and concentrated. Purification by silica gel column chromatography, using pentane:EtOAc 95:5 → 9:1 → 3:1, afforded 400 mg of product that was dissolved in THF (10 mL) and, after addition of 1 M TBAF (0.9 mL), left stirring over the weekend. The reaction mixture was then diluted with ethyl acetate, washed with water (5 mL) and brine (5 mL), dried over MgSO_4_, filtered, and concentrated. The crude material was purified by silica gel column chromatography, using pentane:EtOAc 9:1 → 3:1 → 1:1 → 0:1 to afford the target compound (146 mg) in 70% overall yield. ^1^H NMR (400 MHz, CDCl_3_) δ 7.41–7.19 (m, 10H, Ar), 4.50 (s, 2H, CH_2_Ph), 4.48 (s, 2H, CH_2_Ph), 4.31–4.25 (m, 1H, H-4), 3.86–3.76 (m, 3H, H-2, H-3, H-6a), 3.72 (dd, *J* = 9.6, 5.7 Hz, 1H, H-6b), 3.65–3.49 (m, 2H, CH_2_–2′), 3.20 (br s, 1H, OH), 3.09–2.98 (m, 3H, H-5, H-1a, H-1′a), 2.88 (dt, *J* = 14.2, 5.6 Hz, 1H, H-1′b), 2.37–2.26 (m, 1H, H-1b), 1.50 (s, 3H, CH_3_), 1.34 (s, 3H, CH_3_). ^13^C NMR (101 MHz, CDCl_3_) δ 138.19, 138.15 (*C*_q_ Ar), 128.41, 128.39, 127.69, 127.65 (Ar), 109.1 (*C*_q_ acetonide), 79.8 (C-3), 75.1 (C-4), 73.3, 73.2 (CH_2_Ph), 70.2 (C-6), 69.8 (C-2), 67.6 (C-2′), 59.5 (C-5), 55.1 (C-1), 52.3 (C-1′), 28.4 (CH_3_), 26.0 (CH_3_). This product (145 mg, 0.342 mmol) was dissolved in methanol (20 mL) and, after addition of aqueous 6 M HCl (2 mL) and Pd/C-10% (50% w/w water, 220 mg), hydrogenated at 4 bar in a Parr apparatus for 20 h. The mixture was filtered over a Whatman filter and concentrated to give the title compound in quantitative yield. Purification by RP-HPLC afforded the target compound (81 mg) in 74% yield. ^1^H NMR (400 MHz, D_2_O) δ 4.24 (dd, *J* = 3.1, 1.7 Hz, 1H, H-4), 4.09 (ddd, *J* = 11.1, 9.6, 4.9 Hz, 1H, H-2), 4.01 (dd, *J* = 13.0, 5.0 Hz, 1H, H-6a), 3.97 (dd, *J* = 13.0, 5.0 Hz, 1H, H-6b), 3.95 (t, *J* = 5.2 Hz, 2H, CH_2_–2′), 3.65 (dd, *J* = 12.5, 5.0 Hz, 1H, H-1a), 3.62 (dd, *J* = 9.6, 3.2 Hz, 1H, H-3), 3.59–3.50 (m, 2H, H-5, H-1′a), 3.38 (dt, *J* = 14.1, 5.1 Hz, 1H, H-1′b), 3.04 (dd, *J* = 12.5, 11.2 Hz, 1H, H-1b). ^13^C NMR (101 MHz, D_2_O) δ 72.6 (C-3), 69.4 (C-4), 65.1 (C-5), 64.1 (C-2), 58.6 (C-6), 55.3 (C-2′), 54.2 (C-1′), 53.5 (C-1). HRMS calcd for [C_8_H_18_NO_5_]^+^ 208.1180, found 208.1182.

#### *N*-(5-(Nonyloxy)­pentyl)-L-*Gal*-DNM Trifluoroacetic Acid Salt

Prepared in 39% yield following general procedure A (0.251 mmol scale). ^1^H NMR (400 MHz, D_2_O) δ 4.28–4.17 (s, 1H, H-4), 4.06 (td, *J* = 10.4, 4.9 Hz, 1H. H-2), 4.00–3.87 (m, 2H, CH_2_–6), 3.58 (dd, *J* = 9.4, 2.9 Hz, 1H, H-3), 3.51–3.30 (m, 6H, H-1a, H-5, CH_2_–5′, CH_2_–6′), 3.30–3.09 (m, 2H, CH_2_–1′), 2.89 (t, *J* = 11.6 Hz, 1H, H-1b), 1.72 (s, 2H, CH_2_–2′), 1.64–1.45 (m, 4H, CH_2_–4′, CH_2_–7′), 1.44–1.15 (m, 14H, CH_2_–3′, CH_2_–8′–13′), 0.90–0.78 (m, 3H, CH_3_-14′). ^13^C NMR (101 MHz, D_2_O) δ 72.8 (C-3), 70.8 (C-5′), 70.5 (C-6′), 69.6 (C-4), 64.6 (C-2), 64.2 (C-5), 59.1 (C-6), 53.4 (C-1), 52.9 (C-1′), 31.9, 29.7, 29.5, 29.4, 29.3, 28.7, 26.0, 22.9, 22.6 (9 × CH_2_), 22.2 (C-2′), 13.8 (C-14′). HRMS calcd for [C_20_H_42_NO_5_]^+^ 376.30575, found 376.30578.

#### *N*-(5-(Neopentyloxy)­pentyl)-L-*Gal*-DNM Trifluoroacetic Acid Salt

Prepared in 45% yield following general procedure A (0.316 mmol scale). ^1^H NMR (400 MHz, D_2_O) δ 4.27–4.20 (m, 1H, H-4), 4.05 (td, *J* = 10.6, 5.0 Hz, 1H, H-2), 3.95 (d, *J* = 4.3 Hz, 2H, CH_2_–6), 3.57 (dd, *J* = 9.8, 3.0 Hz, 1H, H-3), 3.53 (m, 3H, H-1a, CH_2_–5′), 3.42 (t, *J* = 4.6 Hz, 1H, H-5), 3.37–3.17 (m, 2H, CH_2_–1′), 3.14 (s, 2H, CH_2_–6′), 2.95 (t, *J* = 11.8 Hz, 1H, H-1b), 1.85–1.64 (m, 2H, CH_2_–2′), 1.64–1.53 (m, 2H, CH_2_–4′), 1.45–1.30 (m, 2H, CH_2_–3′), 0.84 (s, 9H, (CH_3_)_3_). ^13^C NMR (101 MHz, D_2_O) δ 81.2 (C-6′), 72.7 (C-3), 71.1 (C-5′), 69.9 (C-4), 64.5 (C-2), 64.0 (C-5), 59.2 (C-6), 53.4 (C-1), 53.3 (C-1′), 31.3 (C-4′), 27.8 (*C*_q_ *t*Bu), 25.9 ((CH_3_)_3_), 22.5 (C-3′), 21.6 (C-2′). HRMS calcd for [C_16_H_34_NO_5_]^+^ 320.24315, found 320.24297.

#### *N*-(5-([1,1′-Biphenyl]-4-ylmethoxy)­pentyl)-L-*Gal*-DNM Trifluoroacetic Acid Salt

Prepared in 31% yield following general procedure A (0.311 mmol scale). ^1^H NMR (400 MHz, D_2_O) δ 7.26 (s, 2H, Ar), 7.25 (s, 2H, Ar), 7.06 (m, 4H, Ar), 6.98 (t, *J* = 7.1 Hz, 1H, Ar), 4.22 (s, 2H, CH_2_–6′), 4.16 (s, 1H, H-4), 4.01 (m, 1H, H-2), 3.92–3.78 (m, 2H, CH_2_–6), 3.48 (d, *J* = 8.4 Hz, 1H, H-3), 3.33 (d, *J* = 7.7 Hz, 1H, H-1a), 3.27–3.13 (m, 3H, CH_2_–5′, H-5), 3.13–3.02 (m, 1H, CH_2_–1′a), 3.12–2.90 (m, 1H, CH_2_–1′b), 2.73 (t, *J* = 11.4 Hz, 1H, H-1b), 1.59–1.45 (m, 2H, CH_2_–2′), 1.44–1.30 (m, 2H, CH_2_–4′), 1.19–1.01 (m, 2H, CH_2_–3′). ^13^C NMR (101 MHz, D_2_O) δ 140.0, 139.8, 137.0 (*C*_q_ Ar), 128.8, 128.6, 127.4, 126.7, 126.6 (Ar), 72.7 (C-3), 71.9 (C-6′), 69.6 (C-5′), 69.5 (C-4), 64.4 (C-2), 64.0 (C-5), 59.0 (C-6), 53.2 (C-1), 52.8 (C-1′), 28.3 (C-4′), 22.6 (C-3′), 21.9 (C-2′). HRMS calcd for [C_24_H_34_NO_5_] 416.24315, found 416.24291.

#### *N*-Butyl-L-*Tal*-DNM Trifluoroacetic Acid Salt

Prepared in 52% yield following general procedure A (0.110 mmol scale). ^1^H NMR (400 MHz, D_2_O) δ 4.29 (s, 1H, H-4), 4.25 (s, 1H, H-2), 4.06 (dd, *J* = 12.9, 5.2 Hz, 1H, H-6a), 4.00 (dd, *J* = 12.9, 4.8 Hz, 1H, H-6b), 3.87 (s, 1H, H-3), 3.58 (d, *J* = 13.6 Hz, 1H, H-1a), 3.48 (s, 1H, H-5), 3.40 (d, *J* = 13.5 Hz, 1H, H-1b), 3.32 (p, *J* = 4.1 Hz, 2H, CH_2_–1′), 1.74 (m, 2H, CH_2_–2′), 1.39 (h, *J* = 7.3 Hz, 2H, CH_2_–3′), 0.94 (t, *J* = 7.4 Hz, 3H, CH_3_-4′). ^13^C NMR (101 MHz, D_2_O) δ 70.0 (C-4), 67.0 (C-3), 66.9 (C-2), 64.5 (C-5), 59.2 (C-6), 55.5 (C-1), 53.2 (C-1′), 23.9 (C-2′), 19.3 (C-3′), 12.8 (C-4′). HRMS calcd for [C_10_H_22_NO_4_]^+^ 220.1543, found 220.1547.

#### *N*-(2-Benzyloxyethyl)-L-*Tal*-DNM Trifluoroacetic Acid Salt

Prepared in 47% yield following general procedure B, step one (0.154 mmol scale). ^1^H NMR (500 MHz, D_2_O) δ 7.51–7.36 (m, 5H, Ar), 4.61 (s, 2H, CH_2_–3′), 4.23 (s, 1H, H-4), 4.18 (s, 1H, H-2), 4.04 (dd, *J* = 13.1, 5.6 Hz, 1H, CH_2_–6a), 4.00 (dd, *J* = 13.0, 4.5 Hz, 1H, CH_2_–6b), 3.96–3.88 (m, 2H, CH_2_–2′), 3.84 (t, *J* = 3.6 Hz, 1H, H-3), 3.72–3.56 (m, 2H, H-1a, H-1′a), 3.56–3.46 (m, 2H, H-1′b, H-5), 3.36 (d, *J* = 13.2 Hz, 1H, H-1b). ^13^C NMR (126 MHz, D_2_O) δ 136.9 (*C*_q_ Ar), 128.9, 128.6, 128.6 (Ar), 73.1 (C-3′), 69.8 (C-4), 67.2 (C-3), 66.5 (C-2), 65.5 (C-5), 63.1 (C-2′), 59.1 (C-6), 55.9 (C-1), 52.7 (C-2′). HRMS calcd for [C_24_H_34_NO_5_]^+^ 298.1649, found 298.1656.

#### *N*-(2-Hydroxyethyl)-L-*Tal*-DNM Trifluoroacetic Acid Salt

Prepared in 98% yield following general procedure B, step two (0.073 mmol scale). ^1^H NMR (500 MHz, D_2_O) δ 4.28 (s, 1H, H-4), 4.25 (s, 1H, H-2), 4.09 (dd, *J* = 13.1, 5.6 Hz, 1H, H-6a), 4.04 (dd, *J* = 13.1, 4.5 Hz, 1H, H-6b), 4.04–3.96 (m, 2H, CH_2_–2′), 3.91 (s, 1H, H-3), 3.77–3.68 (m, 1H, H-1a), 3.66–3.57 (m, 2H, H-5, CH_2_–1′a), 3.47 (m, 2H, H-1b, CH_2_–1′b). ^13^C NMR (126 MHz, D_2_O) δ 69.9 (C-4), 67.1 (C-3), 66.5 (C-2), 65.8 (C-5), 59.2 (C-6), 55.8 (C-1), 55.2 (C-2′), 54.6 (C-1′). HRMS calcd for [C_8_H_18_NO_5_]^+^ 208.11795, found 208.11799.

#### *N*-Nonyl-L-*Tal*-DNM Trifluoroacetic Acid Salt

Prepared in 18% yield following general procedure A (0.112 mmol scale). ^1^H NMR (400 MHz, D_2_O) δ 4.22 (s, 1H, H-4), 4.18 (s, 1H, H-2), 4.01 (dd, *J* = 12.9, 5.4 Hz, 1H, H-6a), 3.95 (dd, *J* = 12.9, 4.7 Hz, 1H, H-6b), 3.83 (t, *J* = 3.4 Hz, 1H, H-3), 3.48 (d, *J* = 13.6 Hz, 1H, H-1a), 3.39 (s, 1H, H-5), 3.35–3.11 (m, 3H, H-1b, CH_2_–1′), 1.82–1.56 (m, 2H, CH_2_–2′), 1.42–1.15 (m, 12H, CH_2_–3′–8′), 0.90–0.75 (t, *J* = 6.4 Hz, 3H, CH_3_-9′). ^13^C NMR (101 MHz, MeOD) δ 70.7 (C-4), 68.4 (C-3), 67.9 (C-2), 65.1 (C-5), 60.0 (C-6), 54.4 (C-1), 32.1, 29.4, 29.3, 29.2, 26.8, 23.0 (6 × CH_2_), 14.4 (C-9′). HRMS calcd for [C_15_H_32_NO_4_]^+^ 290.2326, found 290.2331.

#### *N*-(5-(Nonyloxy)­pentyl)-L-*Tal*-DNM Trifluoroacetic Acid Salt

Prepared in 8% yield following general procedure A (0.299 mmol scale). ^1^H NMR (400 MHz, D_2_O) δ 4.17 (s, 1H, H-4), 4.12 (s, 1H, H-2), 4.04–3.86 (m, 2H, CH_2_–6), 3.79 (s, 1H, H-3), 3.59–3.31 (m, 5H, H-1a, CH_2_–5′, CH_2_–6′), 3.26 (s, 1H, H-5), 3.24–2.97 (m, 3H, H-1b, CH_2_–1′), 1.86–1.46 (m, 6H, CH_2_–2′, CH_2_–4′, CH_2_–7′), 1.42–1.12 (m, 14H, CH_2_–3′, CH_2_–8′–13′), 0.92–0.74 (m, 3H, CH_3_-14′). ^13^C NMR (101 MHz, D_2_O) δ 70.8 (C-5′), 70.5 (C-6′), 69.7 (C-4), 67.7 (C-3), 67.1 (C-2), 64.1 (C-5), 54.5 (C-1), 53.1 (C-1′), 32.0, 31.8, 29.6, 29.4, 29.3, 28.7, 25.9, 23.0, 22.7, 22.6 (10 × CH_2_), 13.8 (C-14′). HRMS calcd for [C_20_H_42_NO_5_]^+^ 376.30575, found 376.30578.

#### *N*-(5-(Neopentyloxy)­pentyl)-L-*Tal*-DNM Trifluoroacetic Acid Salt

Prepared in 12% yield following general procedure A (0.223 mmol scale). ^1^H NMR (400 MHz, D_2_O) δ 4.28 (s, 1H, H-4), 4.24 (s, 1H, H-2), 4.07 (dd, *J* = 12.9, 5.5 Hz, 1H, H-6a), 4.01 (dd, *J* = 13.0, 4.6 Hz, 1H, H-6b), 3.88 (t, *J* = 3.4 Hz, 1H, H-3), 3.60- 3.50 (m, 3H, H-1a, CH_2_–5′), 3.46 (br s, 1H, H-5), 3.41–3.24 (m, 3H, H-1b, CH_2_–1′), 3.21 (s, 2H, CH_2_–6′), 1.88–1.70 (m, 2H, CH_2_–2′), 1.69–1.60 (m, 2H, CH_2_–4′), 1.50–1.38 (m, 2H, CH_2_–3′), 0.91 (s, 9H, C­(CH_3_)_3_).^13^C NMR (101 MHz, D_2_O) δ 81.3 (C-6′), 71.2 (C-5′), 70.0 (C-4), 67.3 (C-3), 66.9 (C-2), 64.4 (C-5), 59.4 (C-6), 55.1 (C-1), 53.3 (C-1′), 31.3 (*C*_q_ *t*Bu), 27.9 (C-4′), 26.0 ((CH_3_)_3_), 22.6 (C-3′), 22.0 (C-2′). HRMS calcd for [C_16_H_34_NO_5_]^+^ 320.24315, found 320.24281.

#### *N*-(5-((Adamantan-1-yl)­methoxy)­pentyl)-L-*Tal*-DNM Trifluoroacetic Acid Salt

Prepared in 36% yield following general procedure A (0.281 mmol scale). ^1^H NMR (400 MHz, D_2_O) δ 4.25 (s, 1H, H-4), 4.21 (s, 1H, H-2), 4.01 (dd, *J* = 12.8, 4.9 Hz, 1H, H-6a), 3.95 (dd, *J* = 12.8, 4.4 Hz, 1H, H-6b), 3.83 (d, *J* = 3.5 Hz, 1H, H-3), 3.51 (d, *J* = 11.9 Hz, 1H, H-1a), 3.47–3.38 (m, 3H, H-5, CH_2_–5′), 3.35 (d, *J* = 13.1 Hz, 1H, H-1b), 3.30–3.19 (m, 2H, CH_2_–1′), 2.99 (s, 2H, CH_2_–6′), 1.91 (s, 3H, 3 × CH Ada), 1.81–1.53 (m, 10H, 3 × CH_2_ Ada, CH_2_–2′, CH_2_–4′), 1.52–1.46 (m, 6H, 3 × CH_2_ Ada), 1.42–1.28 (m, 2H, CH_2_–3′). ^13^C NMR (101 MHz, D_2_O) δ 81.8 (C-6′), 71.4 (C-5′), 70.0 (C-4), 66.92 (C-3), 66.86 (C-2), 64.8 (C-5), 59.5 (C-6), 55.6 (C-1), 53.2 (C-1′), 39.4 (3 × CH_2_ Ada), 37.0 (3 × CH_2_ Ada), 33.8 (*C*_q_ Ada), 28.3 (C-4′), 28.2 (3 × CH Ada), 22.7 (C-3′), 21.8 (C-2′). HRMS calcd for [C_16_H_34_NO_5_]^+^ 398.2901, found 398.2901.

#### *N*-(5-([1,1′-Biphenyl]-4-ylmethoxy)­pentyl)-L-*Tal*-DNM Trifluoroacetic Acid Salt

Prepared in 11% yield following general procedure A (0.223 mmol scale). ^1^H NMR (400 MHz, D_2_O) δ 7.57 (d, *J* = 7.7 Hz, 4H, Ar), 7.42–7.34 (m, 4H, Ar), 7.30 (t, *J* = 7.3 Hz, 1H, Ar), 4.47 (s, 2H, CH_2_–6′), 4.20 (s, 1H, H-4), 4.09 (s, 1H, H-2), 3.98 (dd, *J* = 13.0, 5.3 Hz, 1H, H-6a), 3.92 (dd, *J* = 13.0, 4.0 Hz, 1H, H-6b), 3.70 (s, 1H, H-3), 3.53–3.37 (m, 3H, H-1a, CH_2_–5′), 3.31 (s, 1H, H-5), 3.25–3.12 (m, 3H, H-1b, CH_2_–1′), 1.70–1.49 (m, 4H, CH_2_–2′, CH_2_–4′), 1.32 (m, 2H, CH_2_–3′). ^13^C NMR (101 MHz, D_2_O) δ 140.1, 140.0, 137.0 (*C*_q_ Ar), 129.2, 127.8, 127.0, 126.8­(Ar), 72.1 (C-6′), 70.0 (C-4), 69.4 (C-5′), 66.9 (C-3), 66.8 (C-2), 64.5 (C-5), 59.3 (C-6), 55.4 (C-1), 53.1 (C-1′), 28.1 (C-4′), 22.4 (C-3′), 21.4 (C-2′). HRMS calcd for [C_24_H_34_NO_5_]^+^ 416.24315, found 416.24223.

### Enzyme Inhibition Assays

#### GBA1

Recombinant human GBA1 (rhGBA1, 6.5 ng of Cerezyme dissolved in 12.5 μL buffer) was incubated with an iminosugar solution (0–1000 μM dissolved in 12.5 μL water containing 5% DMSO) for 30 min at 37 °C. Then, 100 μL of 3.7 mM 4-methylumbelliferyl-β-d-glucopyranoside (4MU-β-d-glucopyranoside) in 150 mM McIlvaine buffer pH 5.2, 0.1% BSA (w/v), 0.1% Triton X-100 (v/v), and 0.2% taurocholate (w/v) were added, and the mixture was incubated for another 30 min. The reaction was then quenched by the addition of 200 μL of 1 M glycine-NaOH (pH 10.3) solution, and liberated 4-MU fluorescence was quantified with a LS55 fluorimeter (PerkinElmer) using λ_Ex_ 366 nm and λ_Em_ 445 nm. Values plotted for log­[inhibitor] correspond to those in the final reaction mixture containing [Enz] + [Inh] + [Subs]. Measurements were performed in technical triplicate. The activity detected in the presence of test compounds at various concentrations was expressed as activity percentage of that observed with the vehicle control. Results were processed and analyzed using GraphPad Prism (v 9.0.0) utilizing nonlinear regression and best fit model between log­(inhibitor) versus normalized response and log­(inhibitor) versus normalized response–variable slope.

#### GBA2

Freshly prepared homogenate (12.5 μL) of stable HEK293T cells overexpressing GBA2 containing 0.5 μg/μL total protein was preincubated for 30 min at 37 °C with MDW933 ^17^ (specific GBA1 inhibitor, final concentration 0.1 μM). Next, the homogenate was incubated with dissolved iminosugar (0 to 100 μM dissolved in 12.5 μL water containing 5% DMSO) for 30 min. Then, 100 μL of 3.7 mM 4MU-β-d-glucopyranoside in 150 mM McIlvaine buffer pH 5.8, 0.1% BSA (w/v) was added, and the mixture was incubated for another 60 min. Iminosugar-dependent 4-MU liberation was detected as described for GBA1.

#### GCS

RAW cells were seeded at 0.8 × 10^6^ cells/mL in 12-well plates and grown in RPMI medium with 10% FCS (v/v), 0.1% penicillin/streptomycin (w/v), and 1% Glutamax (v/v) under 5% CO_2_ at 37 °C. Once confluent, the cells were preincubated for 1 h with 300 μM conduritol B epoxide (CBE, selective GBA1 inhibitor), followed by 1 h incubation with 0.8 nmol *N*-[7-(4-nitrobenzo-2-oxa-1,3-diazole)]-6-aminocaproyl-D-erythro-sphingosine (C6-NBD-Cer), fatty acid free bovine serum albumin and iminosugar (0 to 100 μM dissolved in 12.5 μL water containing 0.05% DMSO). Cells were then washed 3× with HBSS and harvested by scraping. Next, lipids were extracted using the Bligh and Dyer method by addition of methanol, chloroform, and water (1:1:0.9, v/v/v). Lipid separation was performed in an Ultimate 3000 HPLC system (ThermoScientific) using a BDS-Hypersil C18 column by applying an isocratic elution of MeOH:H_2_O (85:15 v/v). NBD-lipids were detected by fluorescence (λ_Ex_ 460 nm and λ_Em_ 540 nm) and quantified using Chromeleon 7 software. Measurements were performed in technical duplicates. The activity detected in the presence of test compounds at various concentrations is expressed as % of that observed with the vehicle control. Results were processed and analyzed using GraphPad Prism 9.0.

## Supplementary Material





## Notes

Experimental details of compound synthesis and inhibition studies, IC_50_ curves, HPLC-MS chromatograms, ^1^H and ^13^C NMR spectra (PDF)
Molecular formula strings and IC_50_ values (CSV)

## Abbreviations Used

*All*: allose
*Alt*: altrose
AMP: adamantanemethyloxypentyl
BiPhe: biphenylmethyloxypentyl
BnBr: benzyl bromide
BOC: *tert*-butyloxycarbonyl
BSA: Bovine Serum Albumin
DiBAL-H: diisobutylaluminum hydride
DMF: dimethylformamide
DMSO: dimethyl sulfoxide
DNM: deoxynojirimycin
[Enz]: enzyme concentration
ER: Endoplasmic Reticulum
FCS: Fetal Calf Serum
*Gal*: galactose
α-GalA: α-galactosidase A
GBA1: acid glucosylceramidase
GBA2: neutral glucosylceramidase
GCS: glucosylceramide synthase
*Glc*, glucose; *Gul*, gulose; HBSS: Hank’s balanced salt solution
HPLC: high-performance liquid chromatography
IC_50_: Half-maximal inhibitory concentration
*Ido*: idose
[Inh]: inhibitor concentration
*Man*: mannose
MS: mass spectrometry
*p*TsOH: para-toluenesulfonic acid
[Subs]: substrate concentration
*Tal*: talose
TBAF: tetrabutylammonium fluoride
THF: tetrahydrofuran
TIC; total ion count; *Tx*GH116: β-glucosidase enzyme from the bacterium *Thermoanaerobacterium xylanolyticum*
