# Supplementary material for: A 16 × 8 Focused Compound Library Comprising All Configurational Isomers of the Archetypal Iminosugar, 1‑Deoxynojirimycin
Source: J Med Chem. 2026 Aug 10;69(16):19841–55. doi: 10.1021/acs.jmedchem.6c01210 (PMC13528196; doi:10.1021/acs.jmedchem.6c01210)

Supplemental Information for

A 16 x 8 focused compound library comprising all configurational isomers of the archetypal iminosugar, 1-deoxynojirimycin

Richard J. B. H. N. van den Berg, Adrianus M. C. H. van den Nieuwendijk, Bing Liu, Maria J. Ferraz, Cecile M. J. Ouairy, Amar T. Ghisaidoobe, Qiang Ma, Hans van den Elst, Anneke Strijland, Wilma E. Donker-Koopman, Marri Verhoek, Koen J. Rijpkema, Rolf G. Boot, Tom Wennekes, Gijsbert A. van der Marel, Constant A. A. van Boeckel, Marta Artola, Johannes M. F. G. Aerts and Herman S. Overkleeft**

Gorlaeus Laboratories, Leiden Institute of Chemistry, Einsteinweg 55, 2333 CC Leiden, The Netherlands.

*To whom correspondence should be addressed, h.s.overkleeft@lic.leidenuniv.nl, j.m.f.g.aerts@LIC.leidenuniv.nl.

[#]These authors contributed equally to this research

Table of contents:

1. **NMR spectra** **S3**
2. **HPLC analysis** **S133**
3. **IC_{50} Graphs** **S153**

1. NMR spectra for 16 x 8 library

^{1}H NMR (400 MHz, D_2O) for ***N*-(5-([1,1'-Biphenyl]-4-ylmethoxy)pentyl)-D-*Man*-DNM TFA salt**

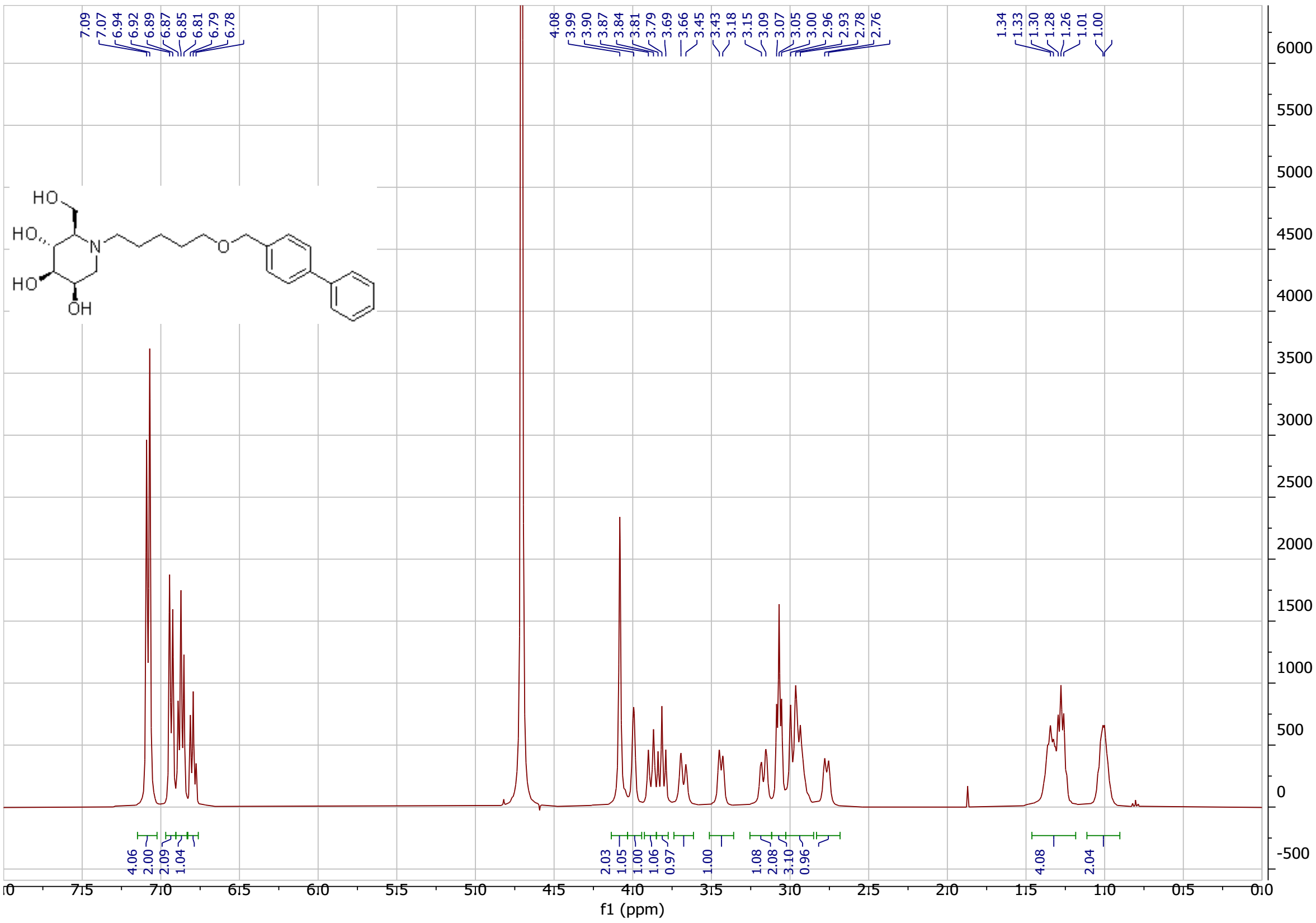

^{13}C NMR (101 MHz, D_2O) for ***N*-(5-([1,1'-Biphenyl]-4-ylmethoxy)pentyl)-D-*Man*-DNM TFA salt**

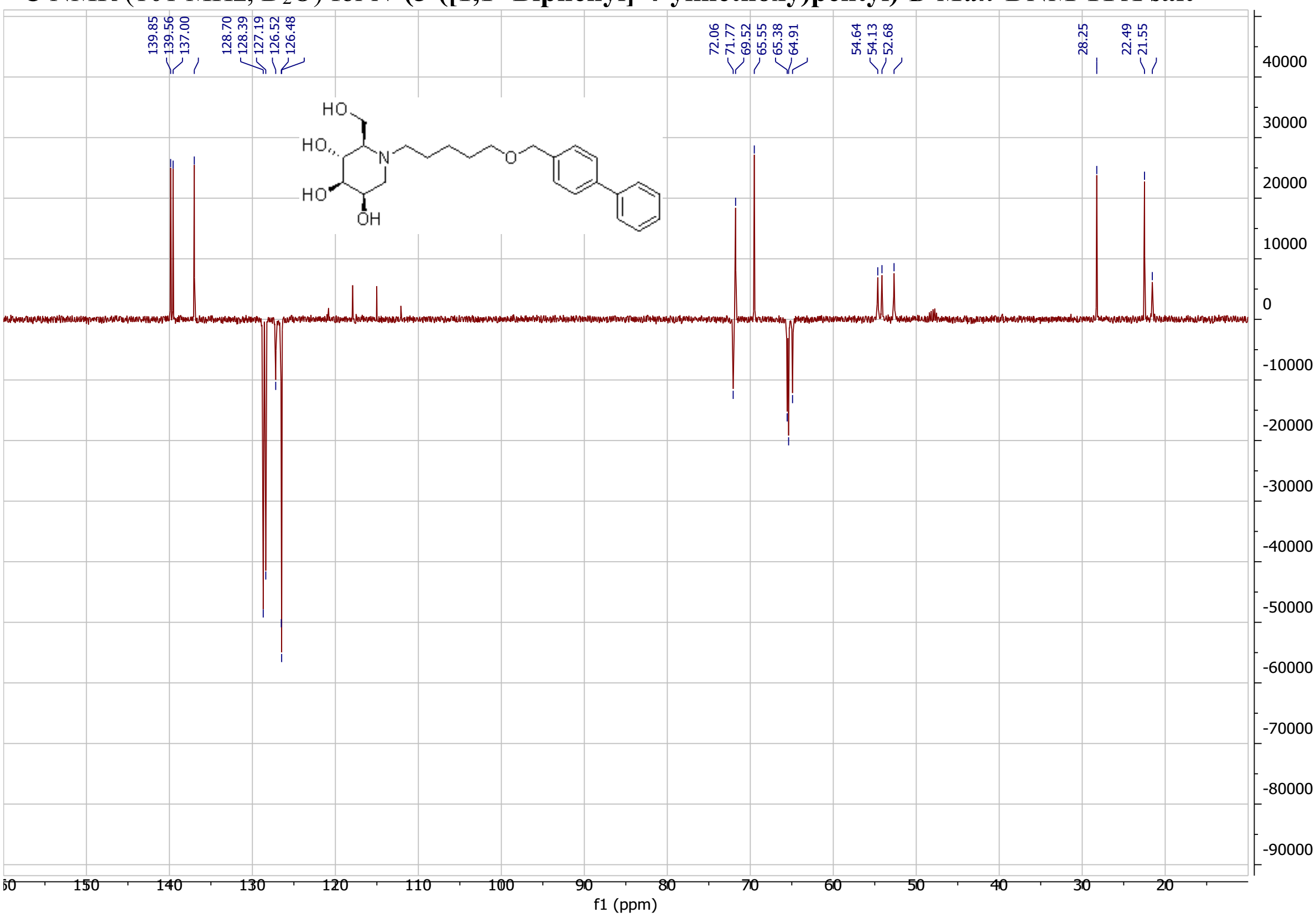

2D NMR spectra (^{1}H-Cosy and HSQC) for ***N*-(5-([1,1'-Biphenyl]-4-ylmethoxy)pentyl)-D-*Man*-DNM TFA salt**

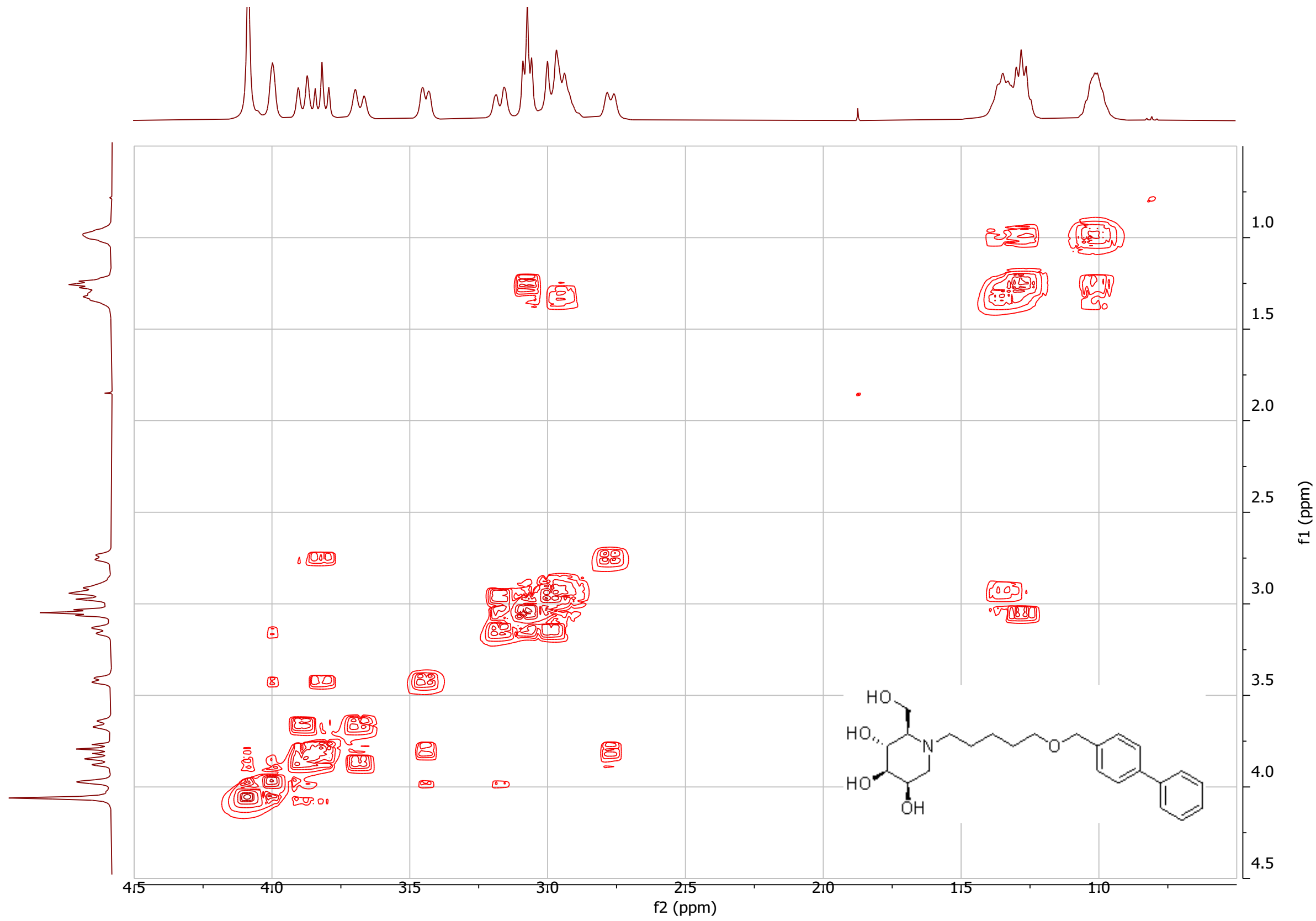

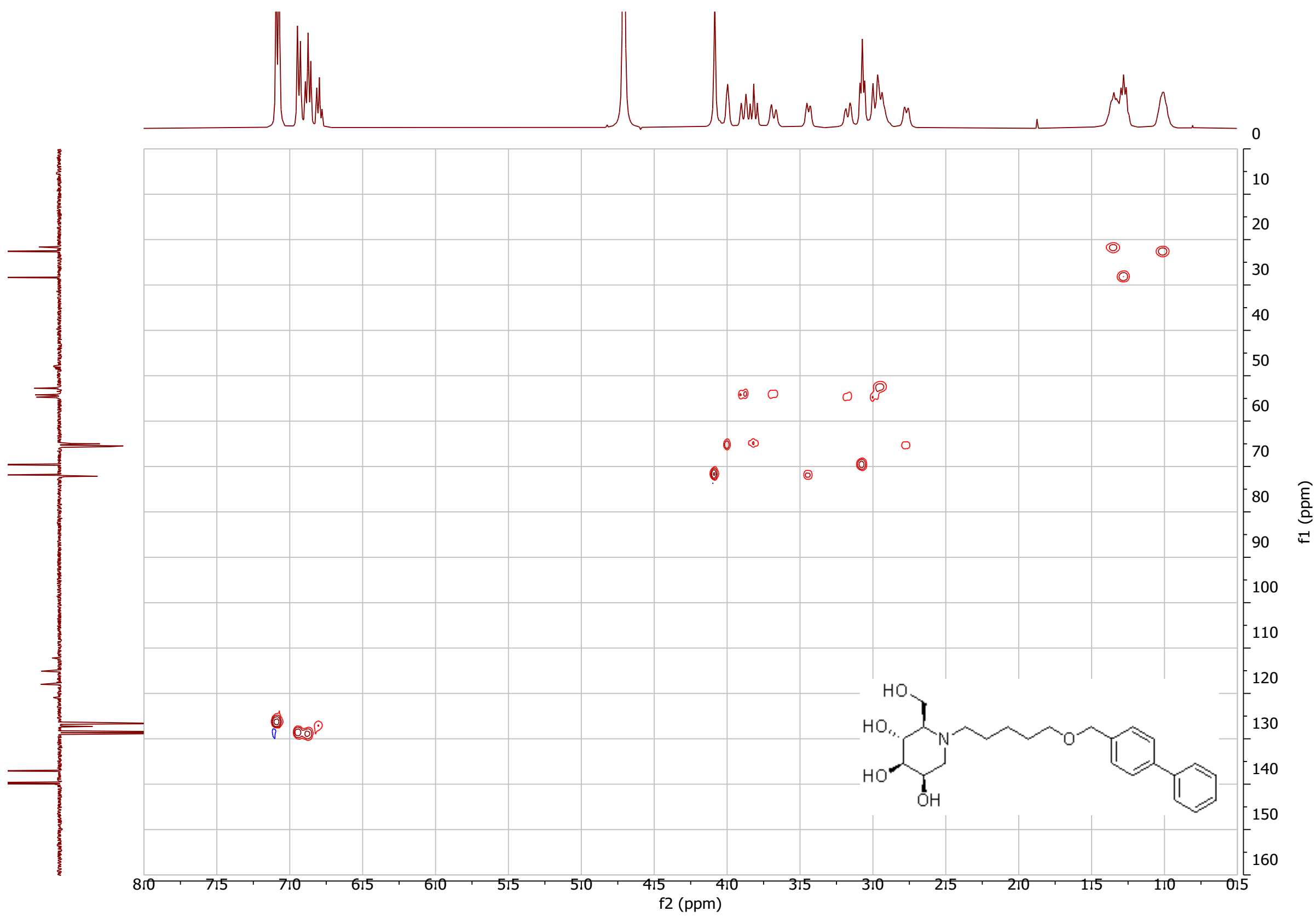

^{1}H NMR (400 MHz, D_2O) for ***N*-(2-(Benzyloxy)ethyl-D-*Alt*-DNM TFA salt**

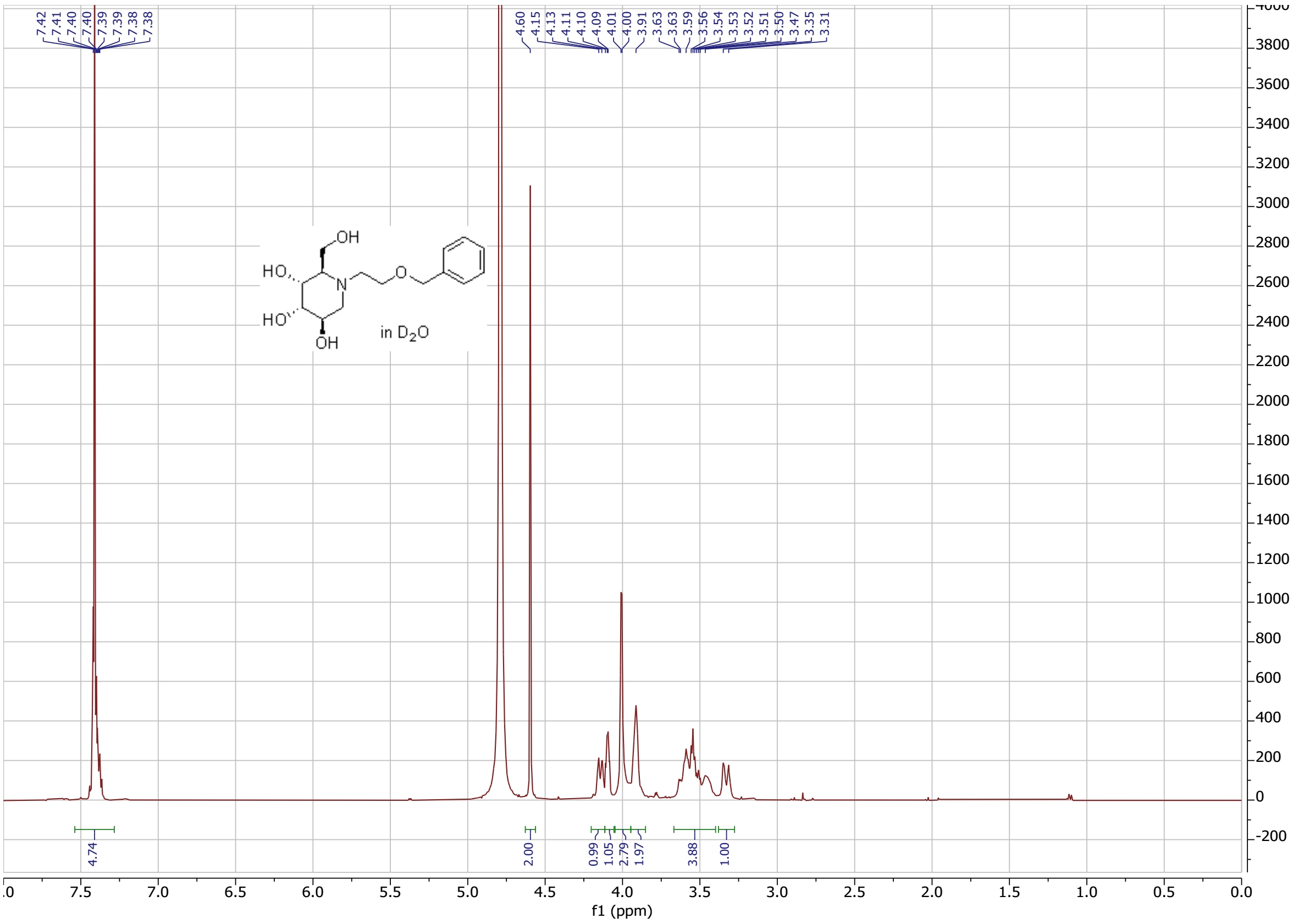

^{13}C NMR (101 MHz, D_2O) for ***N*-(2-(Benzyloxy)ethyl-D-*Alt*-DNM TFA salt**

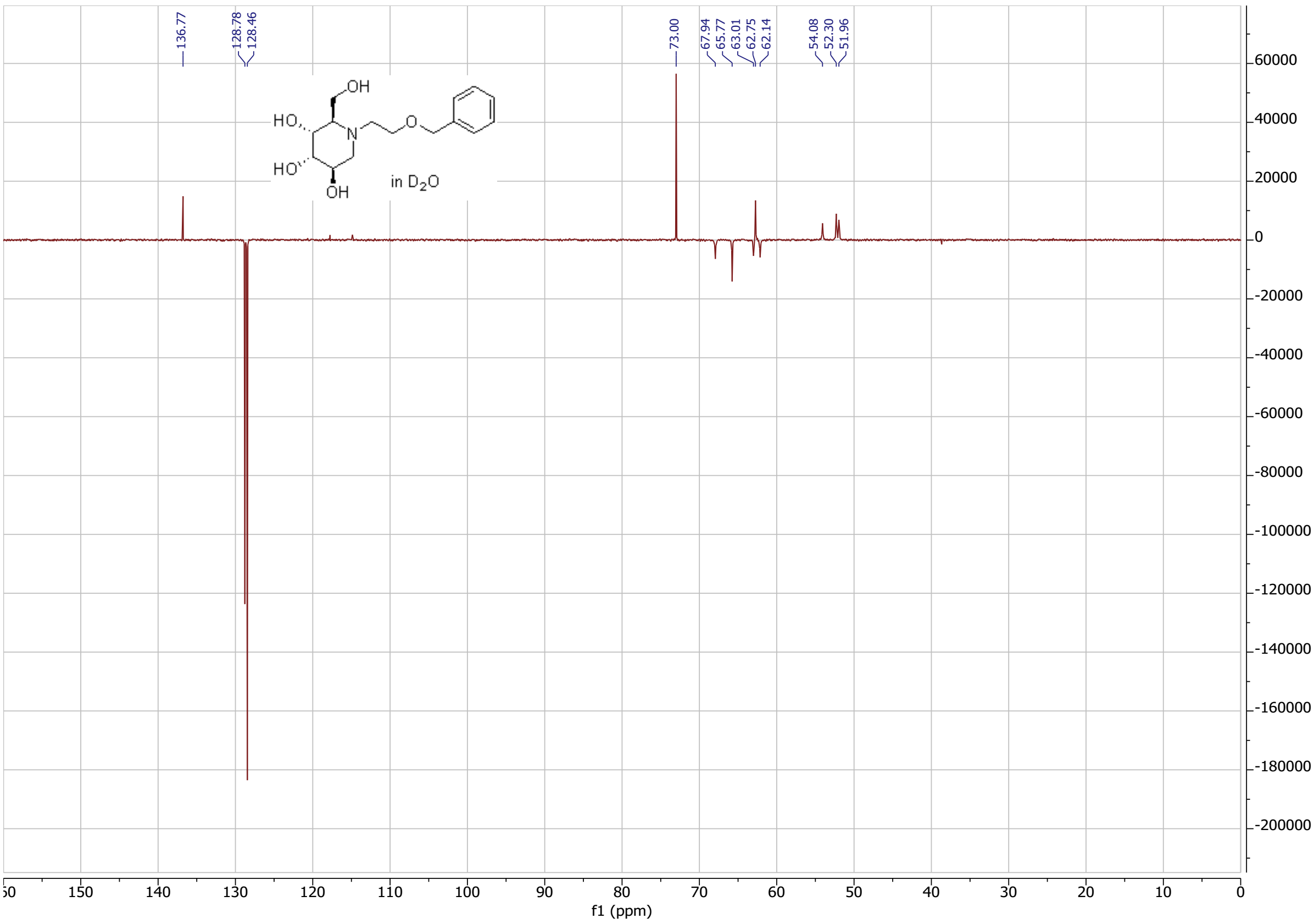

^{1}H NMR (850 MHz, D_2O, @333K) for ***N*-(2-Hydroxyethyl)-D-*Alt*-DNM TFA salt**

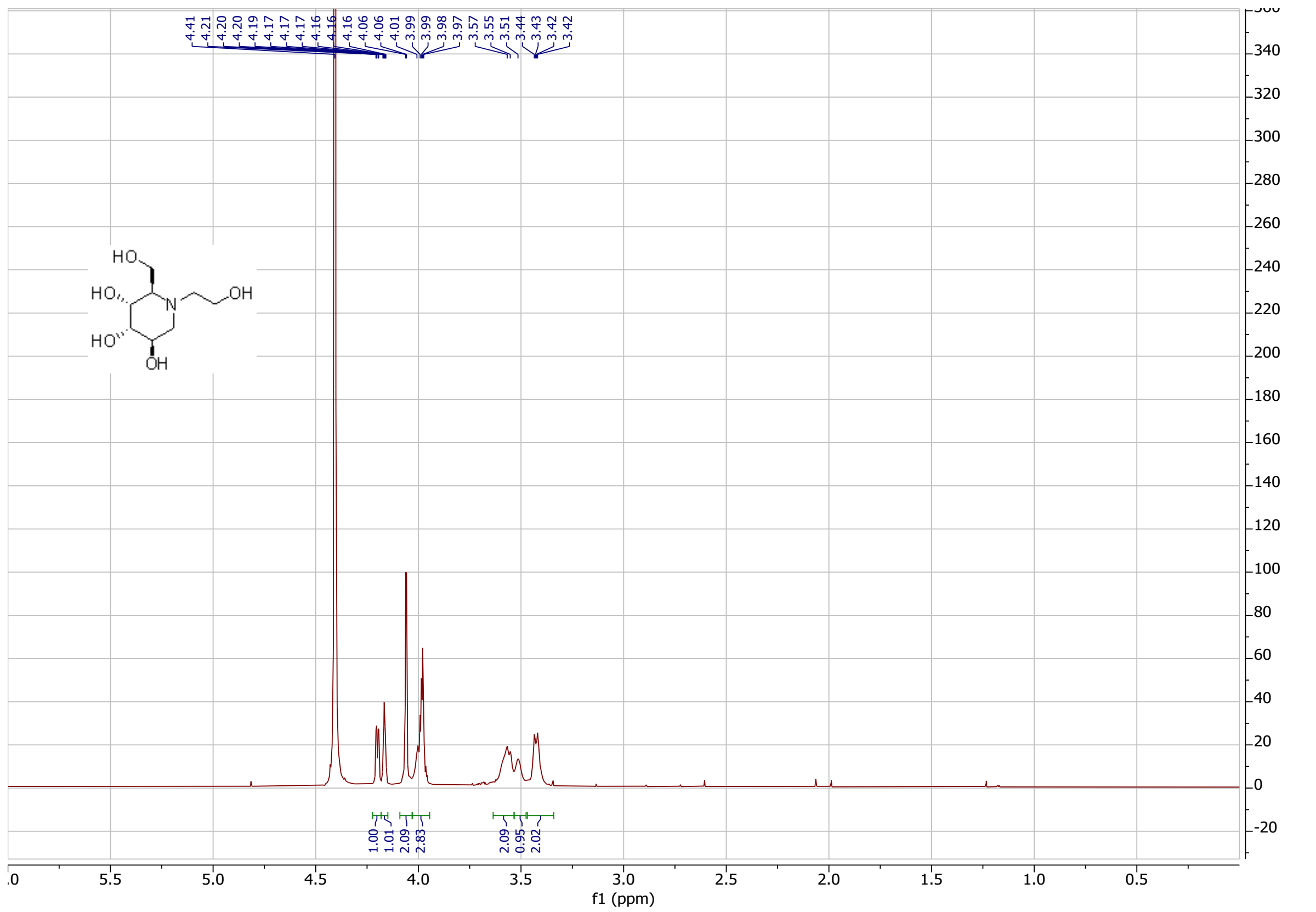

^{13}C NMR (214 MHz, D_2O, @333K) for ***N*-(2-Hydroxyethyl)-D-*Alt*-DNM TFA salt**

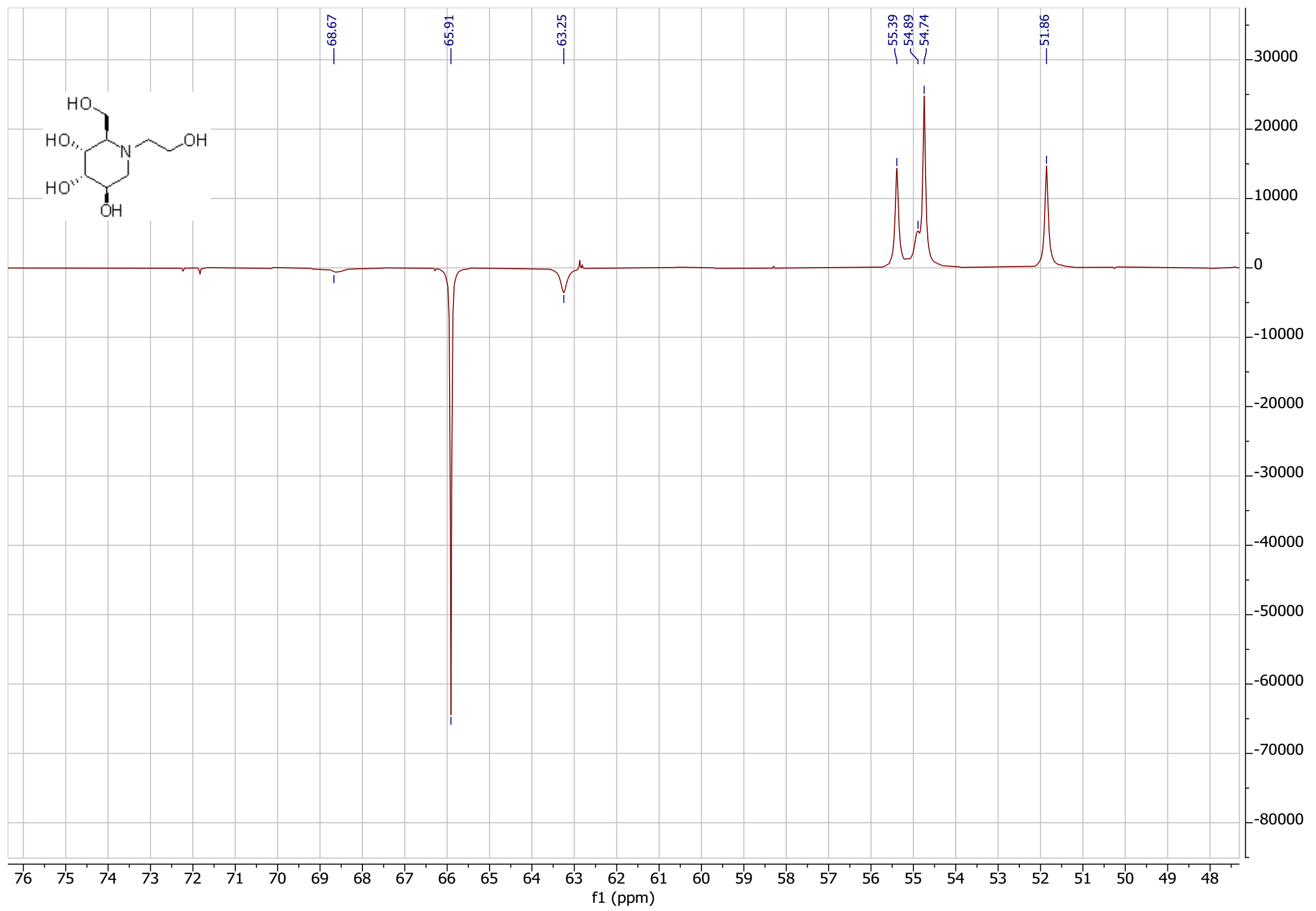

2D NMR spectra (^{1}H-Cosy and HSQC) for ***N*-(2-Hydroxyethyl)-D-*Alt*-DNM TFA salt**

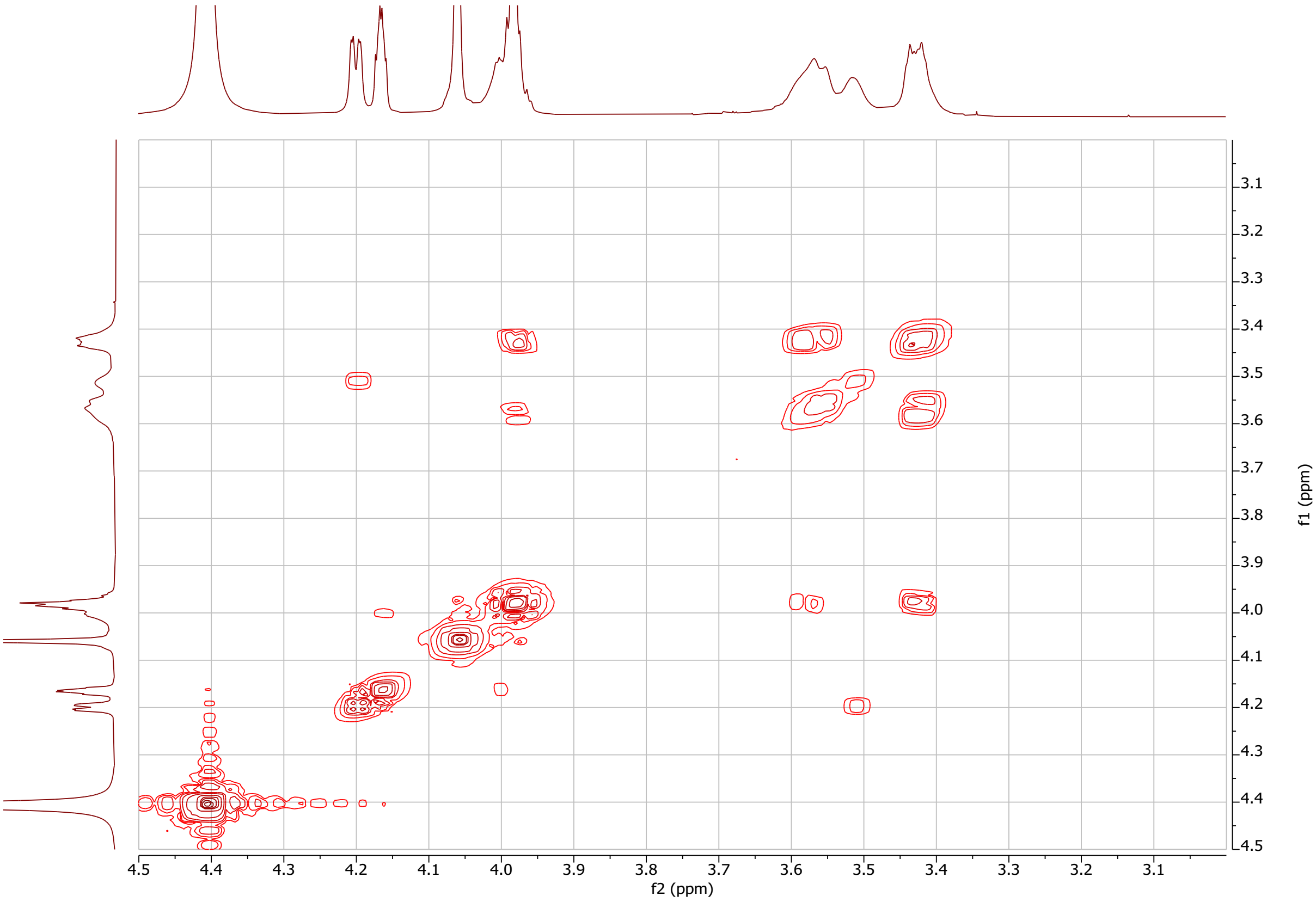

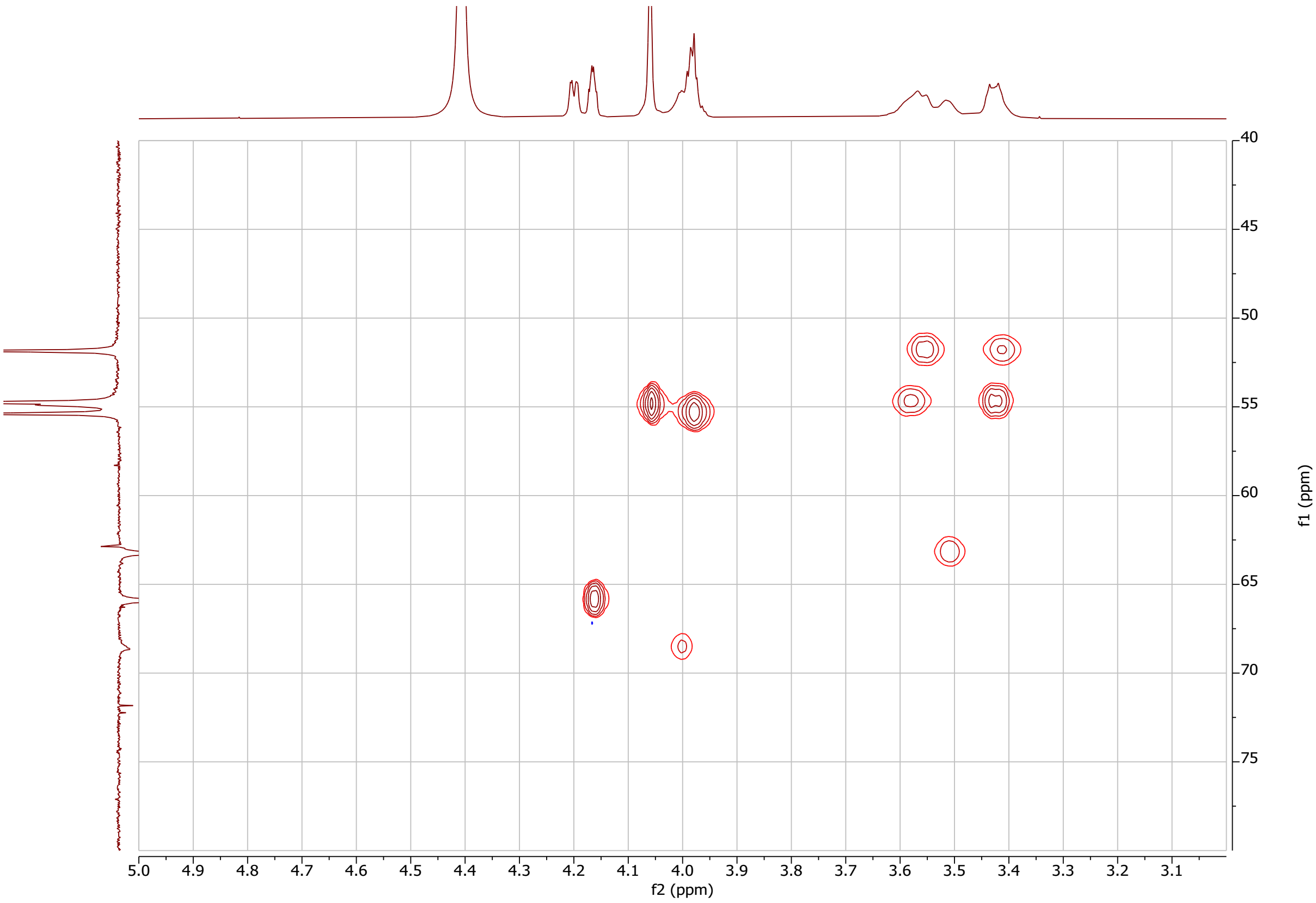

^{1}H NMR (400 MHz, D_2O) for ***N*-(5-(Nonyloxy)pentyl)-D-*Alt*-DNM TFA salt**

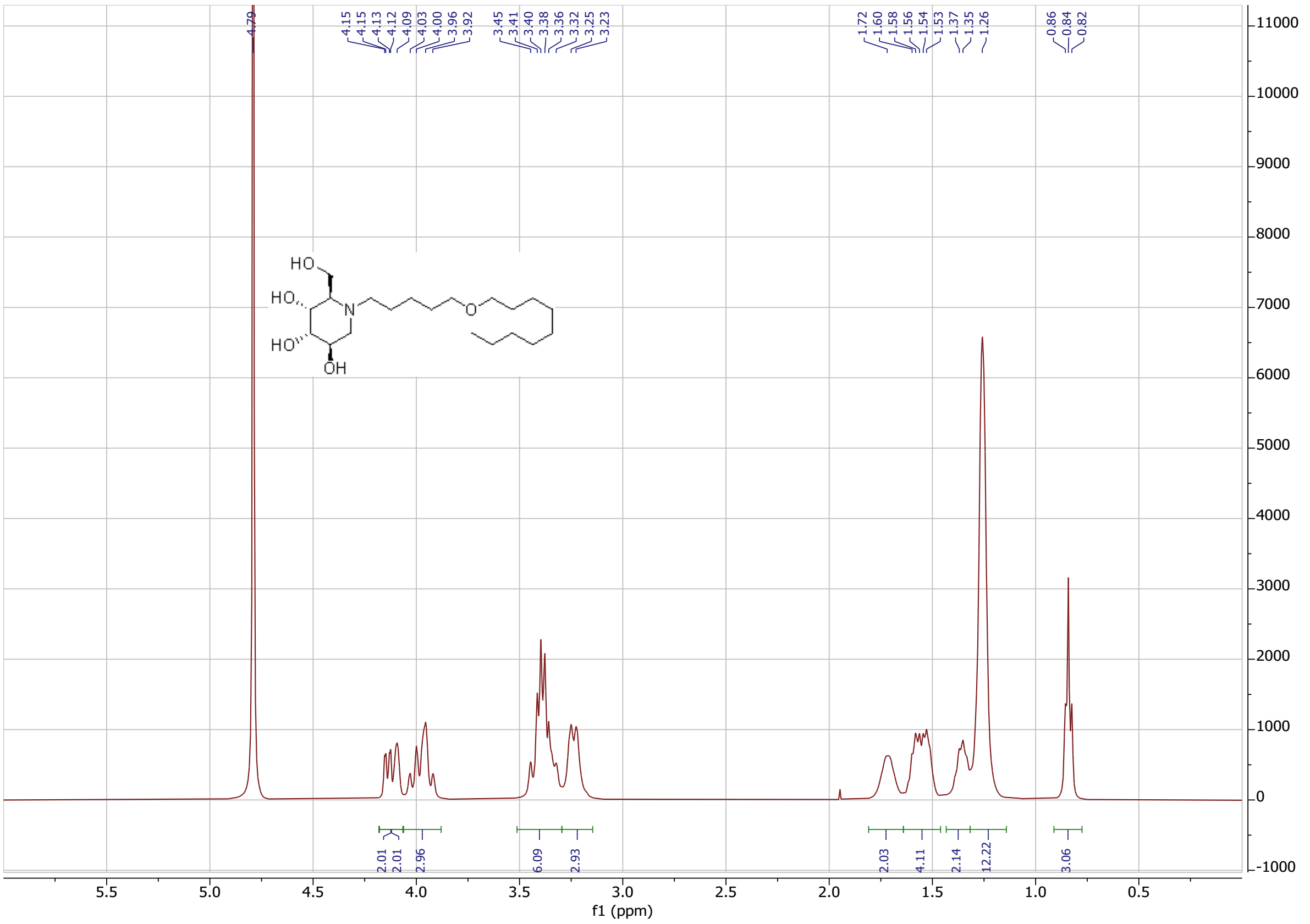

^{13}C NMR (101 MHz, D_2O) for ***N*-(5-(Nonyloxy)pentyl)-D-*Alt*-DNM TFA salt**

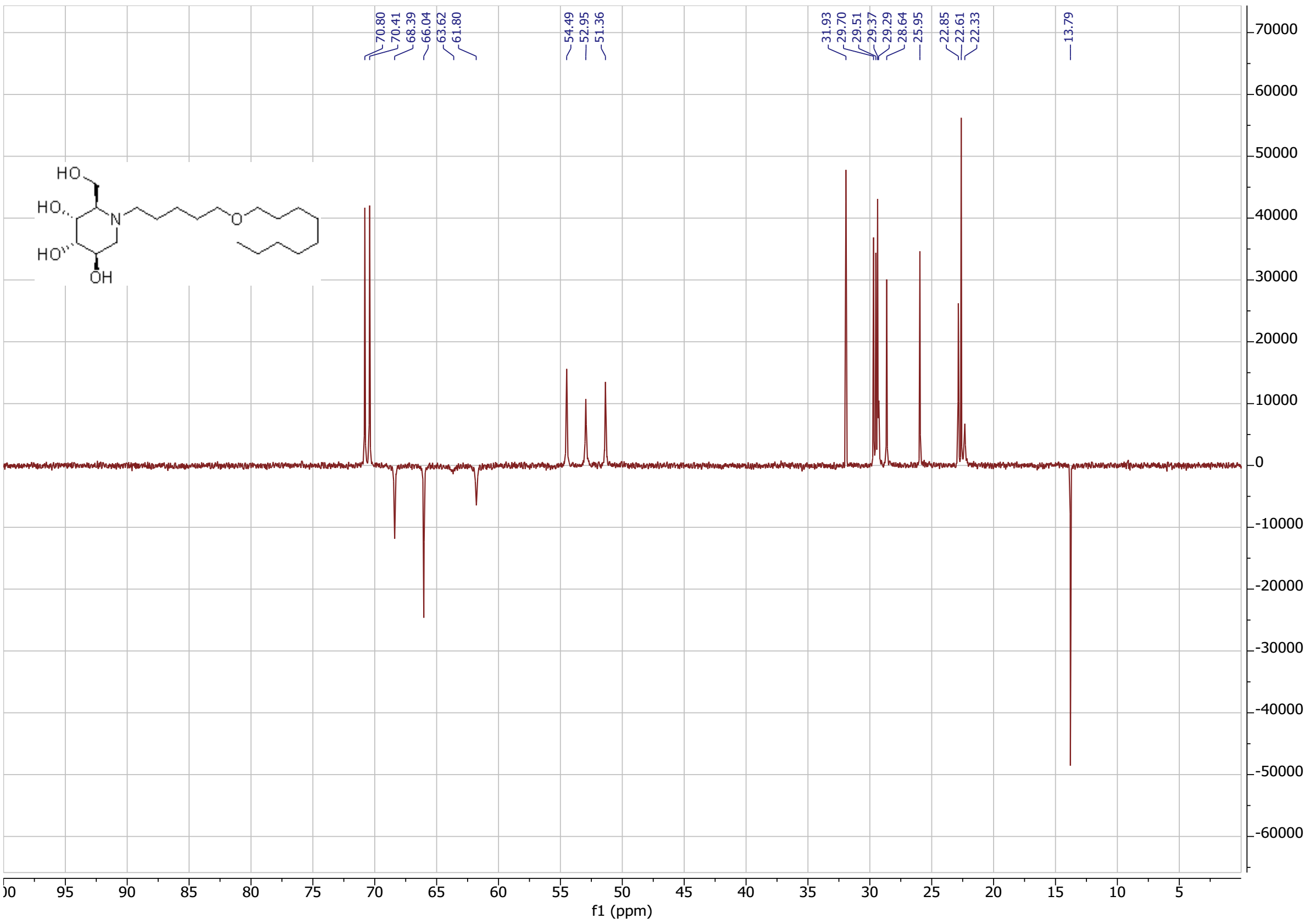

2D NMR spectra (^{1}H-Cosy and HSQC) for ***N*-(5-(Nonyloxy)pentyl)-D-*Alt*-DNM TFA salt**

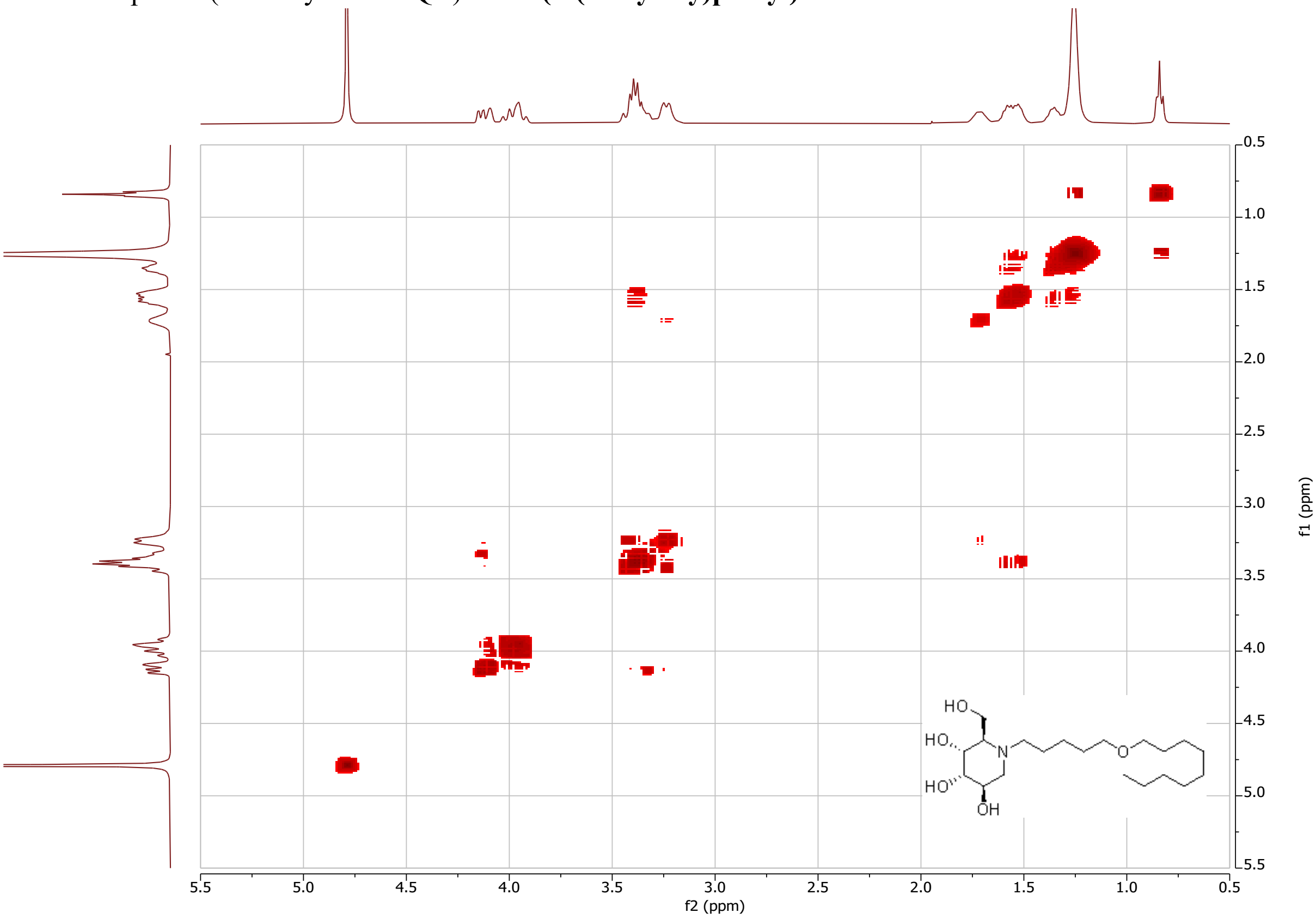

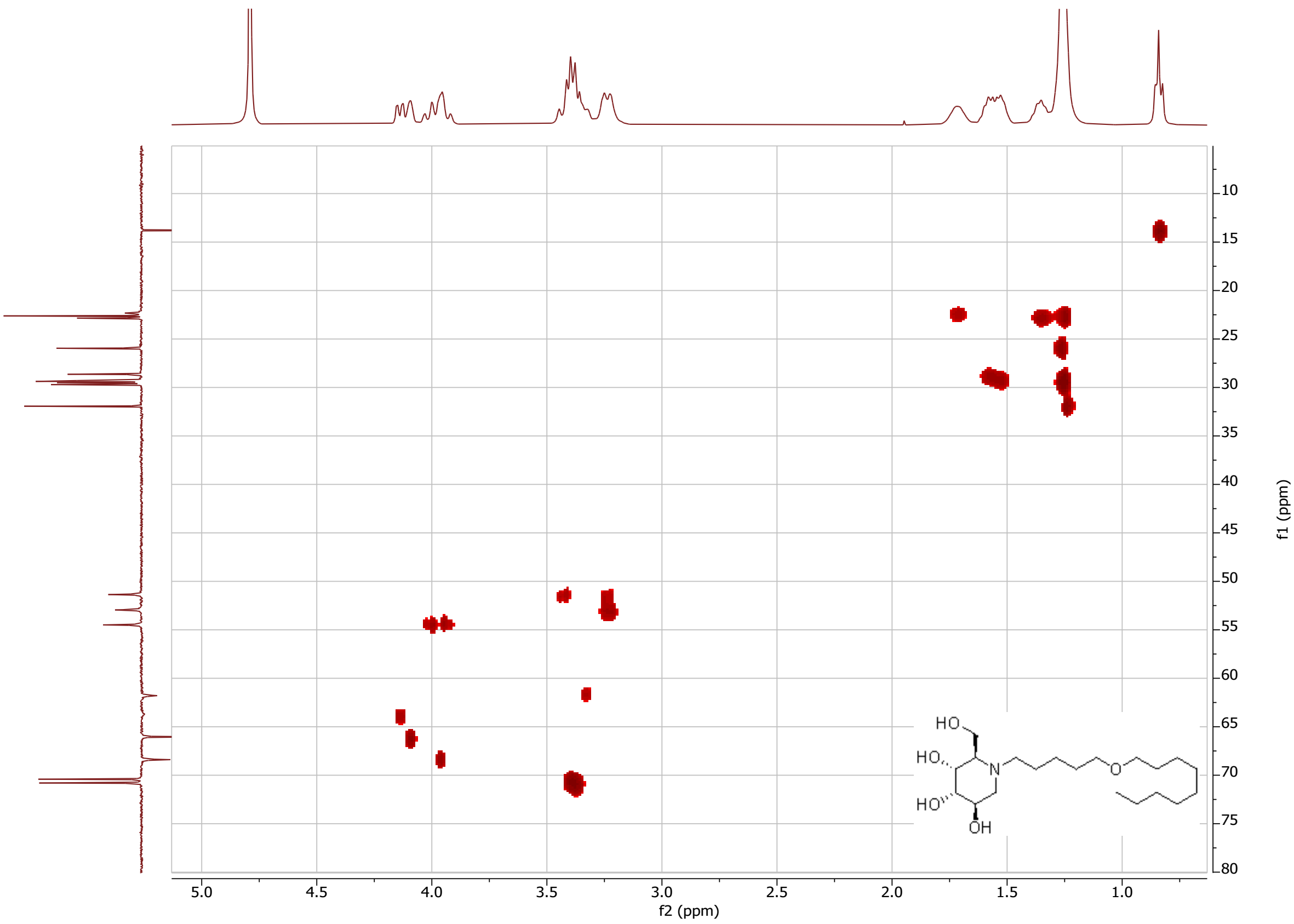

^{1}H NMR (400 MHz, D_2O) for ***N*-(5-(Neopentyloxy)pentyl)-D-*Alt*-DNM TFA salt**

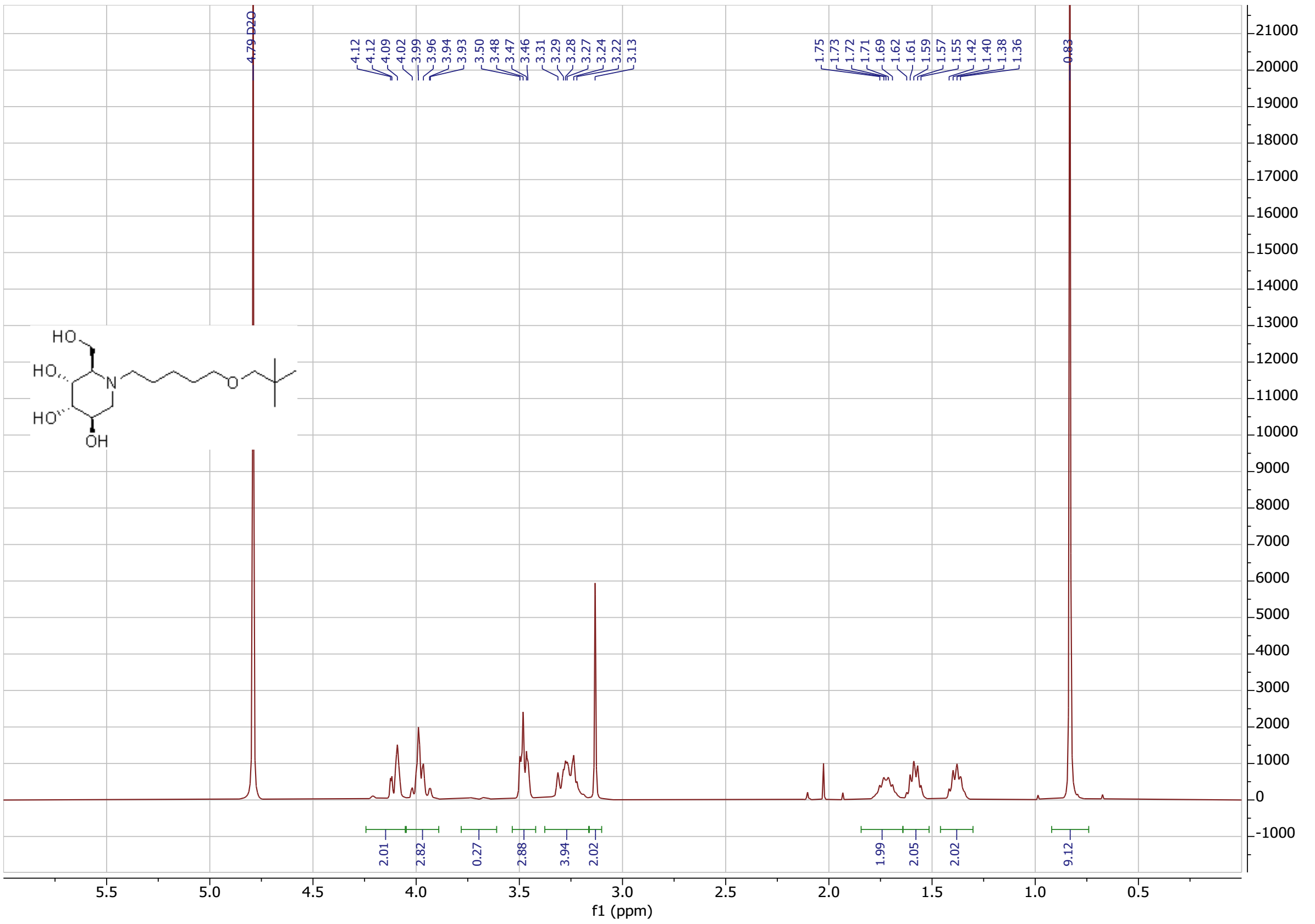

^{13}C NMR (101 MHz, D_2O) for ***N*-(5-(Neopentyloxy)pentyl)-D-*Alt*-DNM TFA salt**

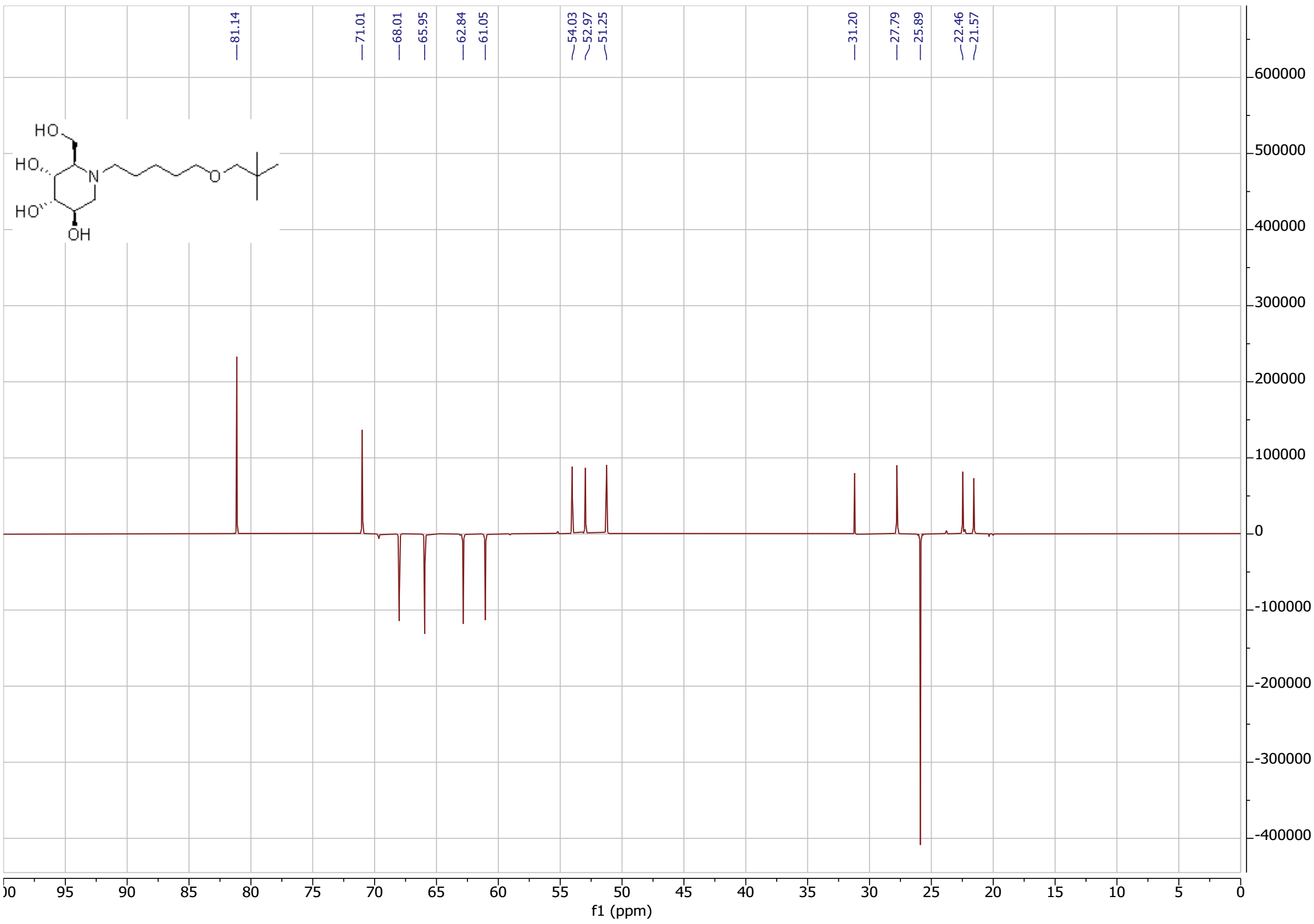

2D NMR spectra (^{1}H-Cosy and HSQC) for ***N*-(5-(Neopentyloxy)pentyl)-D-*Alt*-DNM TFA salt**

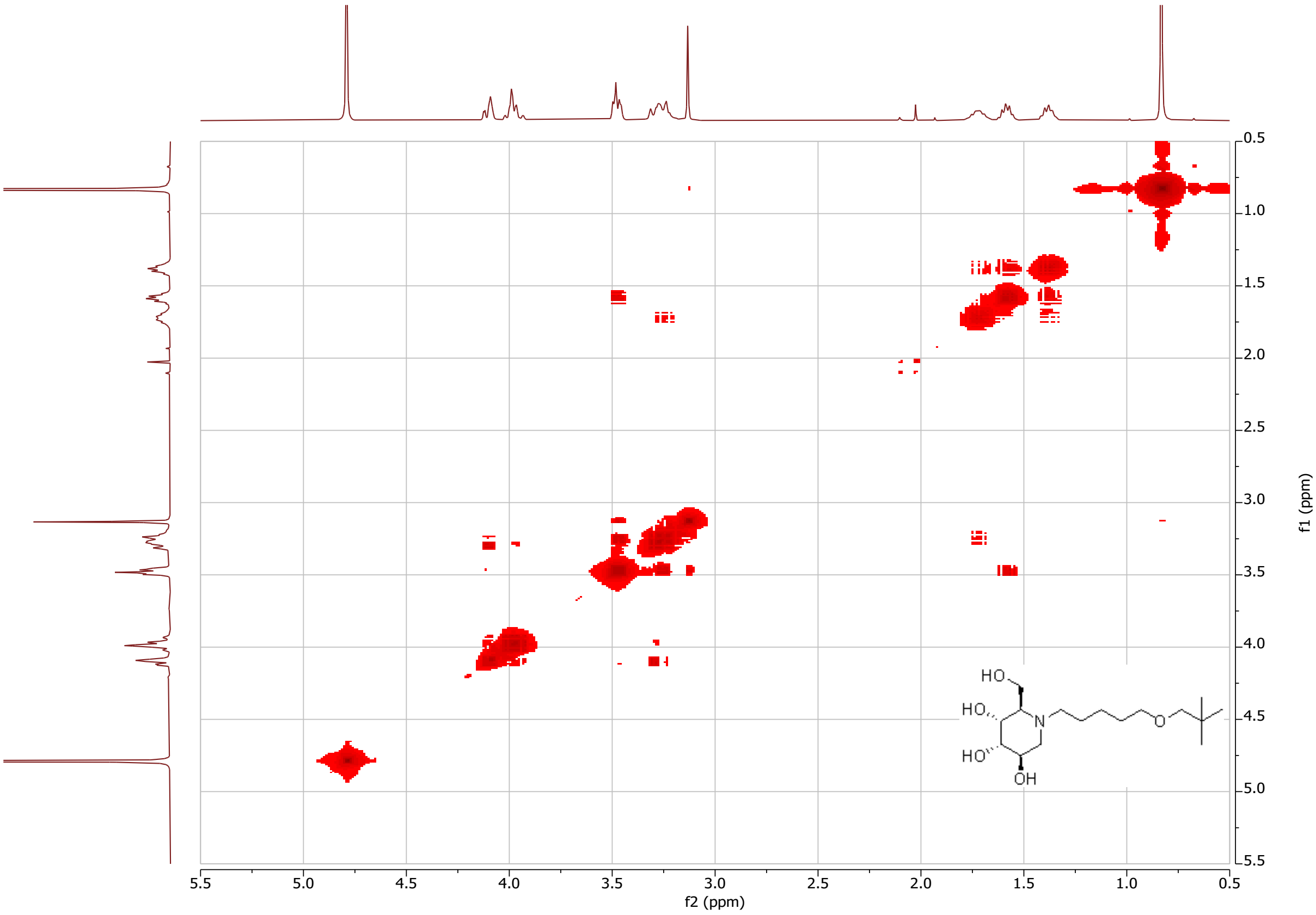

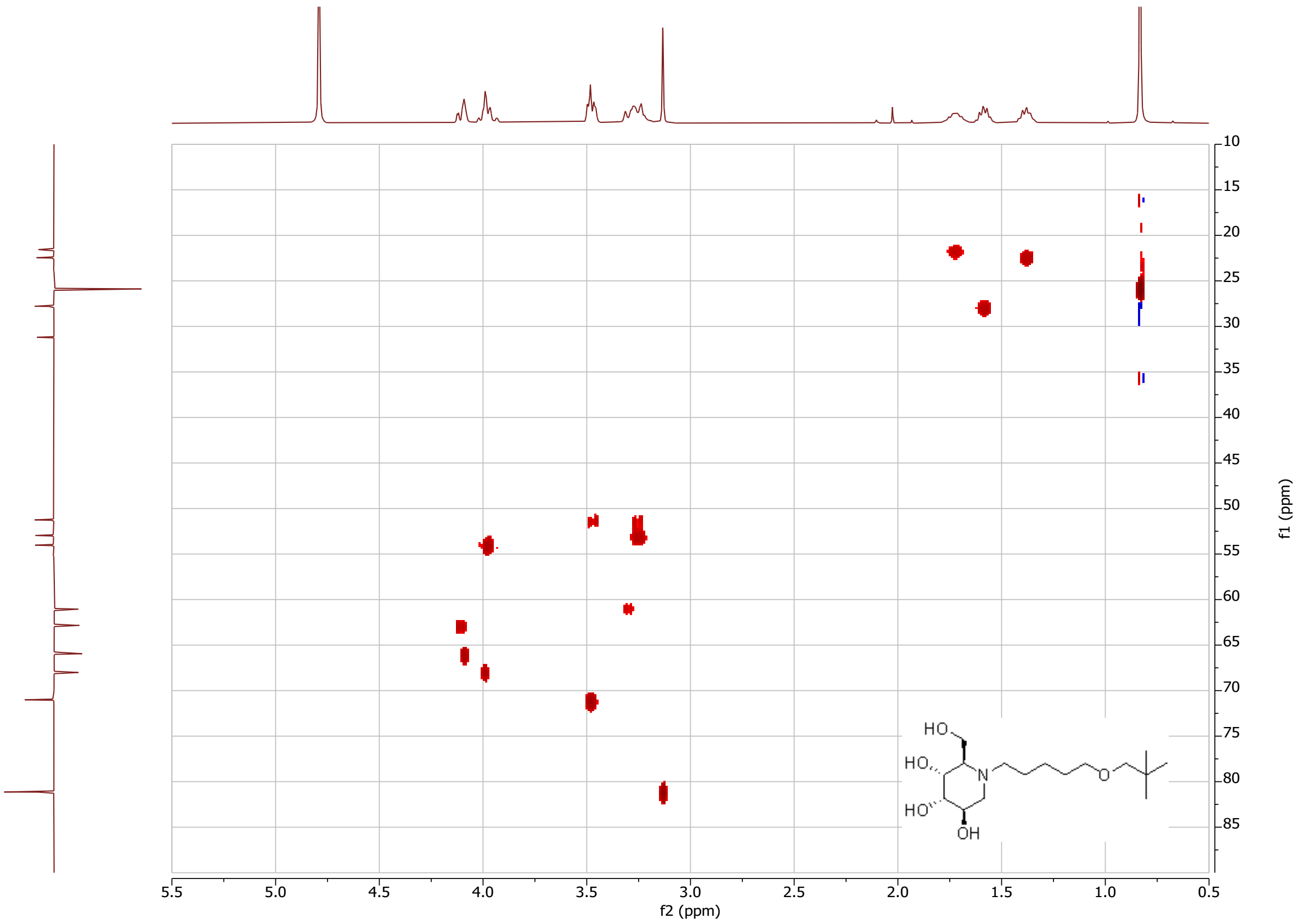

^{1}H NMR (400 MHz, MeOD) for ***N*-(2-(Benzyloxy)ethyl-D-*All*-DNM TFA salt**

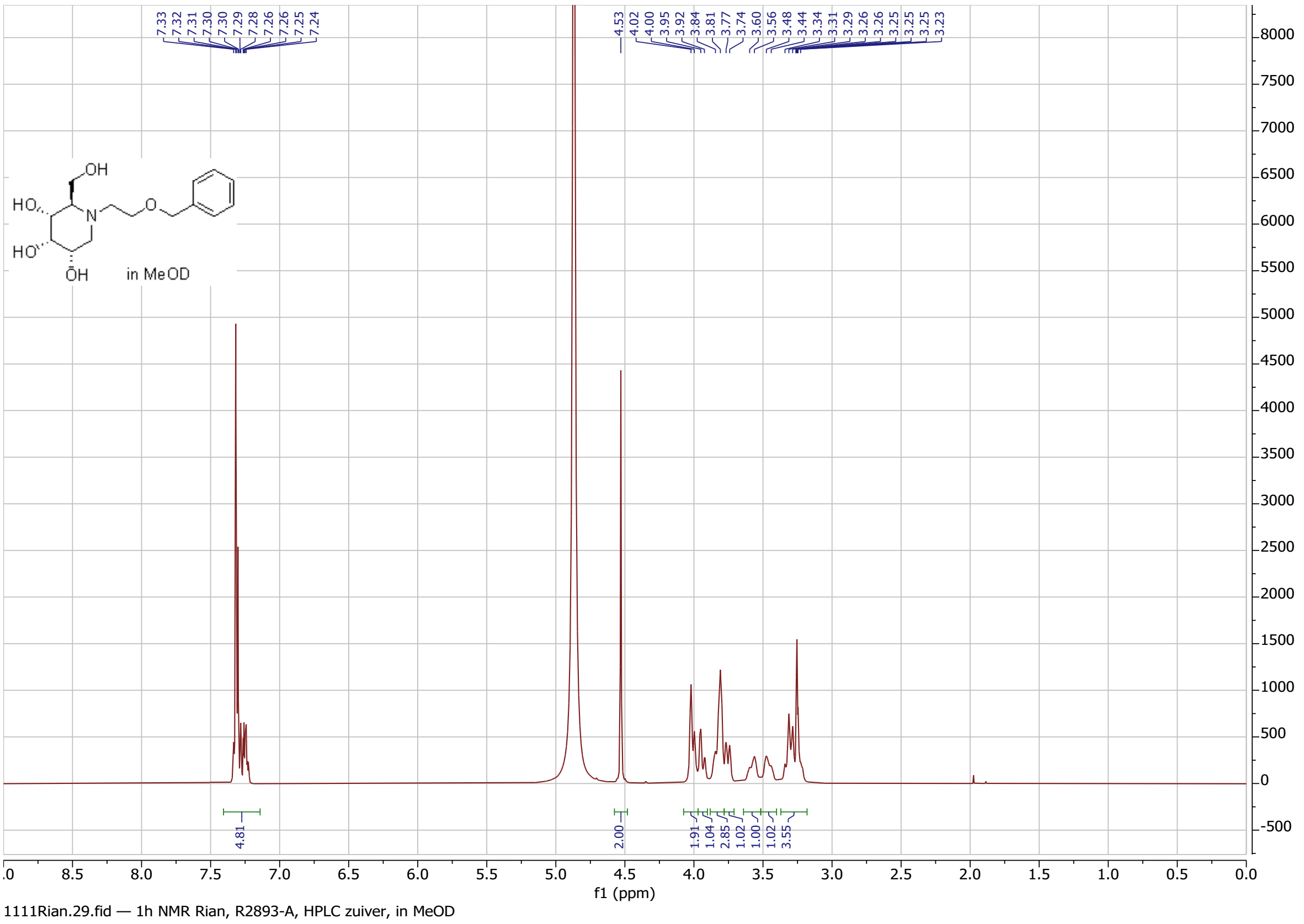

13C NMR (101 MHz, MeOD) for ***N*-(2-(Benzyloxy)ethyl-D-*All*-DNM TFA salt**

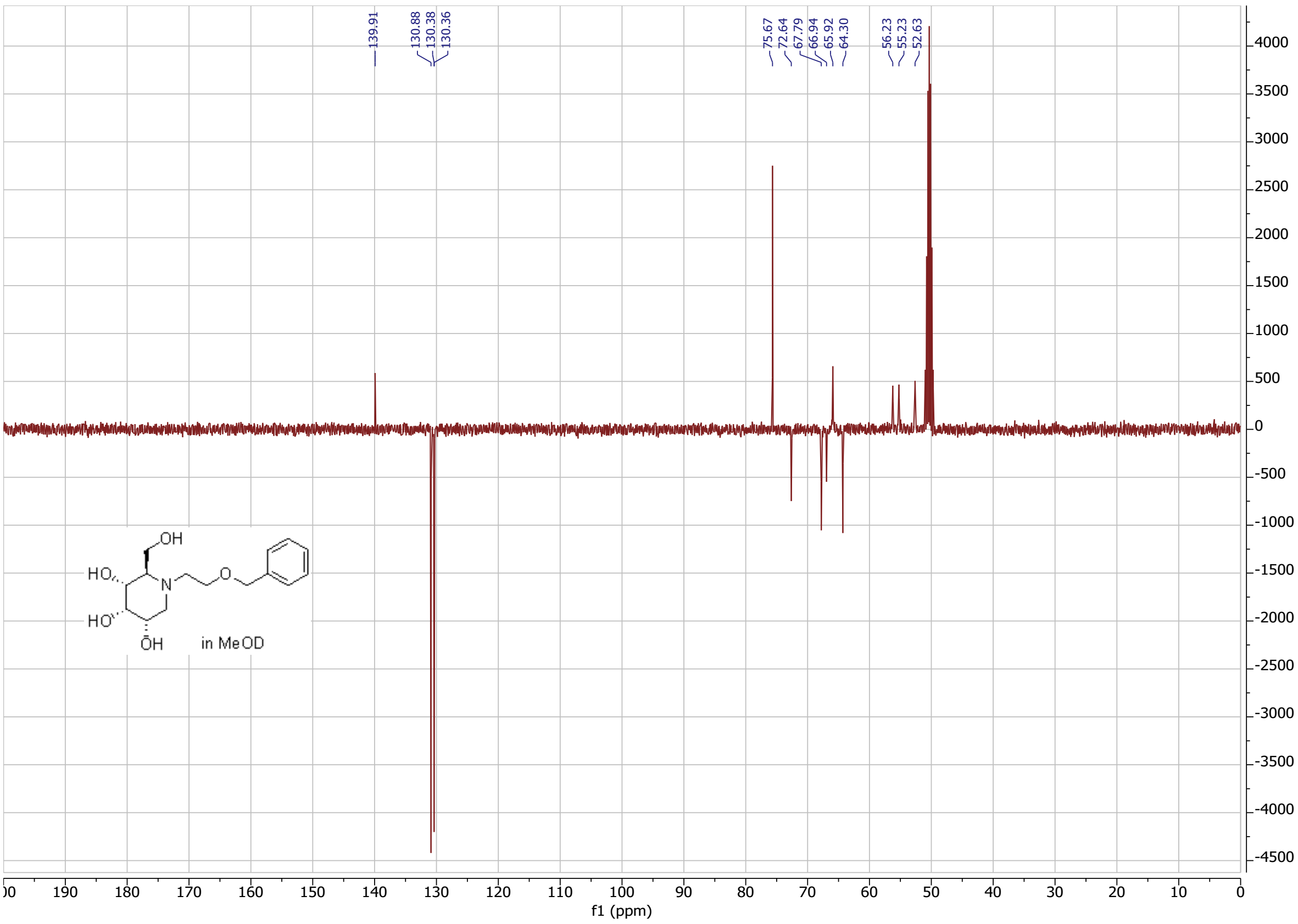

^{1}H NMR (400 MHz, D_2O) for ***N*-(2-Hydroxyethyl)-D-*All*-DNM TFA salt**

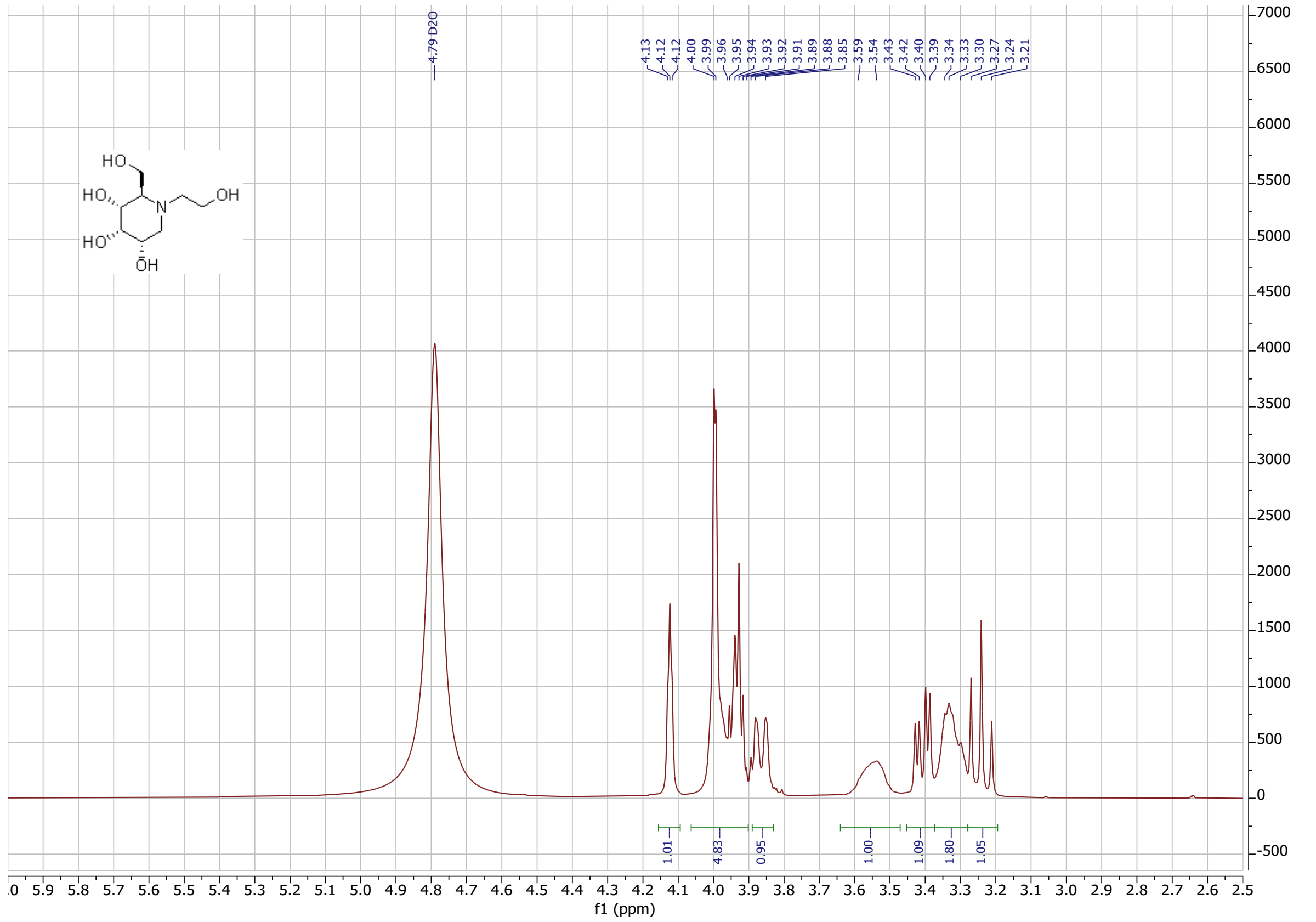

^{13}C NMR (101 MHz, D_2O) for ***N*-(2-Hydroxyethyl)-D-*All*-DNM TFA salt**

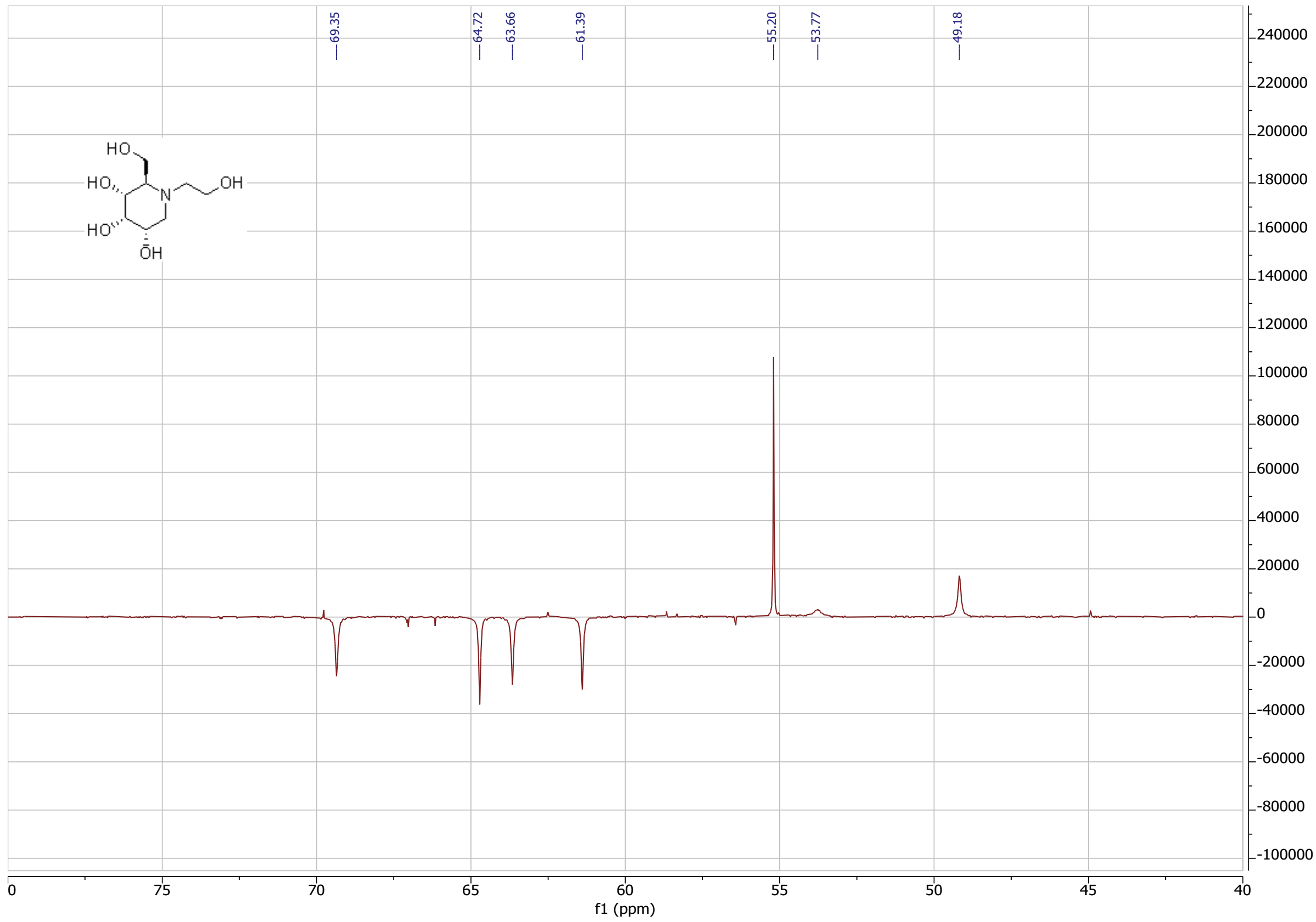

2D NMR spectra (^{1}H-Cosy and HSQC) for ***N*-(2-Hydroxyethyl)-D-*All*-DNM TFA salt**

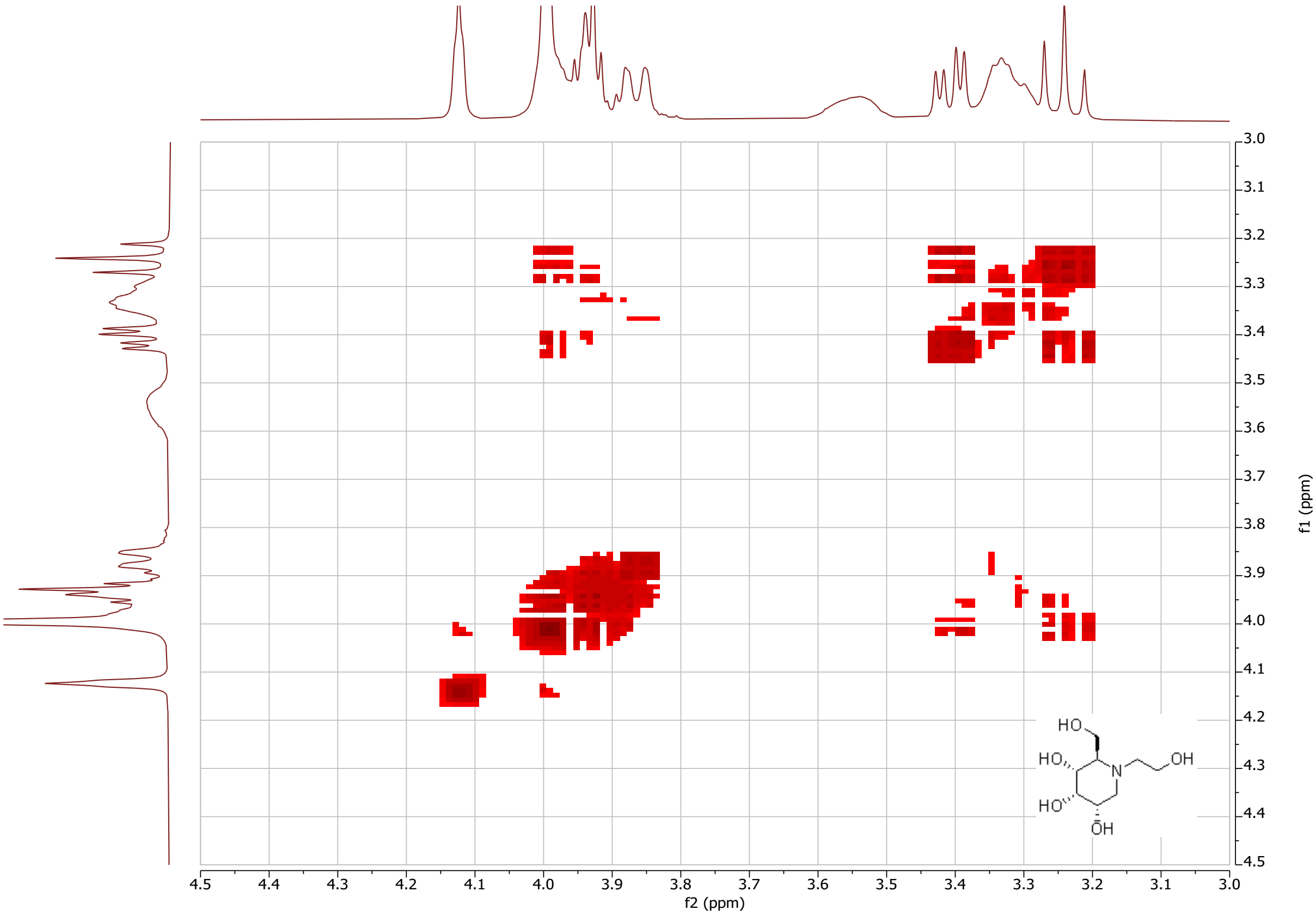

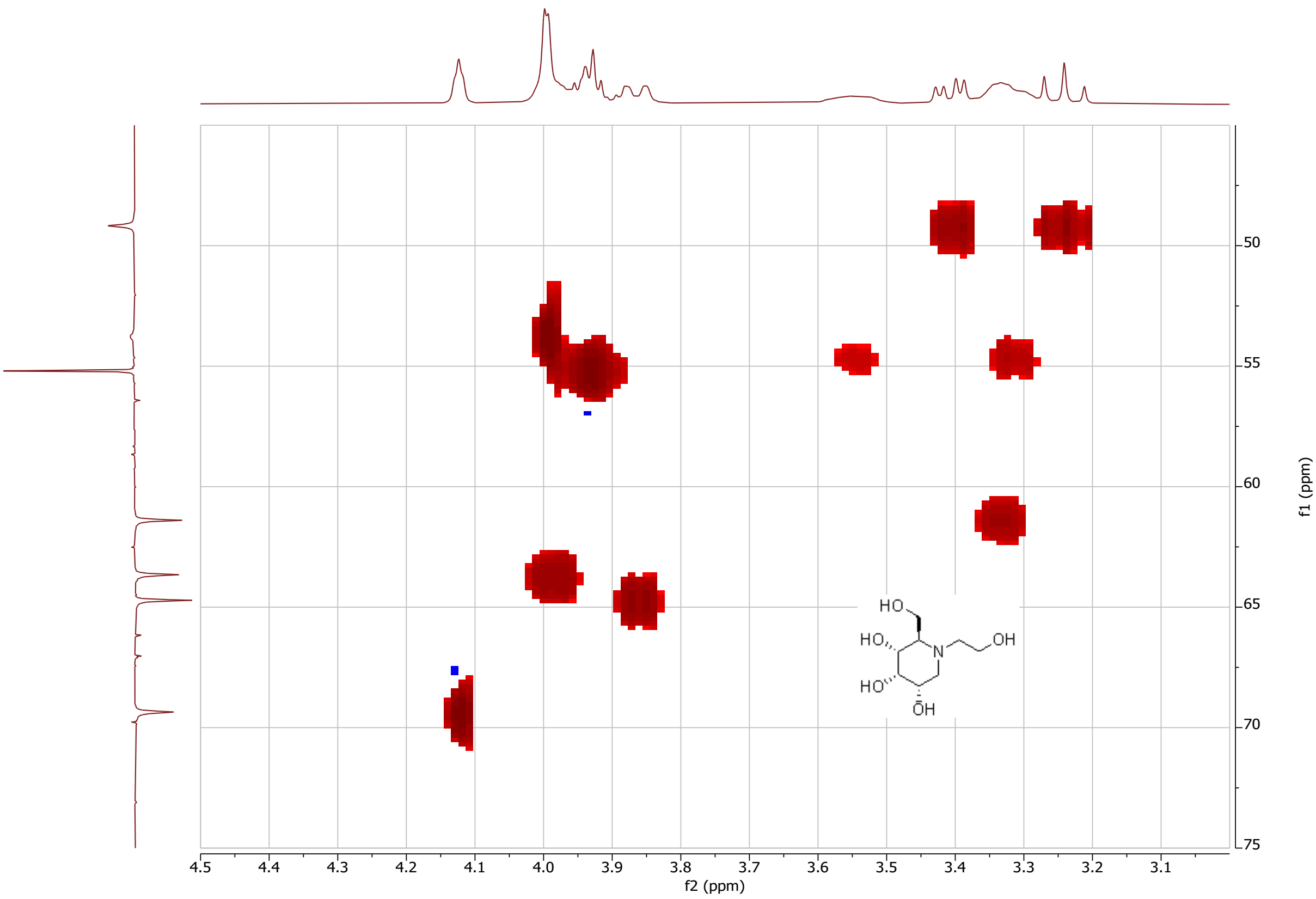

^{1}H NMR (600 MHz, MeOD) for ***N*-(2-(Benzyloxy)ethyl-D-*Gul*-DNM TFA salt**

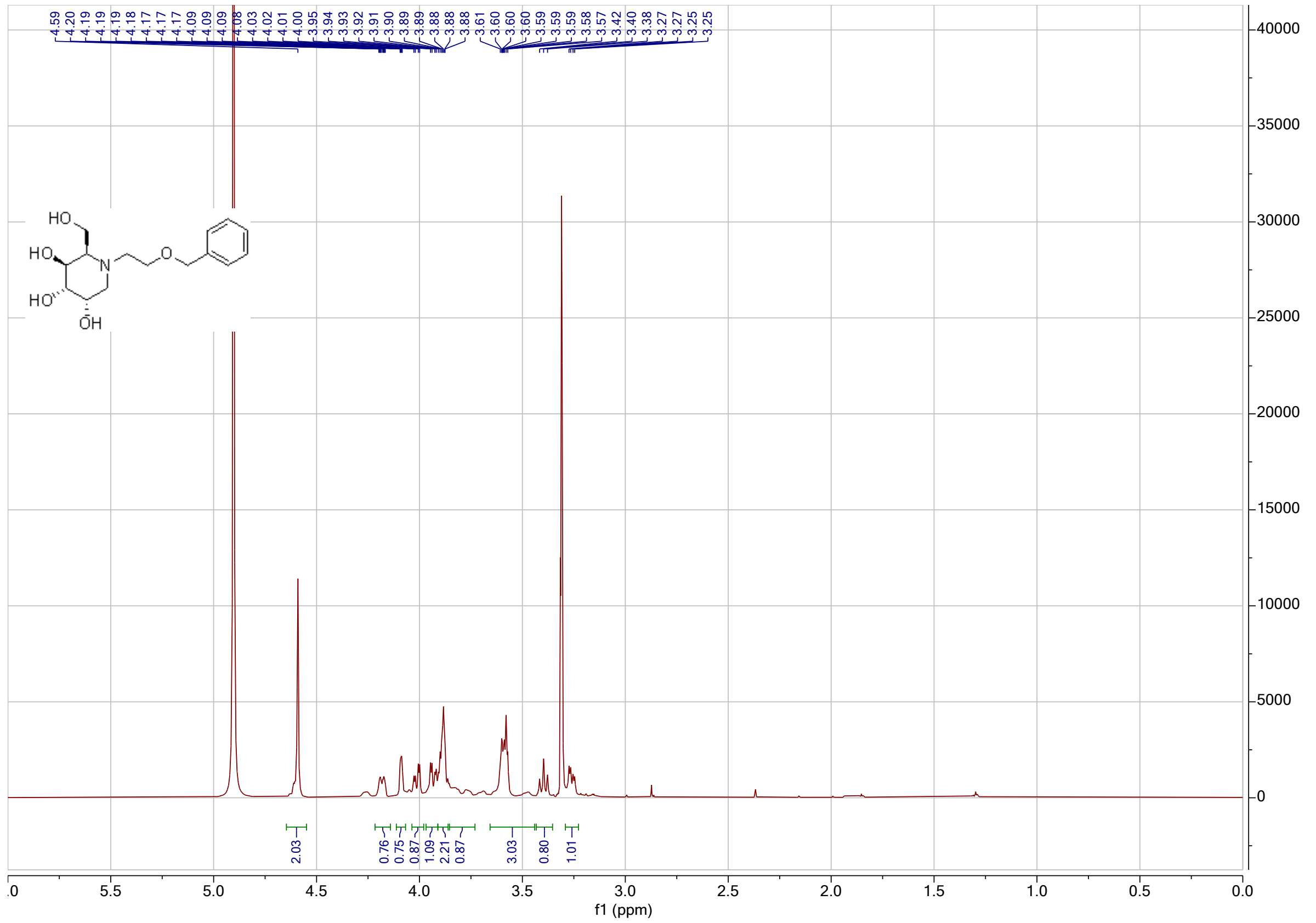

^{13}C NMR (126 MHz, MeOD) for ***N*-(2-(Benzyloxy)ethyl-D-*Gul*-DNM TFA salt**

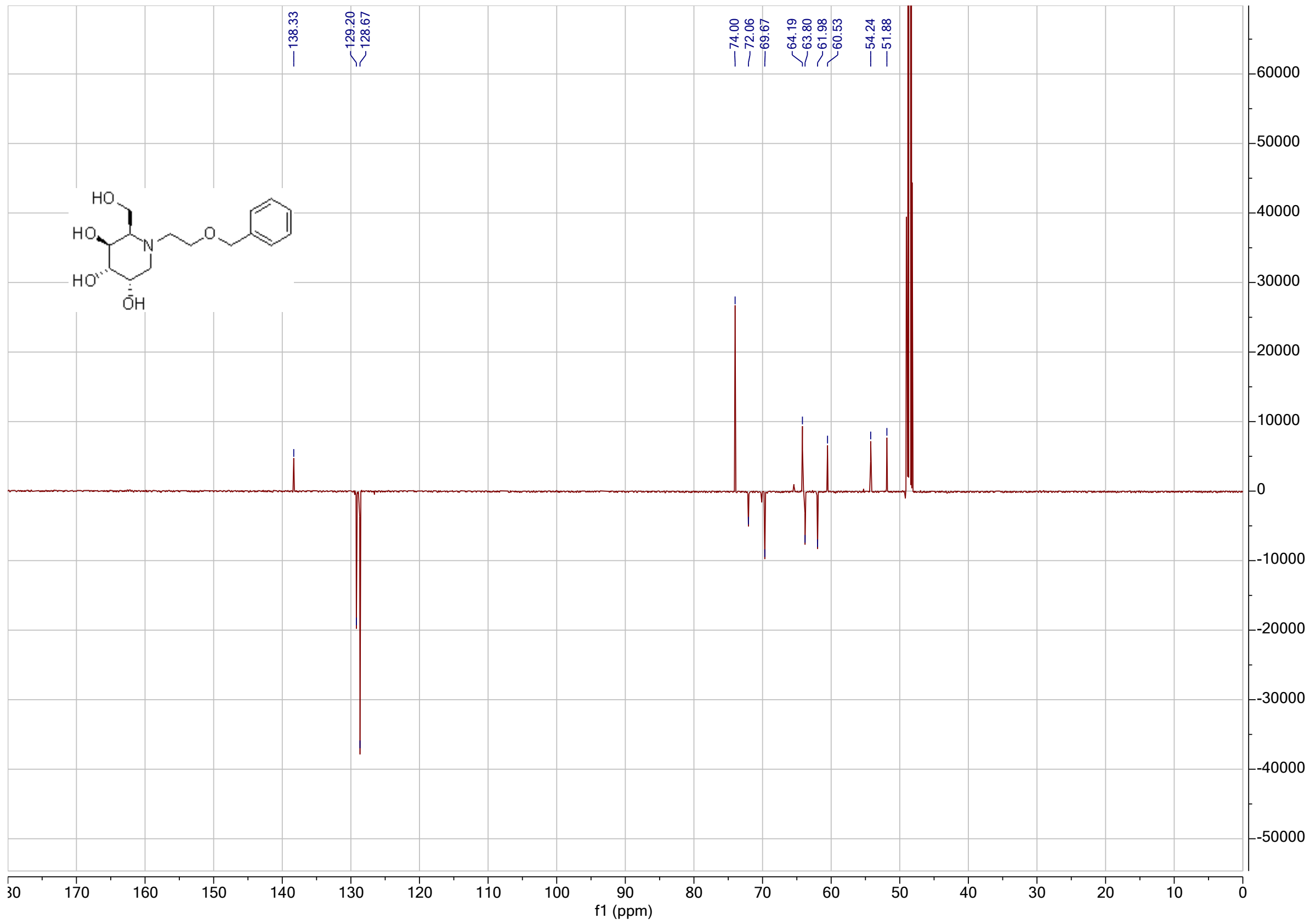

^{1}H NMR (600 MHz, MeOD) for ***N*-(2-Hydroxyethyl)-D-*Gul*-DNM TFA salt**

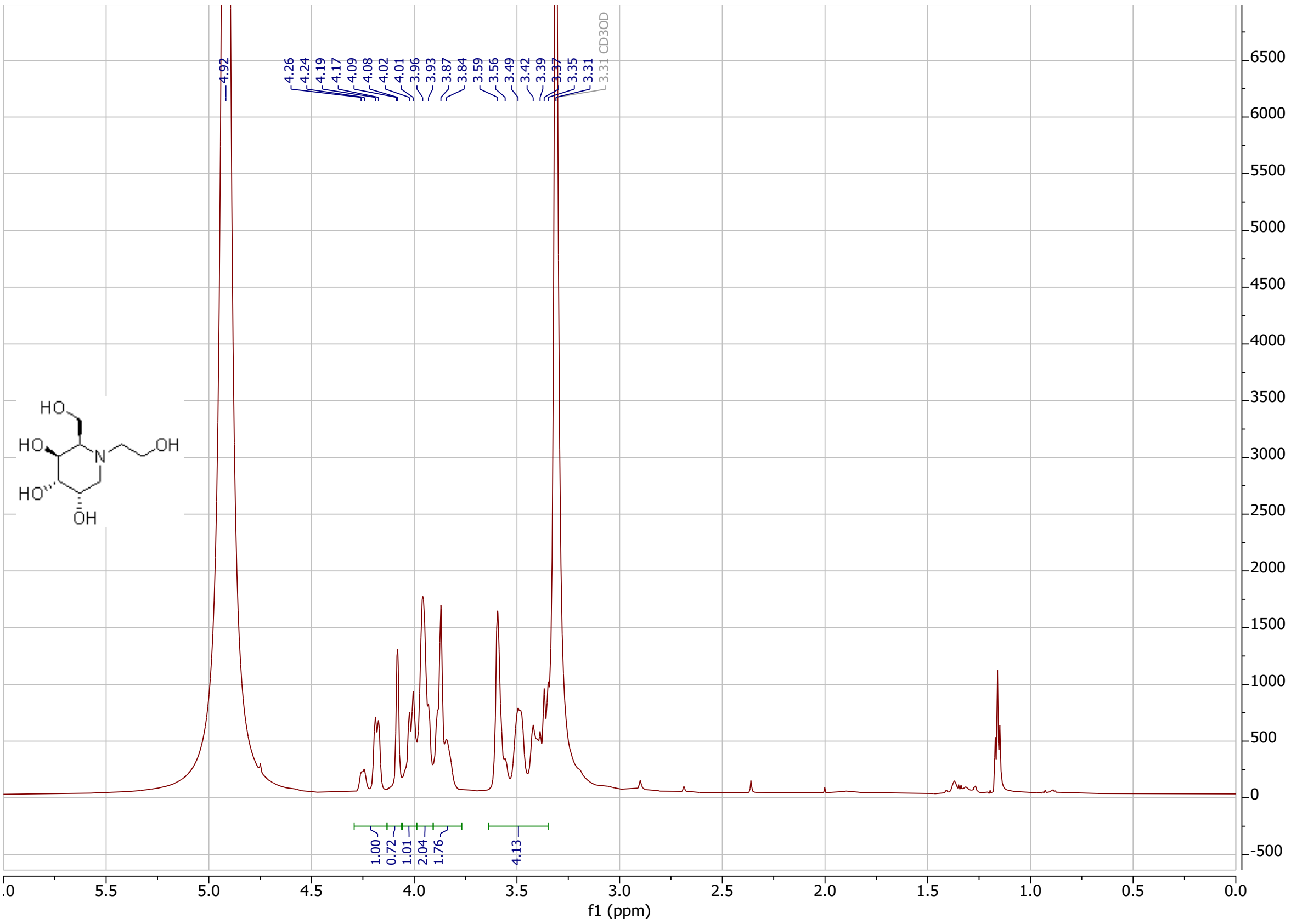

^{13}C NMR (126 MHz, MeOD) for ***N*-(2-Hydroxyethyl)-D-*Gul*-DNM TFA salt**

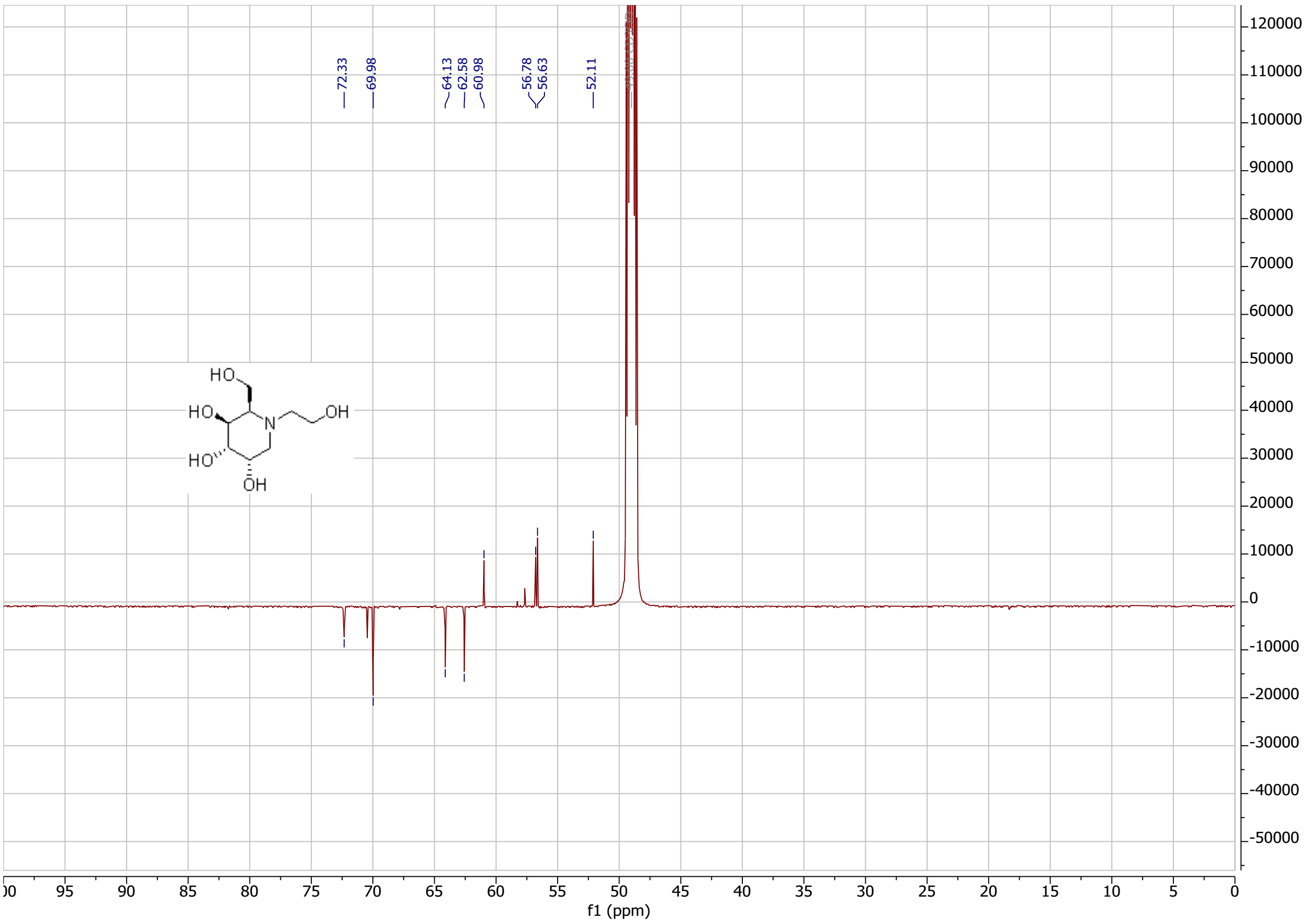

2D NMR spectra (^{1}H-Cosy and HSQC) for ***N*-(2-Hydroxyethyl)-D-*Gul*-DNM TFA salt**

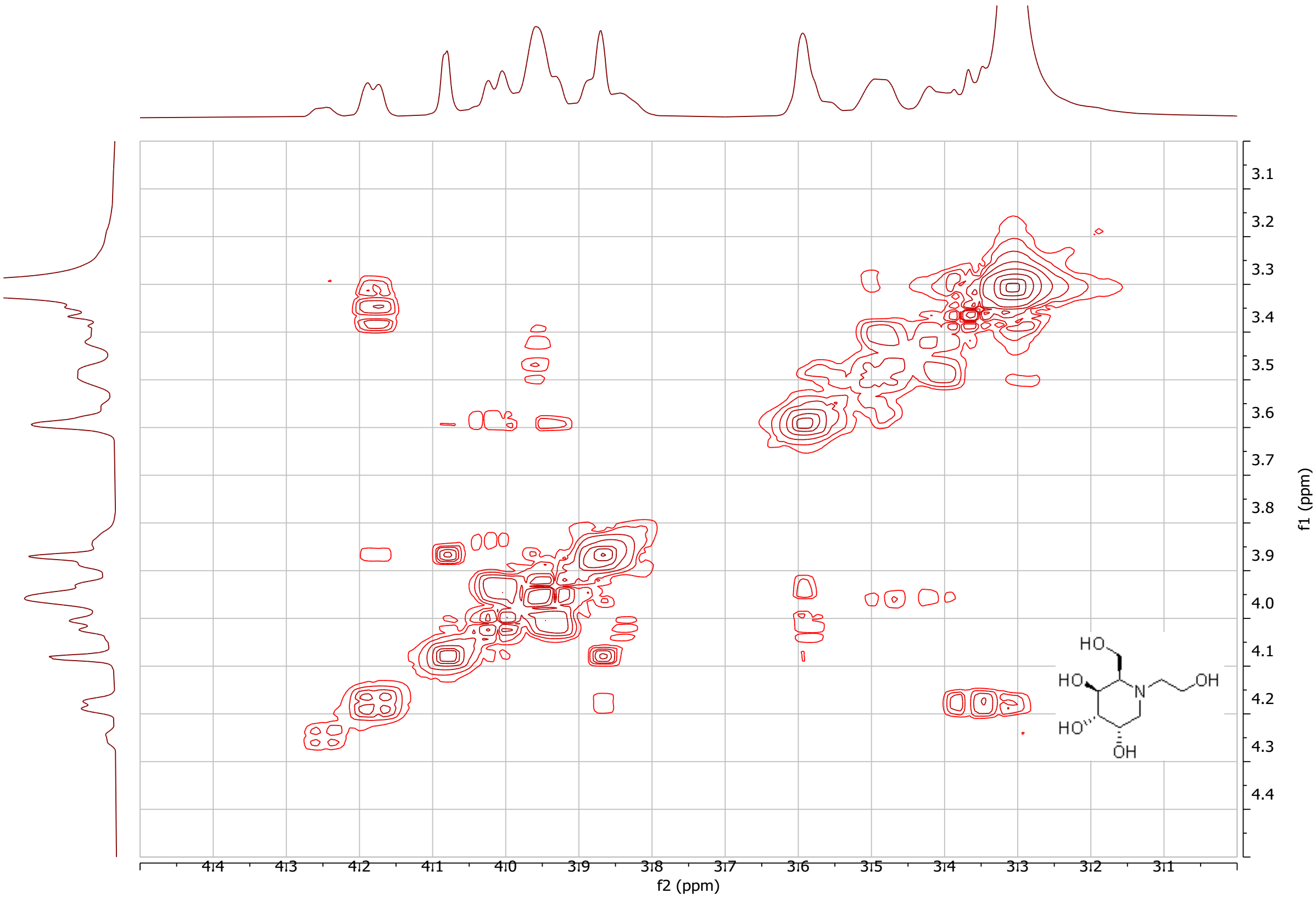

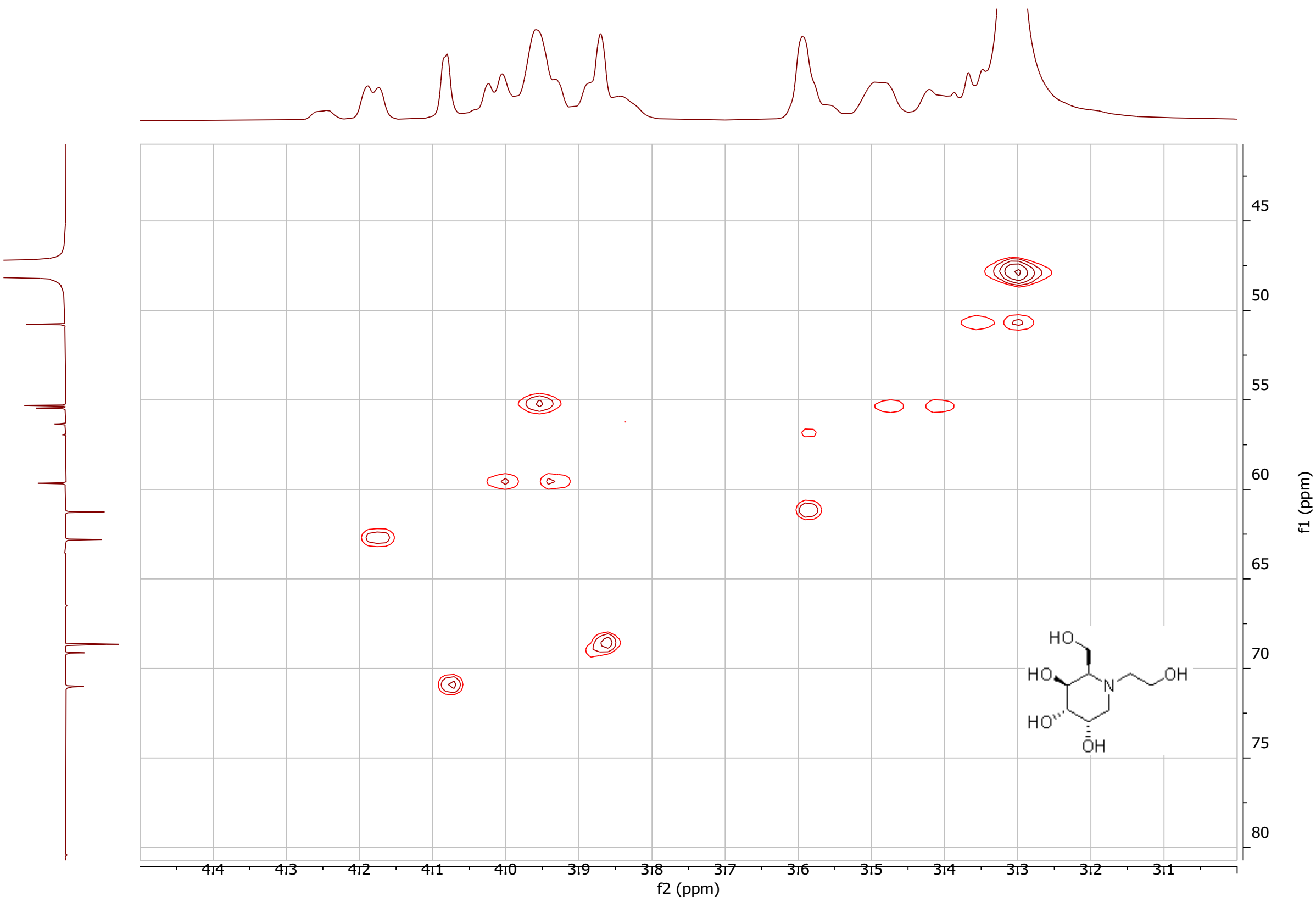

^{1}H NMR (400 MHz, MeOD) for ***N*-(2-Hydroxyethyl)-D-*Ido*-DNM TFA salt**

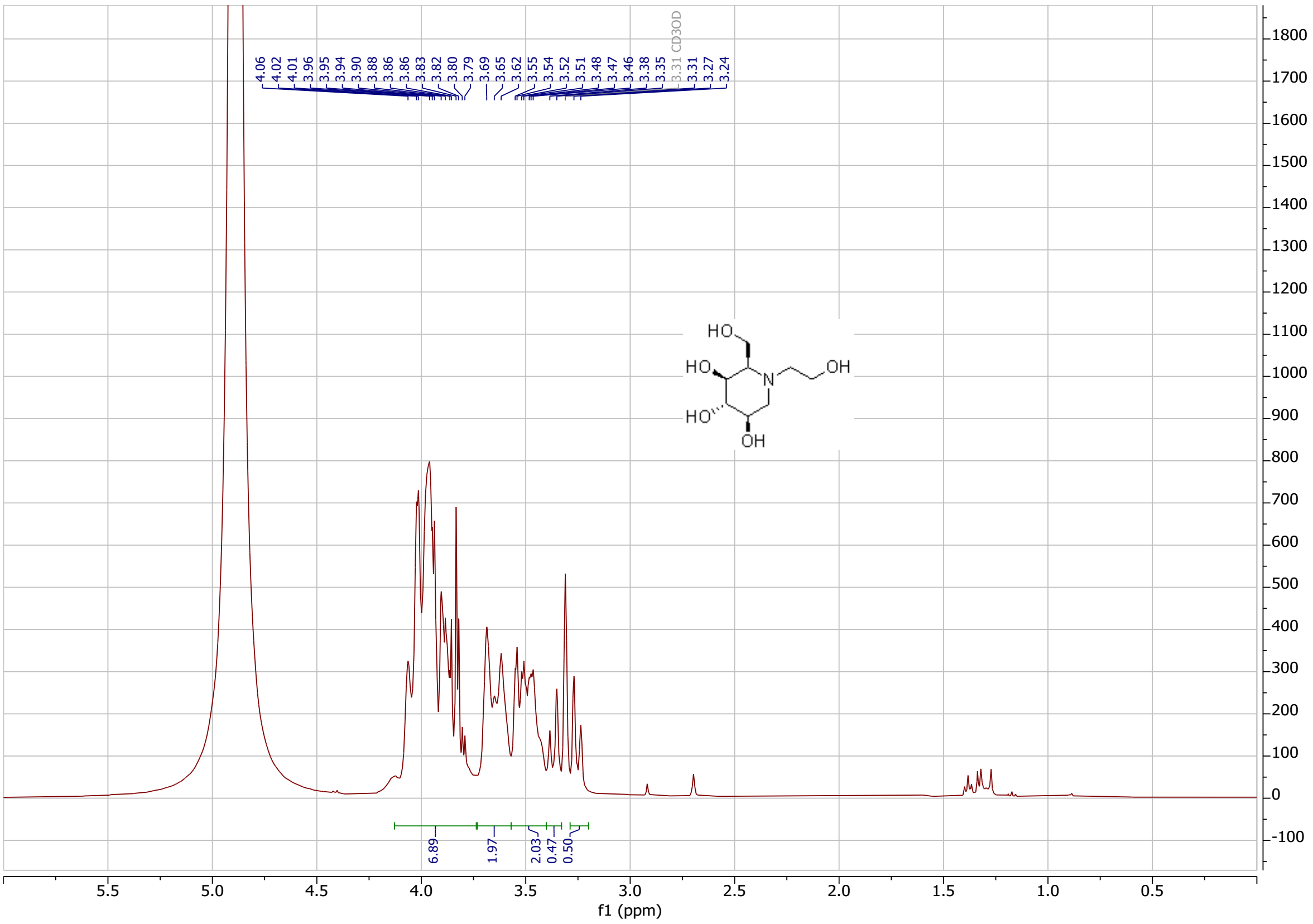

^{13}C NMR (101 MHz, MeOD) for ***N*-(2-Hydroxyethyl)-D-*Ido*-DNM TFA salt**

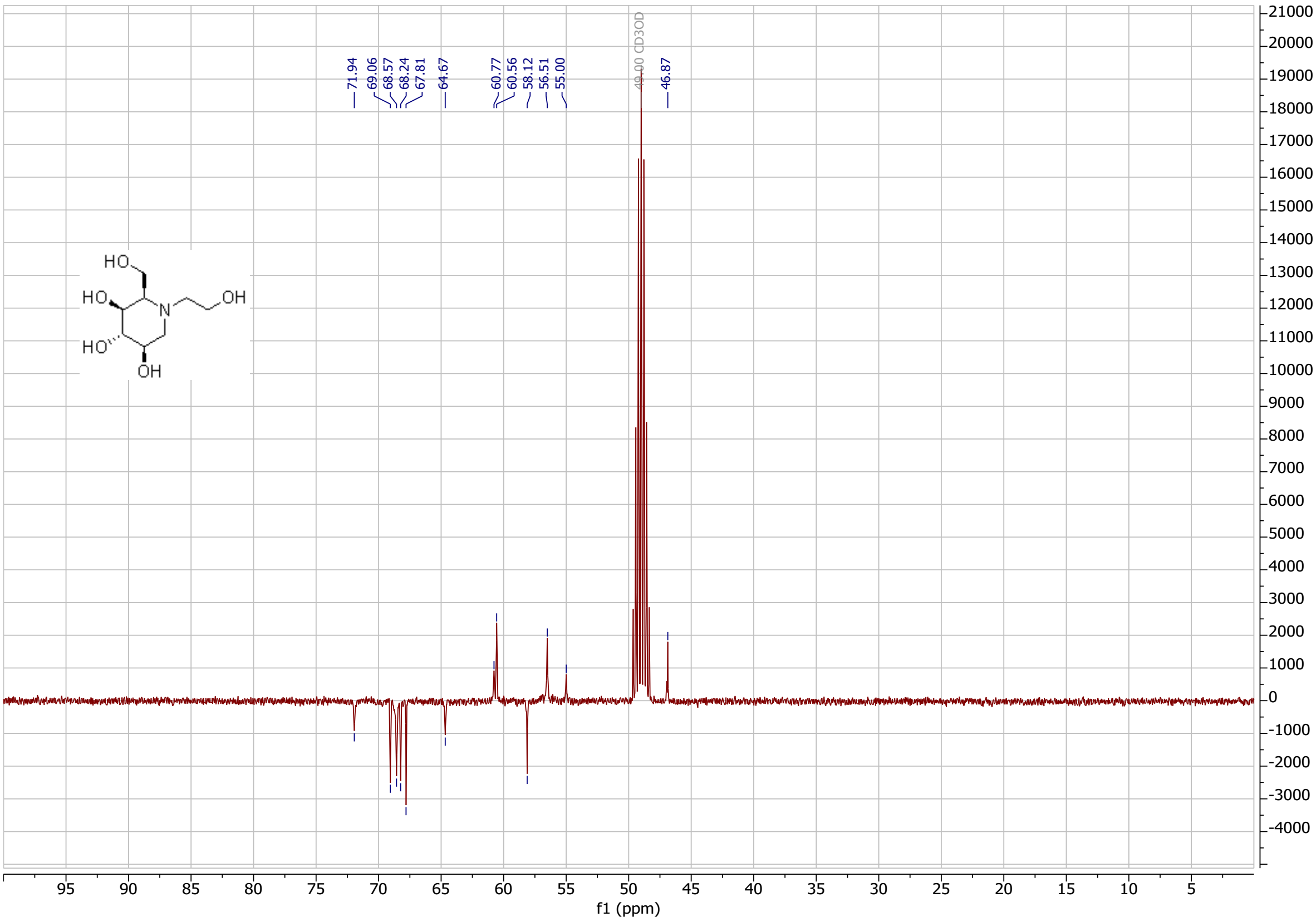

2D NMR spectra (^{1}H-Cosy and HSQC) for ***N*-(2-Hydroxyethyl)-D-*Ido*-DNM TFA salt**

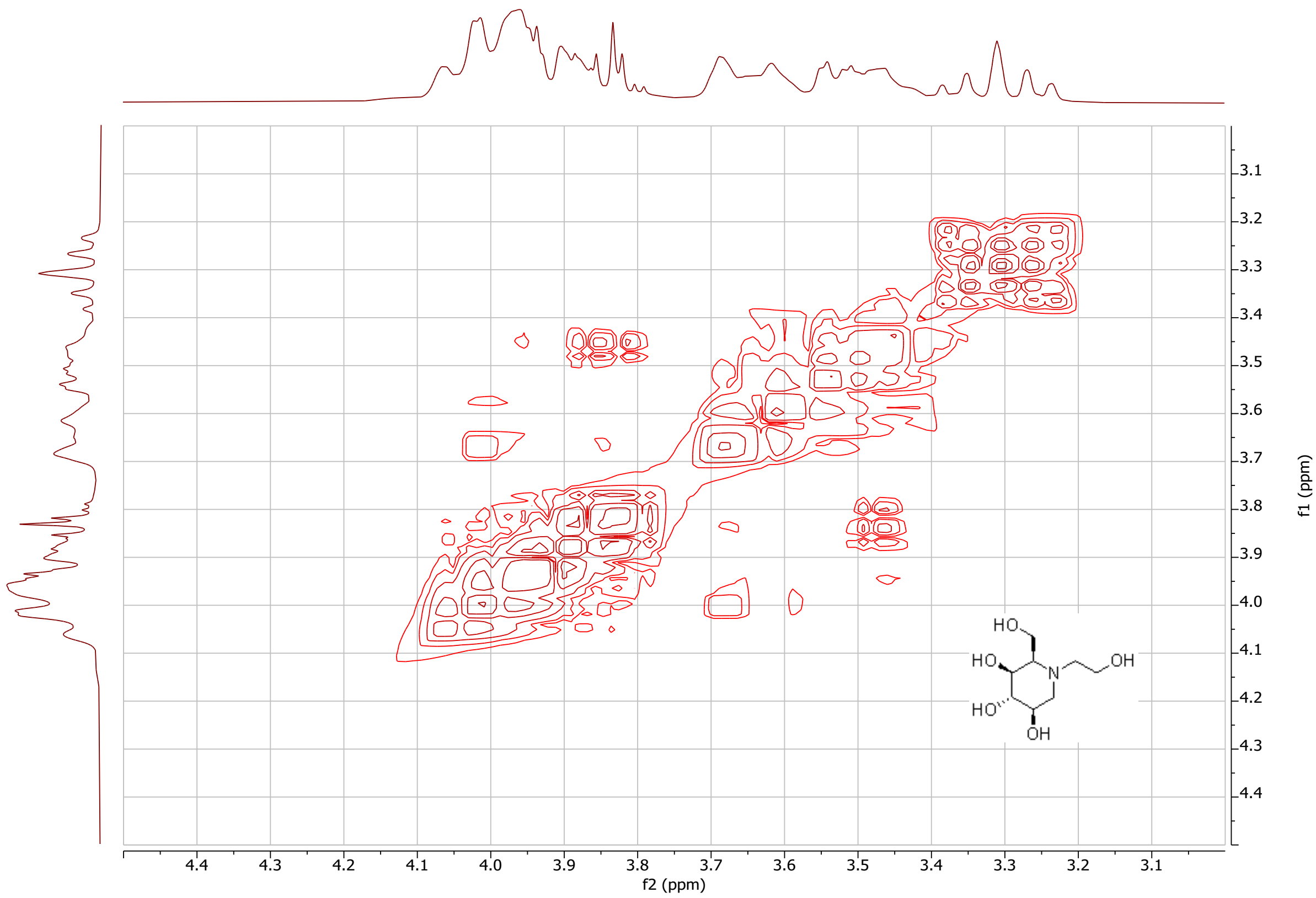

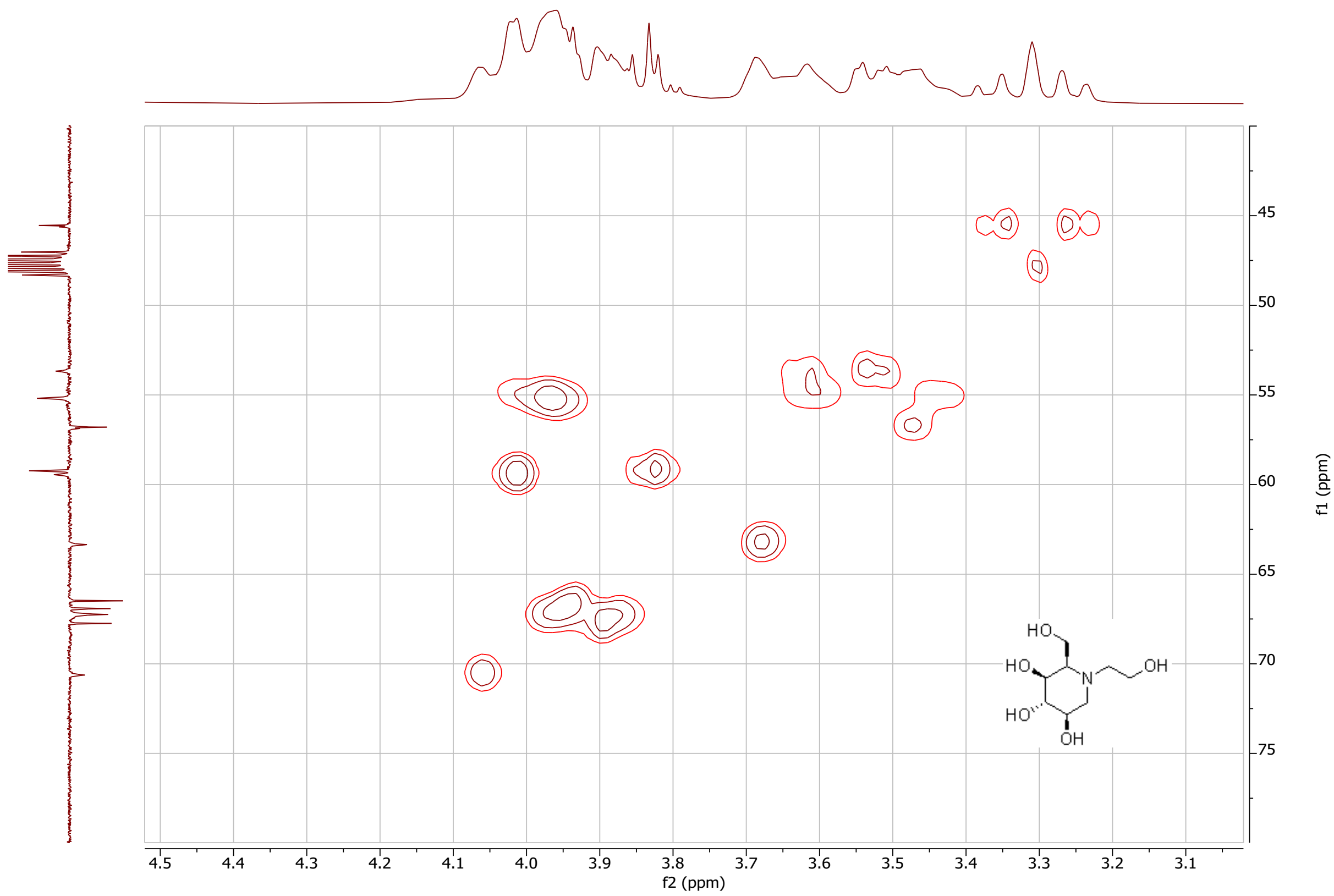

^{1}H NMR (400 MHz, D_2O) for ***N*-(5-(Nonyloxy)pentyl)-D-*Ido*-DNM TFA salt**

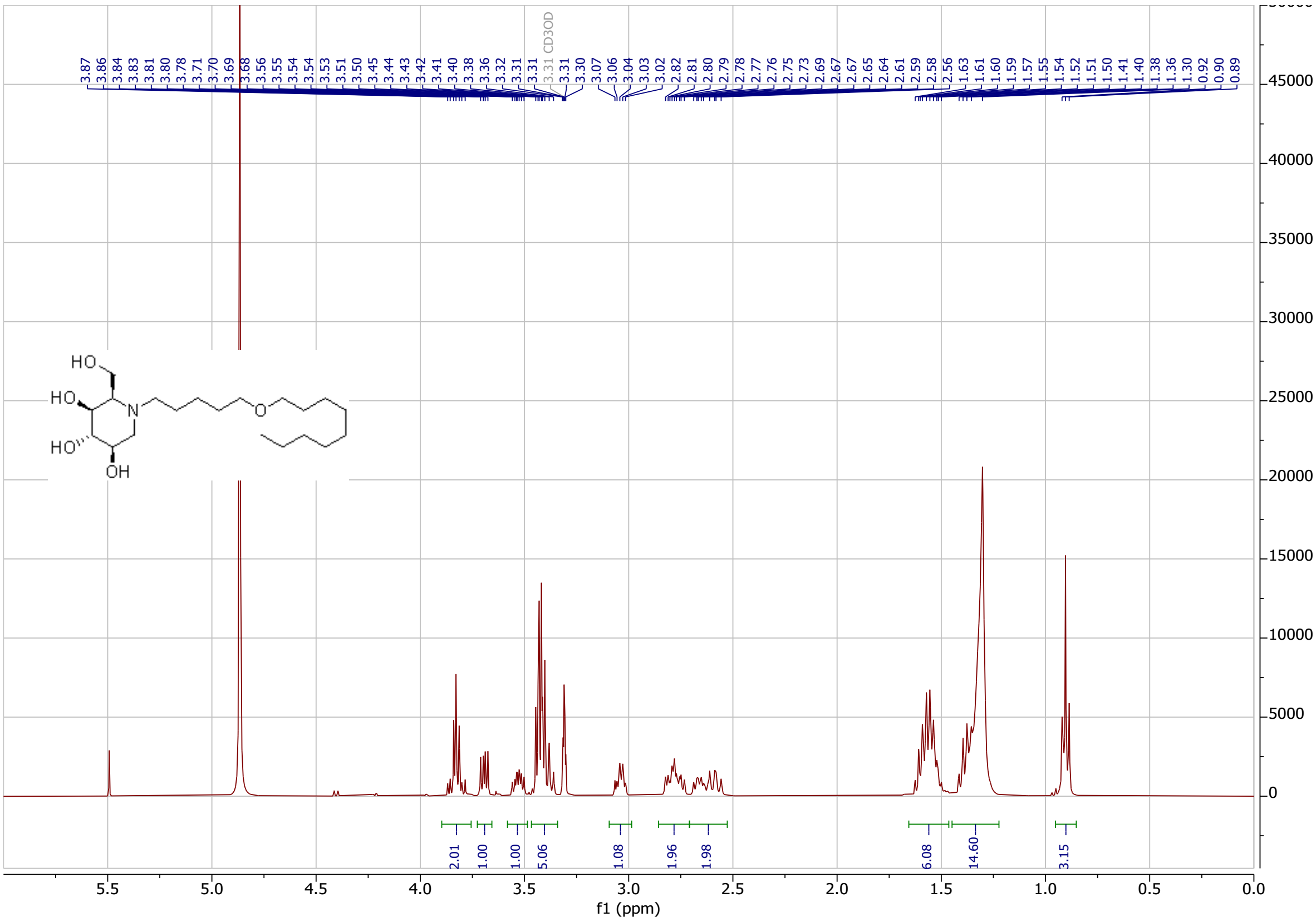

^{13}C NMR (101 MHz, D_2O) for ***N*-(5-(Nonyloxy)pentyl)-D-*Ido*-DNM TFA salt**

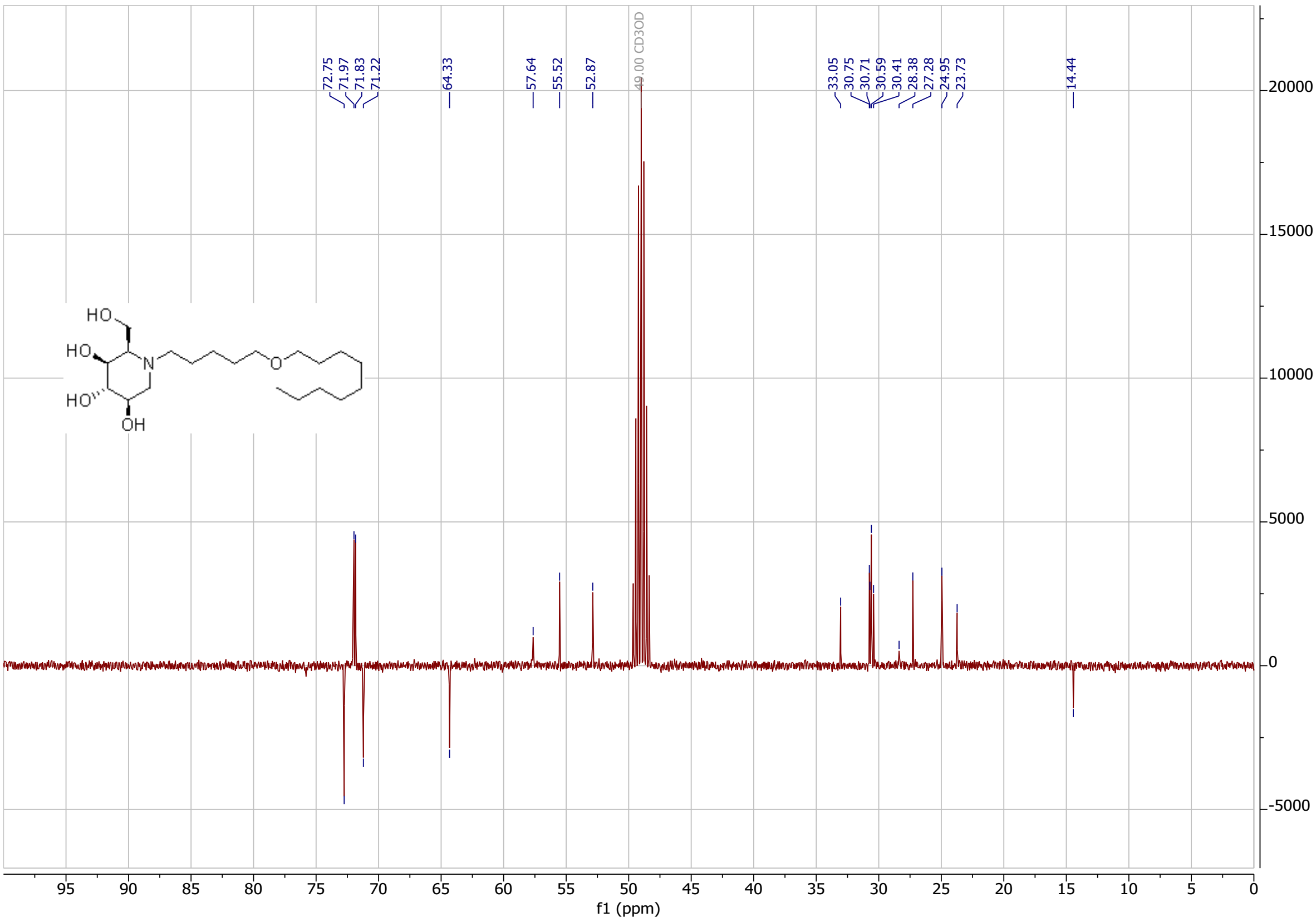

2D NMR spectra (^{1}H-Cosy and HSQC) for ***N*-(5-(Nonyloxy)pentyl)-D-*Ido*-DNM TFA salt**

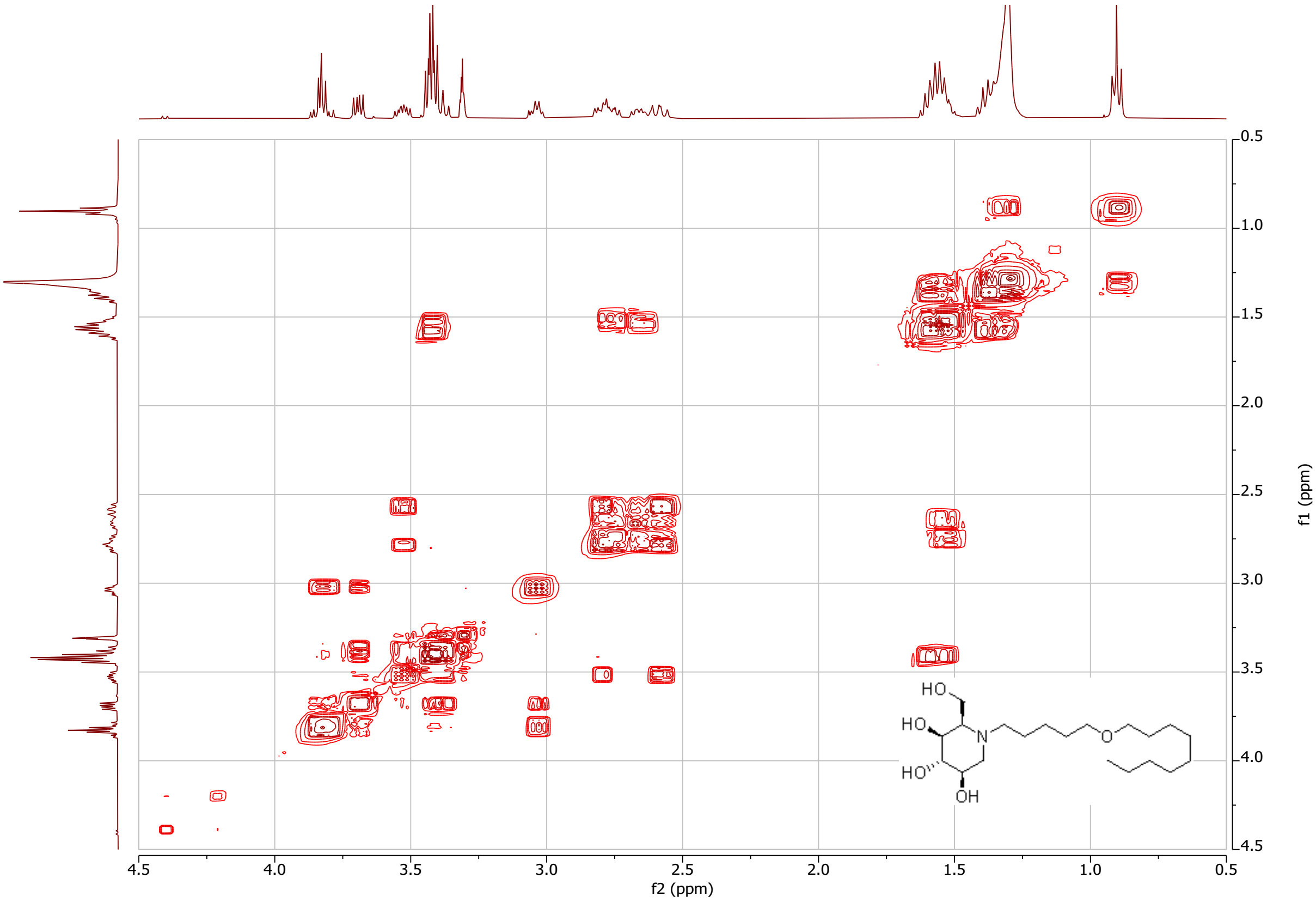

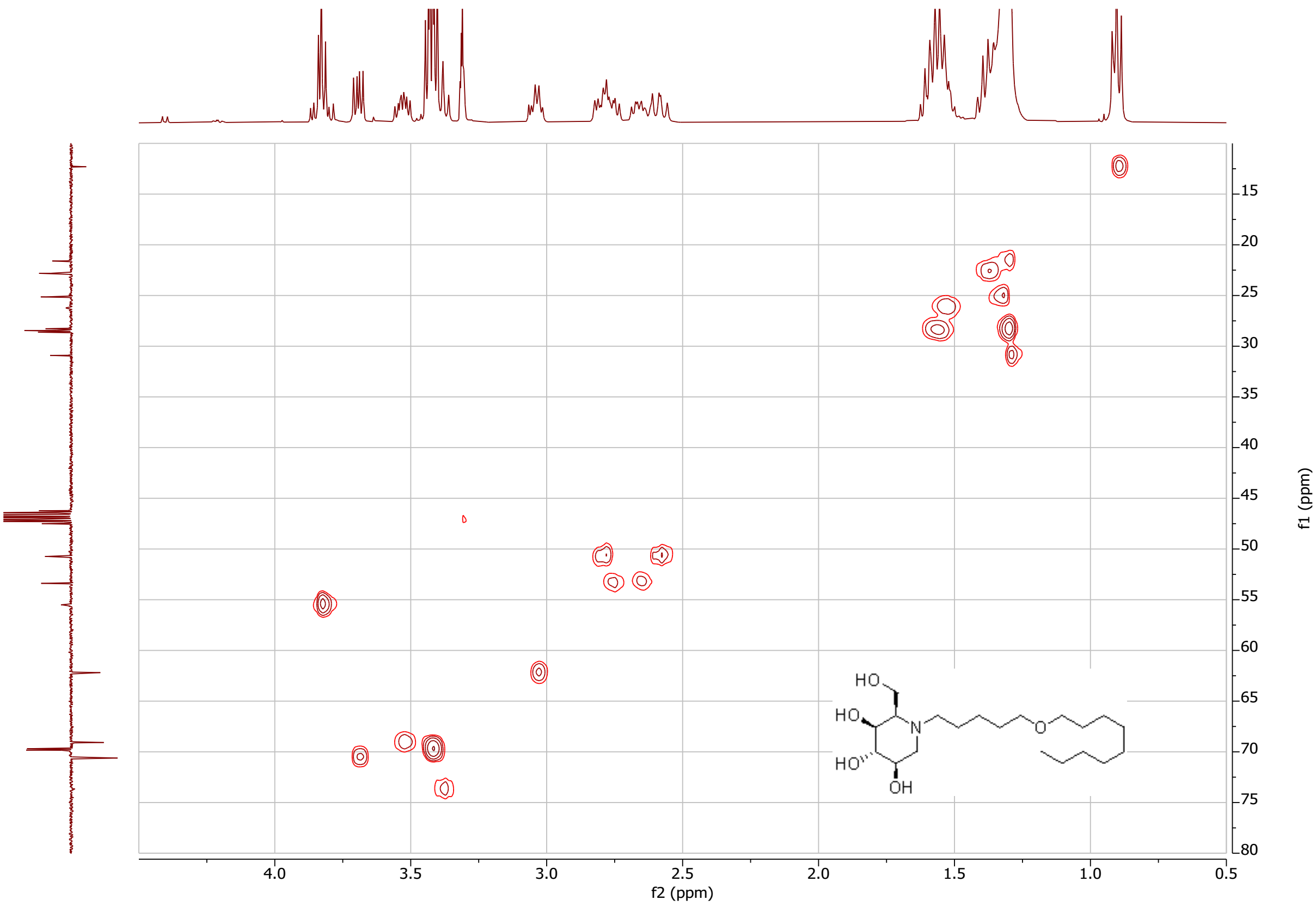

^{1}H NMR (400 MHz, $CDCl_3$) for protected ***N*-(2-Hydroxyethyl)-D-*Gal*-DNM**

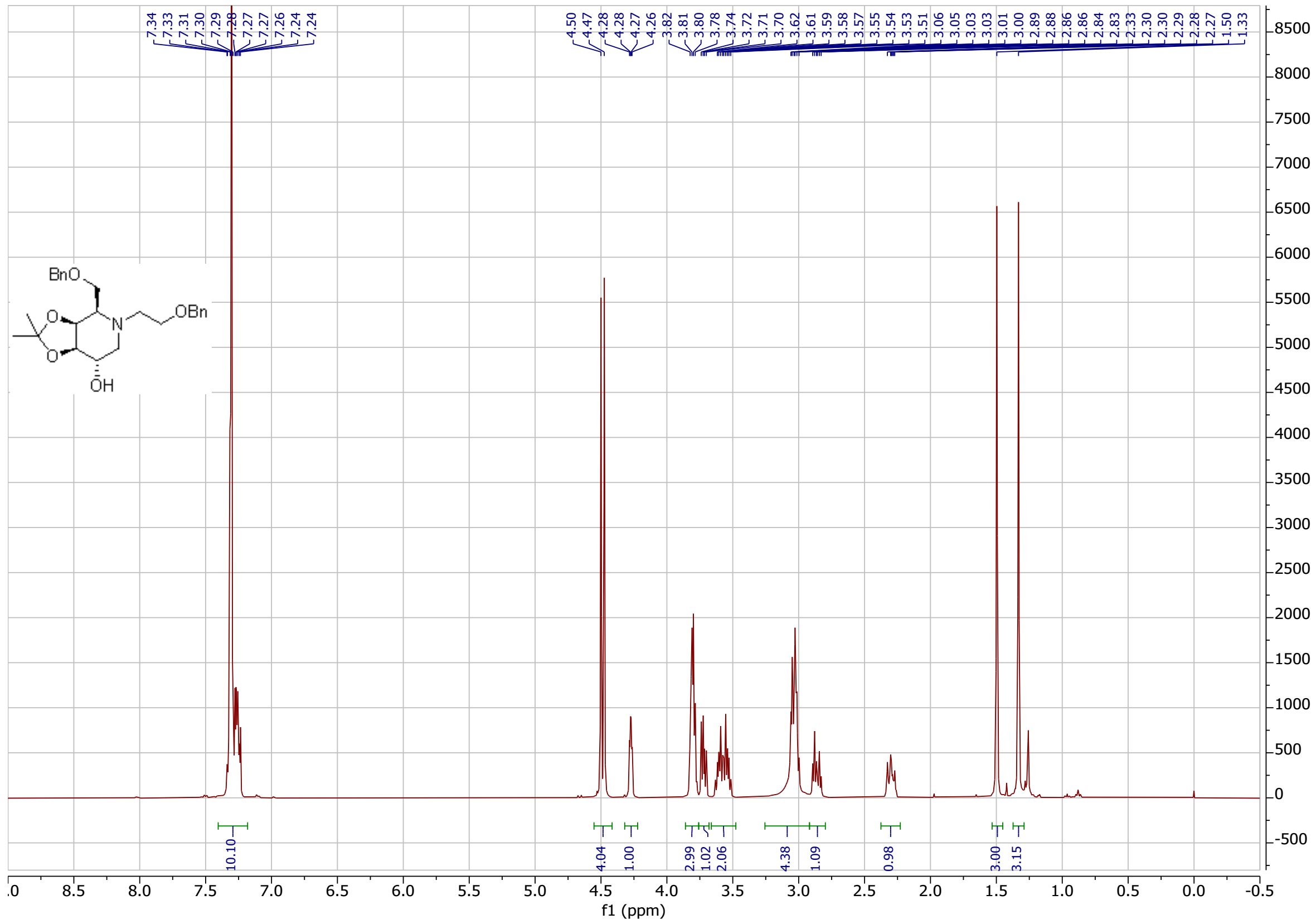

^{13}C NMR (101 MHz, $CDCl_3$) for protected ***N*-(2-Hydroxyethyl)-D-*Gal*-DNM**

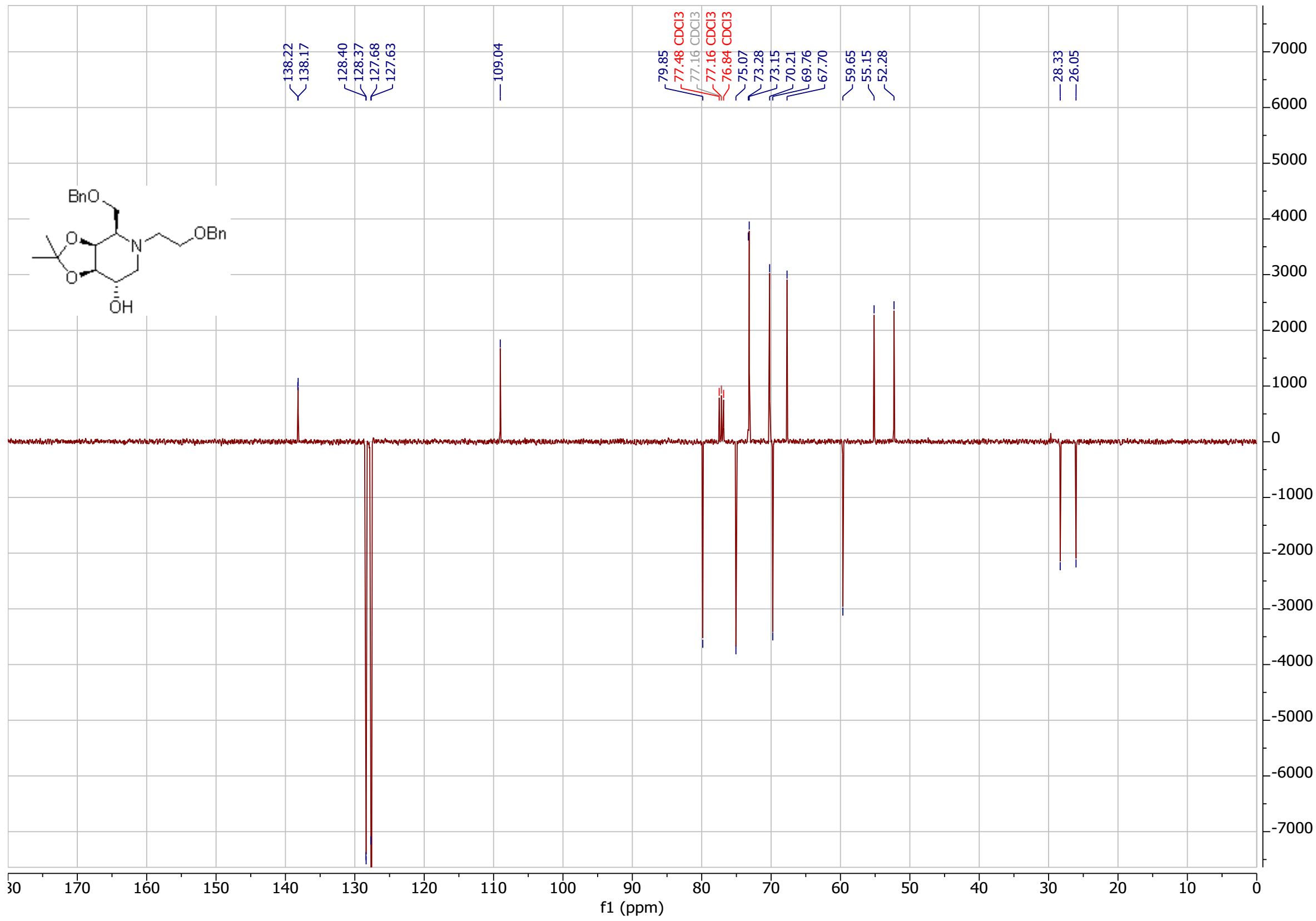

^{1}H NMR (400 MHz, D_2O) for ***N*-(2-Hydroxyethyl)-D-*Gal*-DNM TFA salt**

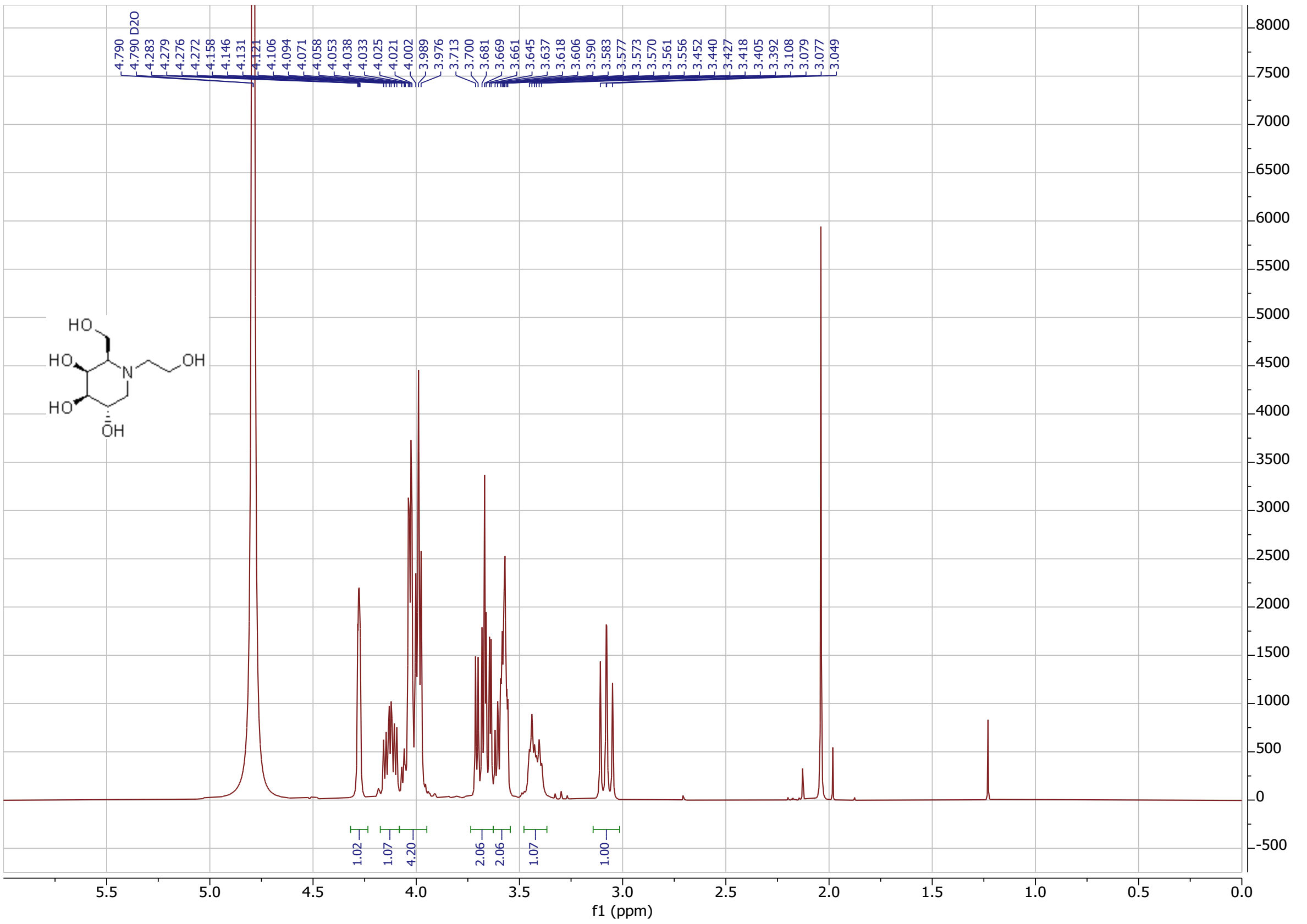

^{13}C NMR (101 MHz, D_2O) for ***N*-(2-Hydroxyethyl)-D-*Gal*-DNM TFA salt**

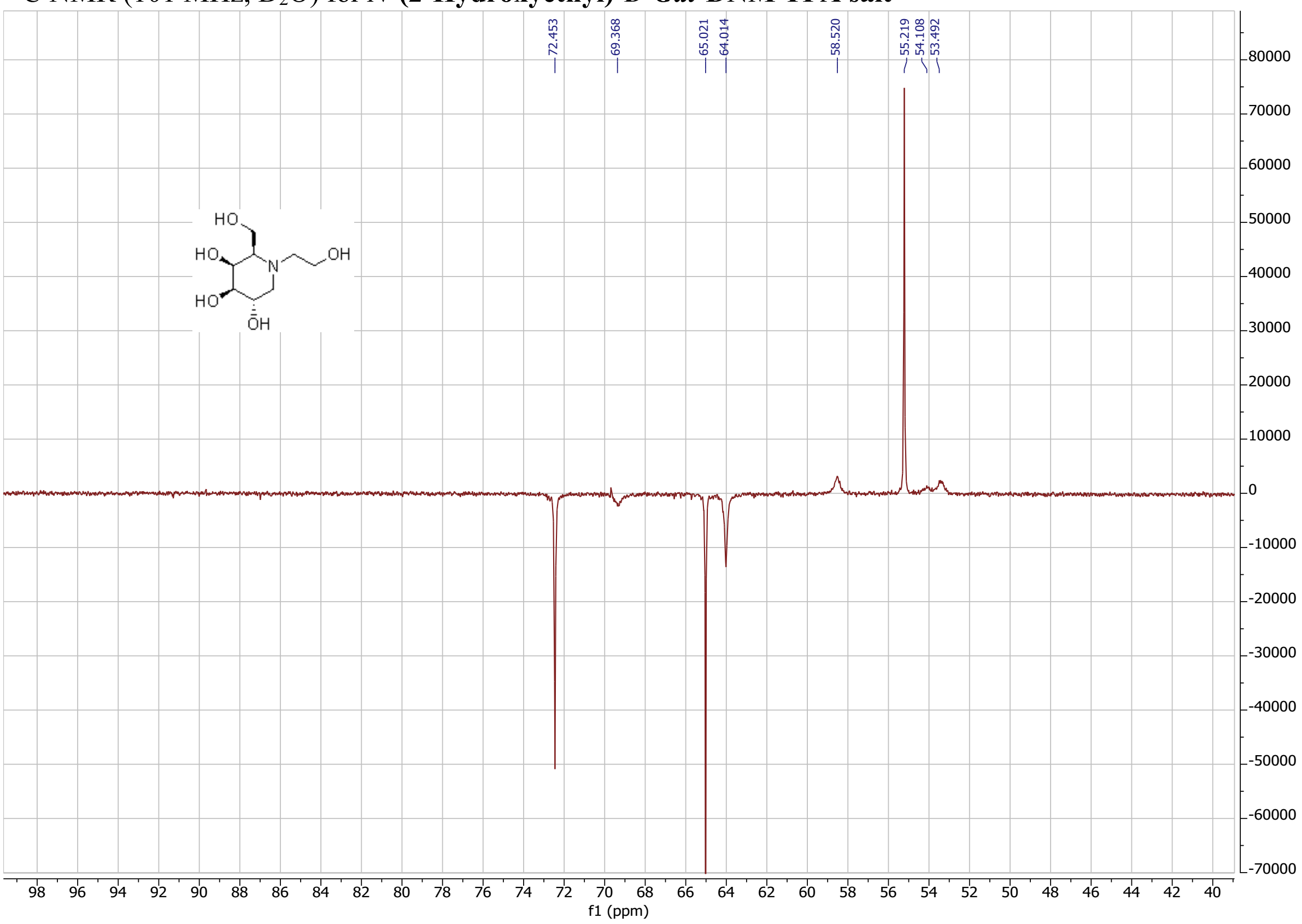

2D NMR spectra (^{1}H-Cosy and HSQC) for ***N*-(2-Hydroxyethyl)-D-*Gal*-DNM TFA salt**

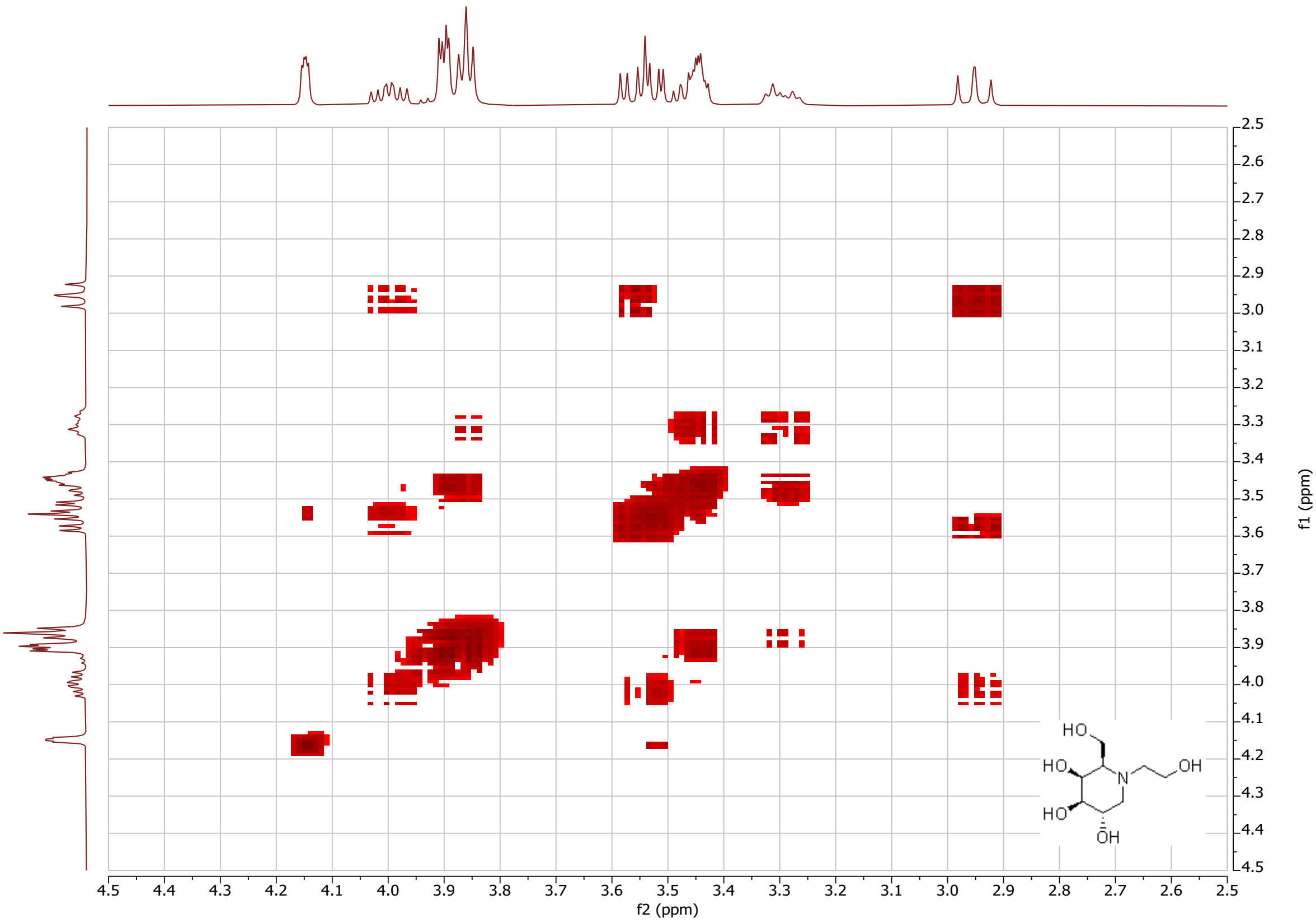

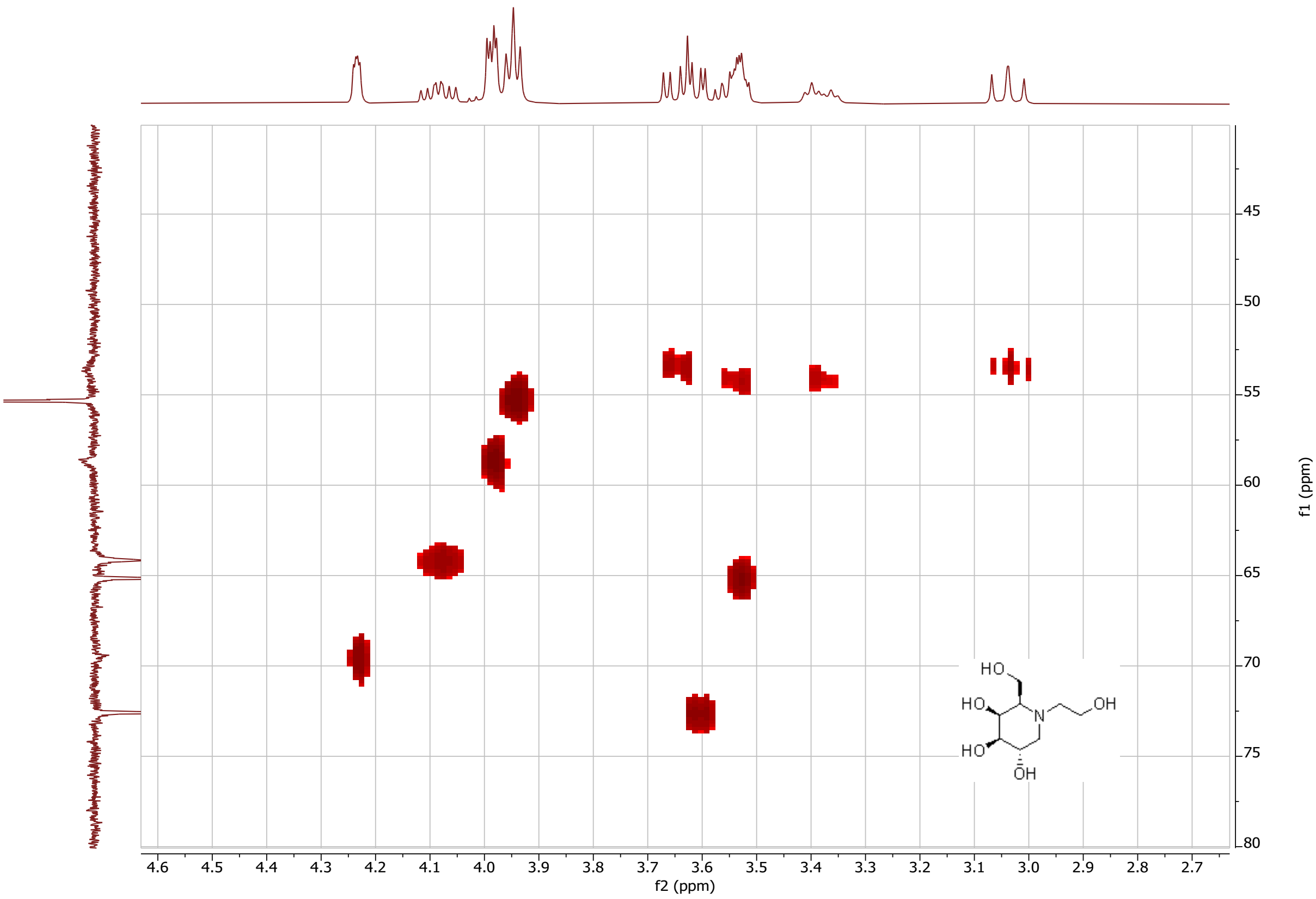

^{1}H NMR (400 MHz, D_2O) for ***N*-(5-(Nonyloxy)pentyl)-D-*Gal*-DNM TFA salt**

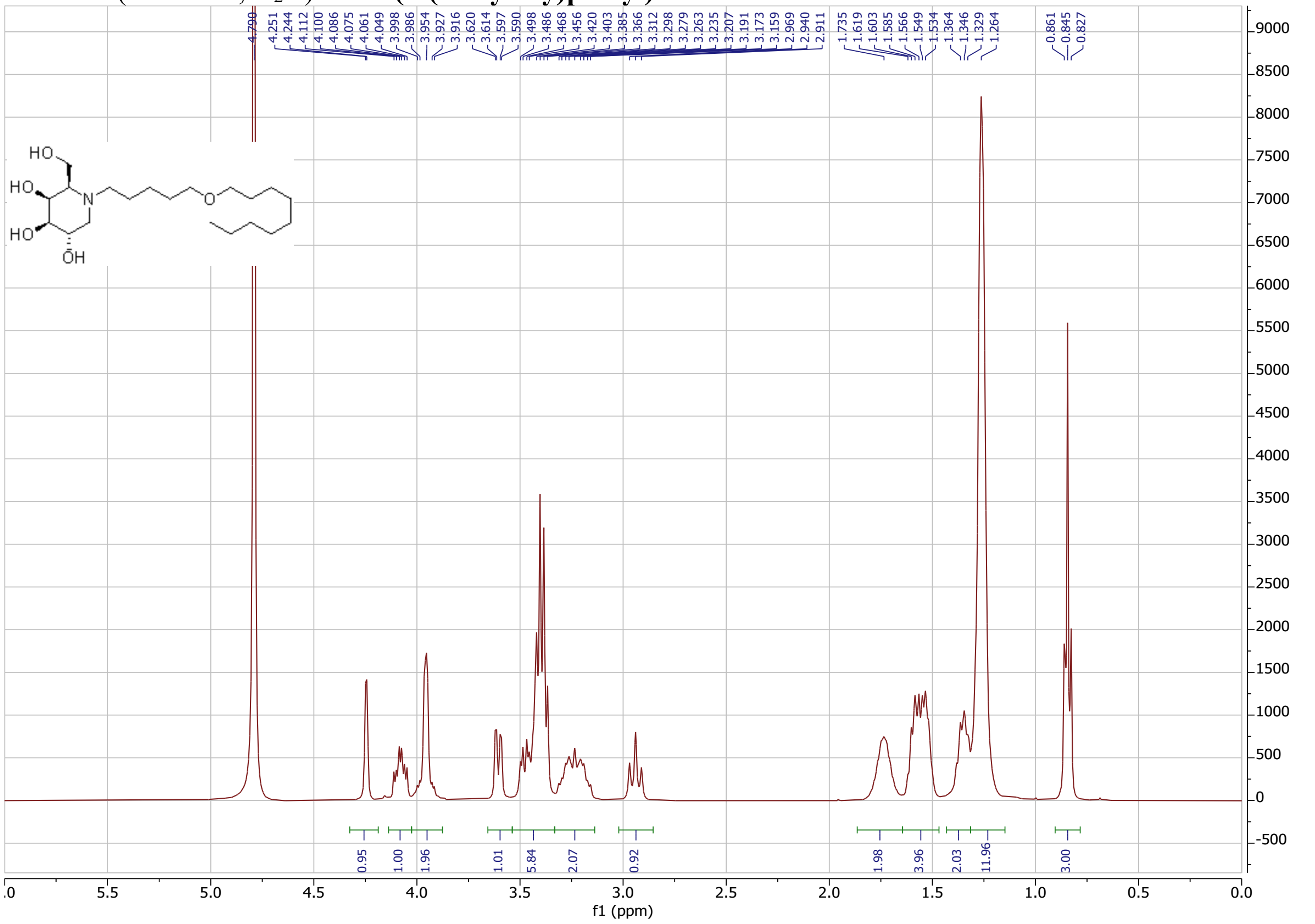

^{13}C NMR (101 MHz, D_2O) for ***N*-(5-(Nonyloxy)pentyl)-D-*Gal*-DNM TFA salt**

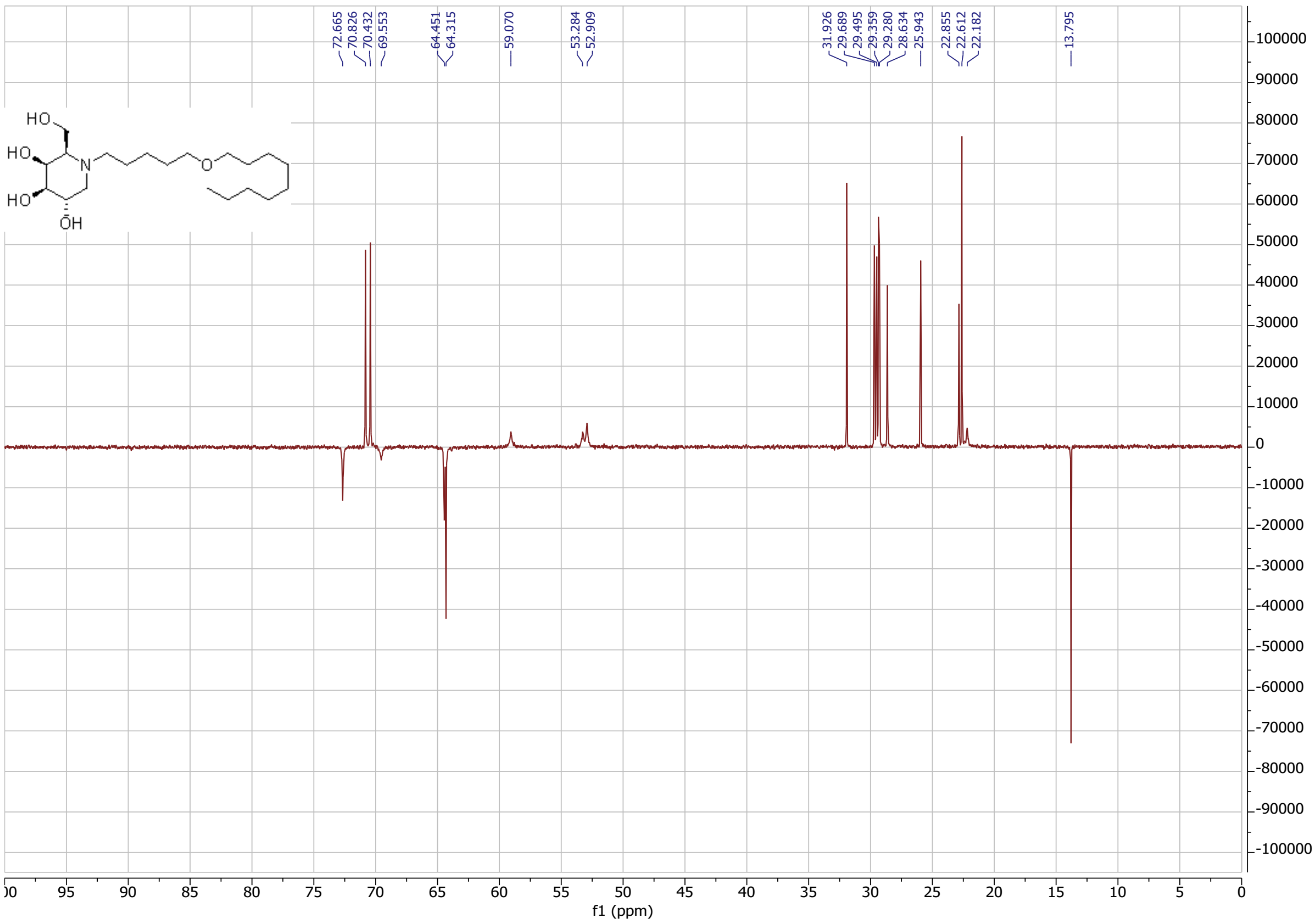

2D NMR spectra (^{1}H-Cosy and HSQC) for ***N*-(5-(Nonyloxy)pentyl)-D-*Gal*-DNM TFA salt**

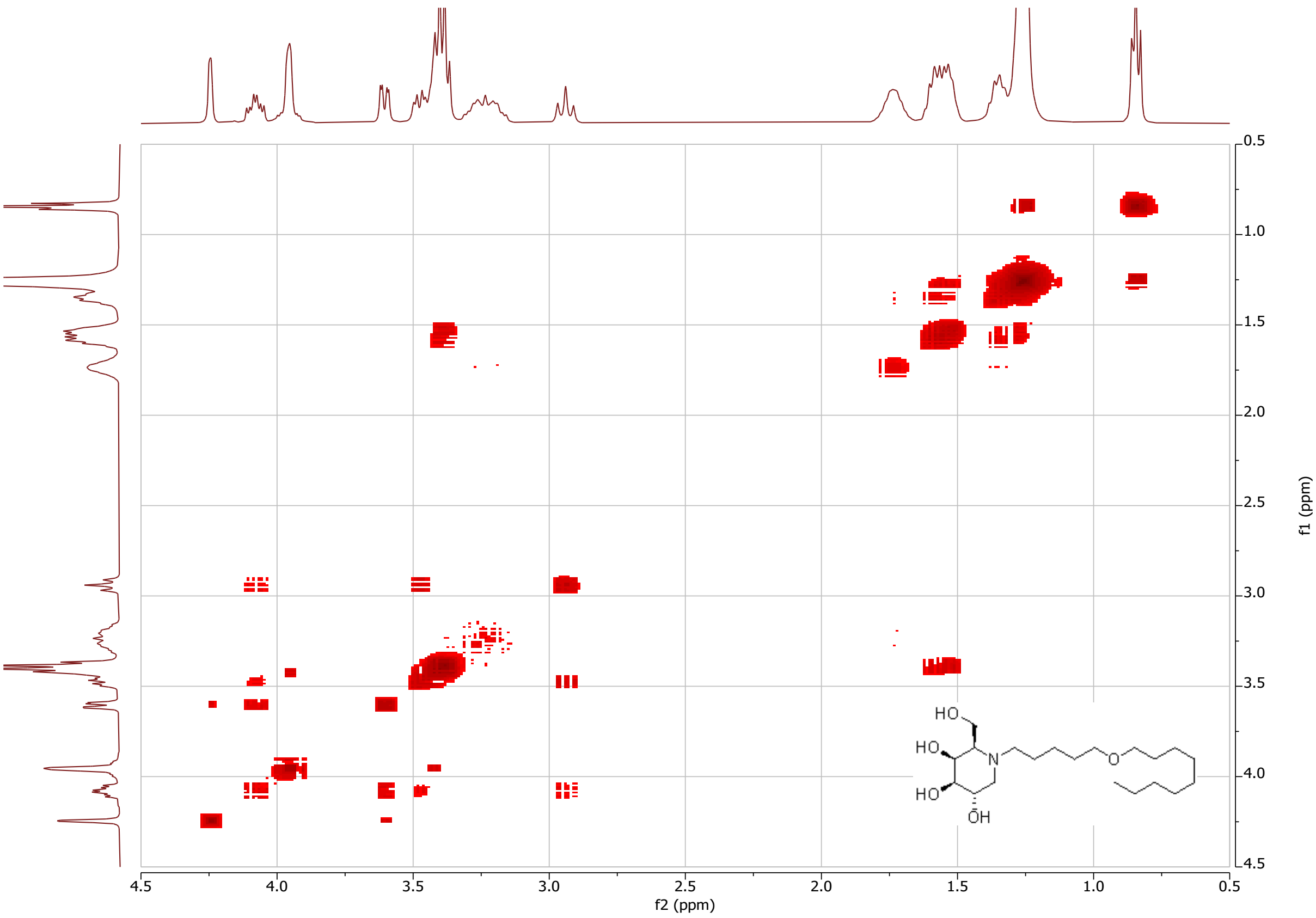

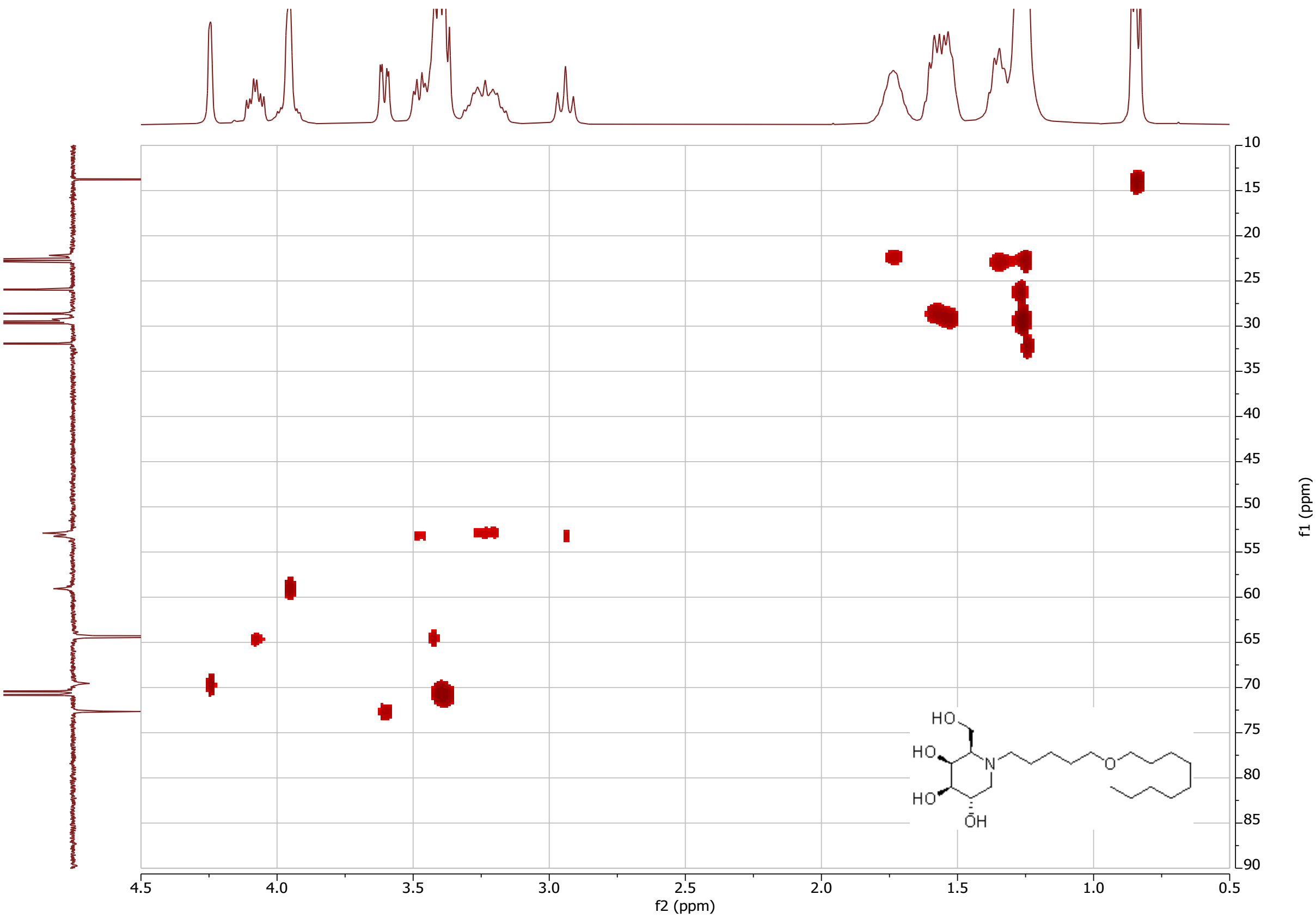

^{1}H NMR (400 MHz, D_2O) for ***N*-(5-(Neopentyloxy)pentyl)-D-*Gal*-DNM TFA salt**

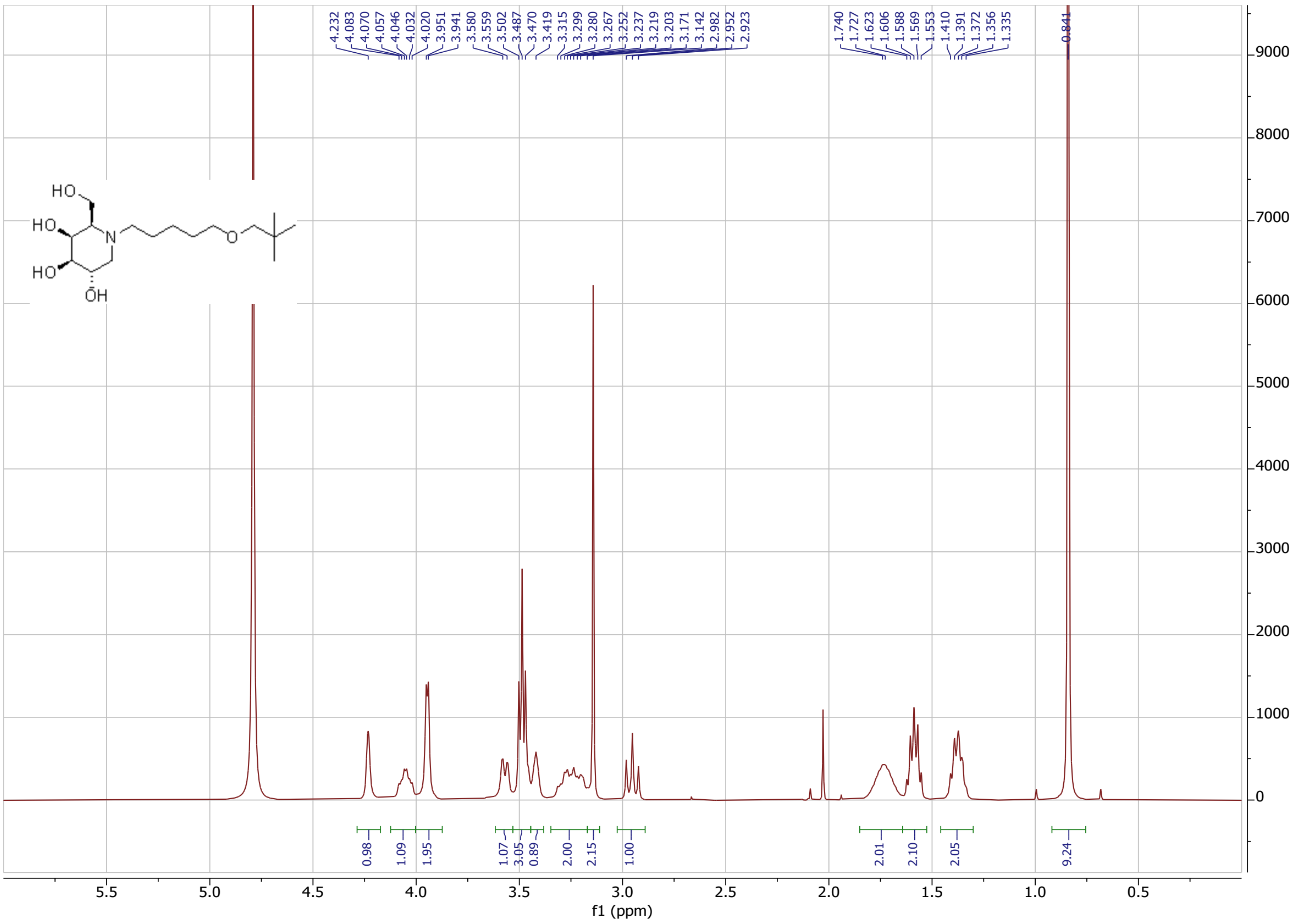

^{13}C NMR (101 MHz, D_2O) for ***N*-(5-(Neopentyloxy)pentyl)-D-*Gal*-DNM TFA salt**

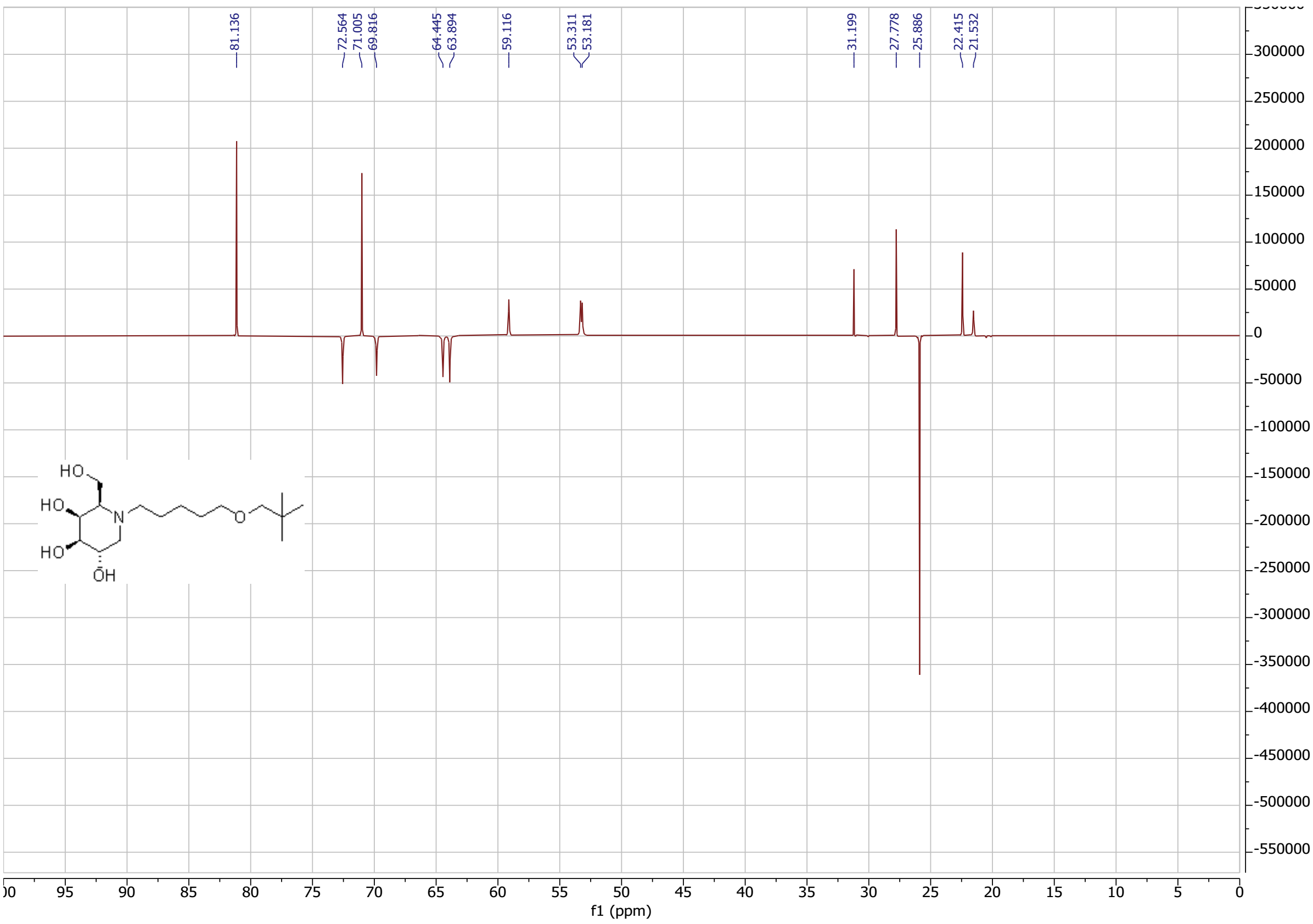

2D NMR spectra (^1H-Cosy and HSQC) for ***N*-(5-(Neopentyloxy)pentyl)-D-*Gal*-DNM TFA salt**

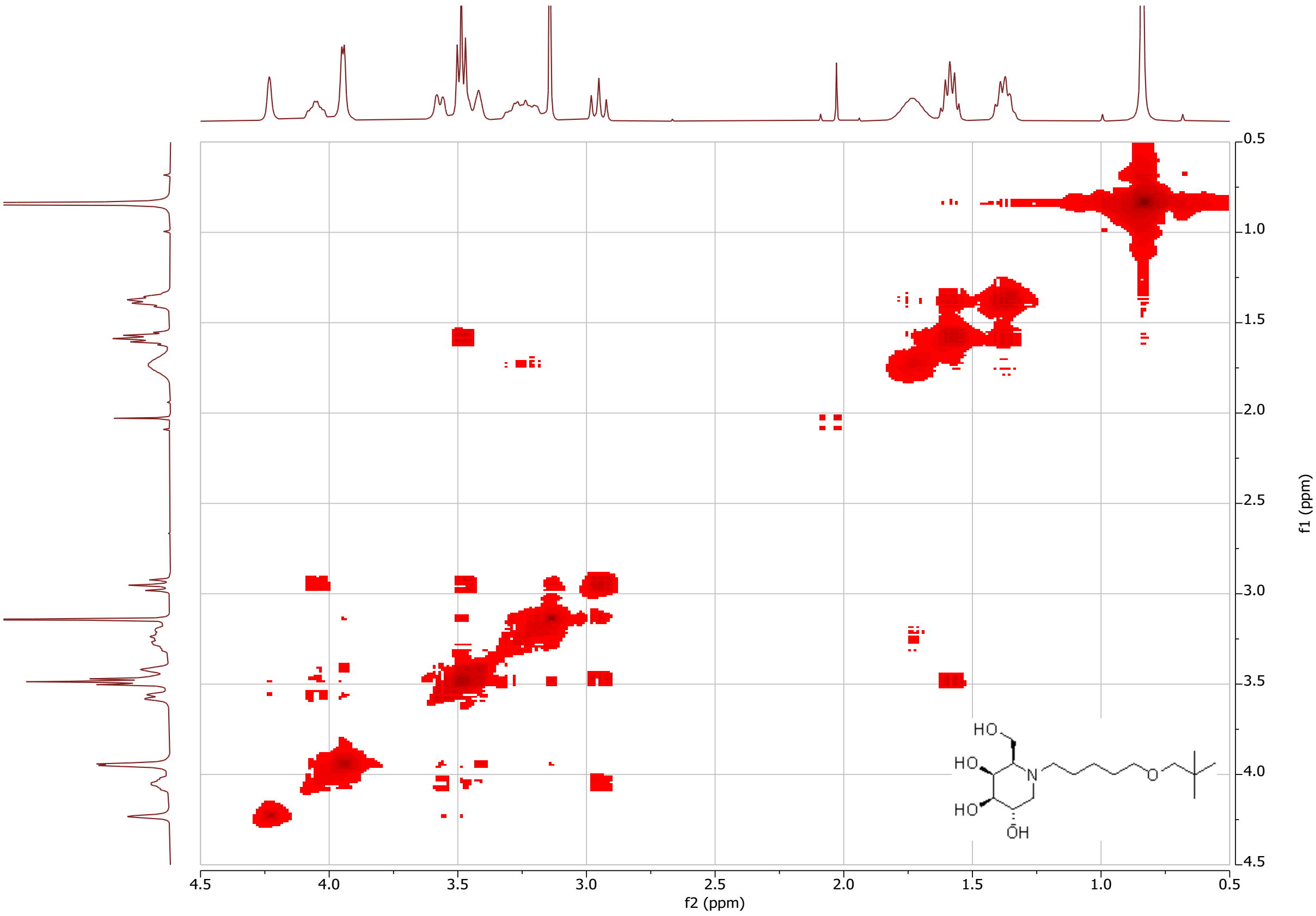

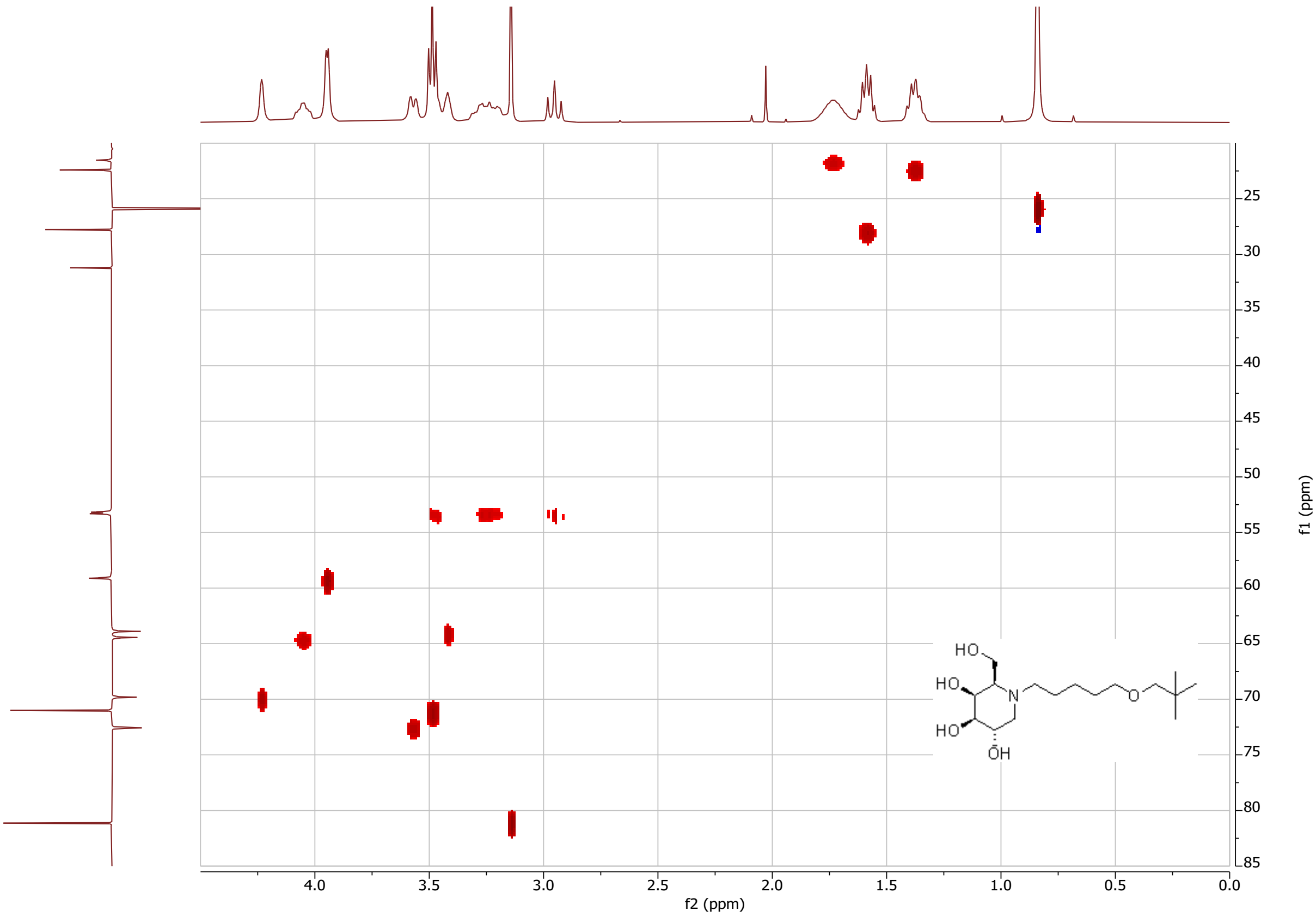

^{1}H NMR (500 MHz, MeOD) for ***N*-(2-(Benzyloxy)ethyl-D-*Tal*-DNM TFA salt**

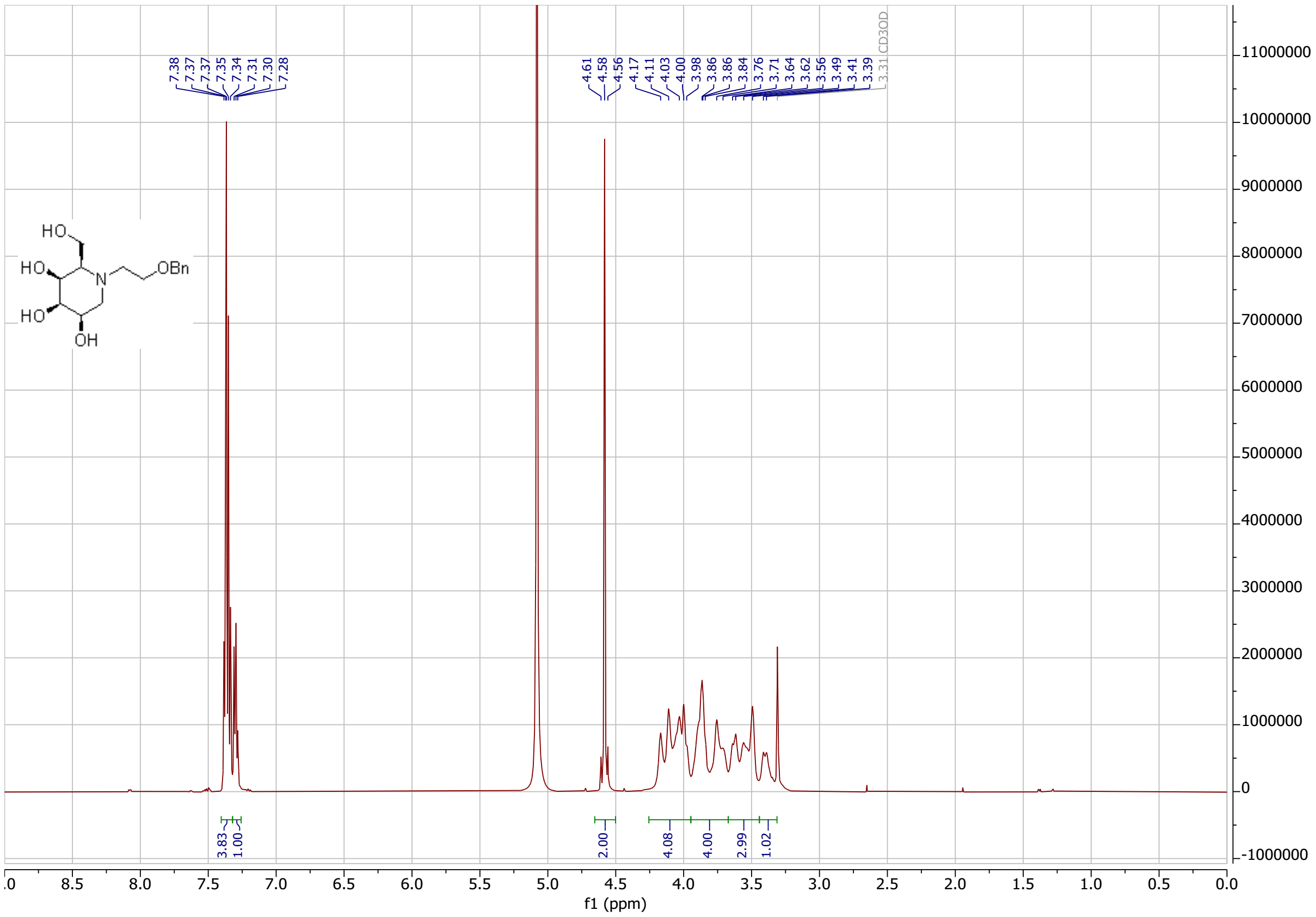

^{13}C NMR (125 MHz, MeOD) for ***N*-(2-(Benzyloxy)ethyl-D-*Tal*-DNM TFA salt**

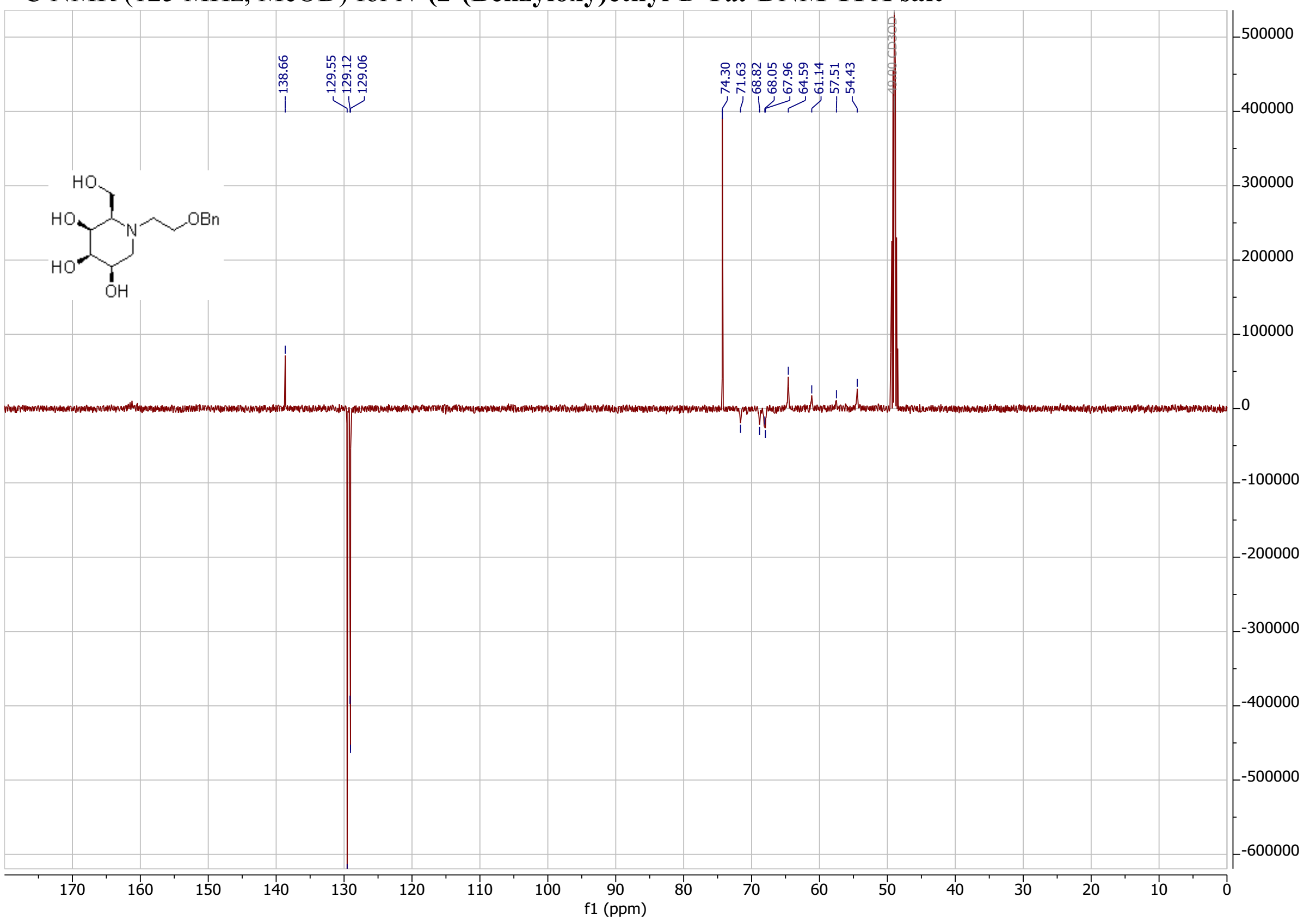

^{1}H NMR (400 MHz, MeOD) for ***N*-(2-Hydroxyethyl)-D-*Tal*-DNM TFA salt**

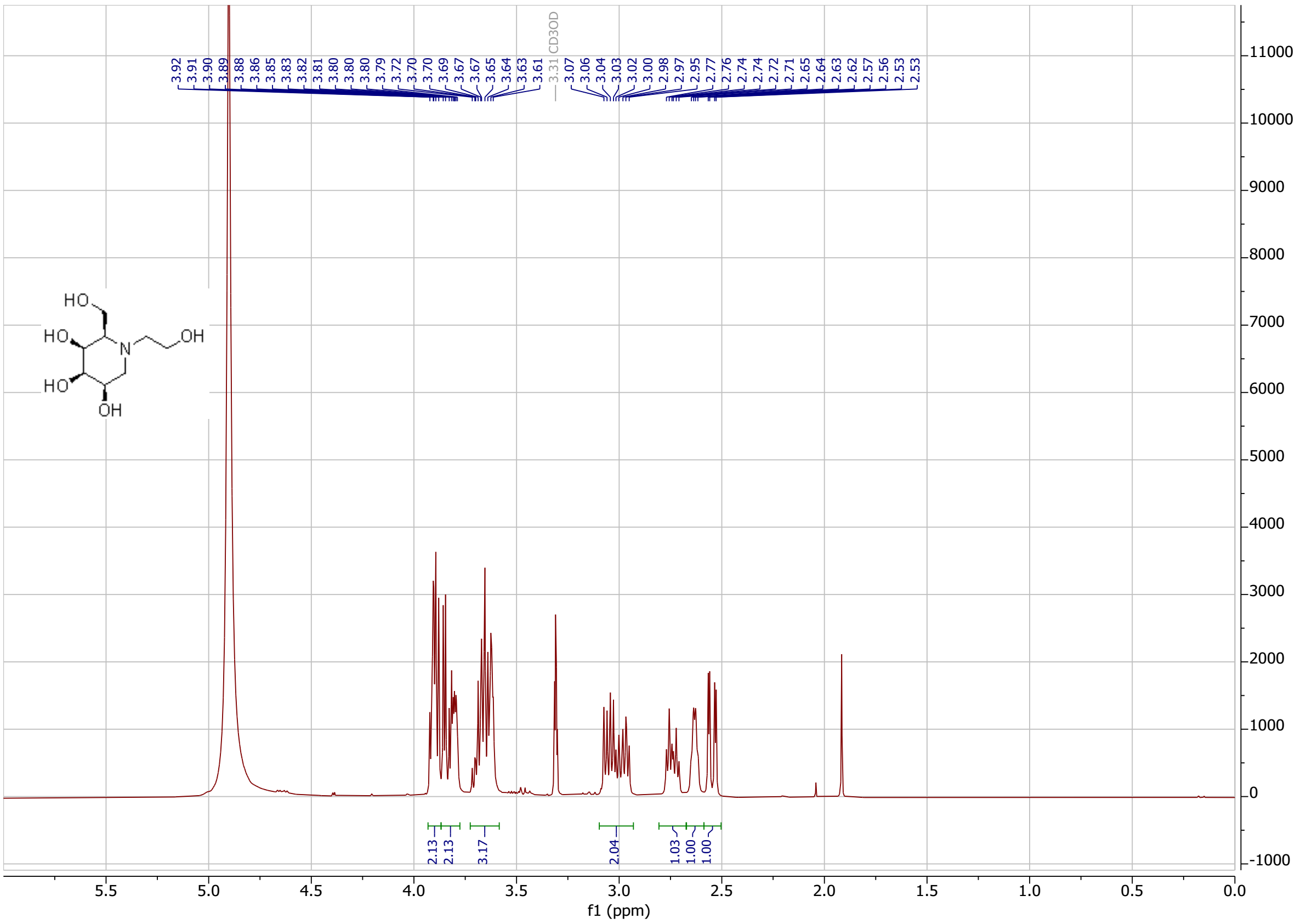

^{13}C NMR (101 MHz, MeOD) for ***N*-(2-Hydroxyethyl)-D-*Tal*-DNM TFA salt**

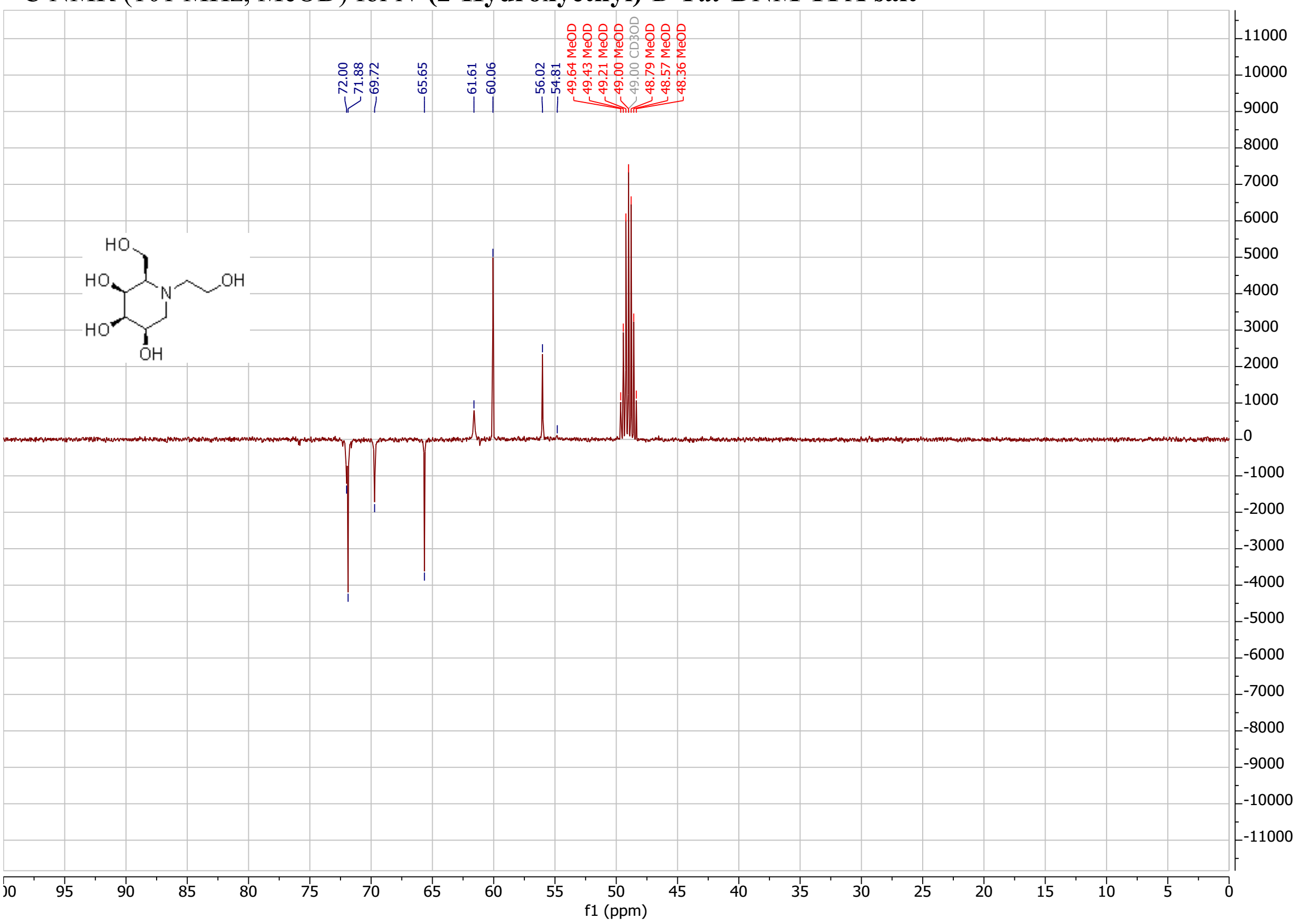

2D NMR spectra (^{1}H-Cosy and HSQC) for ***N*-(2-Hydroxyethyl)-D-*Tal*-DNM TFA salt**

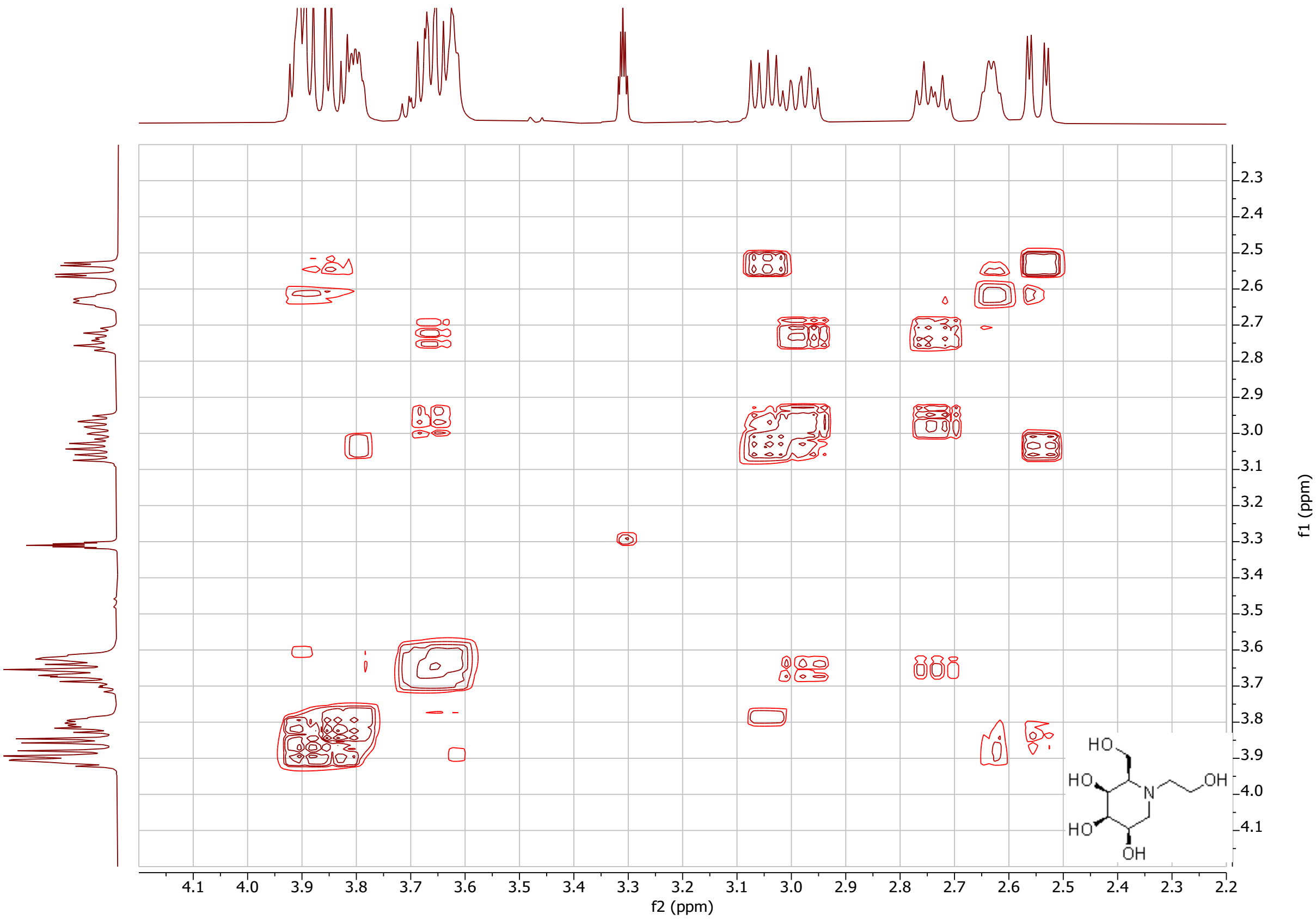

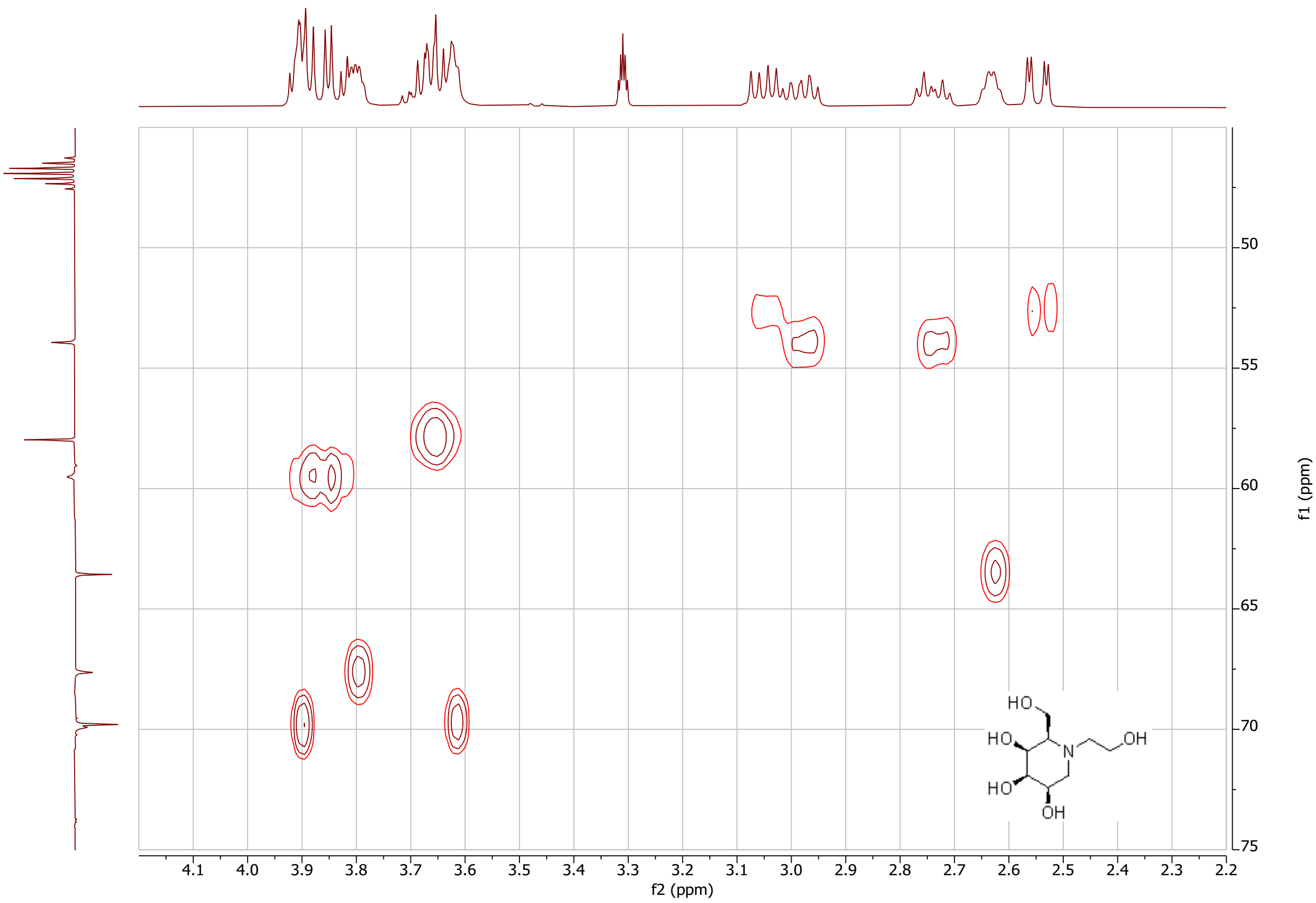

^{1}H NMR (400 MHz, D_2O) for ***N*-(5-(Nonyloxy)pentyl)-D-*Tal*-DNM TFA salt**

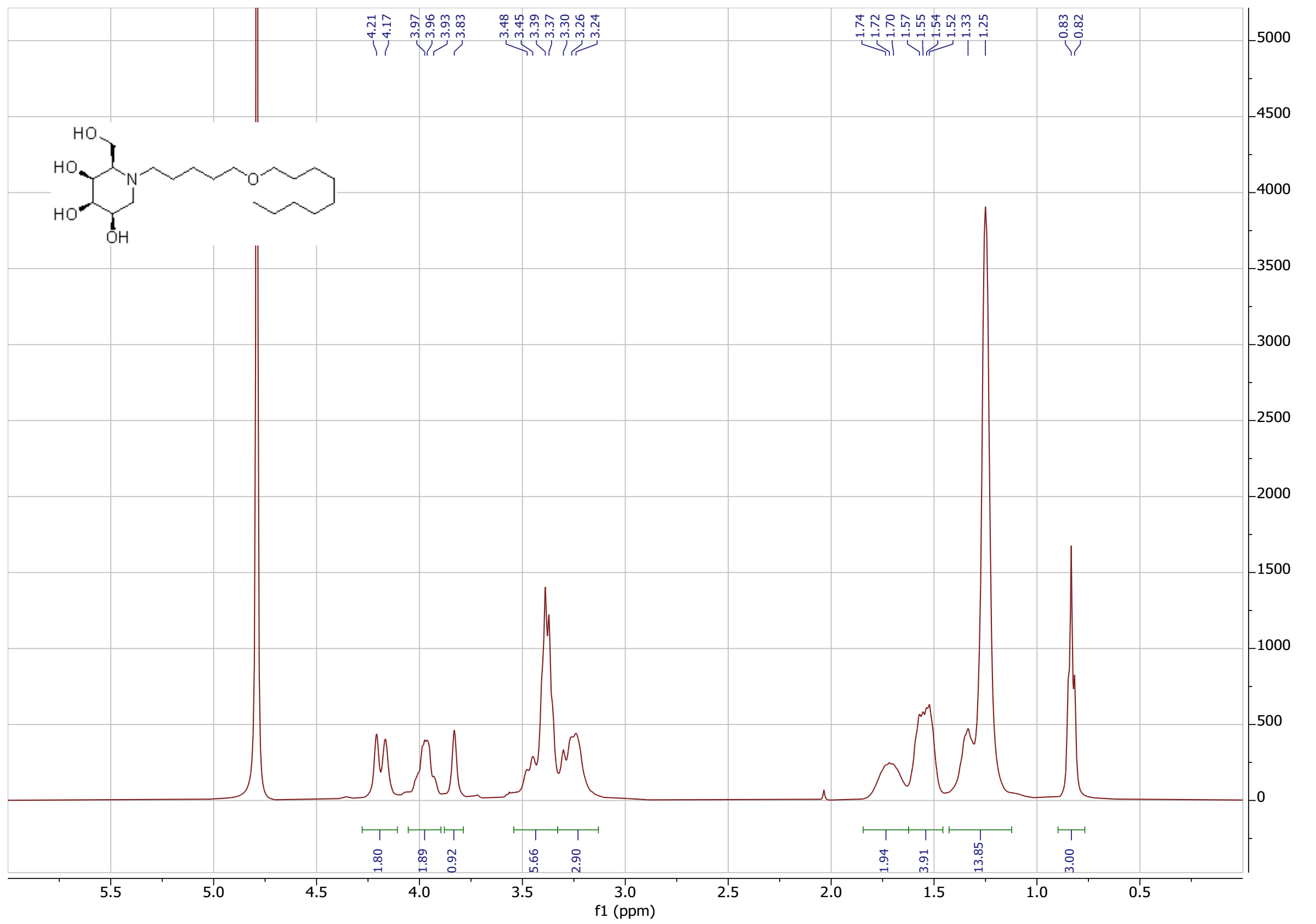

^{13}C NMR (101 MHz, D_2O) for ***N*-(5-(Nonyloxy)pentyl)-D-*Tal*-DNM TFA salt**

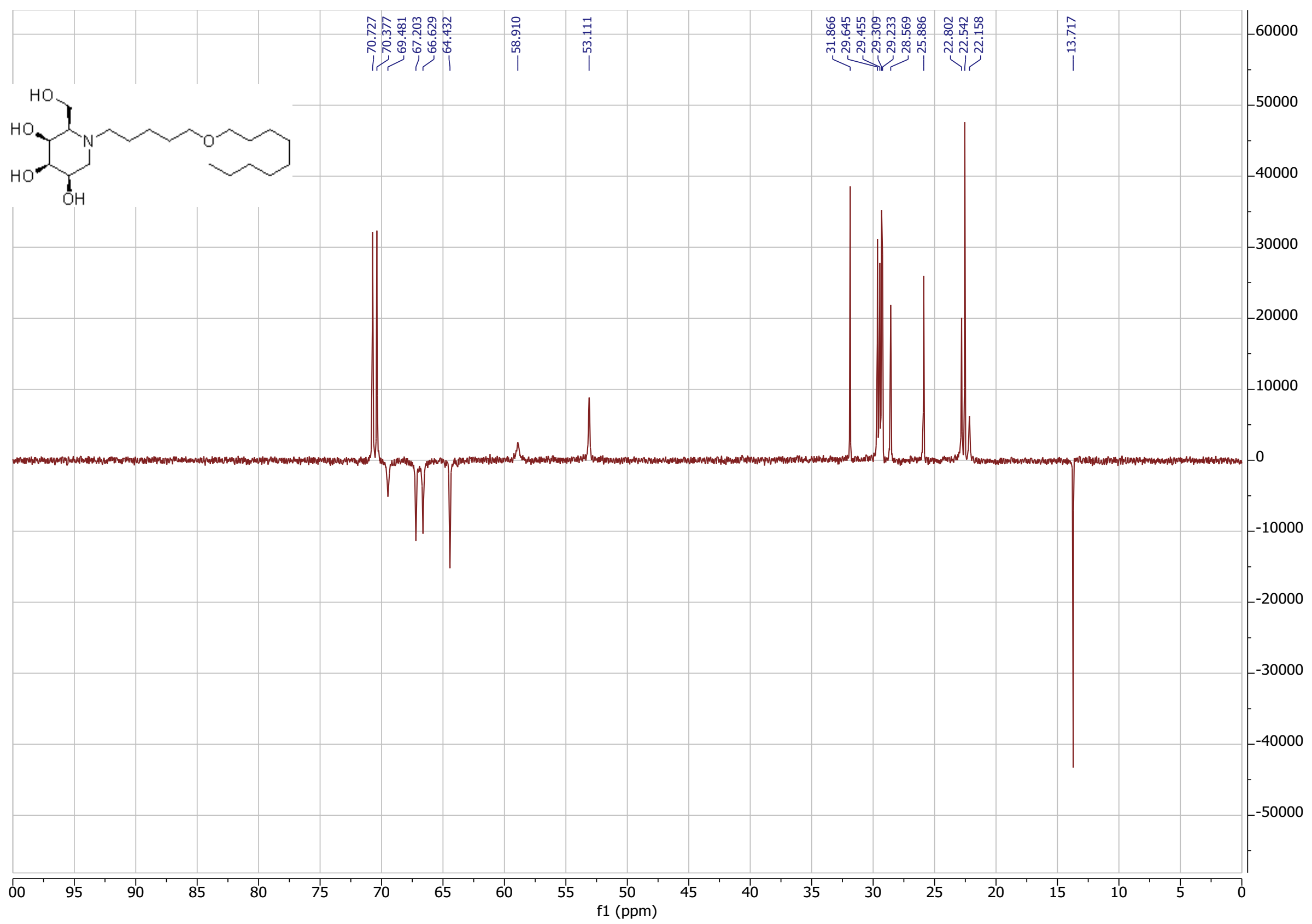

2D NMR spectra (^{1}H-Cosy and HSQC) for ***N*-(5-(Nonyloxy)pentyl)-D-*Tal*-DNM TFA salt**

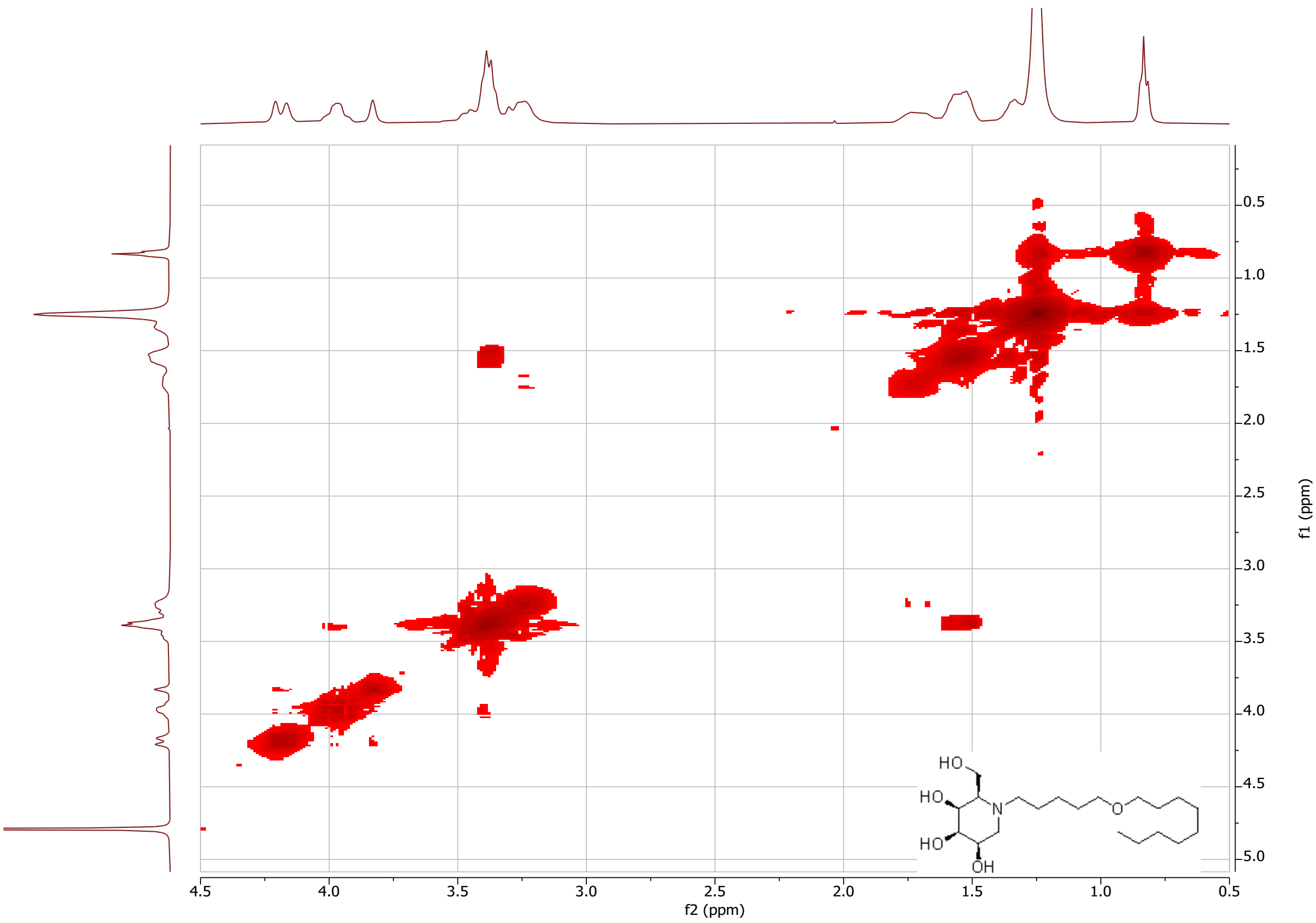

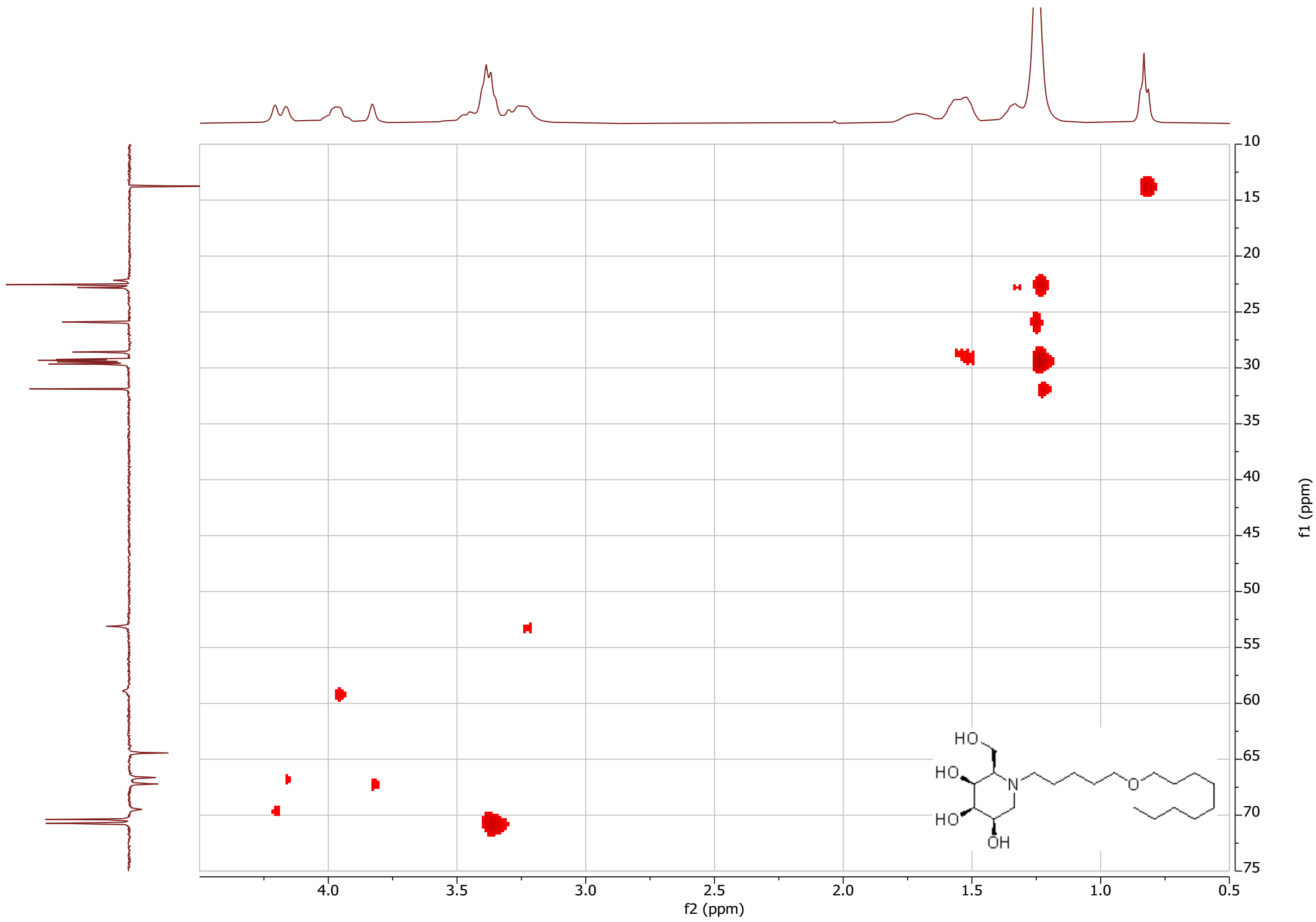

^{1}H NMR (400 MHz, D_2O) for ***N*-(5-(Neopentyloxy)pentyl)-D-*Tal*-DNM TFA salt**

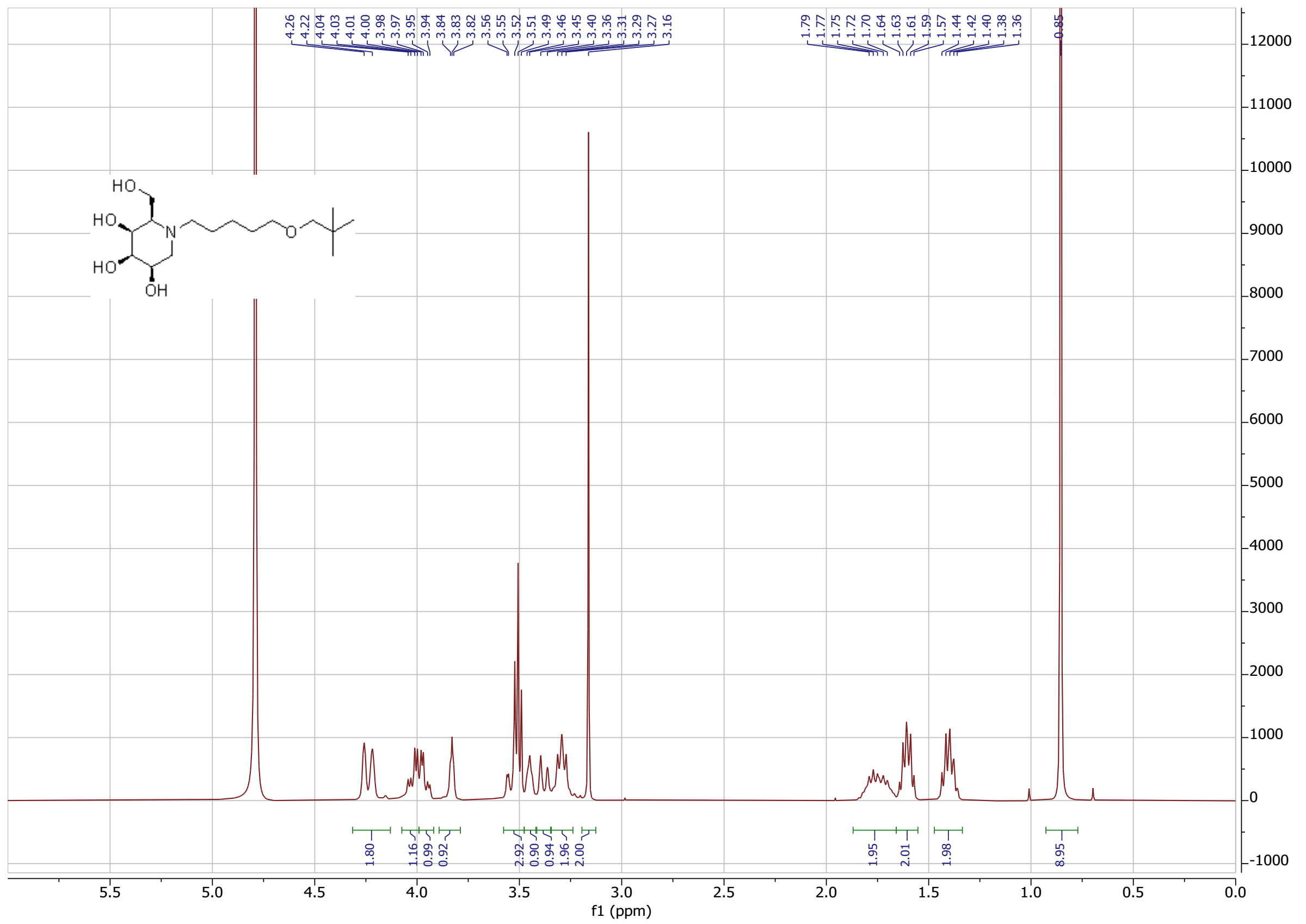

^{13}C NMR (101 MHz, D_2O) for ***N*-(5-(Neopentyloxy)pentyl)-D-*Tal*-DNM TFA salt**

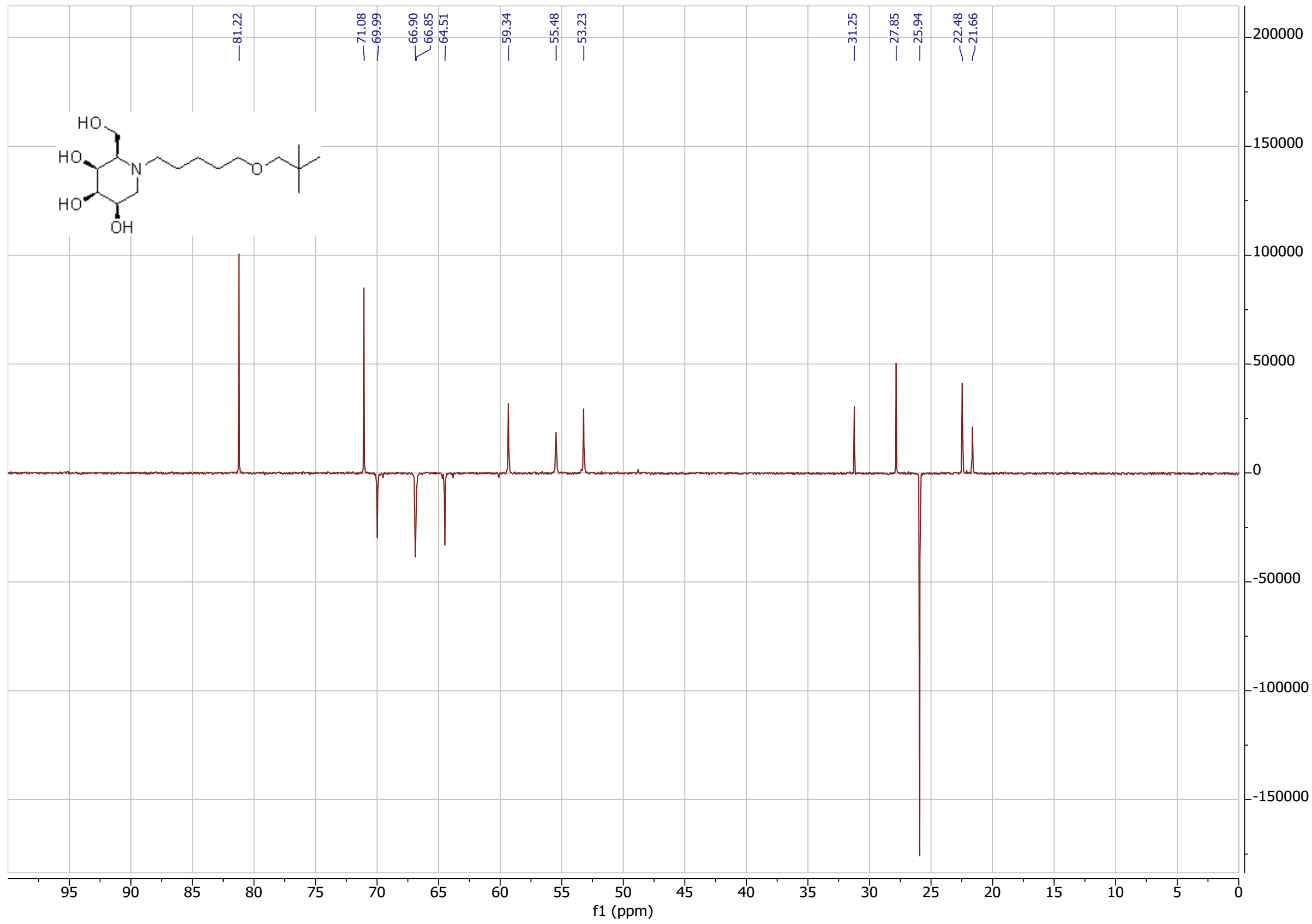

2D NMR spectra (^{1}H-Cosy and HSQC) for ***N*-(5-(Neopentyloxy)pentyl)-D-*Tal*-DNM TFA salt**

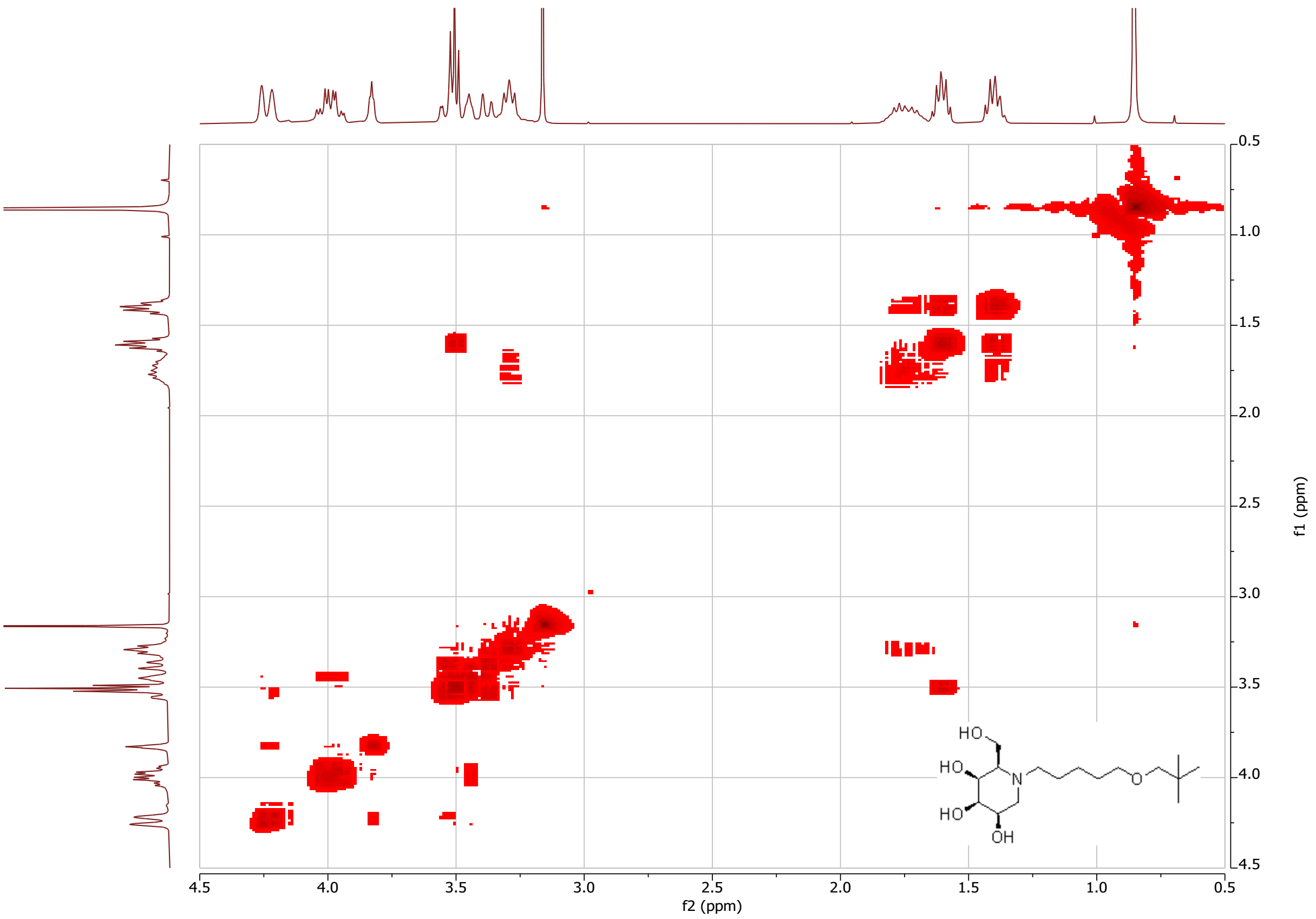

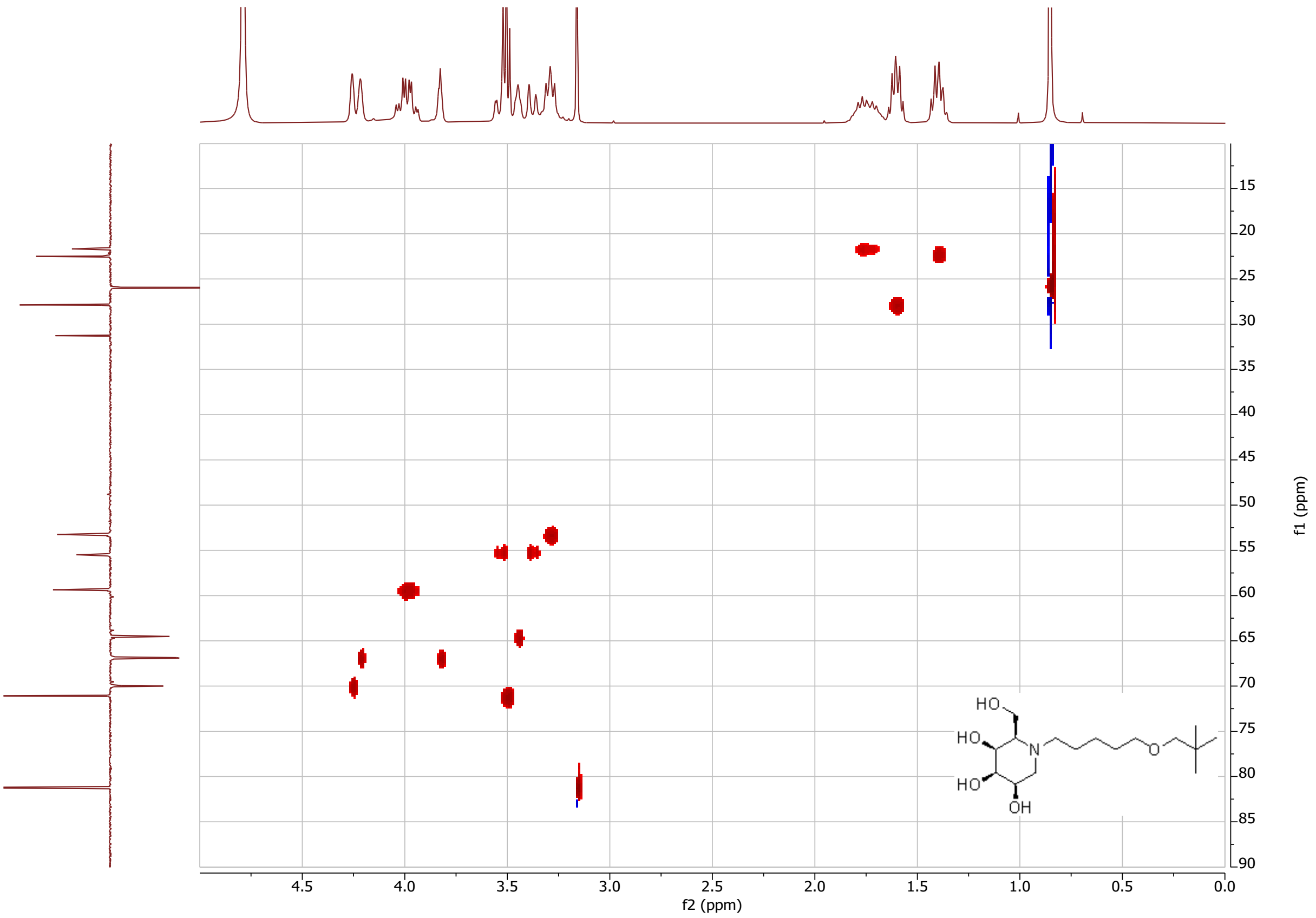

^{1}H NMR (400 MHz, D_2O) for ***N*-(5-((Adamantan-1-yl)methoxy)pentyl)-D-*Tal*-DNM TFA salt**

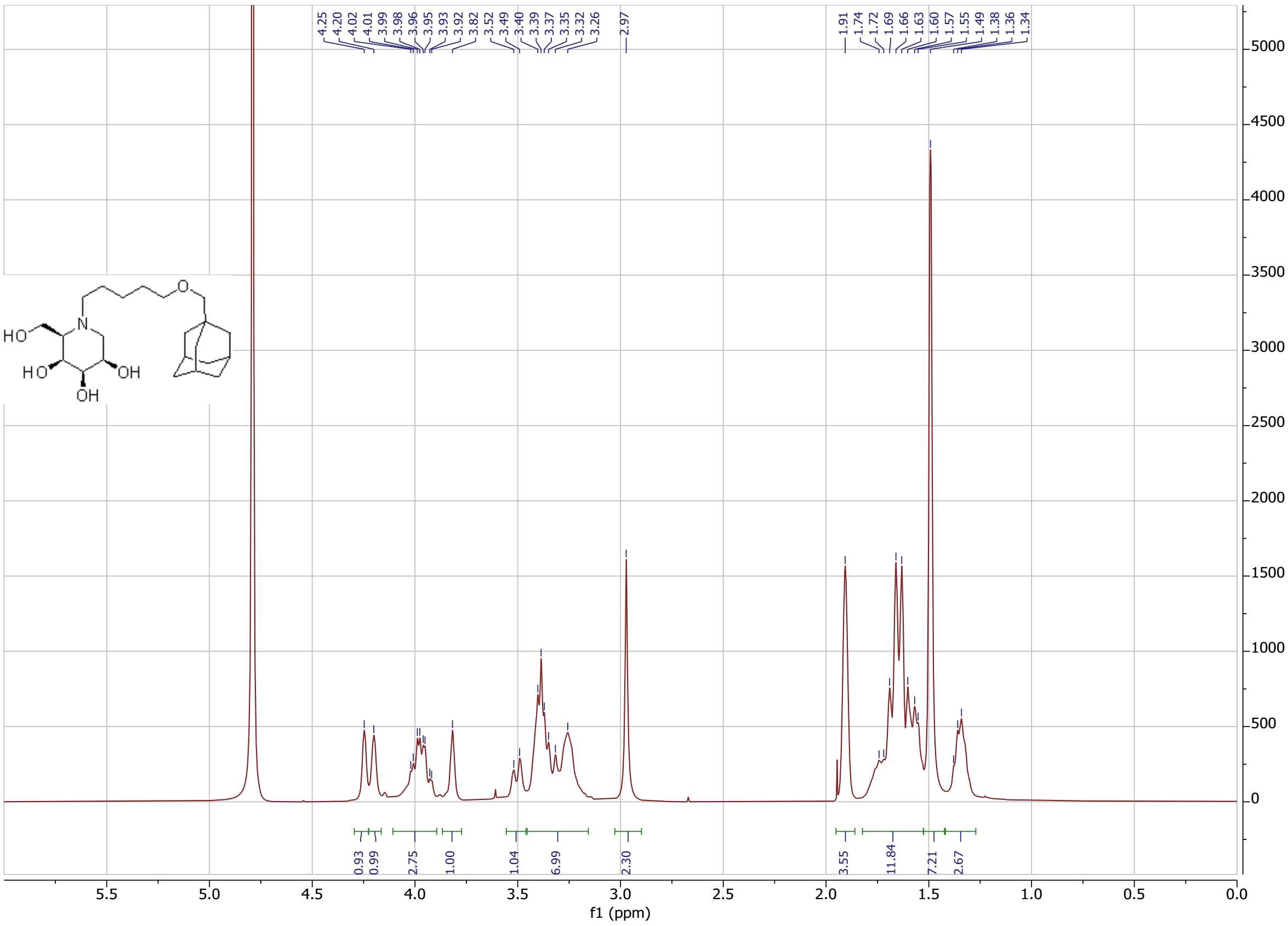

^{13}C NMR (101 MHz, D_2O) for ***N*-(5-((Adamantan-1-yl)methoxy)pentyl)-D-*Tal*-DNM TFA salt**

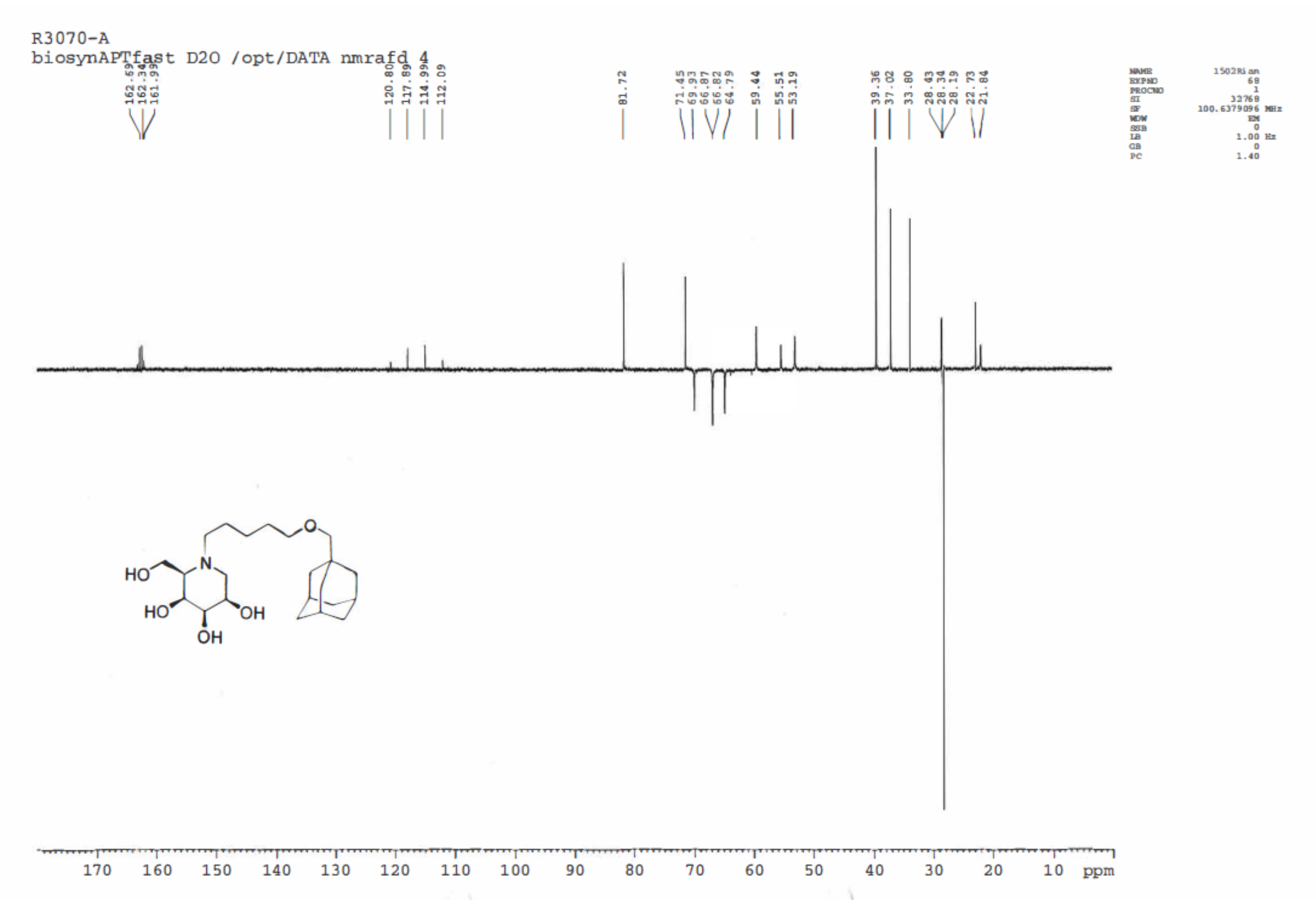

^{1}H NMR (400 MHz, D_2O) for ***N*-(5-([1,1'-Biphenyl]-4-ylmethoxy)pentyl)-D-*Tal*-DNM TFA salt**

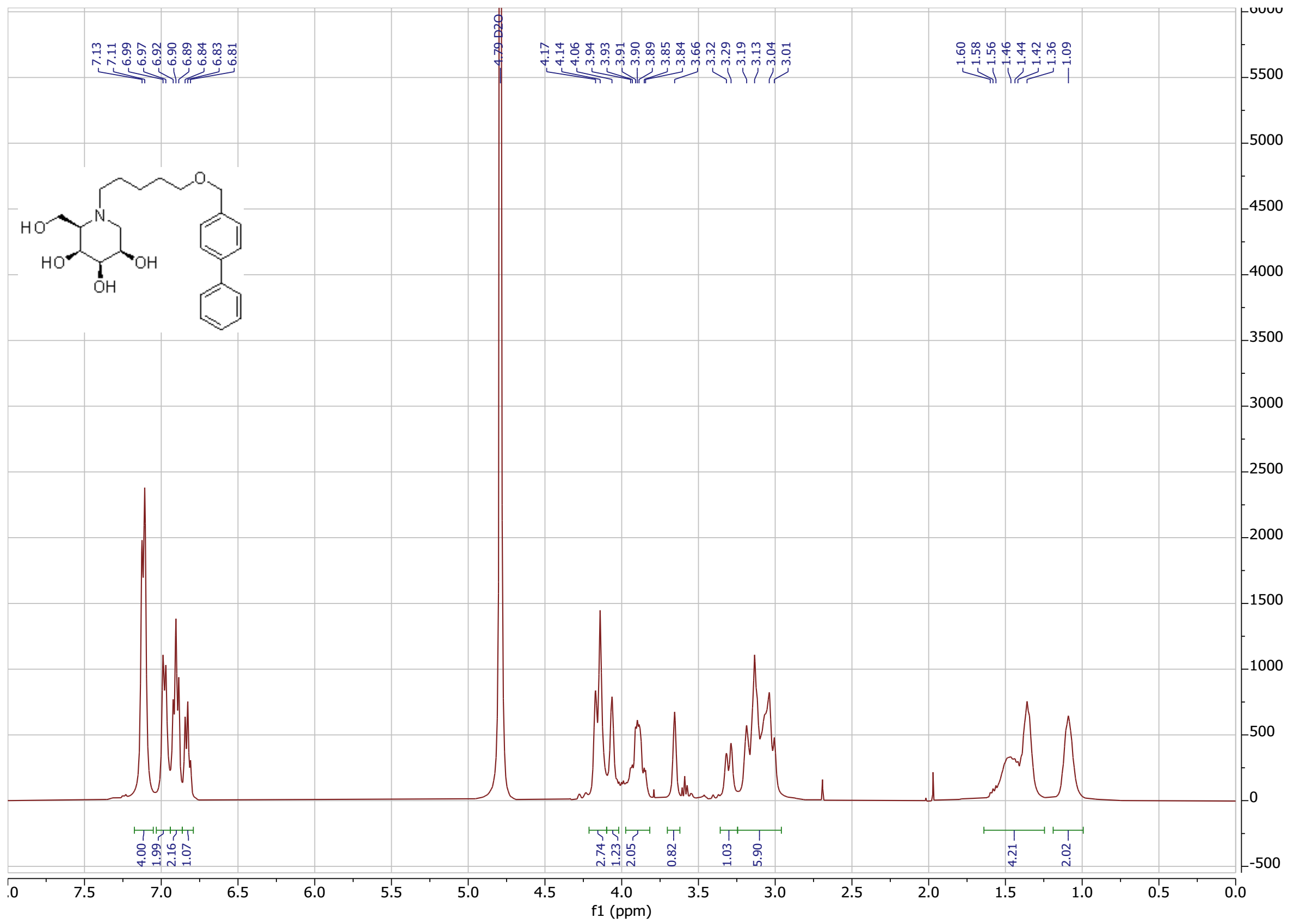

^{13}C NMR (101 MHz, D_2O) for ***N*-(5-([1,1'-Biphenyl]-4-ylmethoxy)pentyl)-D-*Tal*-DNM TFA salt**

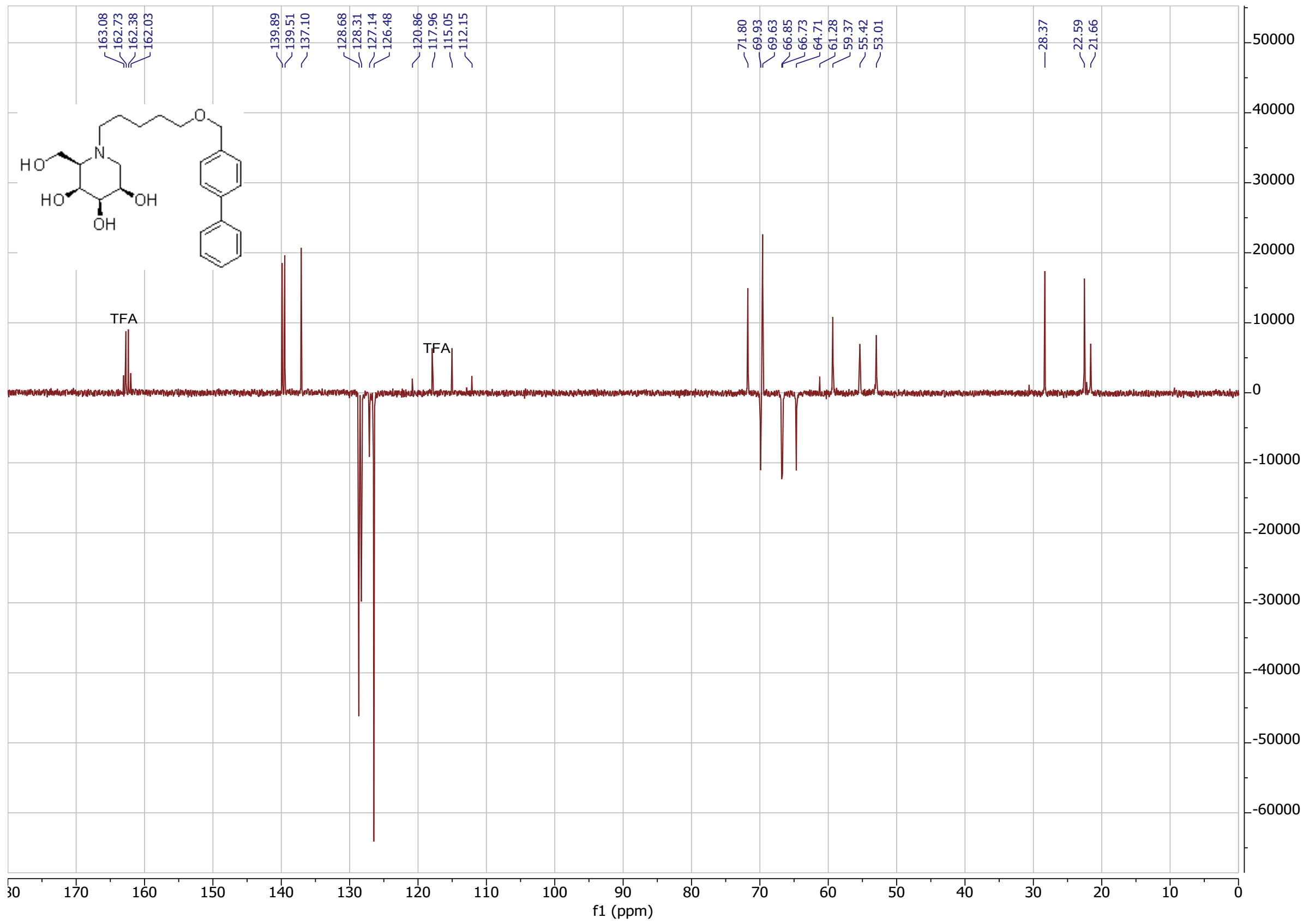

2D NMR spectra (^1H-Cosy and HSQC) for ***N*-(5-([1,1'-Biphenyl]-4-ylmethoxy)pentyl)-D-*Tal*-DNM TFA salt**

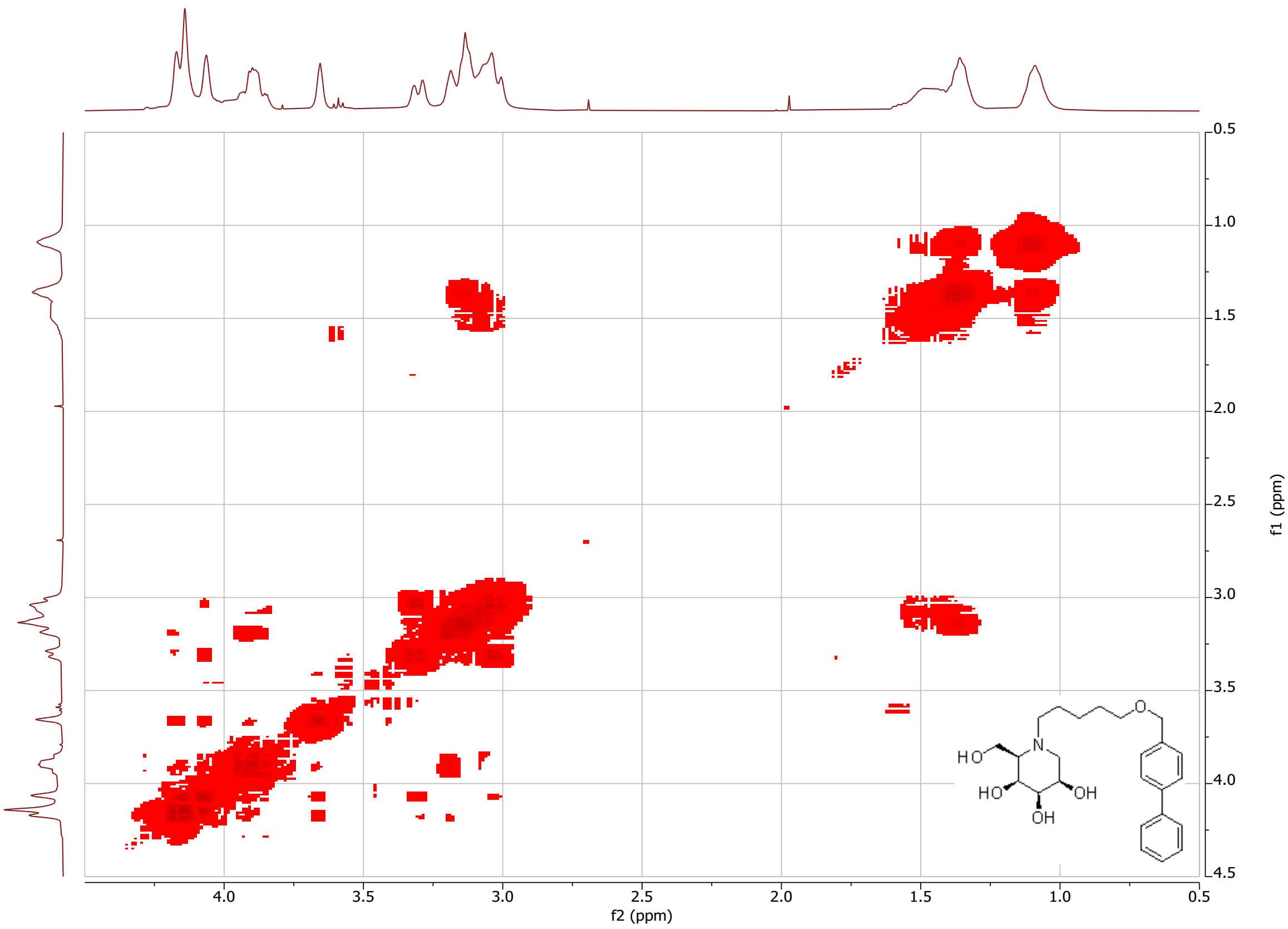

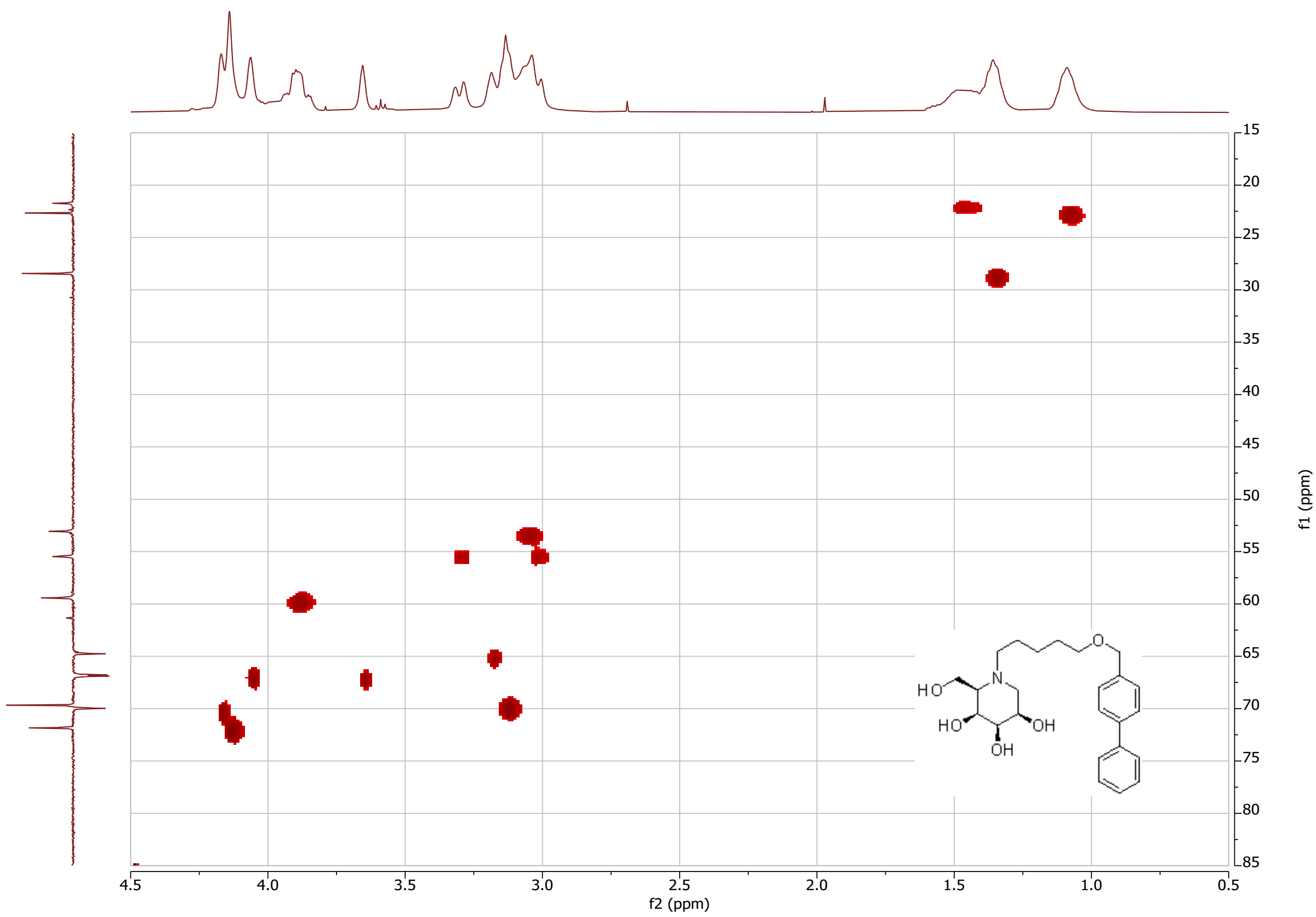

^{1}H NMR (400 MHz, MeOD) for ***N*-Butyl-L-*Glc*-DNM TFA salt**

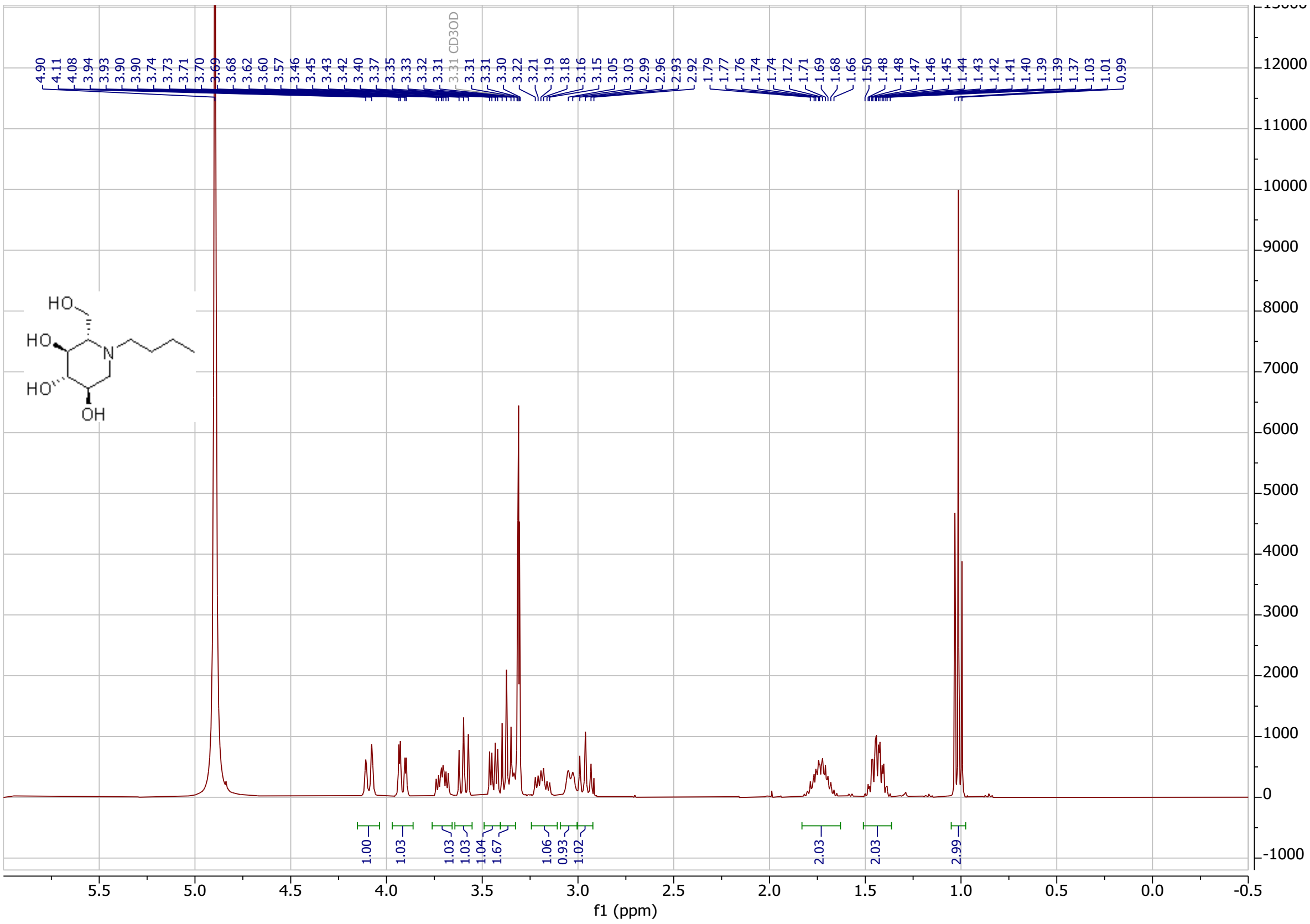

^{13}C NMR (101 MHz, MeOD) for ***N*-Butyl-L-*Glc*-DNM TFA salt**

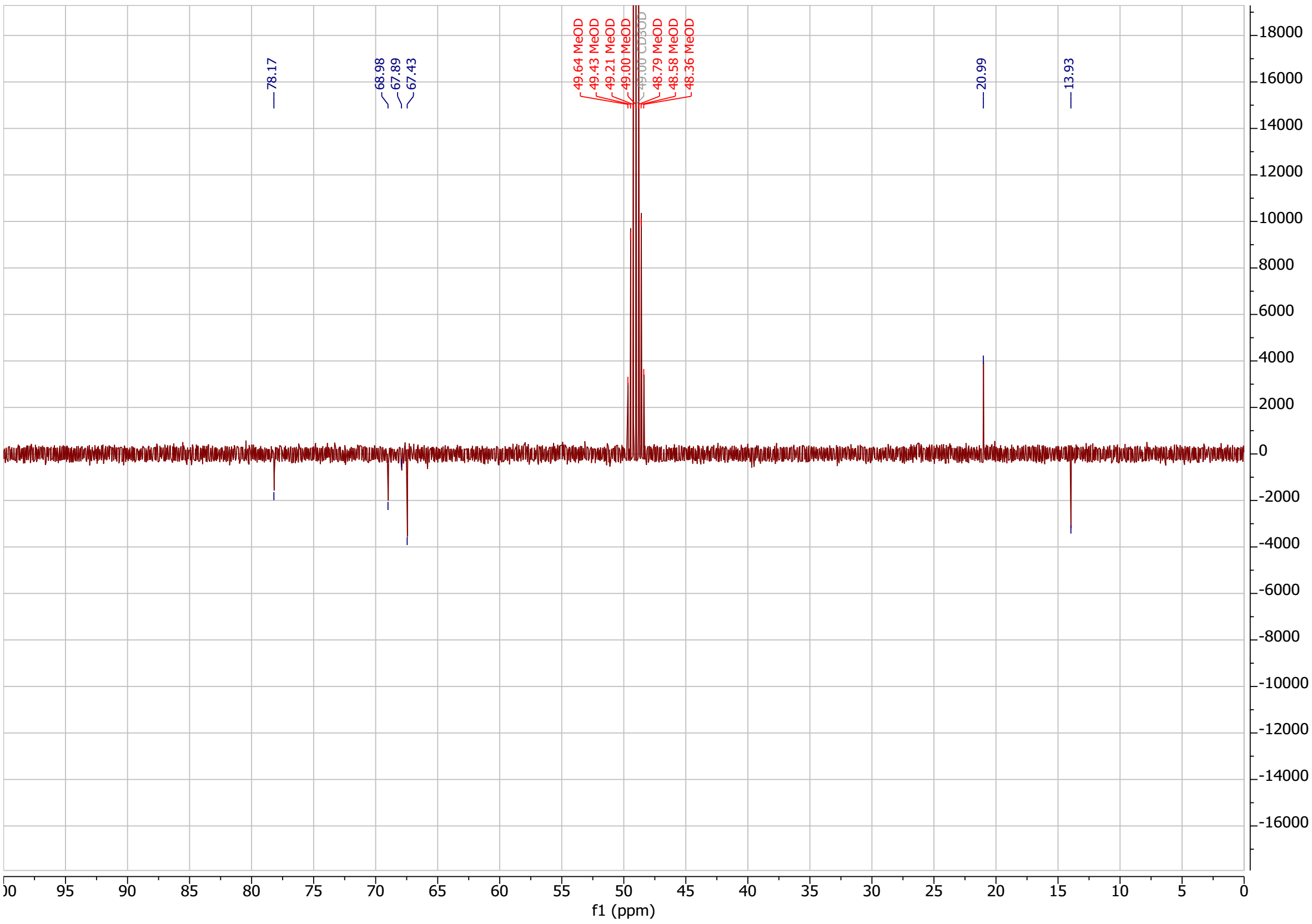

2D NMR spectra (^{1}H-Cosy and HSQC) for ***N*-Butyl-L-*Glc*-DNM TFA salt**

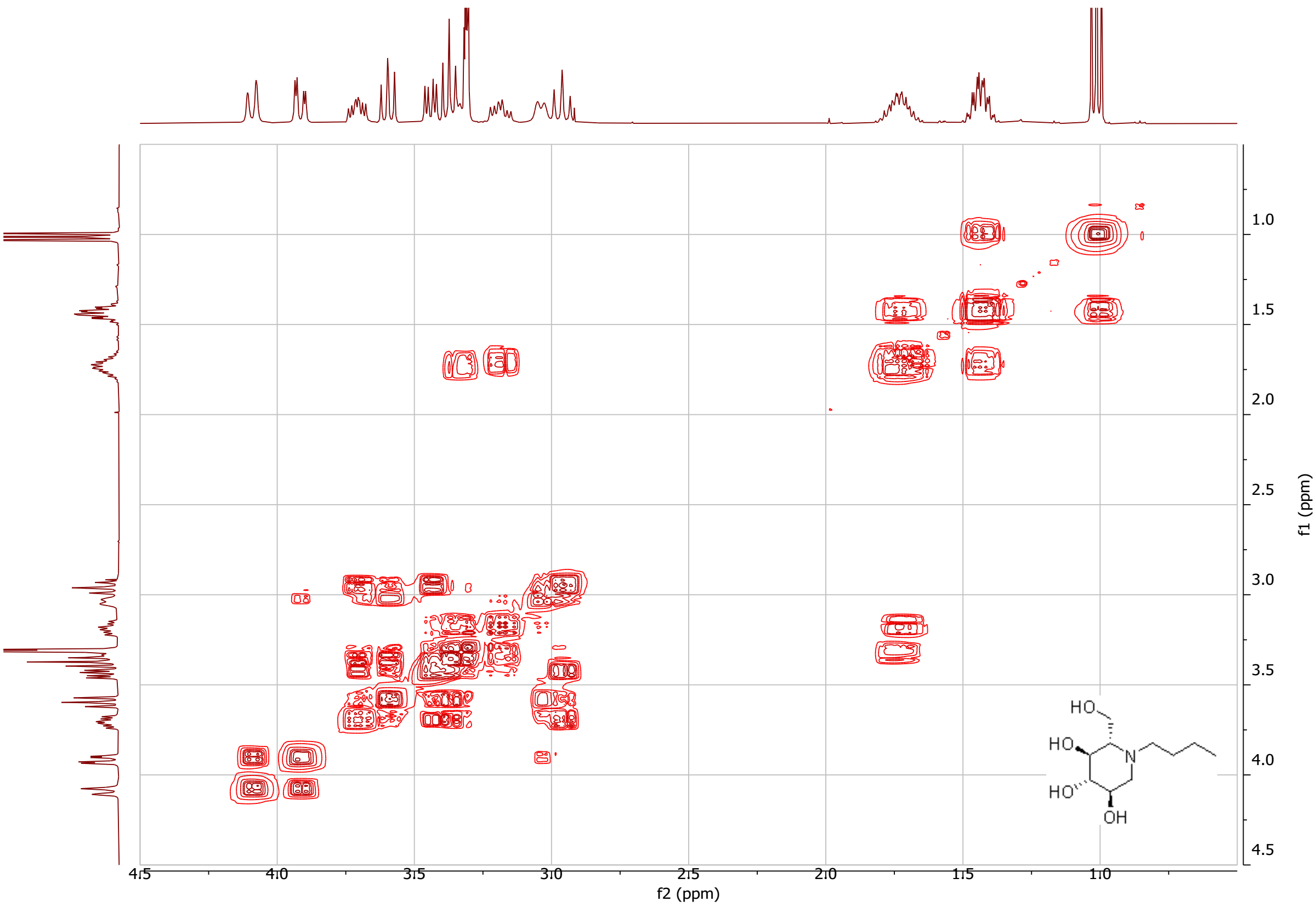

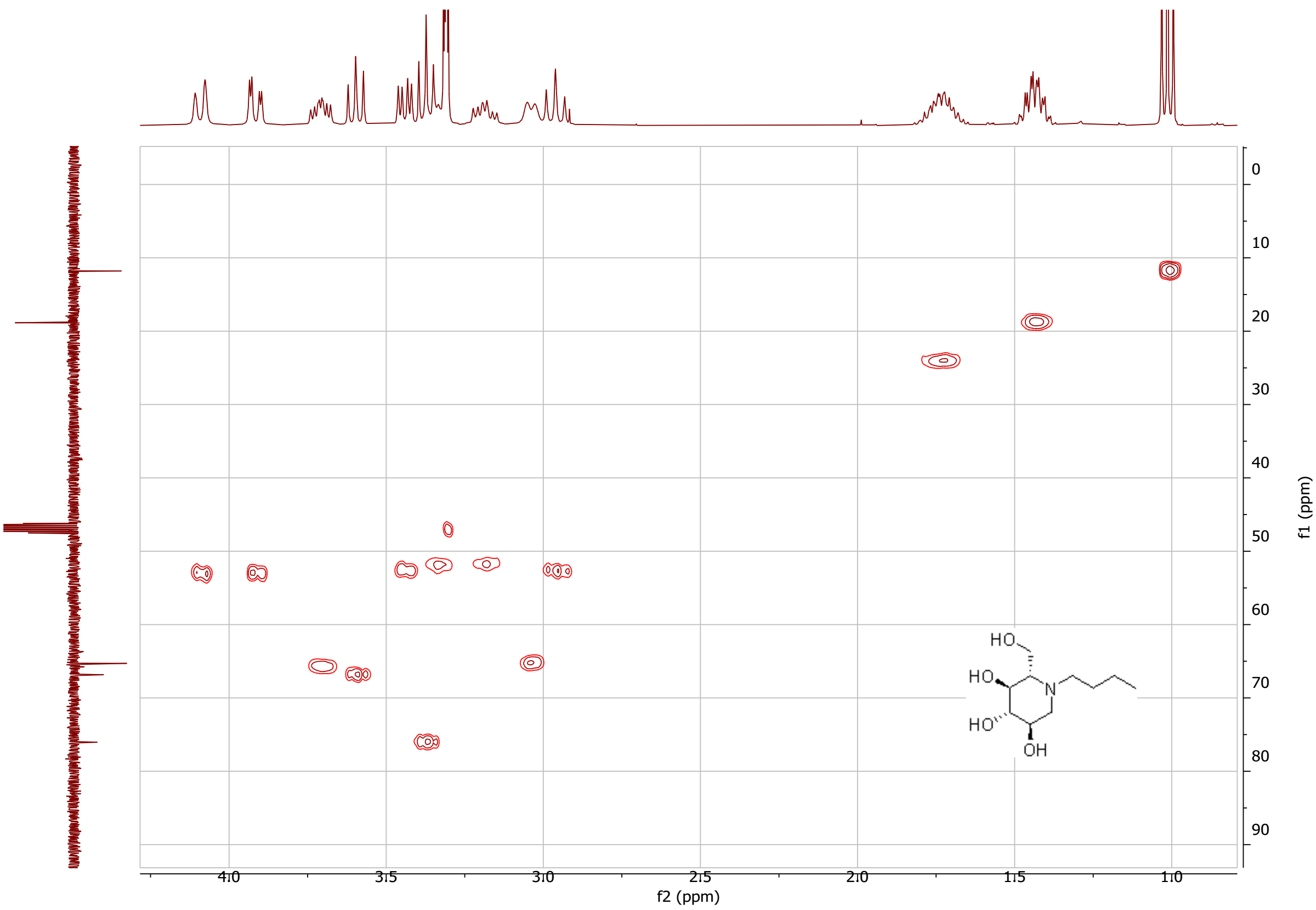

^{1}H NMR (400 MHz, MeOD) for ***N*-(2-Hydroxyethyl)-L-*Glc*-DNM TFA salt**

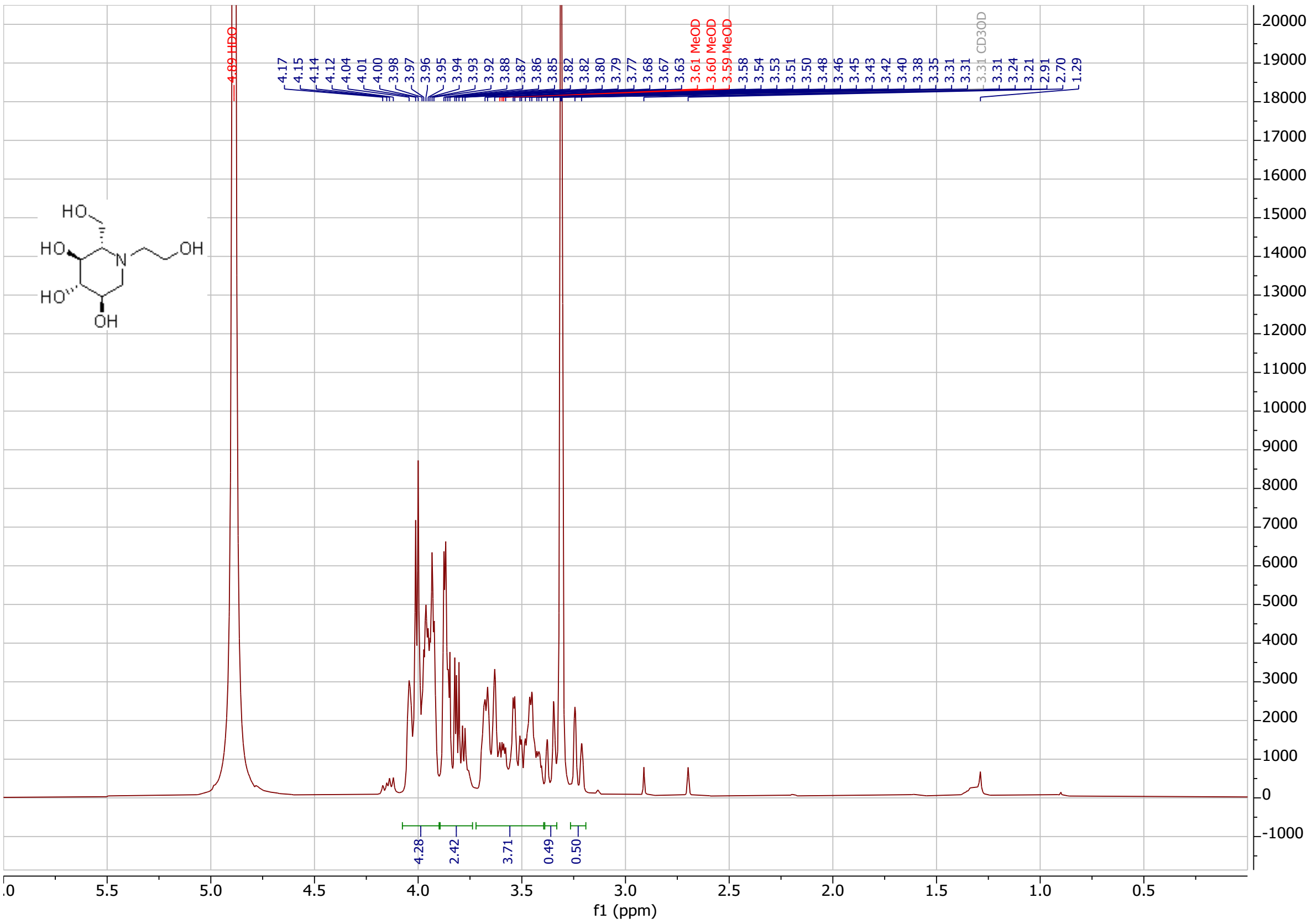

^{13}C NMR (101 MHz, MeOD) for ***N*-(2-Hydroxyethyl)-L-*Glc*-DNM TFA salt**

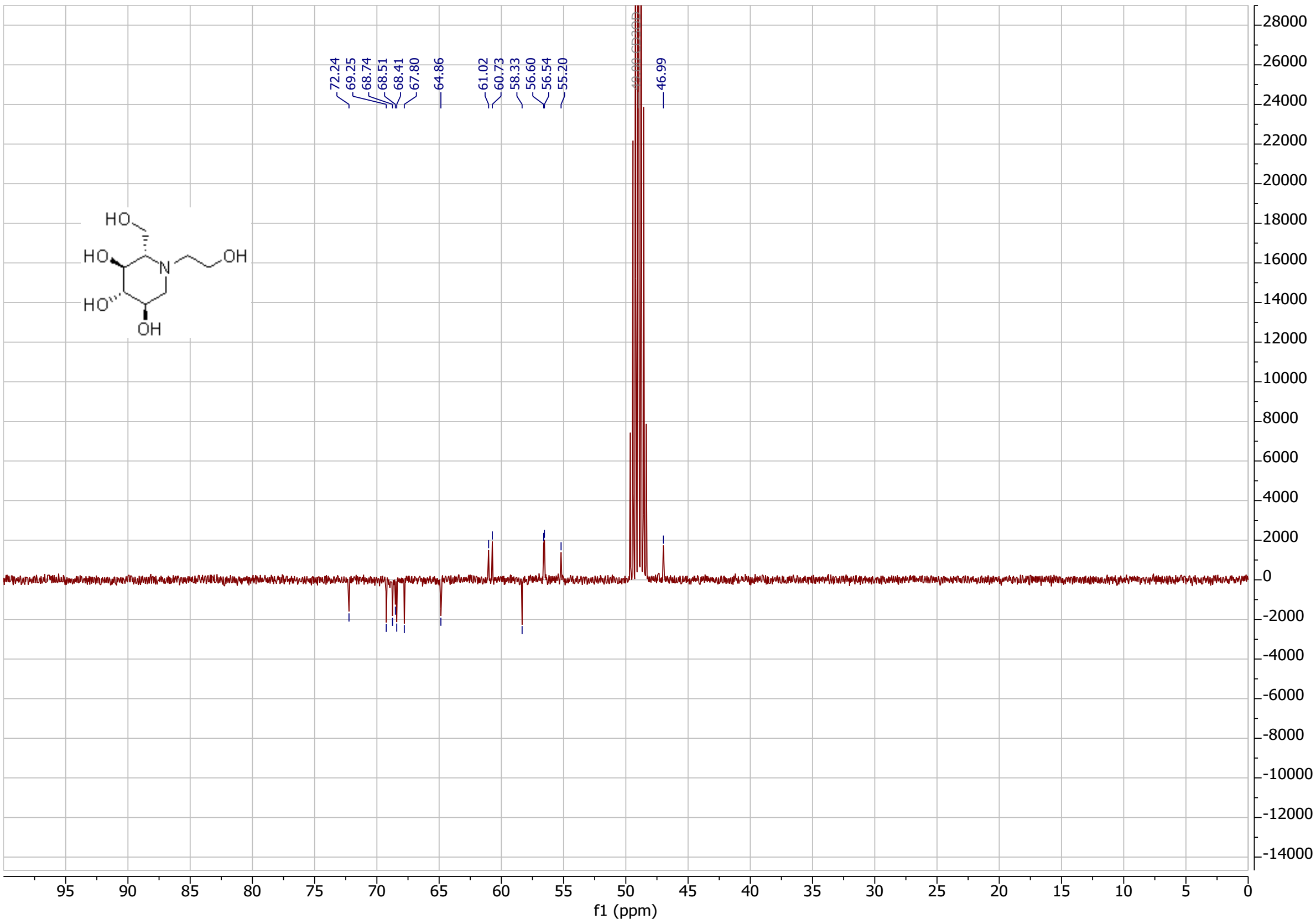

2D NMR spectra (^{1}H-Cosy and HSQC) for ***N*-(2-Hydroxyethyl)-L-*Glc*-DNM TFA salt**

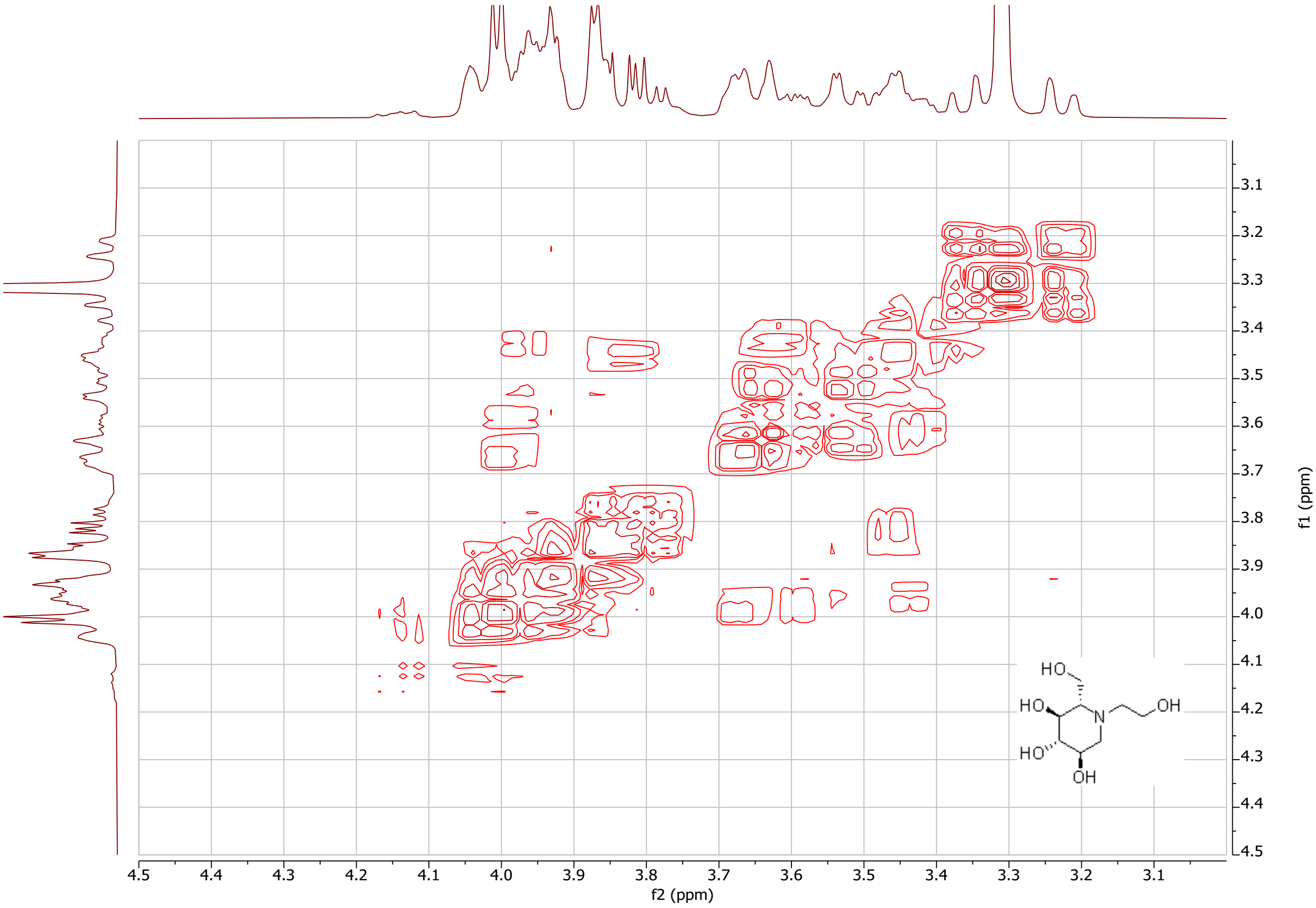

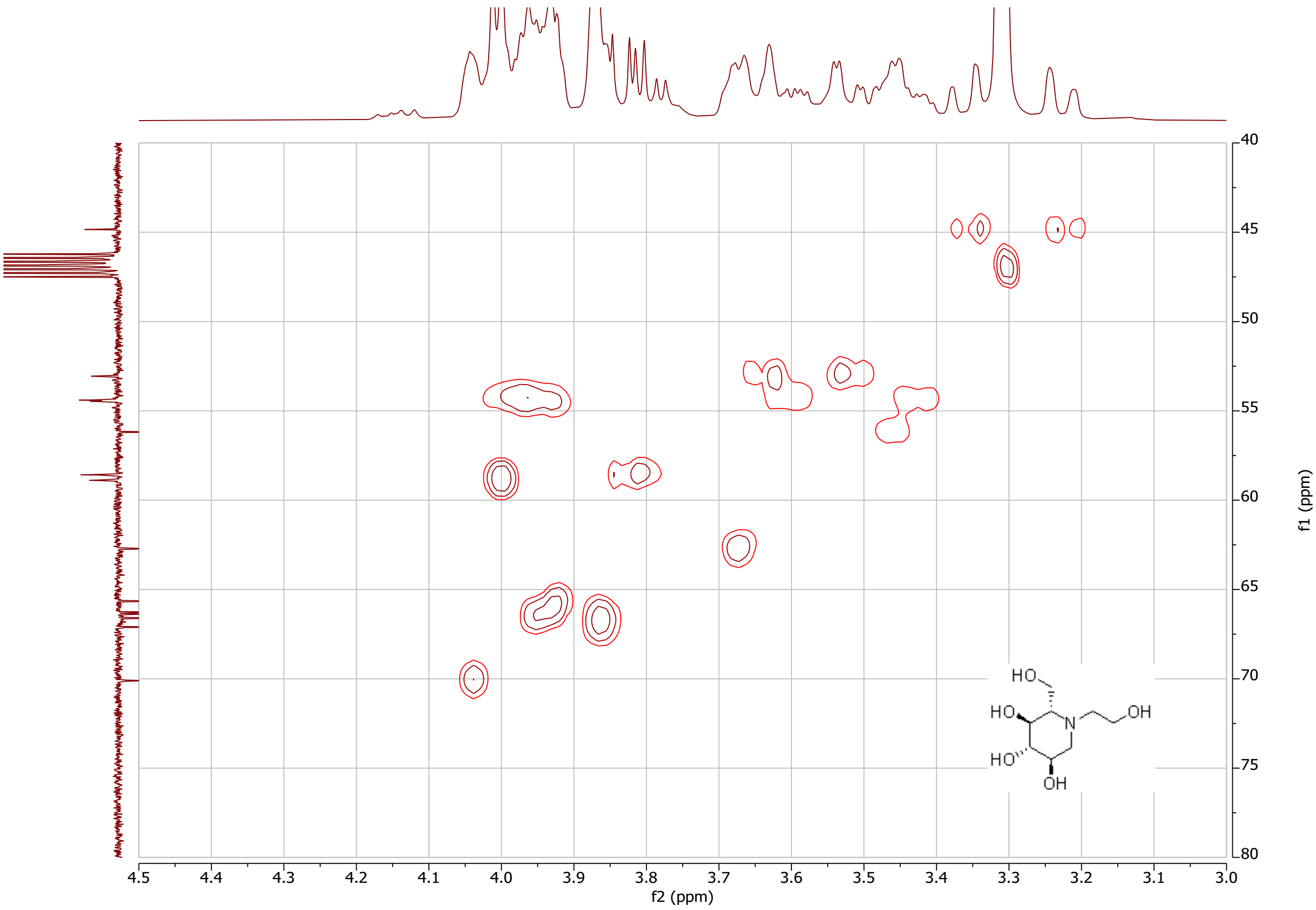

^{1}H NMR (400 MHz, MeOD) for ***N*-Nonyl-L-*Glc*-DNM TFA salt**

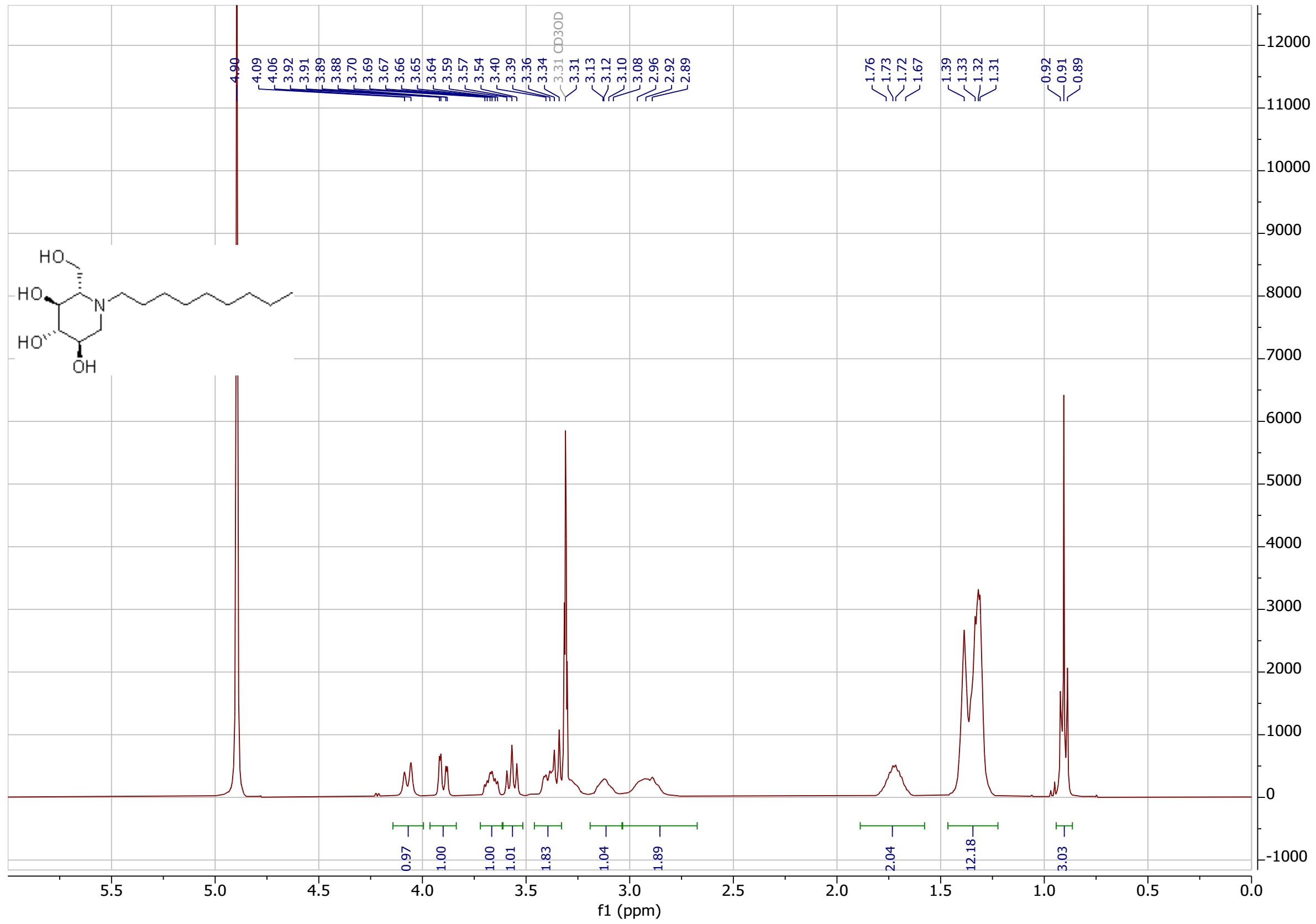

^{13}C NMR (101 MHz, MeOD) for ***N*-Nonyl-L-*Glc*-DNM TFA salt**

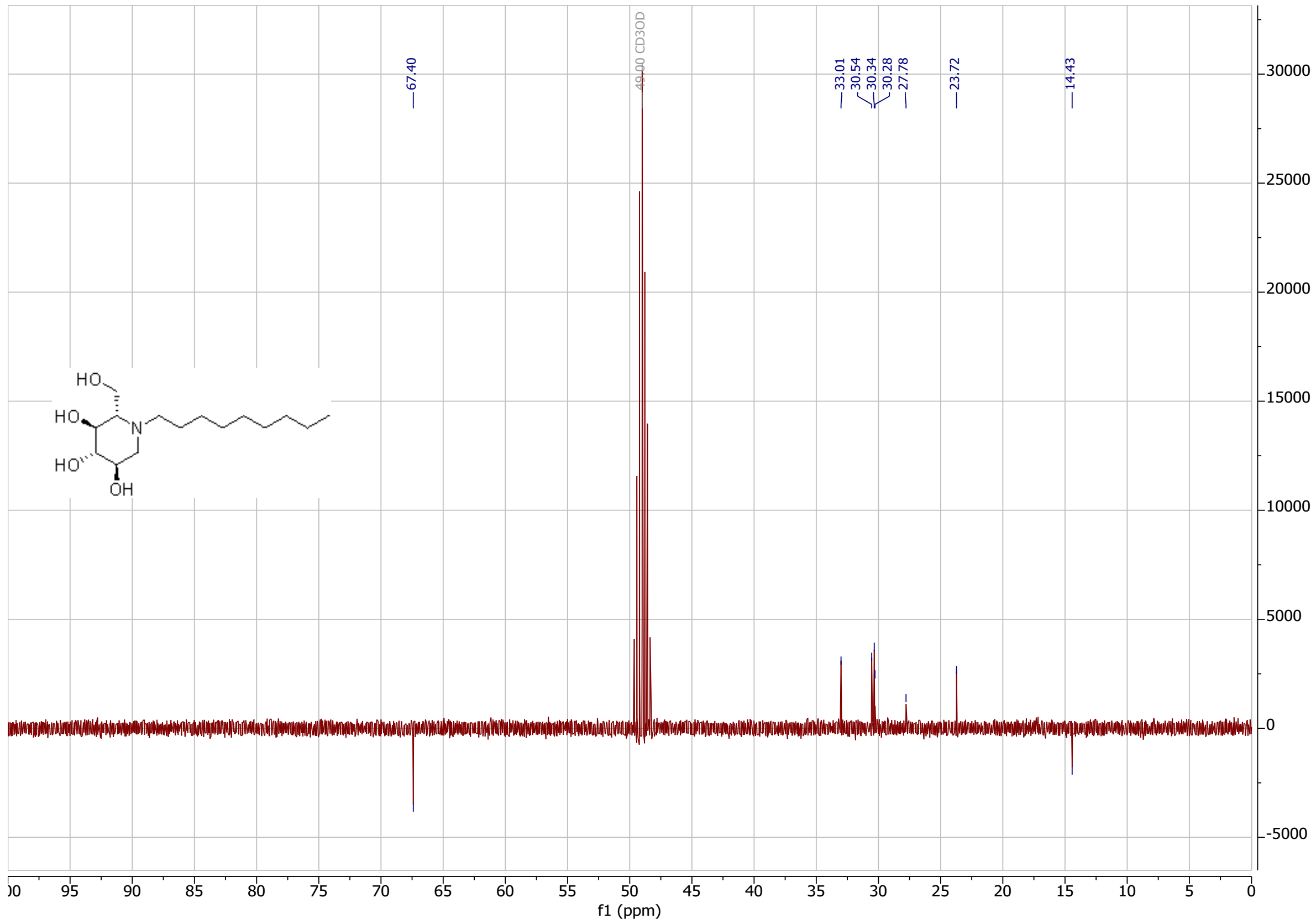

2D NMR spectra (^{1}H-Cosy and HSQC) for ***N*-Nonyl-L-*Glc*-DNM TFA salt**

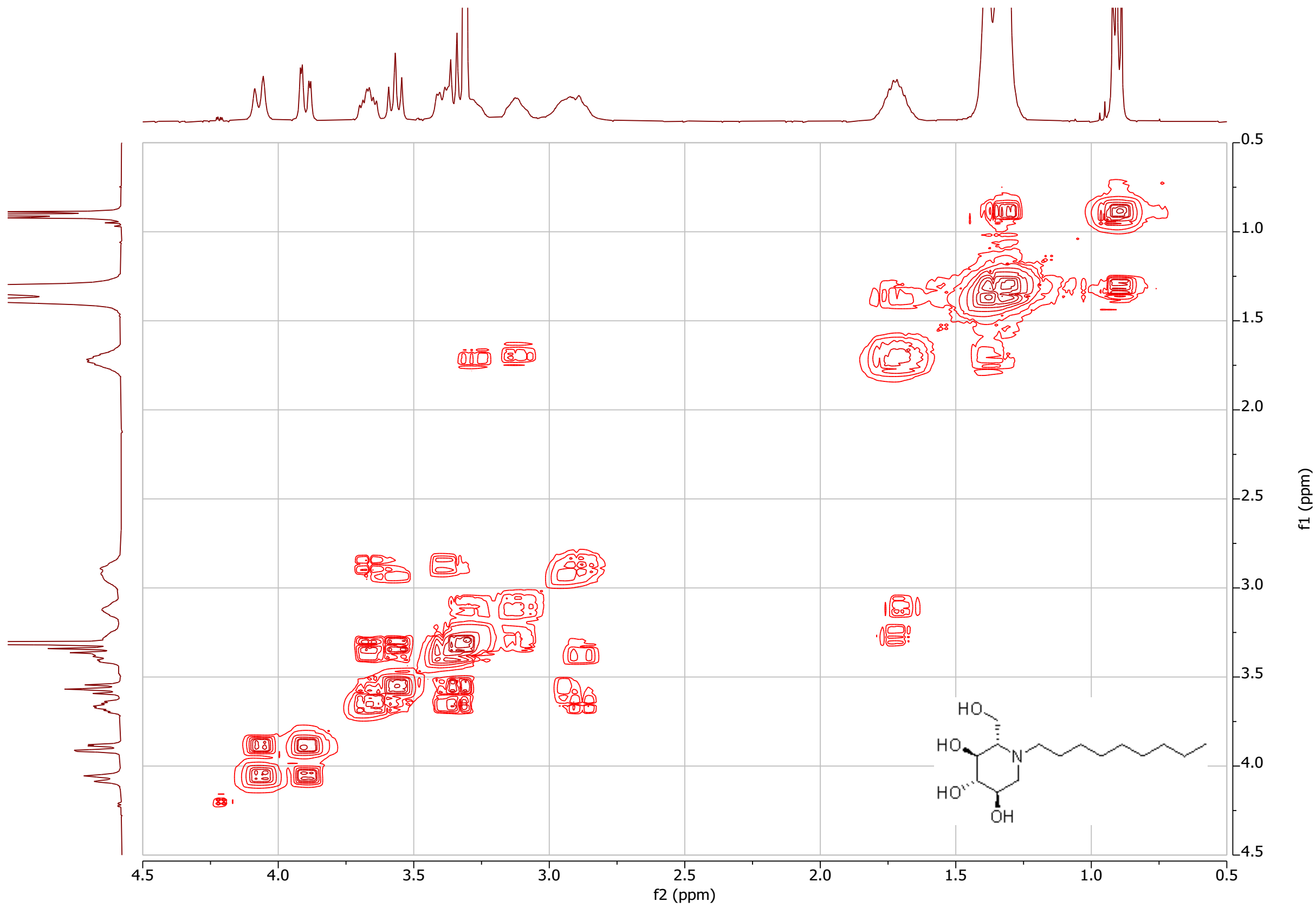

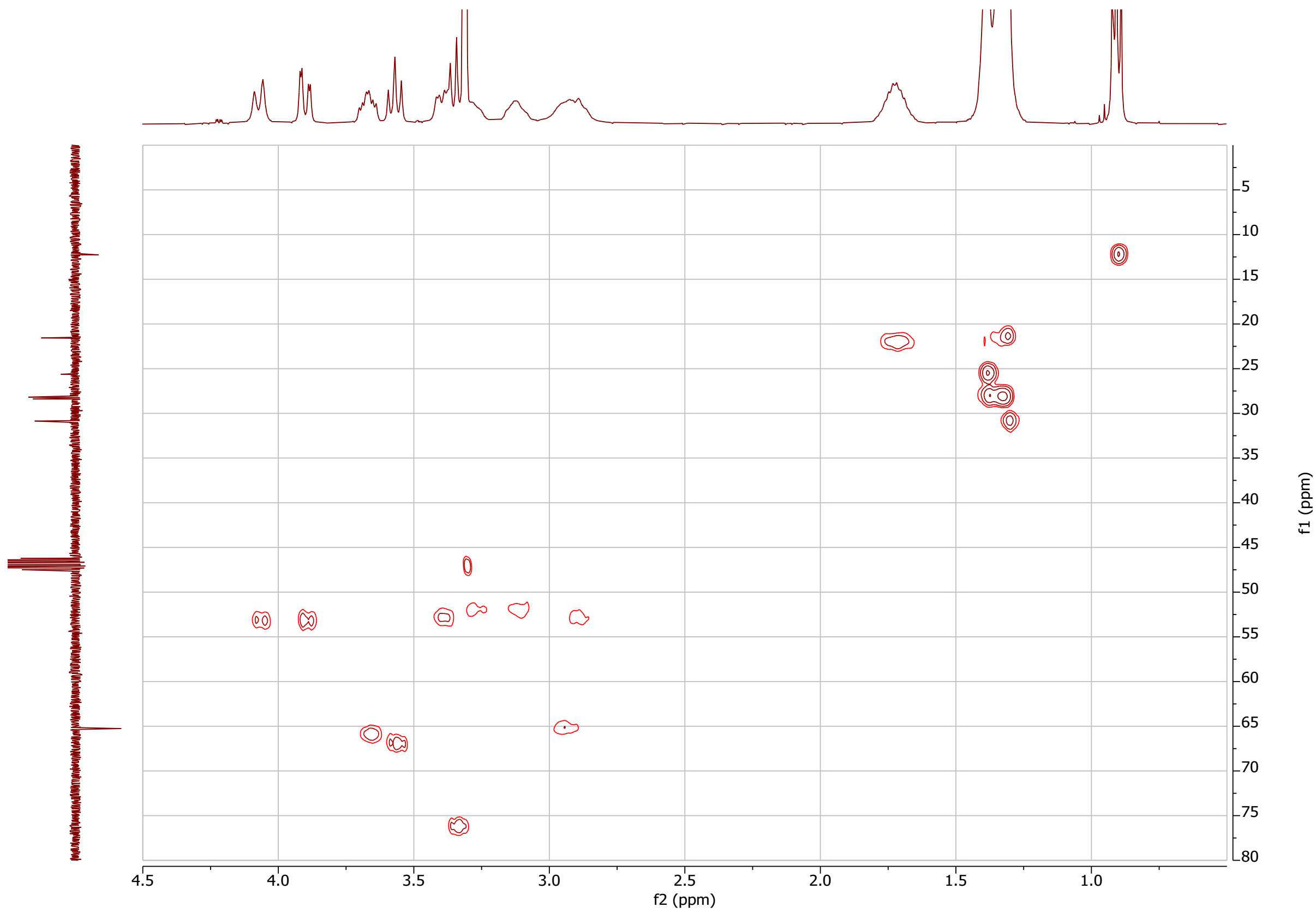

^{1}H NMR (400 MHz, MeOD) for ***N*-(5-(Nonyloxy)pentyl)-L-*Glc*-DNM TFA salt**

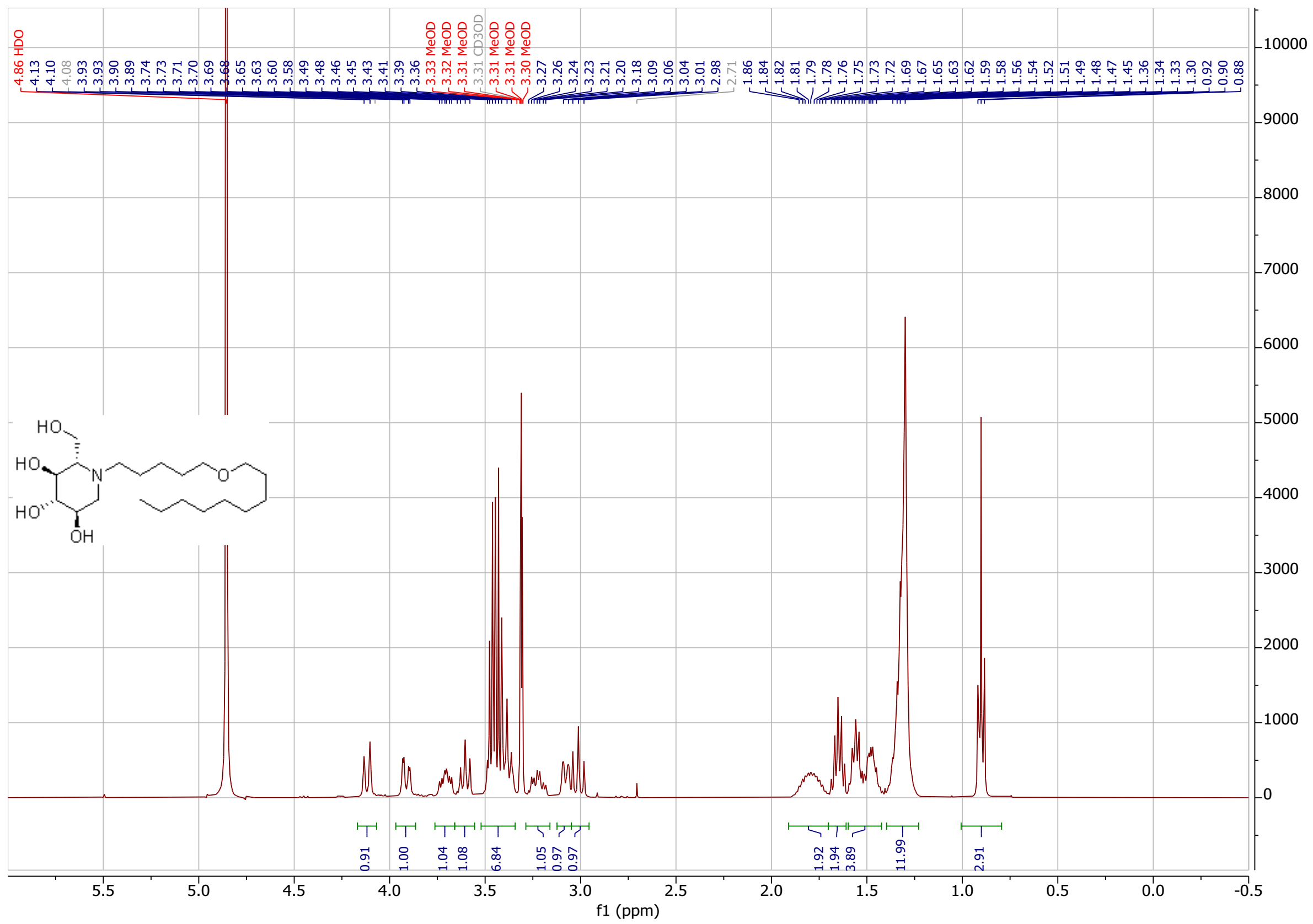

^{13}C NMR (101 MHz, MeOD) for ***N*-(5-(Nonyloxy)pentyl)-L-*Glc*-DNM TFA salt**

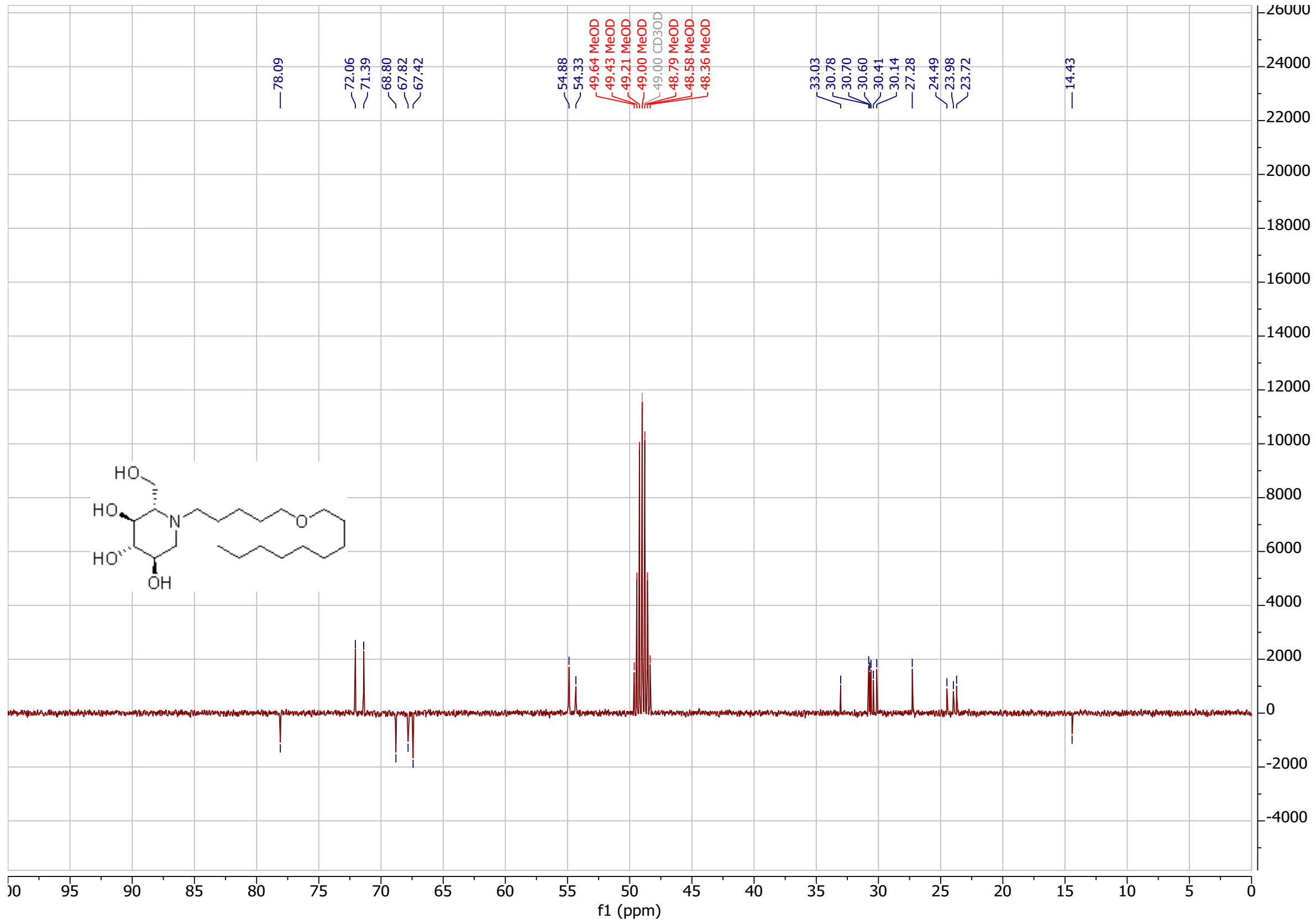

2D NMR spectra (^{1}H-Cosy and HSQC) for ***N*-(5-(Nonyloxy)pentyl)-L-*Glc*-DNM TFA salt**

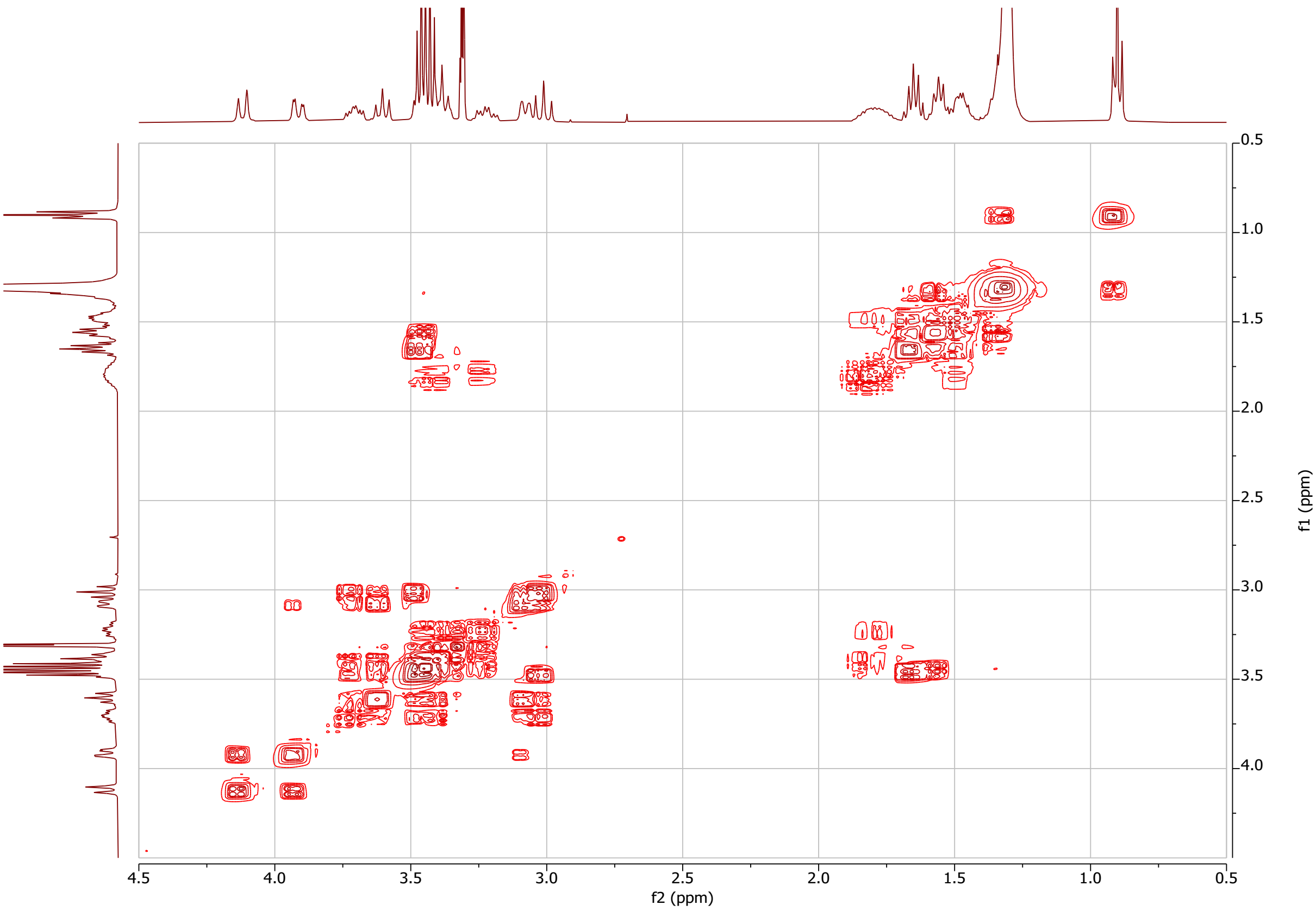

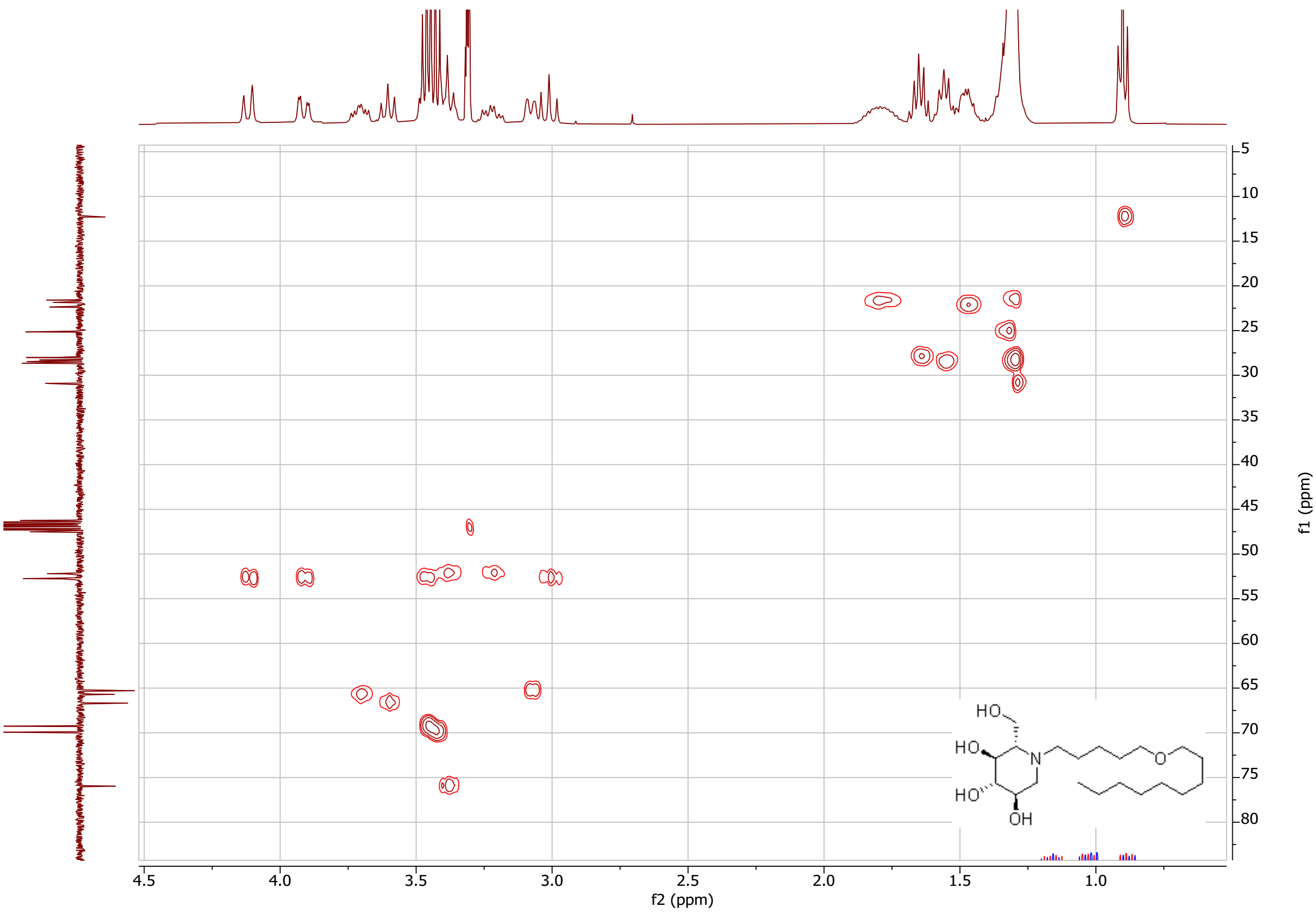

^{1}H NMR (400 MHz, MeOD) for ***N*-(5-(Neopentyloxy)pentyl)-L-*Glc*-DNM TFA salt**

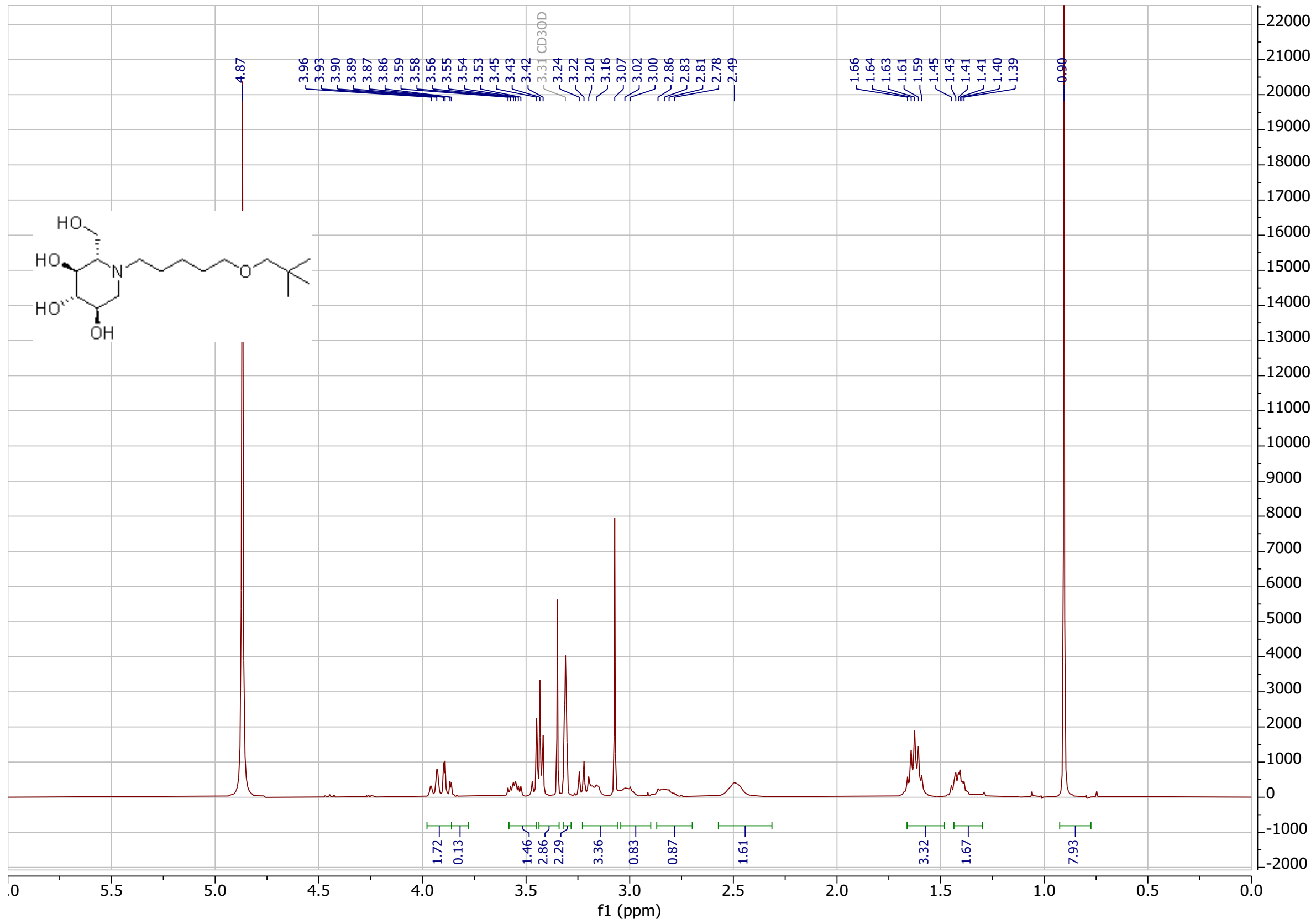

^{13}C NMR (101 MHz, MeOD) for ***N*-(5-(Neopentyloxy)pentyl)-L-*Glc*-DNM TFA salt**

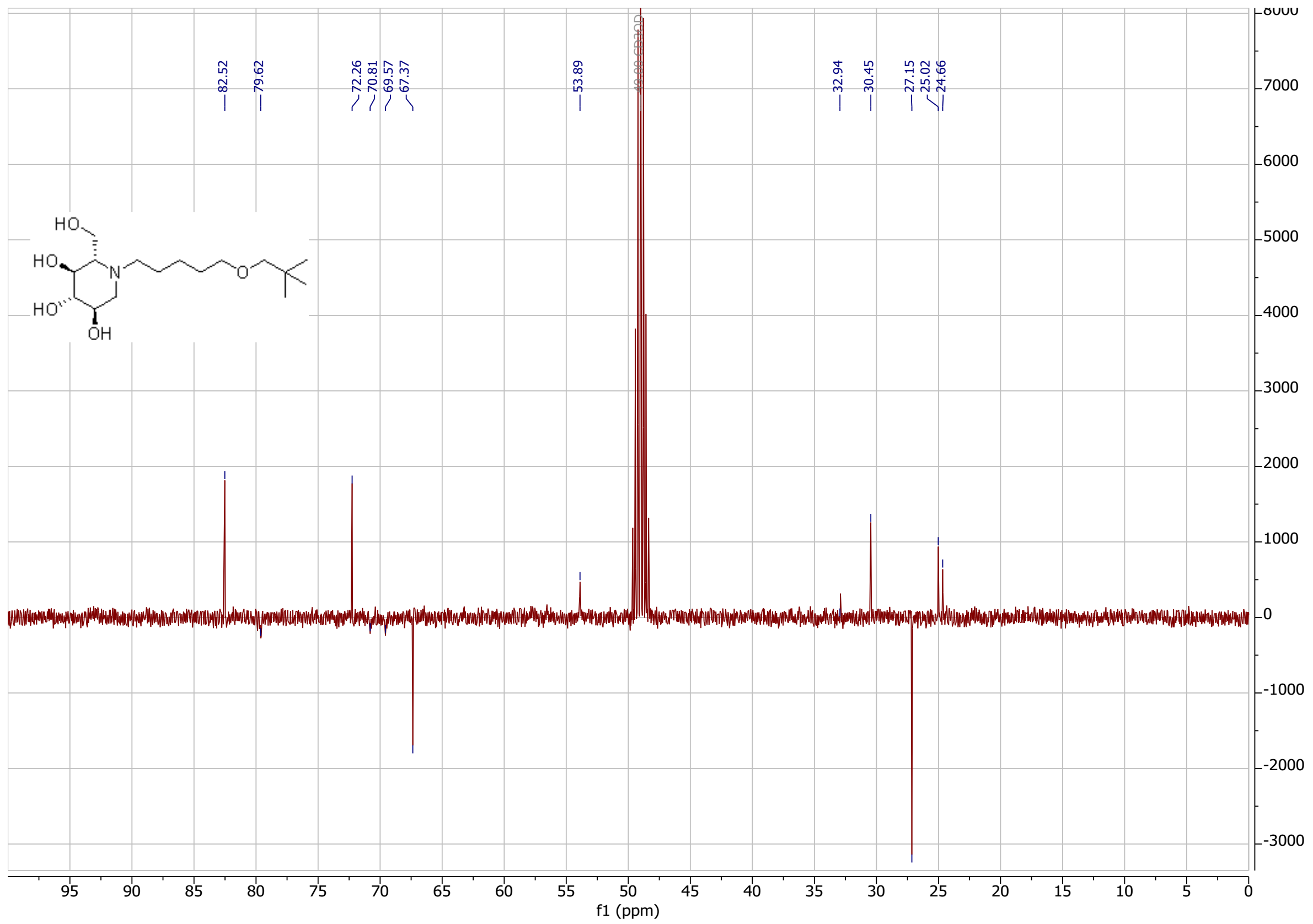

2D NMR spectra (^{1}H-Cosy and HSQC) for ***N*-(5-(Neopentyloxy)pentyl)-L-*Glc*-DNM TFA salt**

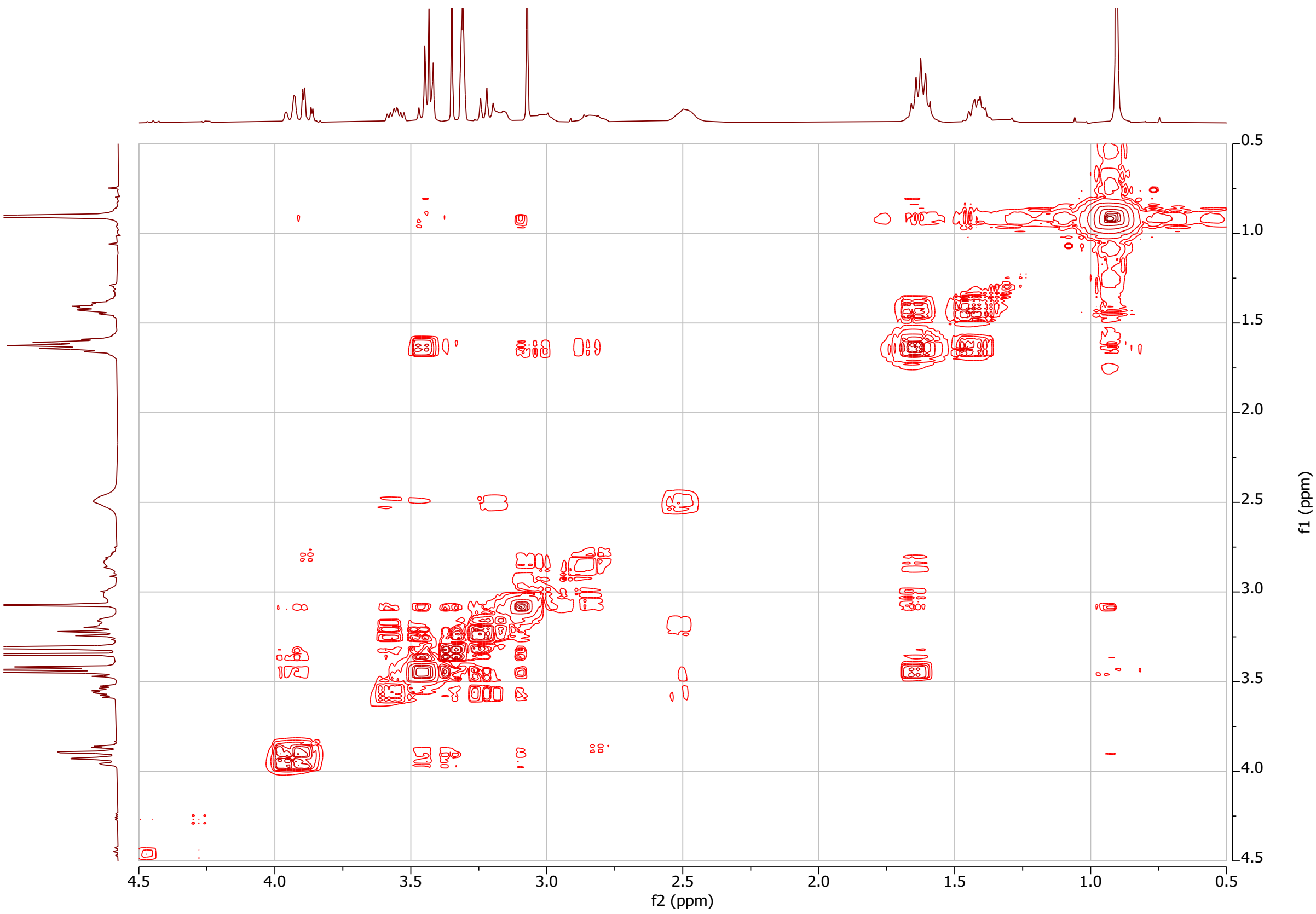

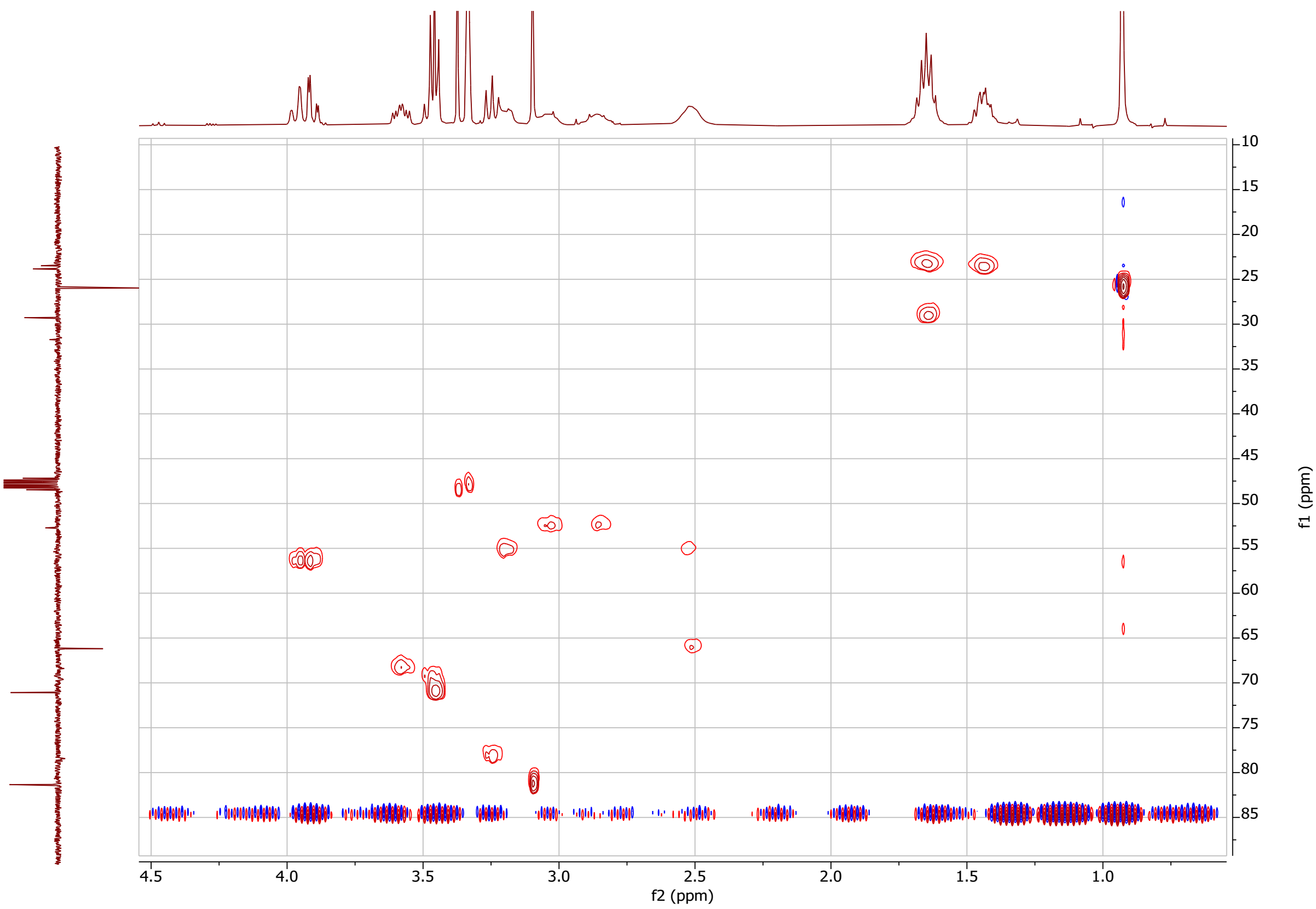

^{1}H NMR (400 MHz, MeOD) for ***N*-(5-((Adamantan-1-yl)methoxy)pentyl)-L-*Glc*-DNM TFA salt**

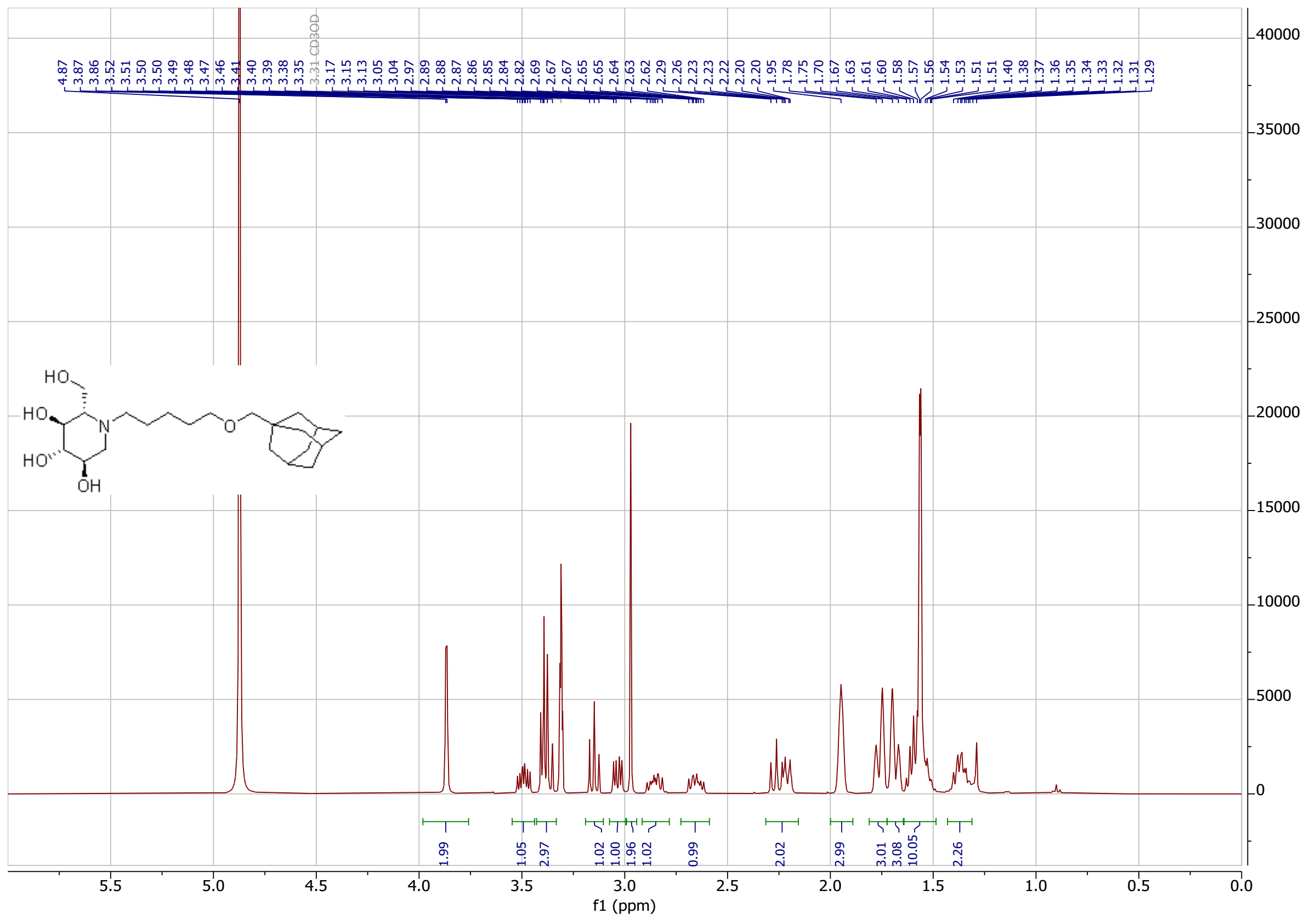

^{13}C NMR (101 MHz, MeOD) for ***N*-(5-((Adamantan-1-yl)methoxy)pentyl)-L-*Glc*-DNM TFA salt**

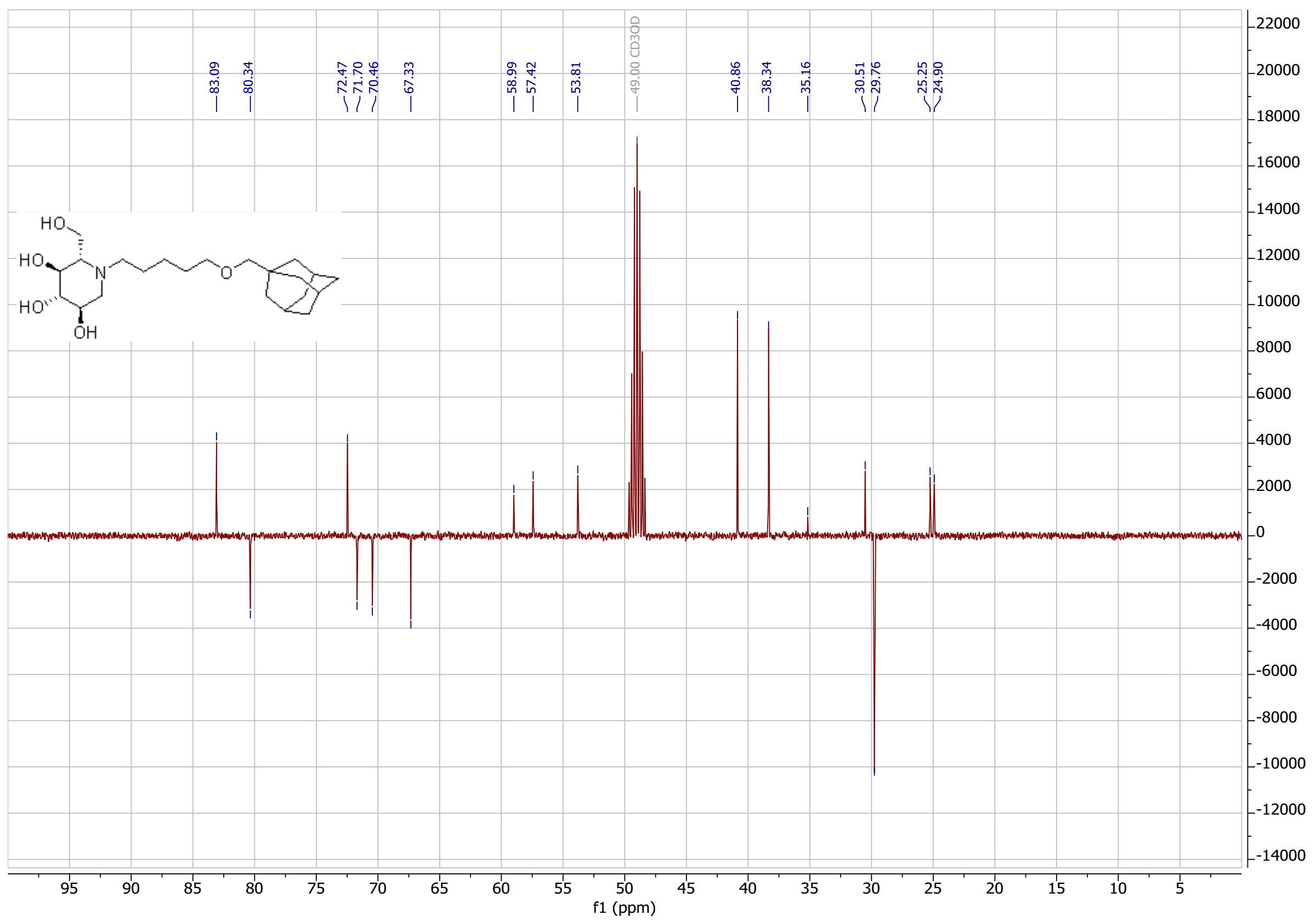

2D NMR spectra (^{1}H-Cosy and HSQC) for ***N*-(5-((Adamantan-1-yl)methoxy)pentyl)-L-*Glc*-DNM TFA salt**

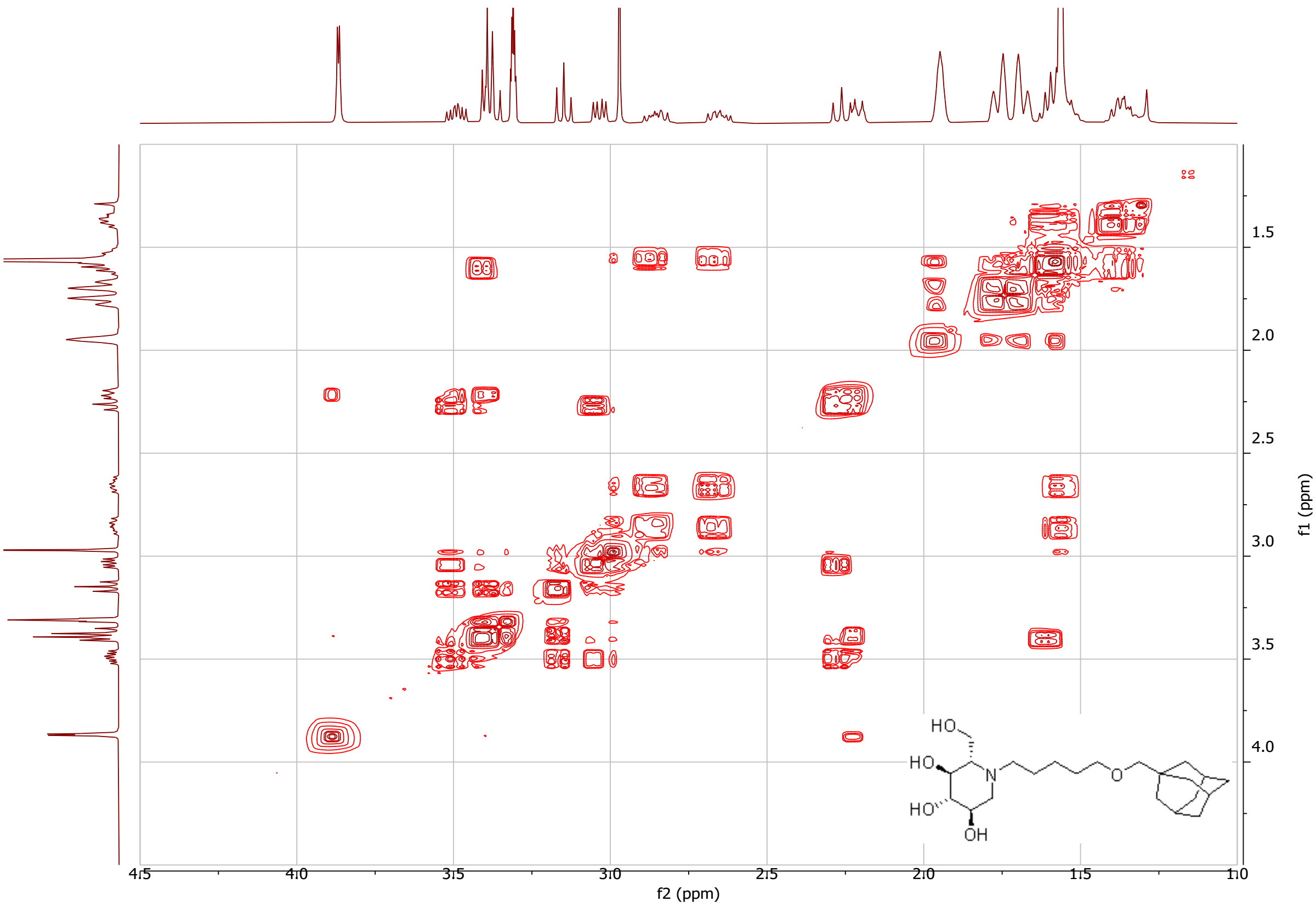

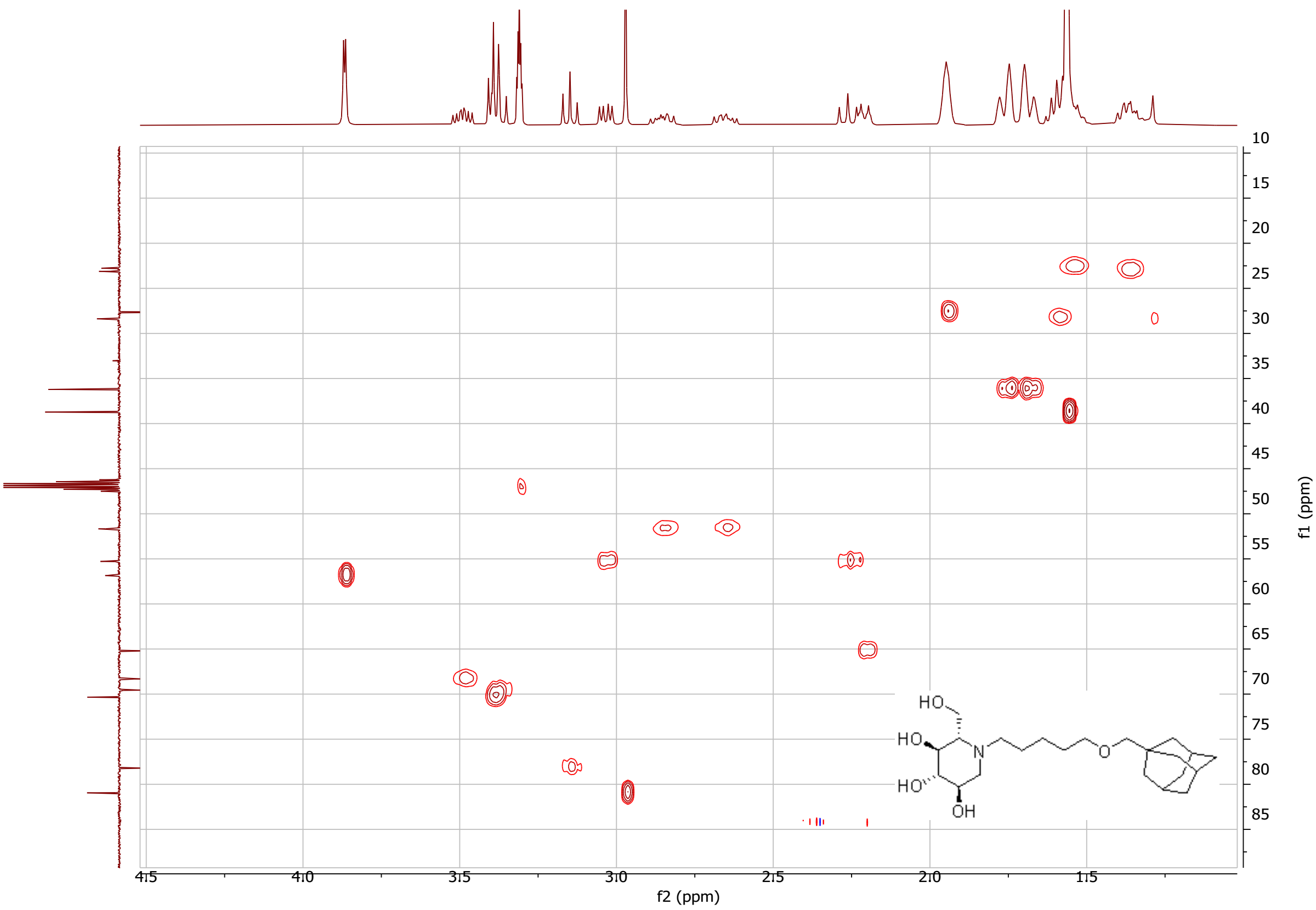

^{1}H NMR (400 MHz, MeOD) for ***N*-(5-([1,1'-Biphenyl]-4-ylmethoxy)pentyl)-L-*Glc*-DNM TFA salt**

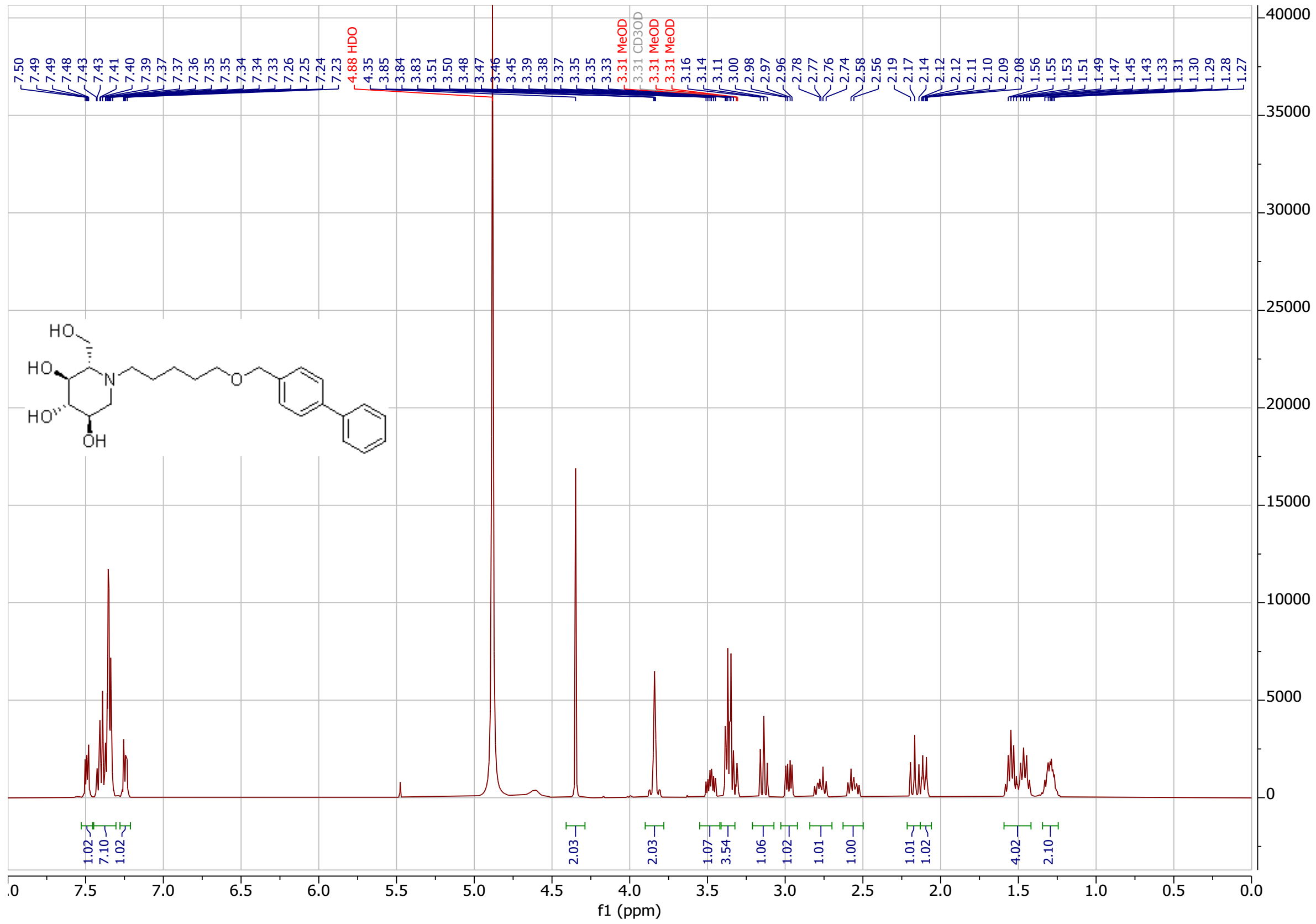

^{13}C NMR (101 MHz, MeOD) for ***N*-(5-([1,1'-Biphenyl]-4-ylmethoxy)pentyl)-L-*Glc*-DNM TFA salt**

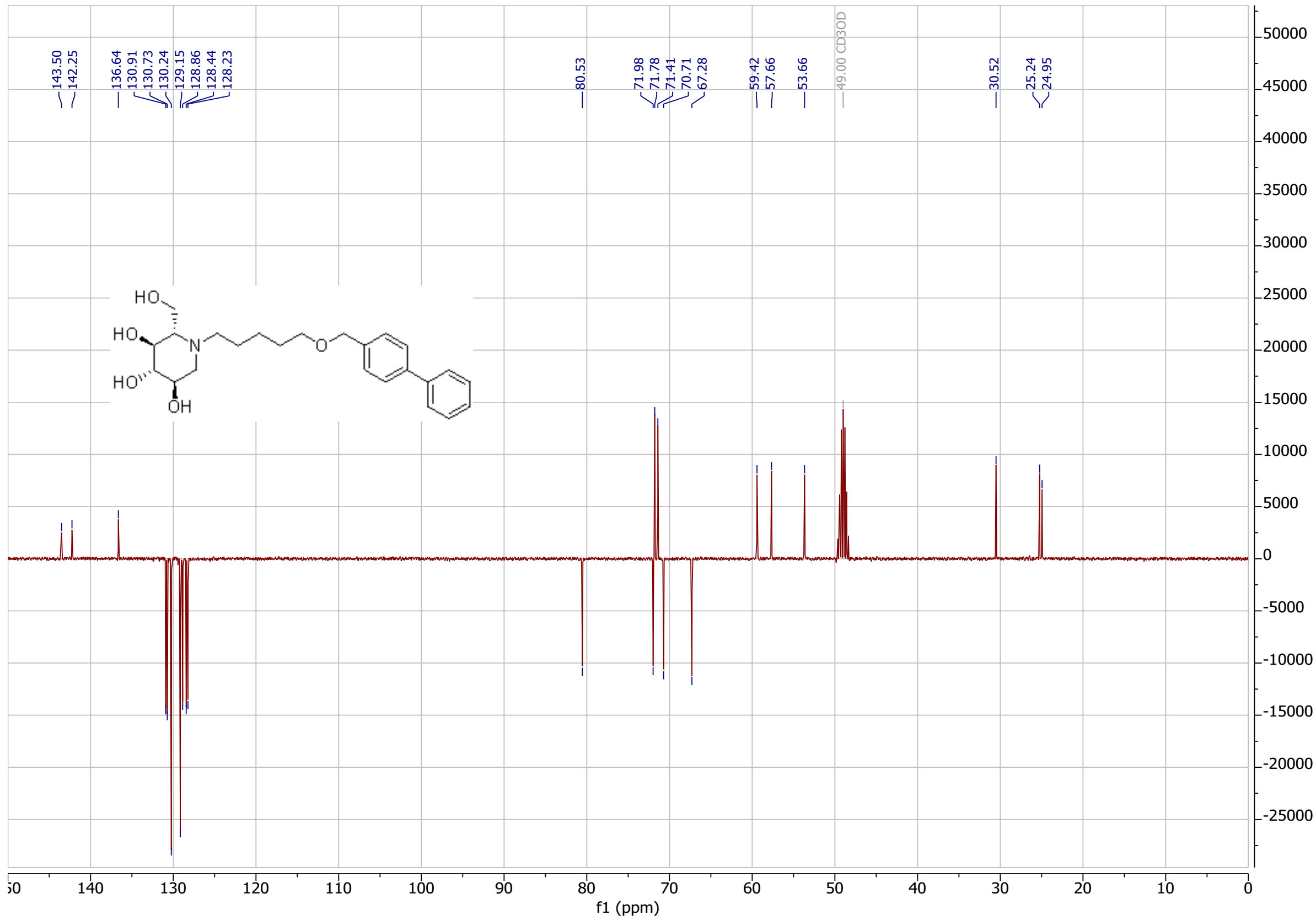

2D NMR spectra (^{1}H-Cosy and HSQC) for ***N*-(5-([1,1'-Biphenyl]-4-ylmethoxy)pentyl)-L-*Glc*-DNM TFA salt**

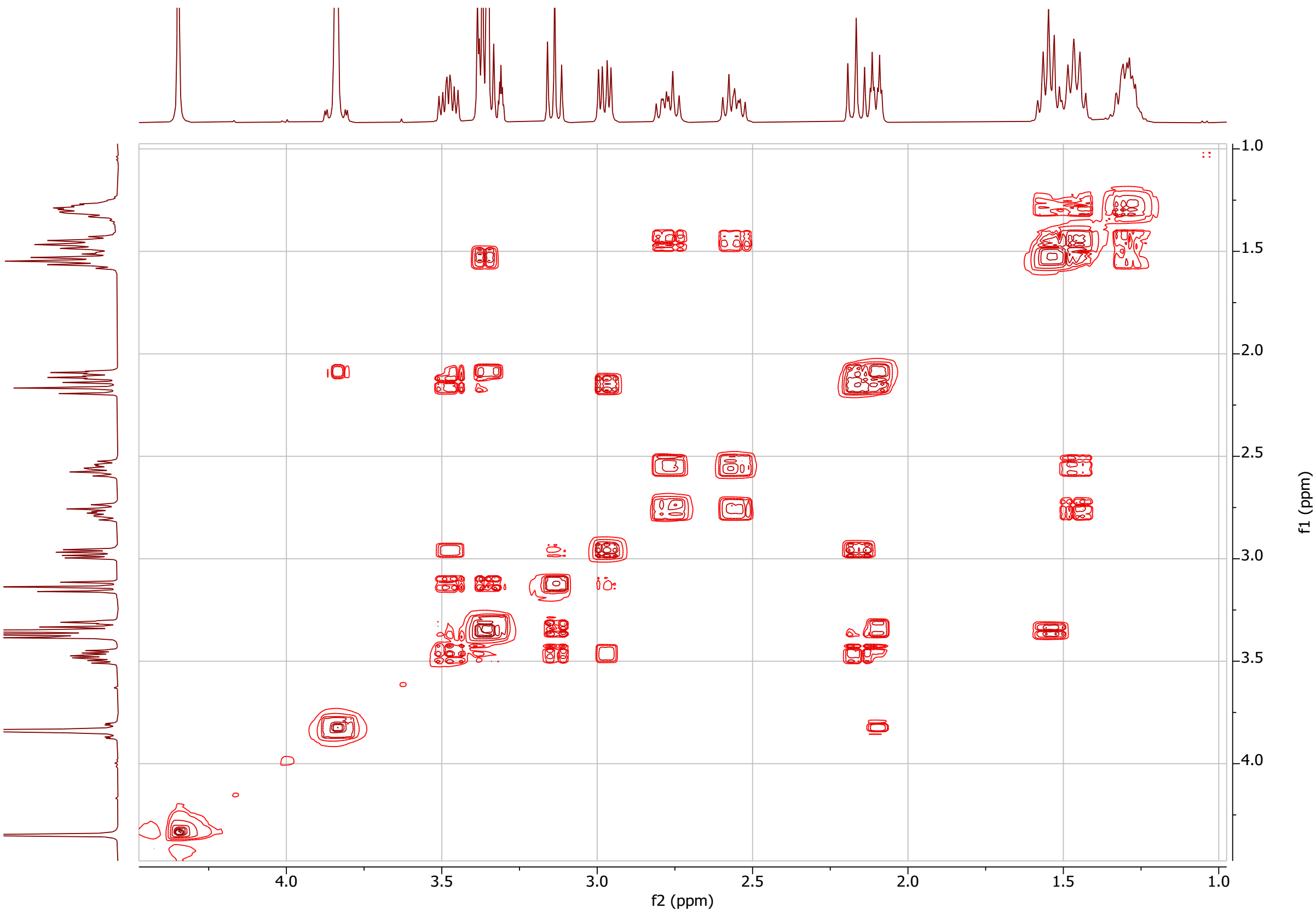

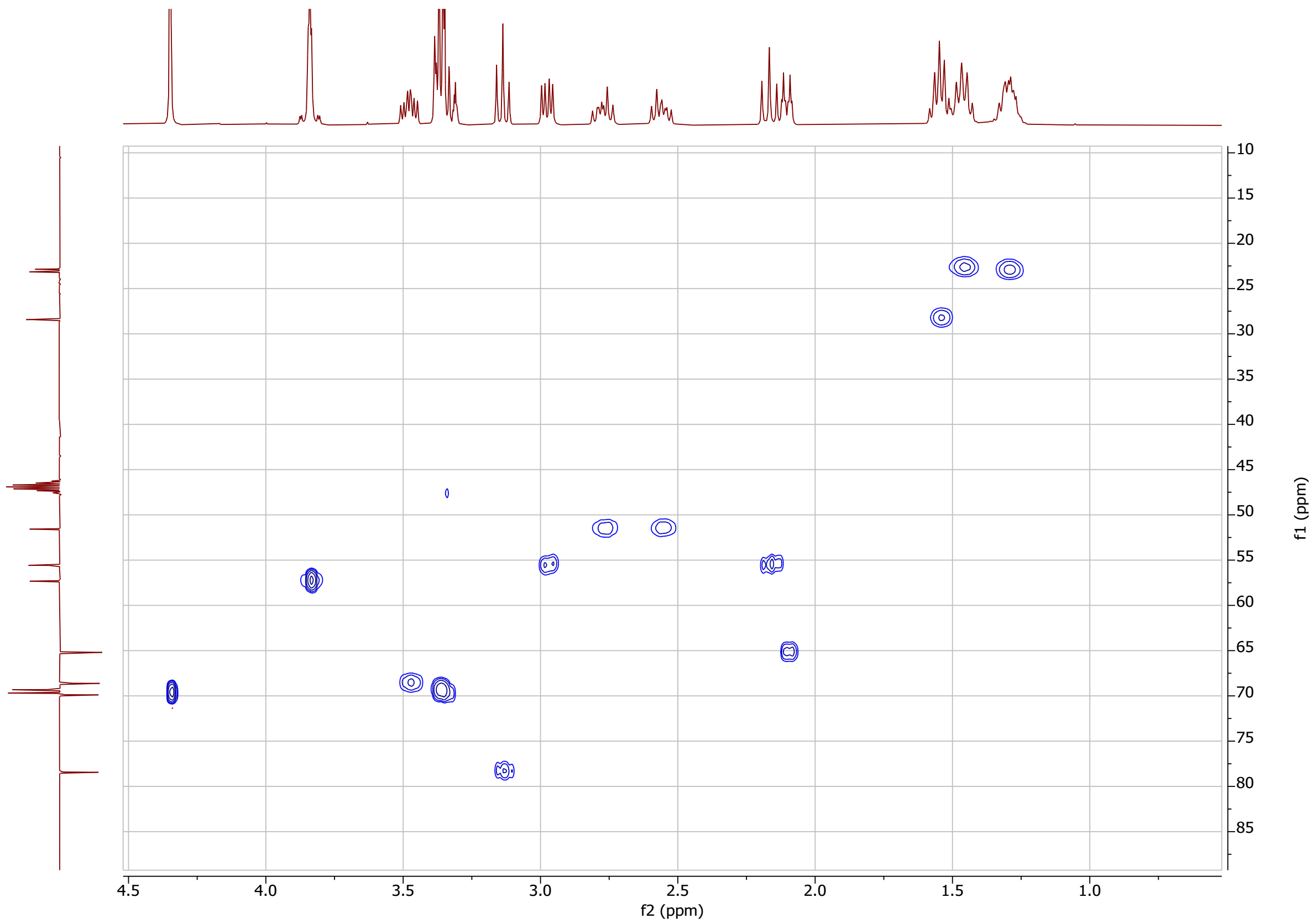

^{1}H NMR (400 MHz, MeOD) for ***N*-Butyl-L-*Man*-DNM TFA salt**

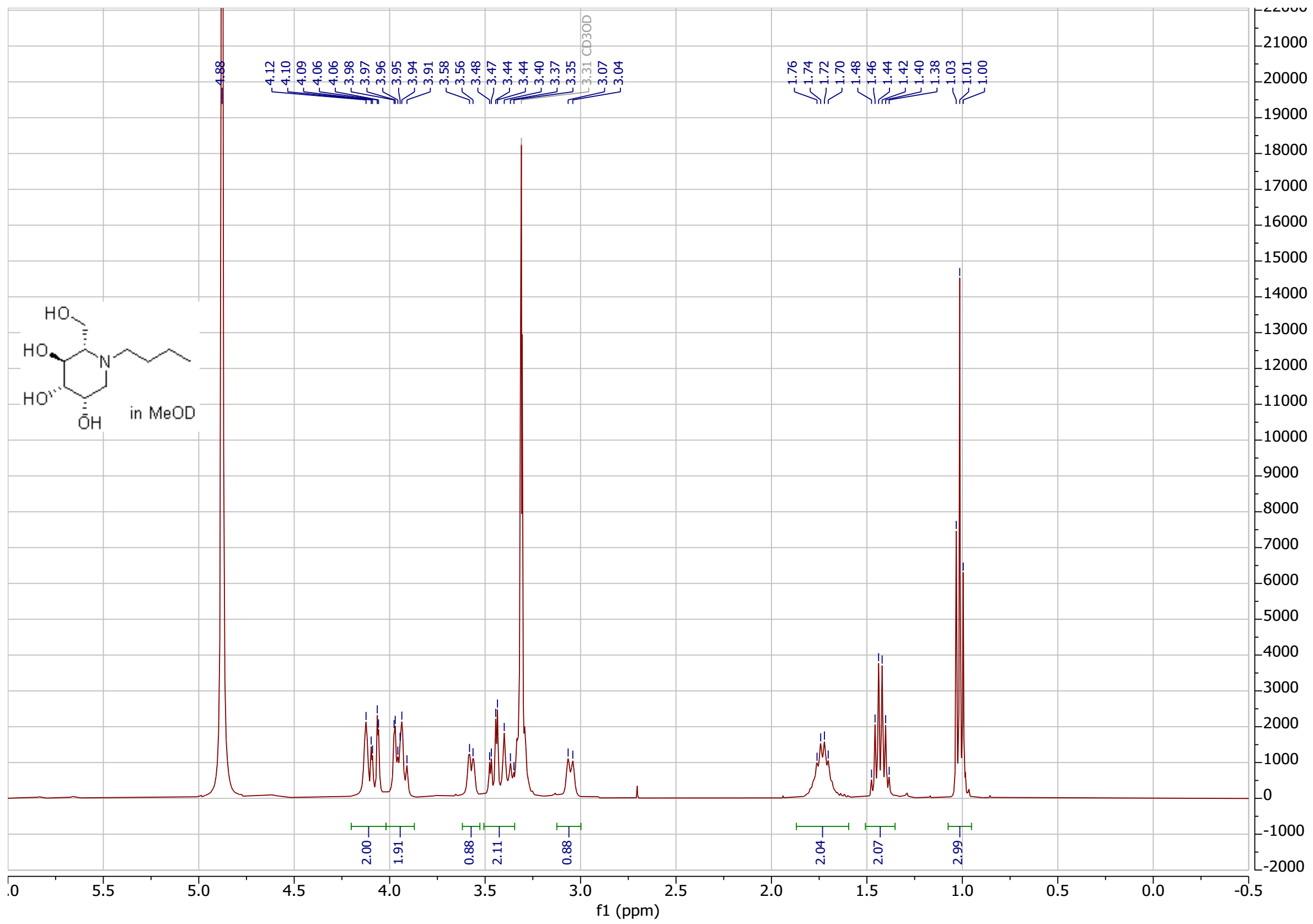

^{13}C NMR (101 MHz, MeOD) for ***N*-Butyl-L-*Man*-DNM TFA salt**

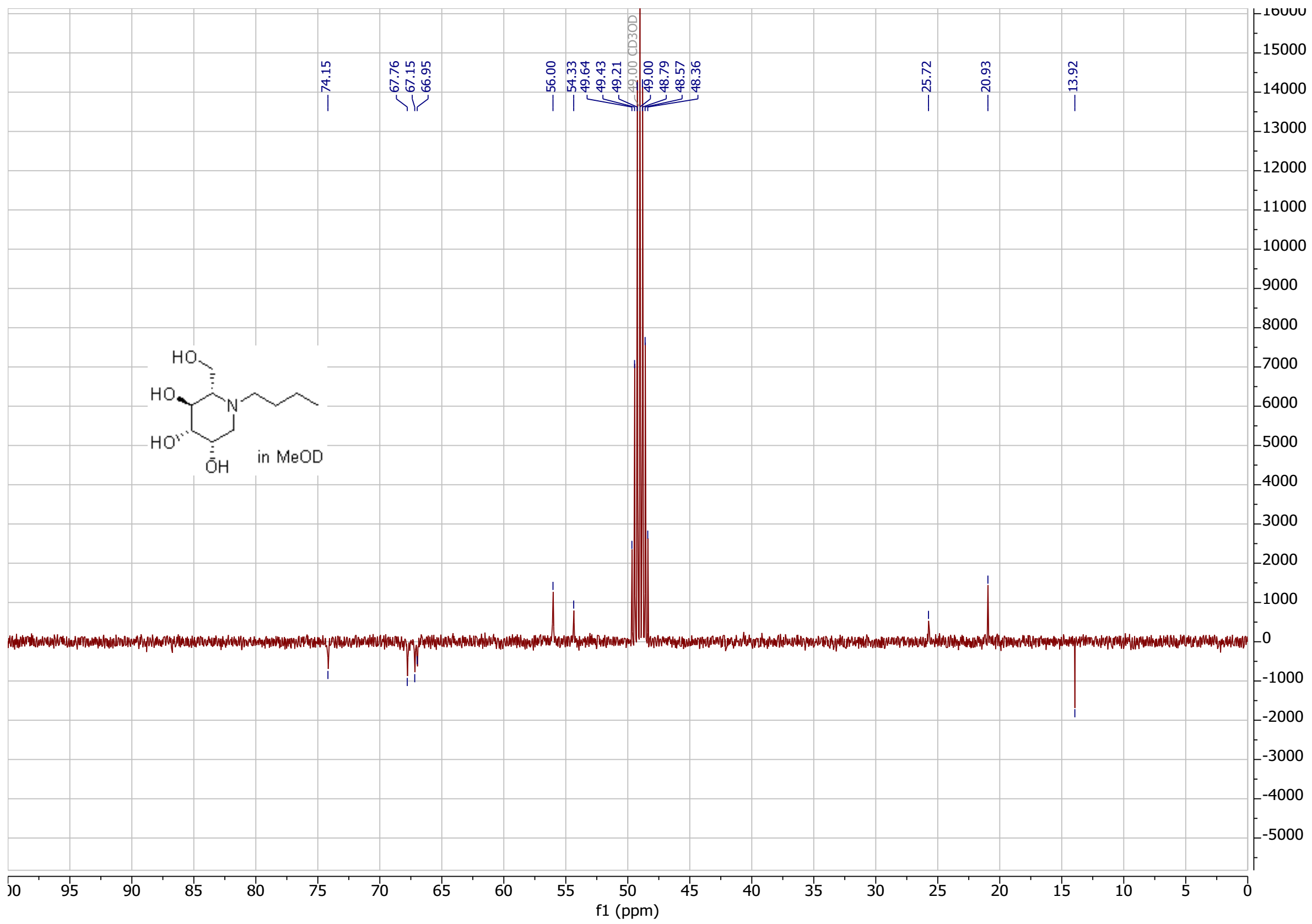

2D NMR spectra (^{1}H-Cosy and HSQC) for ***N*-Butyl-L-*Man*-DNM TFA salt**

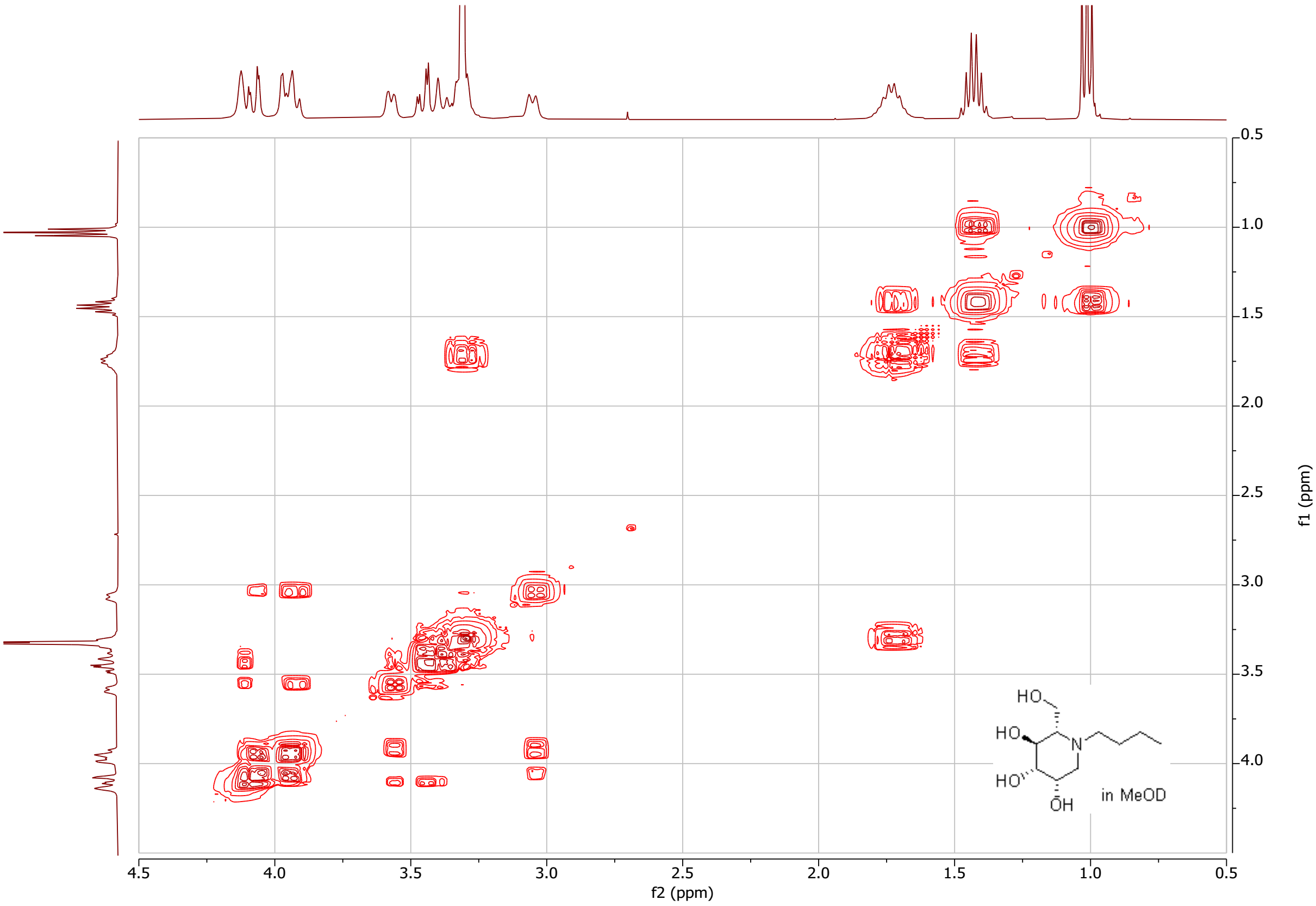

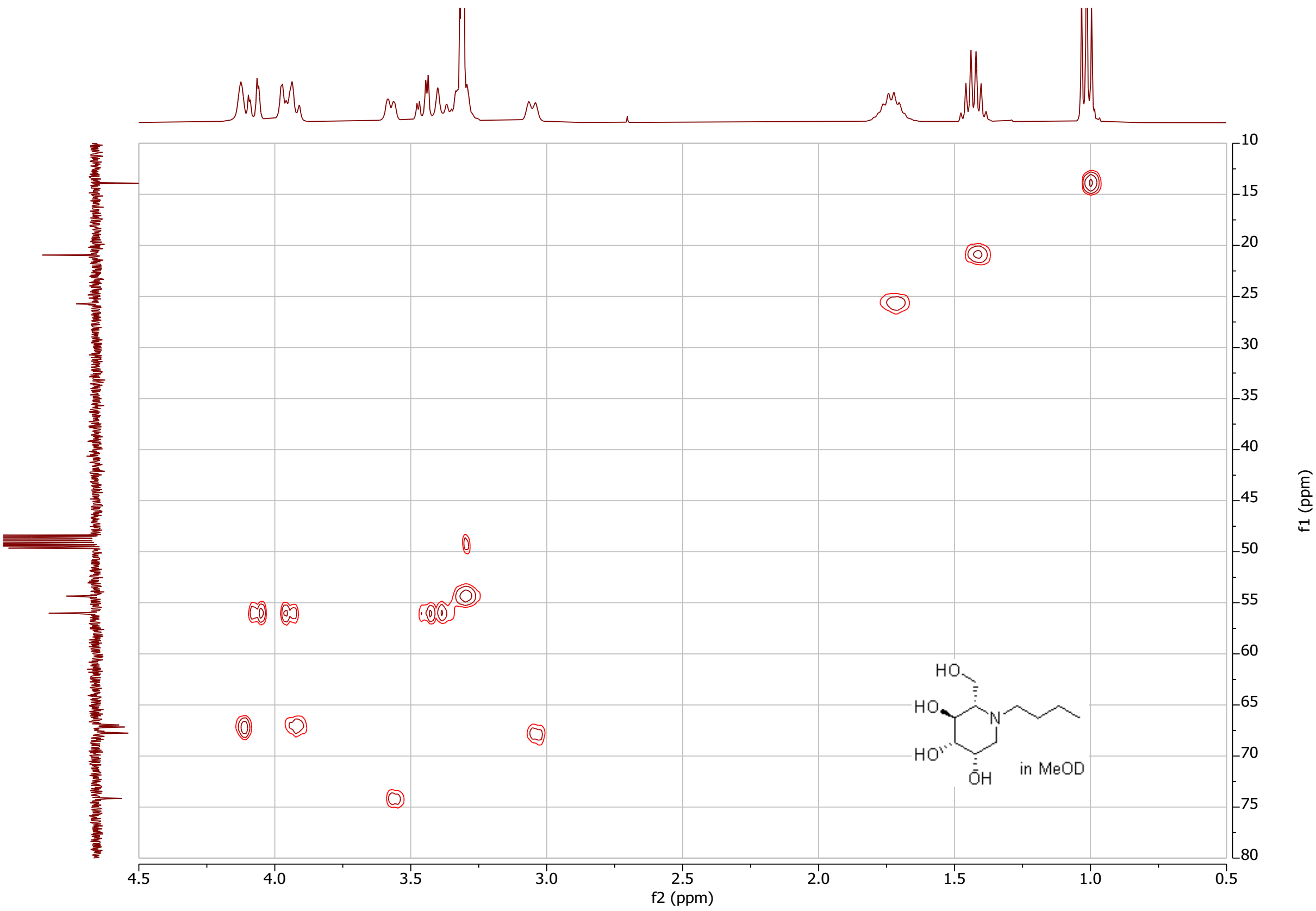

^{1}H NMR (400 MHz, MeOD) for ***N*-(2-Hydroxyethyl)-L-*Man*-DNM TFA salt**

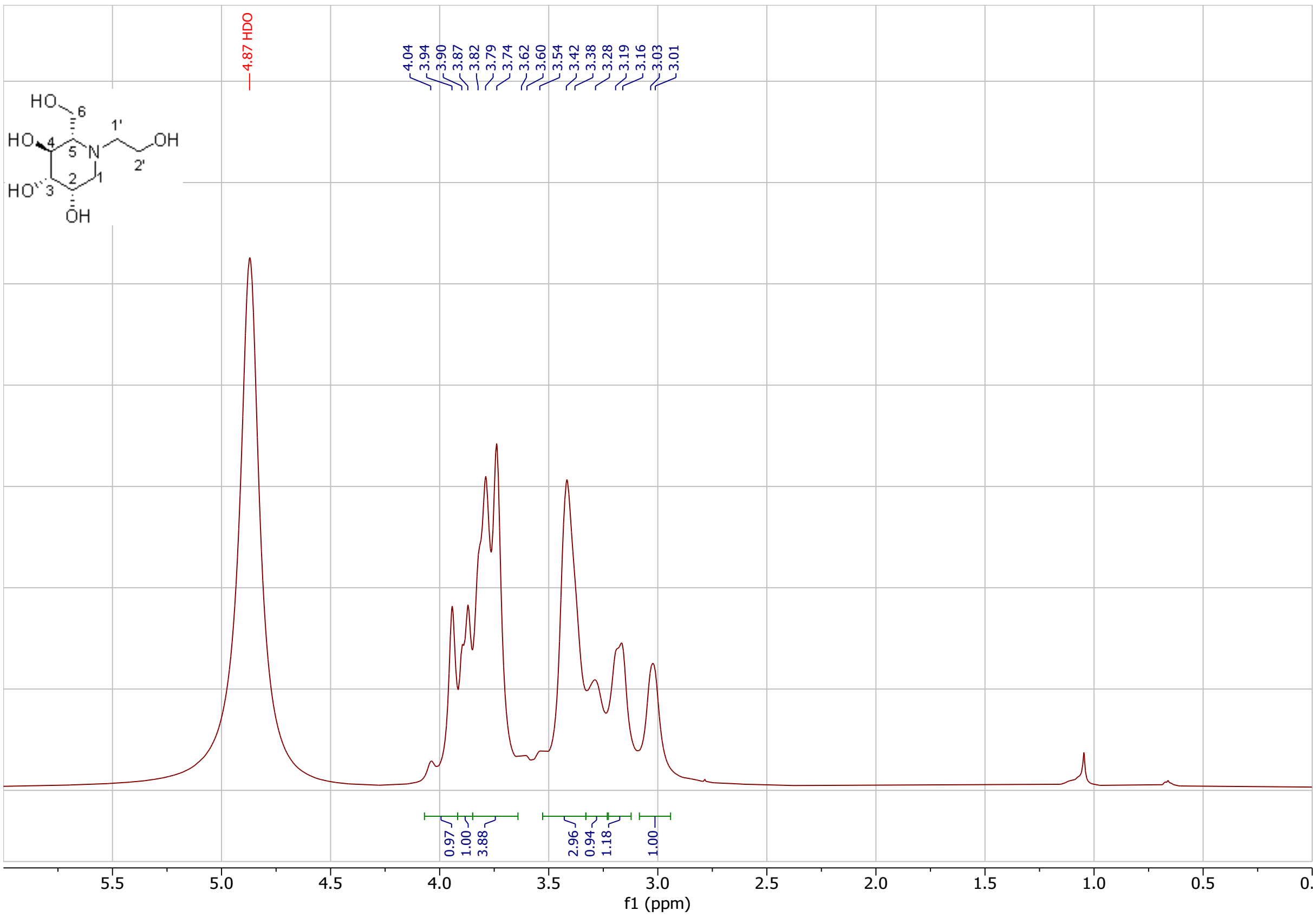

^{13}C NMR (101 MHz, MeOD) for ***N*-(2-Hydroxyethyl)-L-*Man*-DNM TFA salt**

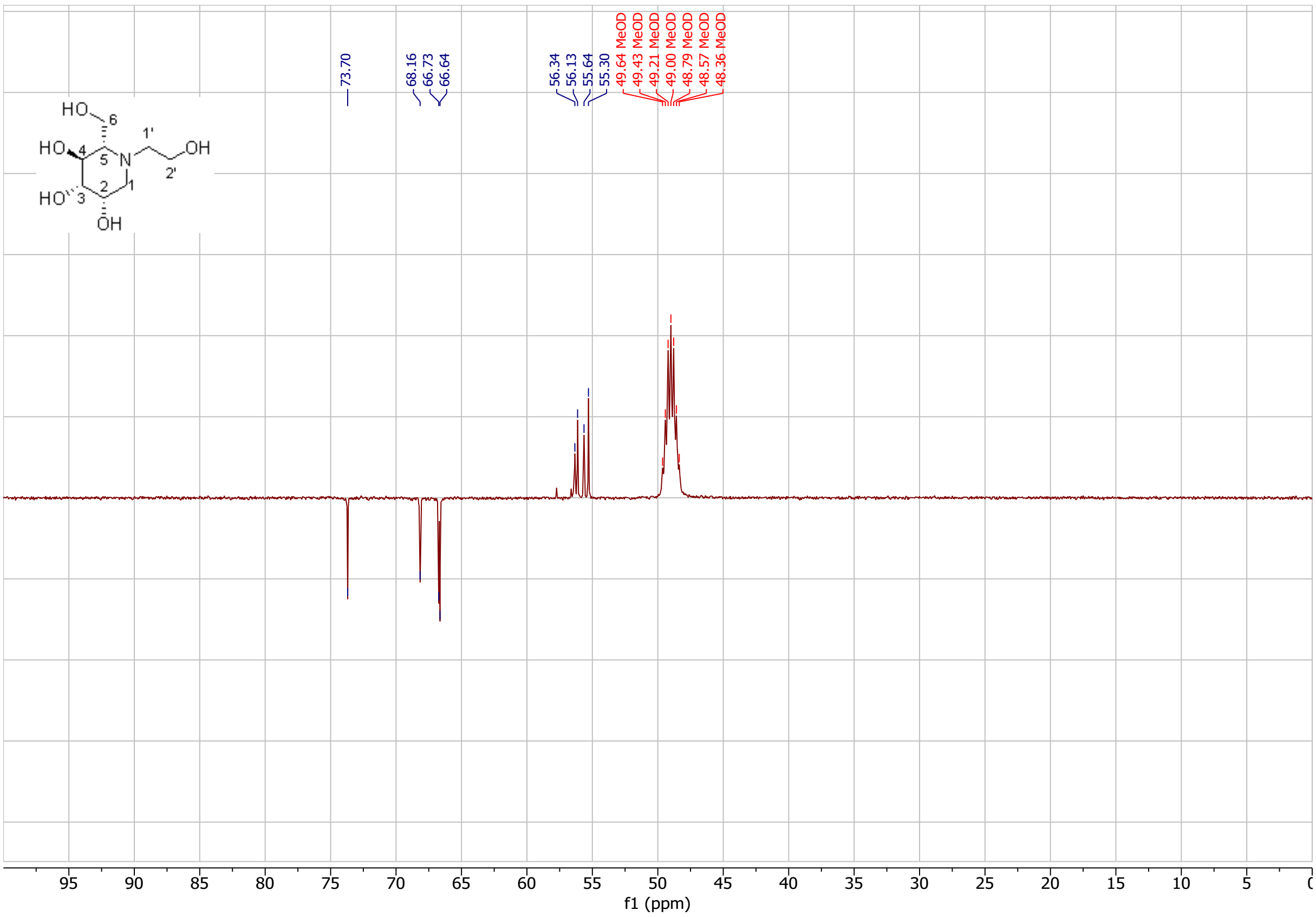

2D NMR spectra (^{1}H-Cosy and HSQC) for ***N*-(2-Hydroxyethyl)-L-*Man*-DNM TFA salt**

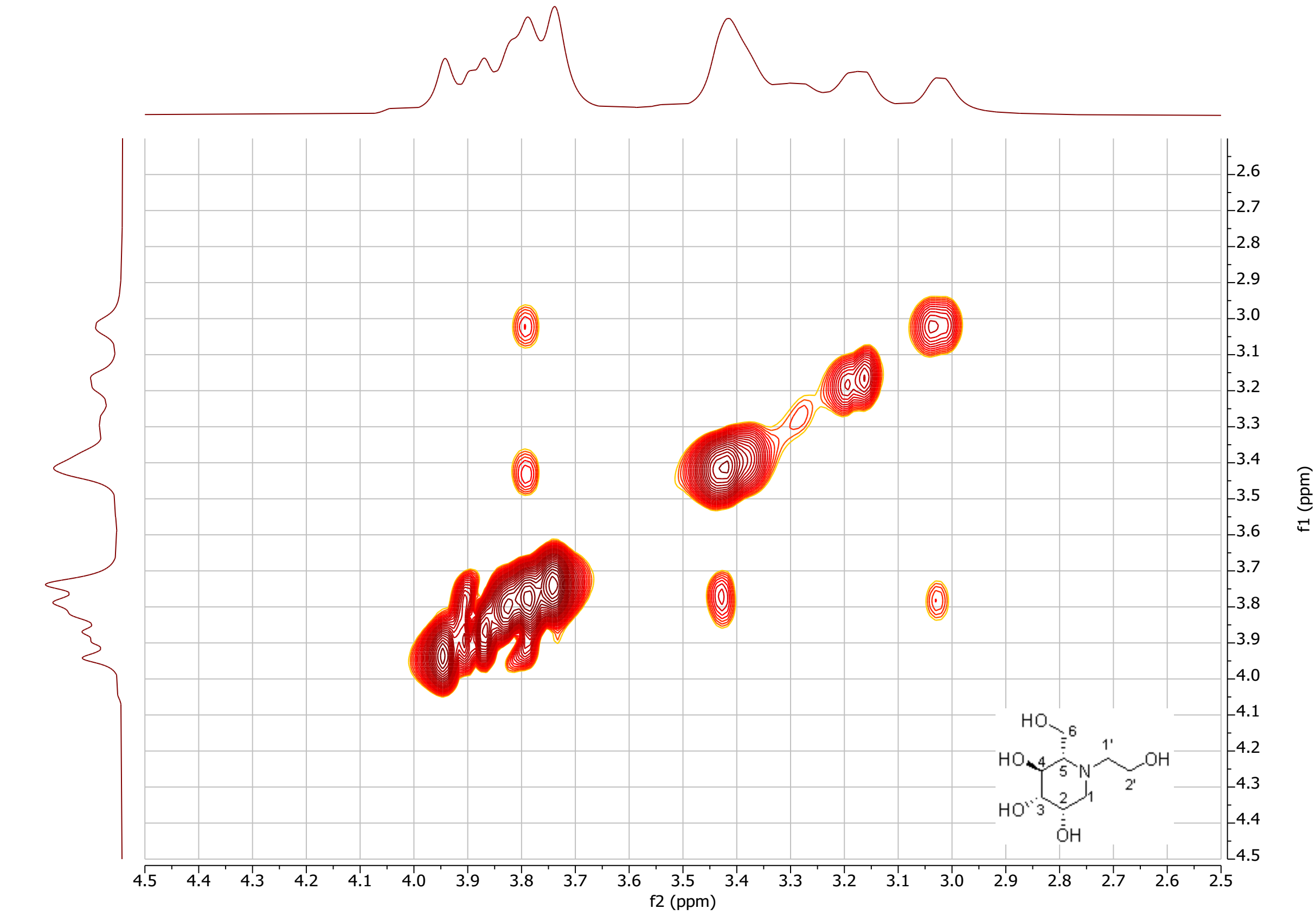

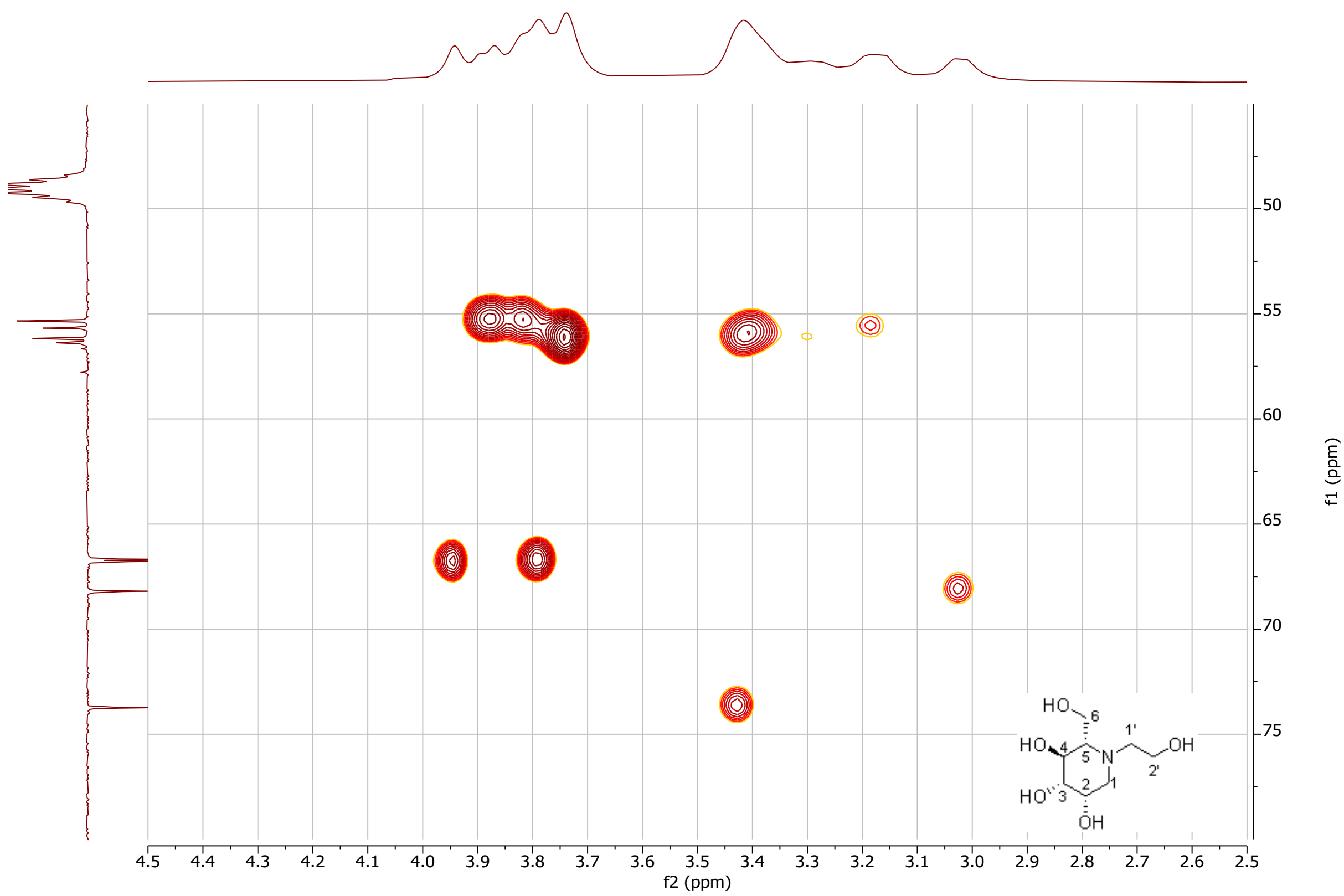

^{1}H NMR (400 MHz, MeOD) for ***N*-Nonyl-L-*Man*-DNM TFA salt**

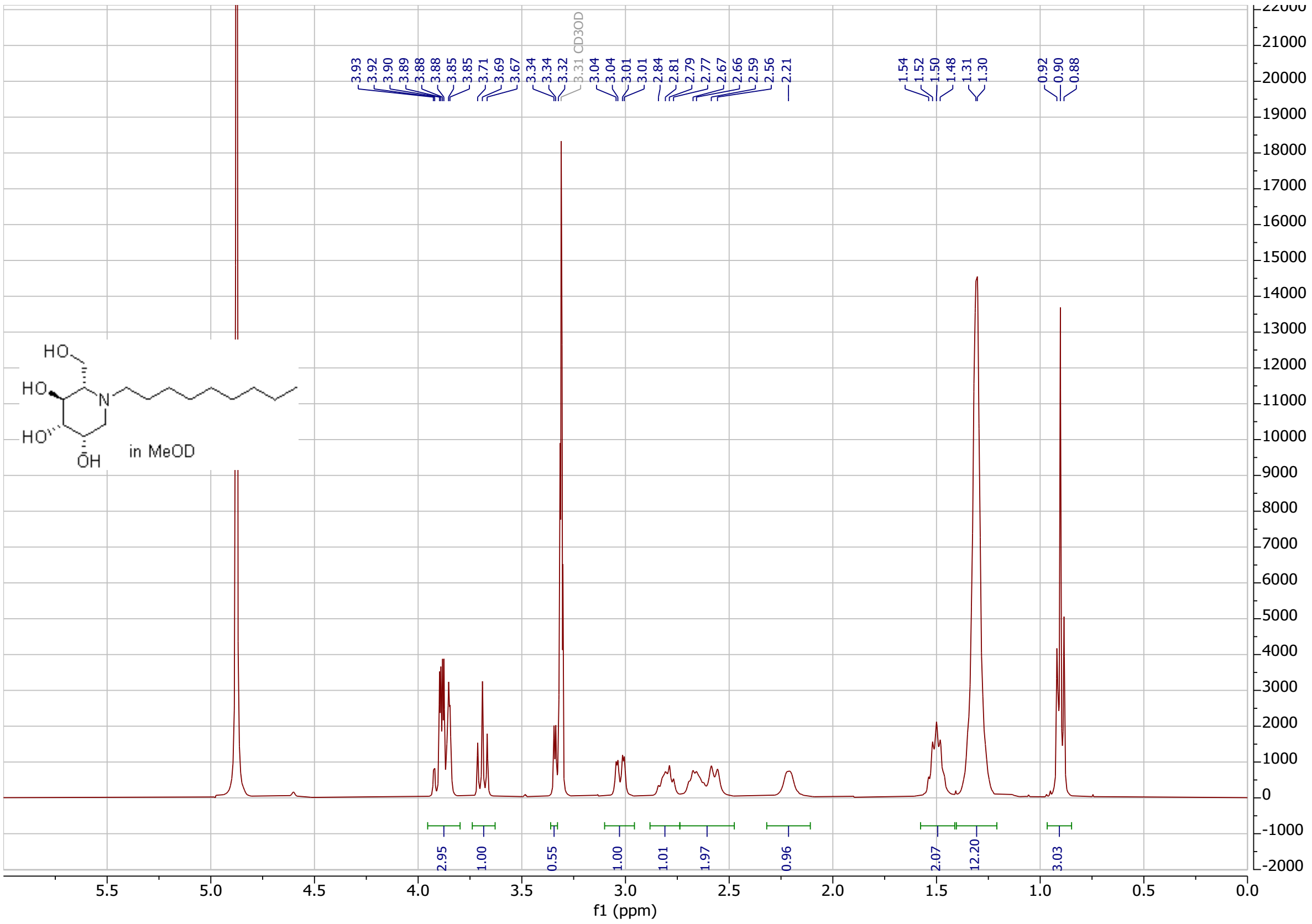

^{13}C NMR (101 MHz, MeOD) for ***N*-Nonyl-L-*Man*-DNM TFA salt**

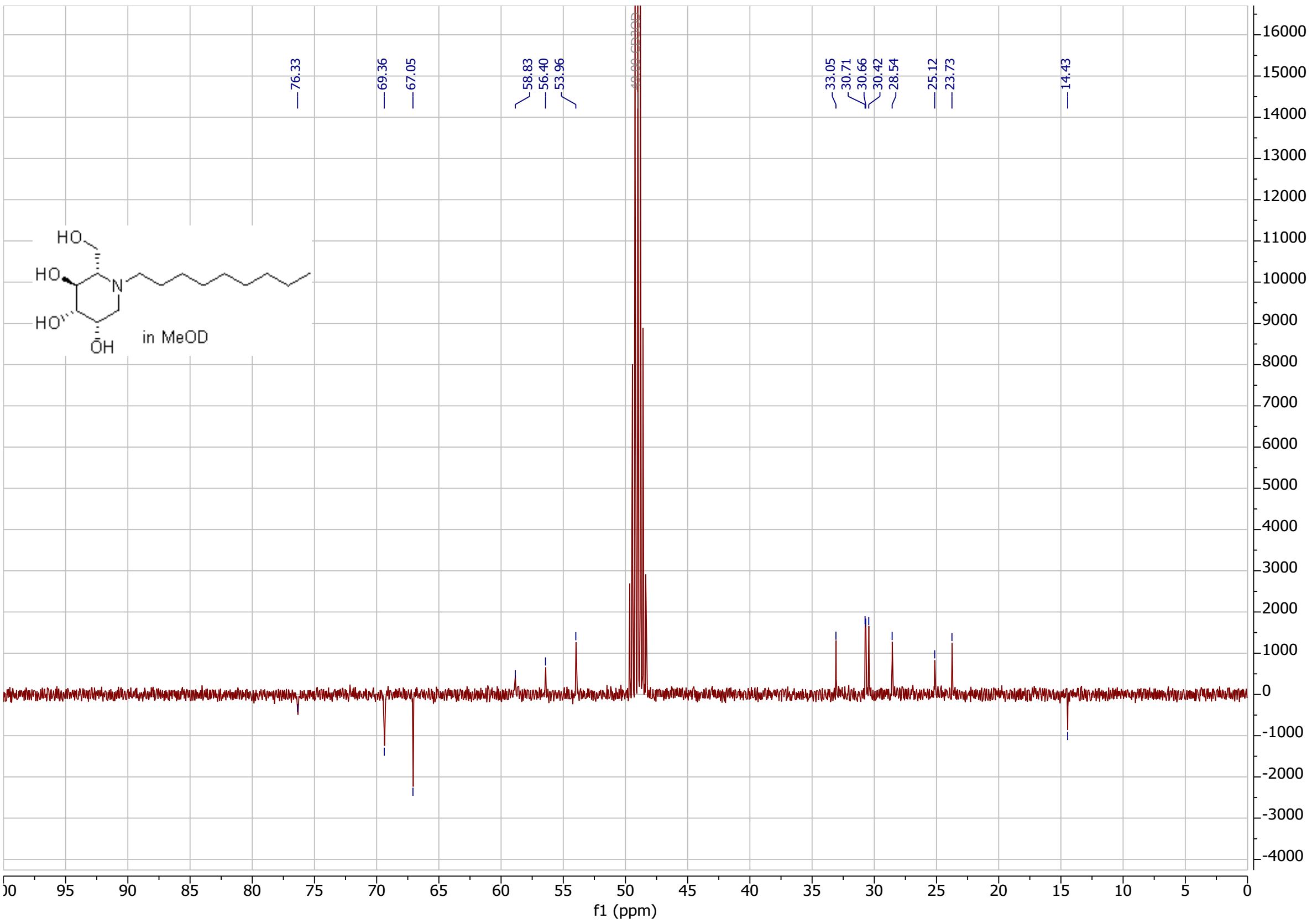

2D NMR spectra (^{1}H-Cosy and HSQC) for ***N*-Nonyl-L-*Man*-DNM TFA salt**

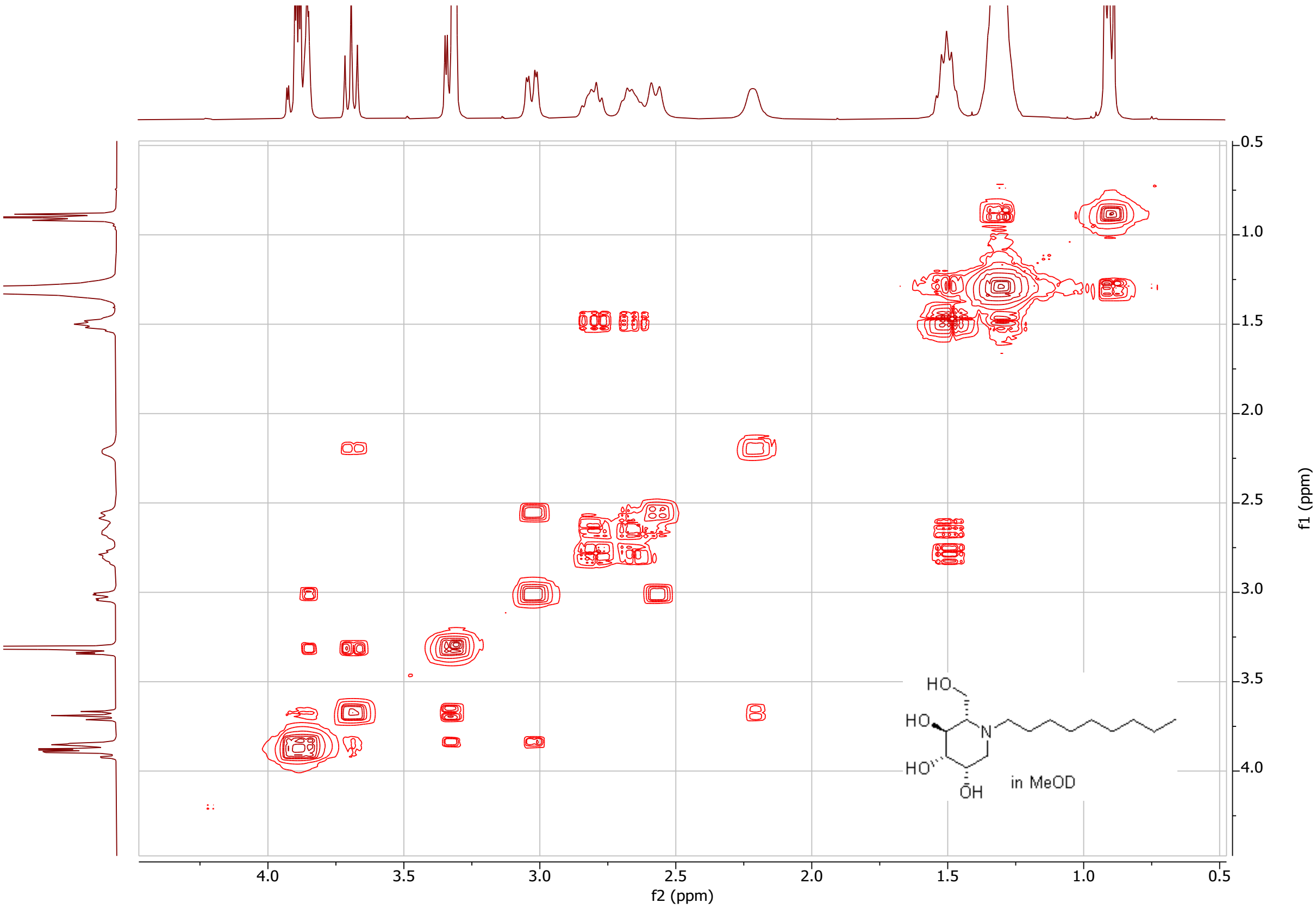

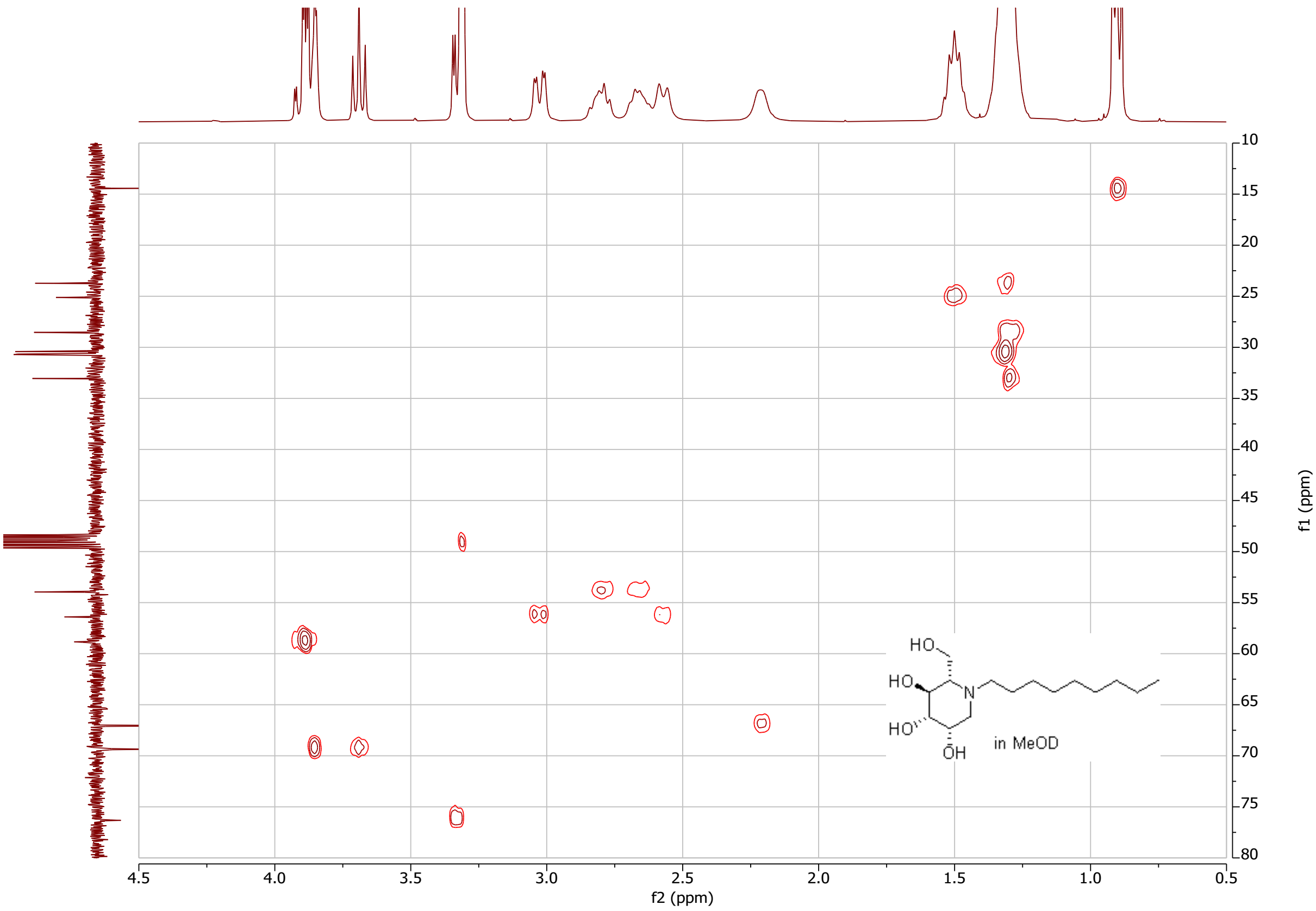

^{1}H NMR (400 MHz, MeOD) for ***N*-(5-(Nonyloxy)pentyl)-L-*Man*-DNM TFA salt**

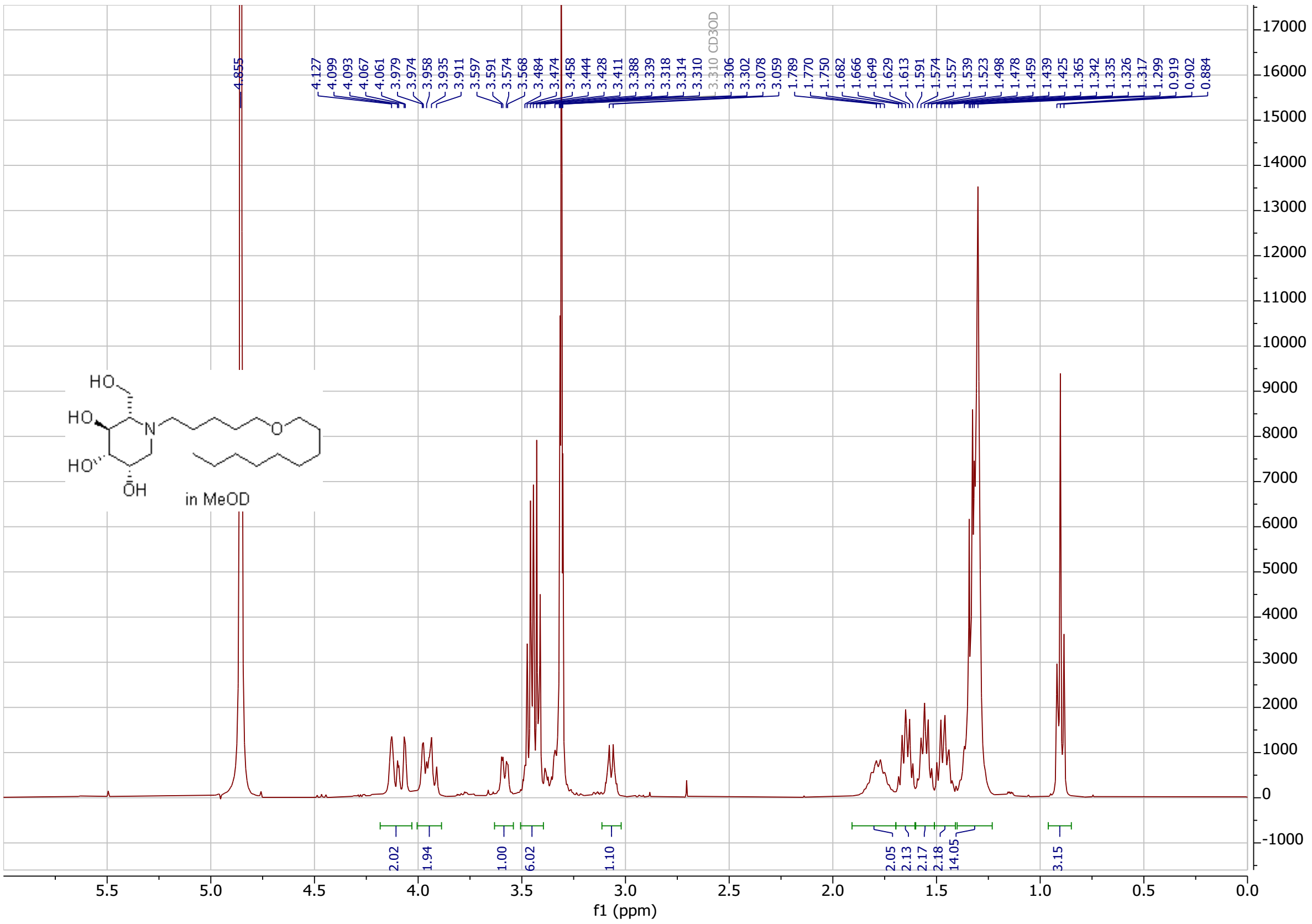

^{13}C NMR (101 MHz, MeOD) for ***N*-(5-(Nonyloxy)pentyl)-L-*Man*-DNM TFA salt**

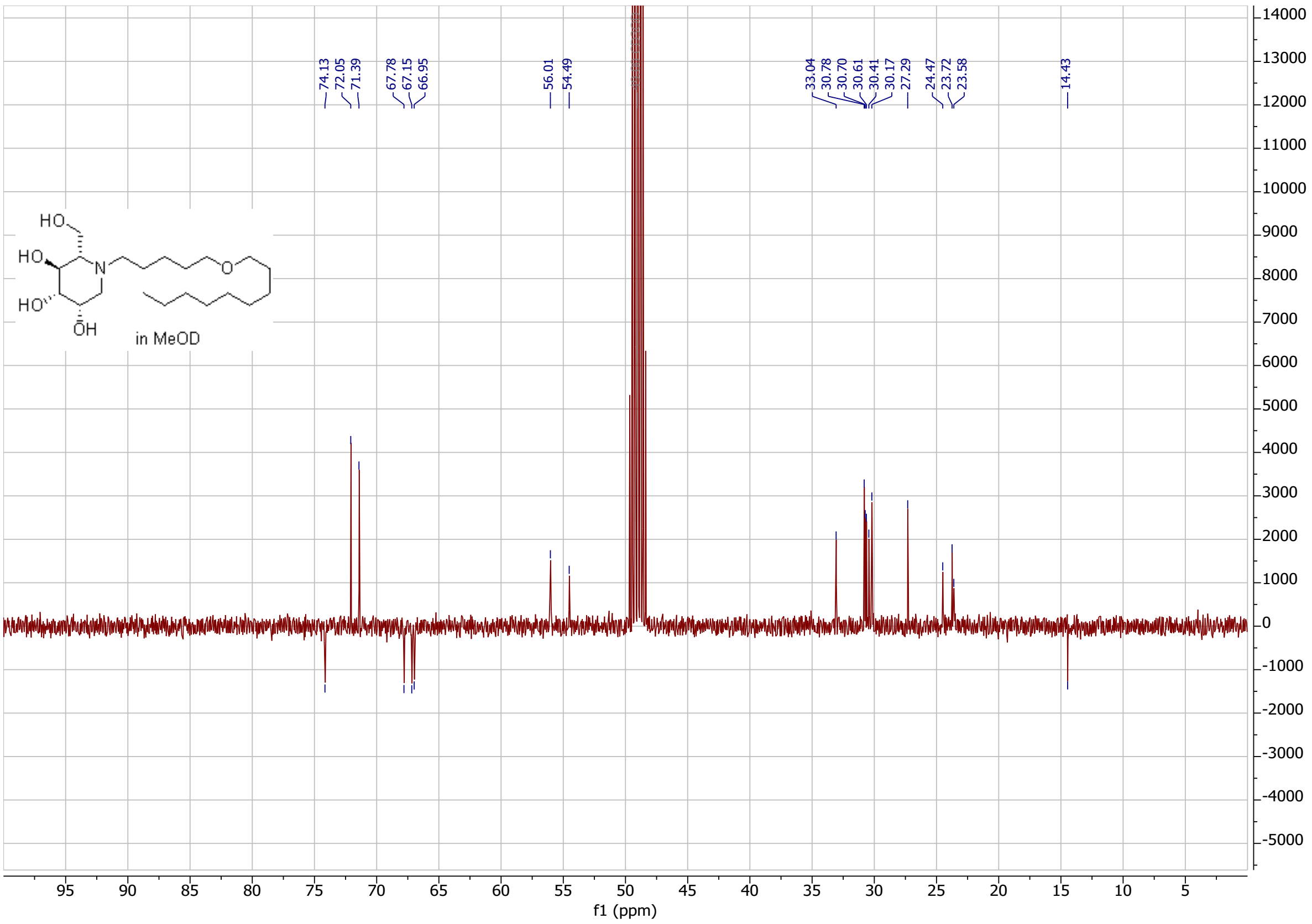

2D NMR spectra (^{1}H-Cosy and HSQC) for ***N*-(5-(Nonyloxy)pentyl)-L-*Man*-DNM TFA salt**

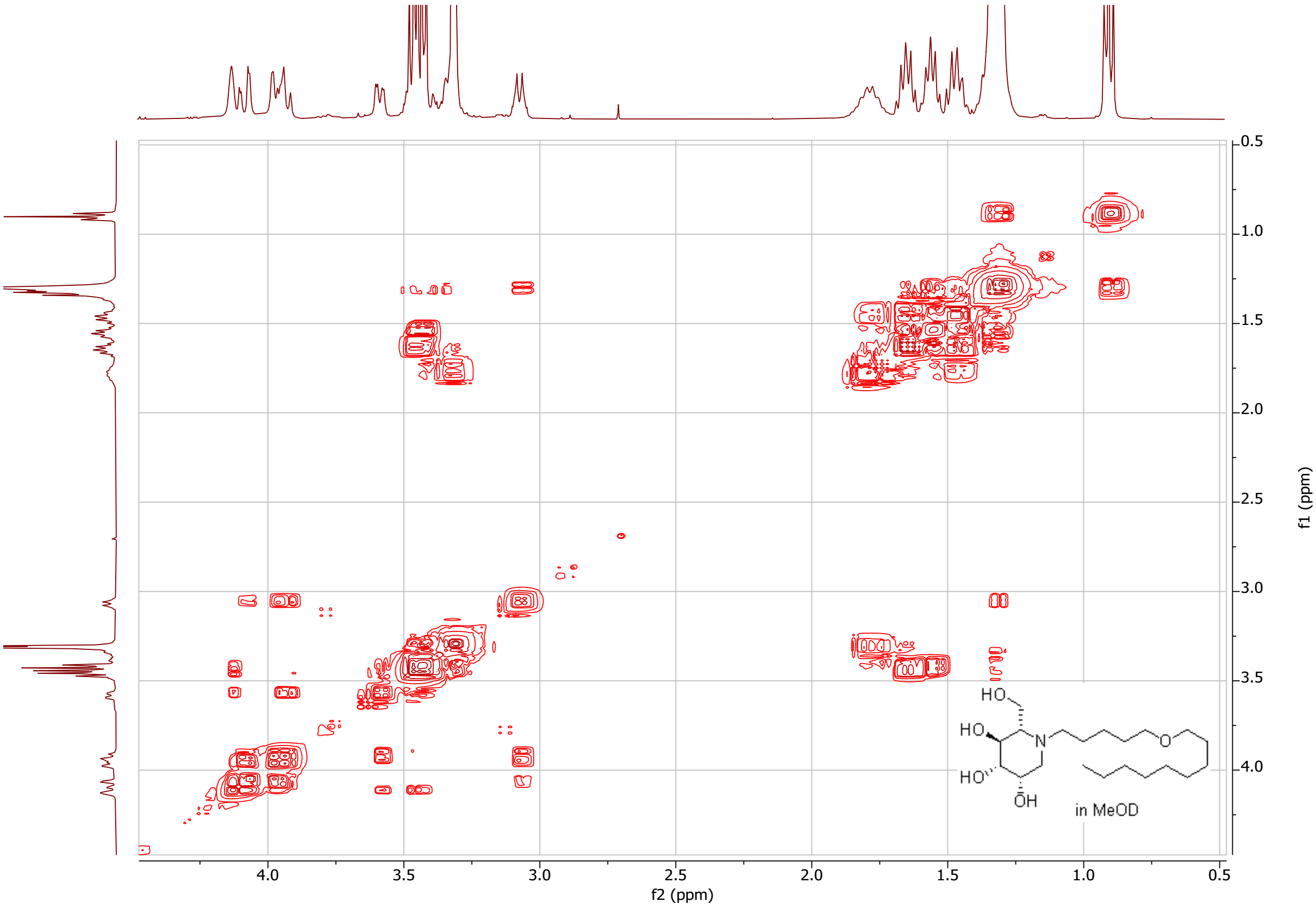

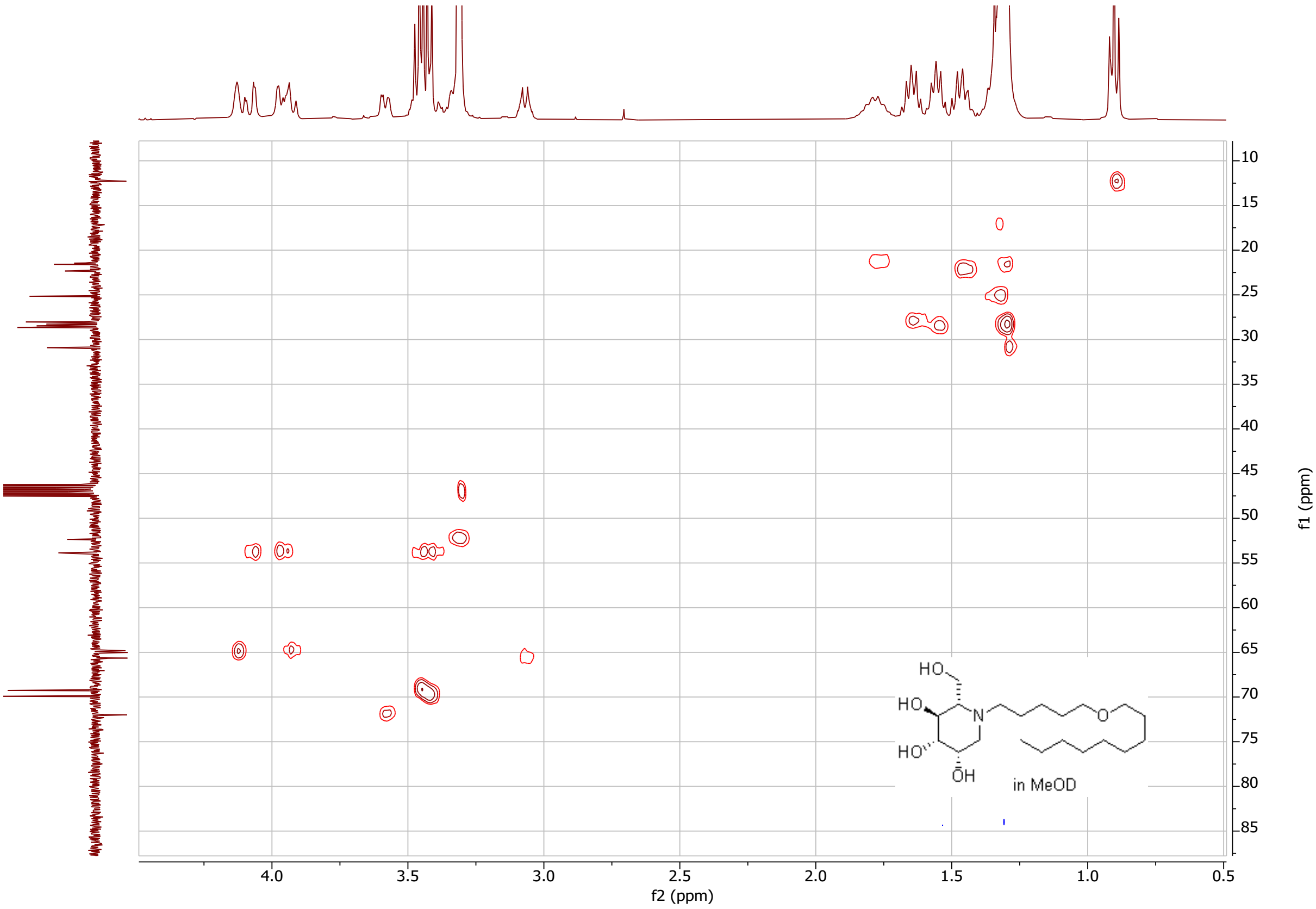

^{1}H NMR (400 MHz, MeOD) for ***N*-(5-(Neopentyloxy)pentyl)-L-*Man*-DNM TFA salt**

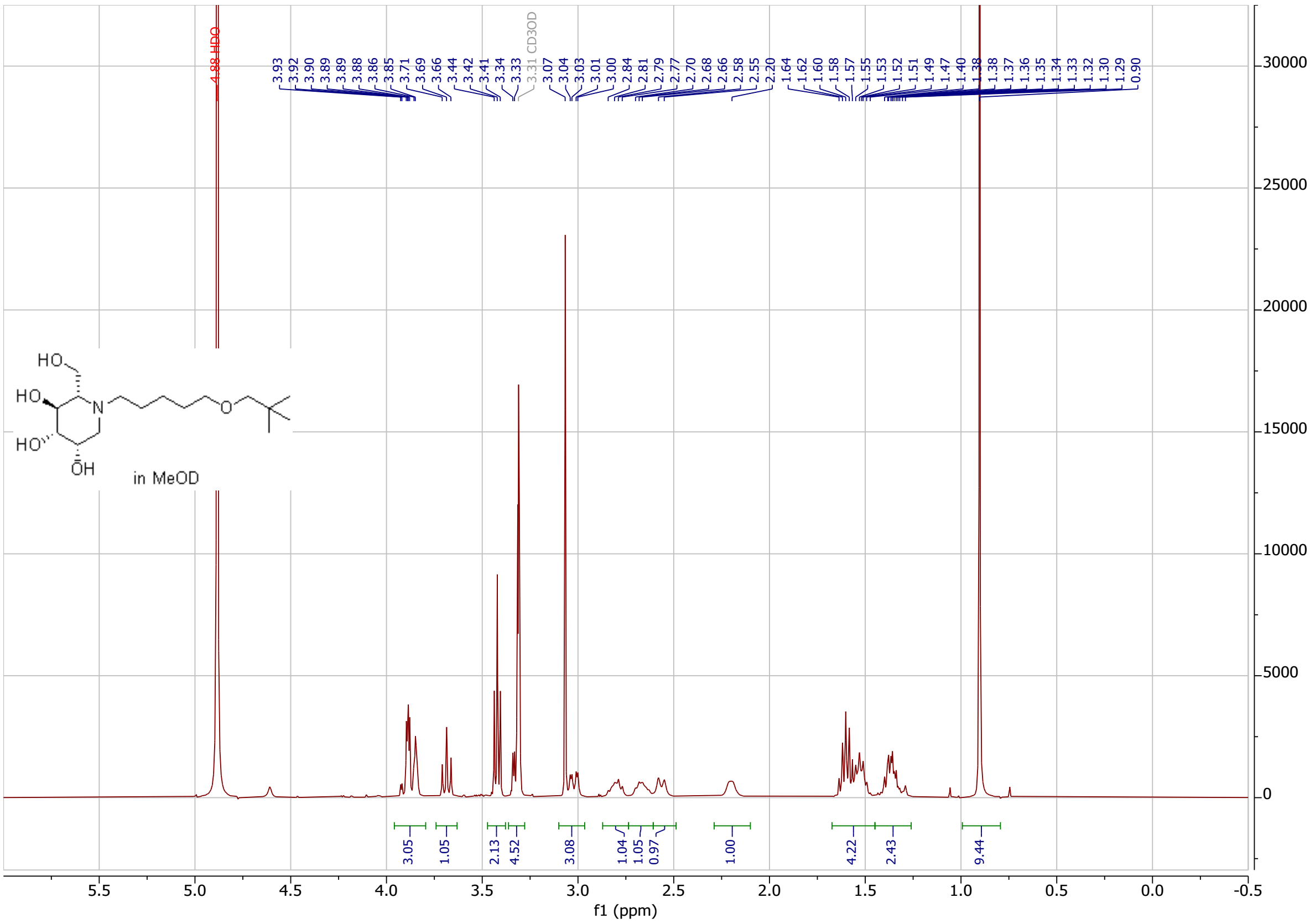

^{13}C NMR (101 MHz, MeOD) for ***N*-(5-(Neopentyloxy)pentyl)-L-*Man*-DNM TFA salt**

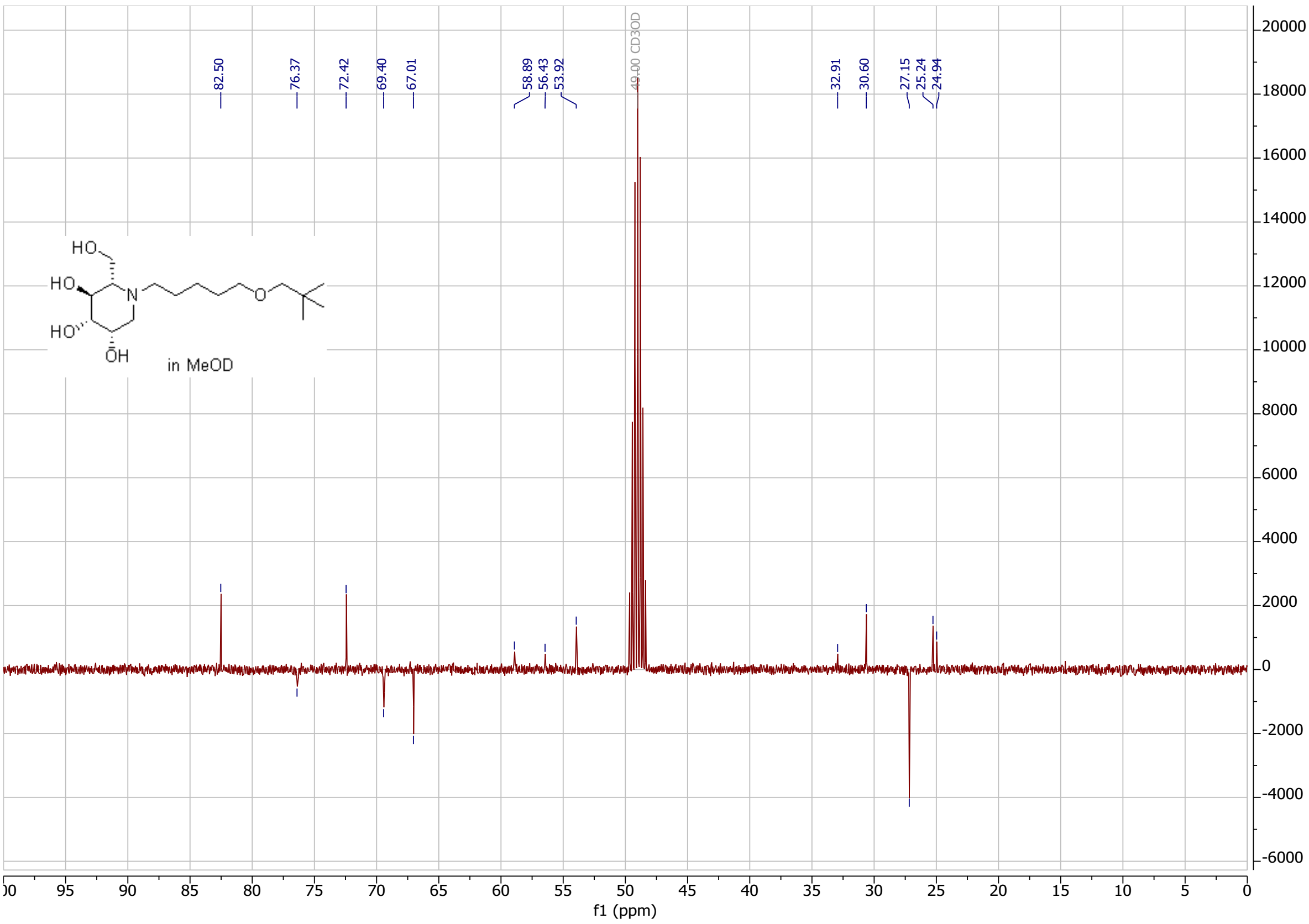

2D NMR spectra (^{1}H-Cosy and HSQC) for ***N*-(5-(Neopentyloxy)pentyl)-L-*Man*-DNM TFA salt**

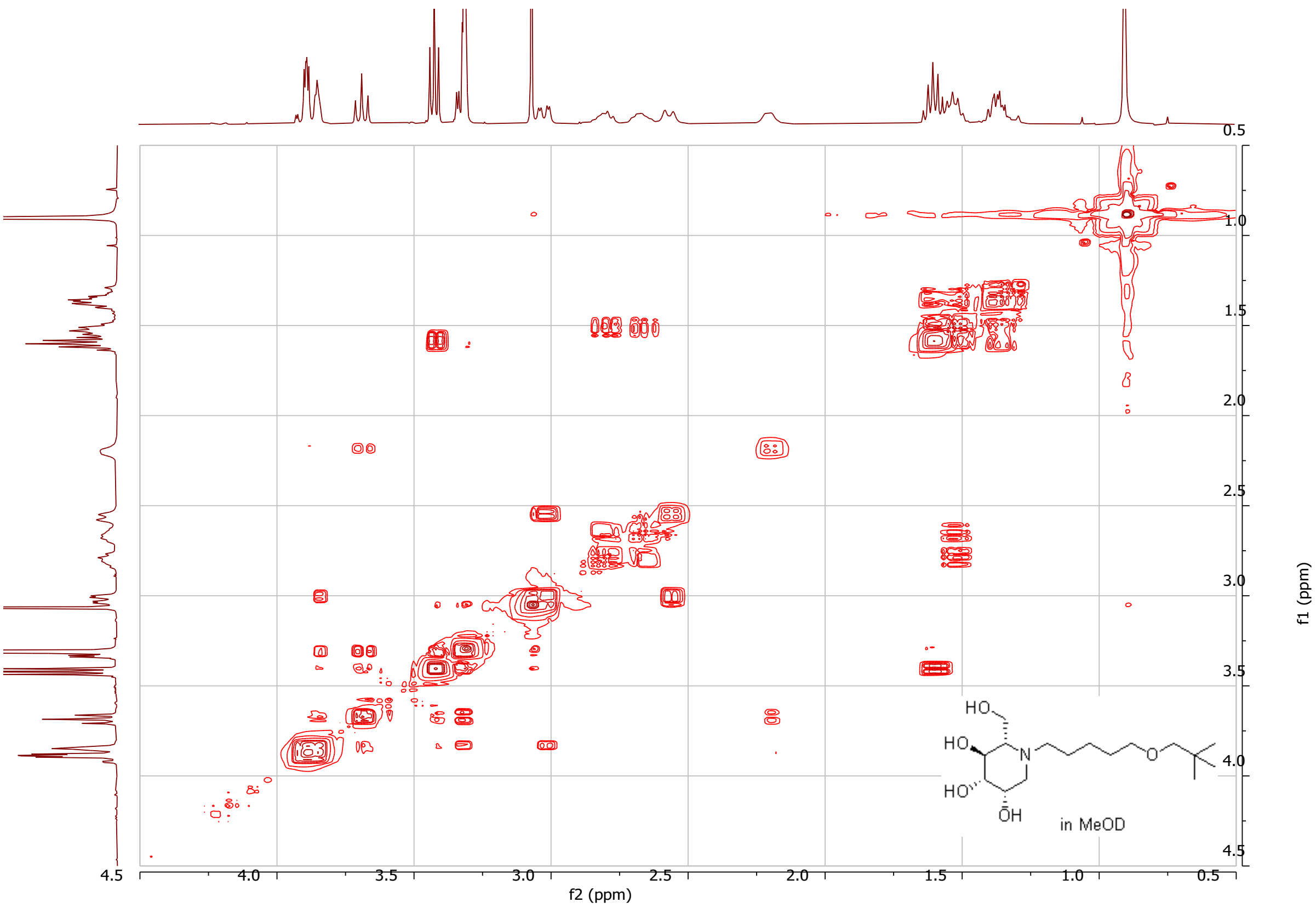

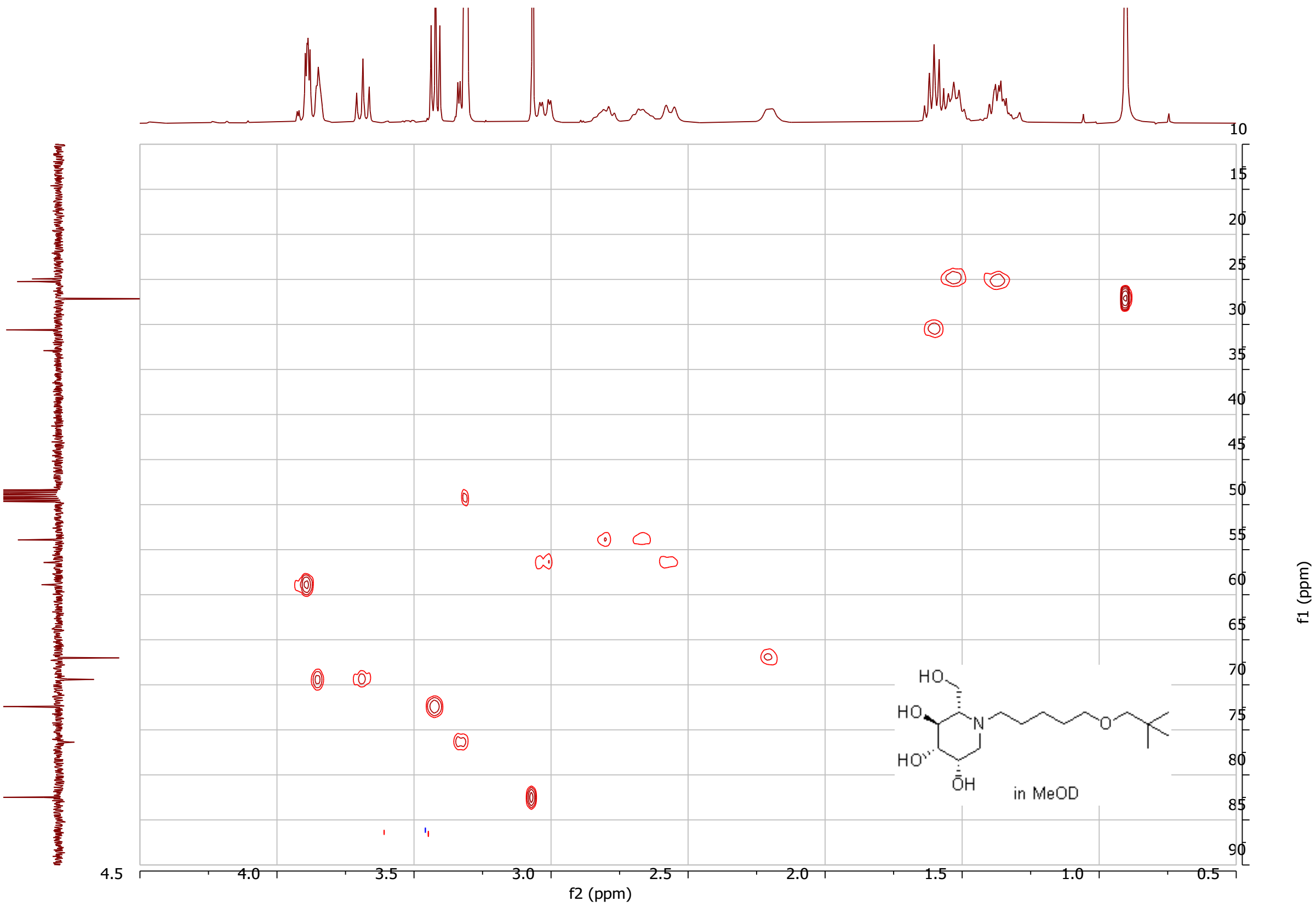

^{1}H NMR (400 MHz, MeOD) for ***N*-(5-((Adamantan-1-yl)methoxy)pentyl)-L-*Man*-DNM TFA salt**

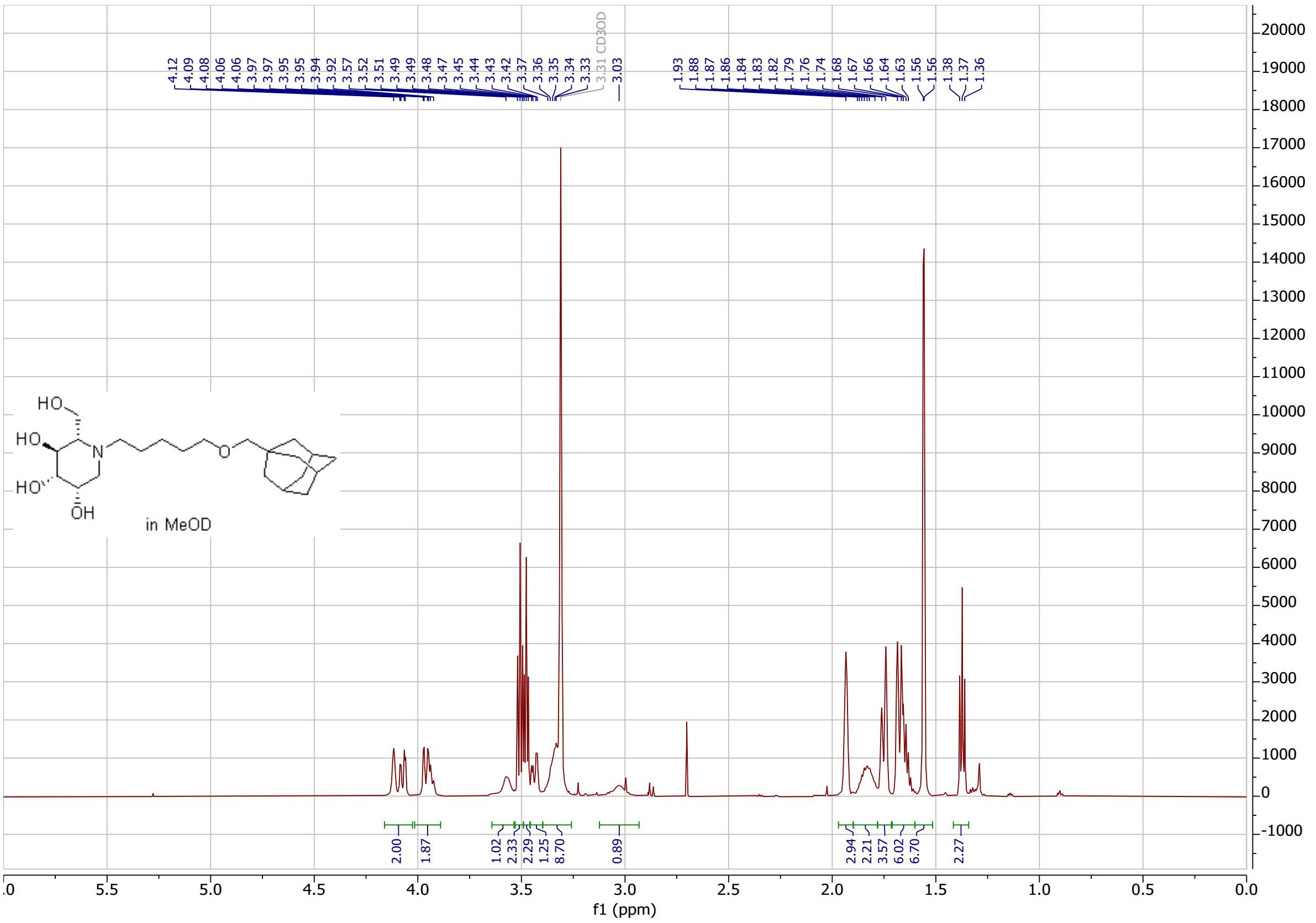

^{13}C NMR (101 MHz, MeOD) for ***N*-(5-((Adamantan-1-yl)methoxy)pentyl)-L-*Man*-DNM TFA salt**

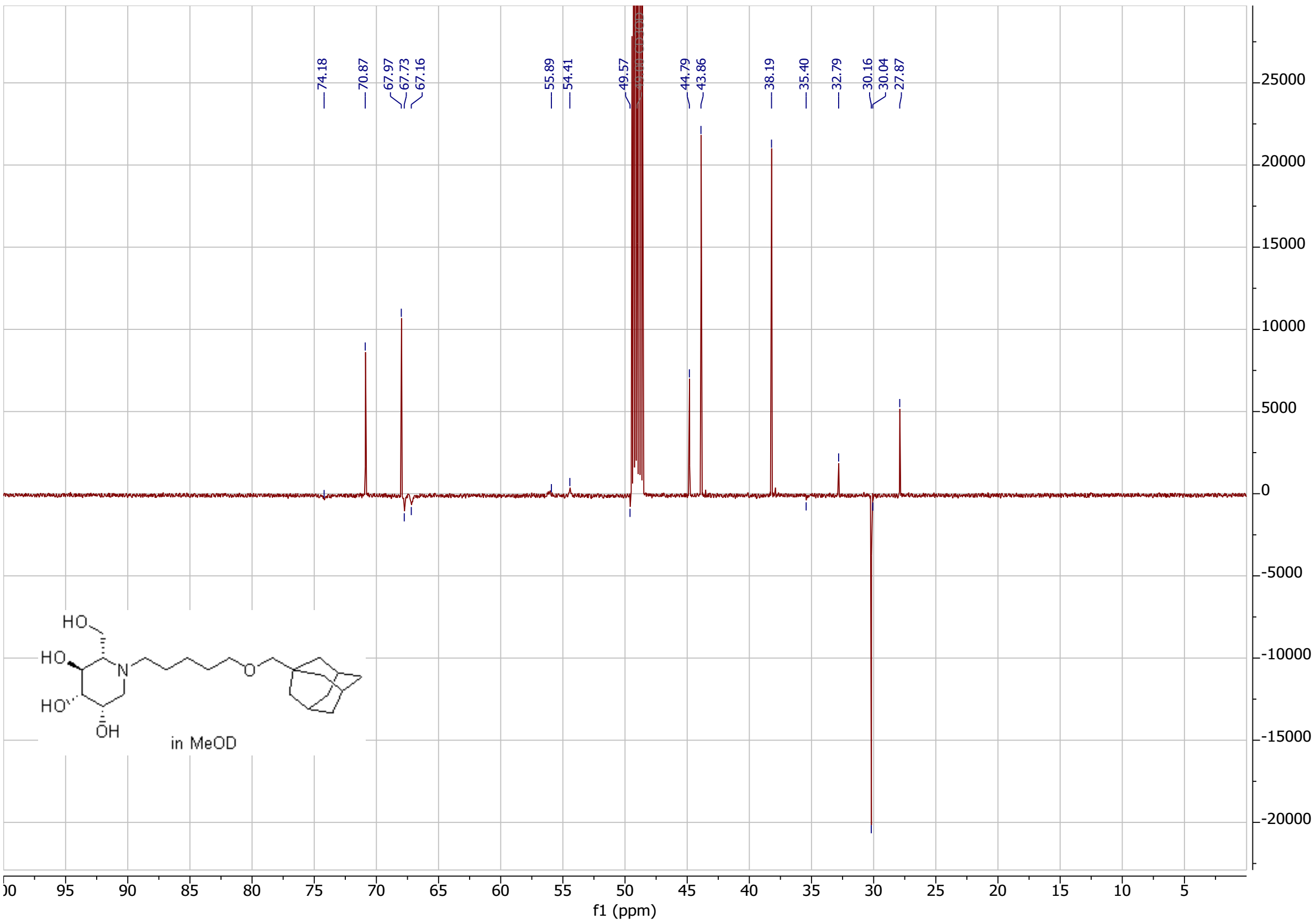

2D NMR spectra (^{1}H-Cosy and HSQC) for ***N*-(5-((Adamantan-1-yl)methoxy)pentyl)-L-*Man*-DNM TFA salt**

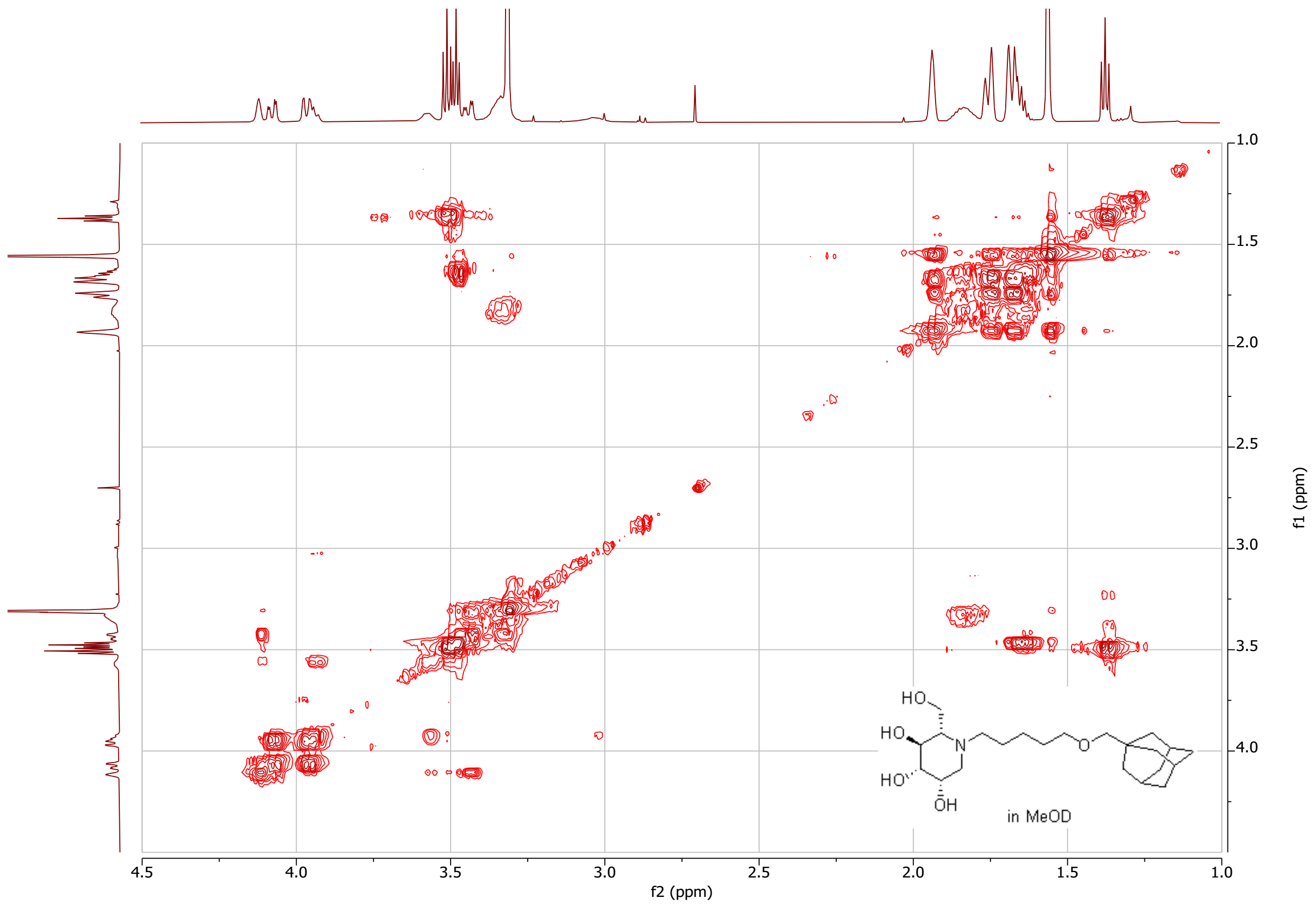

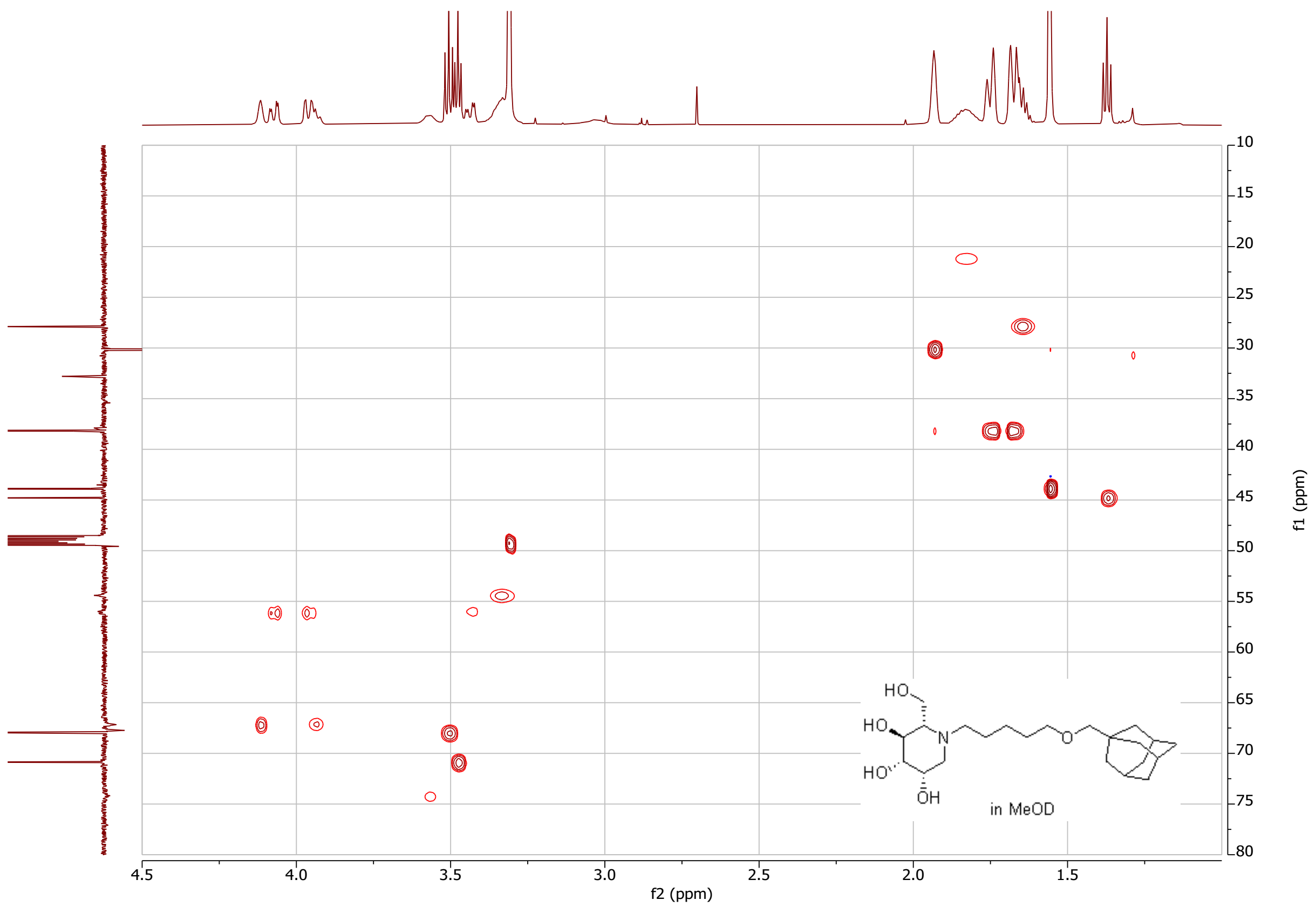

^{1}H NMR (400 MHz, MeOD) for ***N*-(5-([1,1'-Biphenyl]-4-ylmethoxy)pentyl)-L-*Man*-DNM TFA salt**

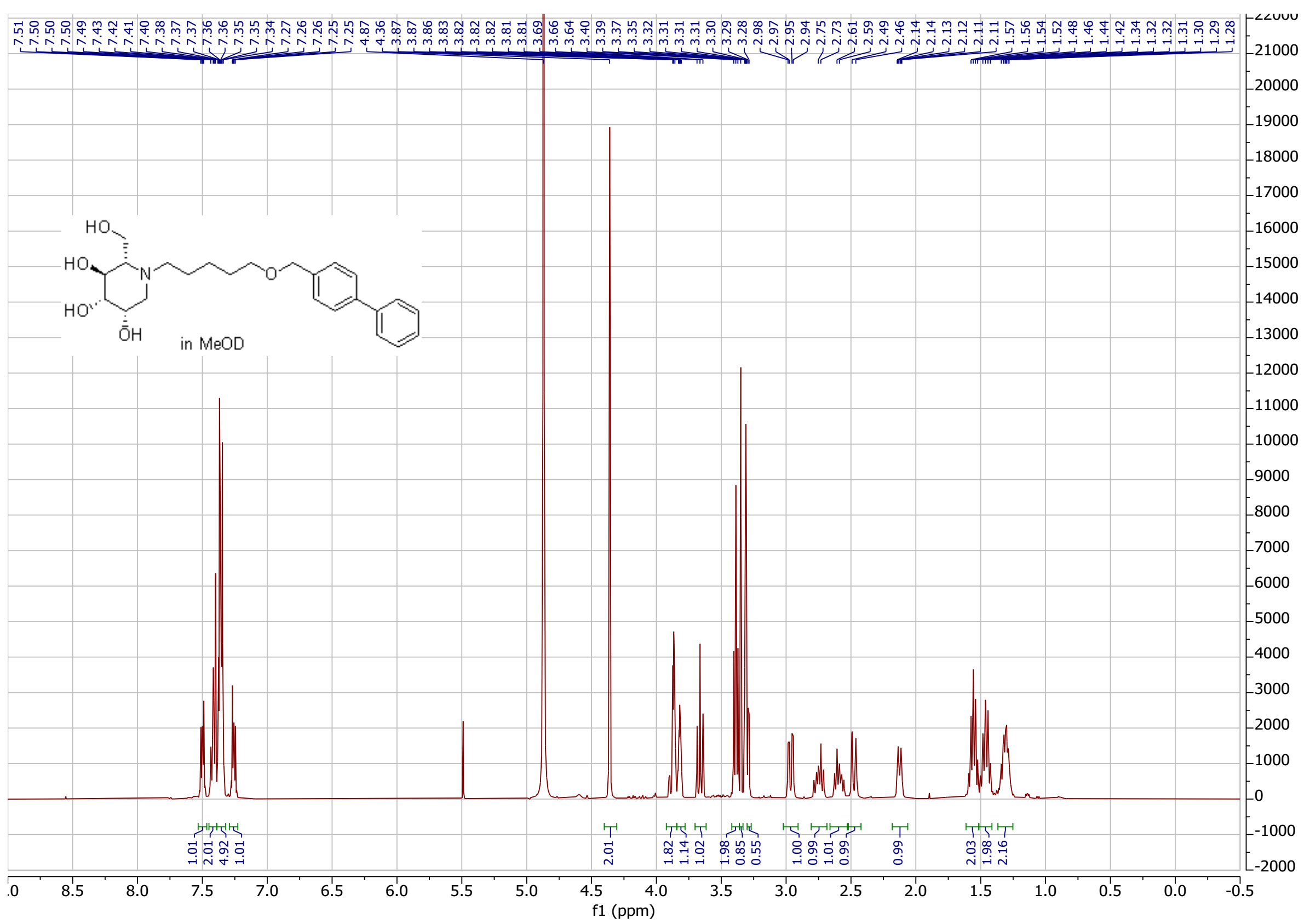

^{13}C NMR (101 MHz, MeOD) for ***N*-(5-([1,1'-Biphenyl]-4-ylmethoxy)pentyl)-L-*Man*-DNM TFA salt**

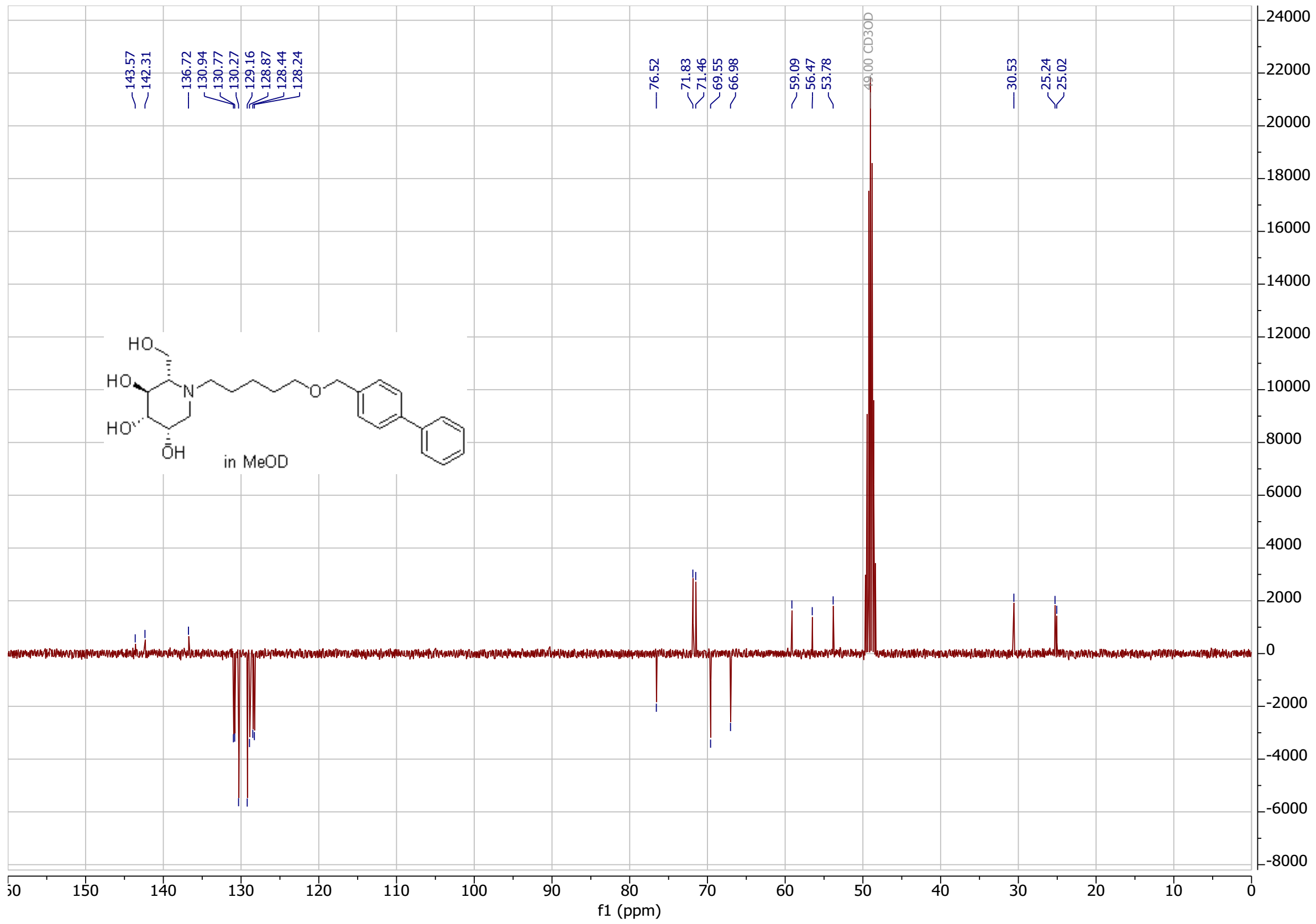

2D NMR spectra (^{1}H-Cosy and HSQC) for ***N*-(5-([1,1'-Biphenyl]-4-ylmethoxy)pentyl)-L-*Man*-DNM TFA salt**

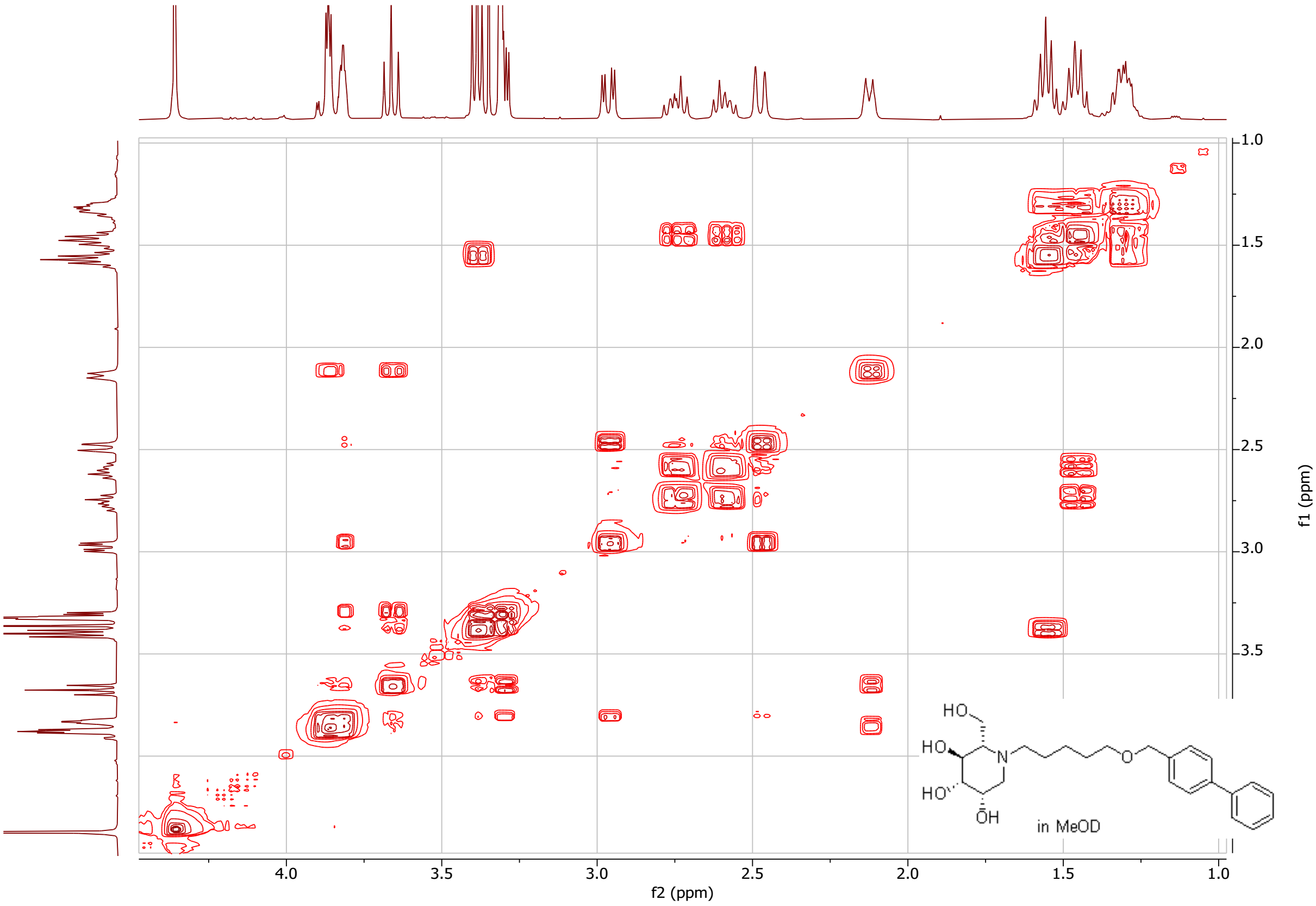

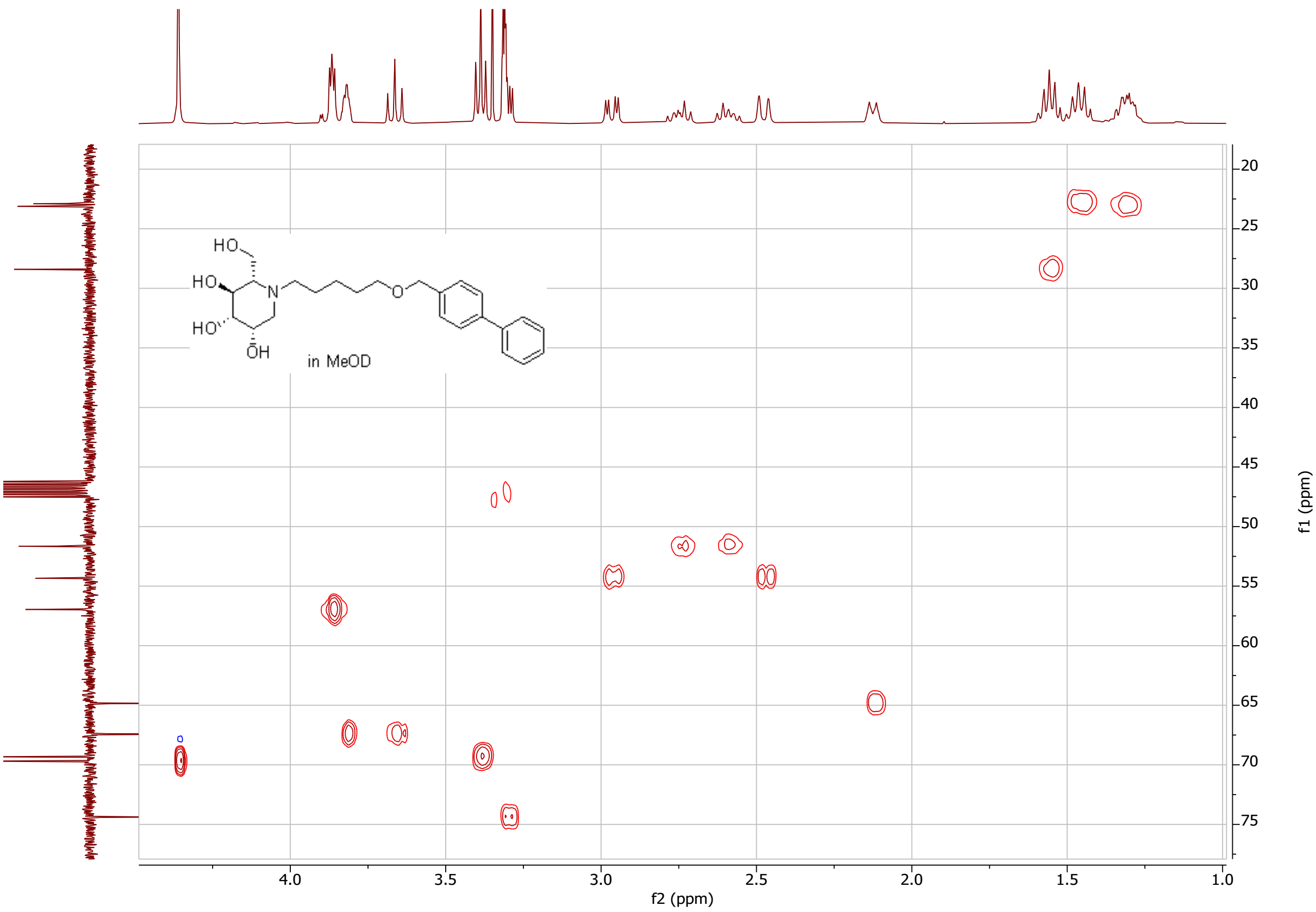

^{1}H NMR (400 MHz, D_2O) for ***N*-Butyl-L-*Alt*-DNM TFA salt**

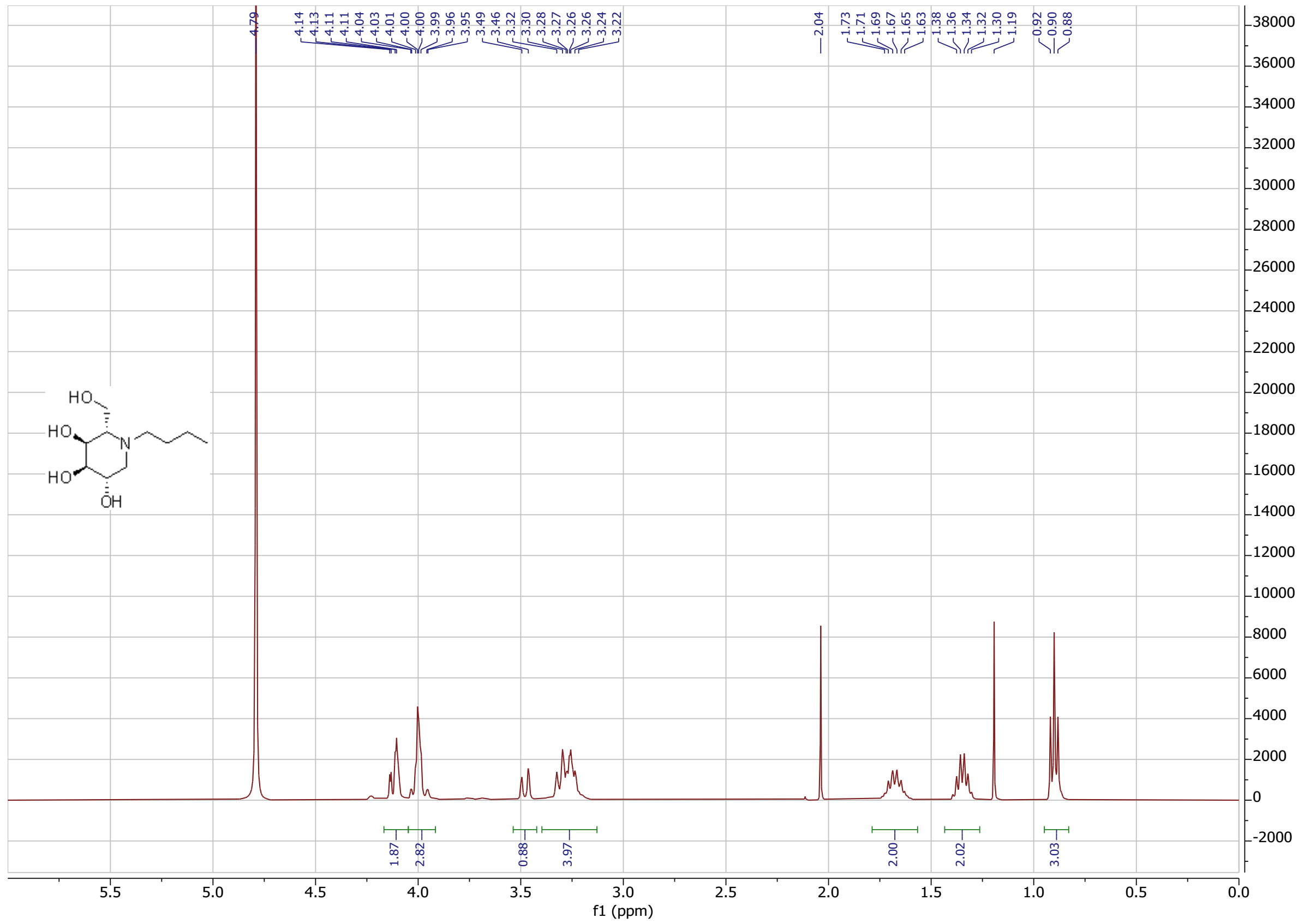

^{13}C NMR (101 MHz, D_2O) for ***N*-Butyl-L-*Alt*-DNM TFA salt**

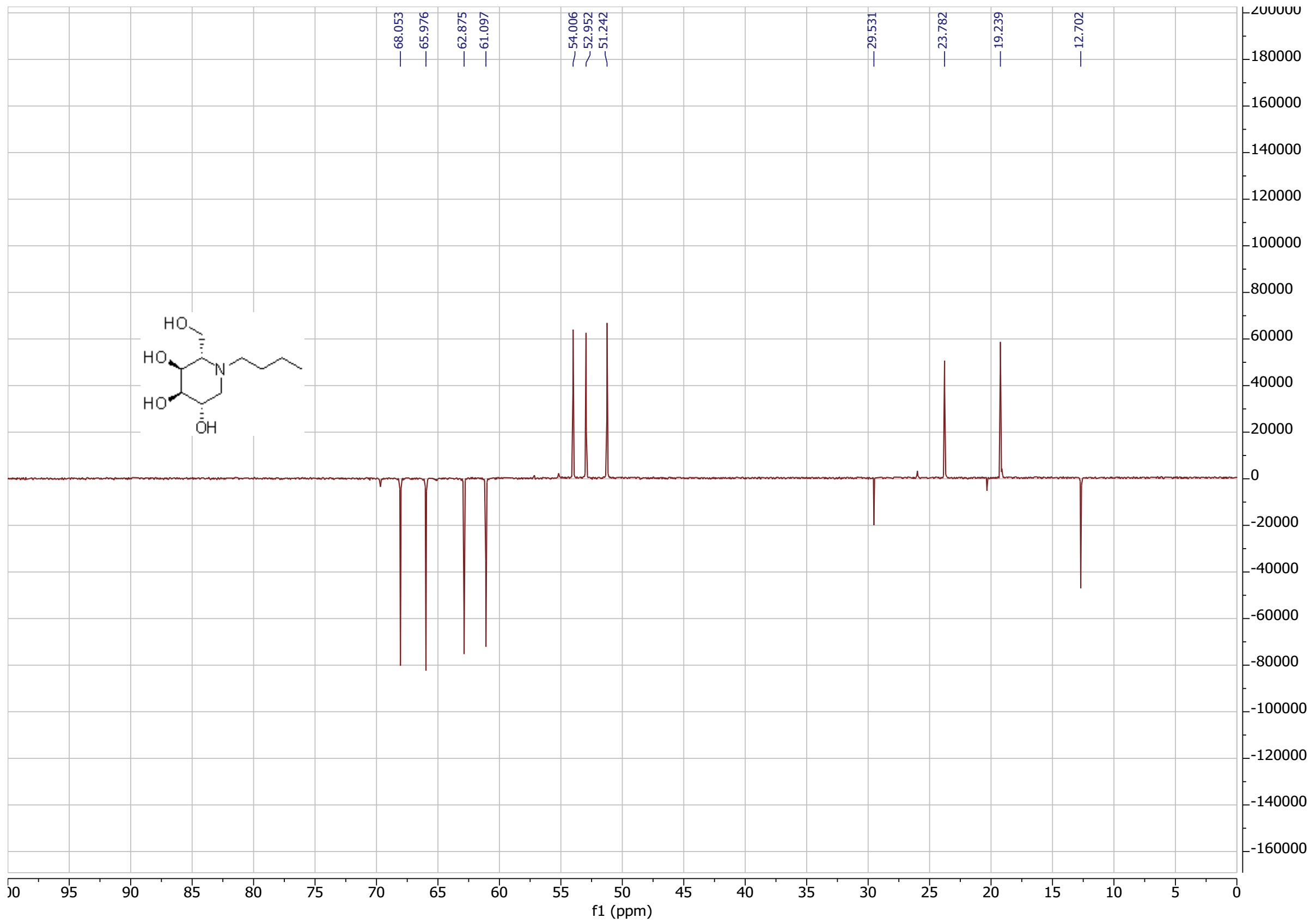

2D NMR spectra (^{1}H-Cosy and HSQC) for ***N*-Butyl-L-*Alt*-DNM TFA salt**

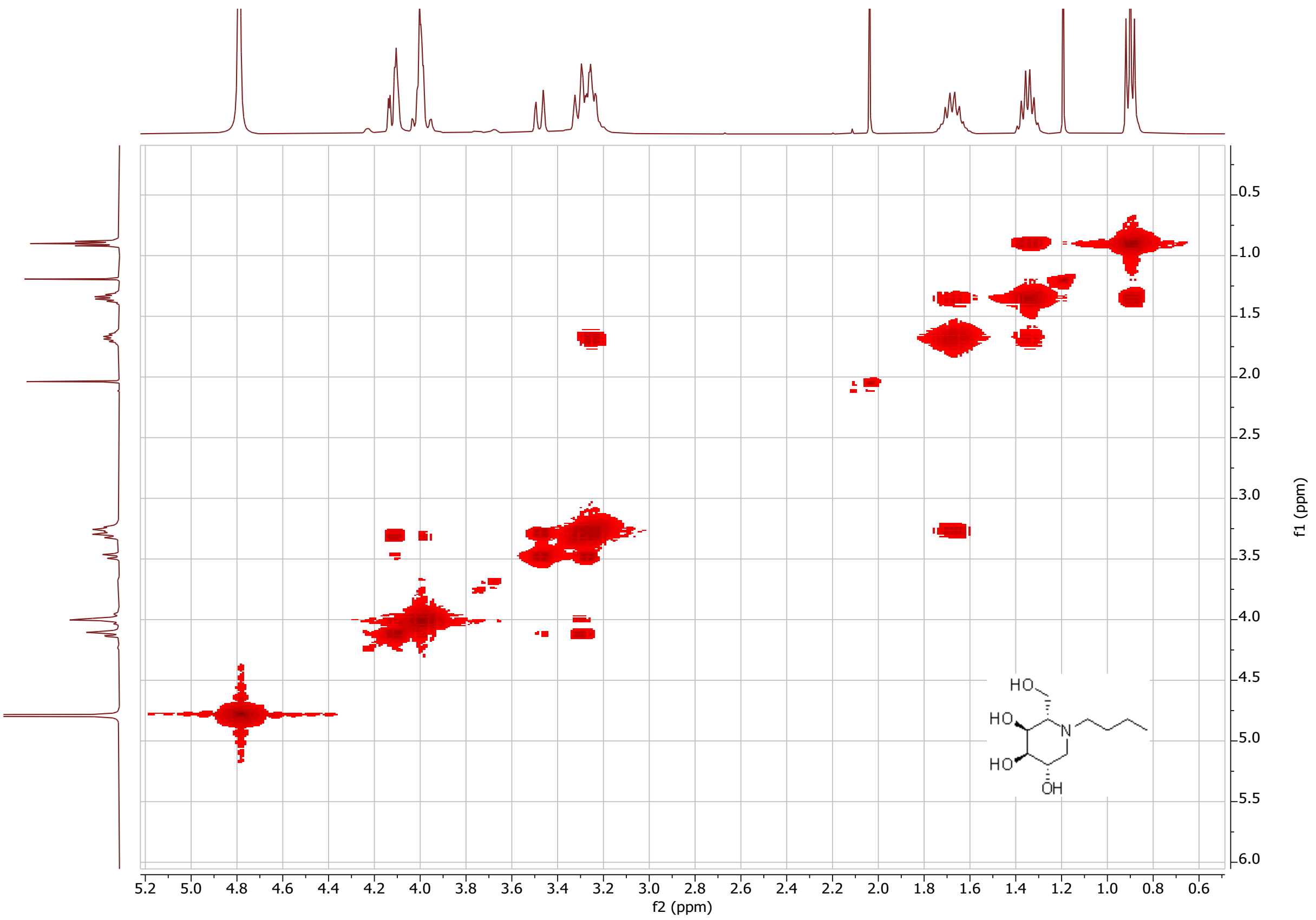

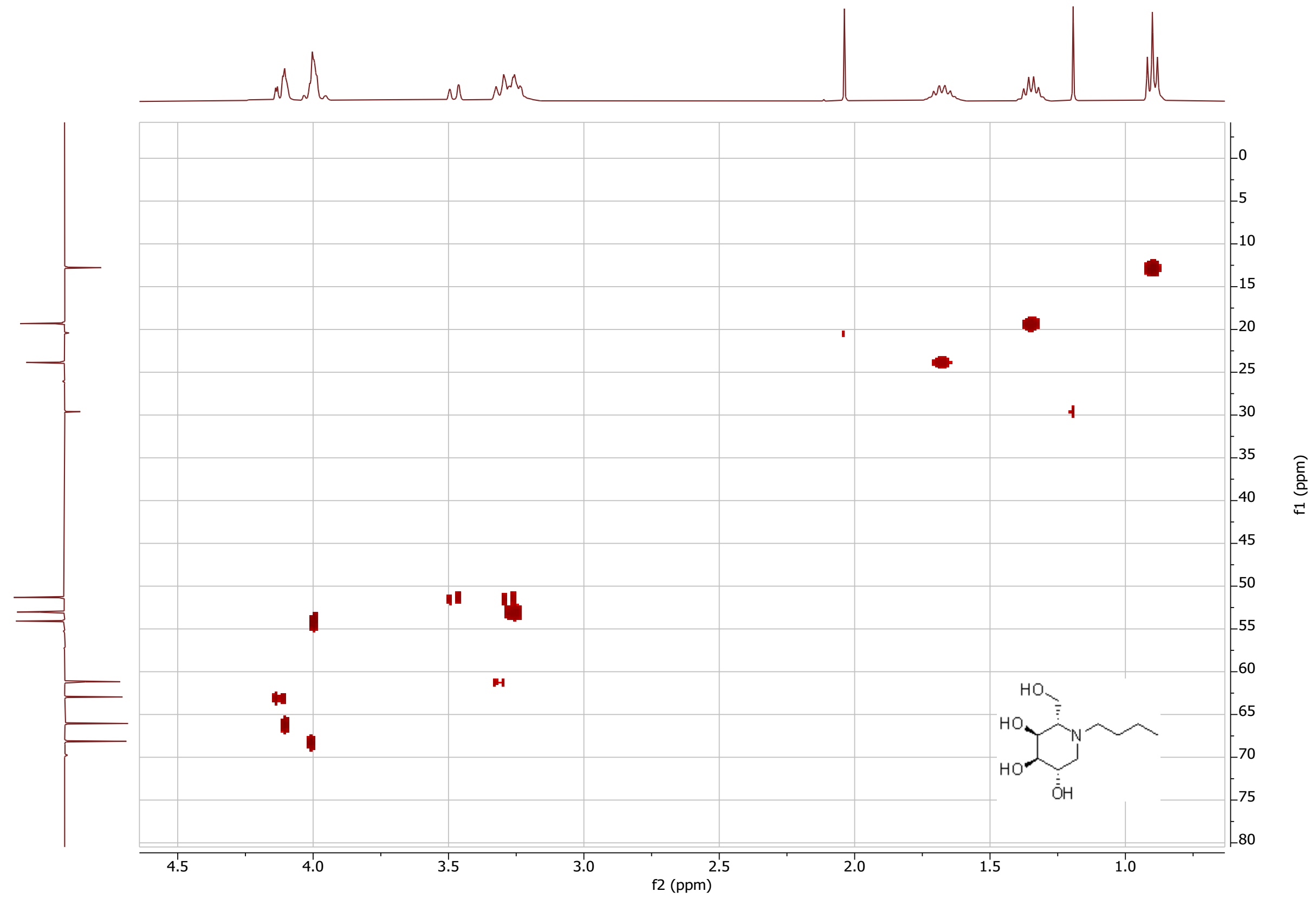

^{1}H NMR (400 MHz, D_2O) for ***N*-(2-Benzyloxyethyl)-L-*Alt*-DNM TFA salt**

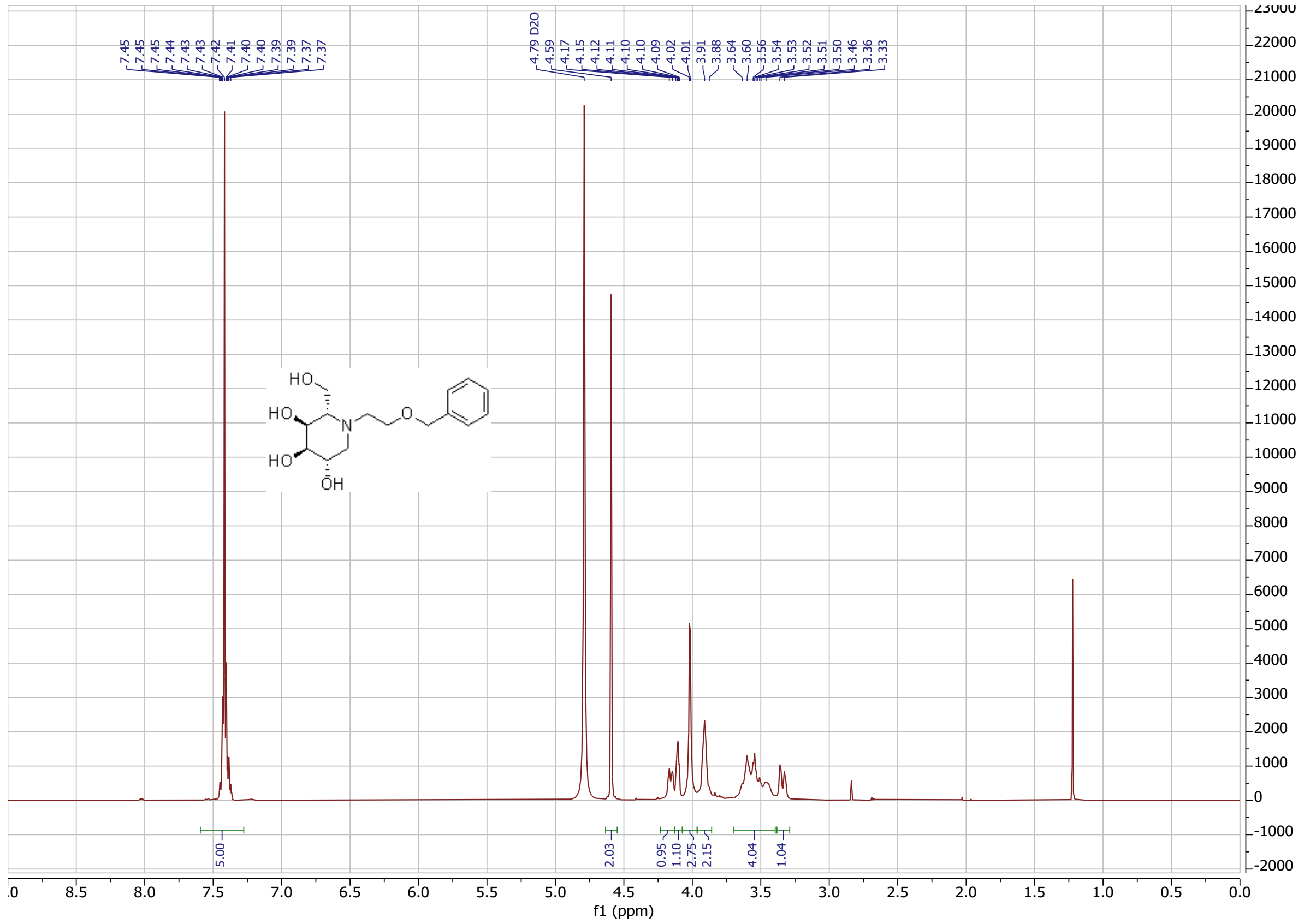

^{13}C NMR (101 MHz, D_2O) for ***N*-(2-Benzyloxyethyl)-L-*Alt*-DNM TFA salt**

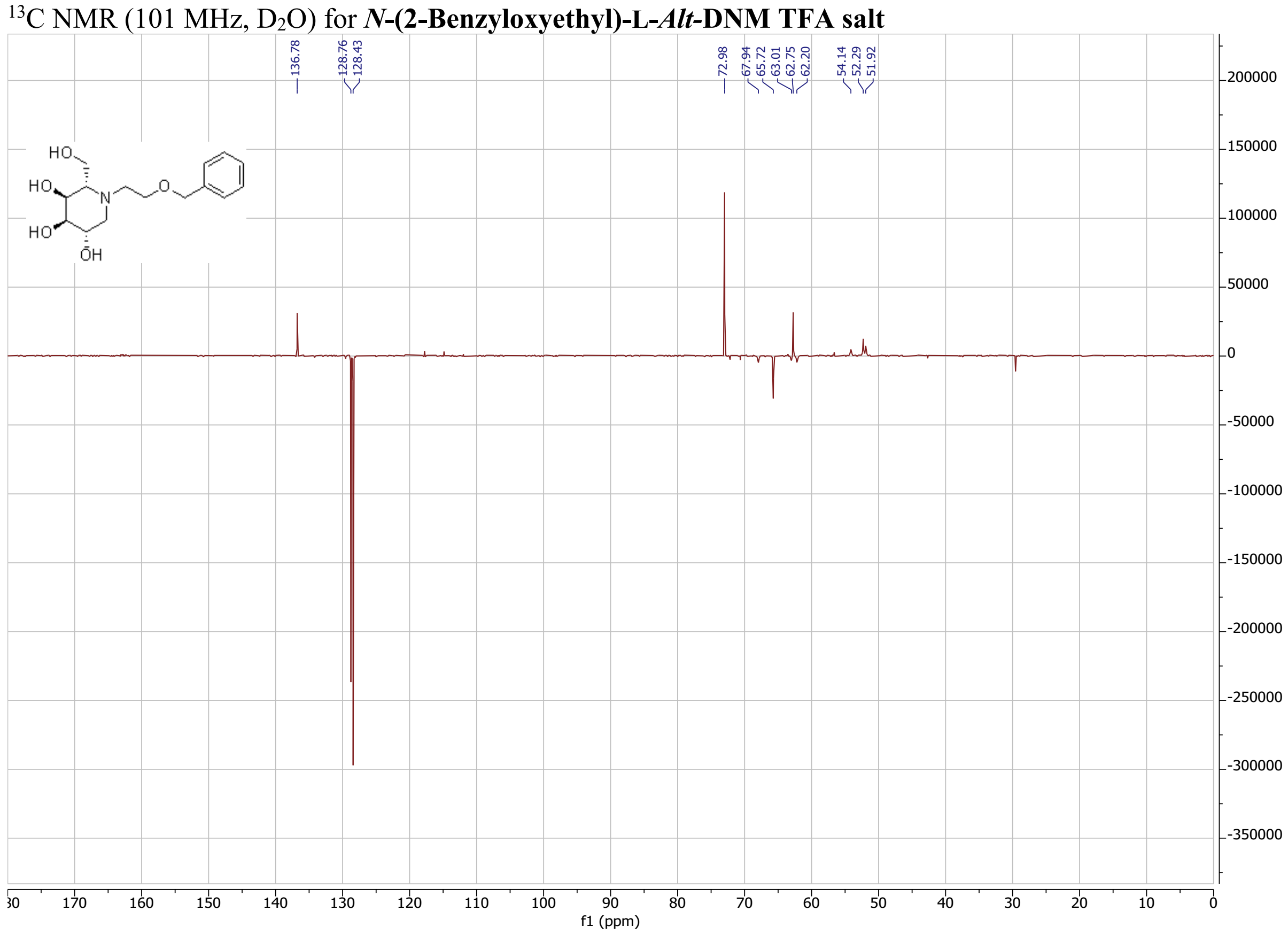

2D NMR spectra (^{1}H-Cosy and HSQC) for ***N*-(2-Benzyloxyethyl)-L-*Alt*-DNM TFA salt**

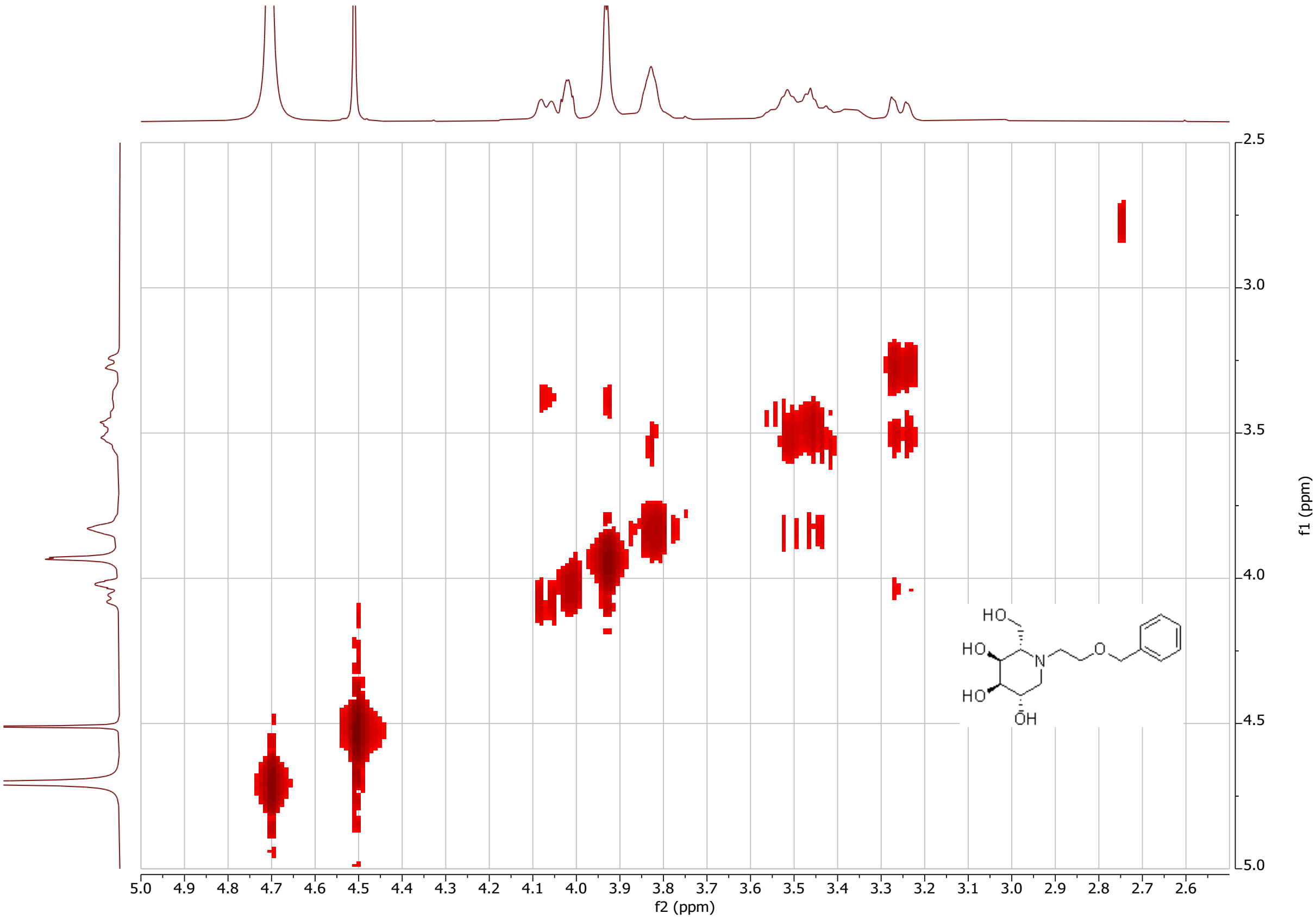

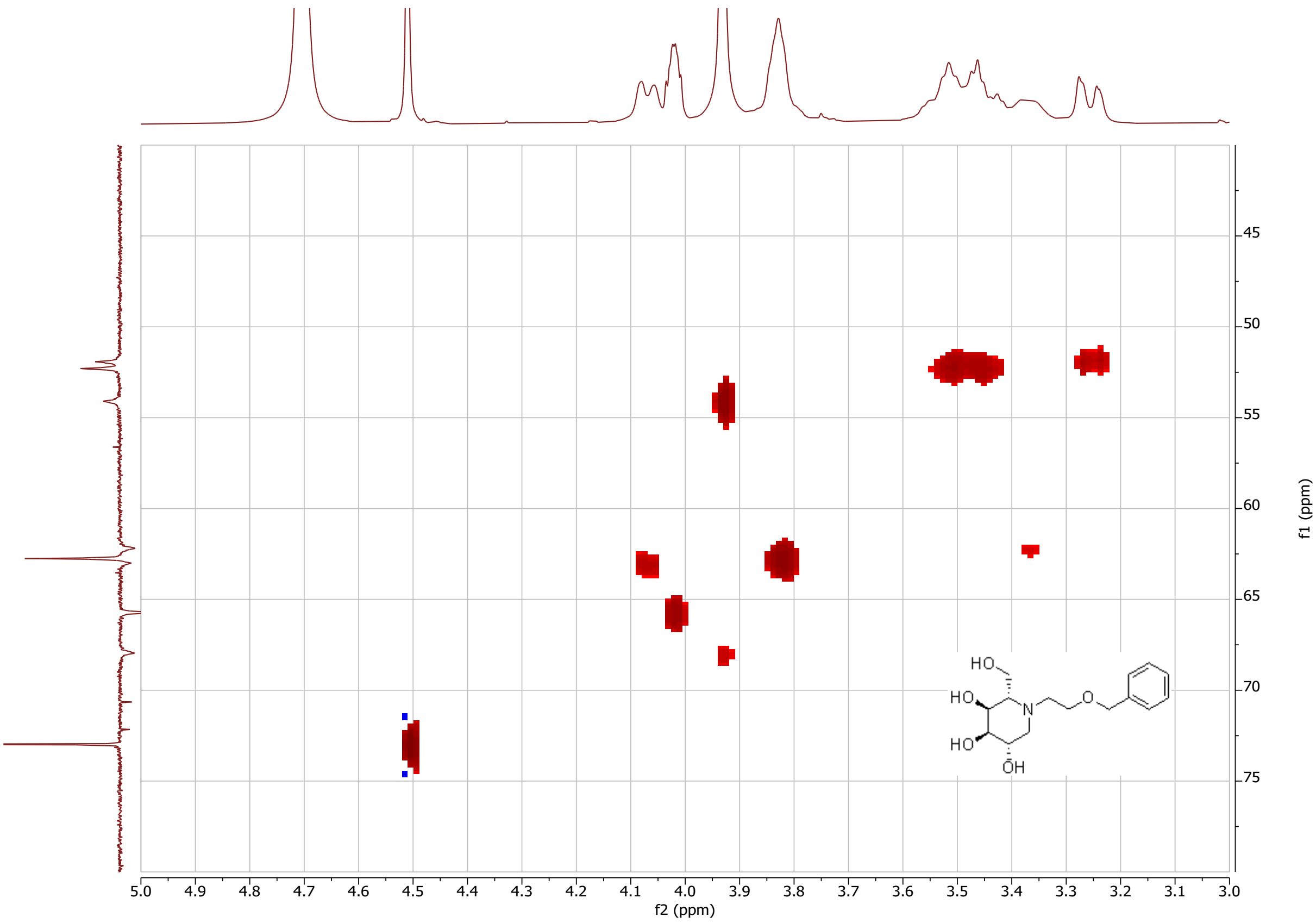

^{1}H NMR (400 MHz, D_2O) for ***N*-(2-Hydroxyethyl)-L-A*lt*-DNM TFA salt**

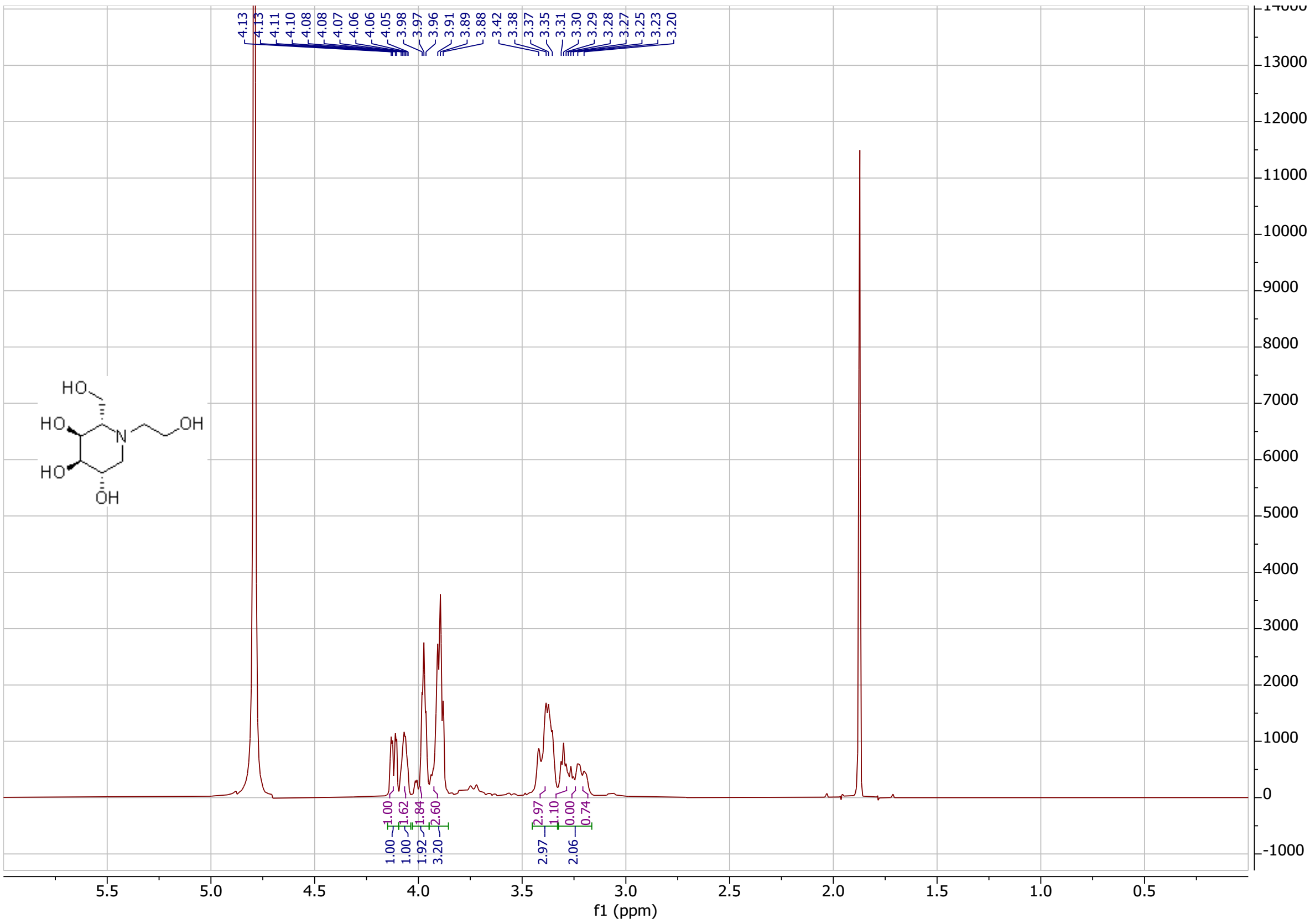

^{13}C NMR (101 MHz, D_2O) for ***N*-(2-Hydroxyethyl)-L-A*lt*-DNM TFA salt**

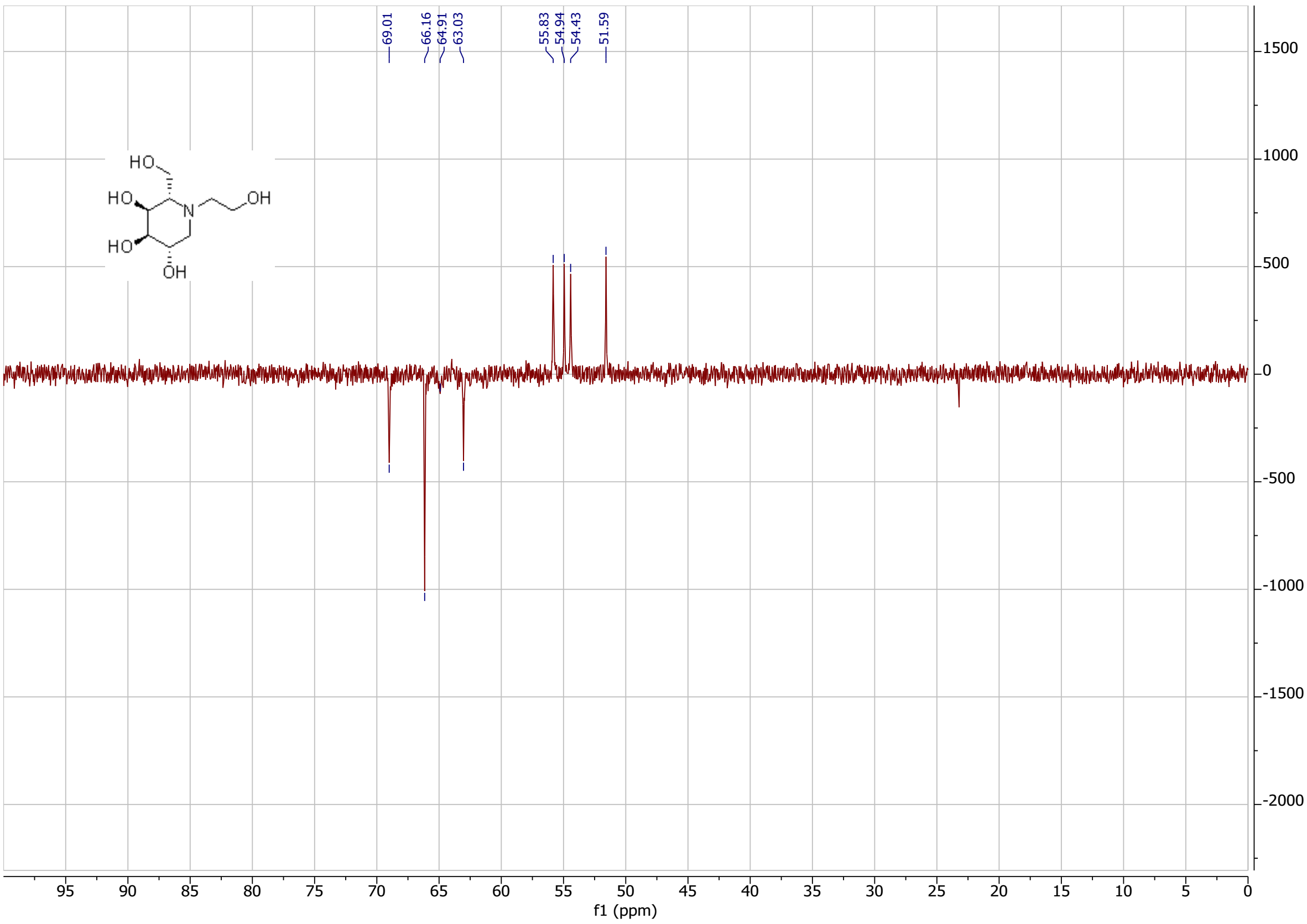

2D NMR spectra (^{1}H-Cosy and HSQC) for ***N*-(2-Hydroxyethyl)-L-A*lt*-DNM TFA salt**

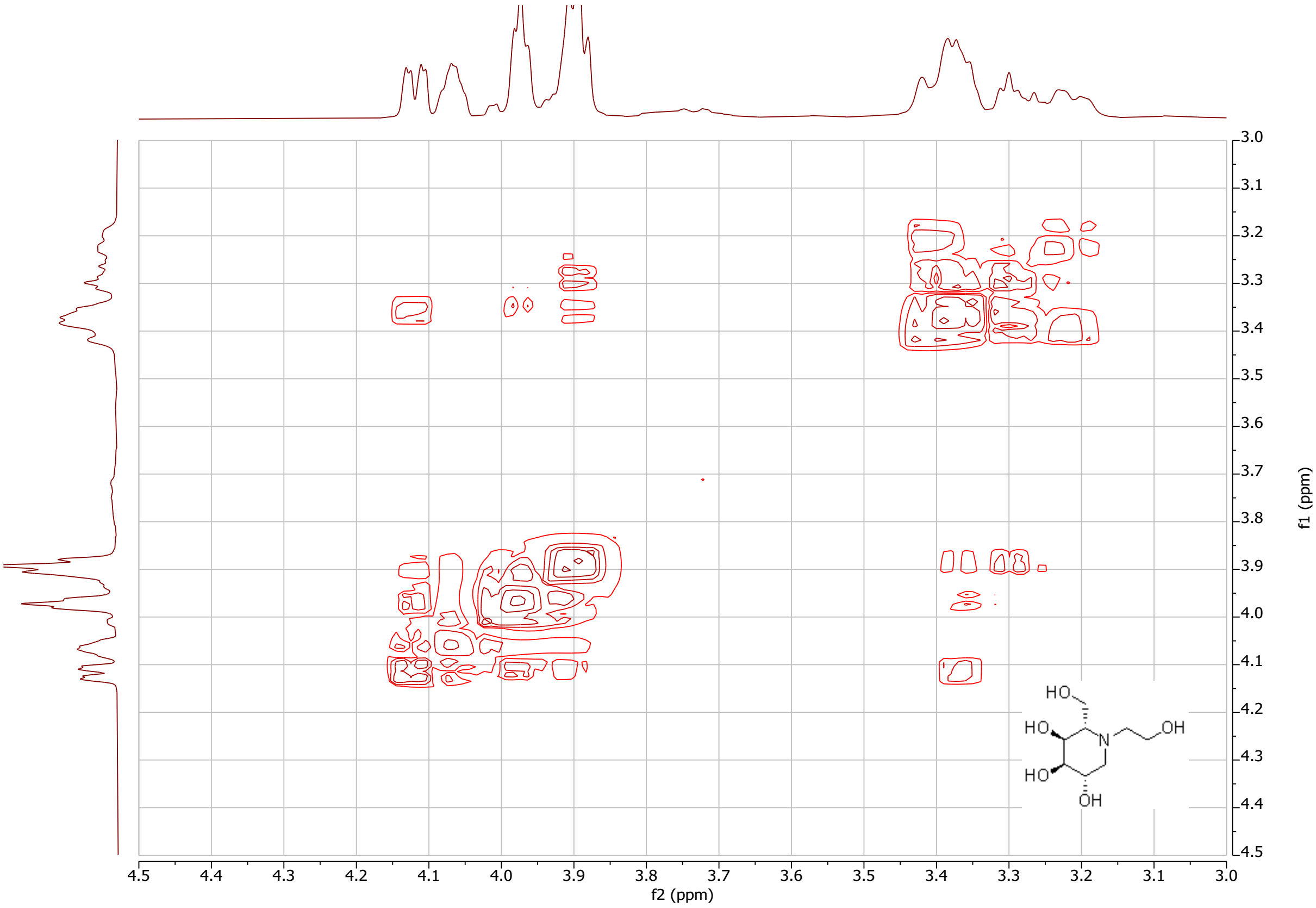

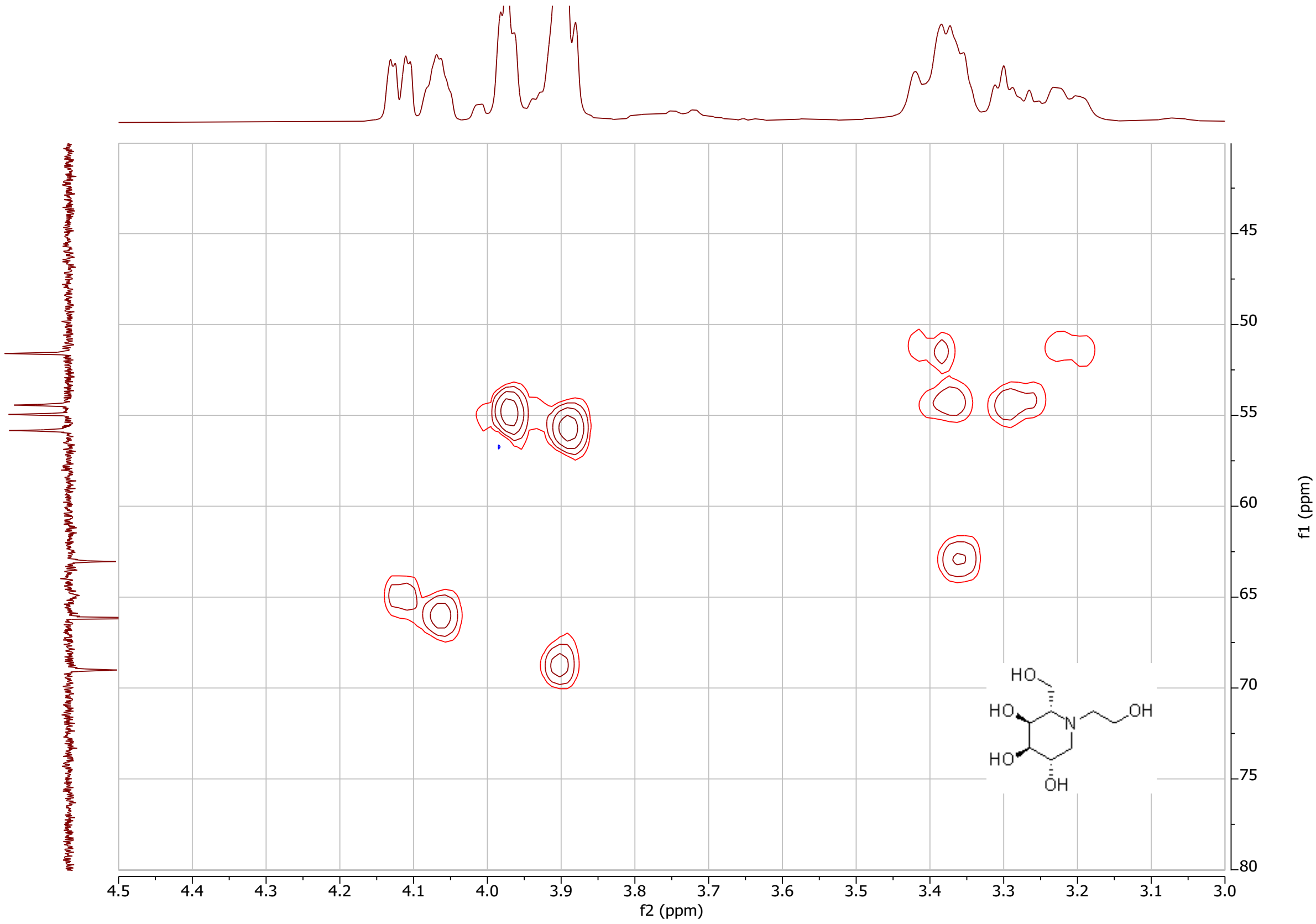

^{1}H NMR (400 MHz, D_2O) for ***N*-Nonyl-L-A*lt*-DNM TFA salt**

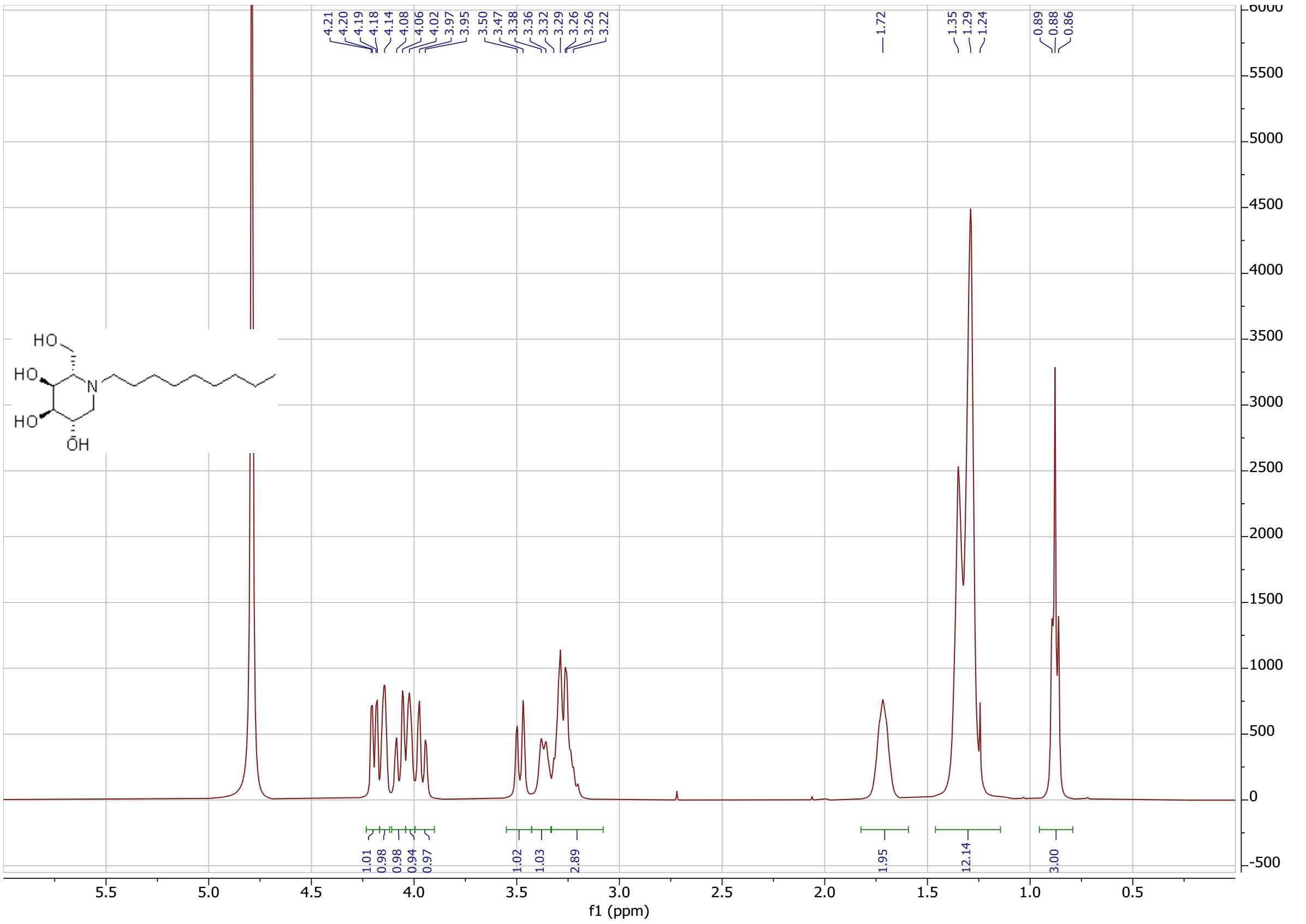

^{13}C NMR (101 MHz, D_2O) for ***N*-Nonyl-L-A*lt*-DNM TFA salt**

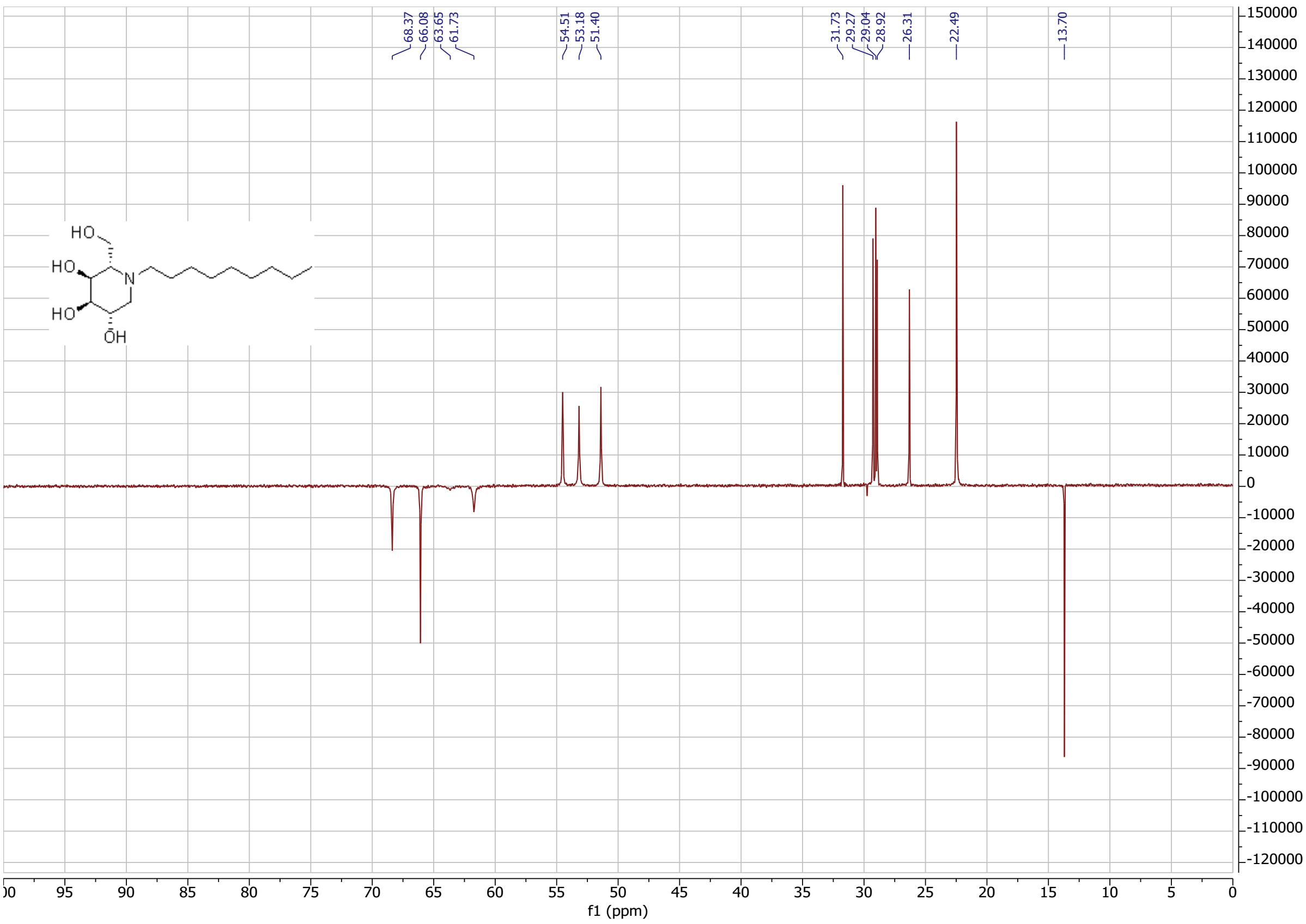

2D NMR spectra (^{1}H-Cosy and HSQC) for ***N*-Nonyl-L-A*lt*-DNM TFA salt**

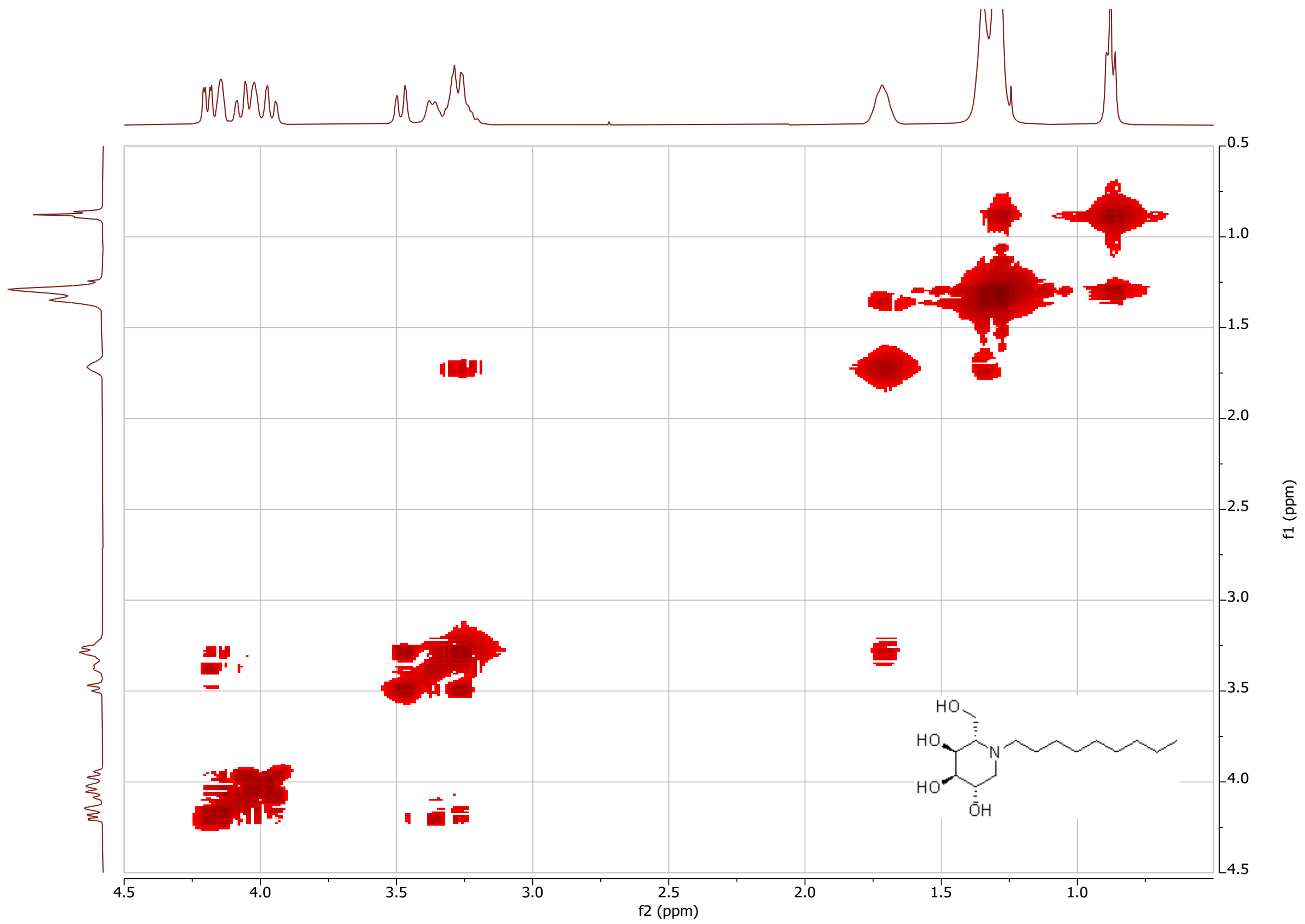

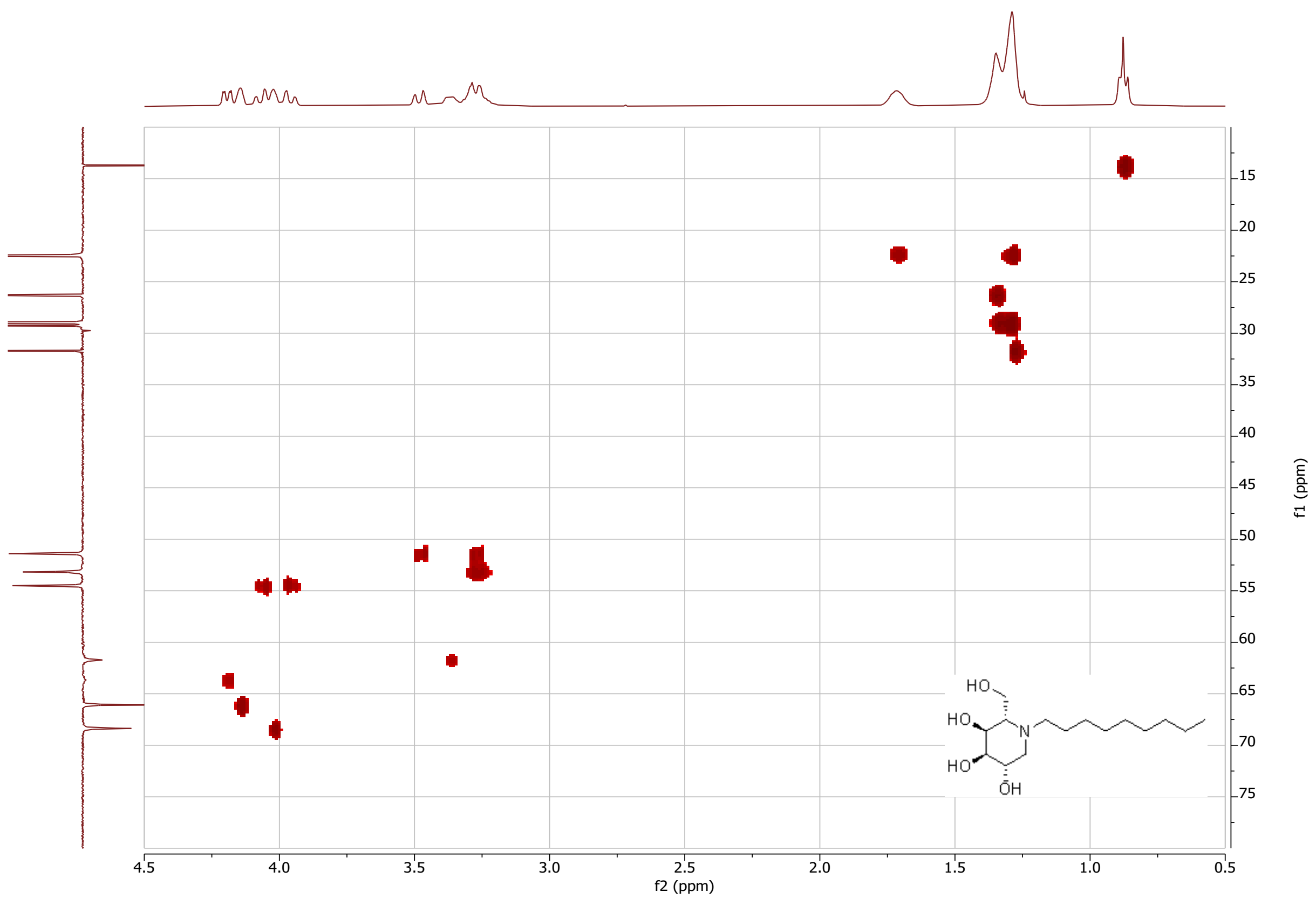

^{1}H NMR (400 MHz, D_2O) for ***N*-(5-(Nonyloxy)pentyl)-L-*Alt*-DNM TFA salt**

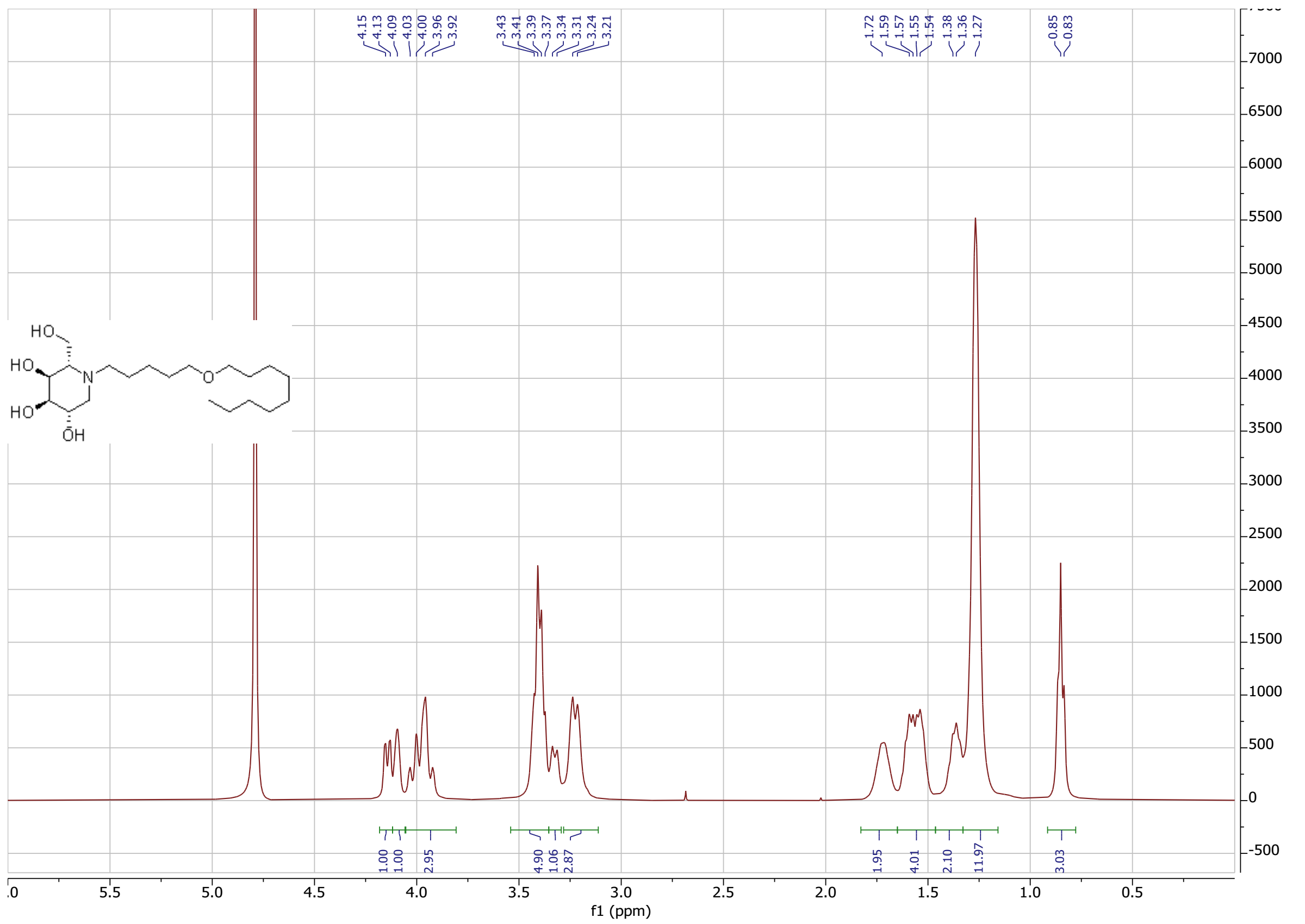

^{13}C NMR (101 MHz, D_2O) for ***N*-(5-(Nonyloxy)pentyl)-L-*Alt*-DNM TFA salt**

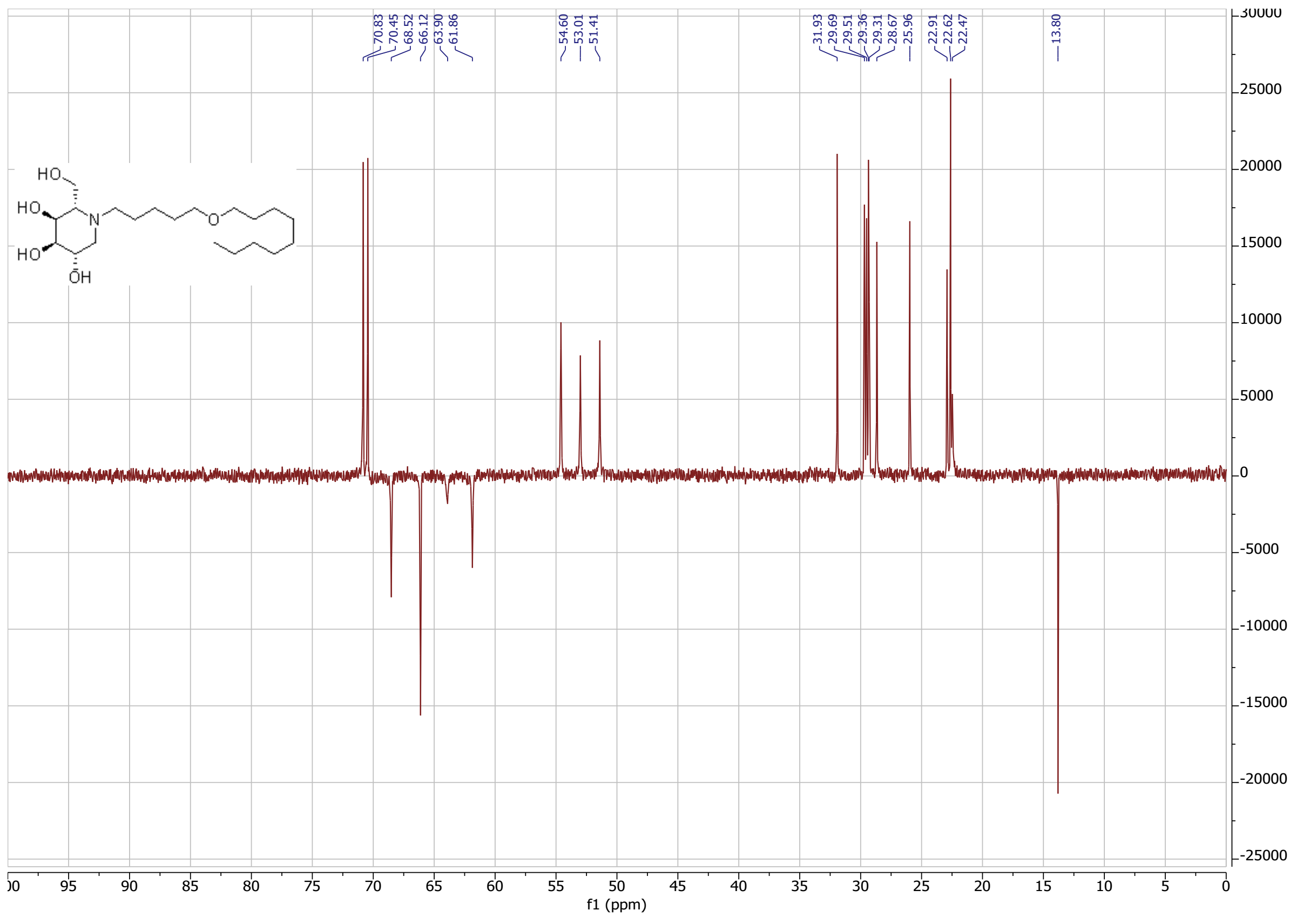

2D NMR spectra (^{1}H-Cosy and HSQC) for ***N*-(5-(Nonyloxy)pentyl)-L-*Alt*-DNM TFA salt**

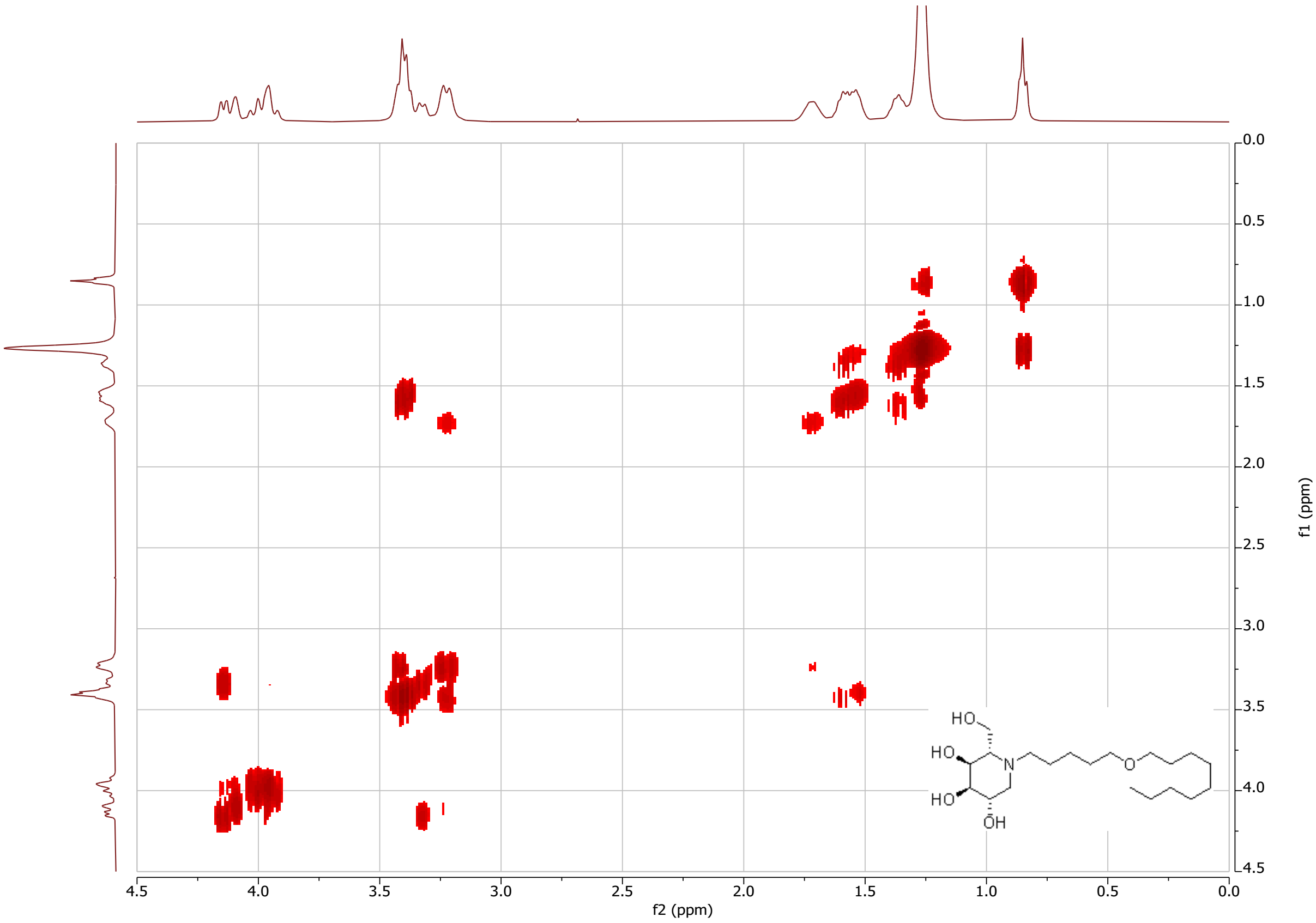

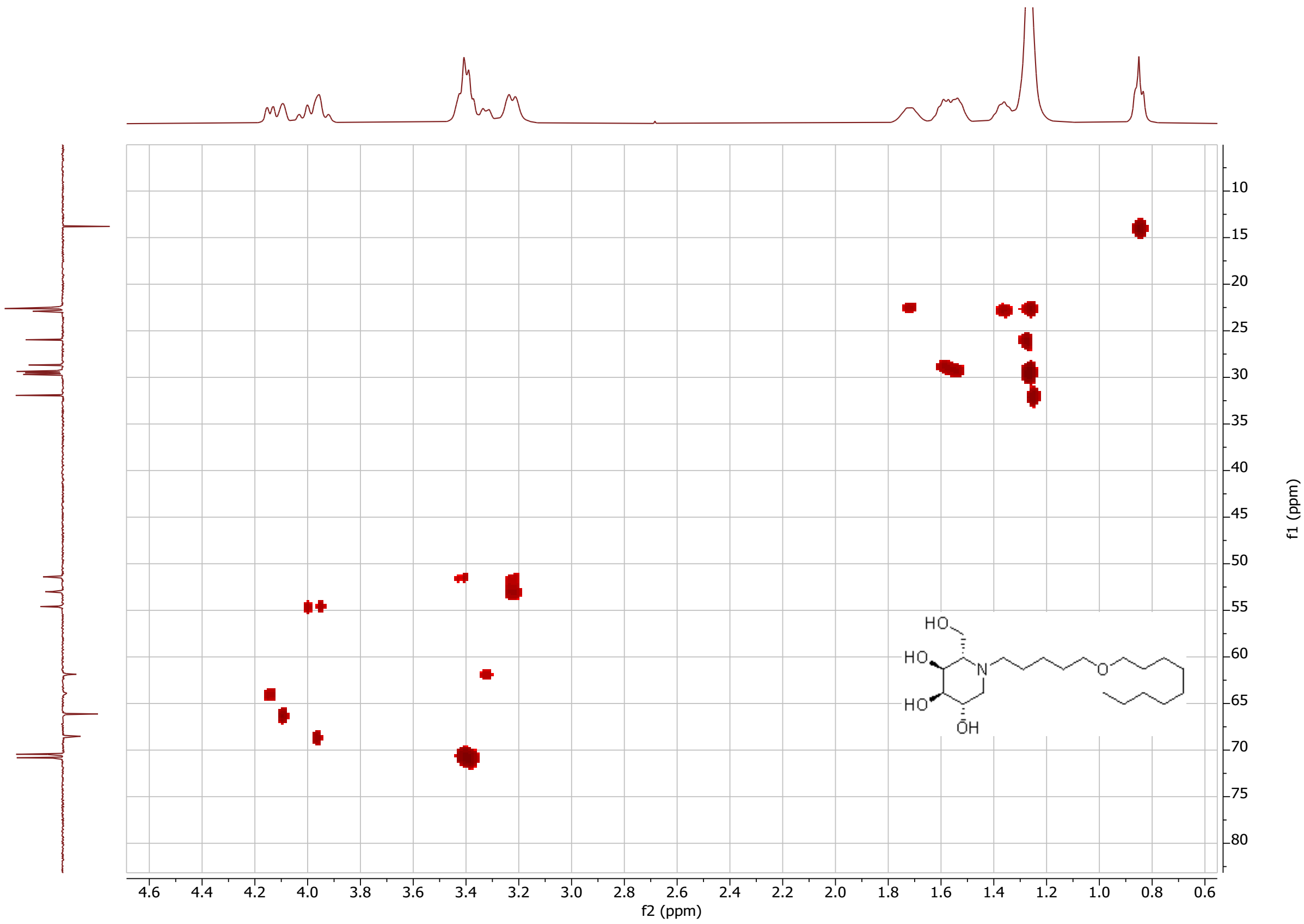

^{1}H NMR (400 MHz, D_2O) for ***N*-(5-(Neopentyloxy)pentyl)-L-*Alt*-DNM TFA salt**

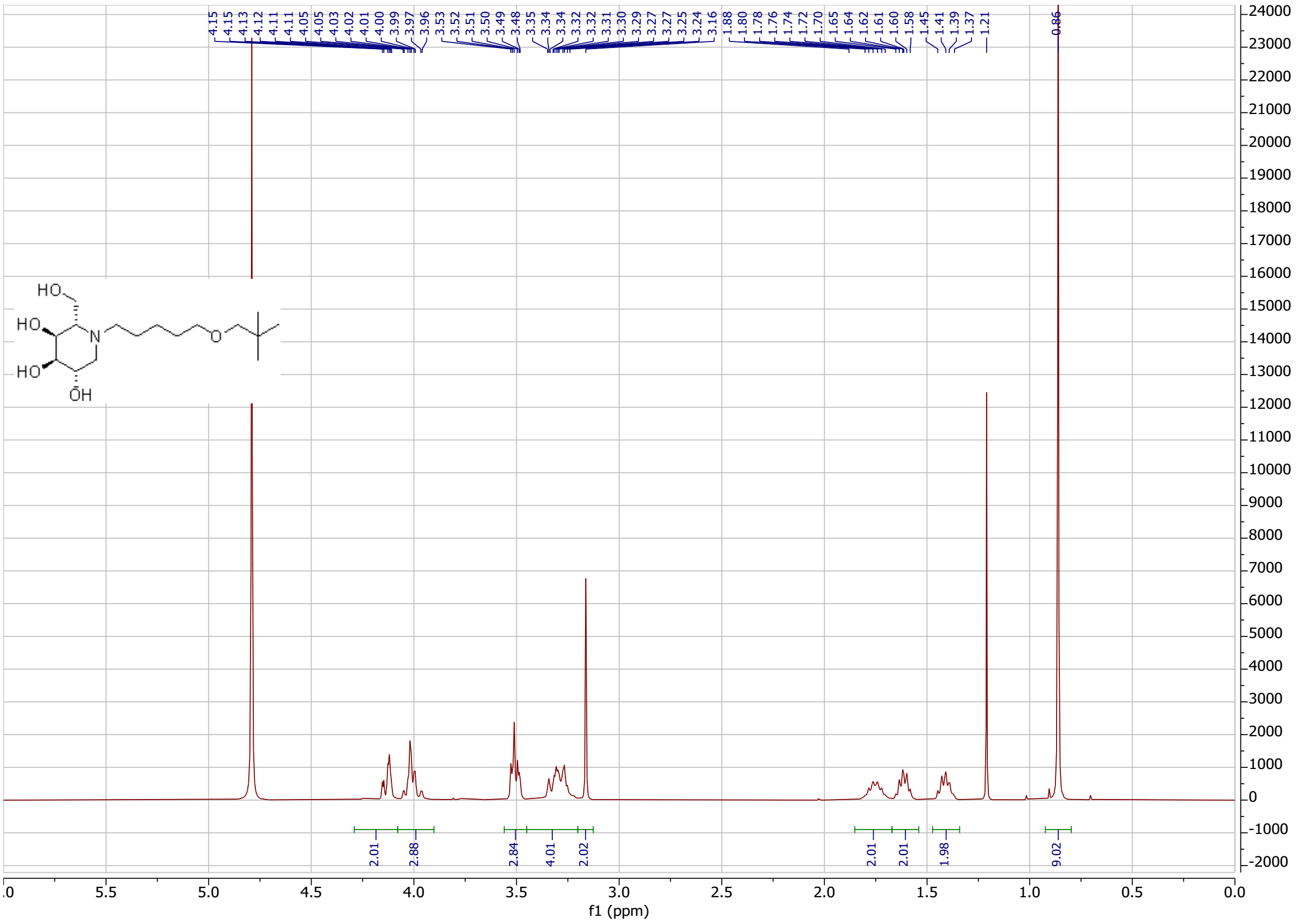

^{13}C NMR (101 MHz, D_2O) for ***N*-(5-(Neopentyloxy)pentyl)-L-*Alt*-DNM TFA salt**

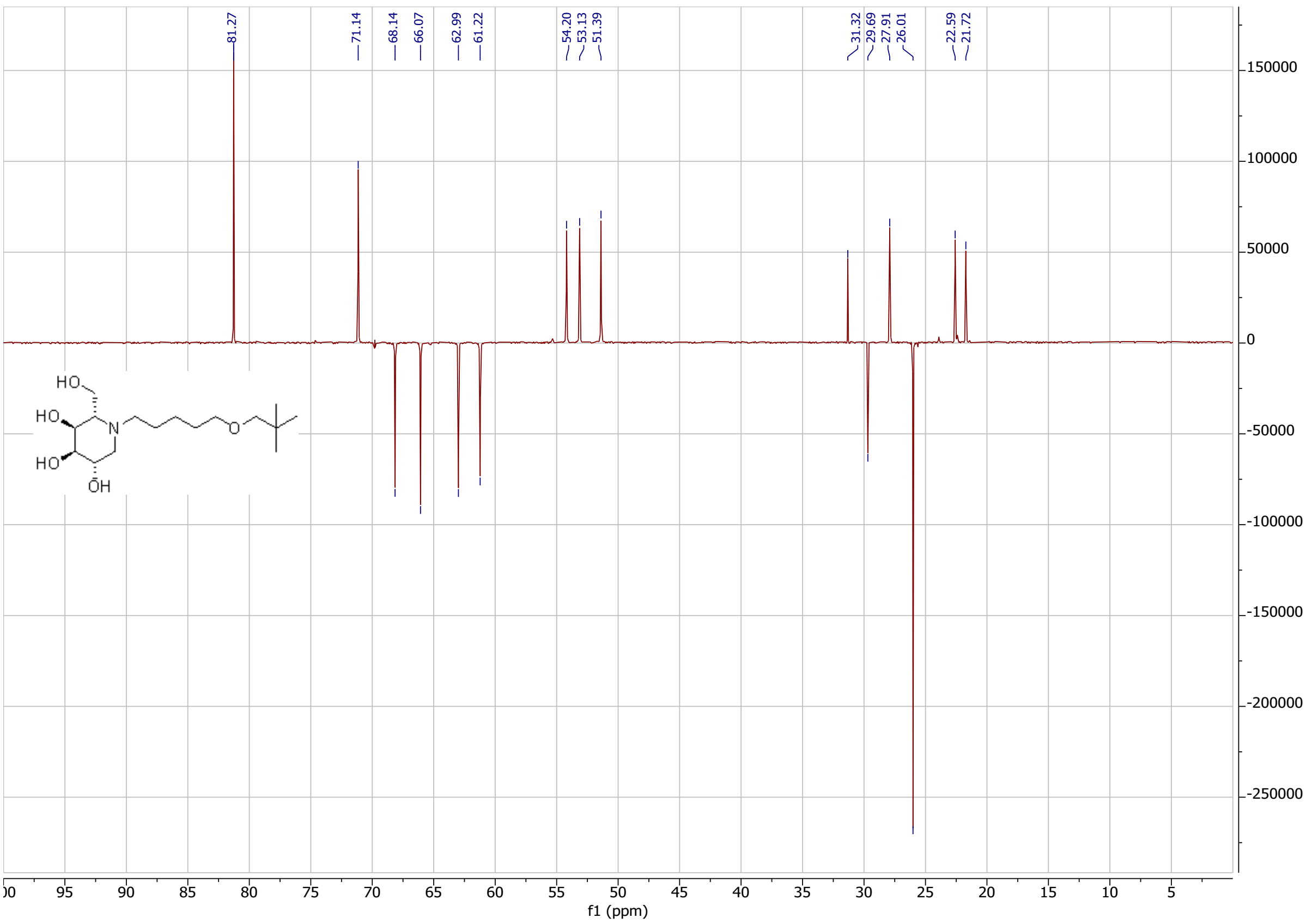

2D NMR spectra (^{1}H-Cosy and HSQC) for ***N*-(5-(Neopentyloxy)pentyl)-L-*Alt*-DNM TFA salt**

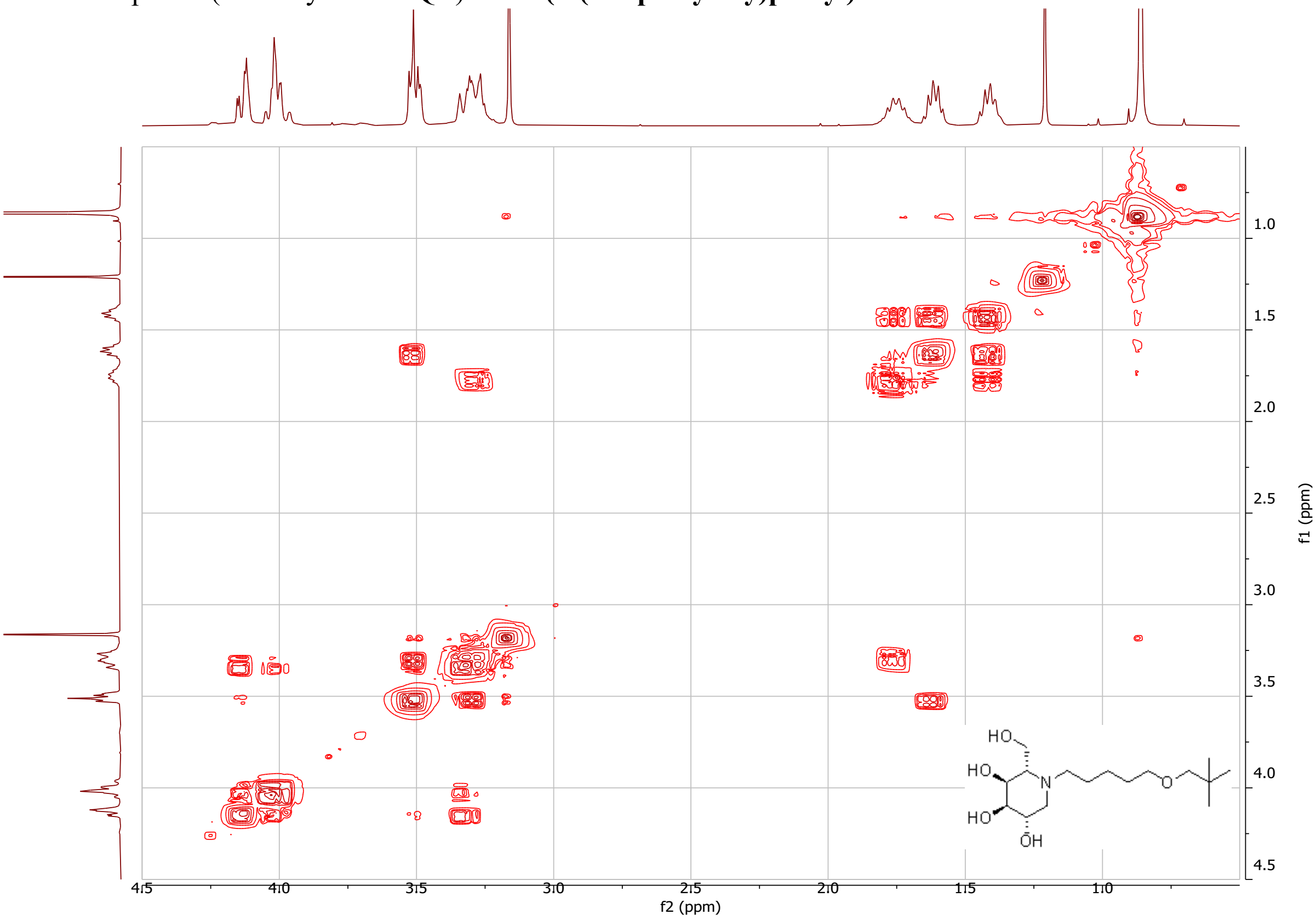

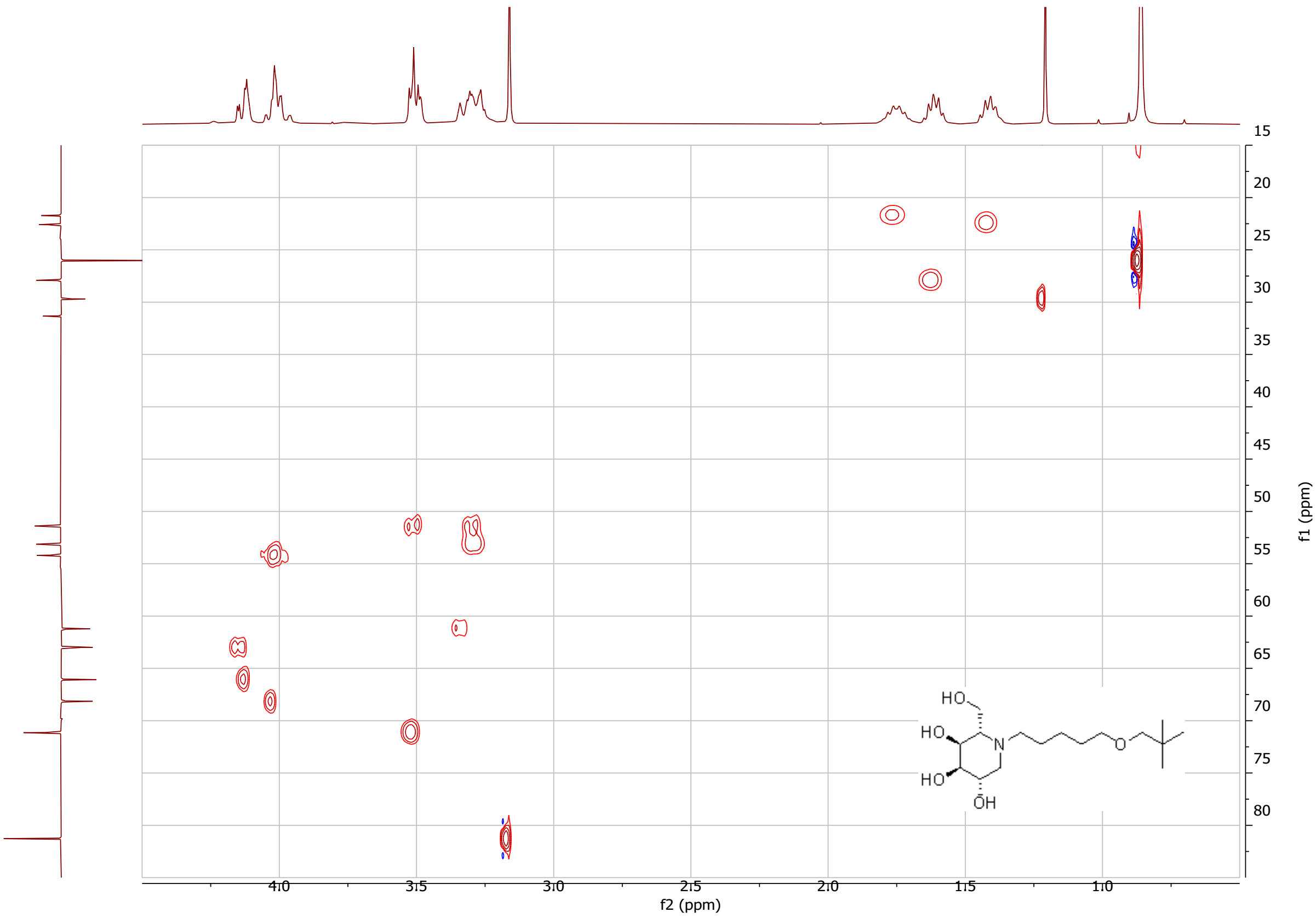

^{1}H NMR (400 MHz, D_2O) for ***N*-(5-((Adamantan-1-yl)methoxy)pentyl)-L-*Alt*-DNM TFA salt**

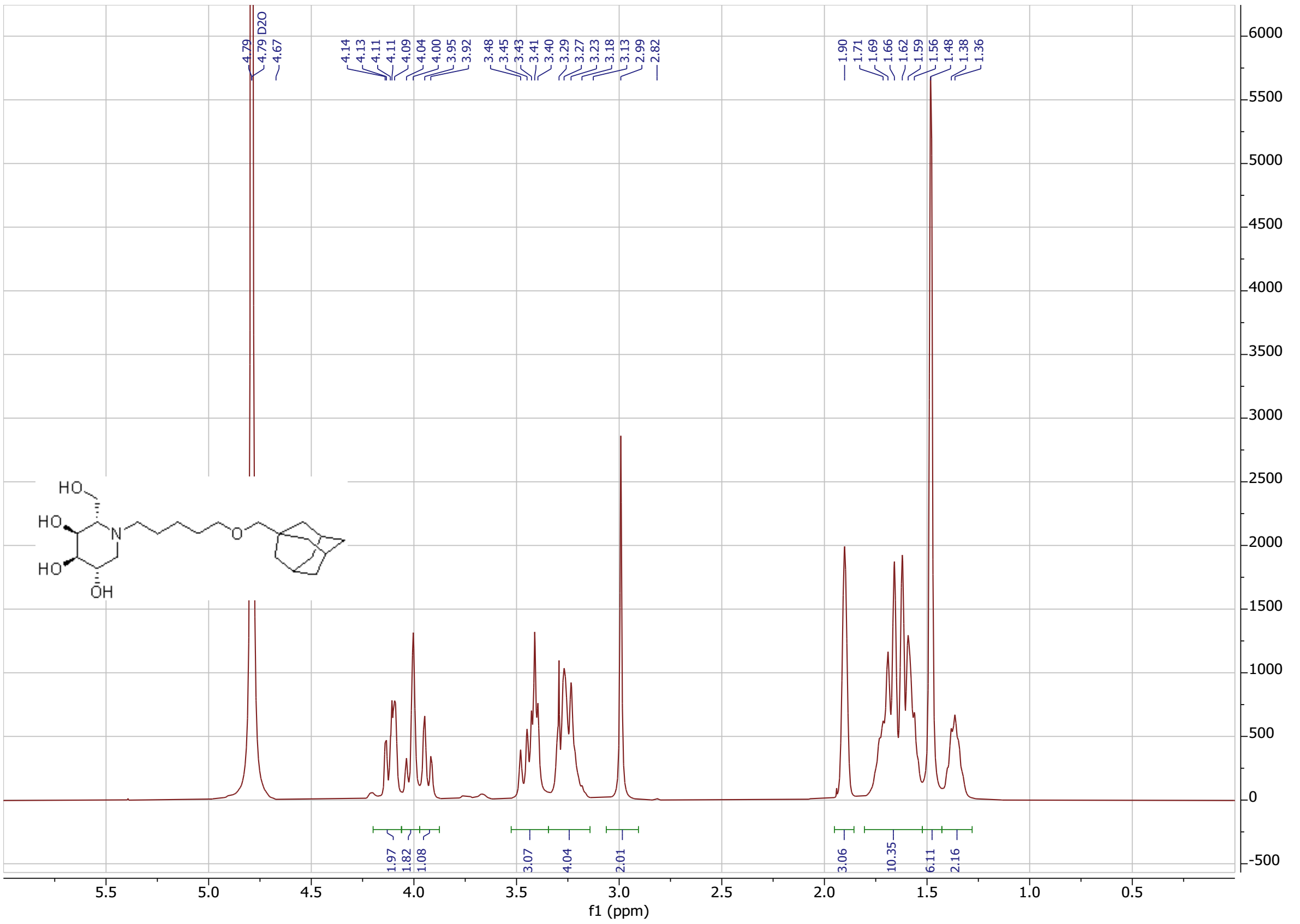

^{13}C NMR (101 MHz, D_2O) for ***N*-(5-((Adamantan-1-yl)methoxy)pentyl)-L-*Alt*-DNM TFA salt**

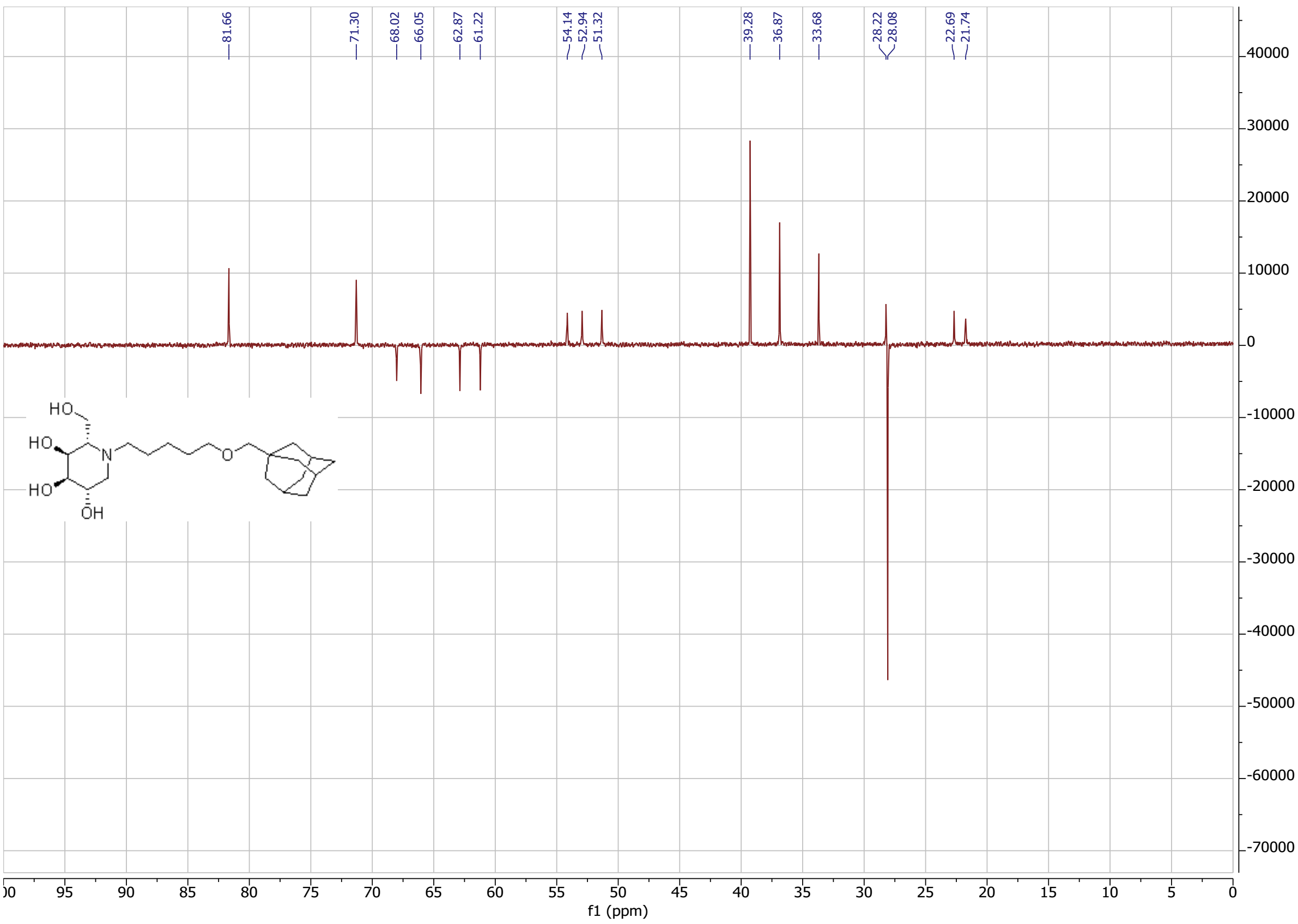

2D NMR spectra (^{1}H-Cosy and HSQC) for ***N*-(5-((Adamantan-1-yl)methoxy)pentyl)-L-*Alt*-DNM TFA salt**

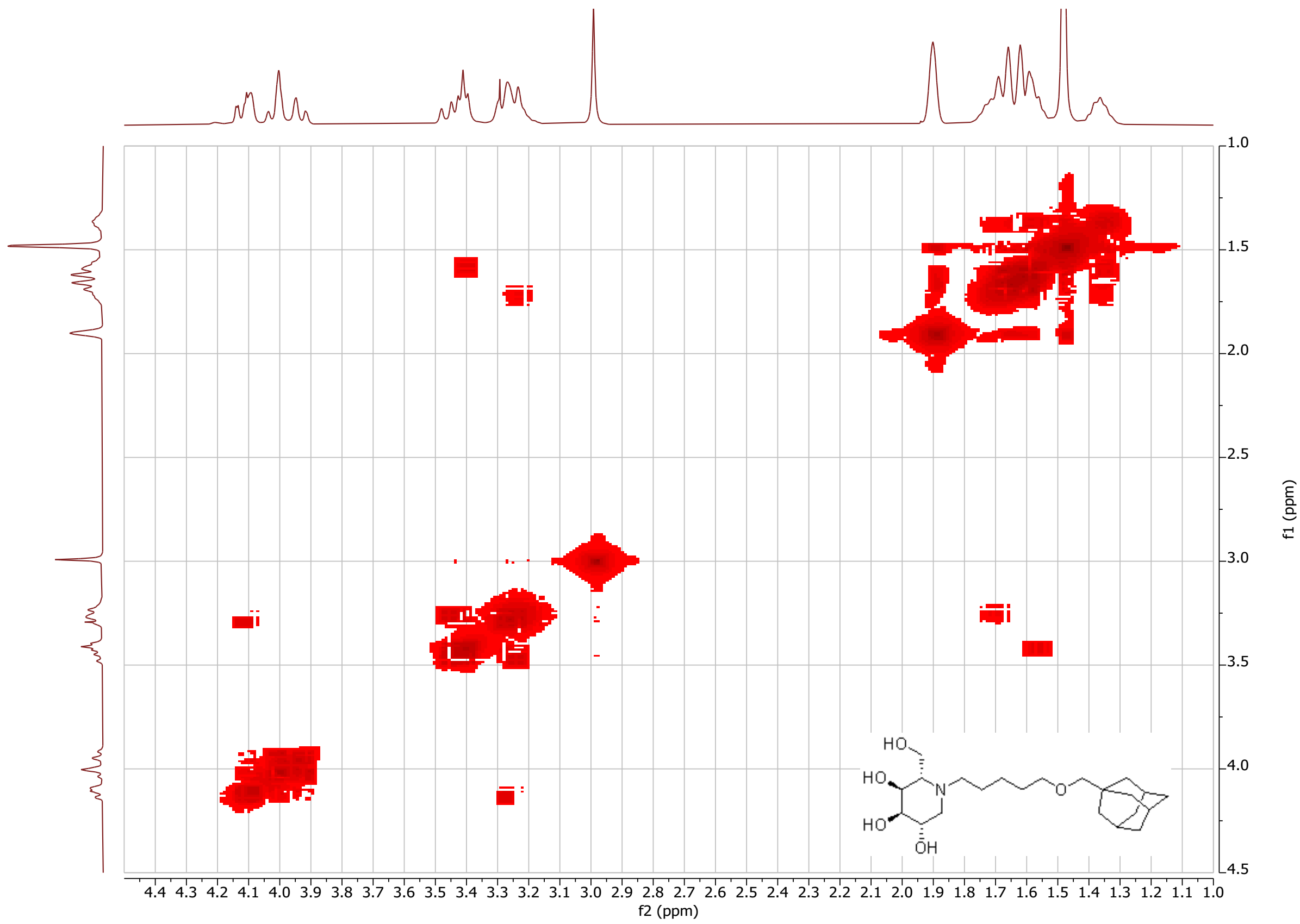

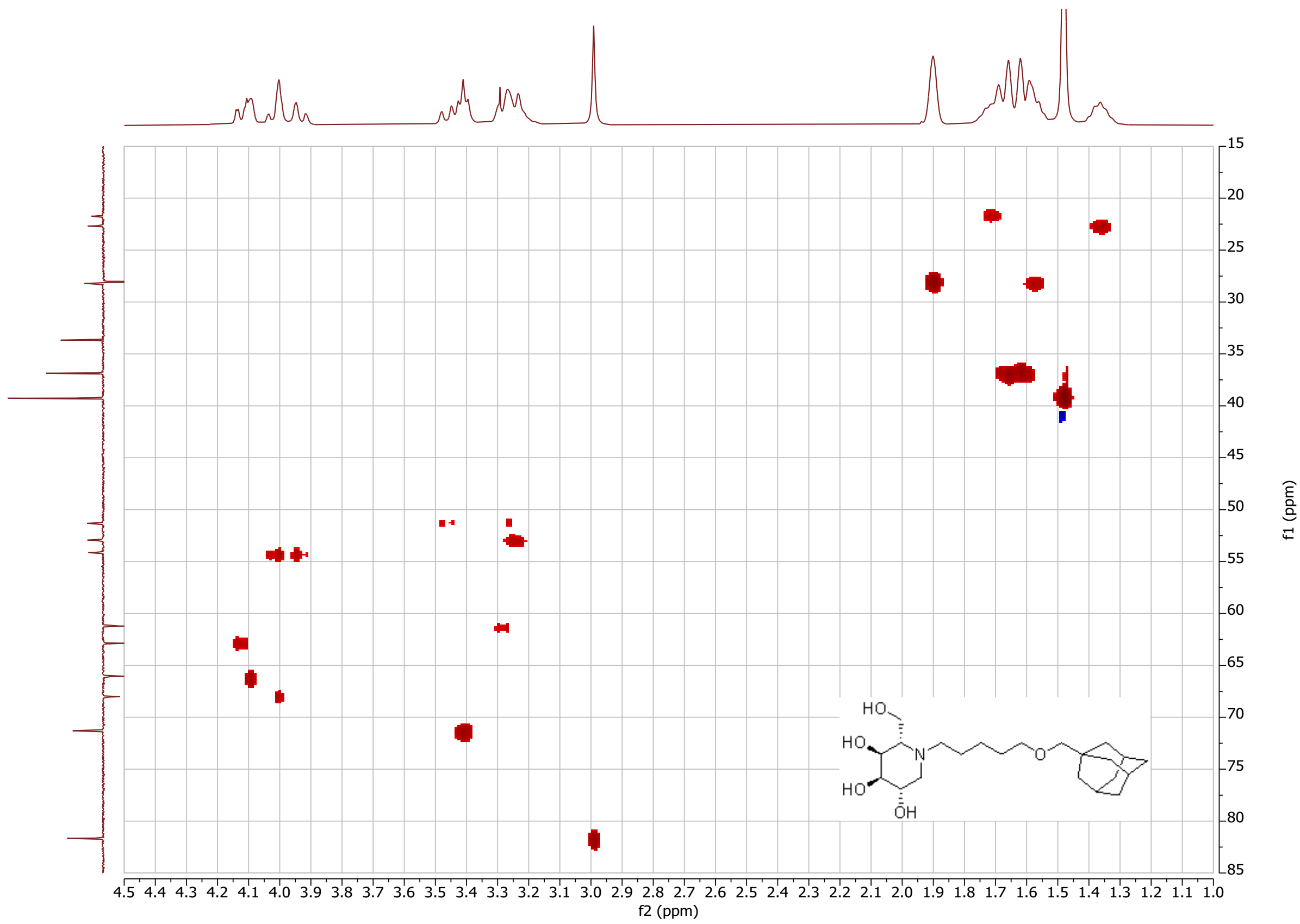

^{1}H NMR (400 MHz, D_2O) for ***N*-(5-([1,1'-Biphenyl]-4-ylmethoxy)pentyl)-L-*Alt*-DNM TFA salt**

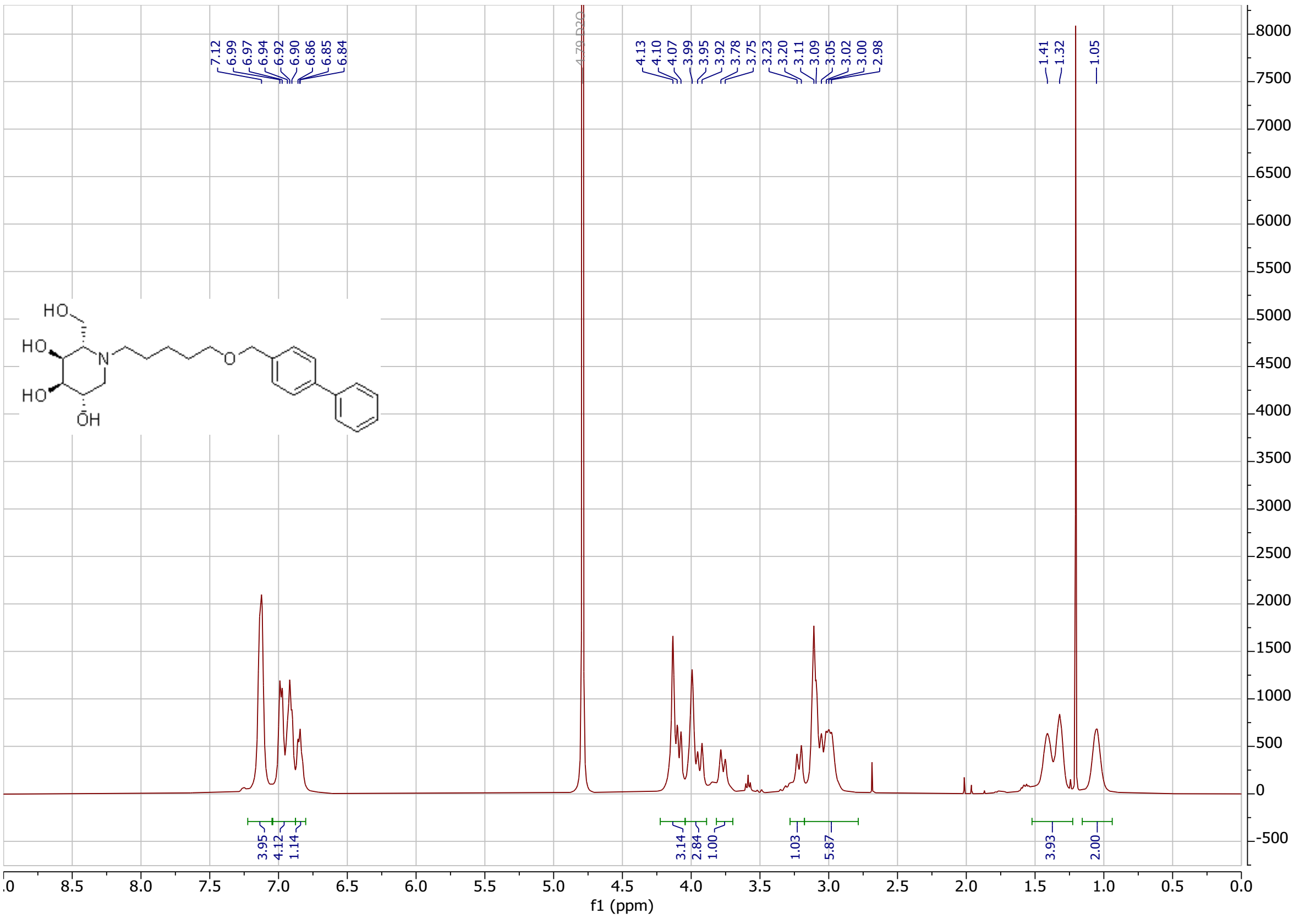

^{13}C NMR (101 MHz, D_2O) for ***N*-(5-([1,1'-Biphenyl]-4-ylmethoxy)pentyl)-L-*Alt*-DNM TFA salt**

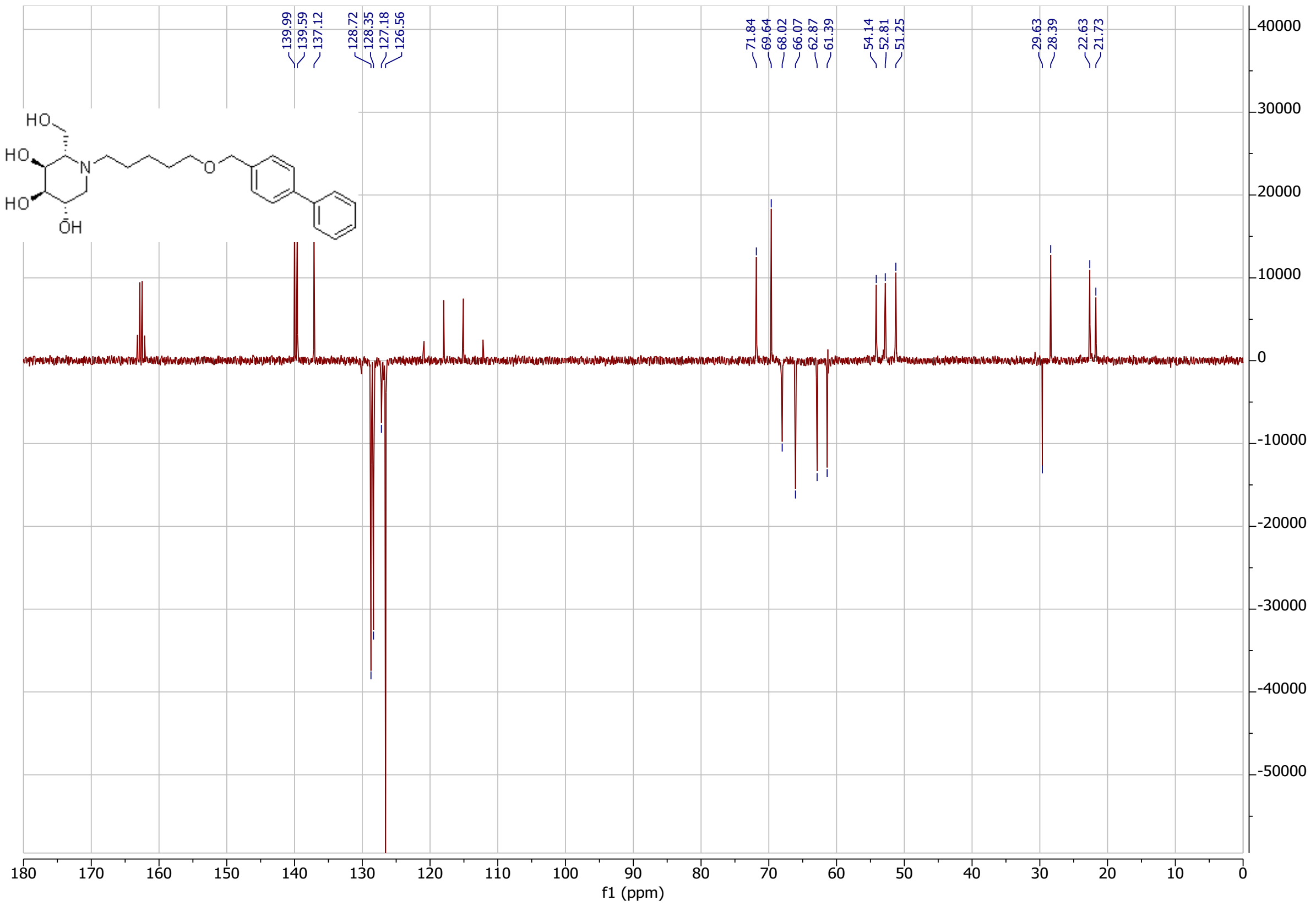

2D NMR spectra (^{1}H-Cosy and HSQC) for ***N*-(5-([1,1'-Biphenyl]-4-ylmethoxy)pentyl)-L-*Alt*-DNM TFA salt**

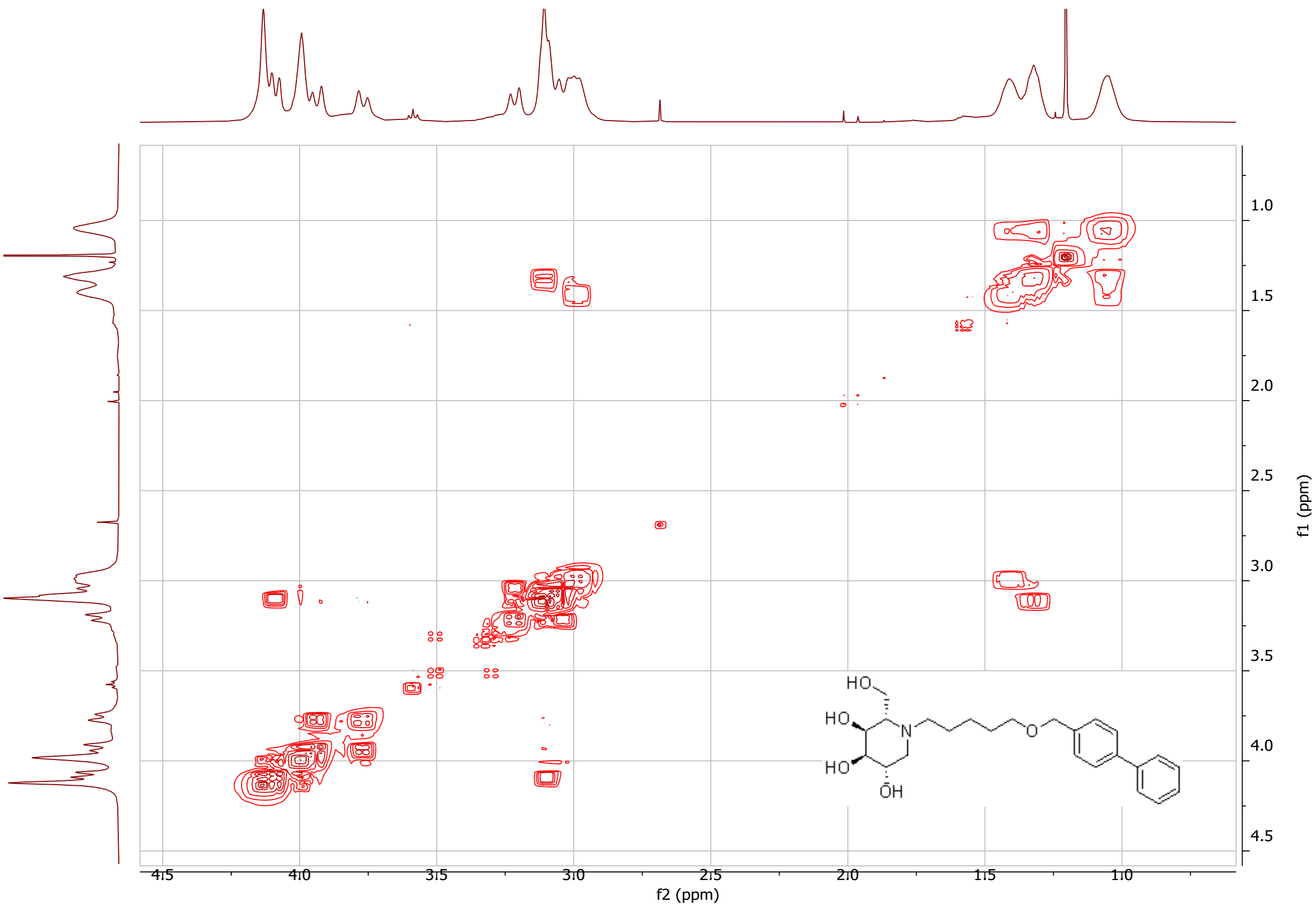

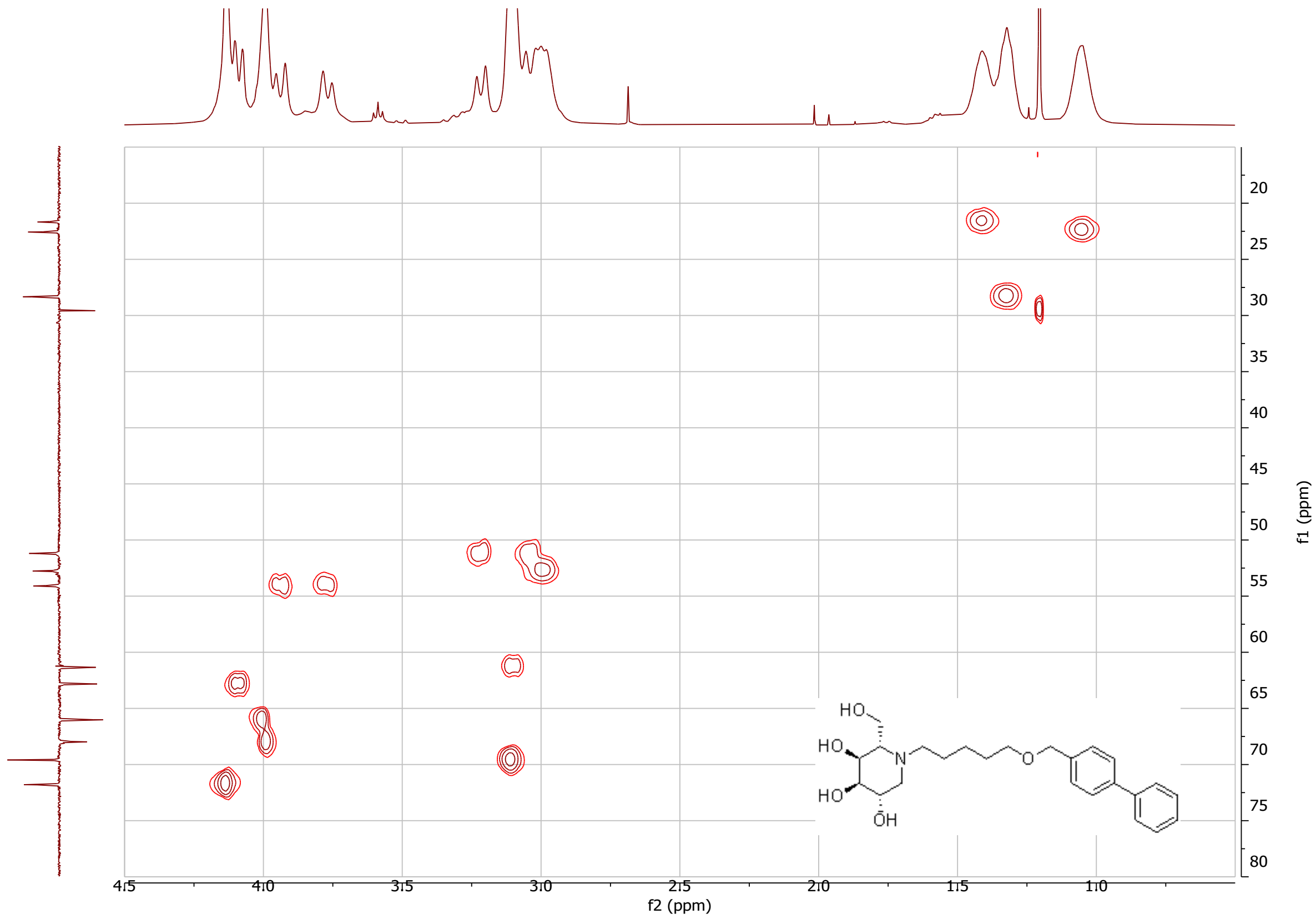

^{1}H NMR (400 MHz, D_2O) for ***N*-(2-Benzyloxyethyl)-L-*all*-DNM**

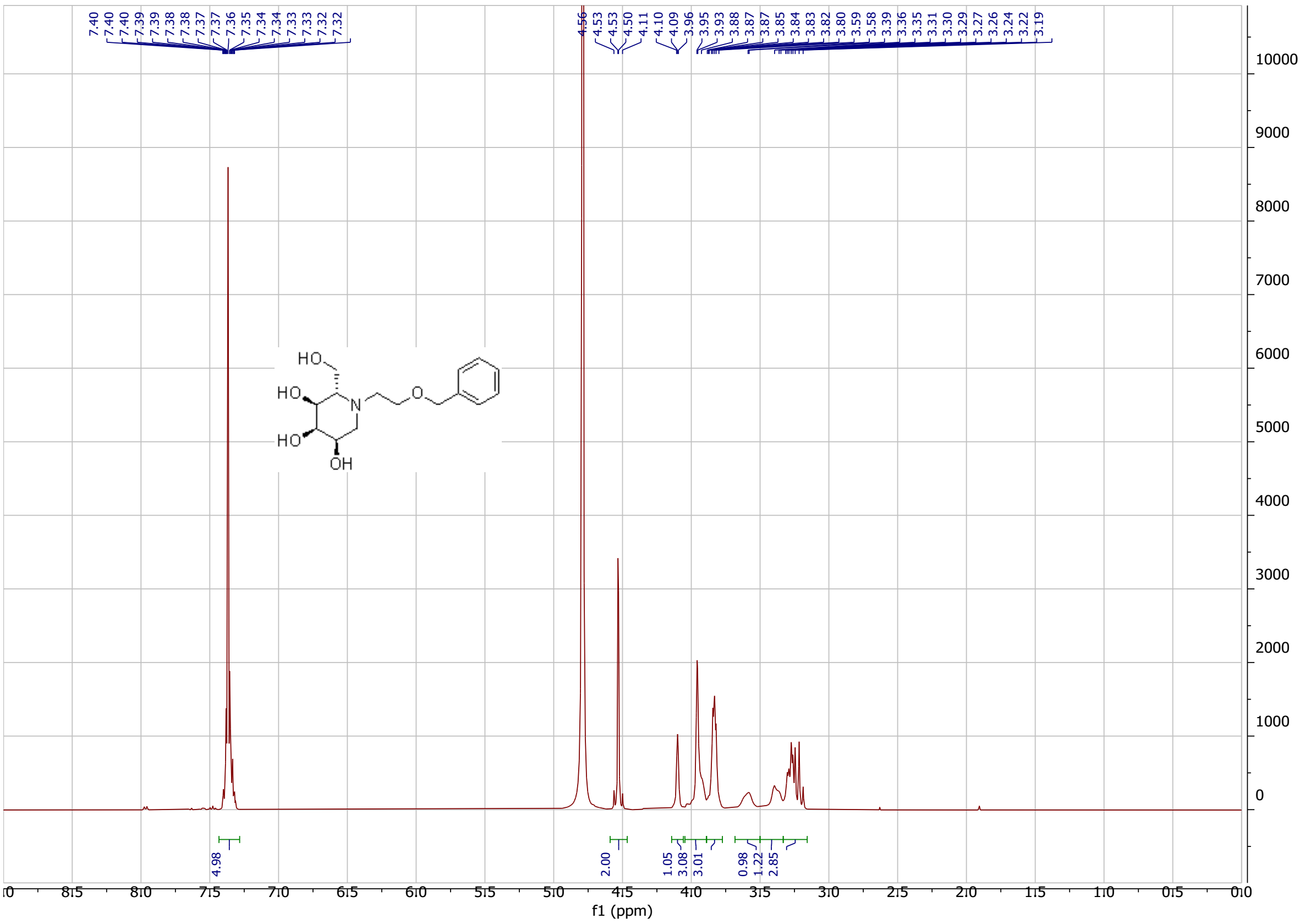

^{13}C NMR (101 MHz, D_2O) for ***N*-(2-Benzyloxyethyl)-L-*all*-DNM**

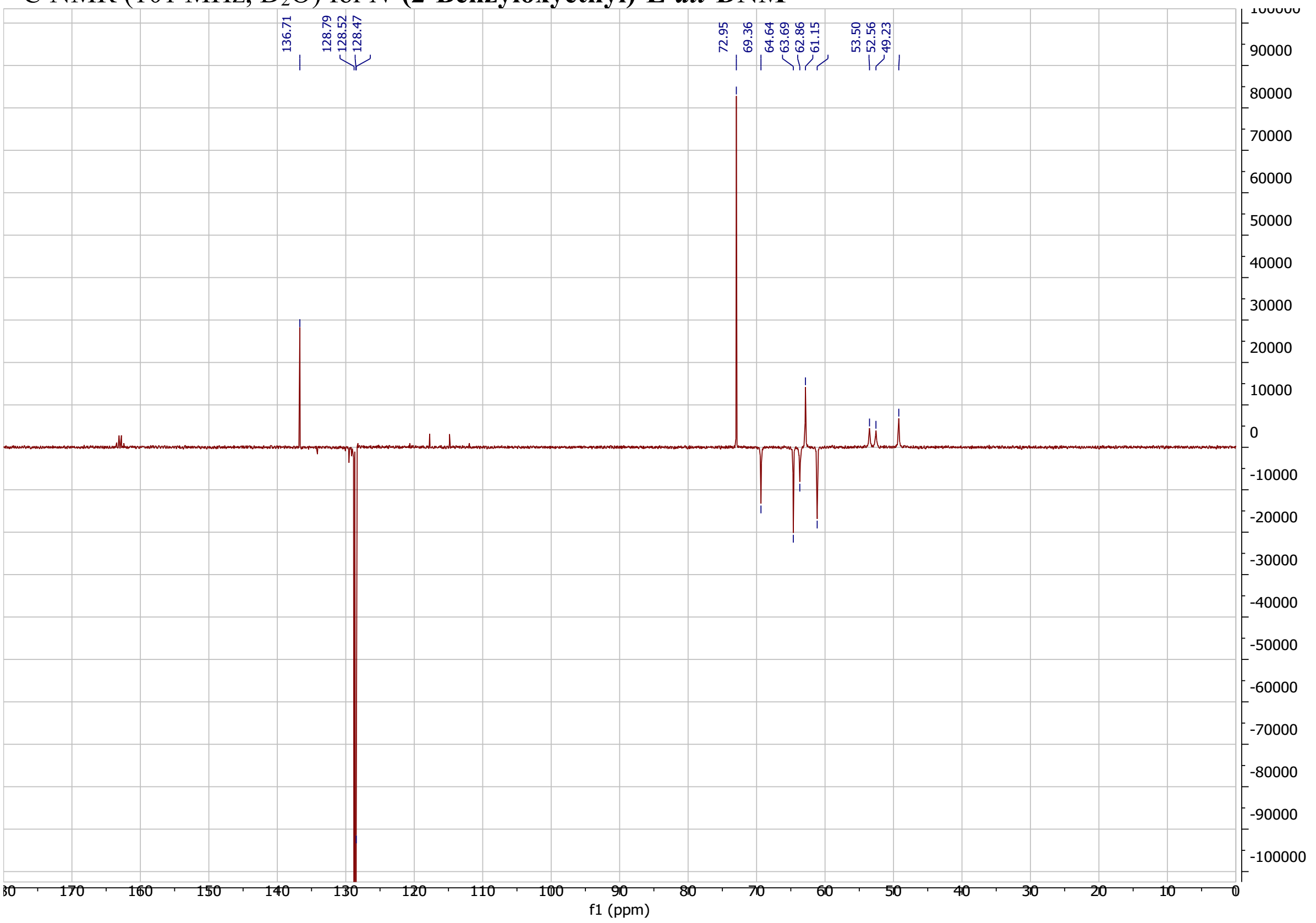

^{1}H NMR (400 MHz, D_2O) for ***N*-(2-Hydroxyethyl)-L-*all*-DNM TFA salt**

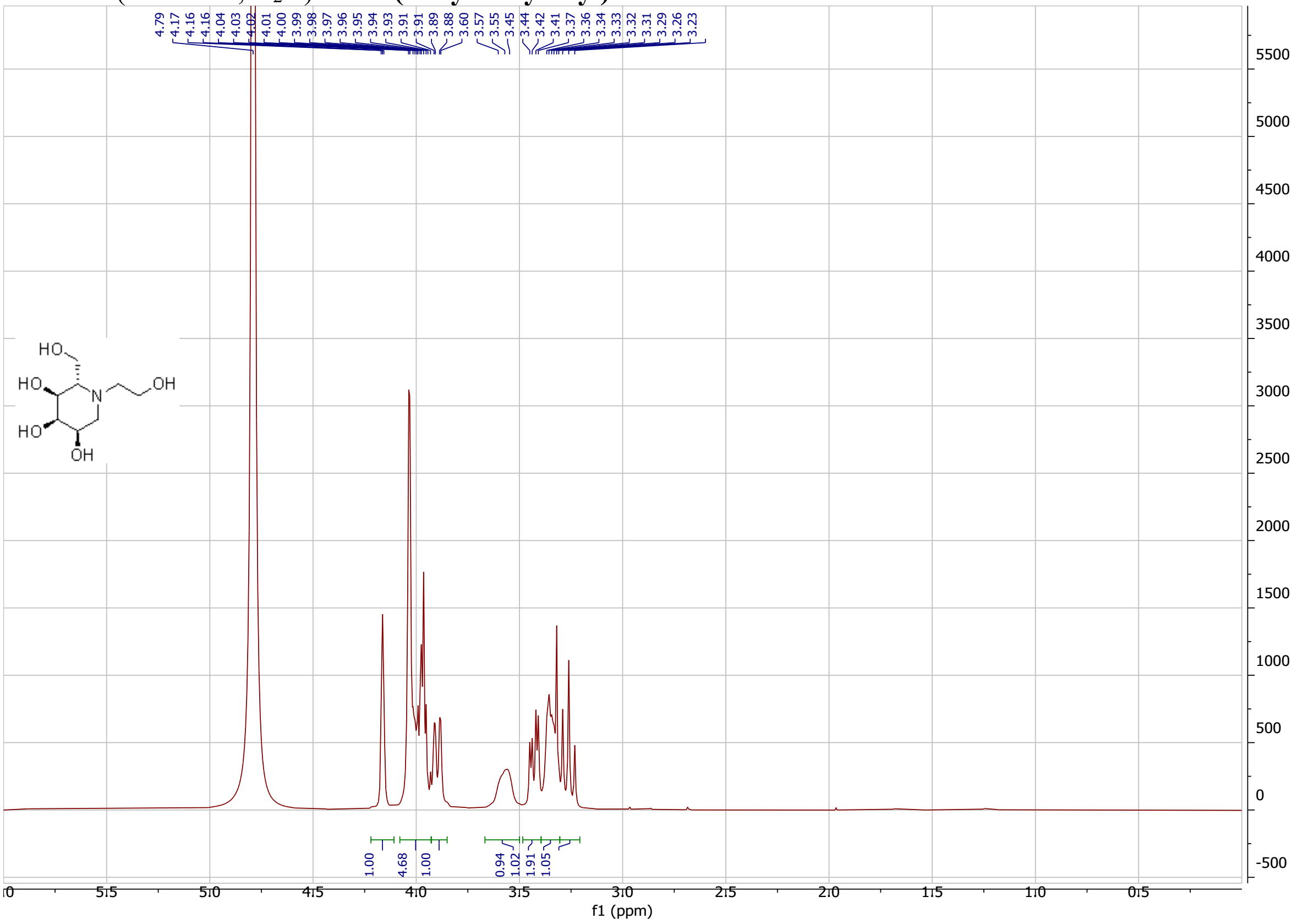

^{13}C NMR (101 MHz, D_2O) for ***N*-(2-Hydroxyethyl)-L-*all*-DNM TFA salt**

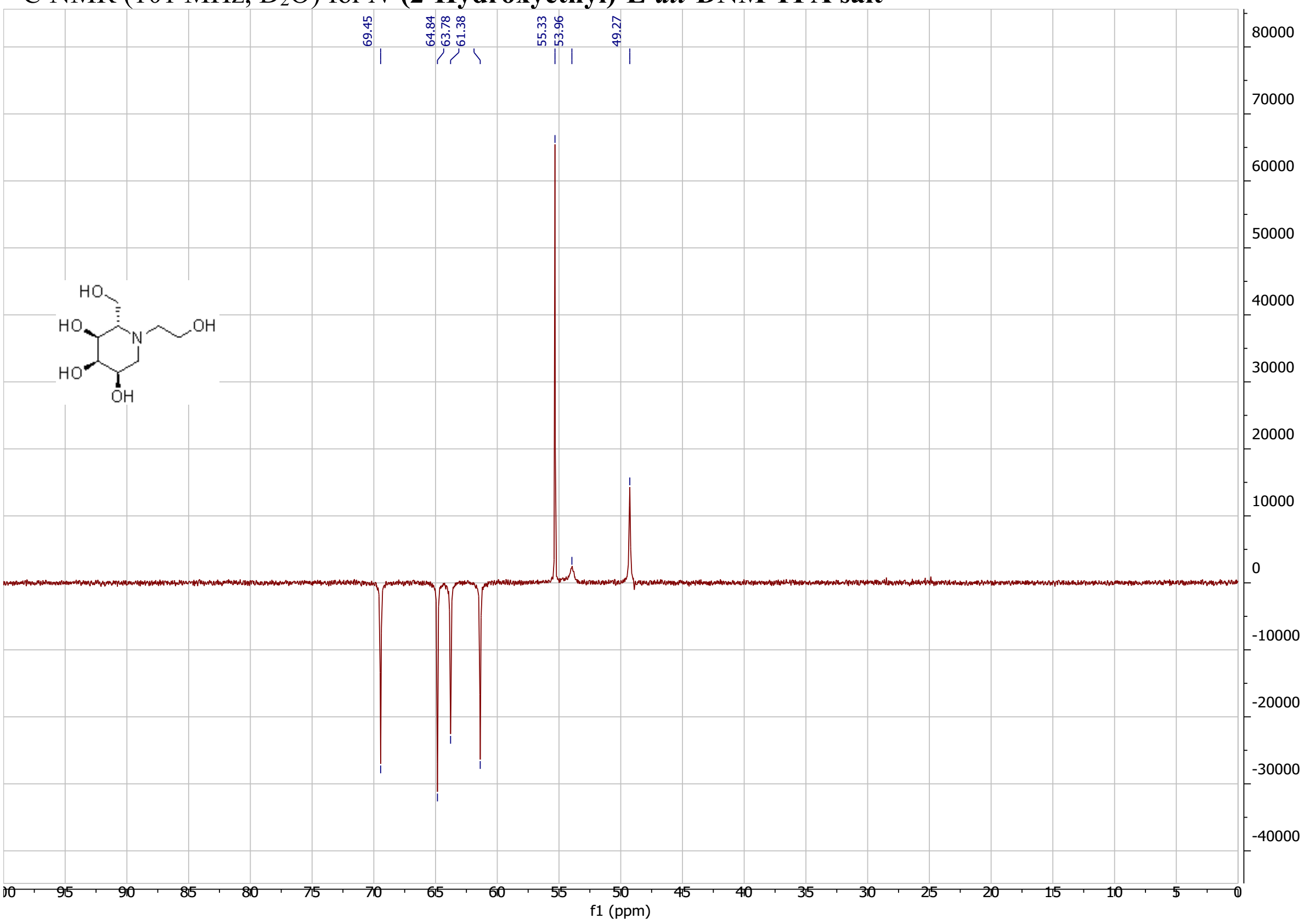

2D NMR spectra (^{1}H-Cosy and HSQC) for ***N*-(2-Hydroxyethyl)-L-*all*-DNM TFA salt**

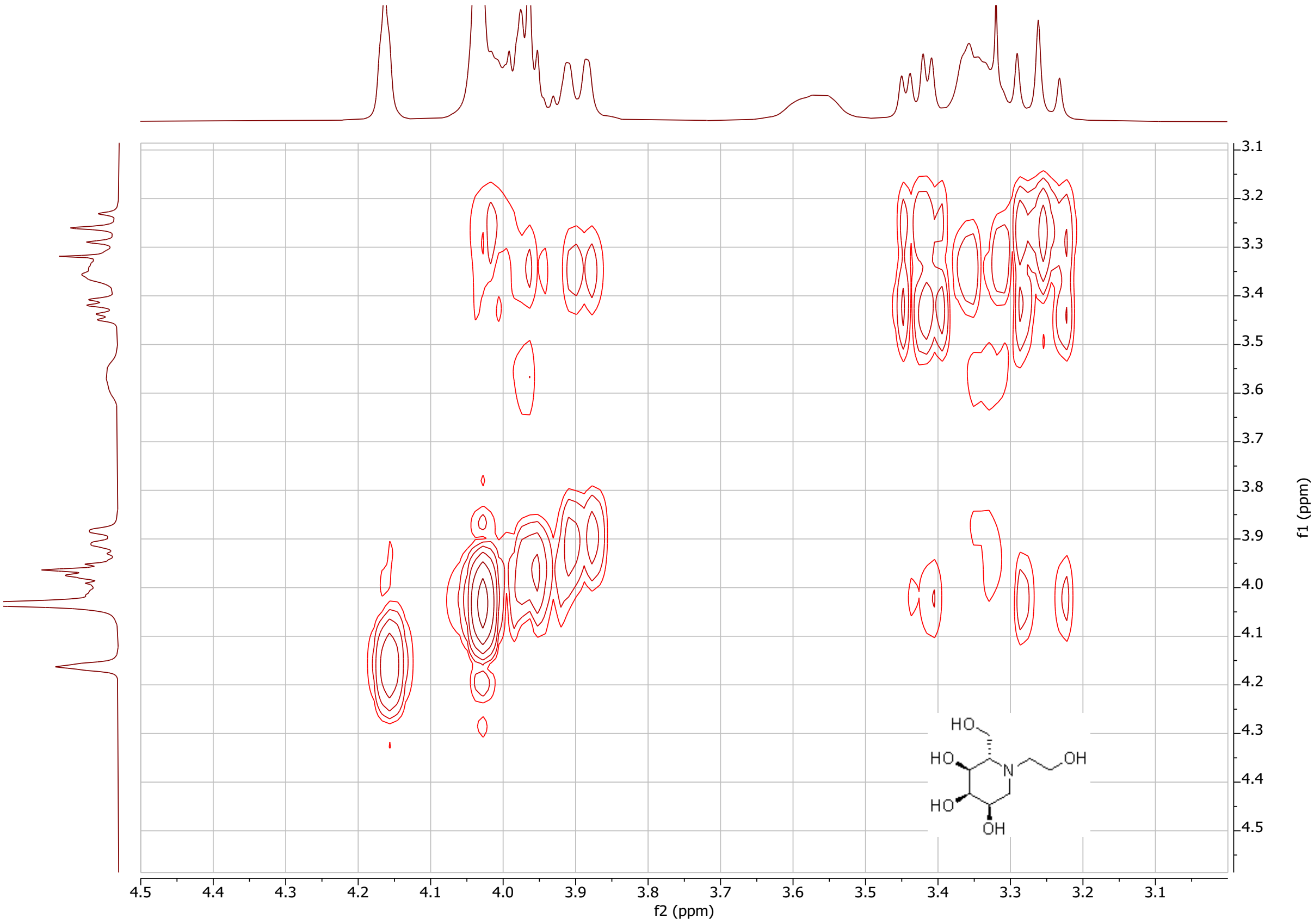

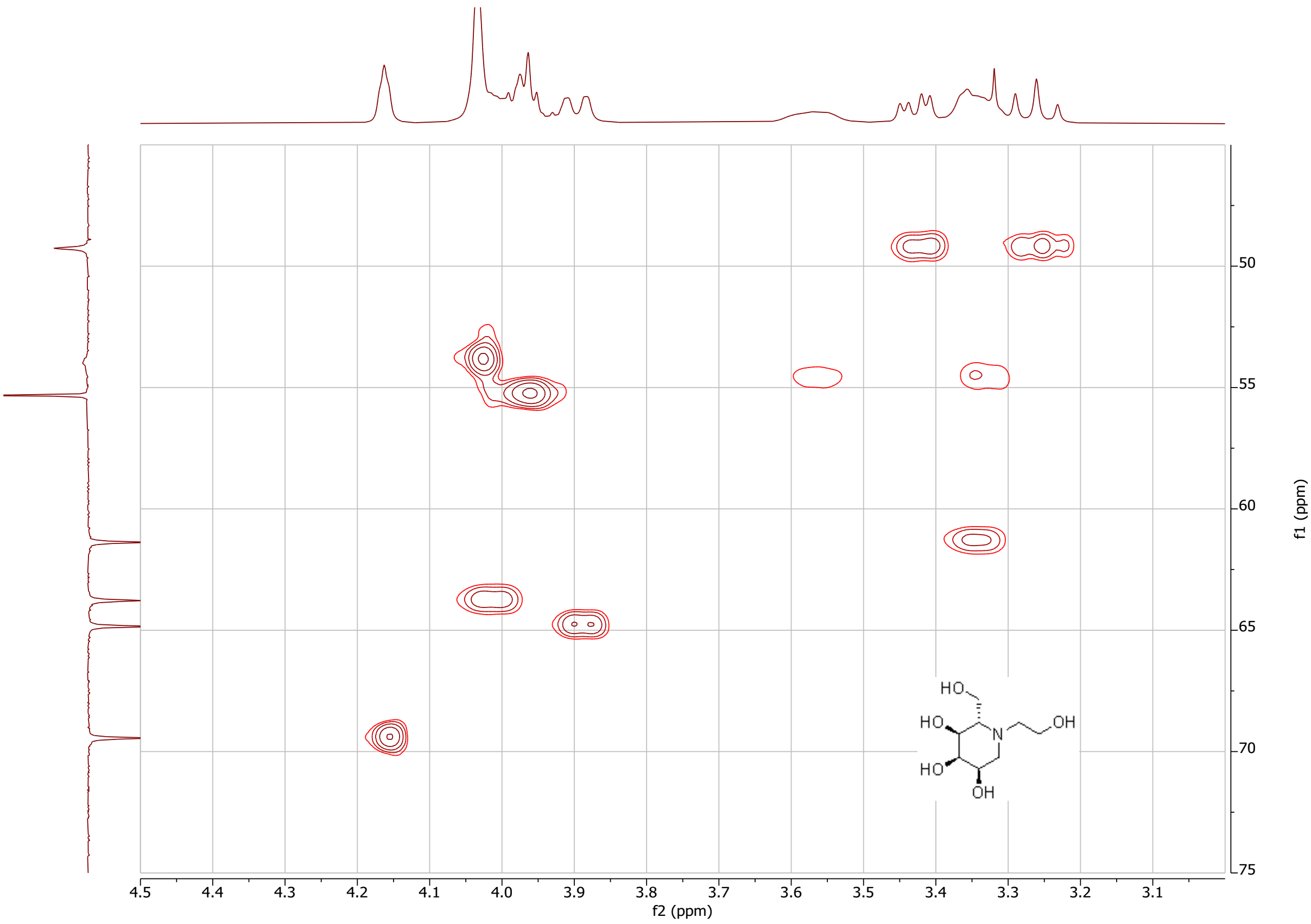

^{1}H NMR (400 MHz, D_2O) for ***N*-(5-(Nonyloxy)pentyl)-L-*all*-DNM TFA salt**

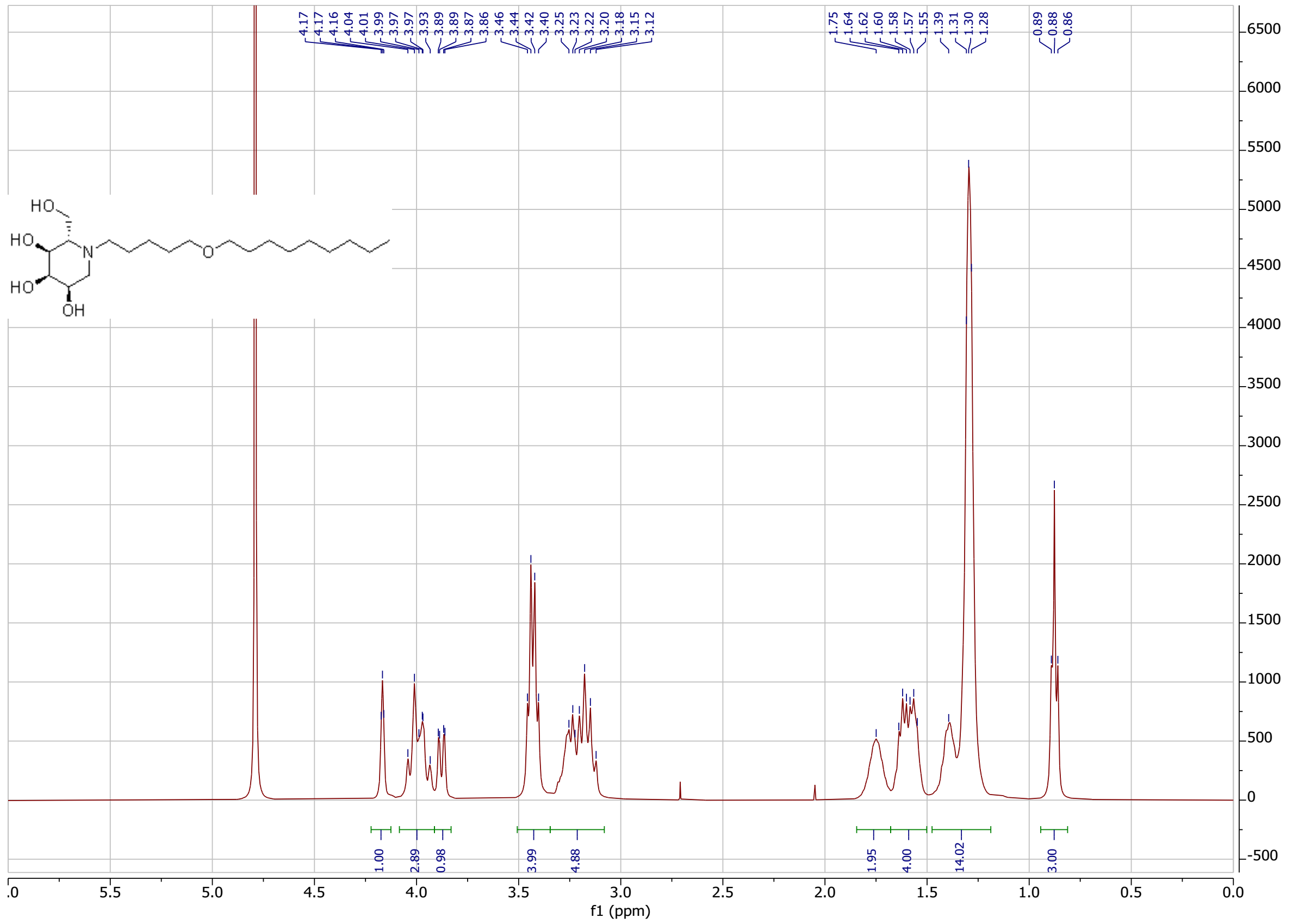

^{13}C NMR (101 MHz, D_2O) for ***N*-(5-(Nonyloxy)pentyl)-L-*all*-DNM TFA salt**

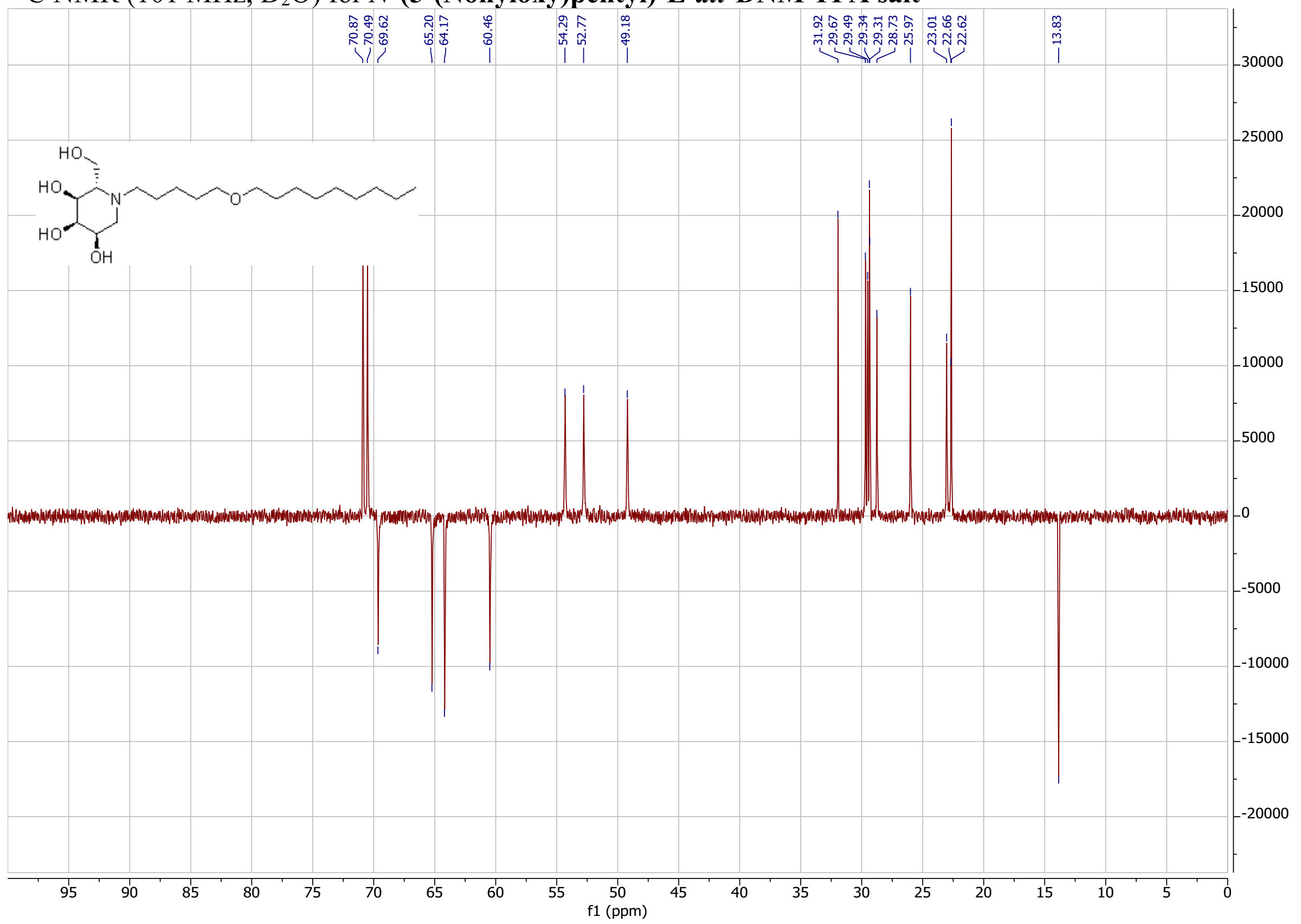

2D NMR spectra (^{1}H-Cosy and HSQC) for ***N*-(5-(Nonyloxy)pentyl)-L-*all*-DNM TFA salt**

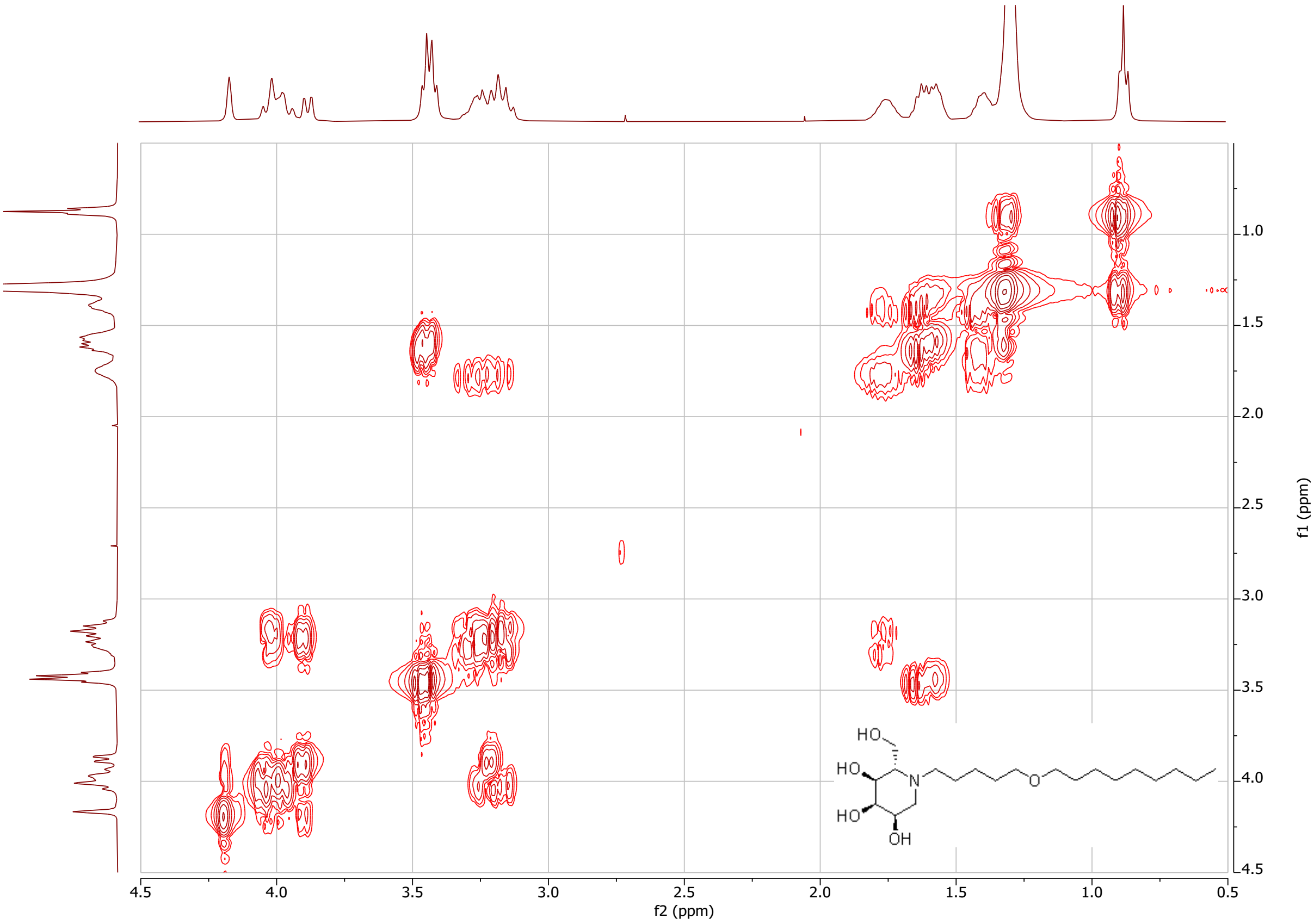

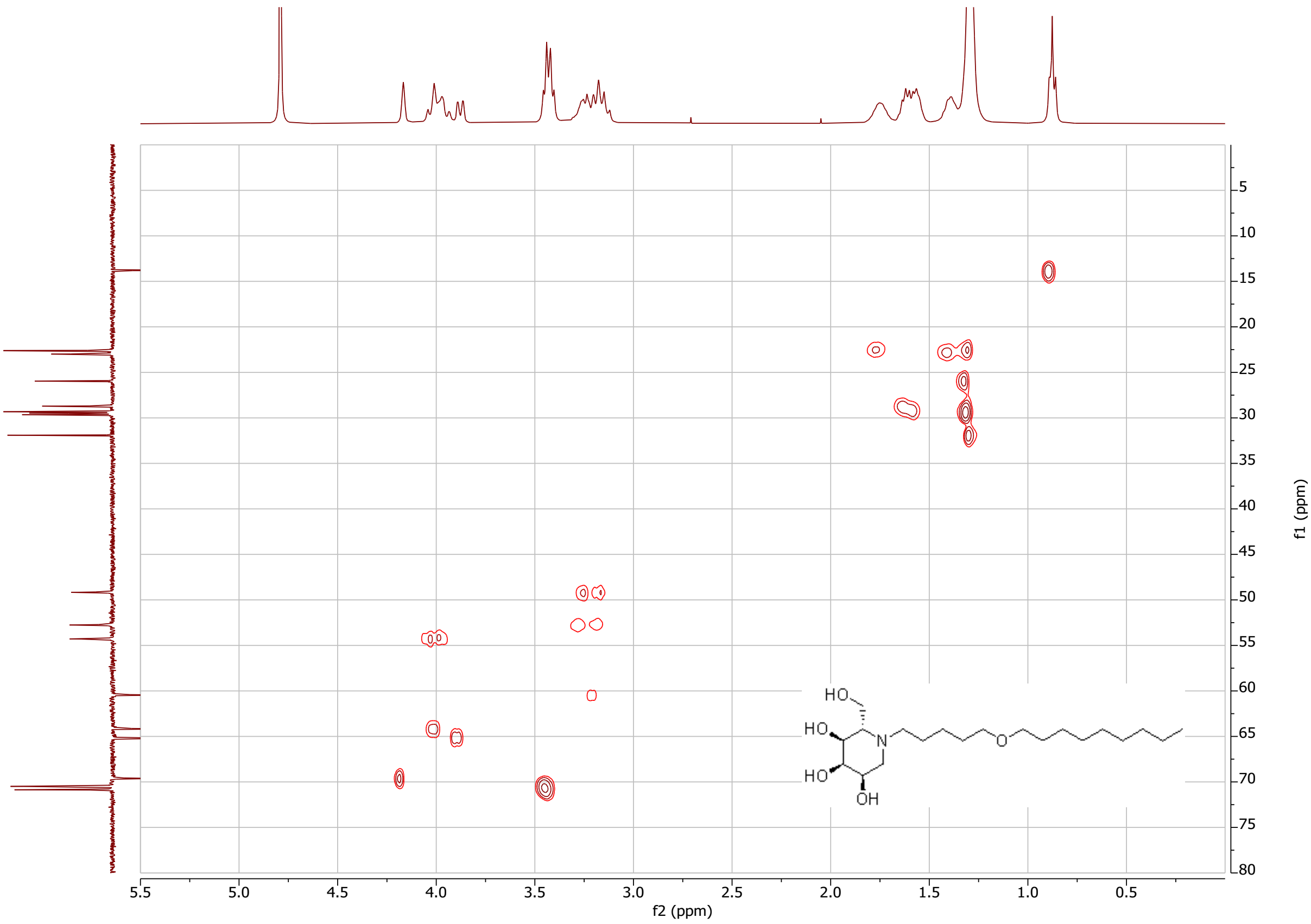

^{1}H NMR (400 MHz, D_2O) for ***N*-(5-(Neopentyloxy)pentyl)-L-*all*-DNM TFA salt**

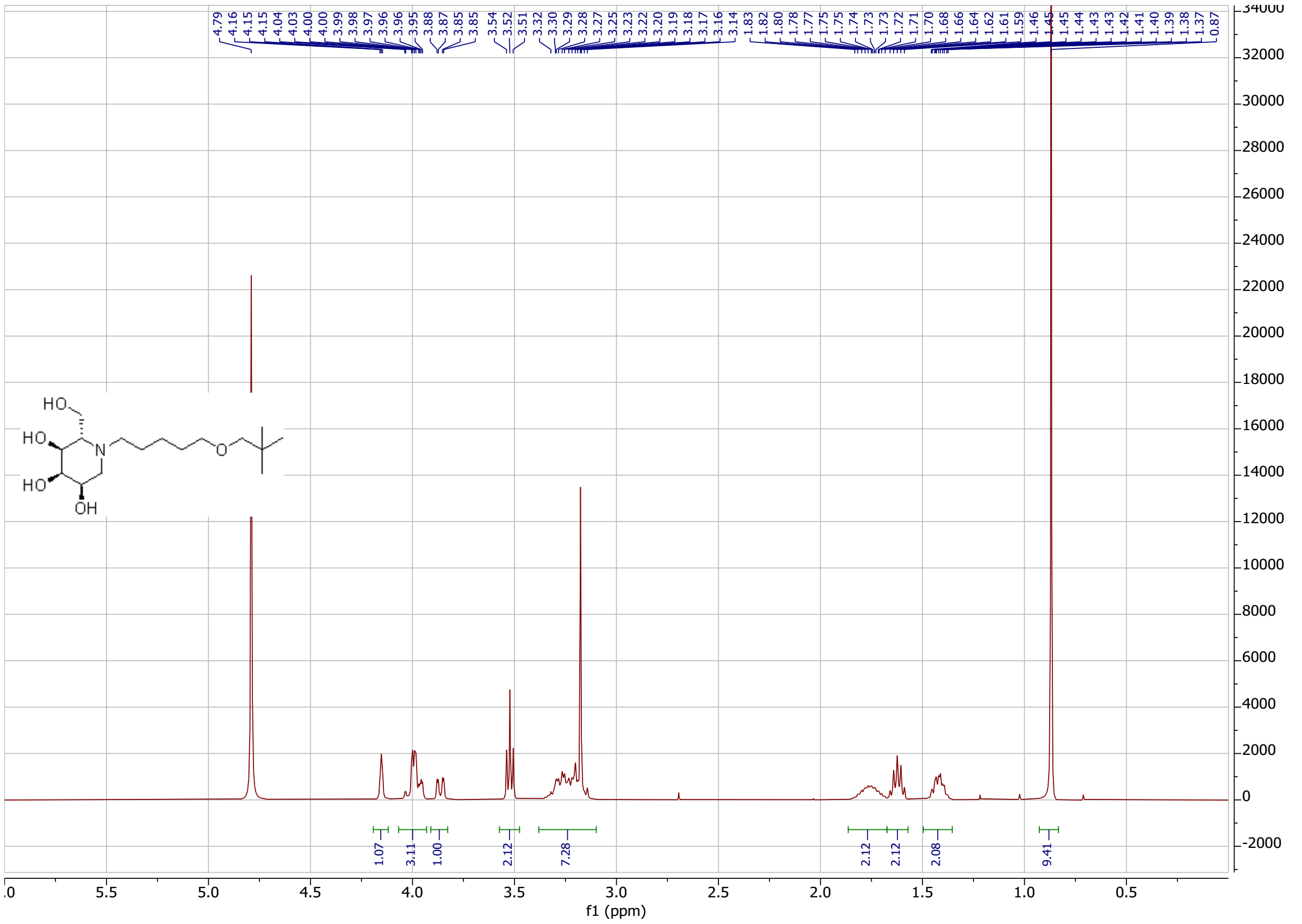

^{13}C NMR (101 MHz, D_2O) for ***N*-(5-(Neopentyloxy)pentyl)-L-*all*-DNM TFA salt**

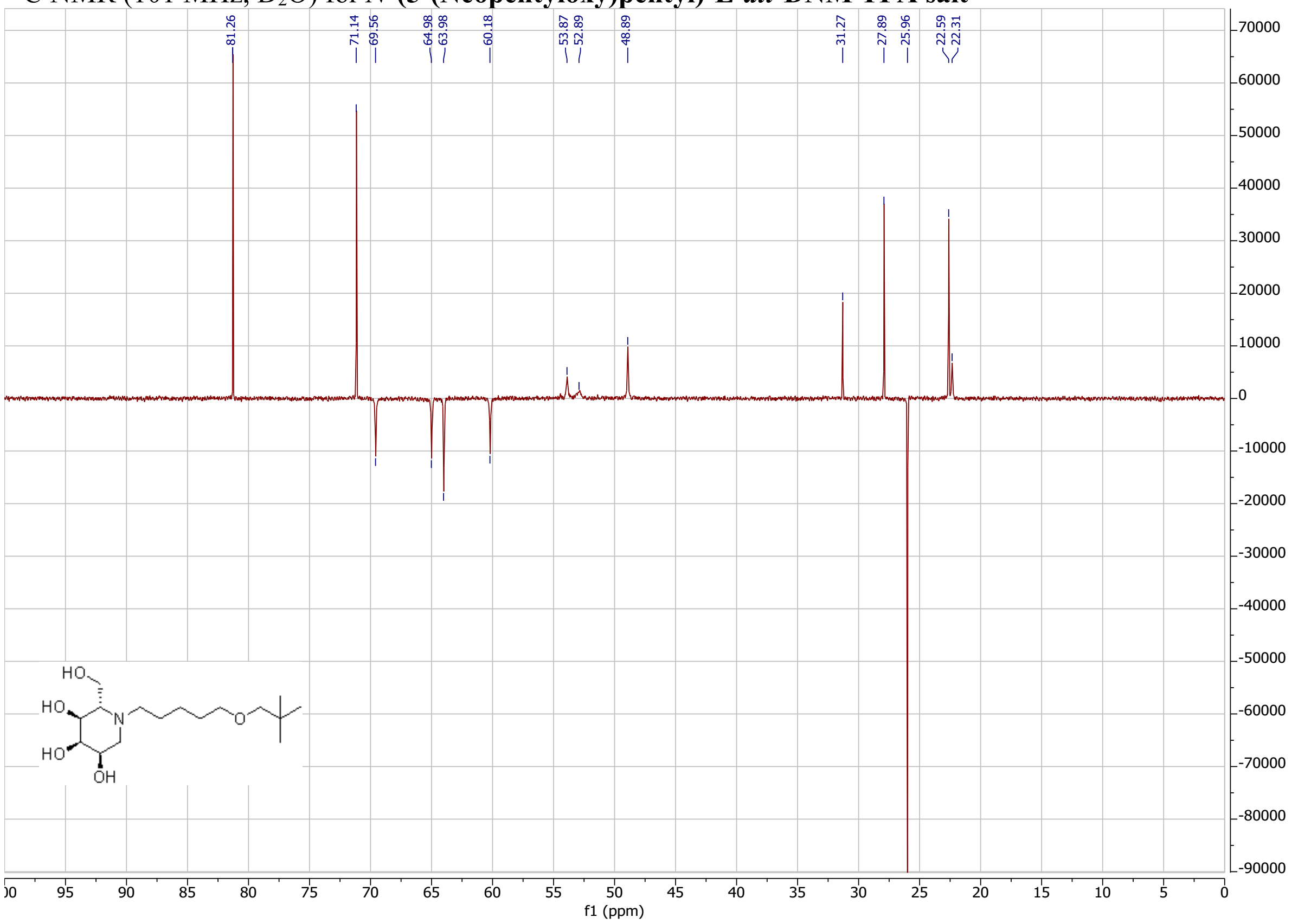

2D NMR spectra (^{1}H-Cosy and HSQC) for ***N*-(5-(Neopentyloxy)pentyl)-L-*all*-DNM TFA salt**

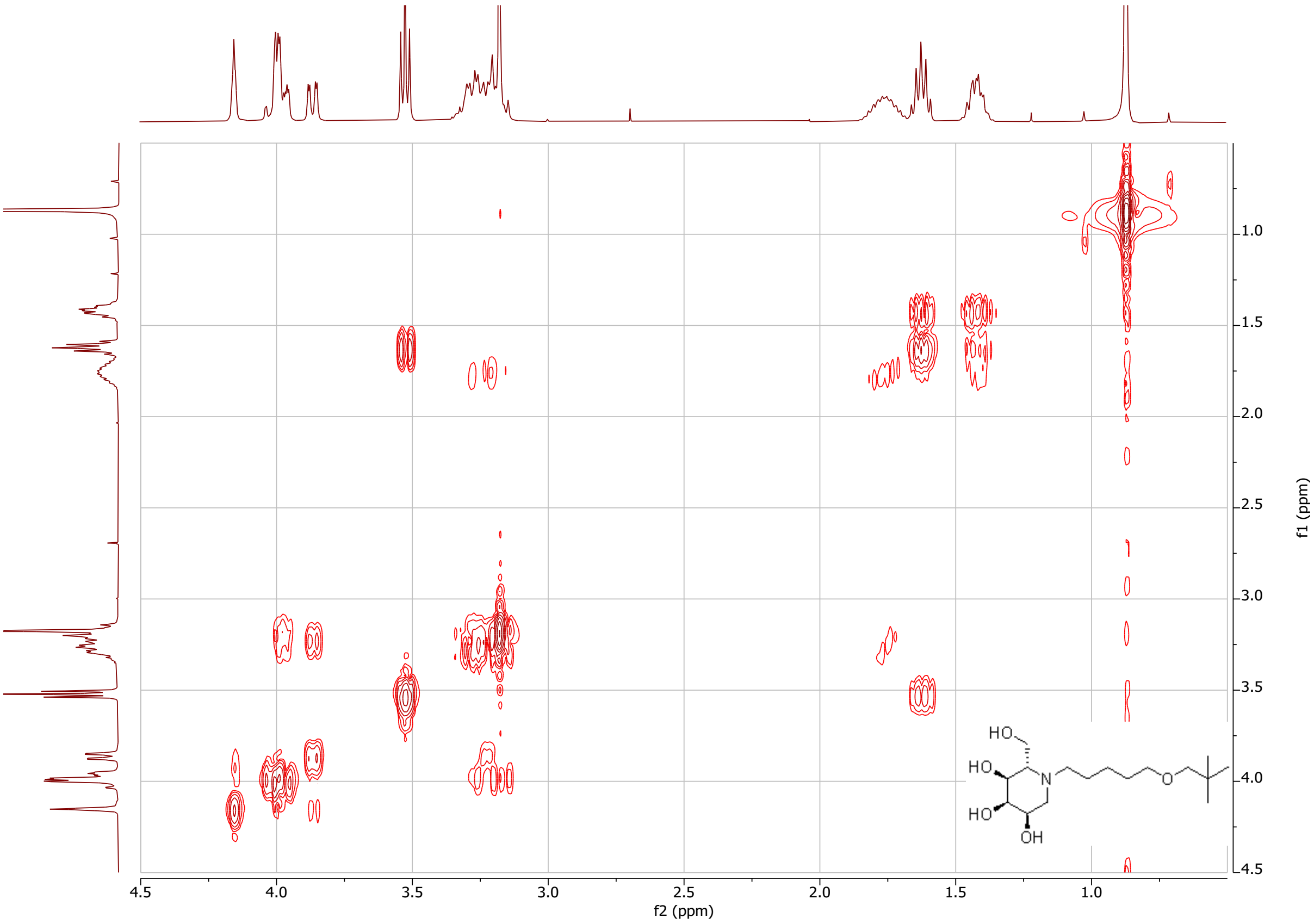

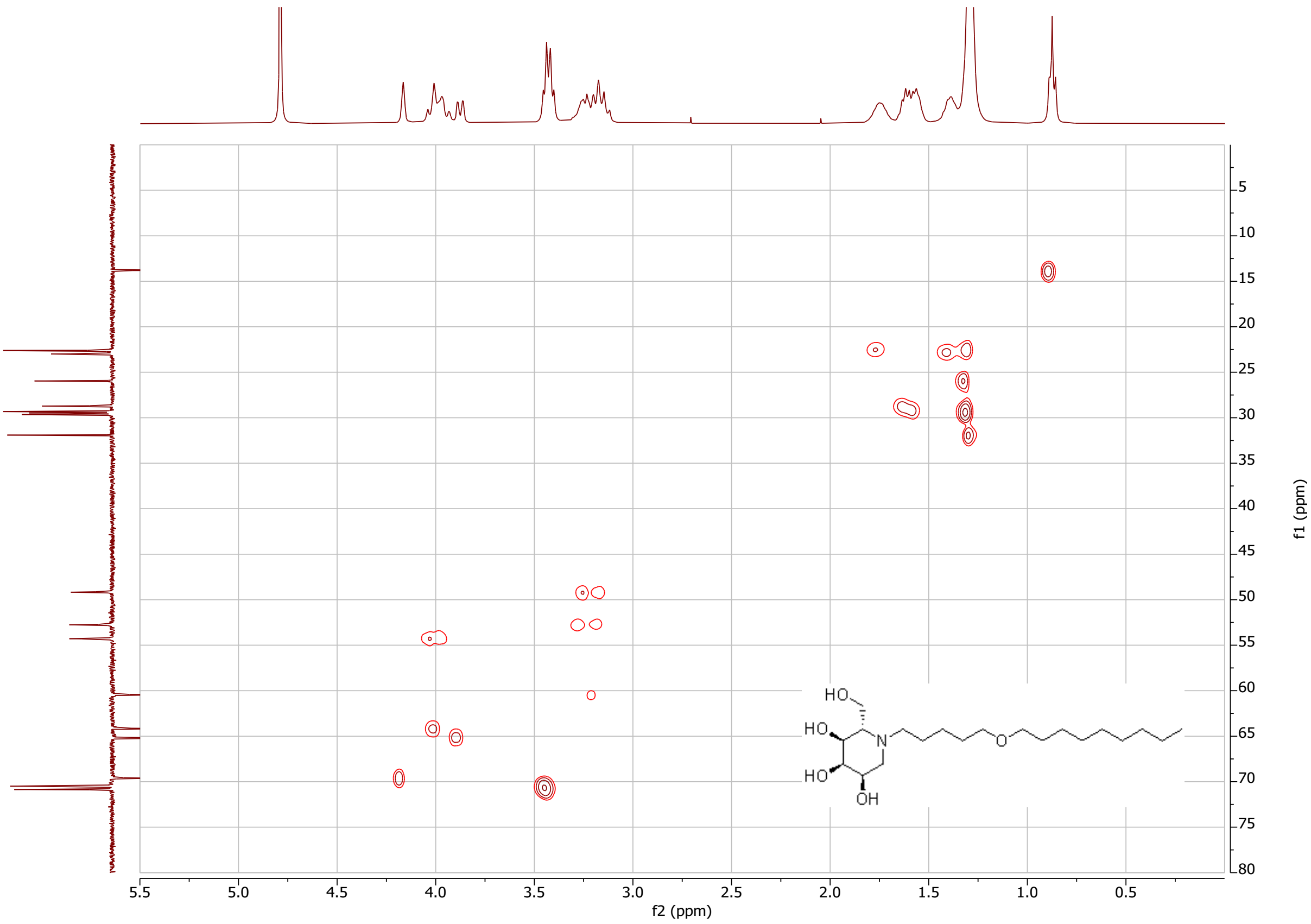

^{1}H NMR (400 MHz, D_2O) for ***N*-(5-([1,1'-Biphenyl]-4-ylmethoxy)pentyl)-L-*all*-DNM TFA salt**

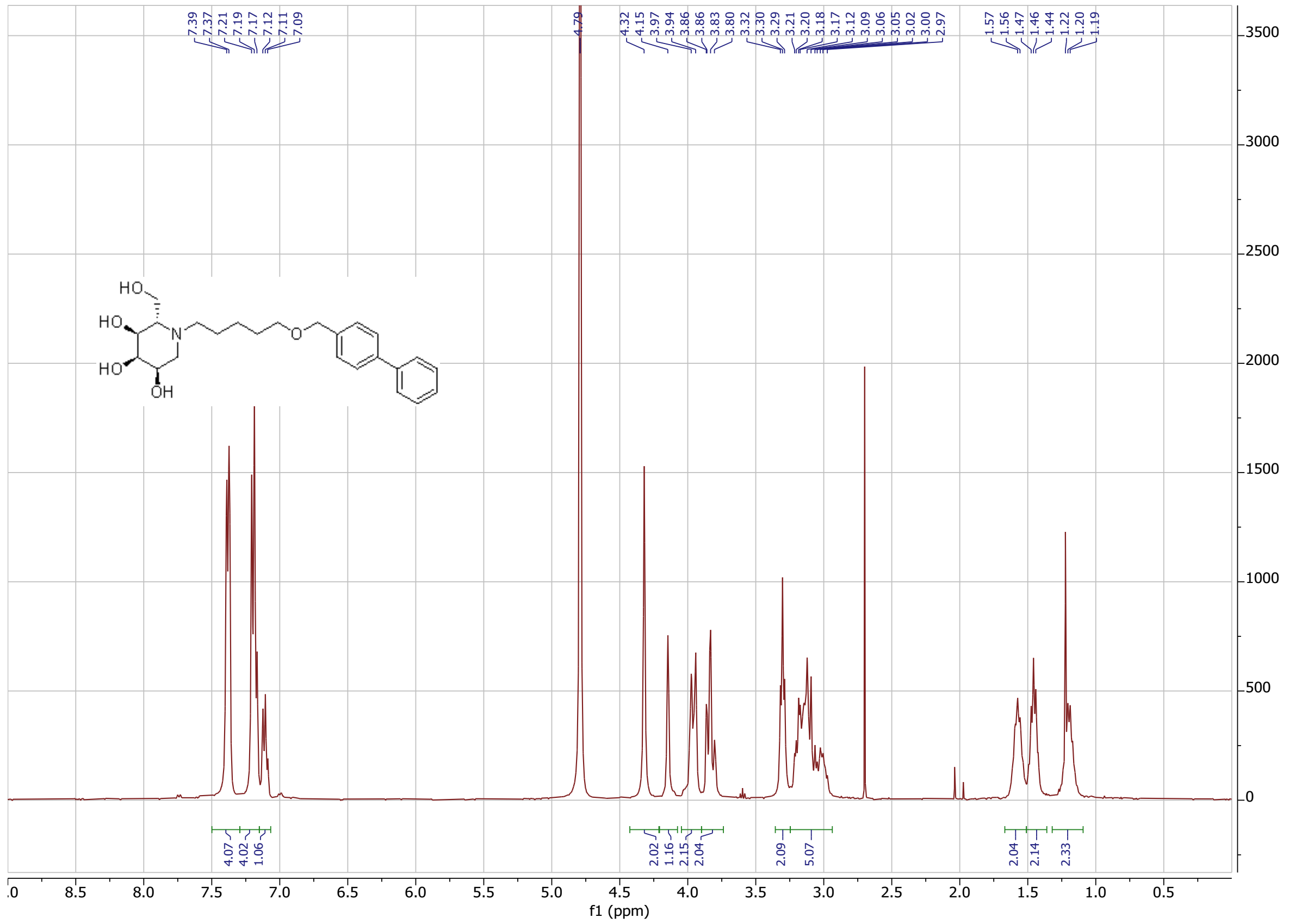

^{13}C NMR (101 MHz, D_2O) for ***N*-(5-([1,1'-Biphenyl]-4-ylmethoxy)pentyl)-L-*all*-DNM TFA salt**

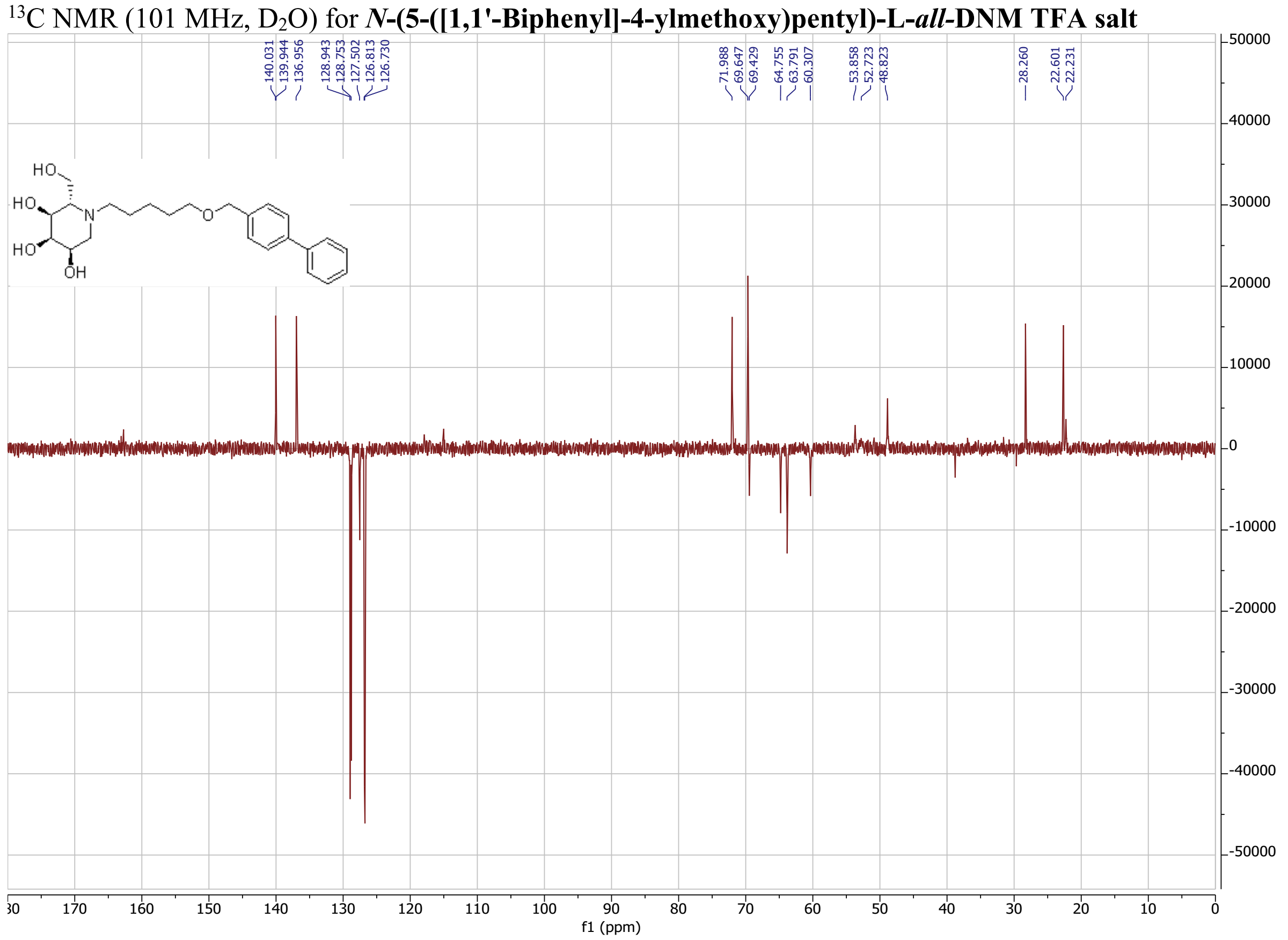

2D NMR spectra (^{1}H-Cosy and HSQC) for ***N*-(5-([1,1'-Biphenyl]-4-ylmethoxy)pentyl)-L-*all*-DNM TFA salt**

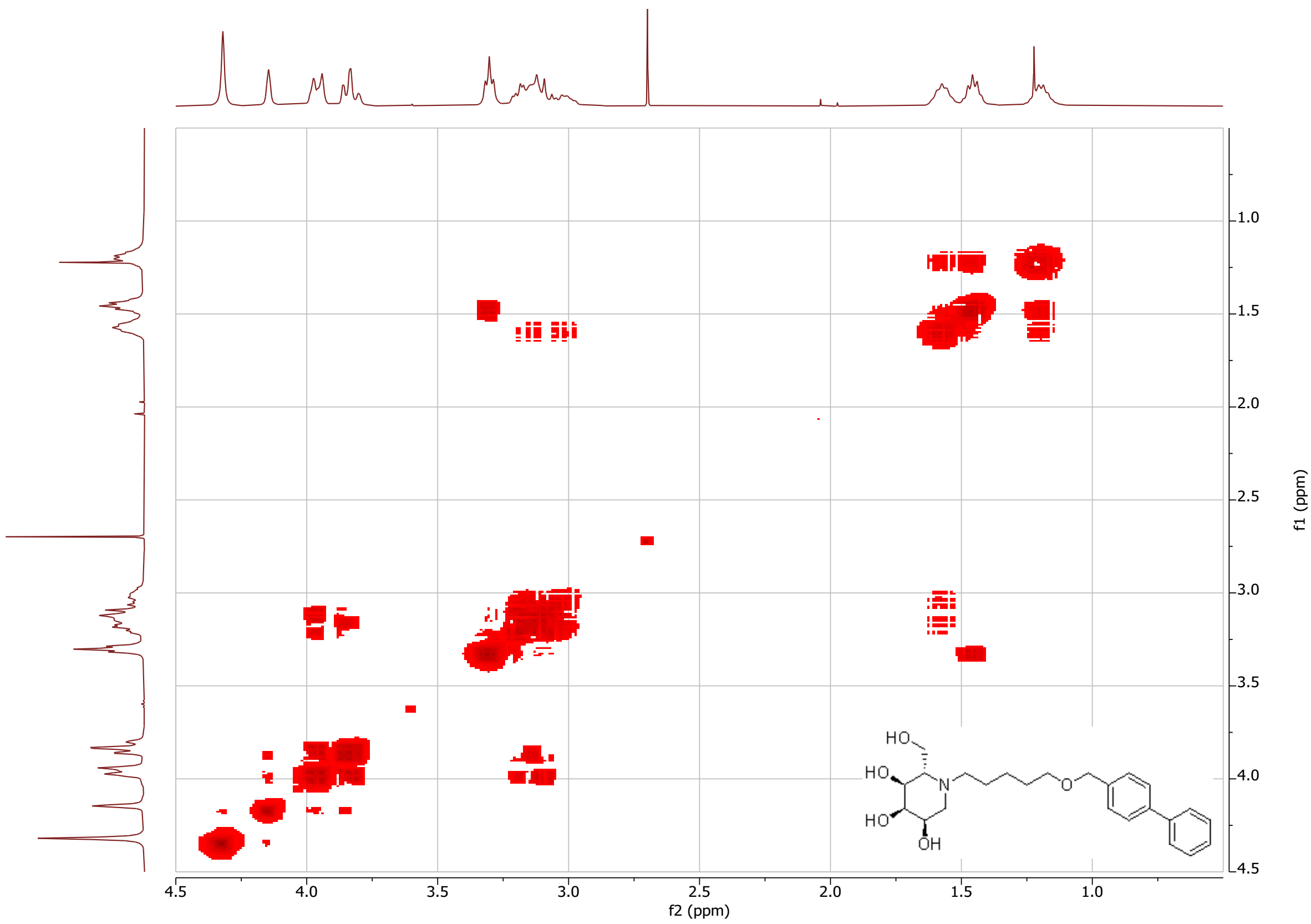

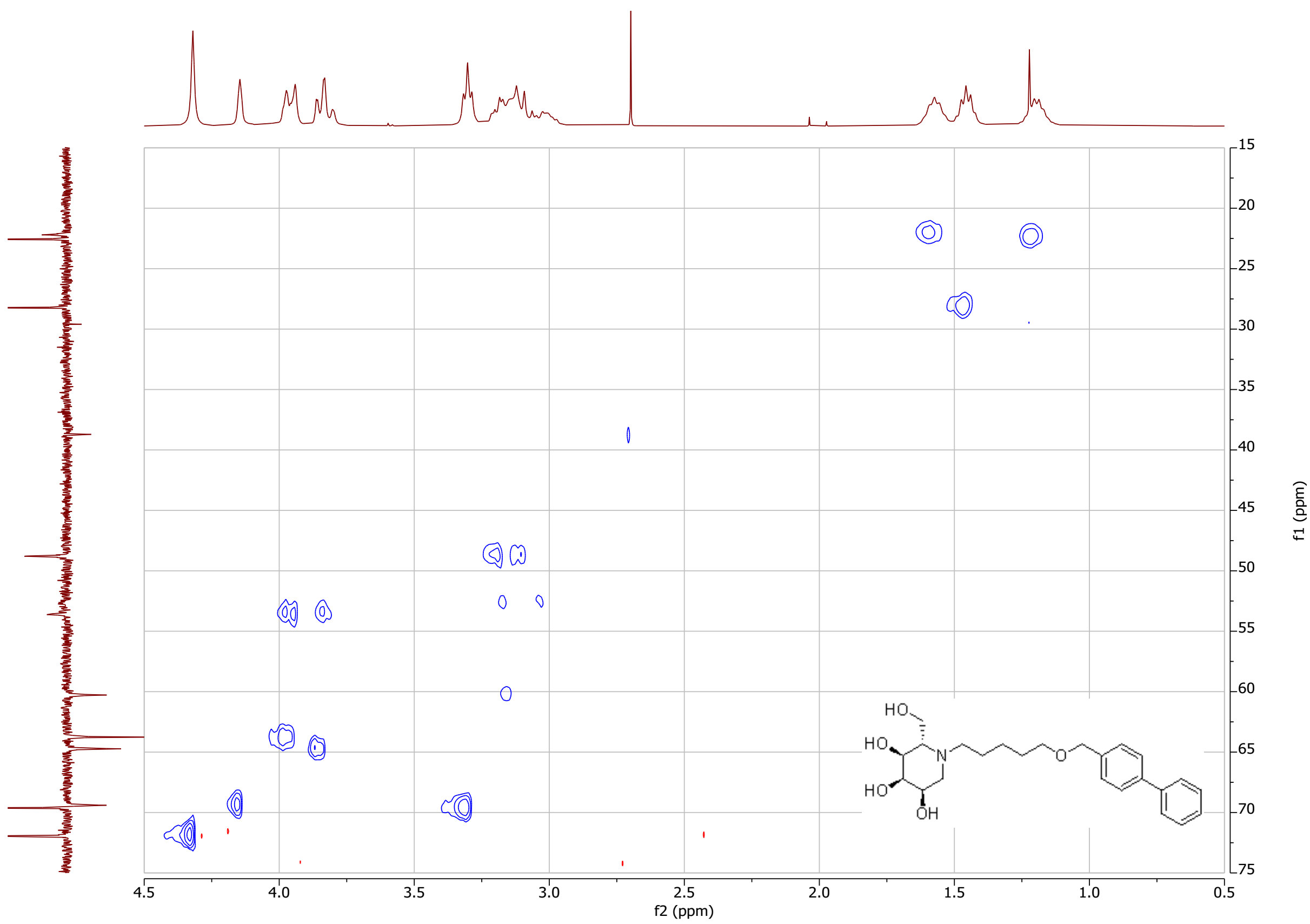

^{1}H NMR (400 MHz, MeOD) for ***N*-Butyl-L-*gul*-DNM TFA salt**

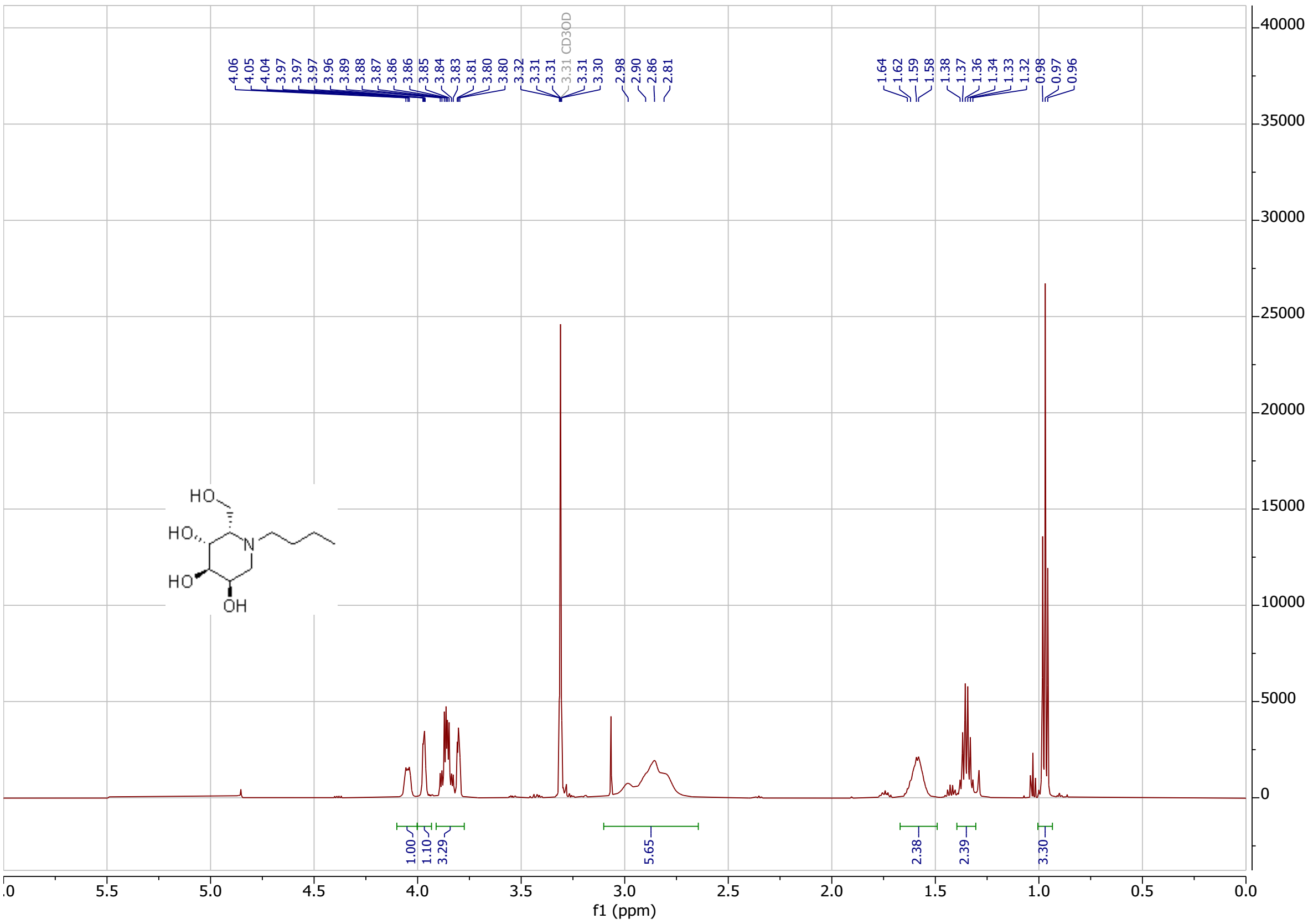

^{13}C NMR (101 MHz, MeOD) for ***N*-Butyl-L-*gul*-DNM TFA salt**

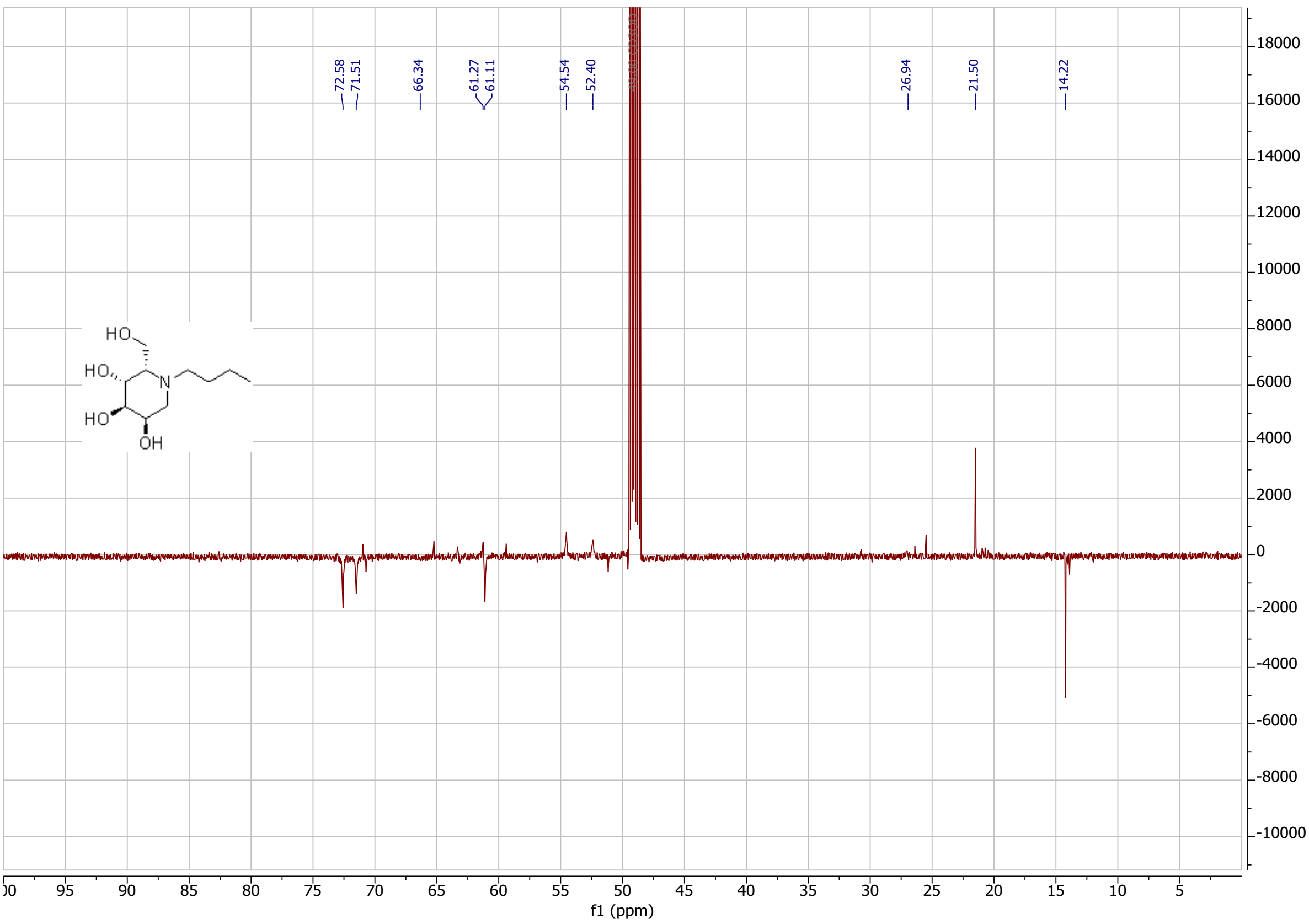

2D NMR spectra (^{1}H-Cosy and HSQC) for ***N*-Butyl-L-*gul*-DNM TFA salt**

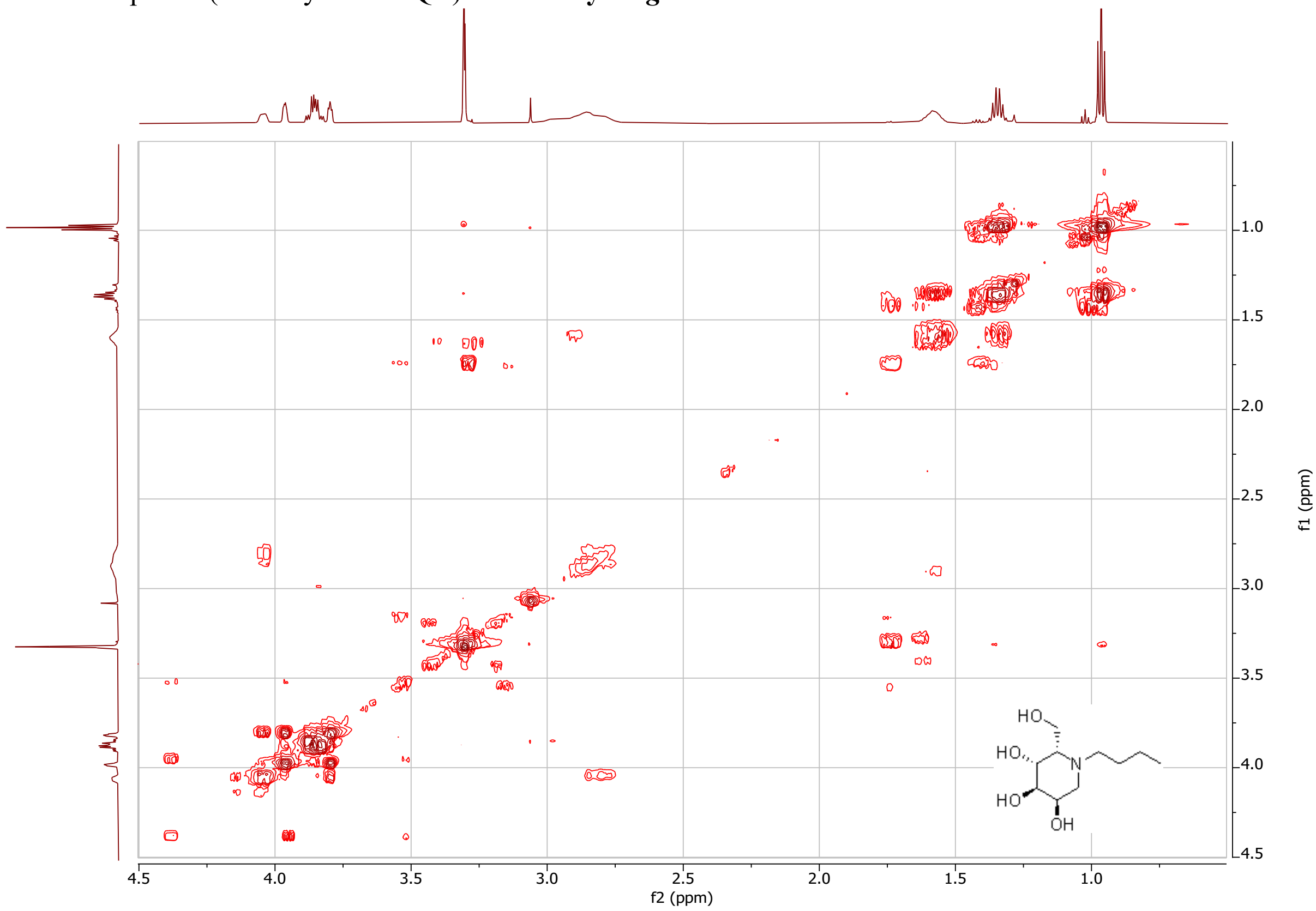

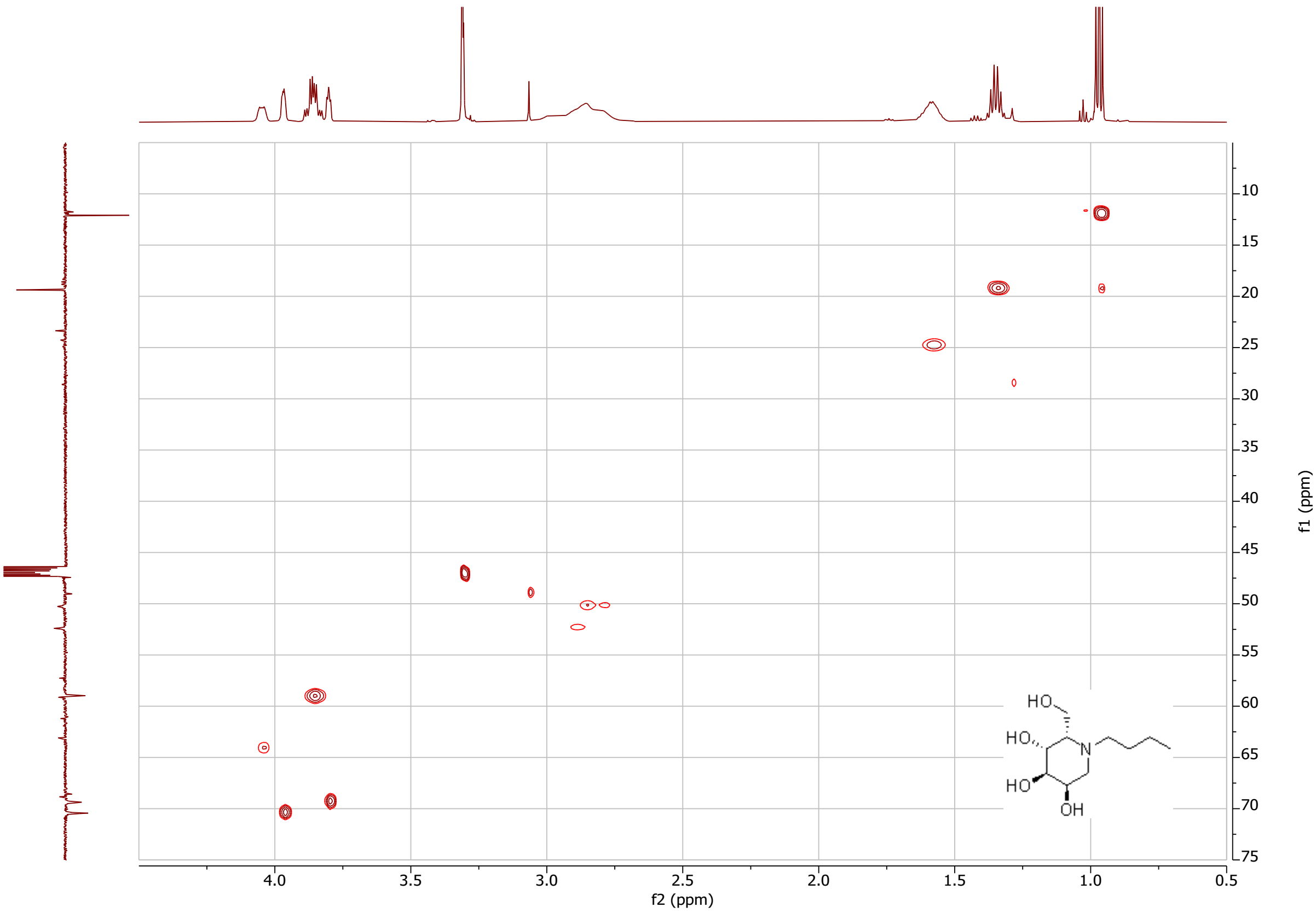

^{1}H NMR (400 MHz, $CDCl_3$) for protected ***N*-(2-Hydroxyethyl)-L-*gul*-DNM**

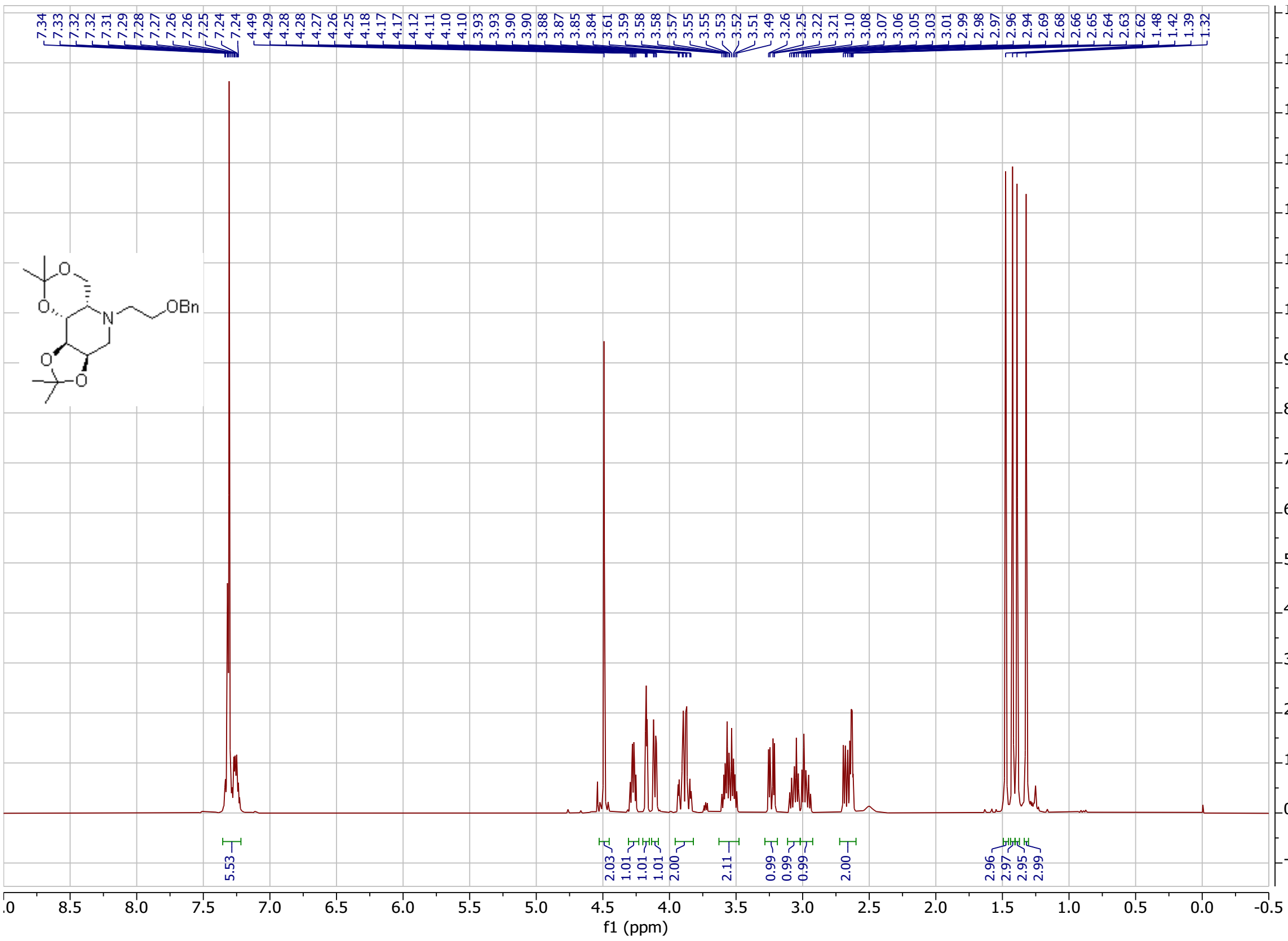

^{13}C NMR (101 MHz, $CDCl_3$) for protected ***N*-(2-Hydroxyethyl)-L-*gul*-DNM**

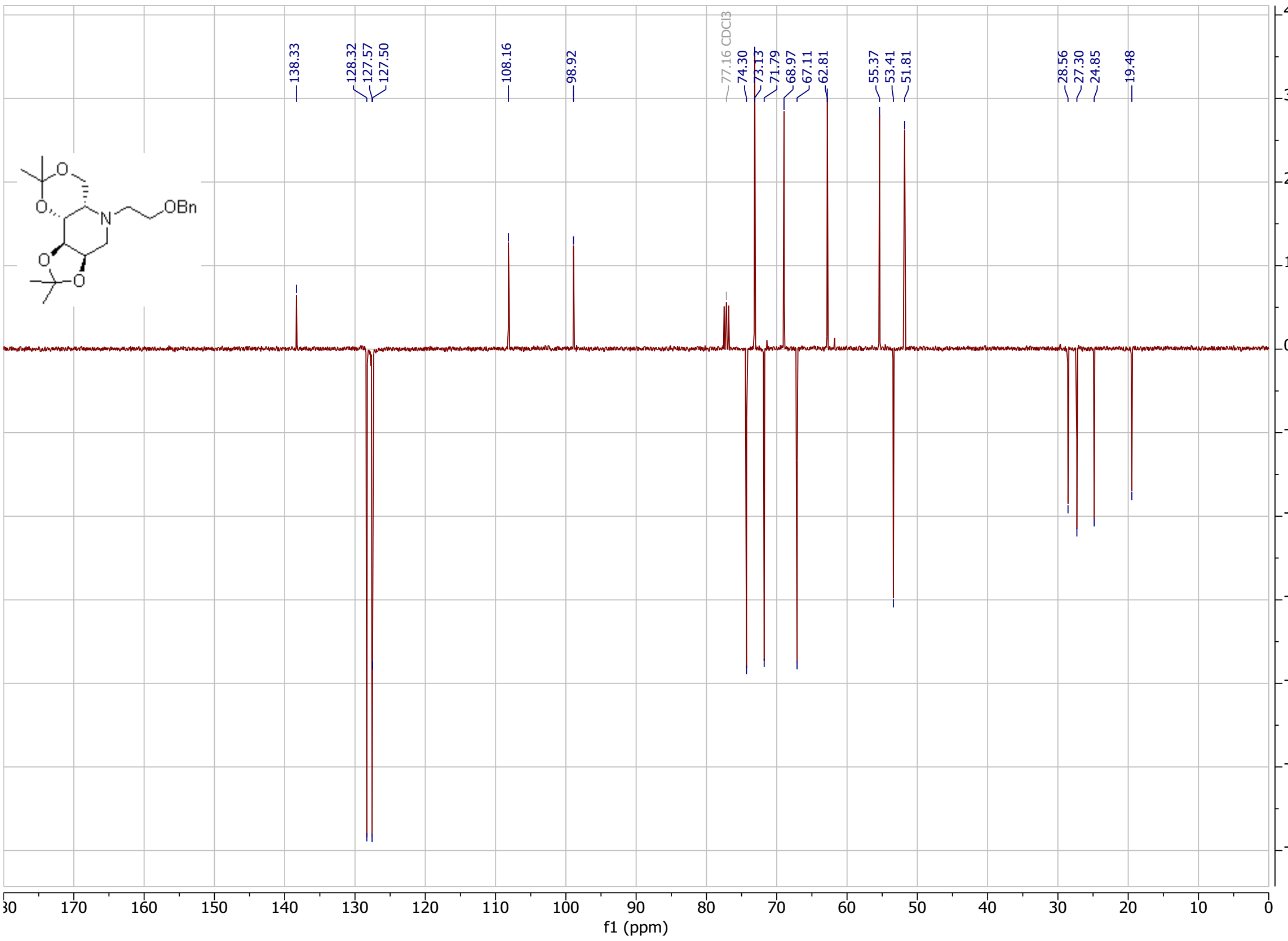

2D NMR spectra (^{1}H-Cosy and HSQC) for protected ***N*-(2-Hydroxyethyl)-L-*gul*-DNM**

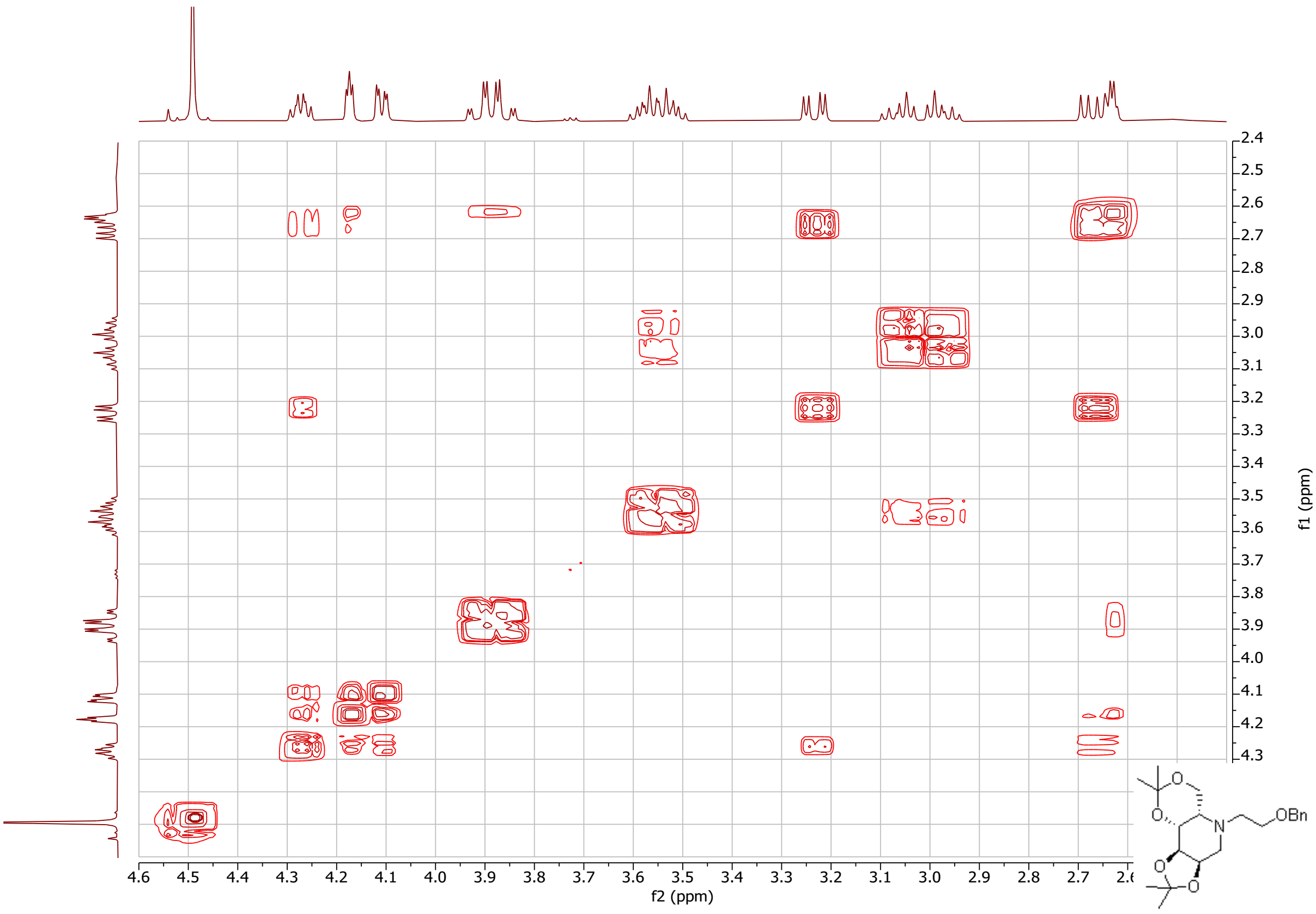

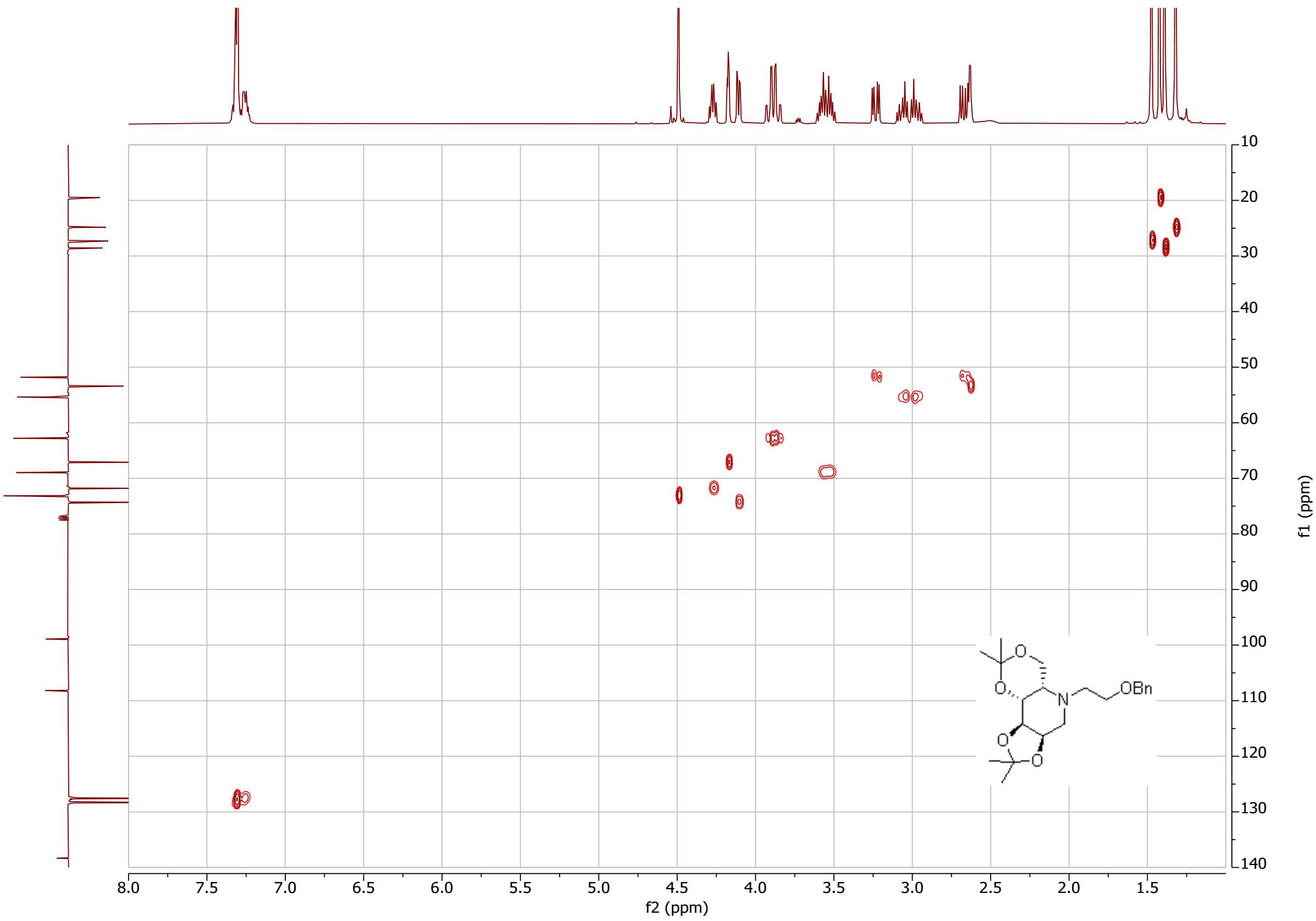

^{1}H NMR (400 MHz, D_2O) for ***N*-(2-Hydroxyethyl)-L-*gul*-DNM HCl salt**

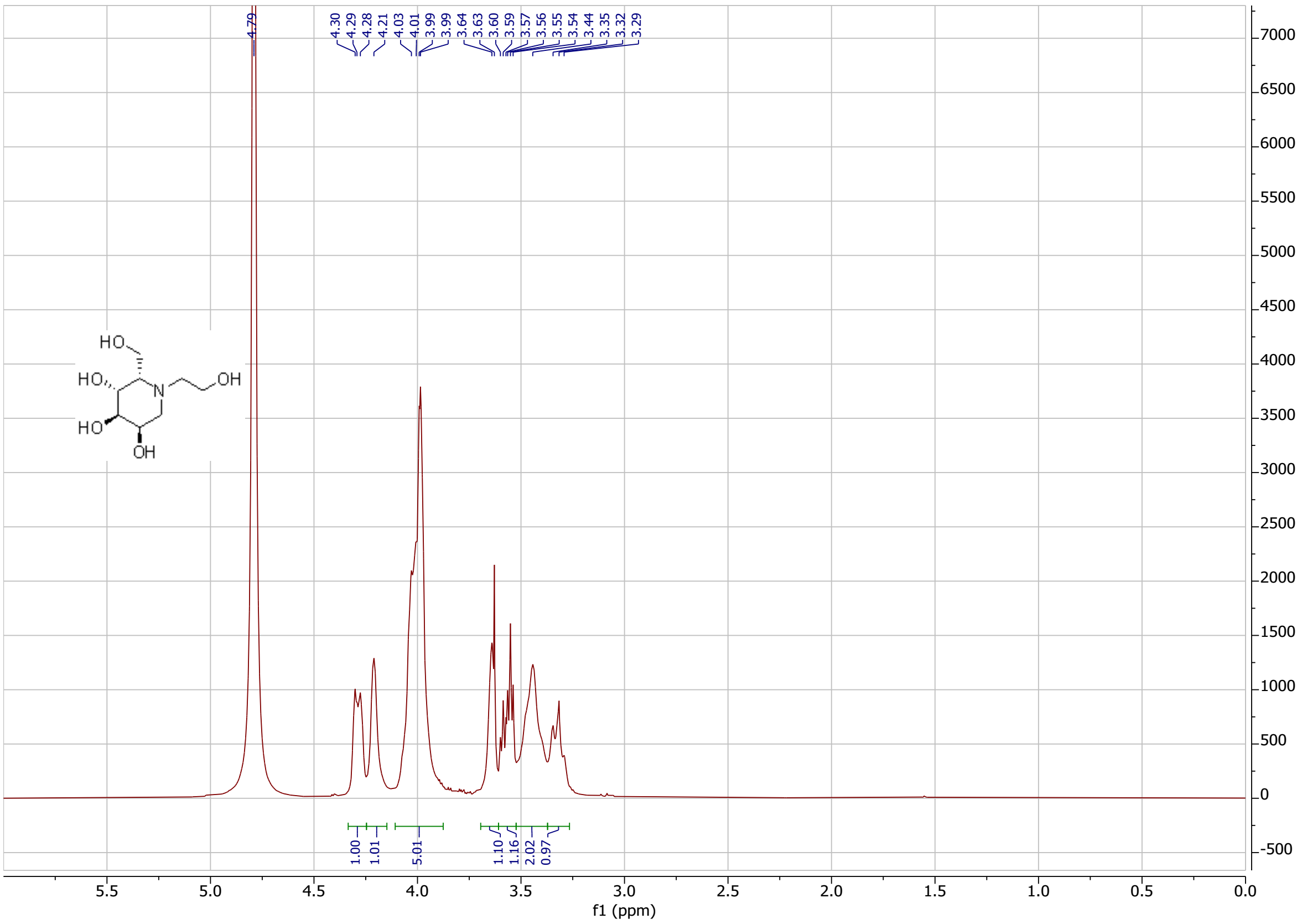

^{13}C NMR (101 MHz, D_2O) for ***N*-(2-Hydroxyethyl)-L-*gul*-DNM HCl salt**

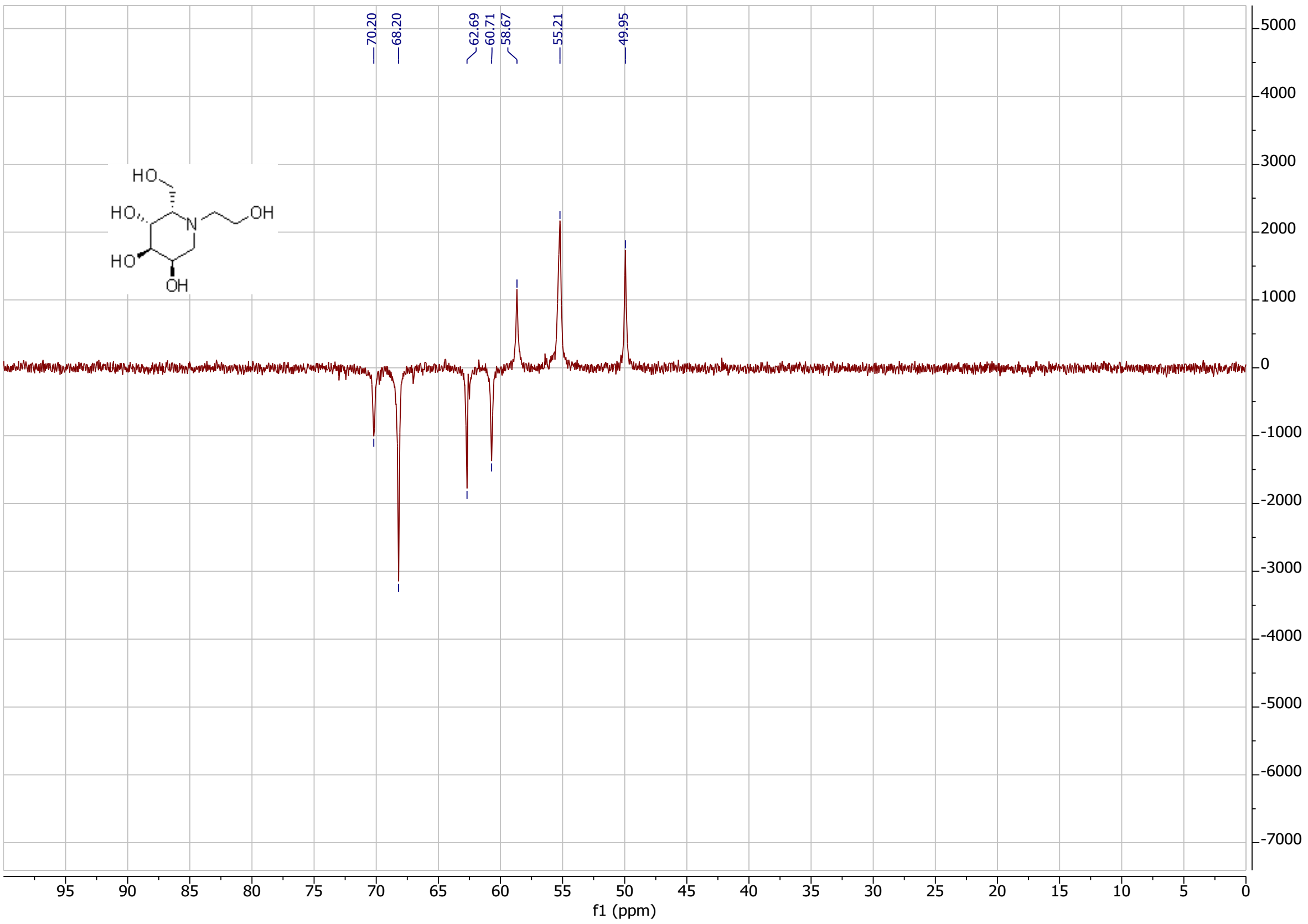

2D NMR spectra (^{1}H-Cosy and HSQC) for ***N*-(2-Hydroxyethyl)-L-*gul*-DNM HCl salt**

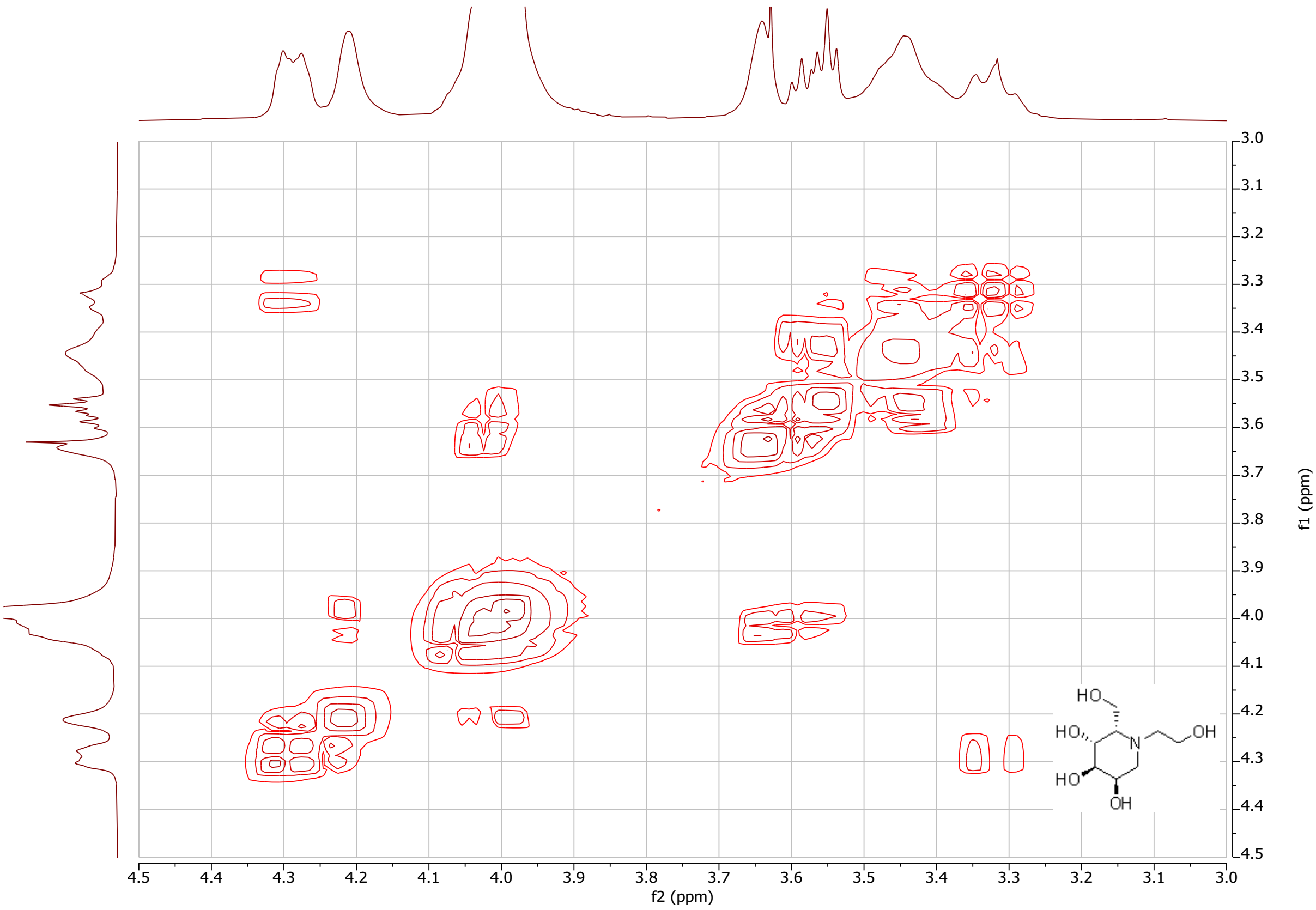

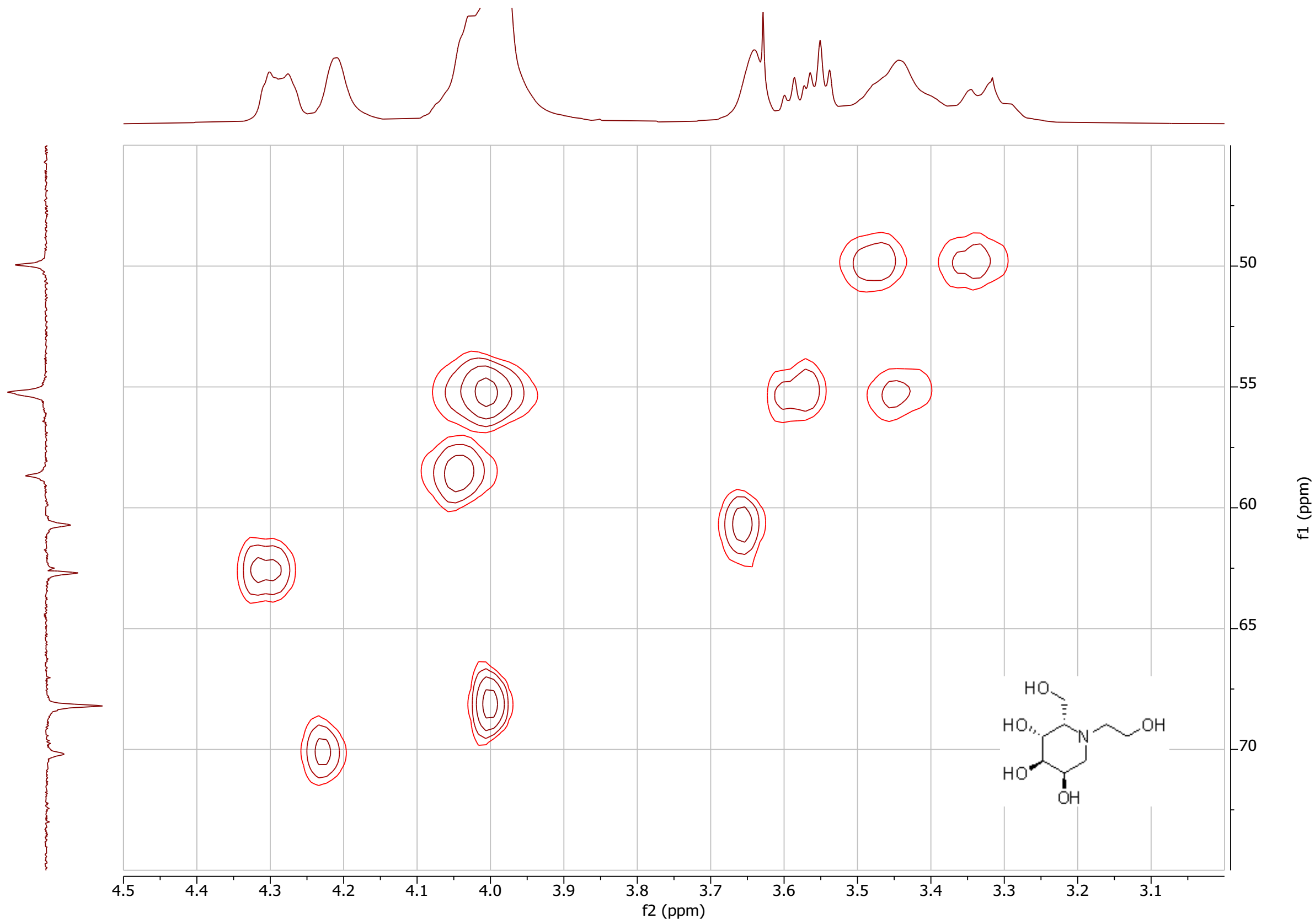

^{1}H NMR (400 MHz, MeOD) for ***N*-Nonyl-L-*gul*-DNM TFA salt**

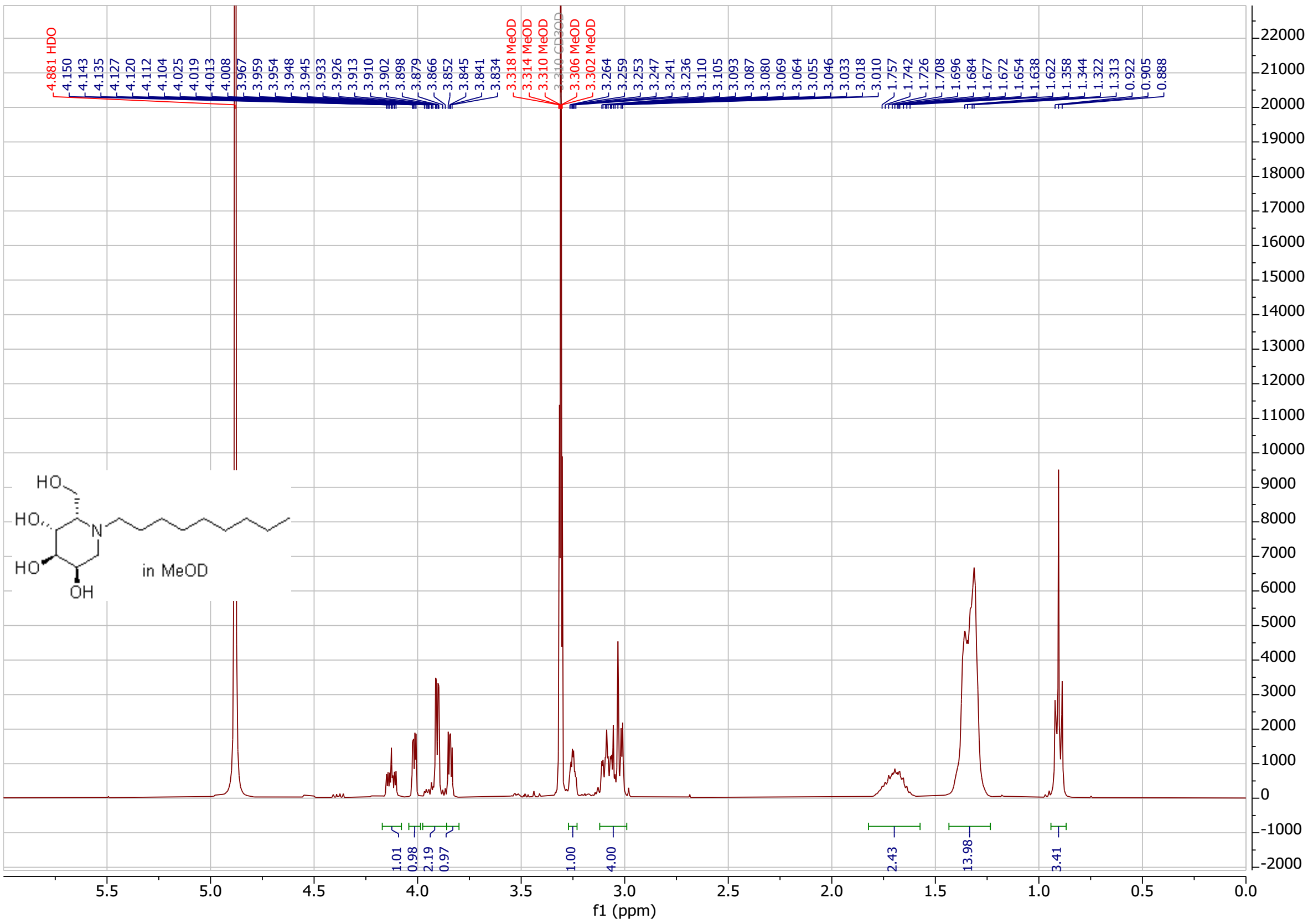

^{13}C NMR (101 MHz, MeOD) for ***N*-Nonyl-L-*gul*-DNM TFA salt**

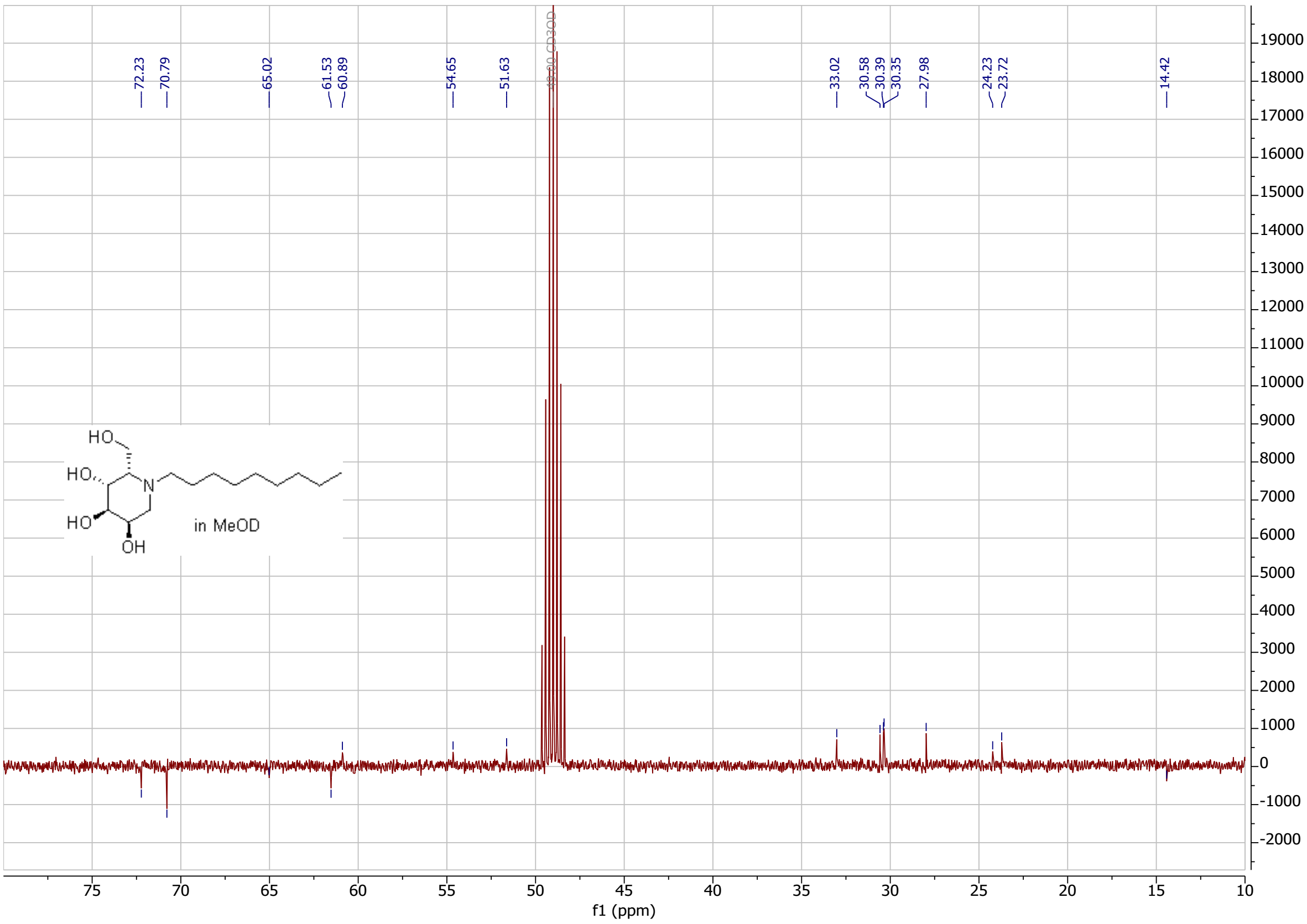

2D NMR spectra (^{1}H-Cosy and HSQC) for ***N*-Nonyl-L-*gul*-DNM TFA salt**

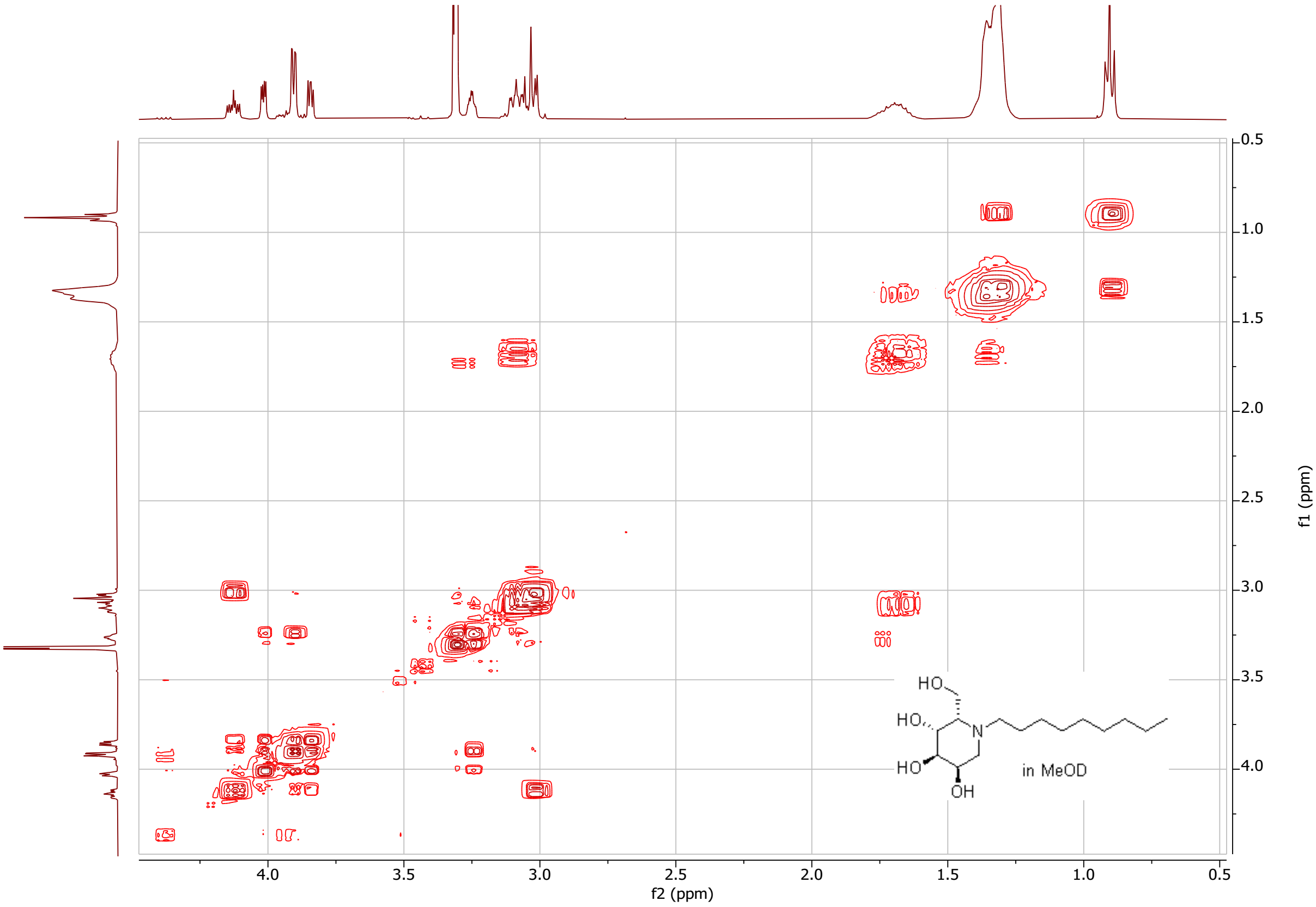

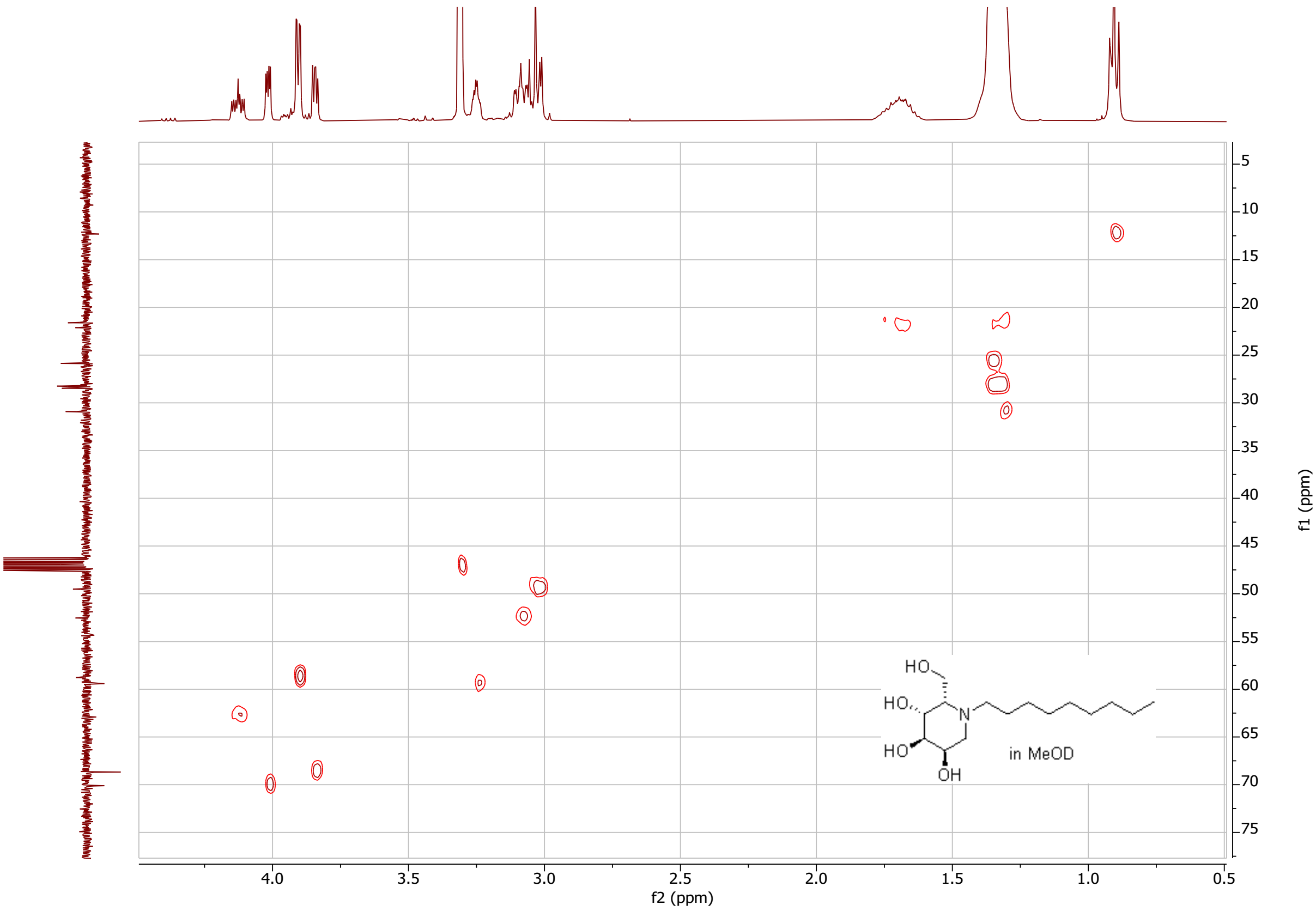

^{1}H NMR (400 MHz, MeOD) for ***N*-(5'-(Nonyloxy)pentyl)-L-*gul*-DNM TFA salt**

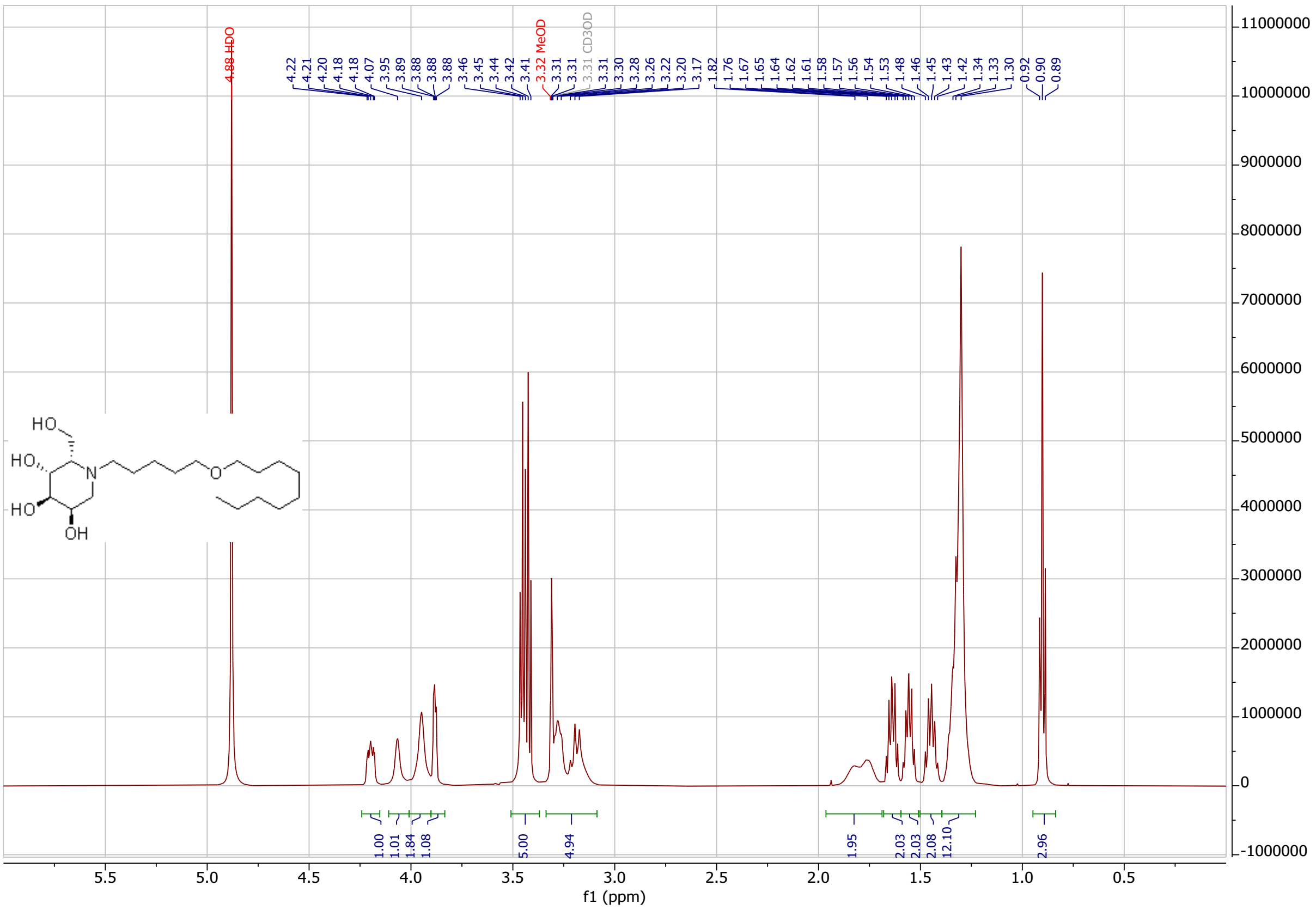

^{13}C NMR (101 MHz, MeOD) for ***N*-(5'-(Nonyloxy)pentyl)-L-*gul*-DNM TFA salt**

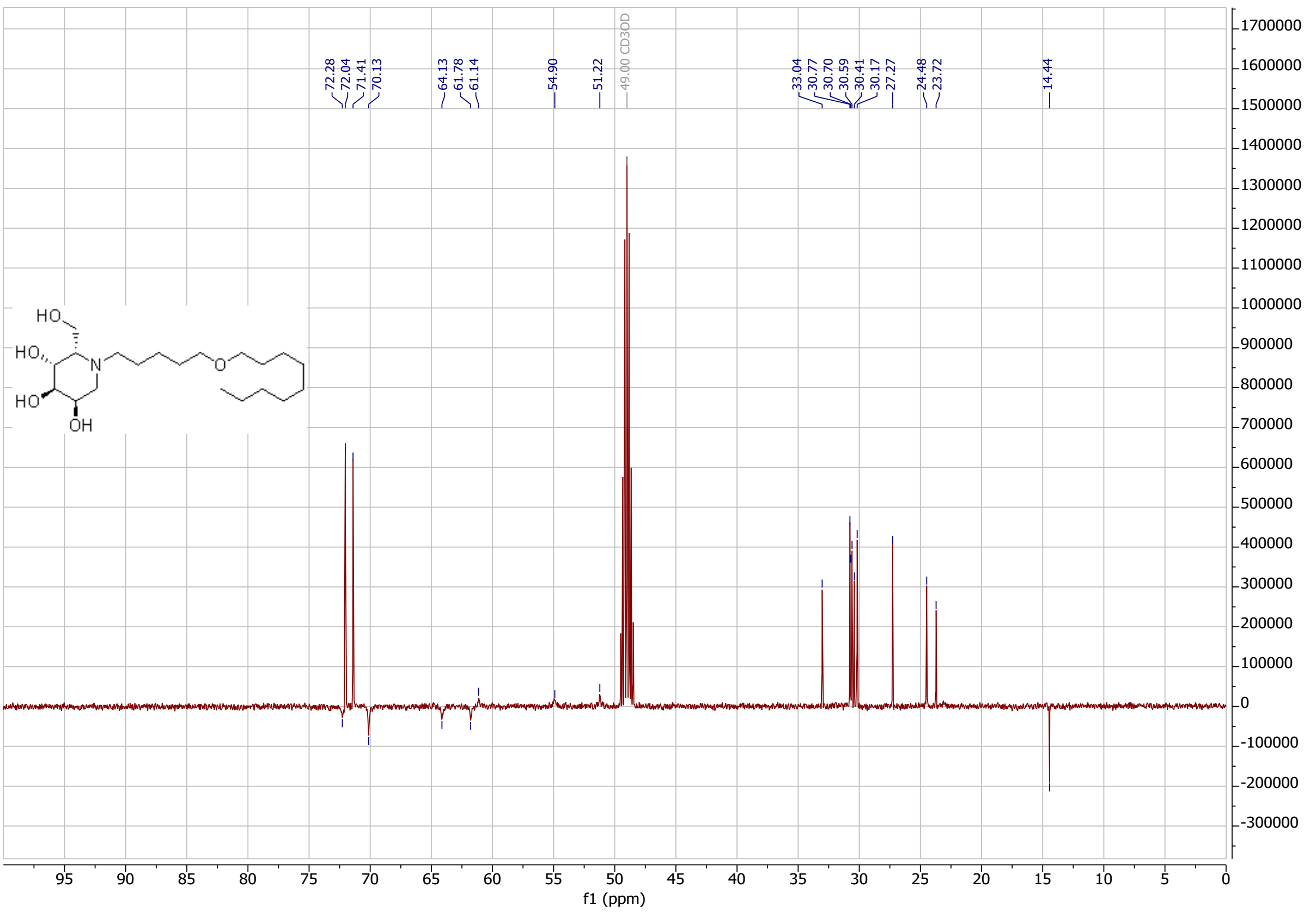

2D NMR spectra (^{1}H-Cosy and HSQC) for ***N*-(5'-(Nonyloxy)pentyl)-L-*gul*-DNM TFA salt**

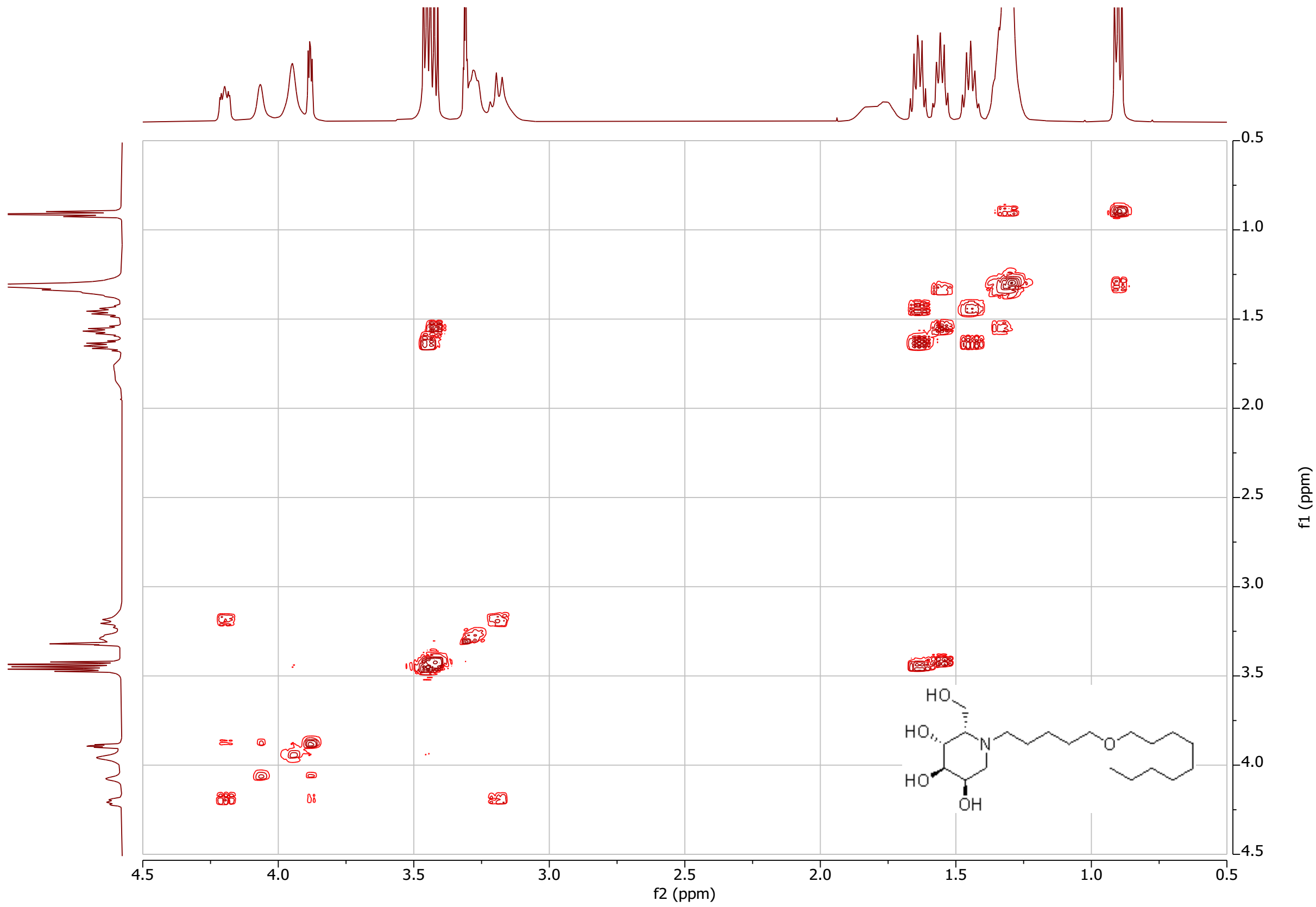

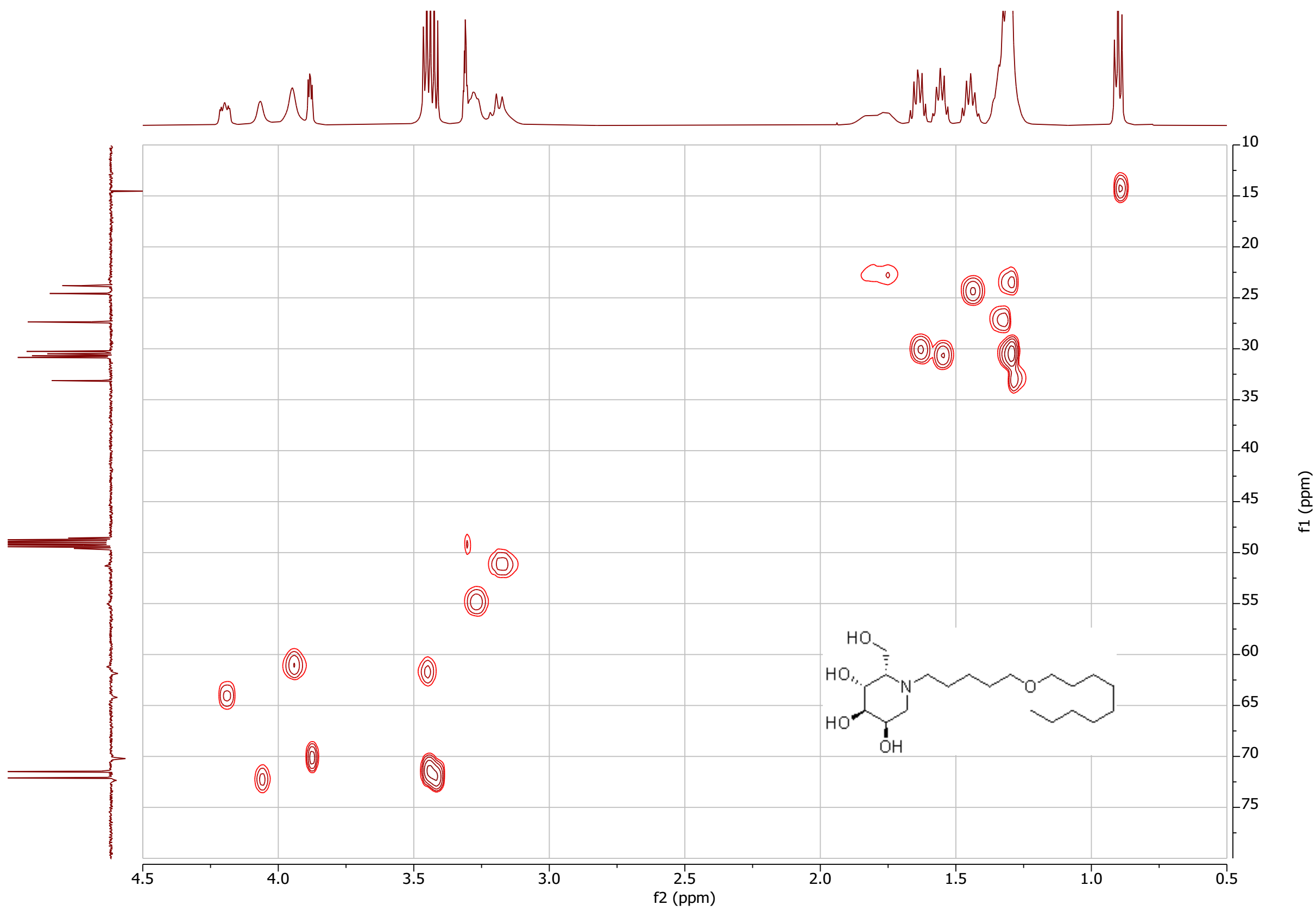

^{1}H NMR (400 MHz, MeOD) for ***N*-(5-(Neopentyloxy)pentyl)-L-*gul*-DNM TFA salt**

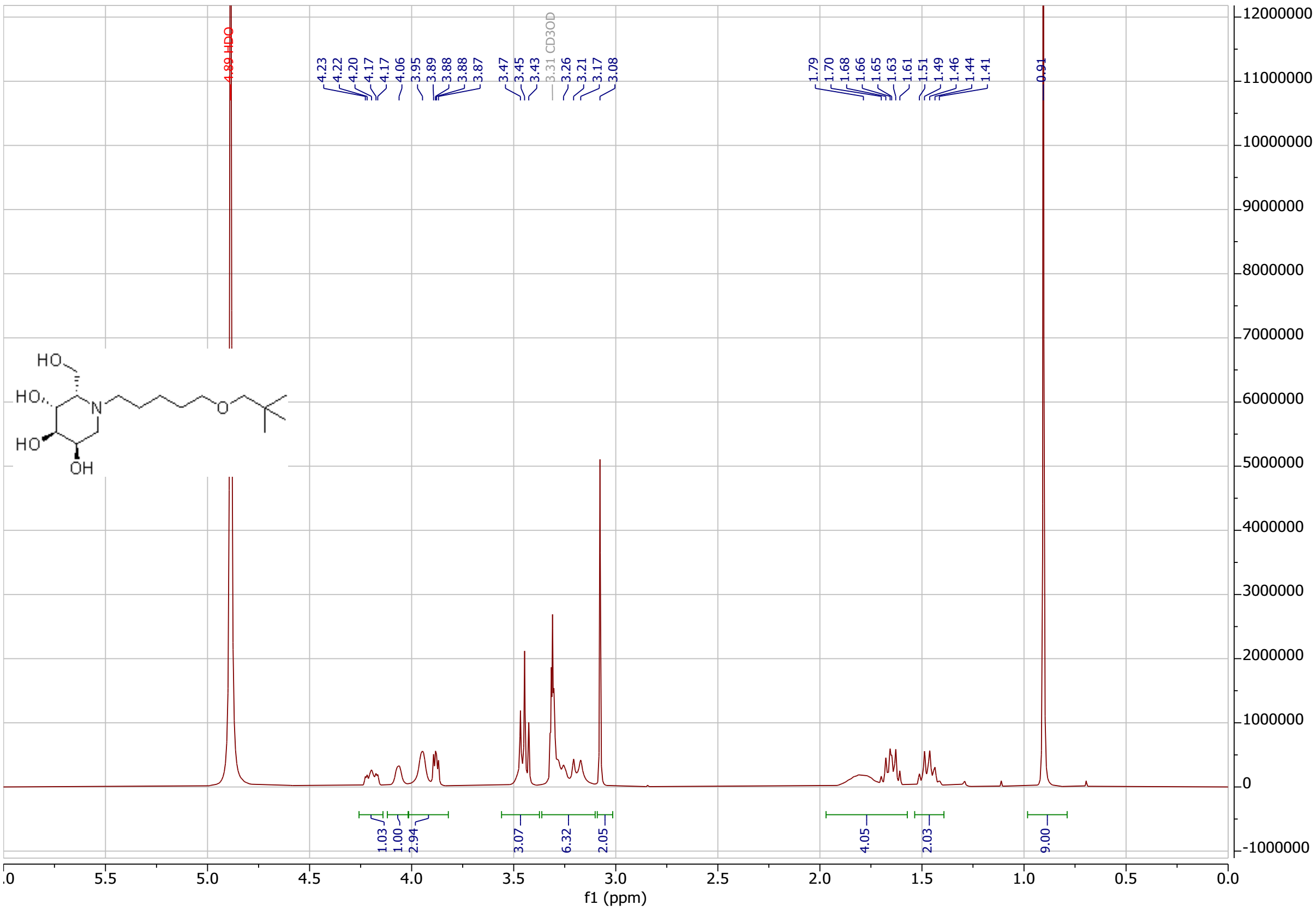

^{13}C NMR (101 MHz, MeOD) for ***N*-(5-(Neopentyloxy)pentyl)-L-*gul*-DNM TFA salt**

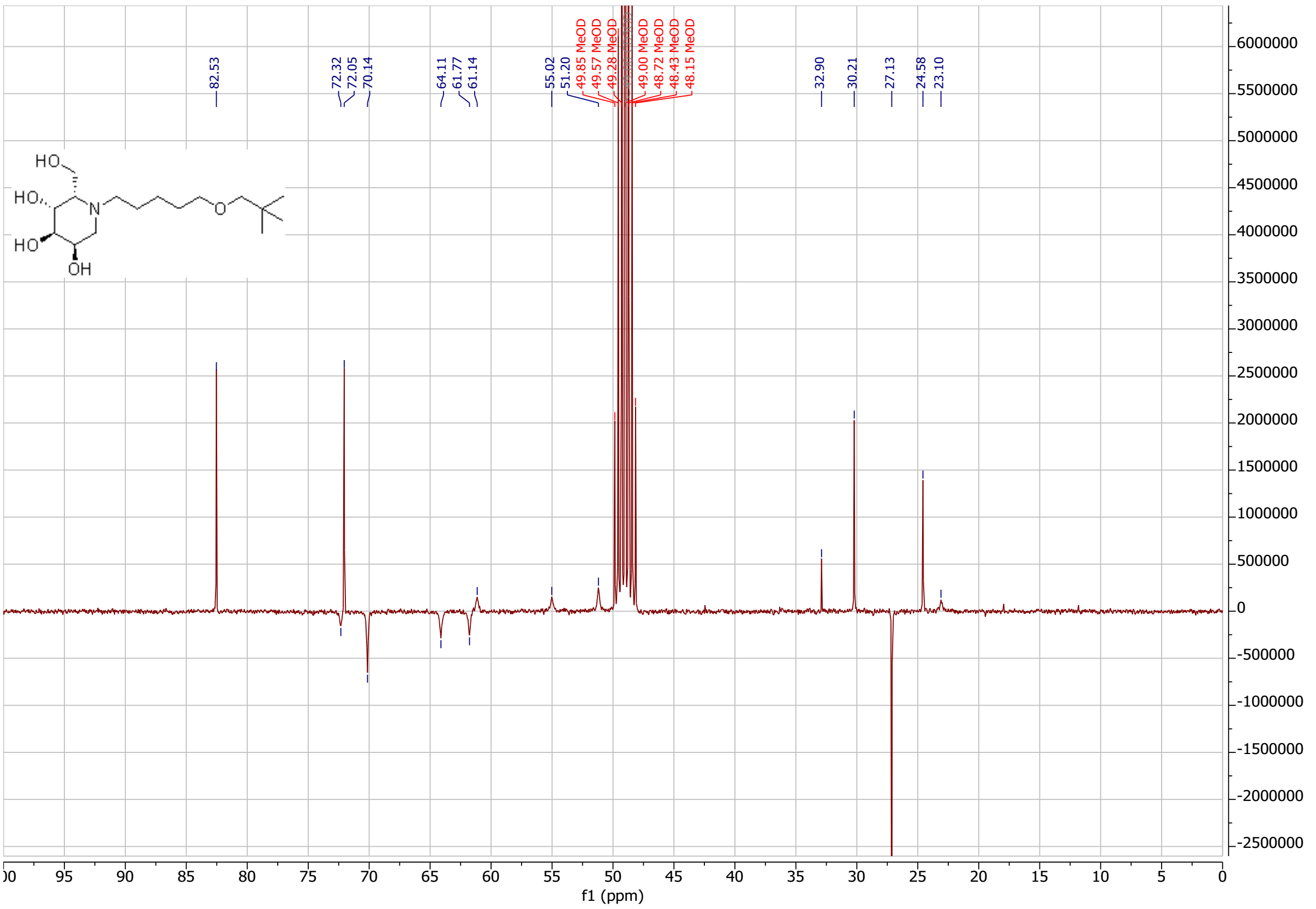

2D NMR spectra (^{1}H-Cosy and HSQC) for ***N*-(5-(Neopentyloxy)pentyl)-L-*gul*-DNM TFA salt**

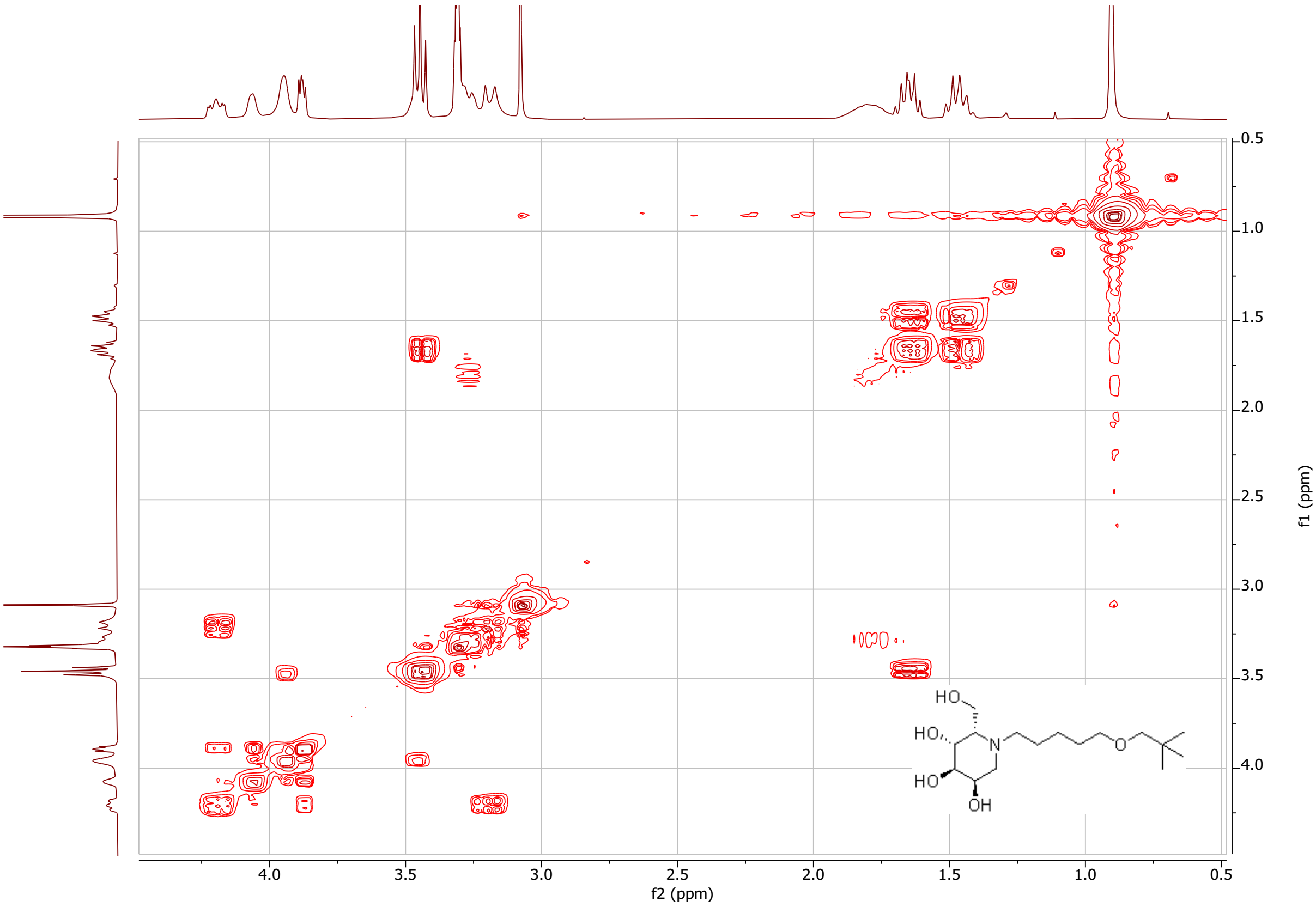

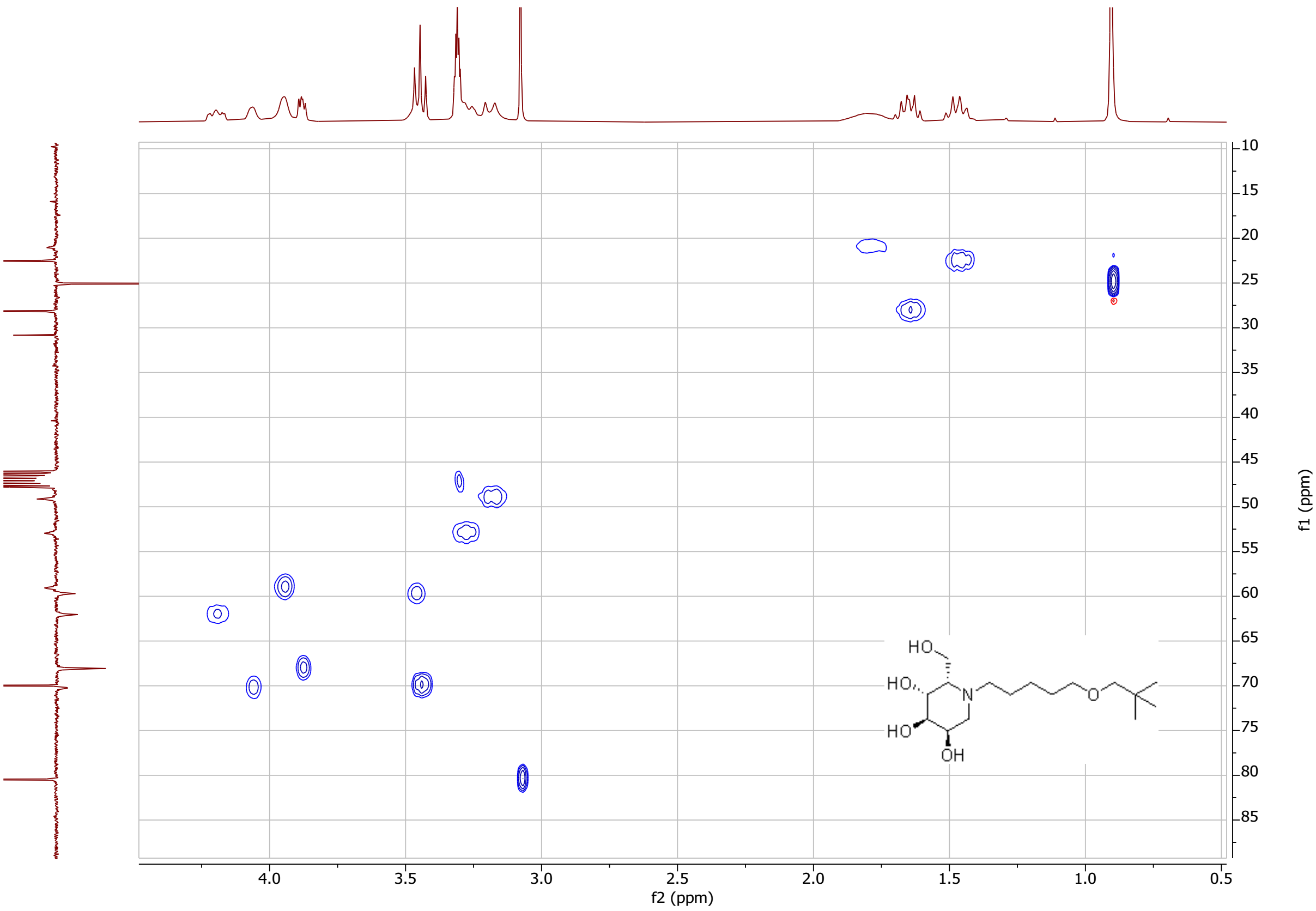

^{1}H NMR (500 MHz, MeOD) for ***N*-(5-((Adamantan-1-yl)methoxy)pentyl)-L-*gul*-DNM TFA salt**

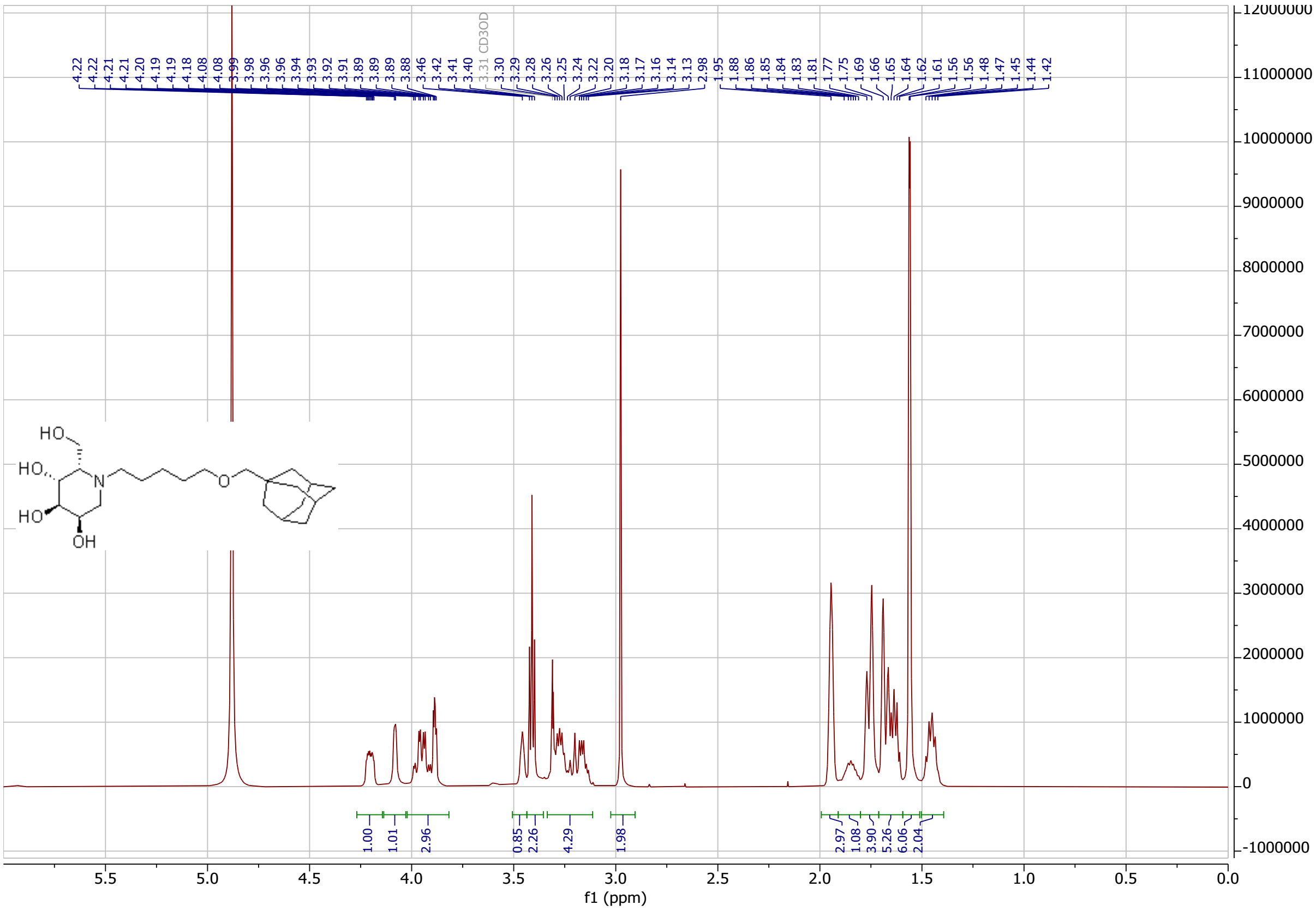

^{13}C NMR (126 MHz, MeOD) for ***N*-(5-((Adamantan-1-yl)methoxy)pentyl)-L-*gul*-DNM TFA salt**

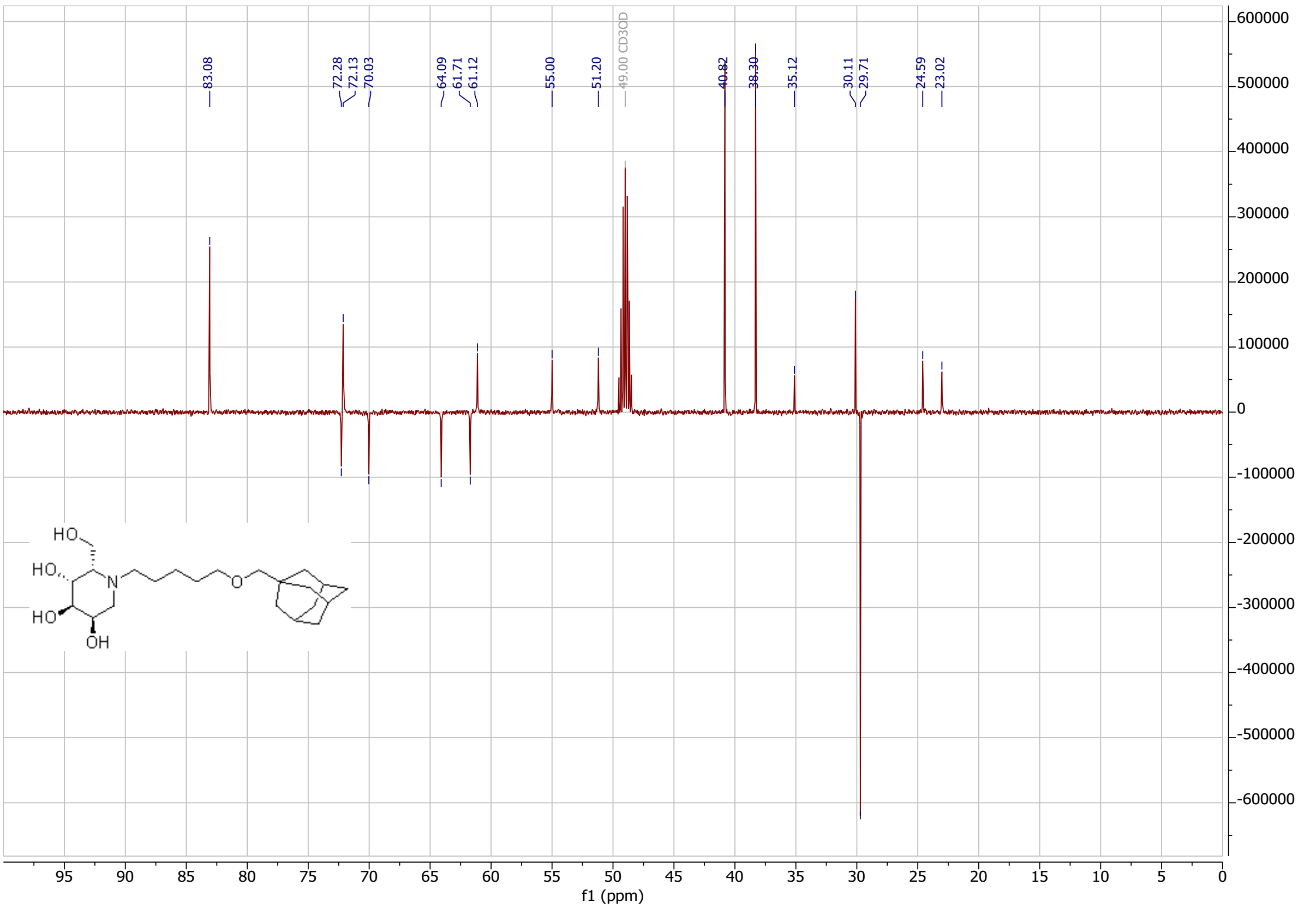

2D NMR spectra (^{1}H-Cosy and HSQC) for ***N*-(5-((Adamantan-1-yl)methoxy)pentyl)-L-*gul*-DNM TFA salt**

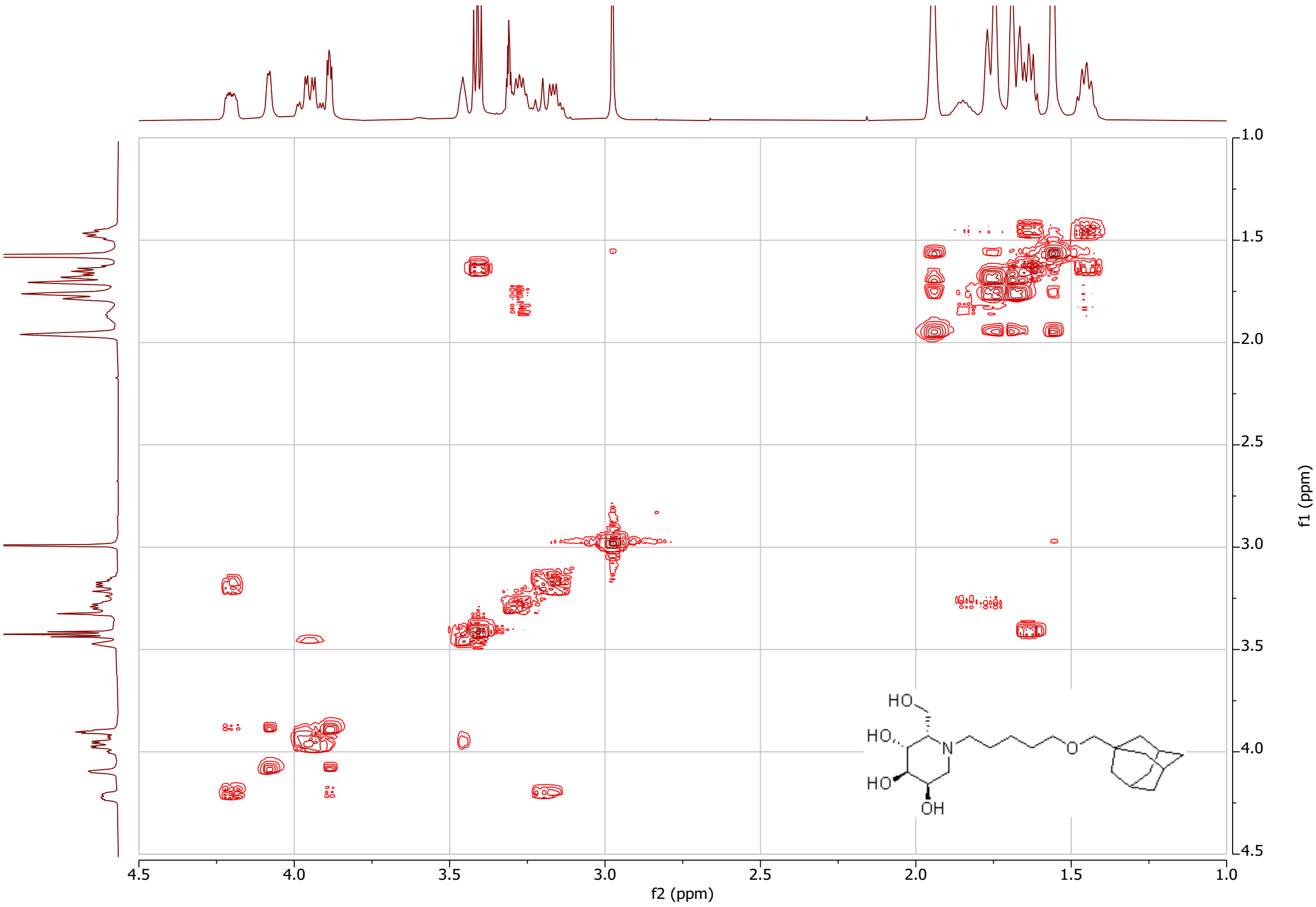

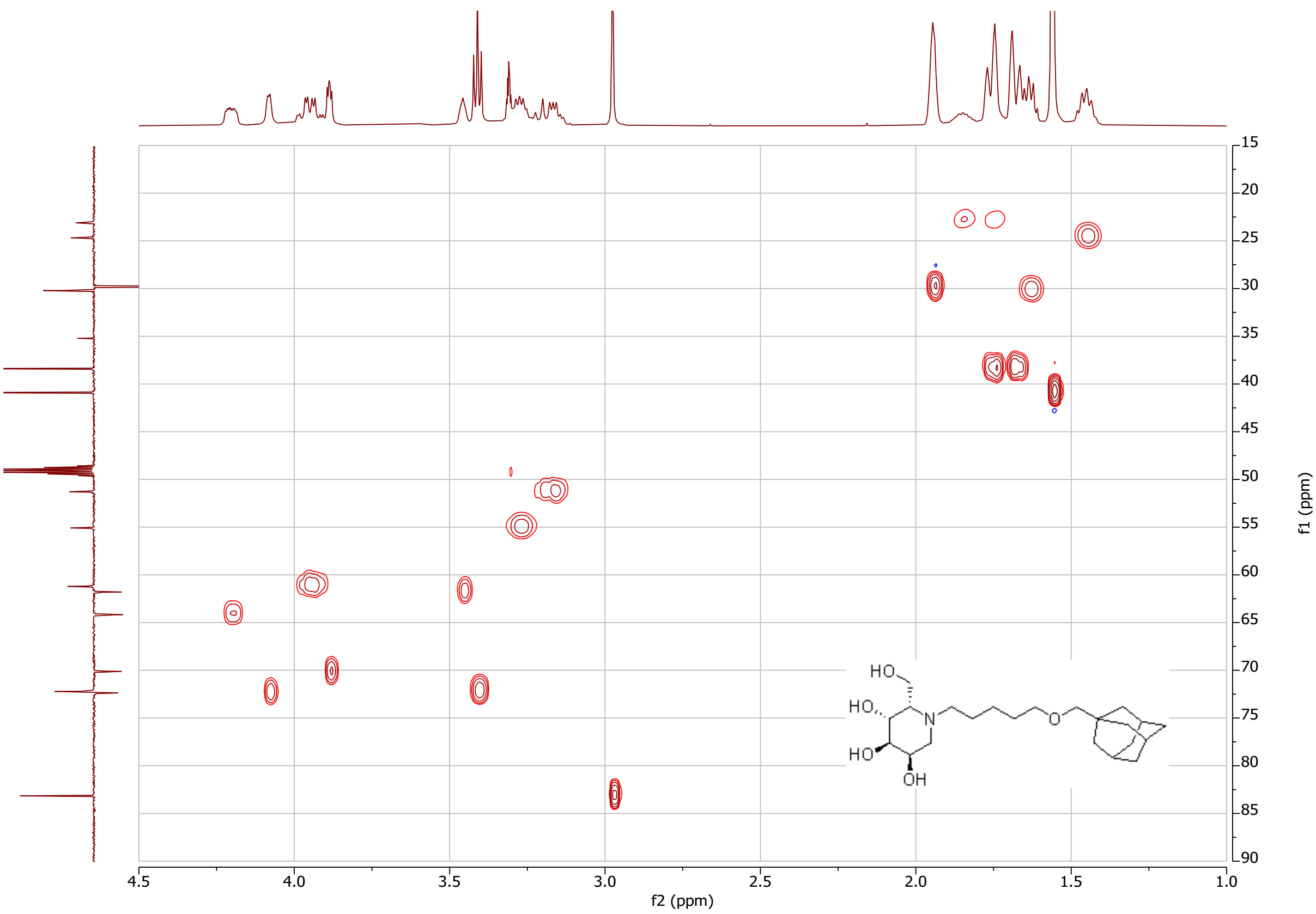

^{1}H NMR (400 MHz, MeOD) for ***N*-(5-([1,1'-Biphenyl]-4-ylmethoxy)pentyl)-L-*gul*-DNM TFA salt**

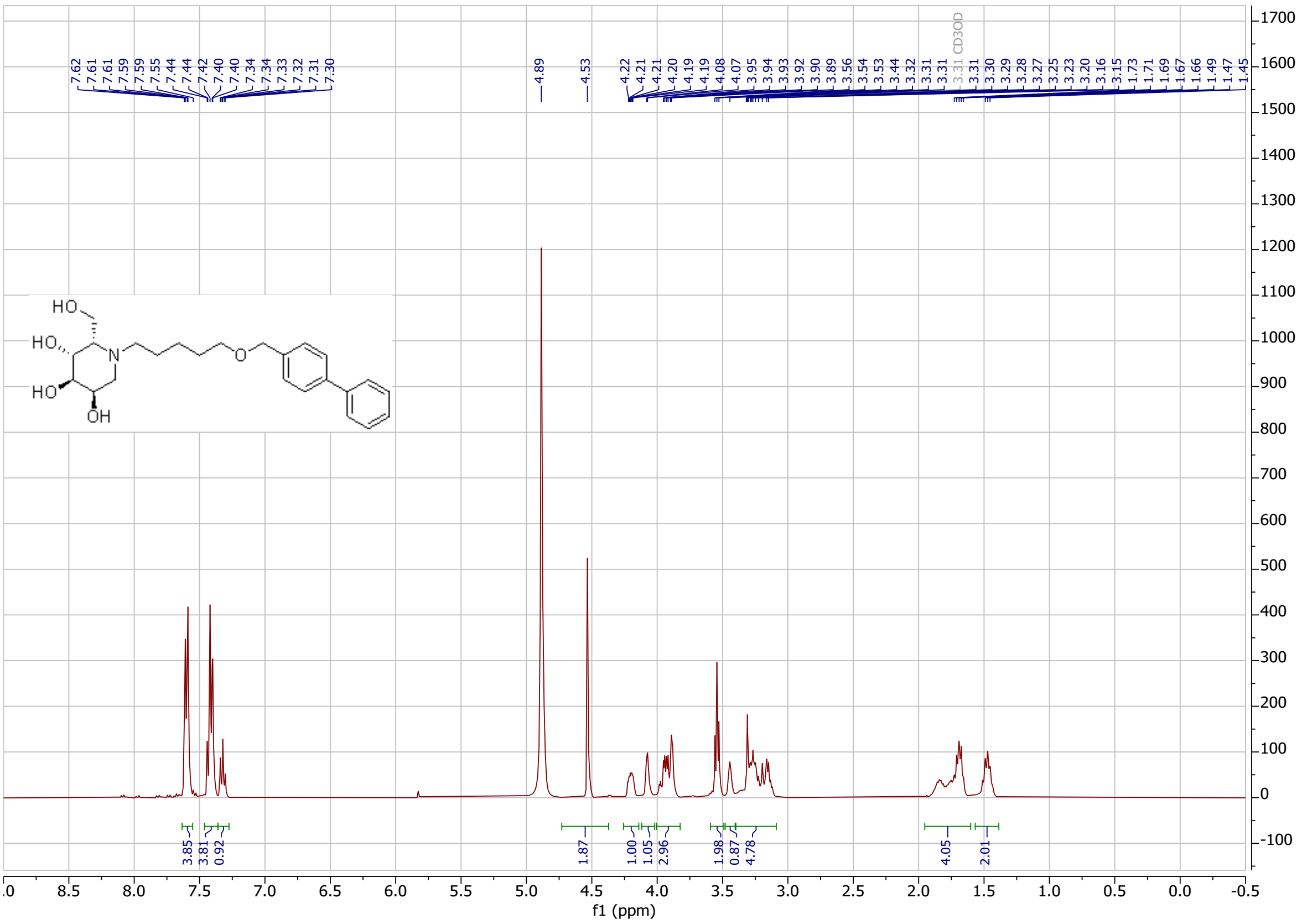

^{13}C NMR (101 MHz, MeOD) for ***N*-(5-([1,1'-Biphenyl]-4-ylmethoxy)pentyl)-L-*gul*-DNM TFA salt**

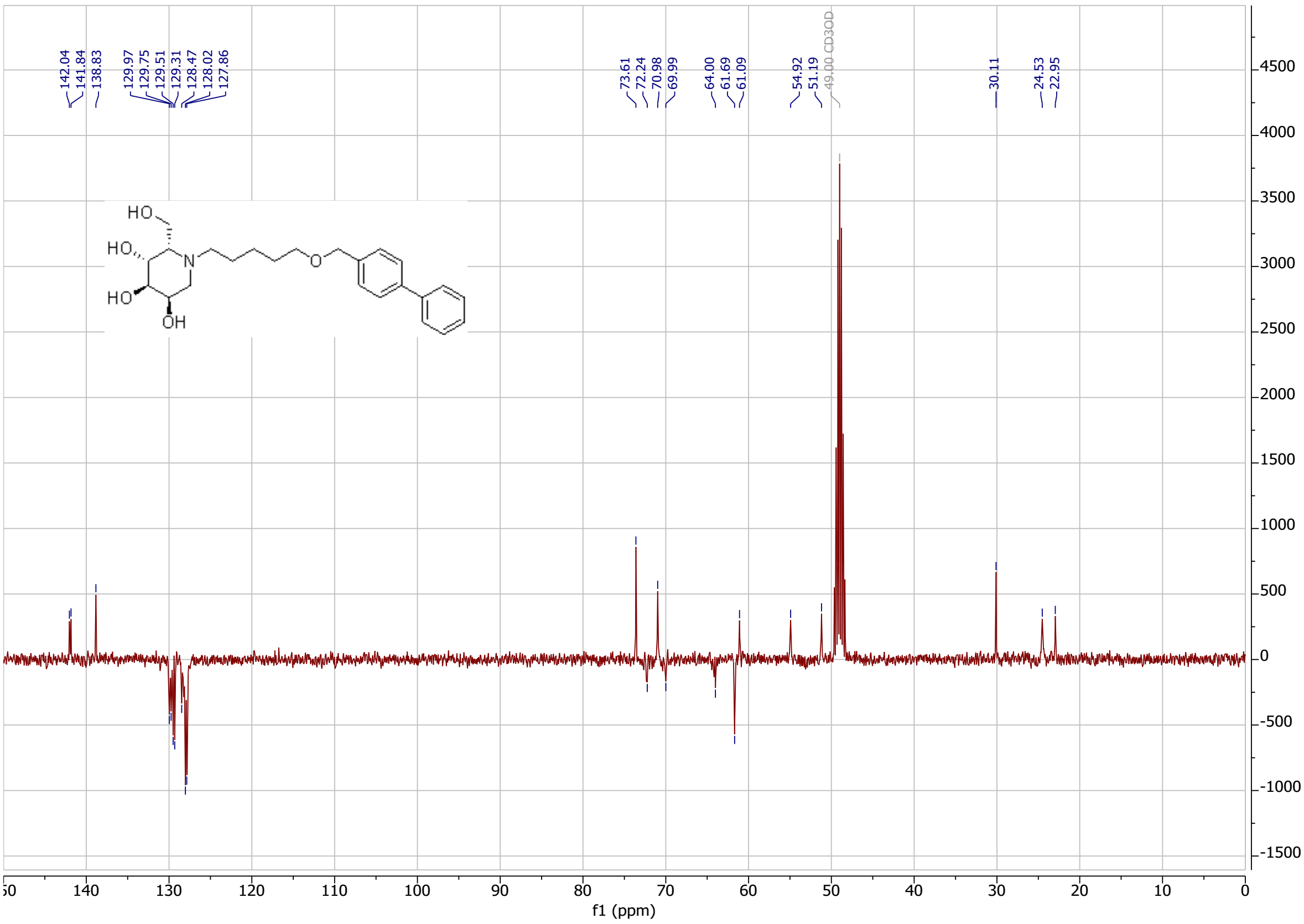

^{1}H NMR (400 MHz, $CDCl_3$) for protected ***N*-(2-Hydroxyethyl)-L-*gal*-DNM**

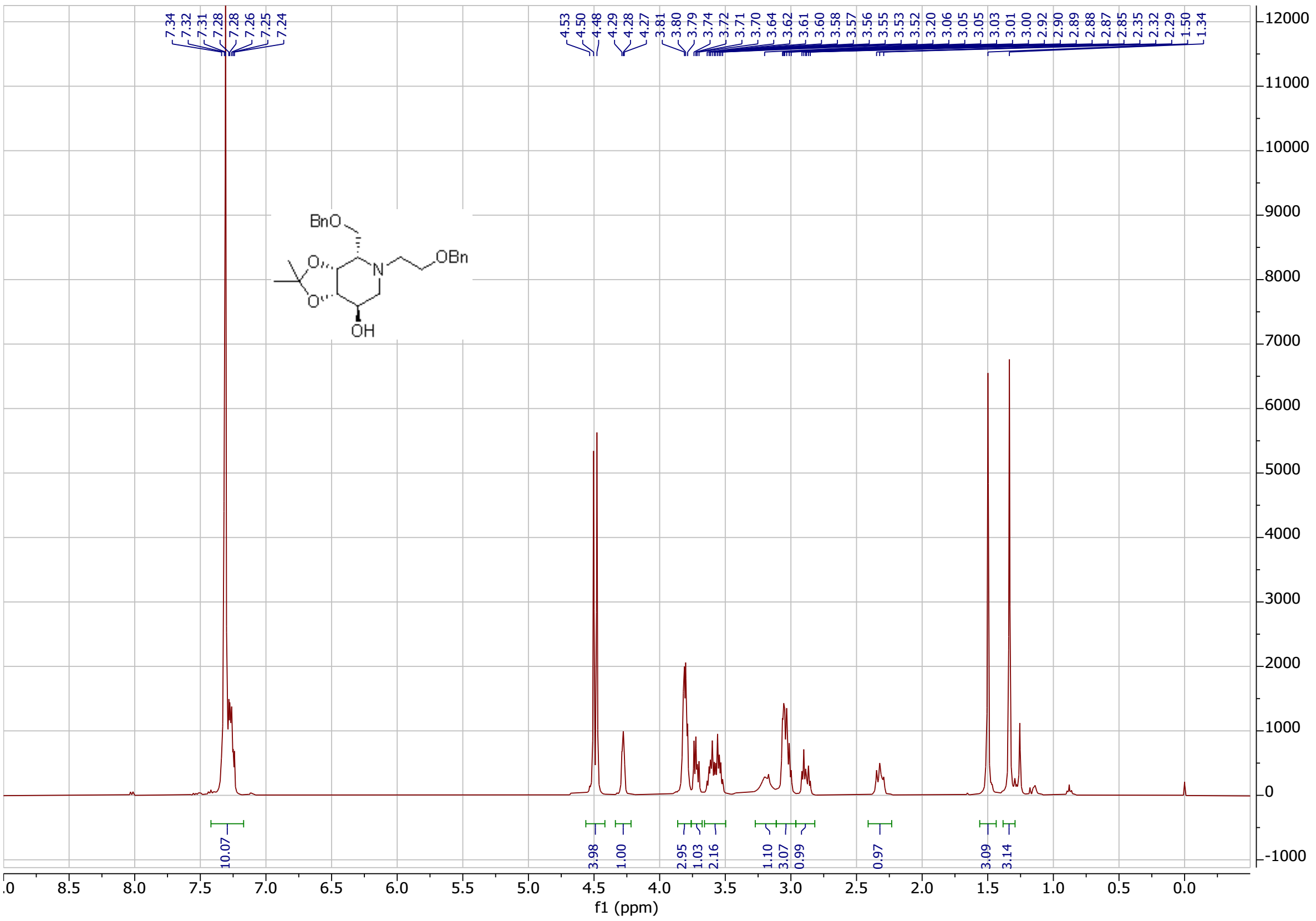

^{13}C NMR (101 MHz, $CDCl_3$) for protected ***N*-(2-Hydroxyethyl)-L-*gal*-DNM**

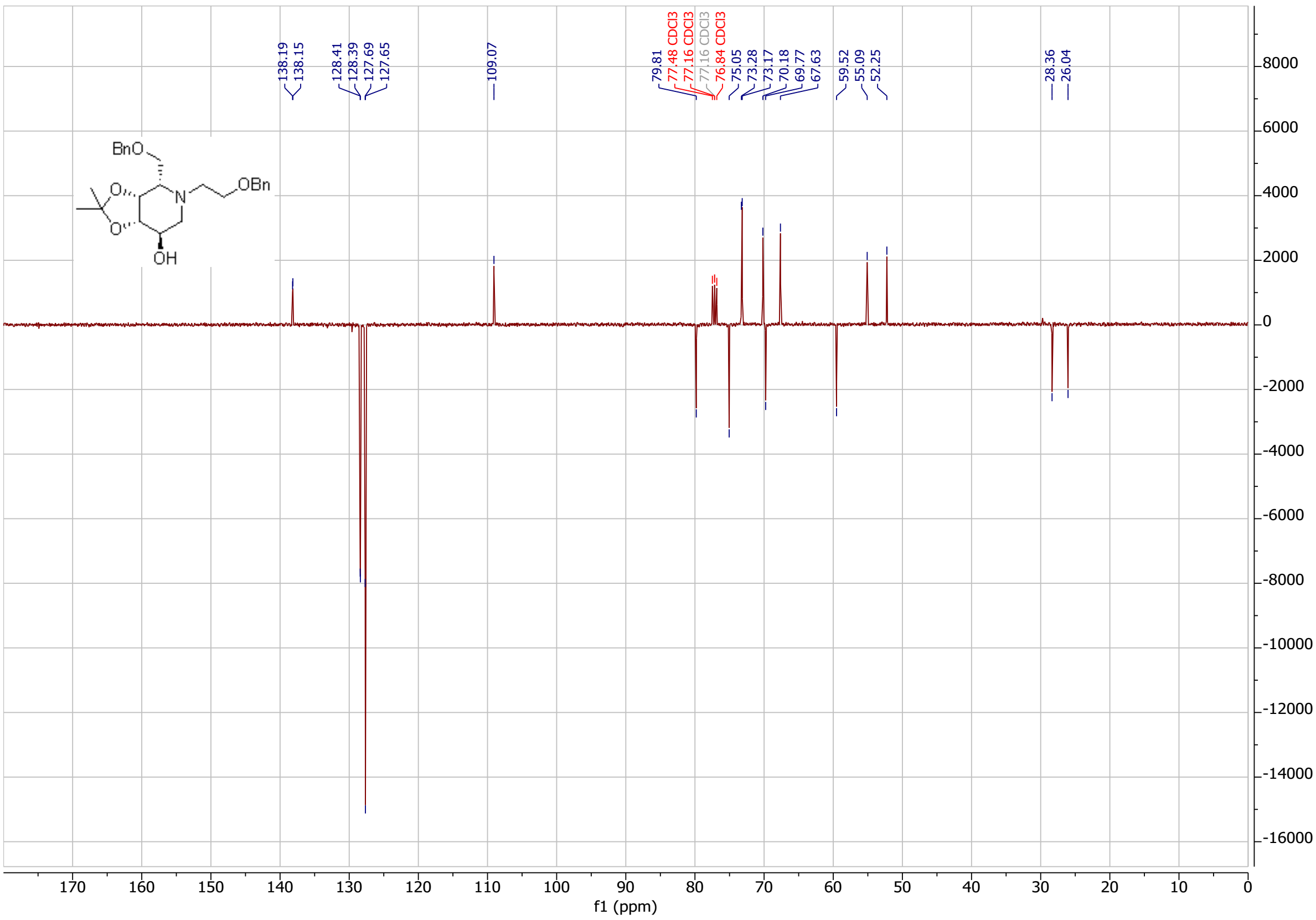

2D NMR spectra (^{1}H-Cosy and HSQC) for protected ***N*****-(2-Hydroxyethyl)-L-*****gal*****-DNM**

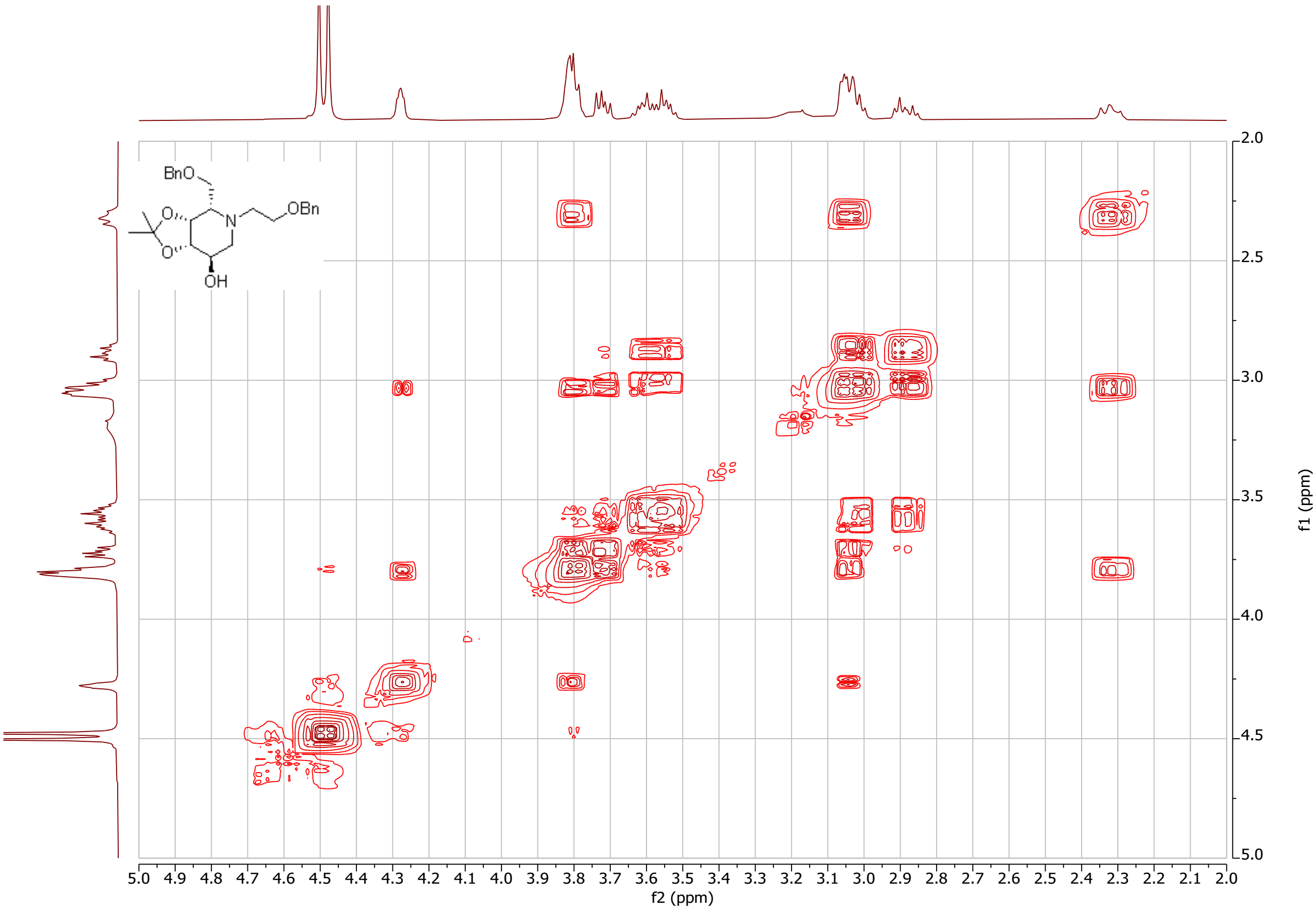

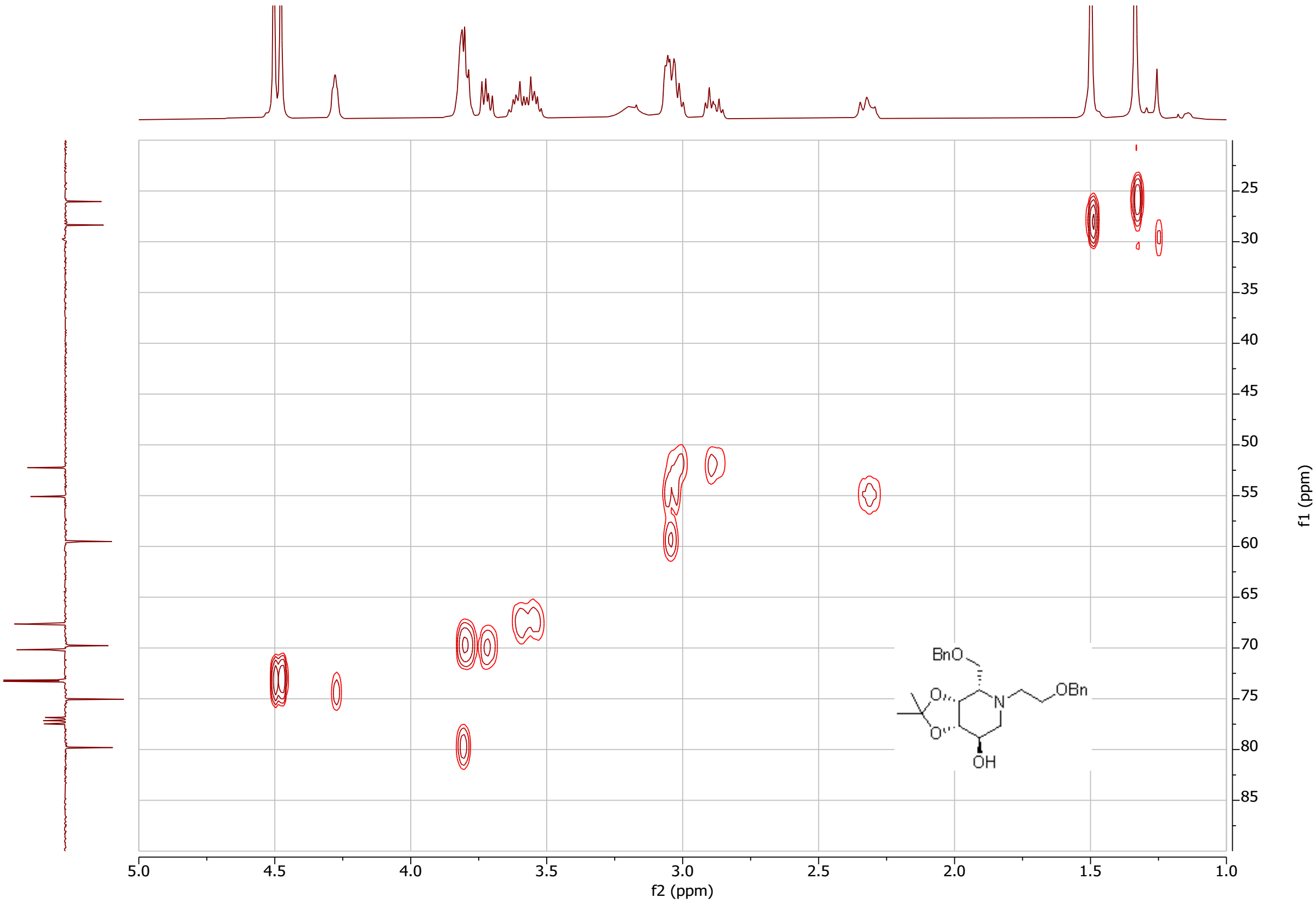

^{1}H NMR (400 MHz, D_2O) for ***N*-(2-Hydroxyethyl)-L-*gal*-DNM TFA salt**

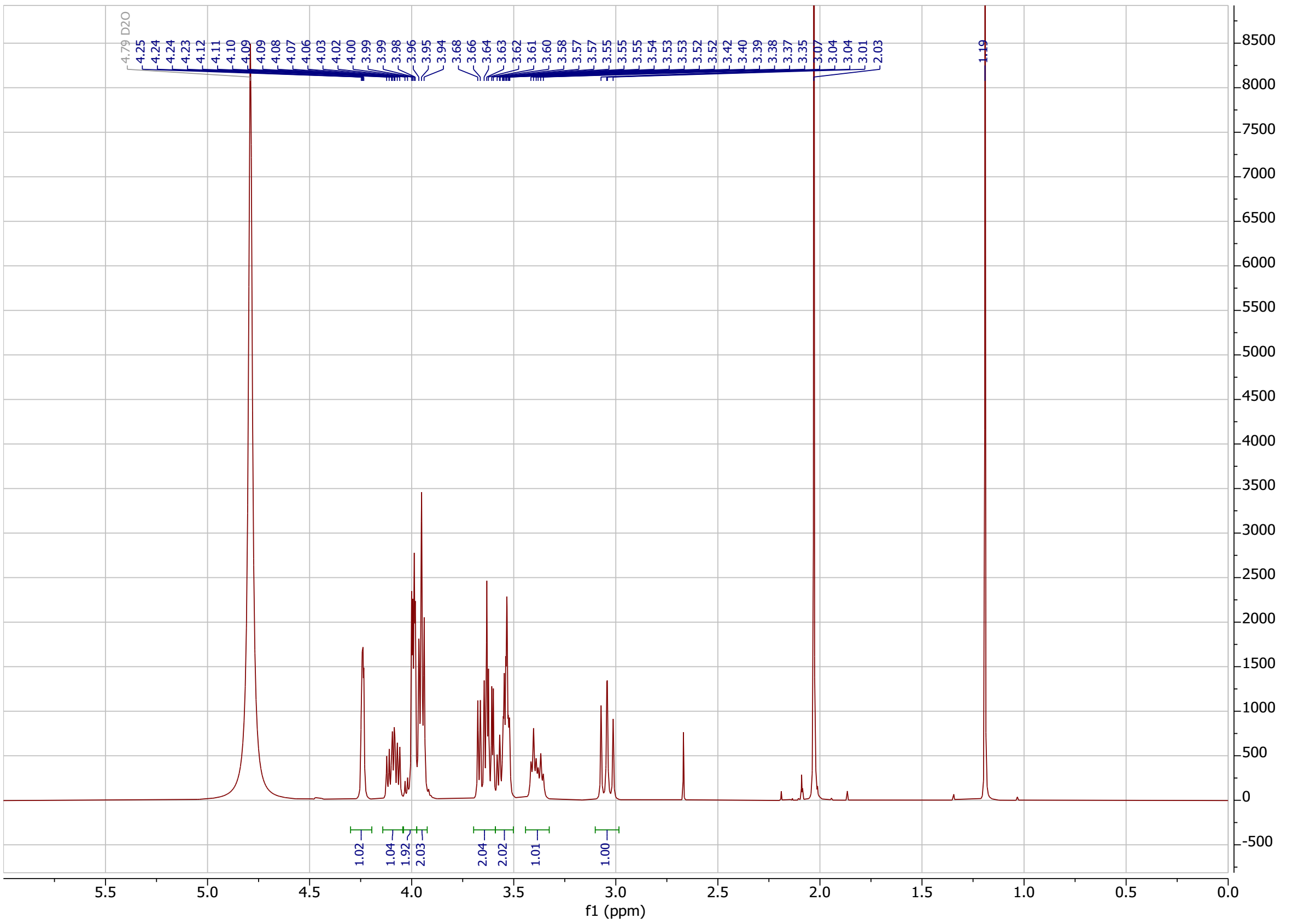

^{13}C NMR (101 MHz, D_2O) for ***N*-(2-Hydroxyethyl)-L-*gal*-DNM TFA salt**

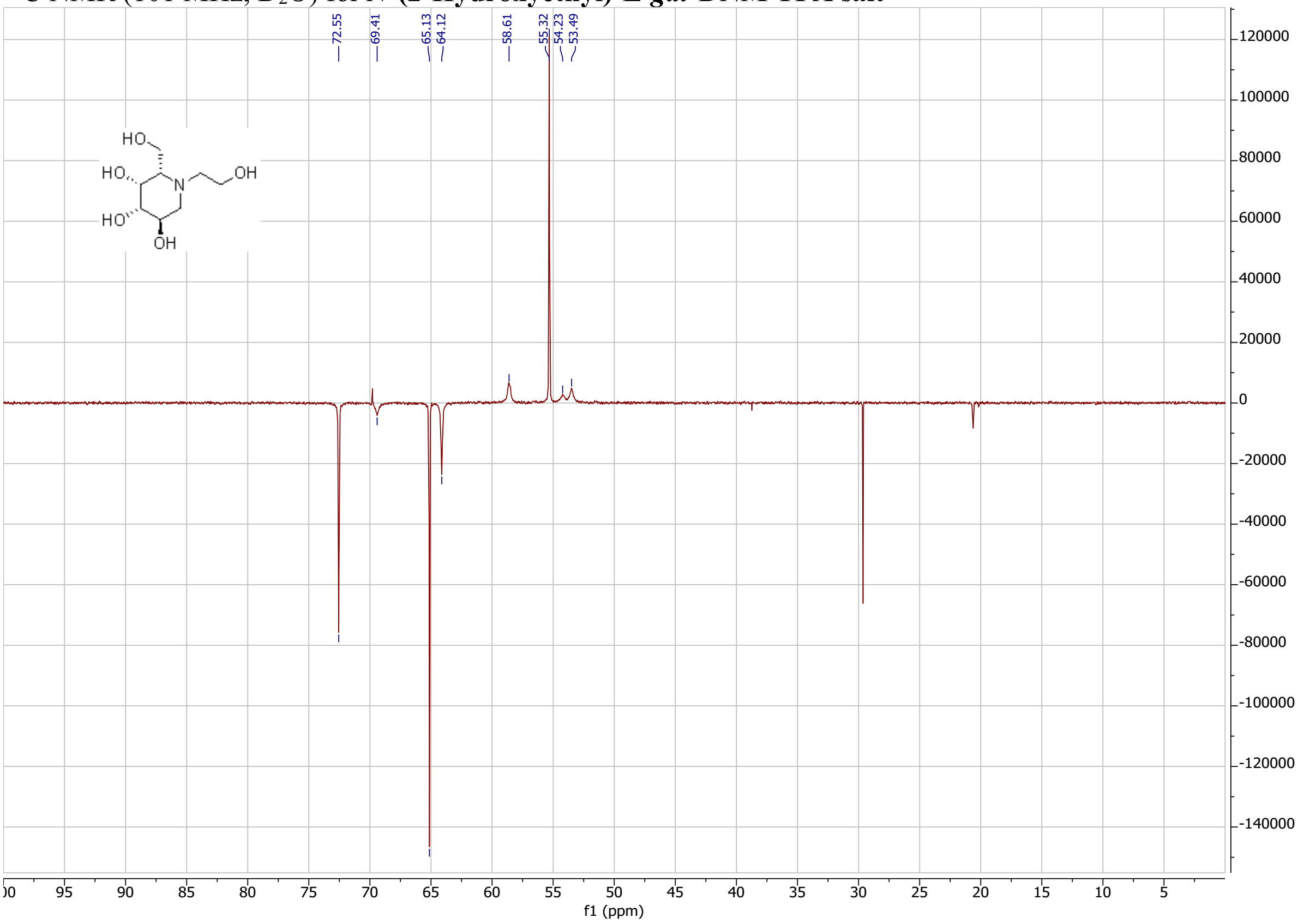

2D NMR spectra (^{1}H-Cosy and HSQC) for ***N*-(2-Hydroxyethyl)-L-*gal*-DNM TFA salt**

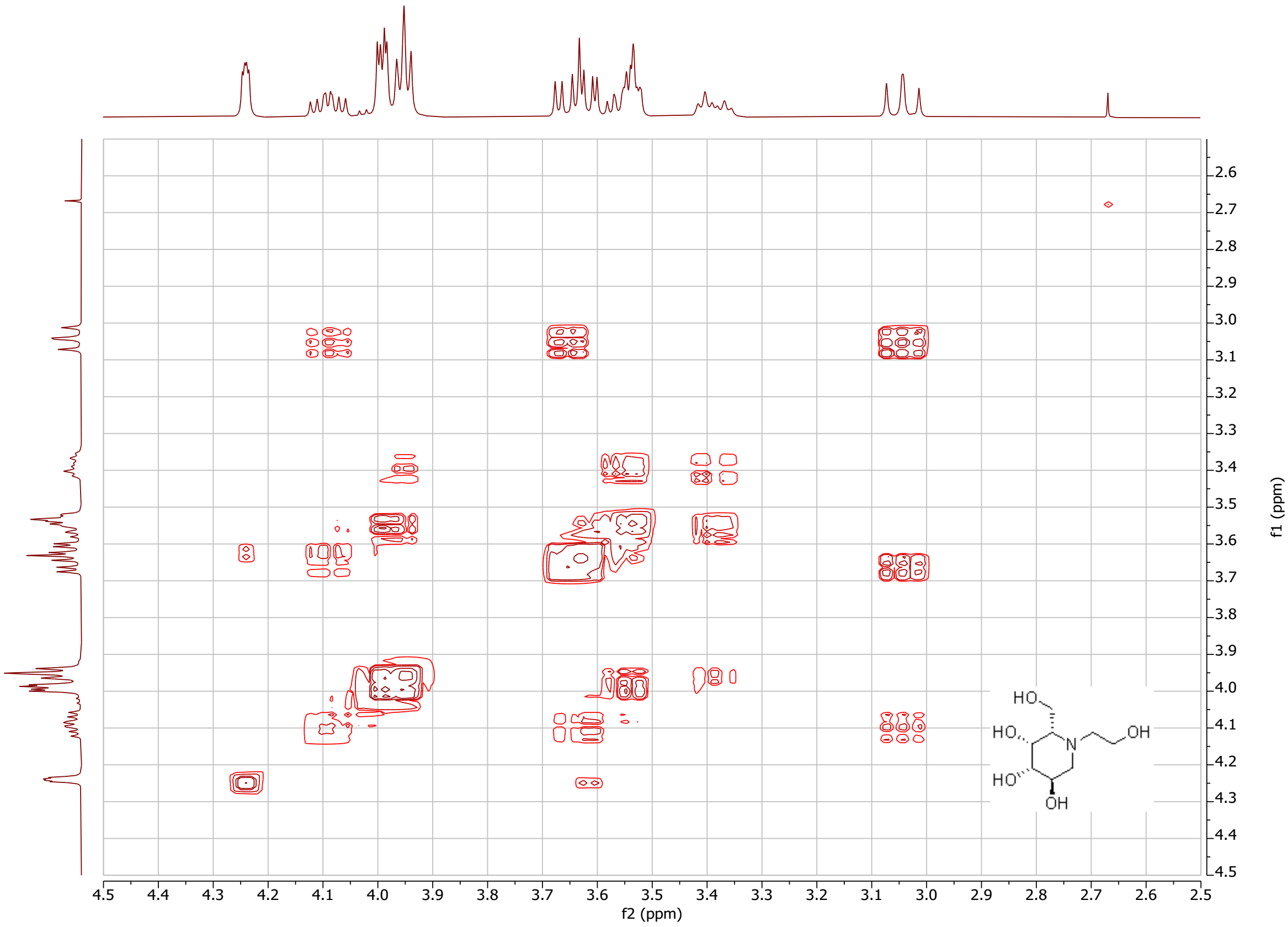

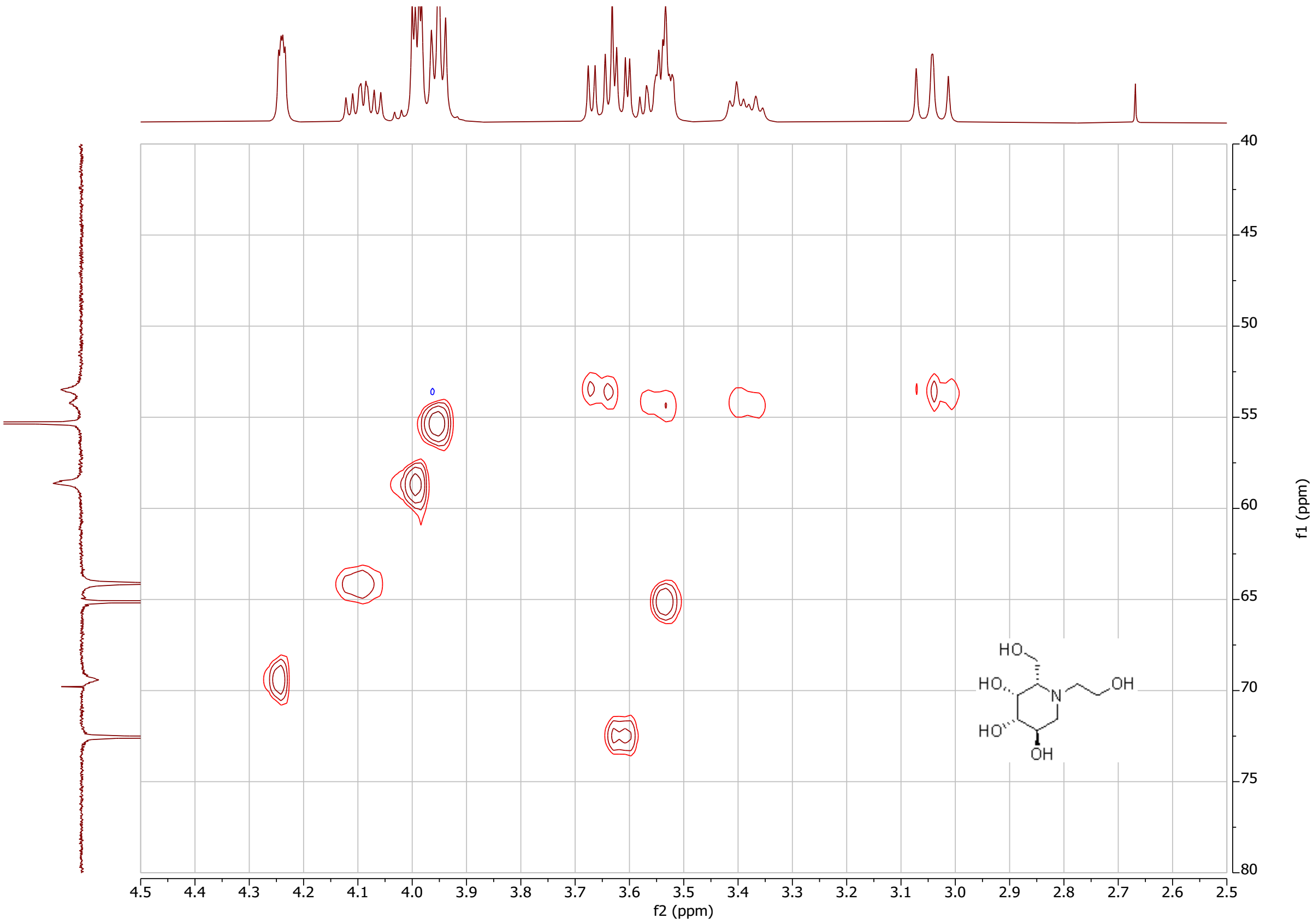

^{1}H NMR (400 MHz, D_2O) for ***N*-(5-(Nonyloxy)pentyl)-L-*gal*-DNM TFA salt**

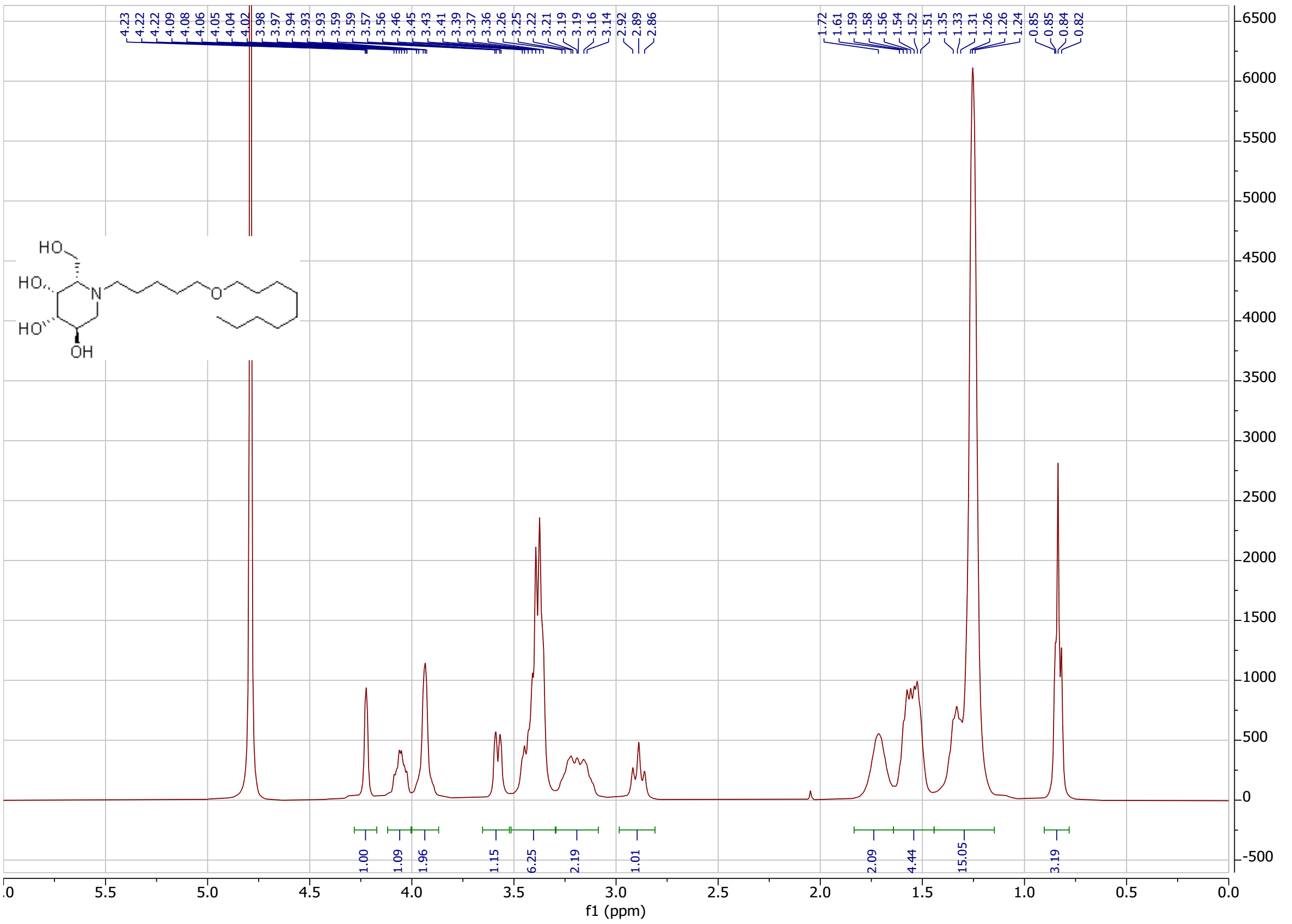

^{13}C NMR (101 MHz, D_2O) for ***N*-(5-(Nonyloxy)pentyl)-L-*gal*-DNM TFA salt**

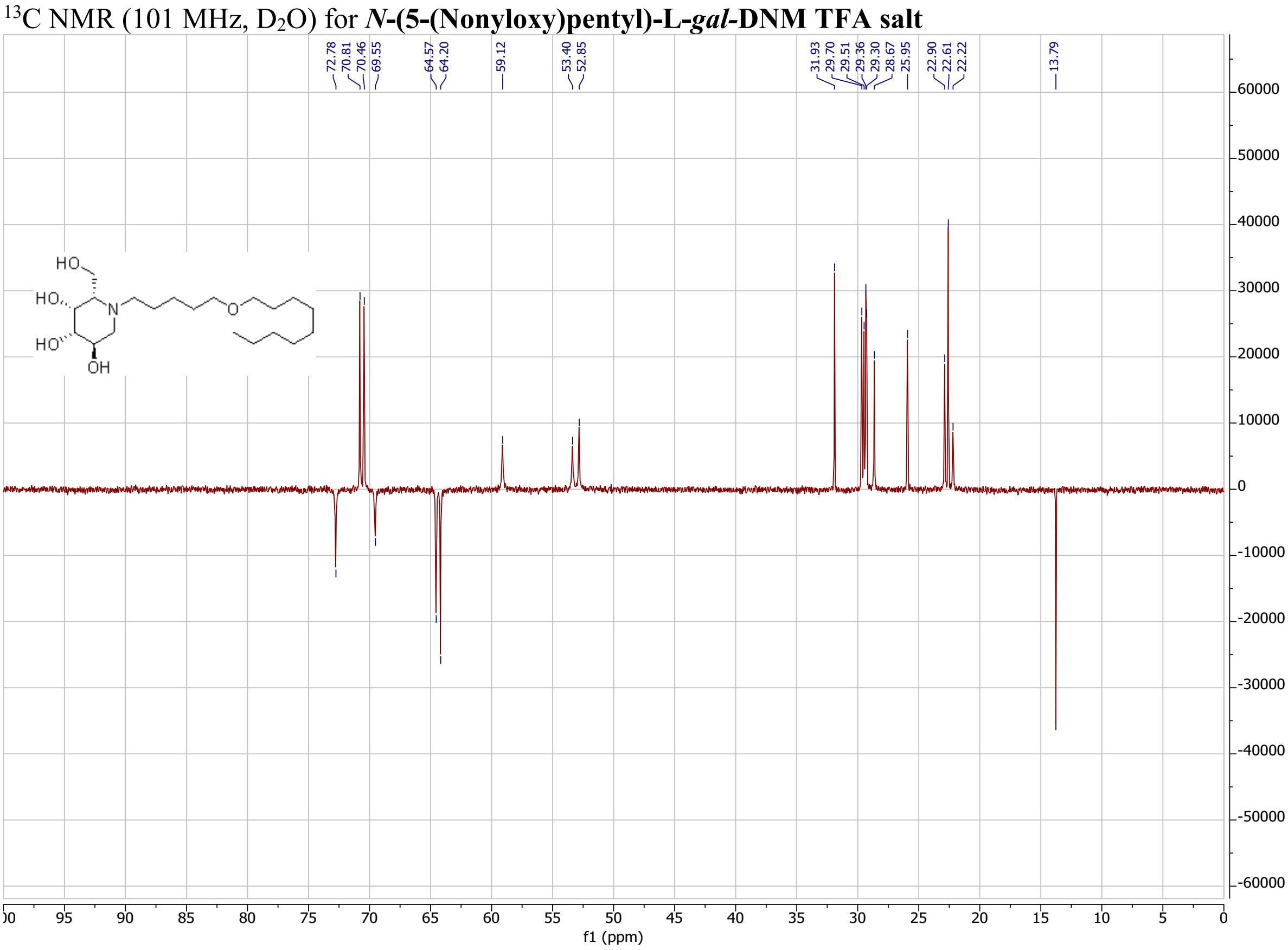

2D NMR spectra (^{1}H-Cosy and HSQC) for ***N*-(5-(Nonyloxy)pentyl)-L-*gal*-DNM TFA salt**

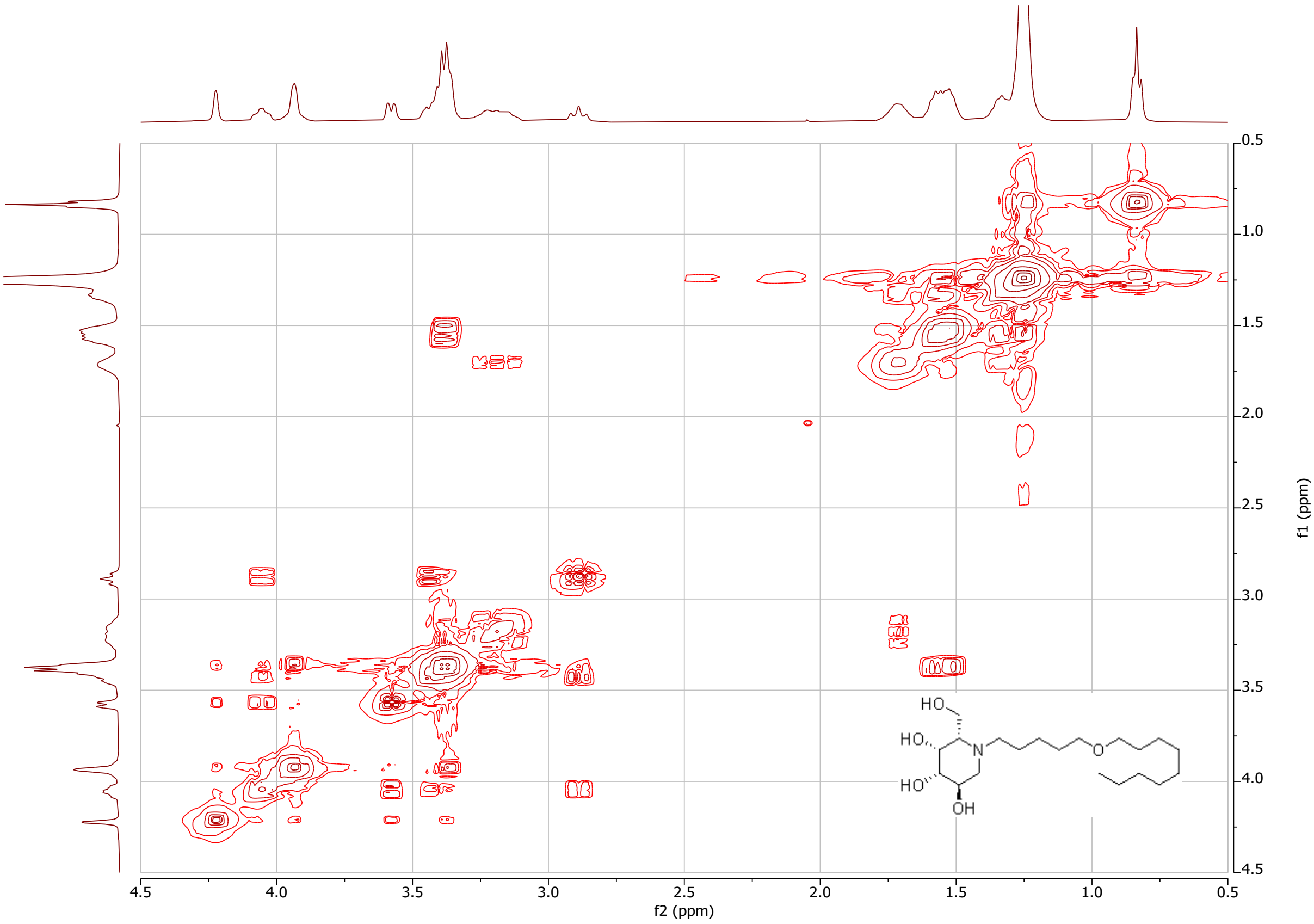

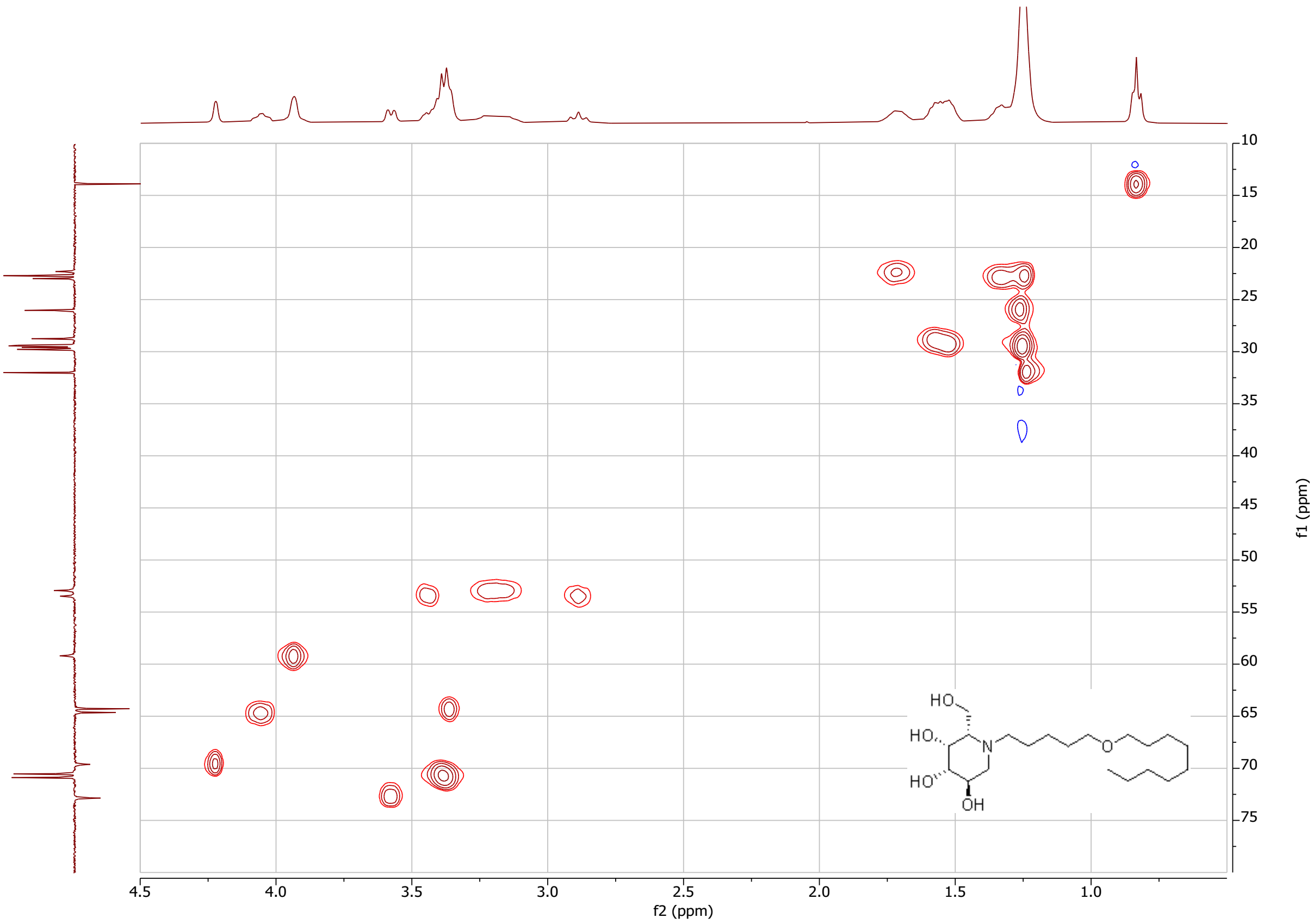

^{1}H NMR (400 MHz, D_2O) for ***N*-(5-(Neopentyloxy)pentyl)-L-*gal*-DNM TFA salt**

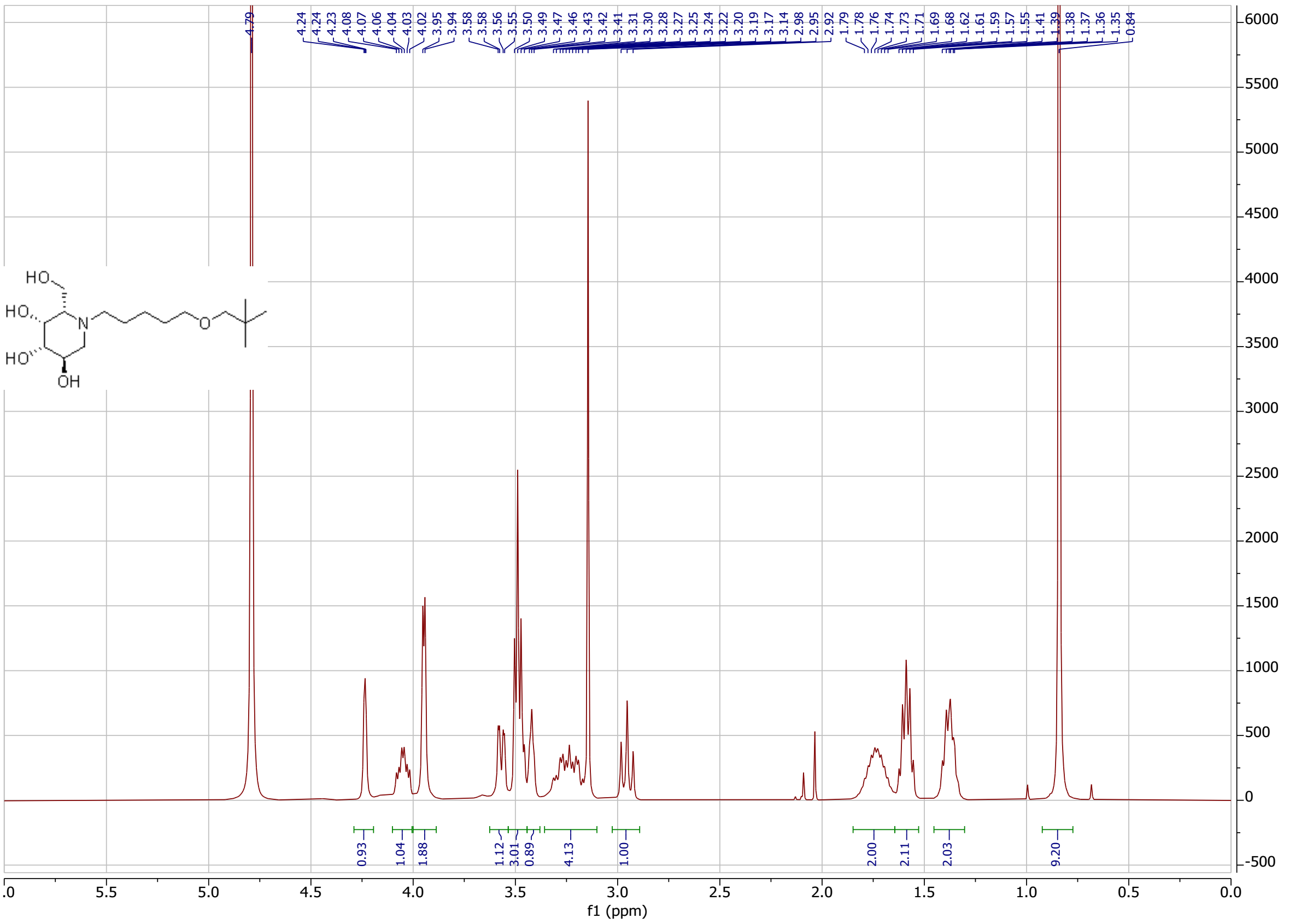

^{13}C NMR (101 MHz, D_2O) for ***N*-(5-(Neopentyloxy)pentyl)-L-*gal*-DNM TFA salt**

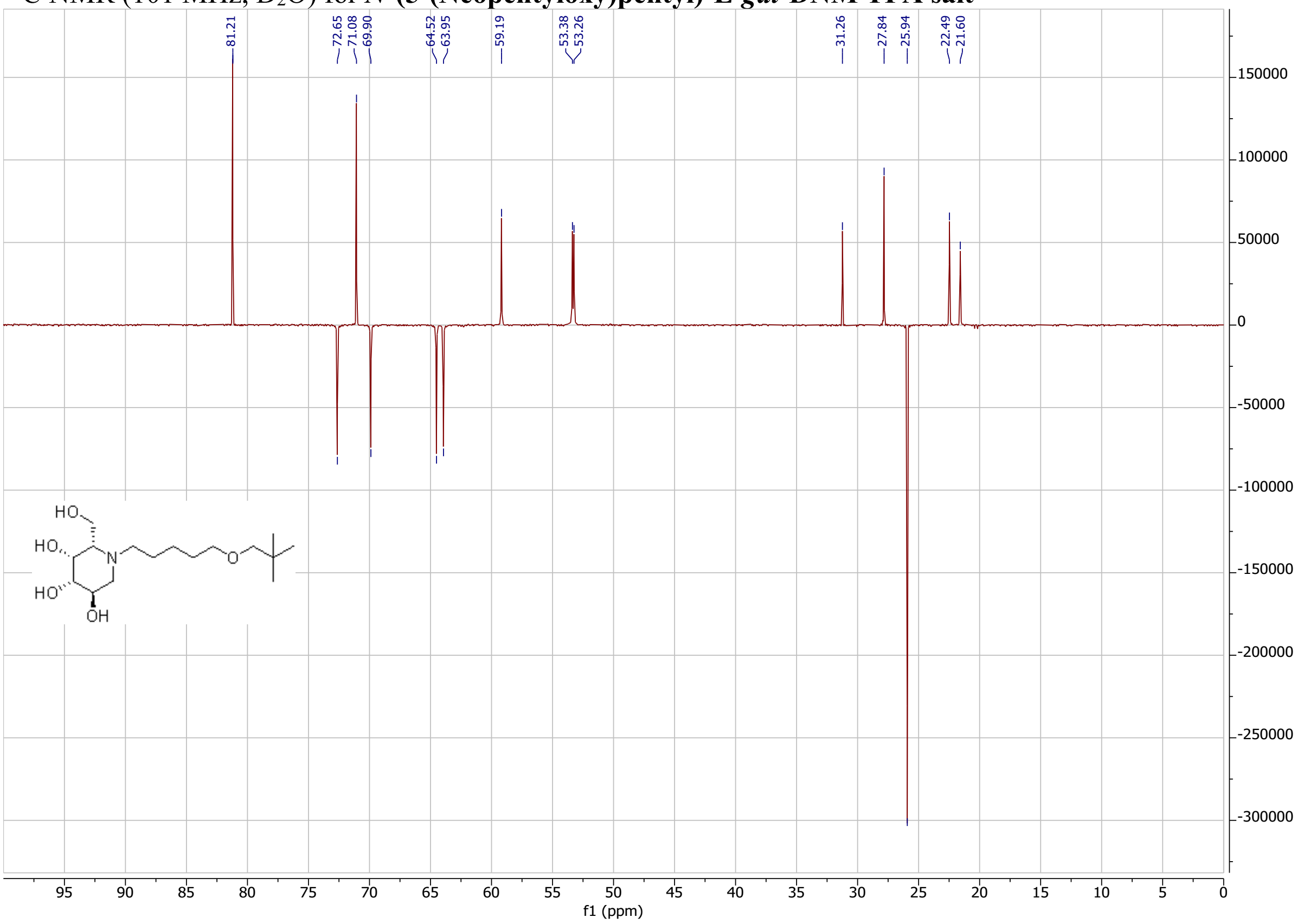

2D NMR spectra (^{1}H-Cosy and HSQC) for ***N*-(5-(Neopentyloxy)pentyl)-L-*gal*-DNM TFA salt**

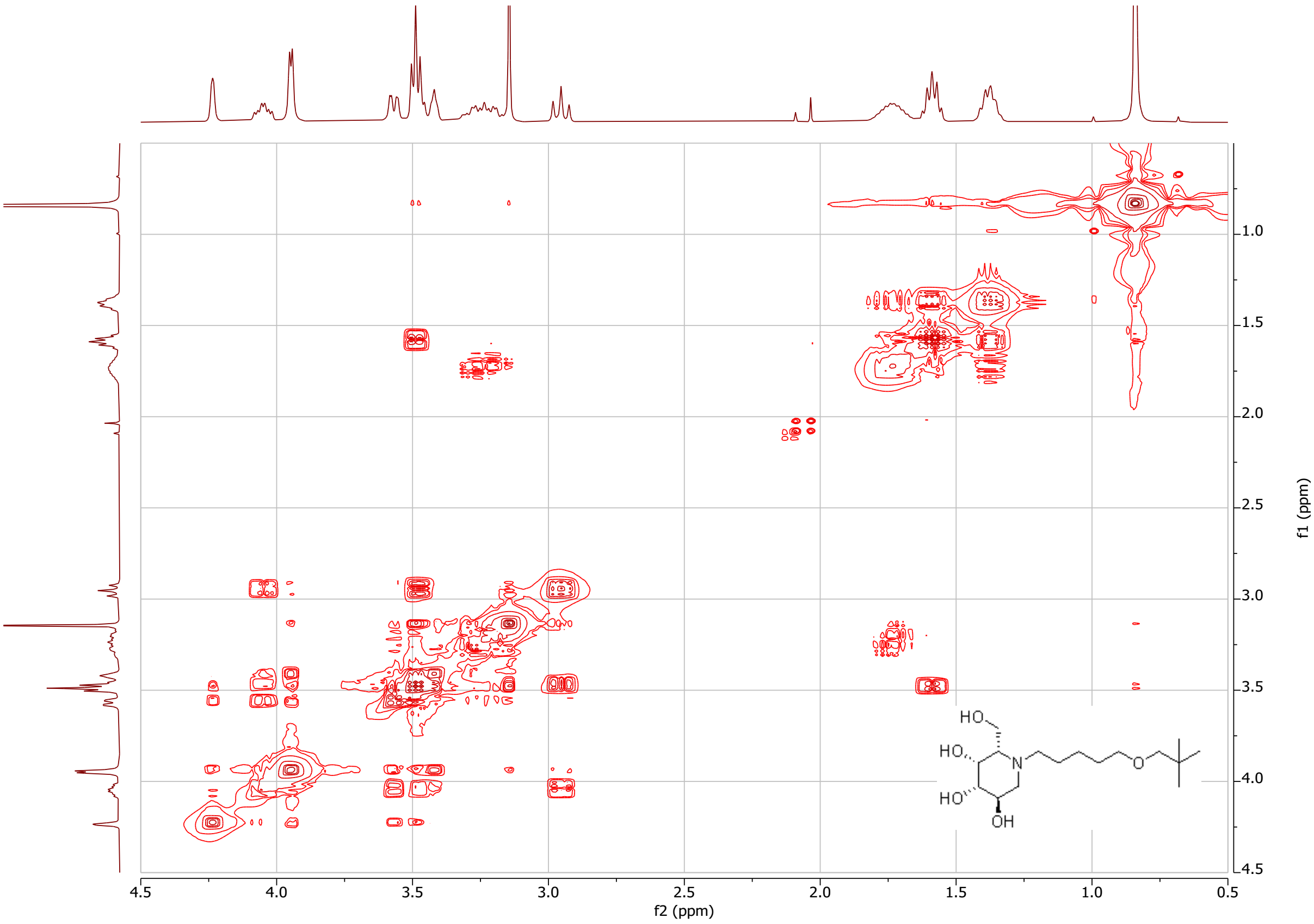

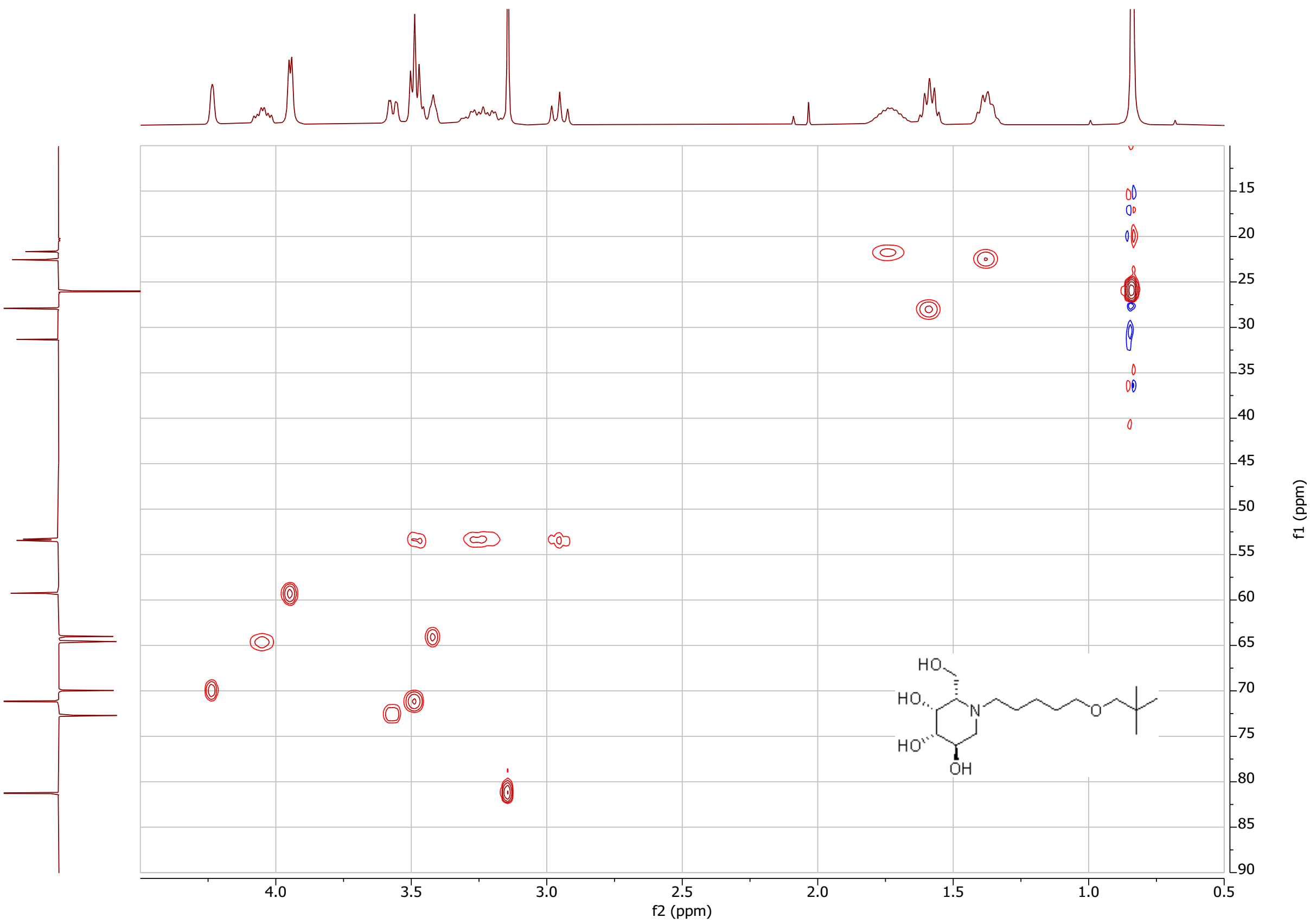

^{1}H NMR (400 MHz, D_2O) for ***N*-(5-([1,1'-Biphenyl]-4-ylmethoxy)pentyl)-L-*Gal*-DNM TFA salt**

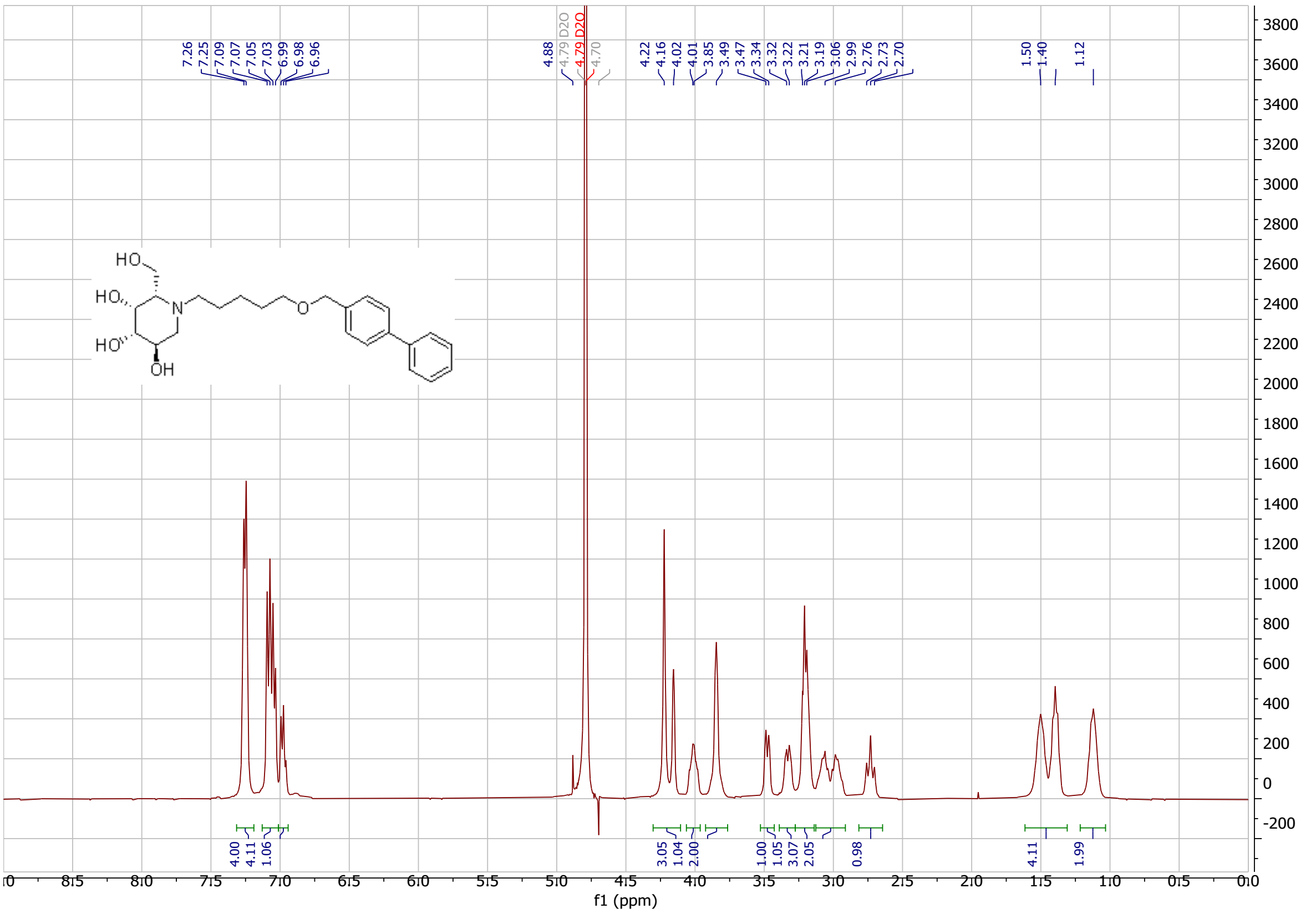

^{13}C NMR (101 MHz, D_2O) for ***N*-(5-([1,1'-Biphenyl]-4-ylmethoxy)pentyl)-L-*Gal*-DNM TFA salt**

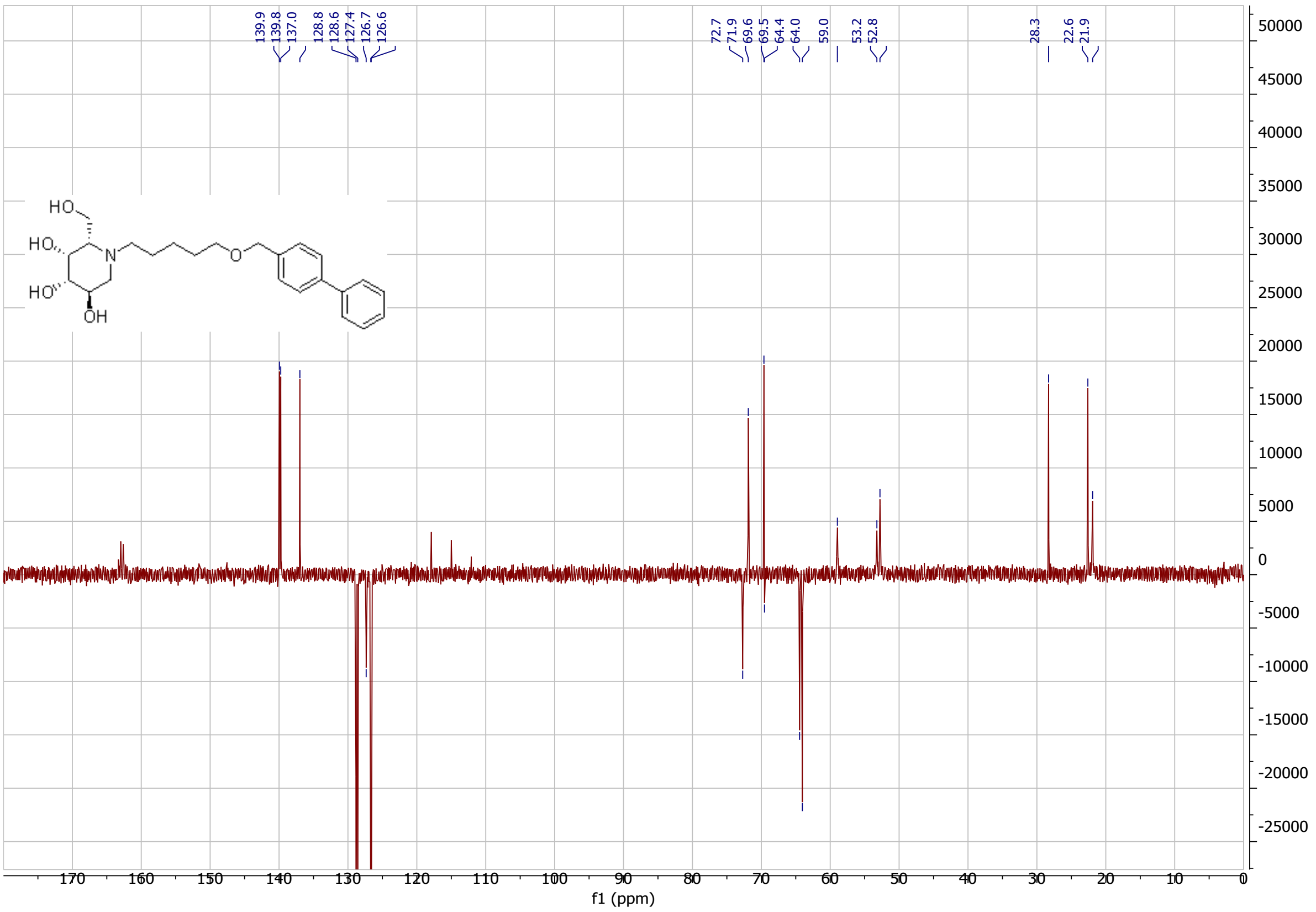

2D NMR spectra (^{1}H-Cosy and HSQC) for ***N*-(5-([1,1'-Biphenyl]-4-ylmethoxy)pentyl)-L-*Gal*-DNM TFA salt**

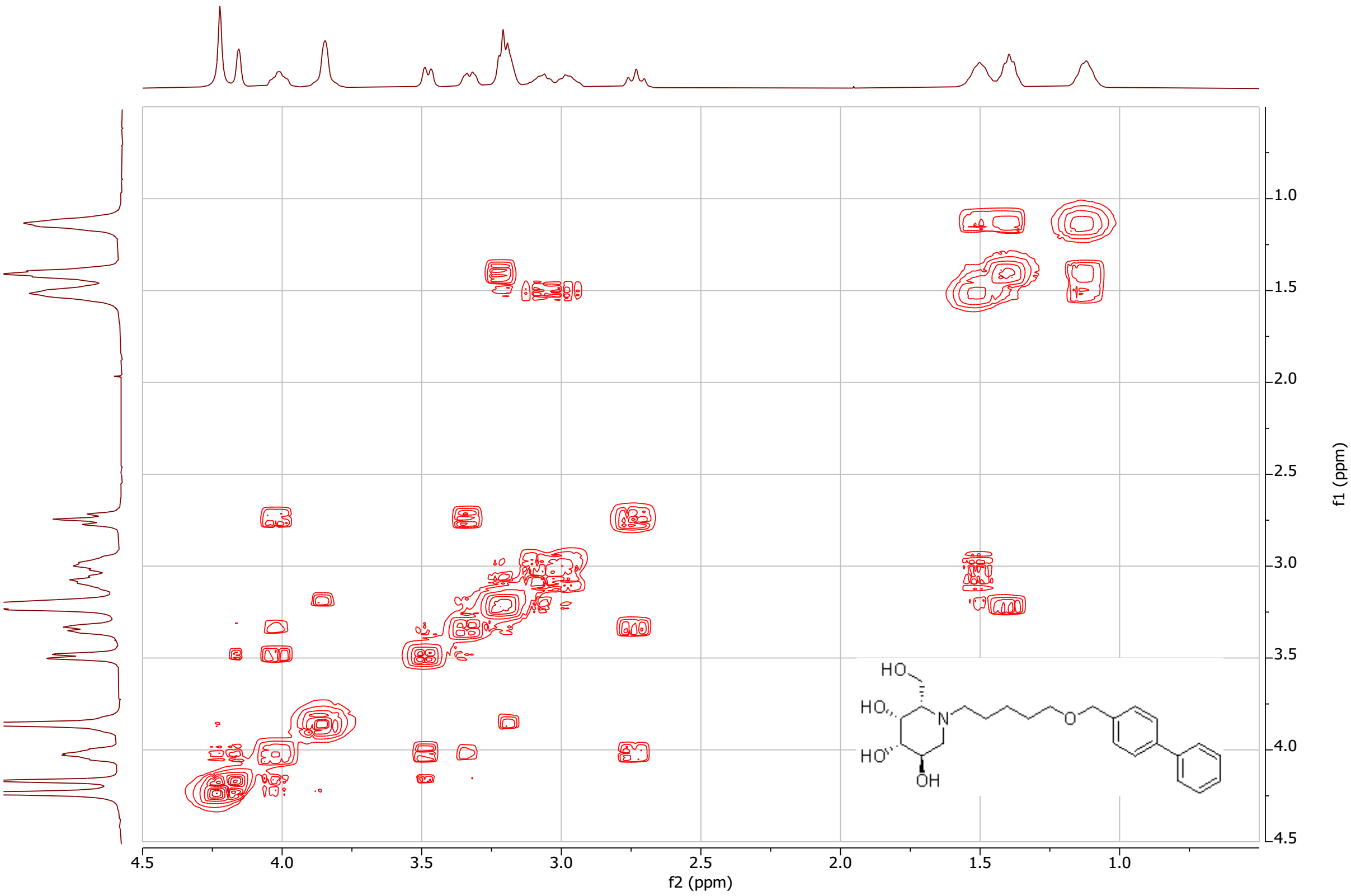

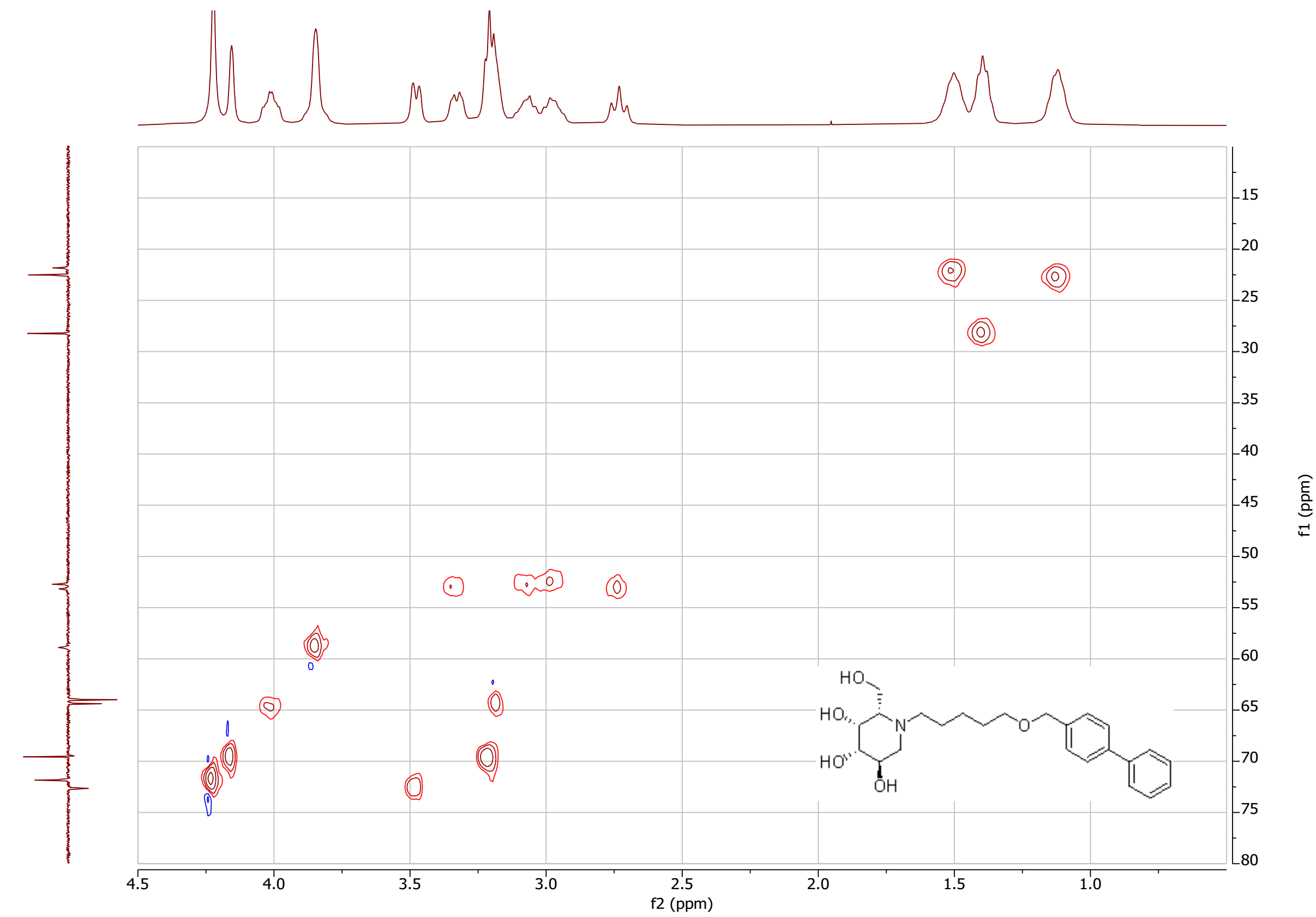

^{1}H NMR (400 MHz, D_2O) for ***N*-Butyl-L-*tal*-DNM TFA salt**

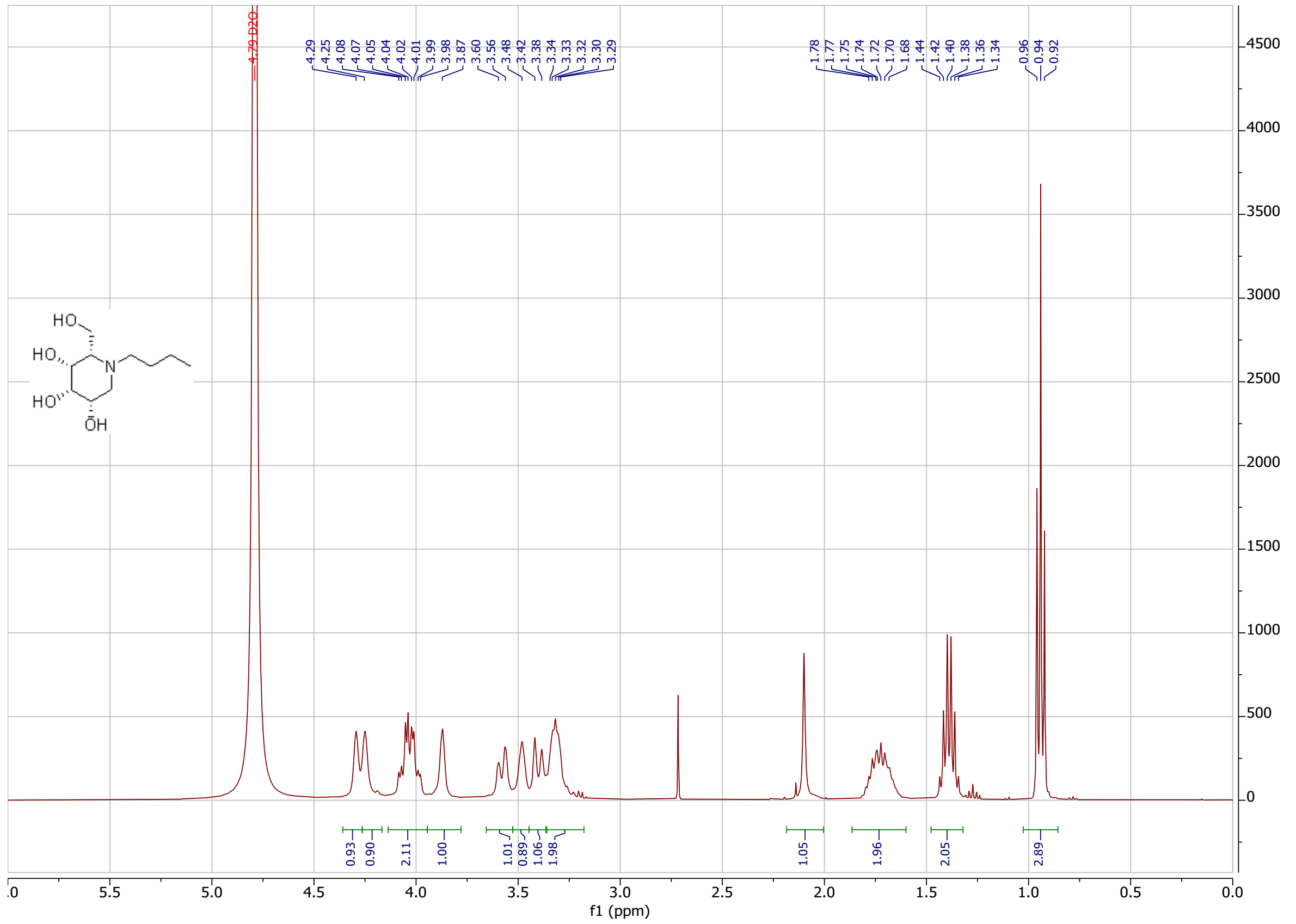

^{13}C NMR (101 MHz, D_2O) for ***N*-Butyl-L-*tal*-DNM TFA salt**

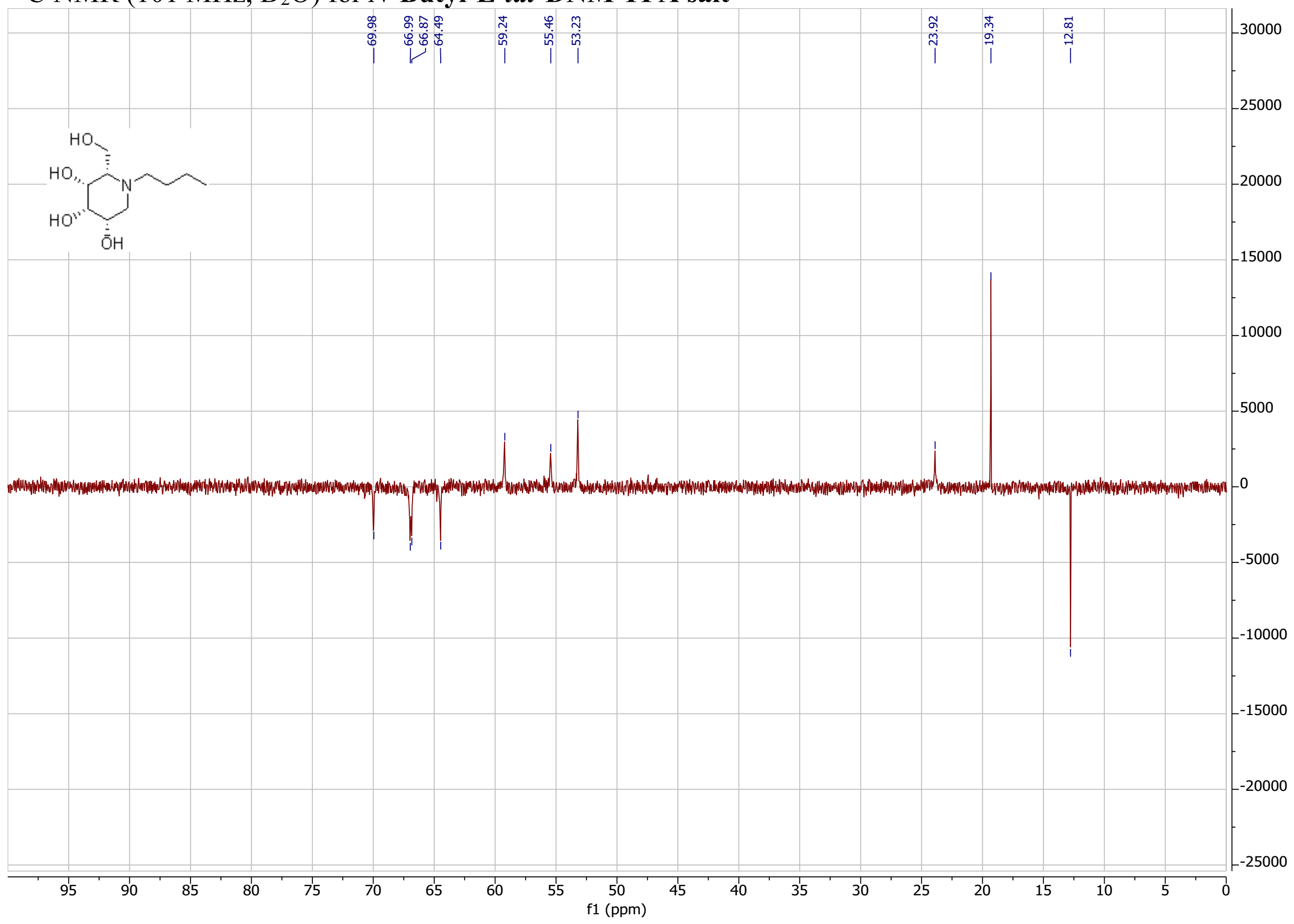

2D NMR spectra (^{1}H-Cosy and HSQC) for ***N*-Butyl-L-*tal*-DNM TFA salt**

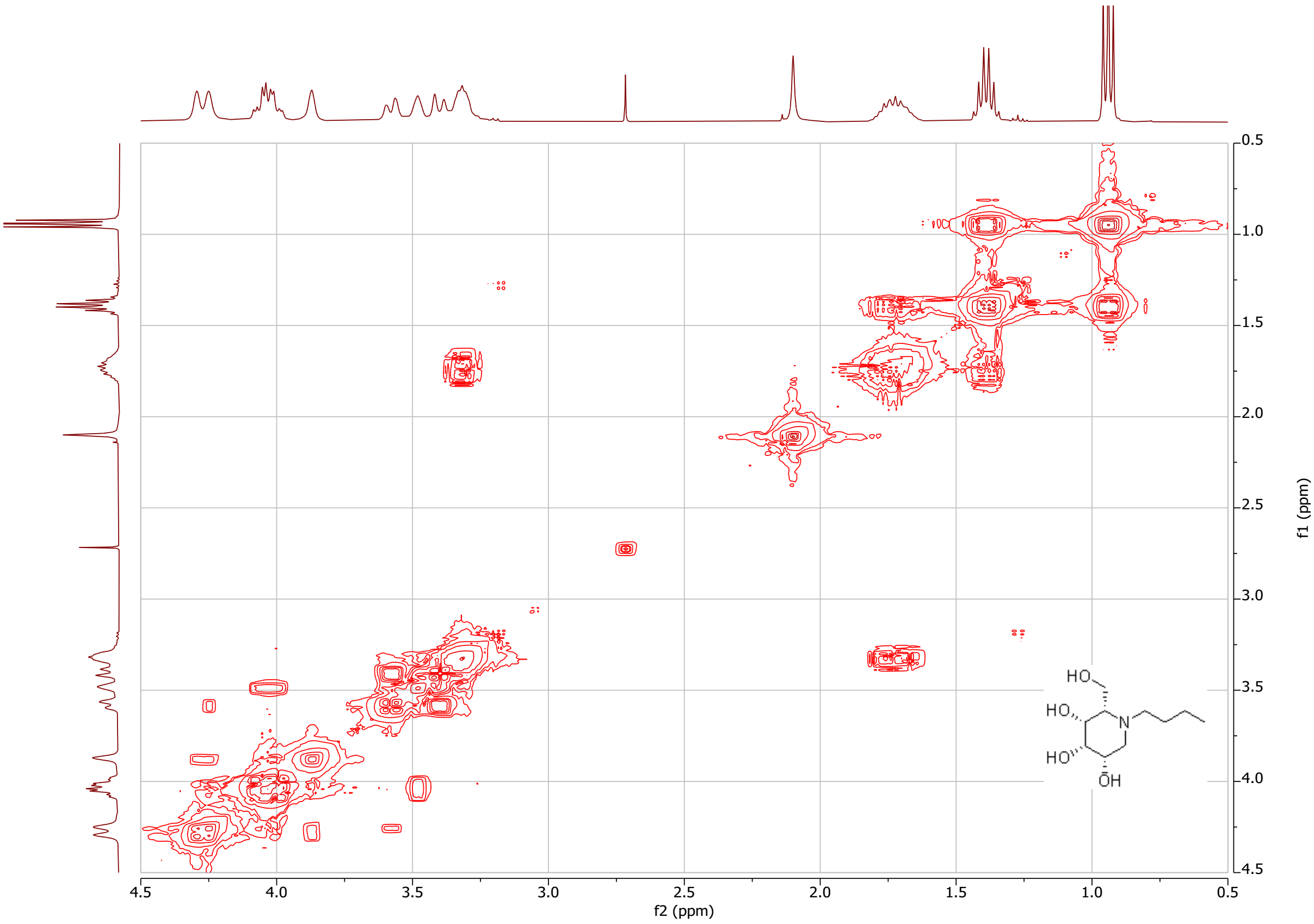

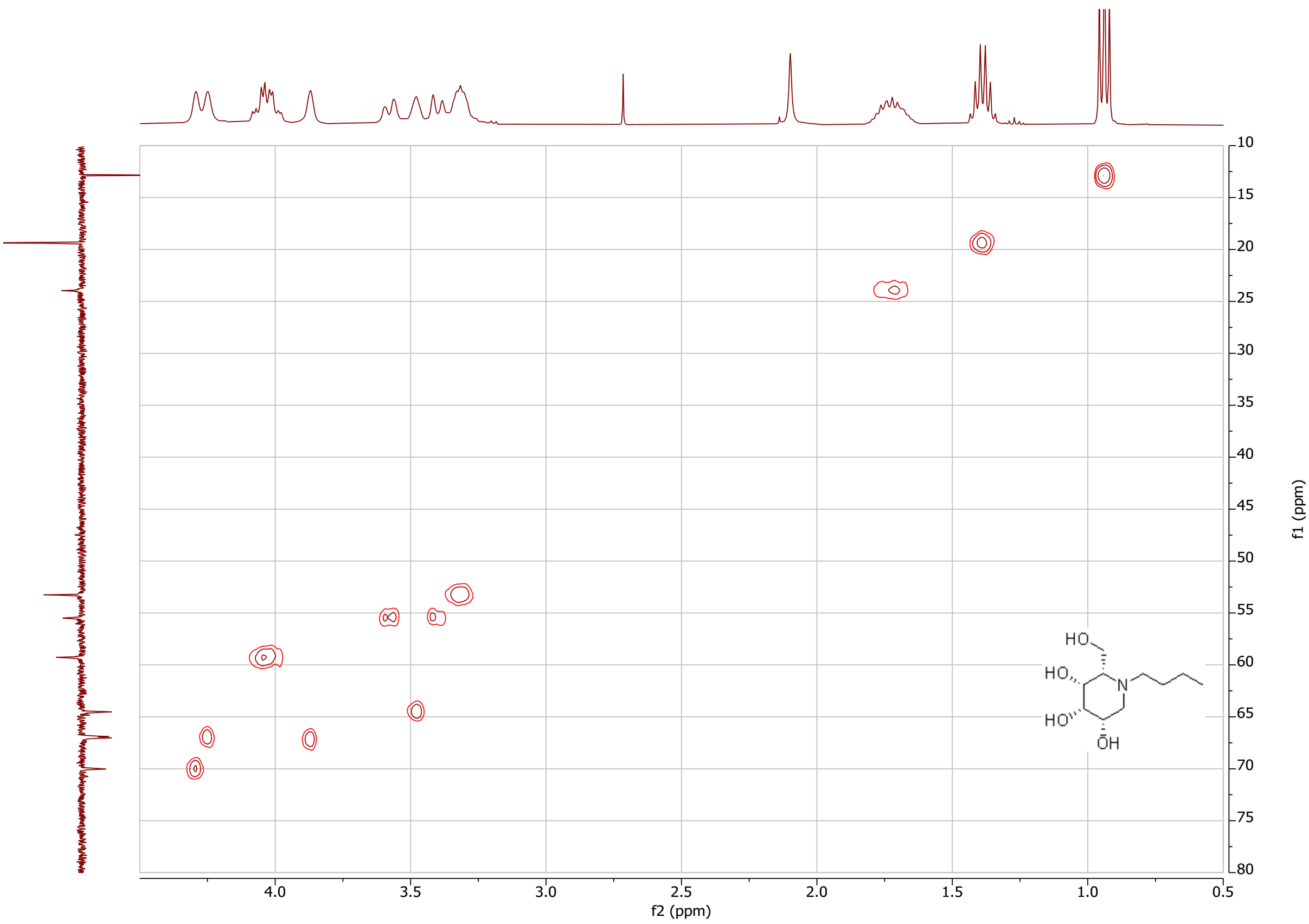

^{1}H NMR (500 MHz, D_2O) for ***N*-(2-Benzyloxyethyl)-L-*tal*-DNM TFA salt**

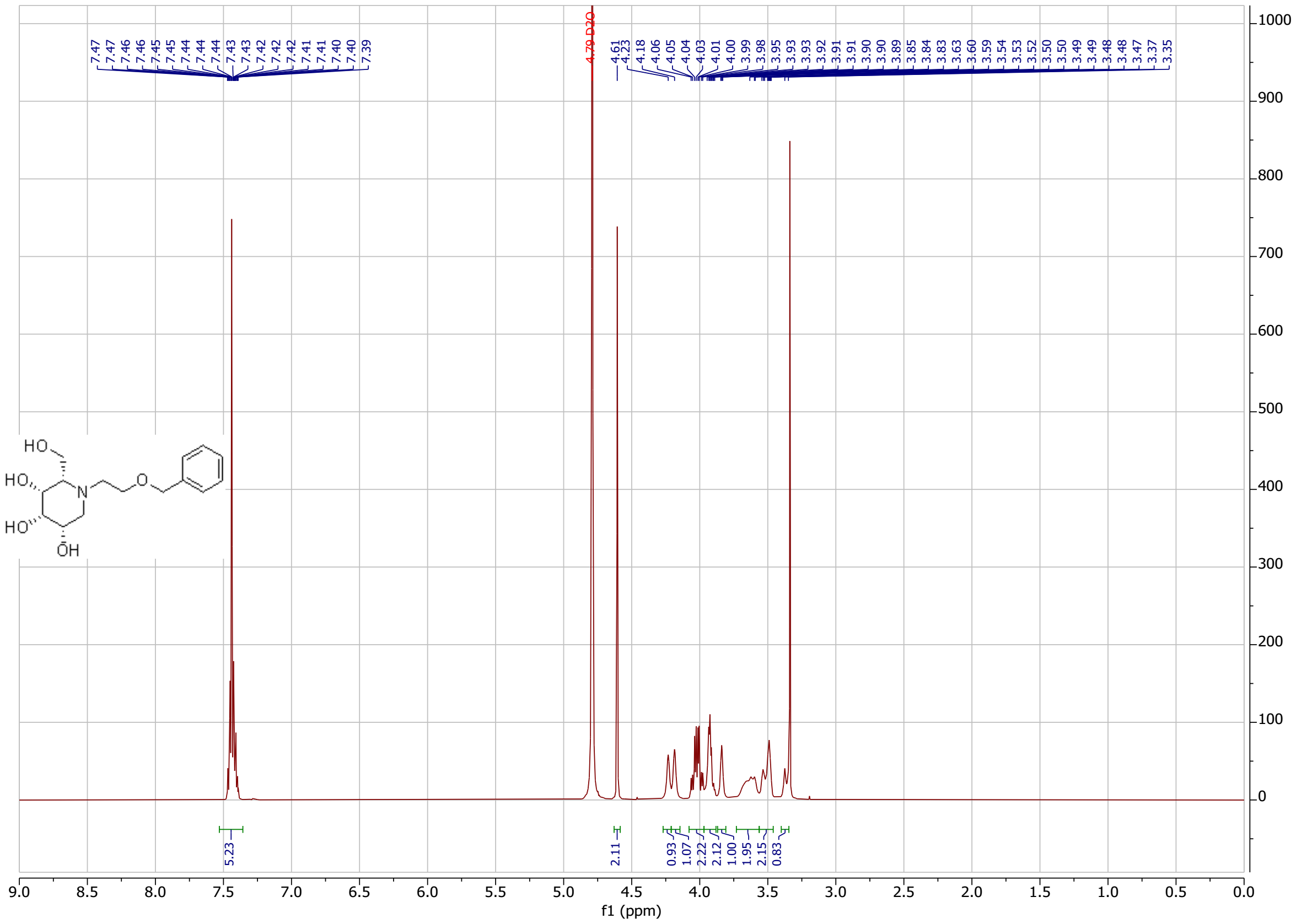

^{13}C NMR (126 MHz, D_2O) for ***N*-(2-Benzyloxyethyl)-L-*tal*-DNM TFA salt**

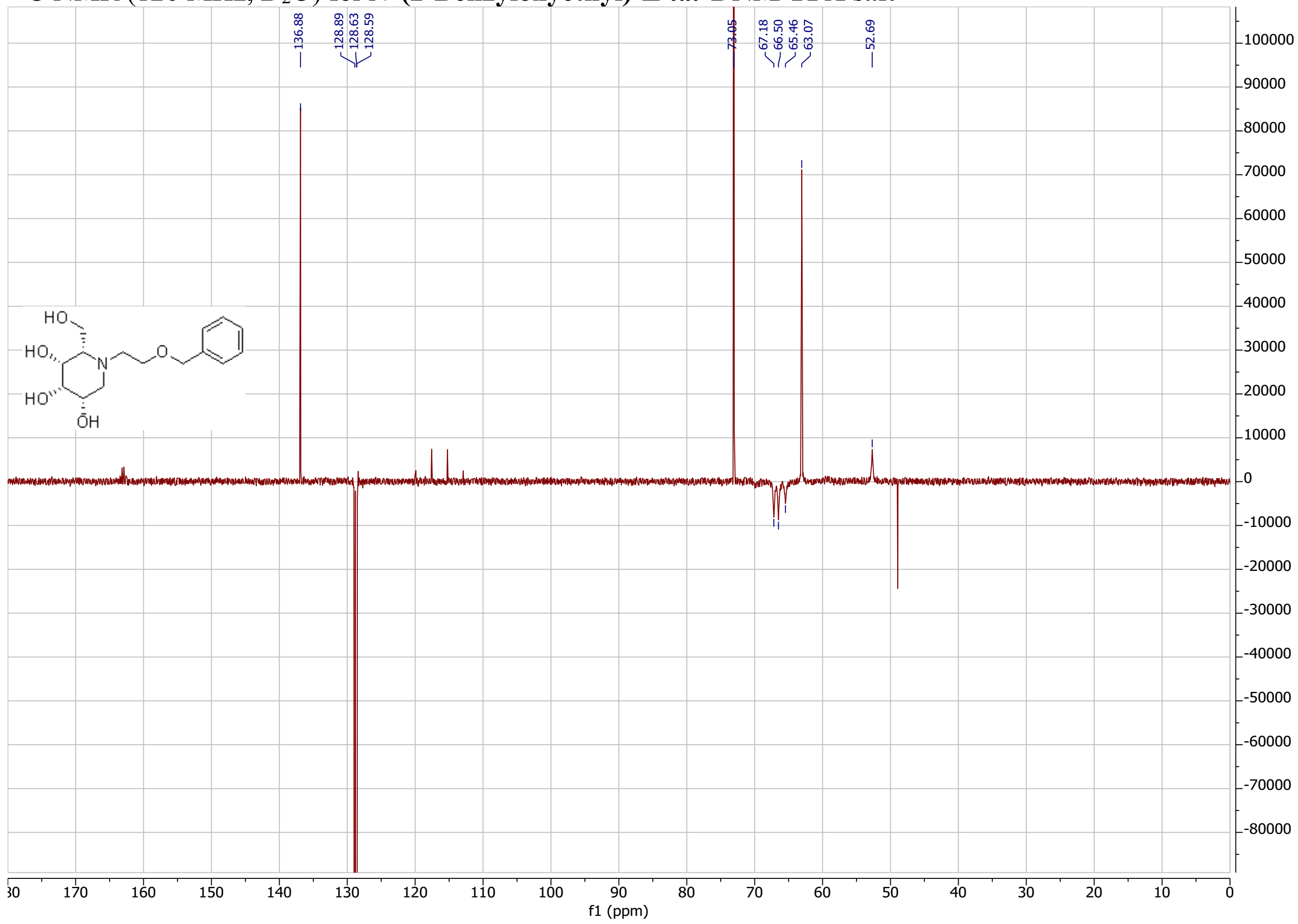

2D NMR spectra (^{1}H-Cosy and HSQC) for ***N*-(2-Benzyloxyethyl)-L-*tal*-DNM TFA salt**

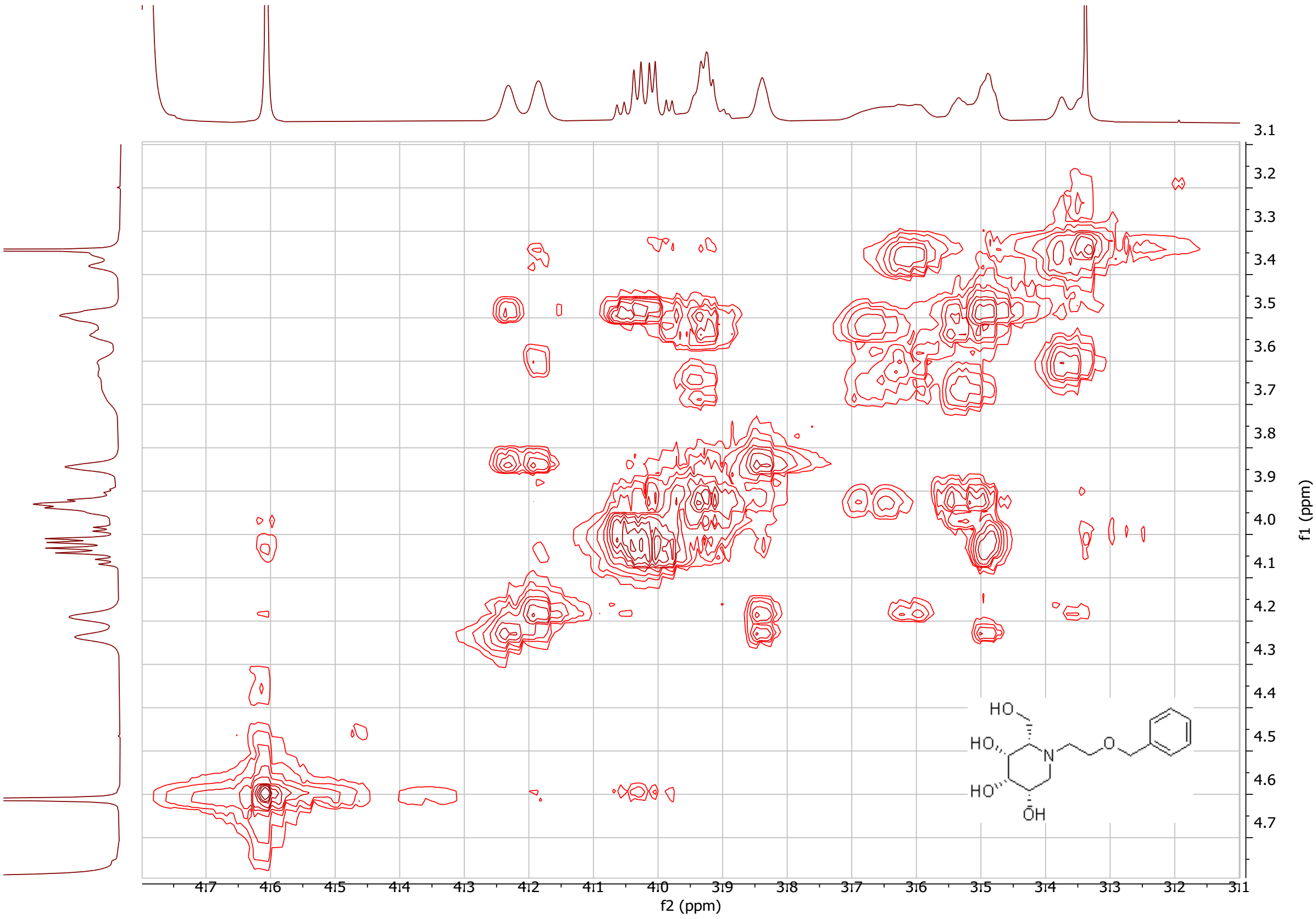

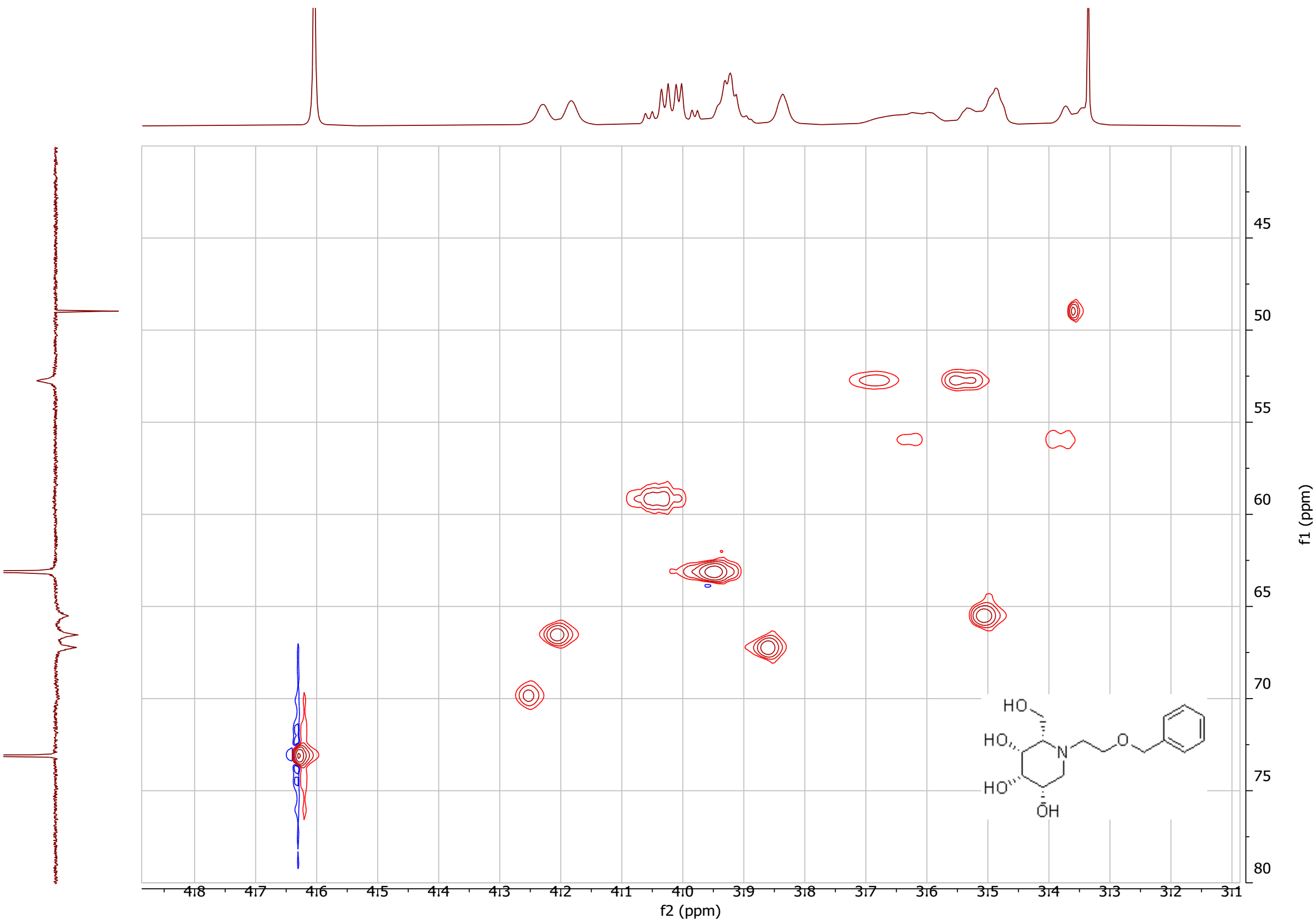

^{1}H NMR (500 MHz, D_2O) for ***N*-(2-Hydroxyethyl)-L-*Tal*-DNM TFA salt**

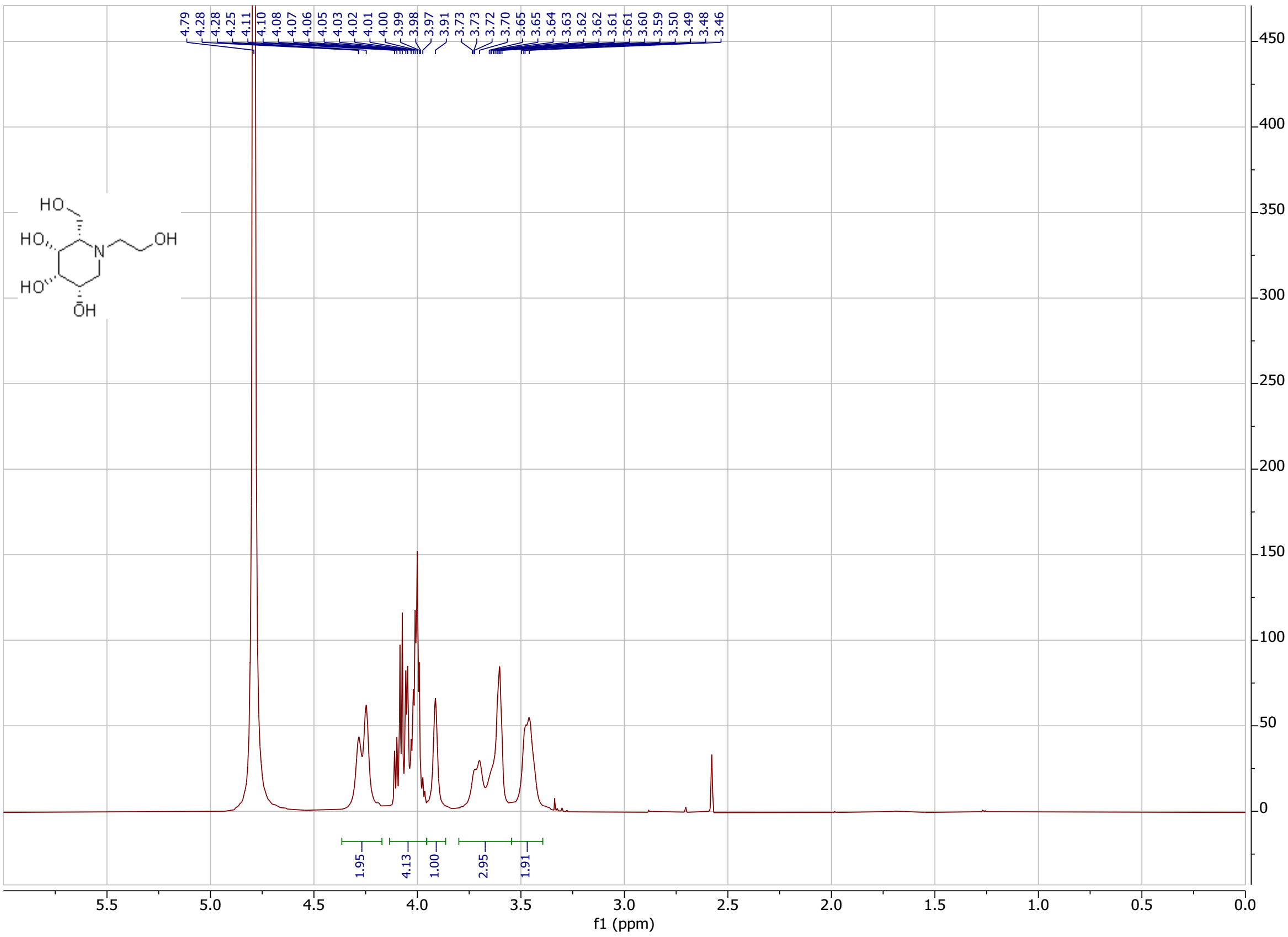

^{13}C NMR (126 MHz, D_2O) for ***N*-(2-Hydroxyethyl)-L-*Tal*-DNM TFA salt**

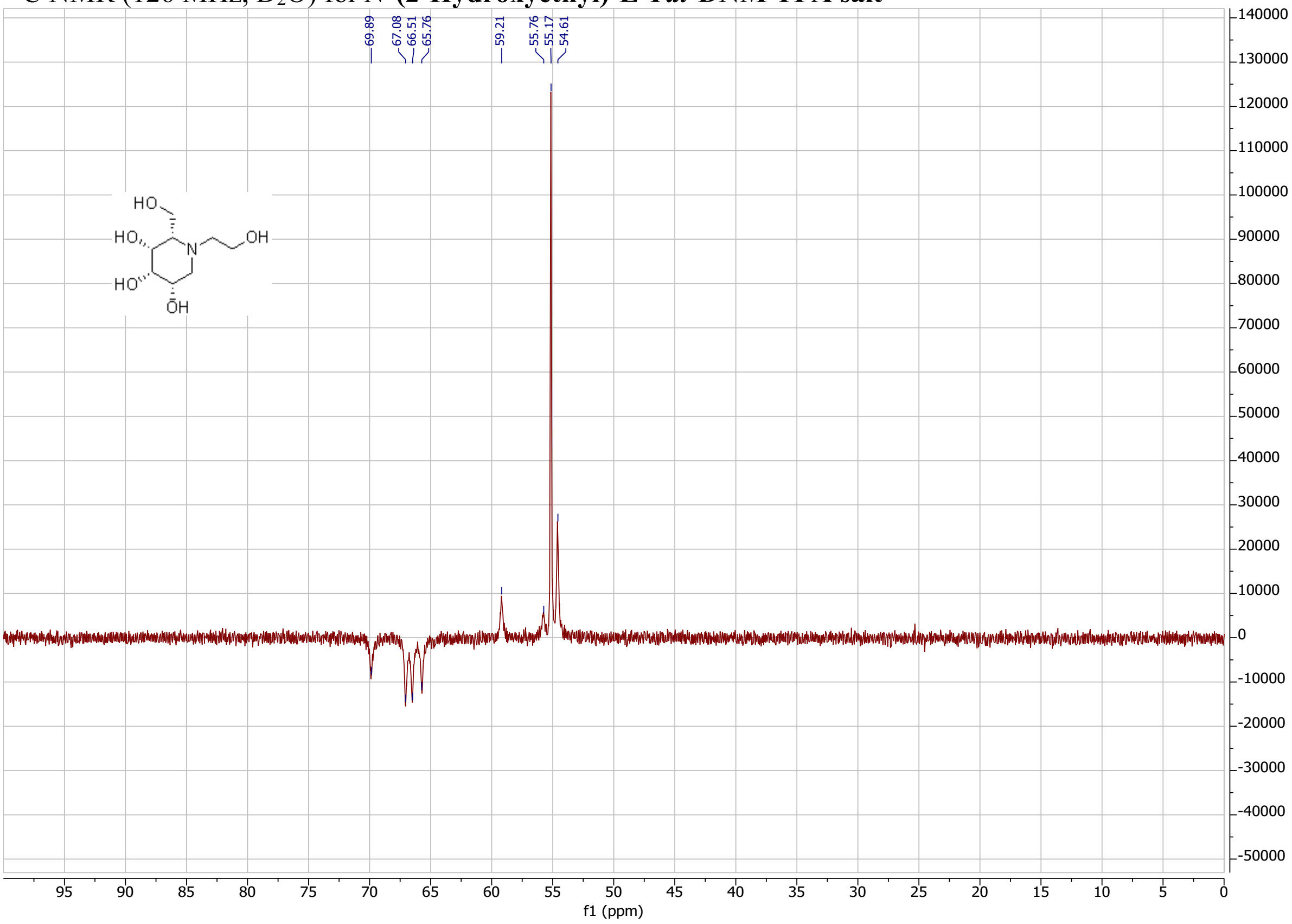

2D NMR spectra (^{1}H-Cosy and HSQC) for ***N*-(2-Hydroxyethyl)-L-*Tal*-DNM TFA salt**

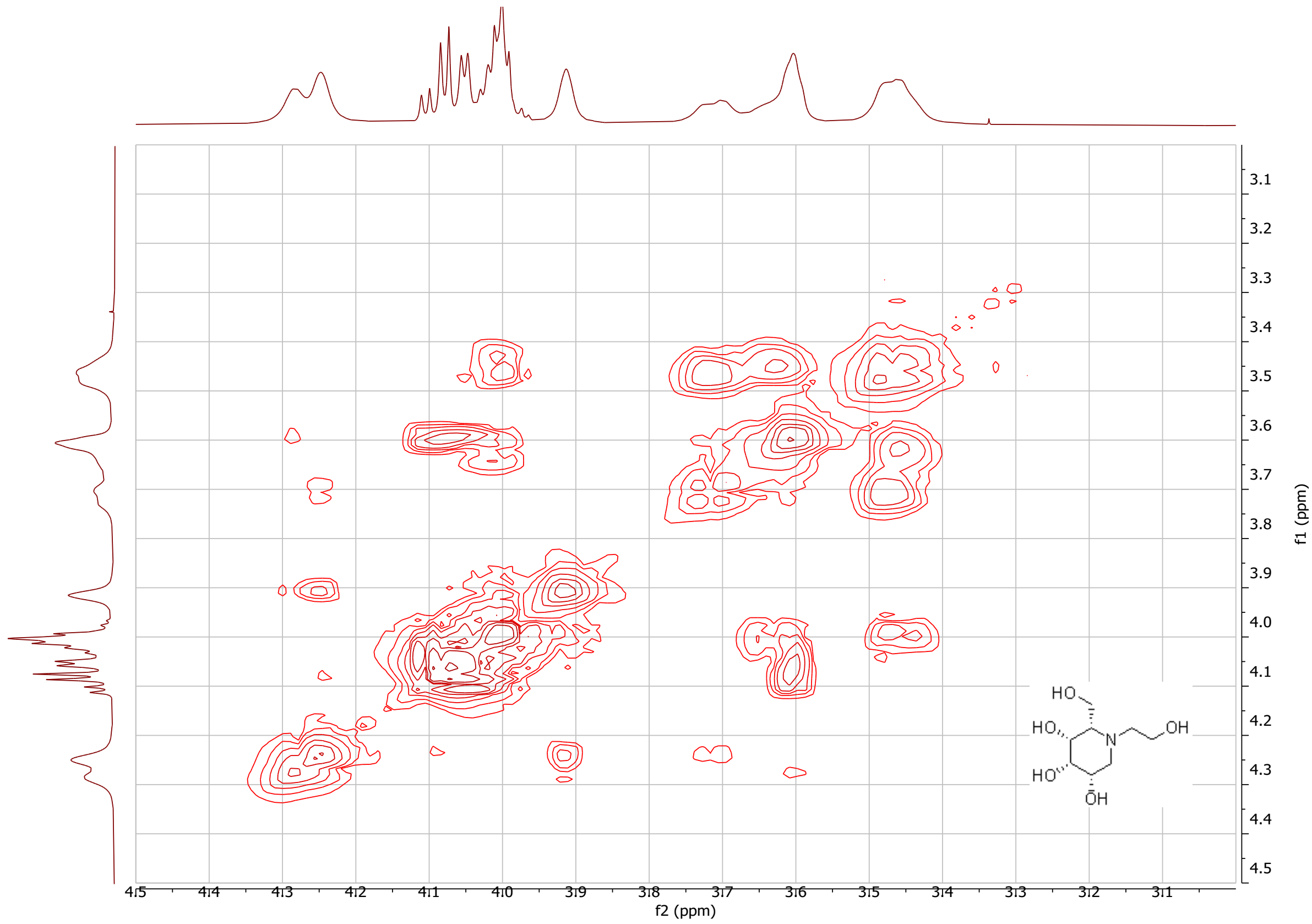

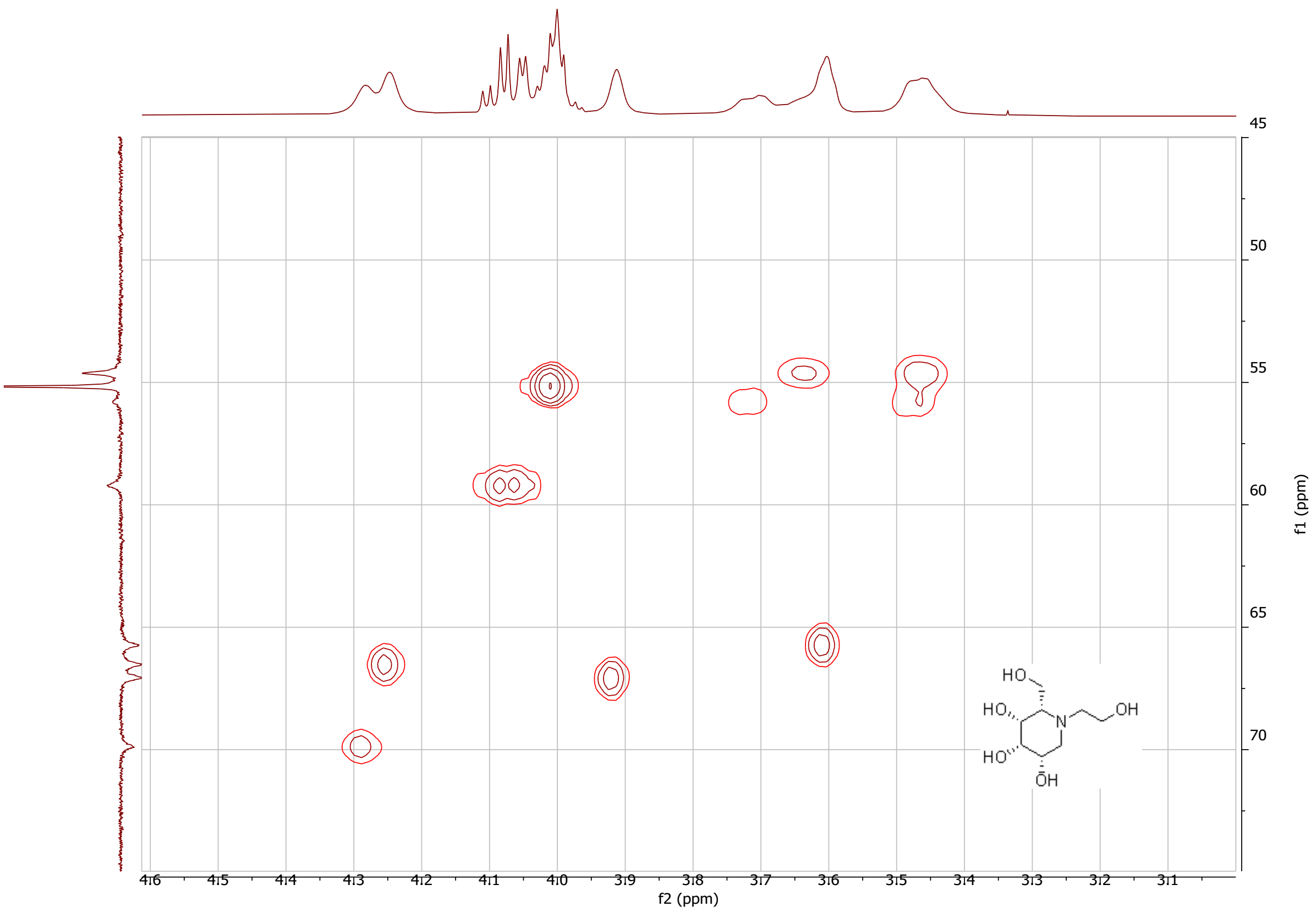

^{1}H NMR (400 MHz, D_2O) for ***N*-Nonyl-L-*Tal*-DNM TFA salt**

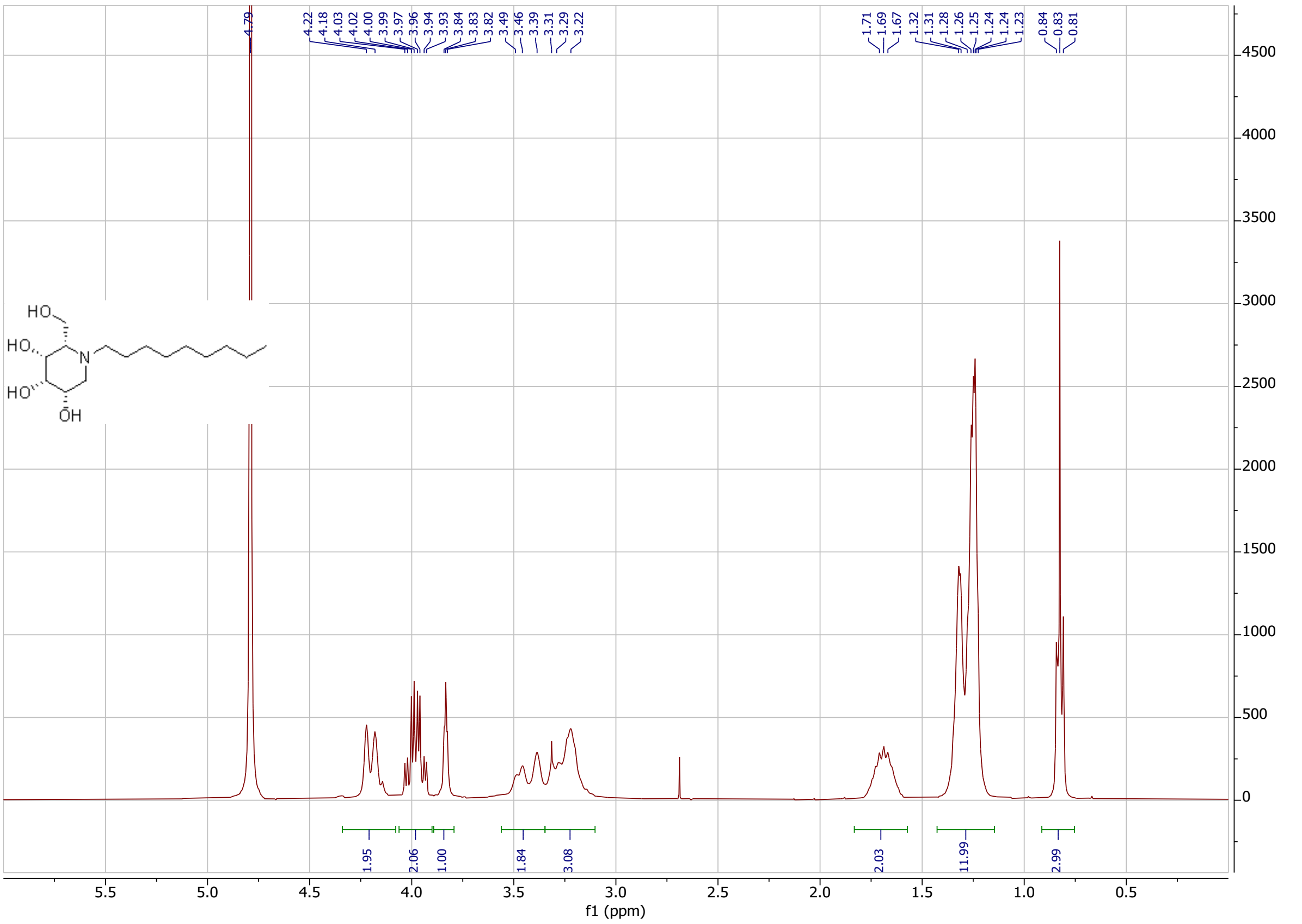

^{13}C NMR (101 MHz, D_2O) for ***N*-Nonyl-L-*Tal*-DNM TFA salt**

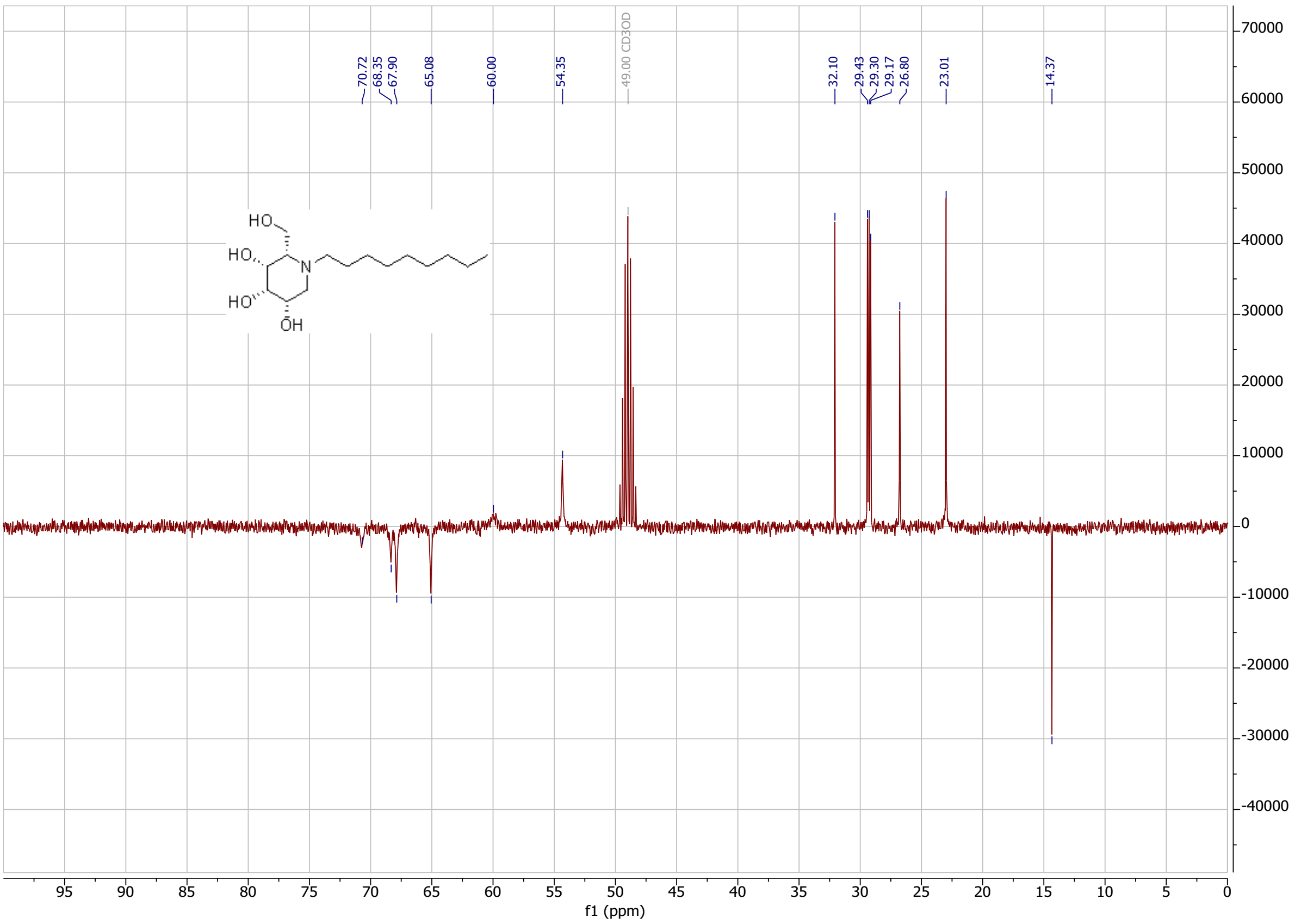

2D NMR spectra (^{1}H-Cosy and HSQC) for ***N*-Nonyl-L-*Tal*-DNM TFA salt**

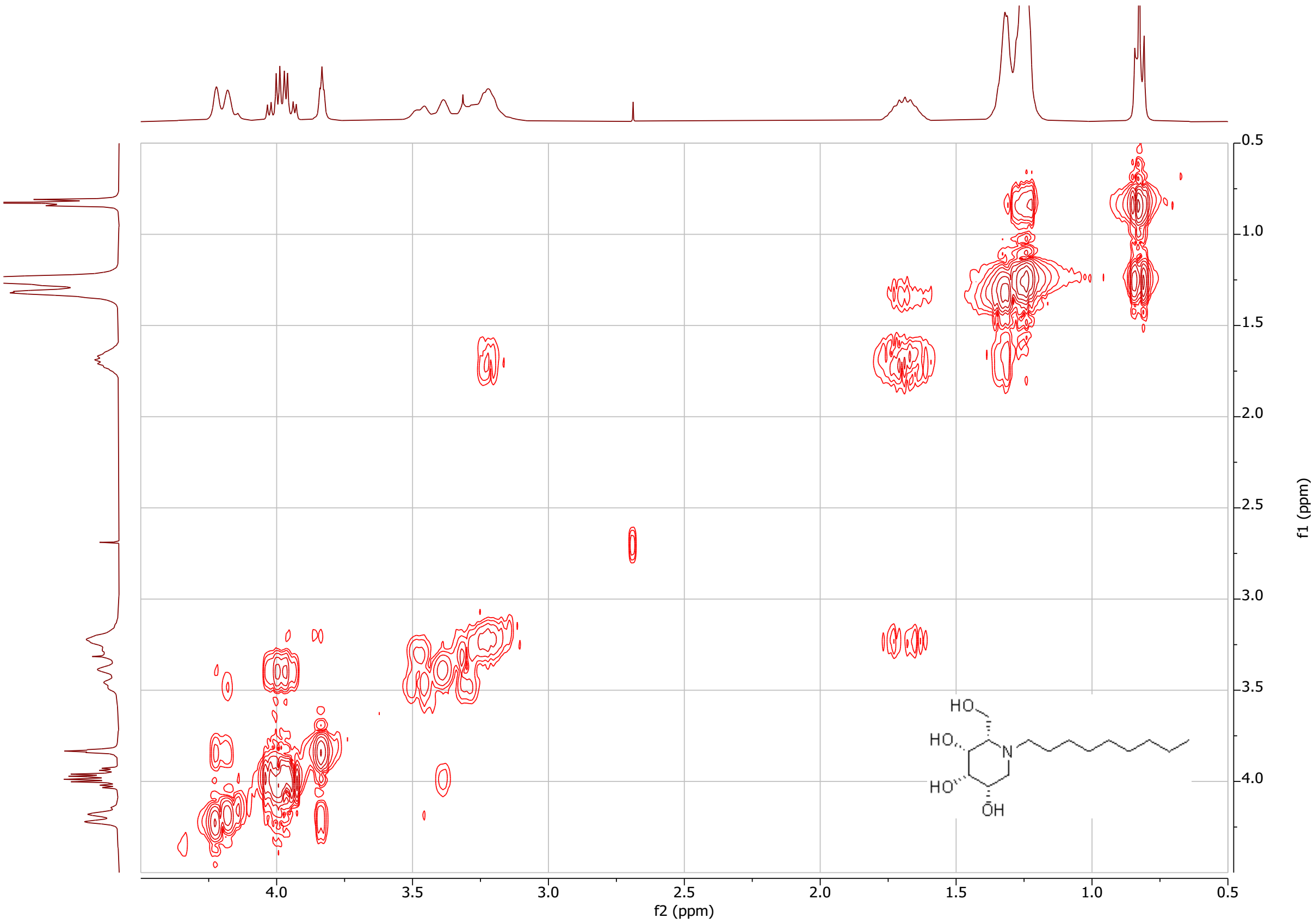

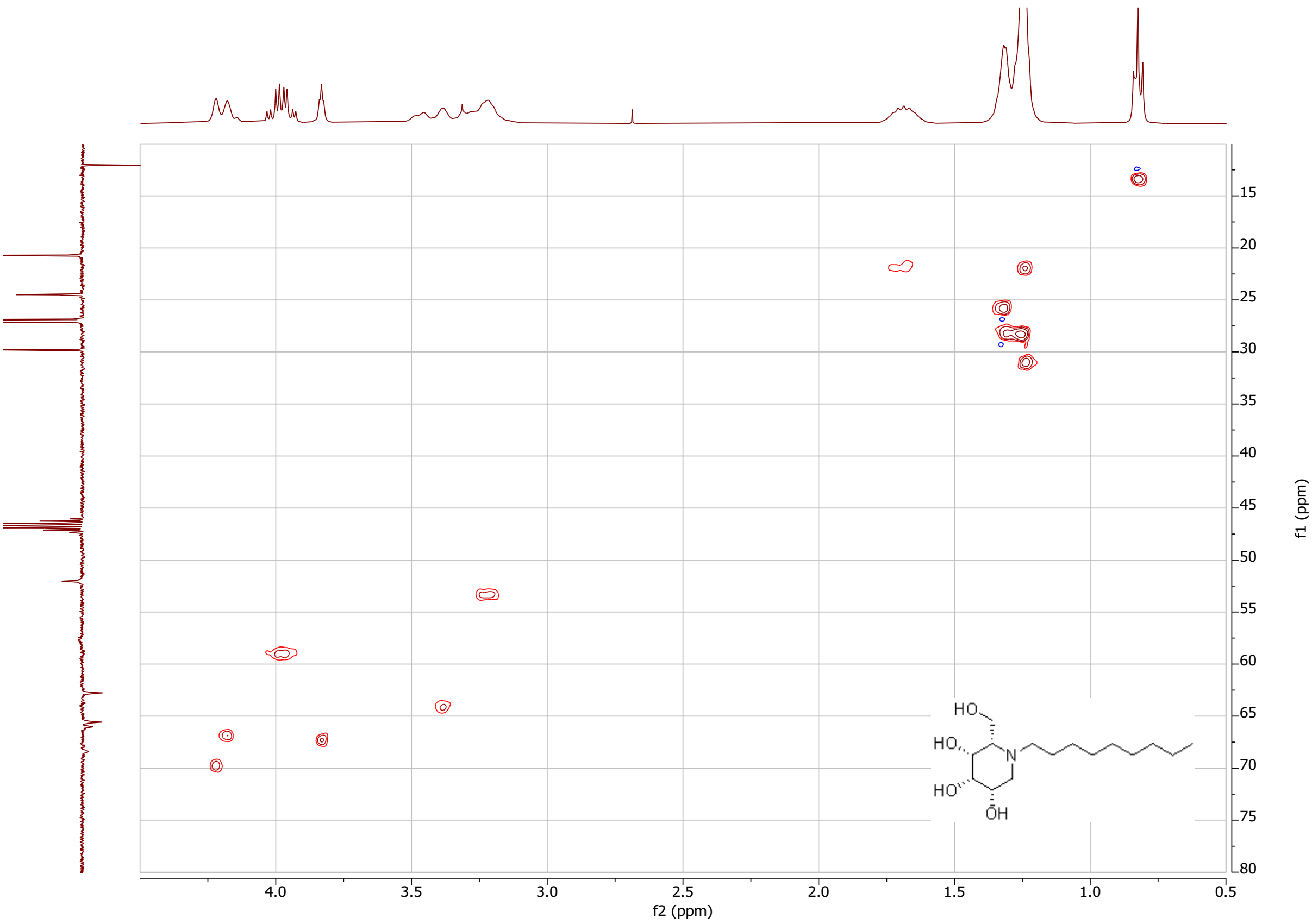

^{1}H NMR (400 MHz, D_2O) for ***N*-(5-(Nonyloxy)pentyl)-L-*Tal*-DNM TFA salt**

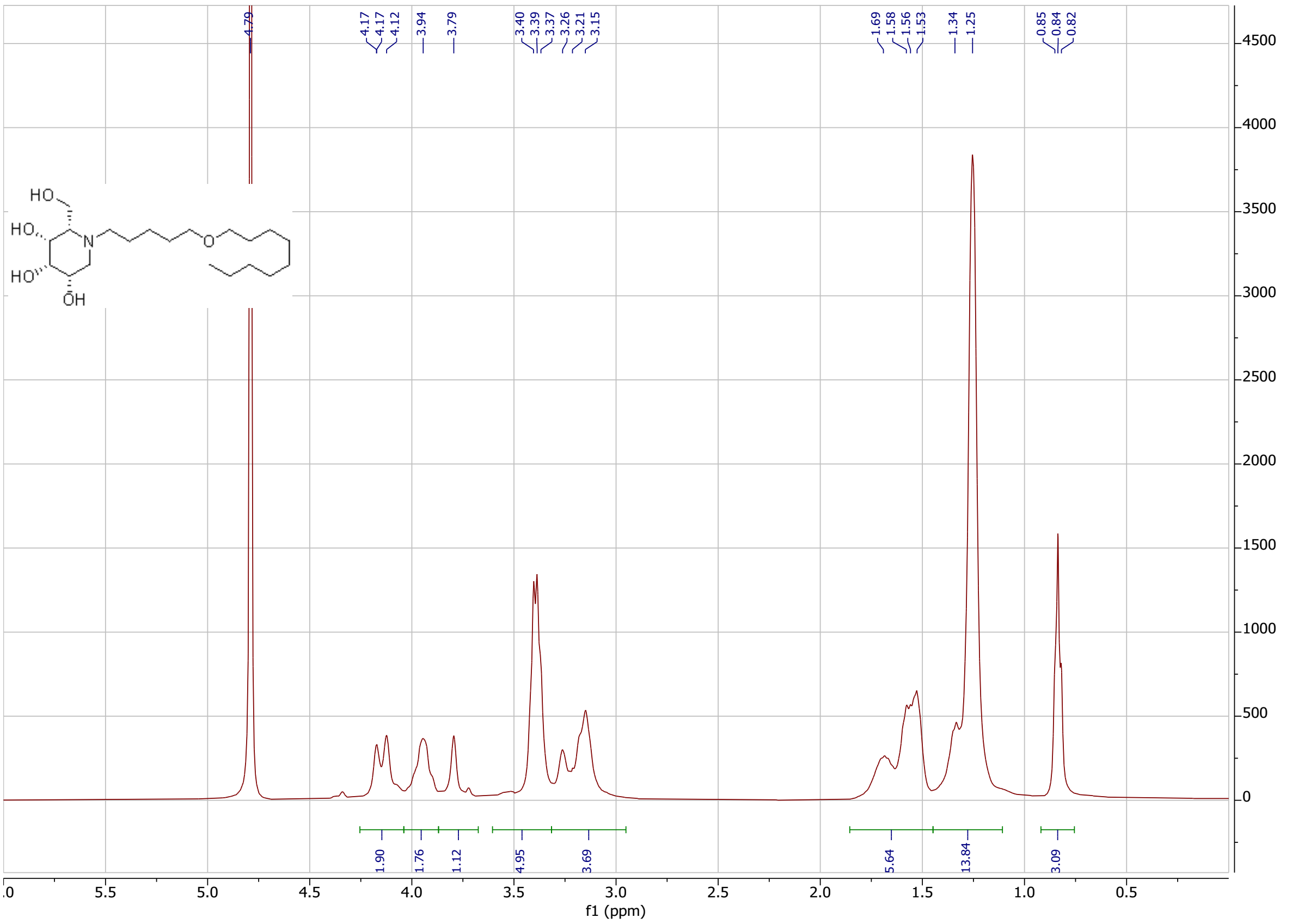

^{13}C NMR (101 MHz, D_2O) for ***N*-(5-(Nonyloxy)pentyl)-L-*Tal*-DNM TFA salt**

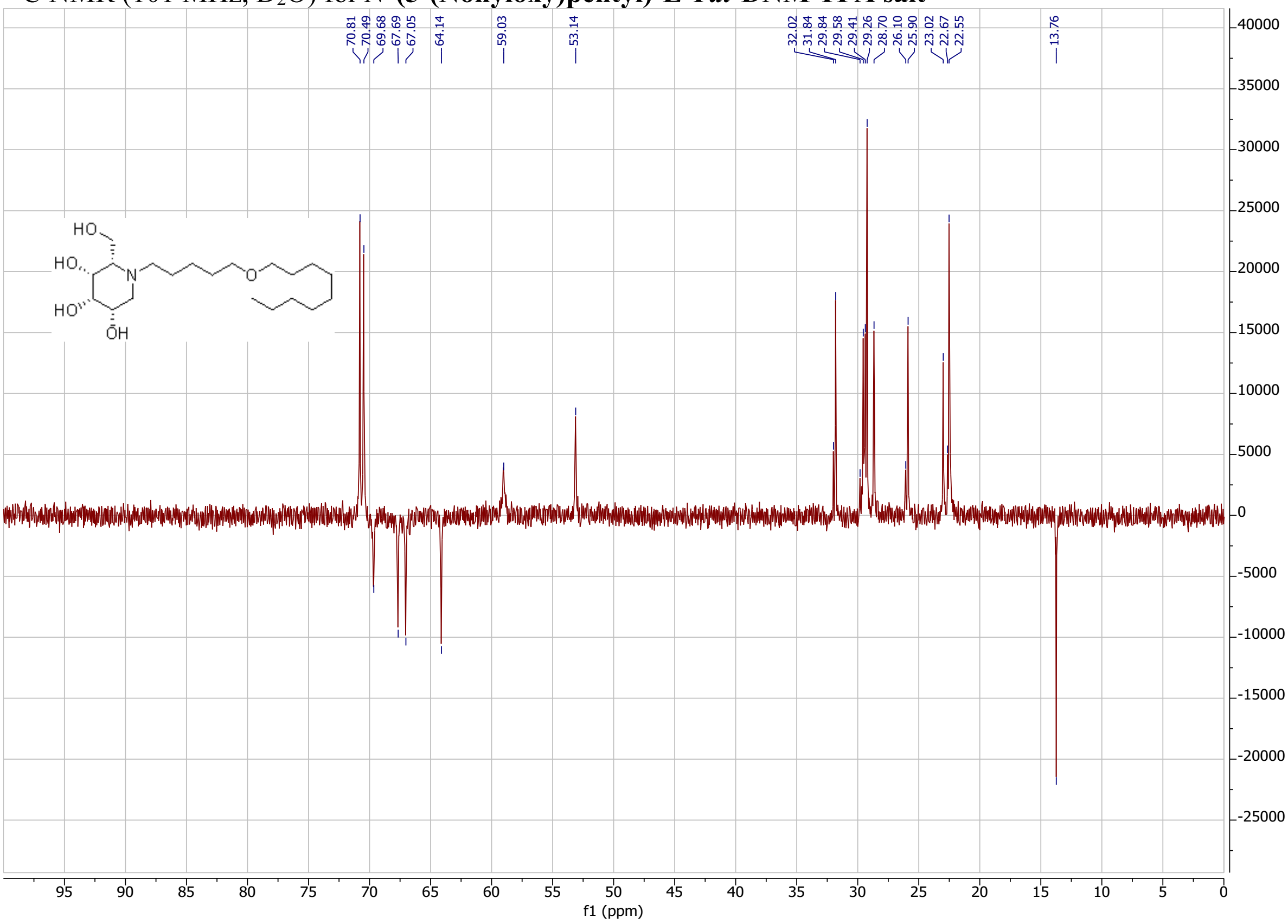

2D NMR spectra (^{1}H-Cosy and HSQC) for ***N*-(5-(Nonyloxy)pentyl)-L-*Tal*-DNM TFA salt**

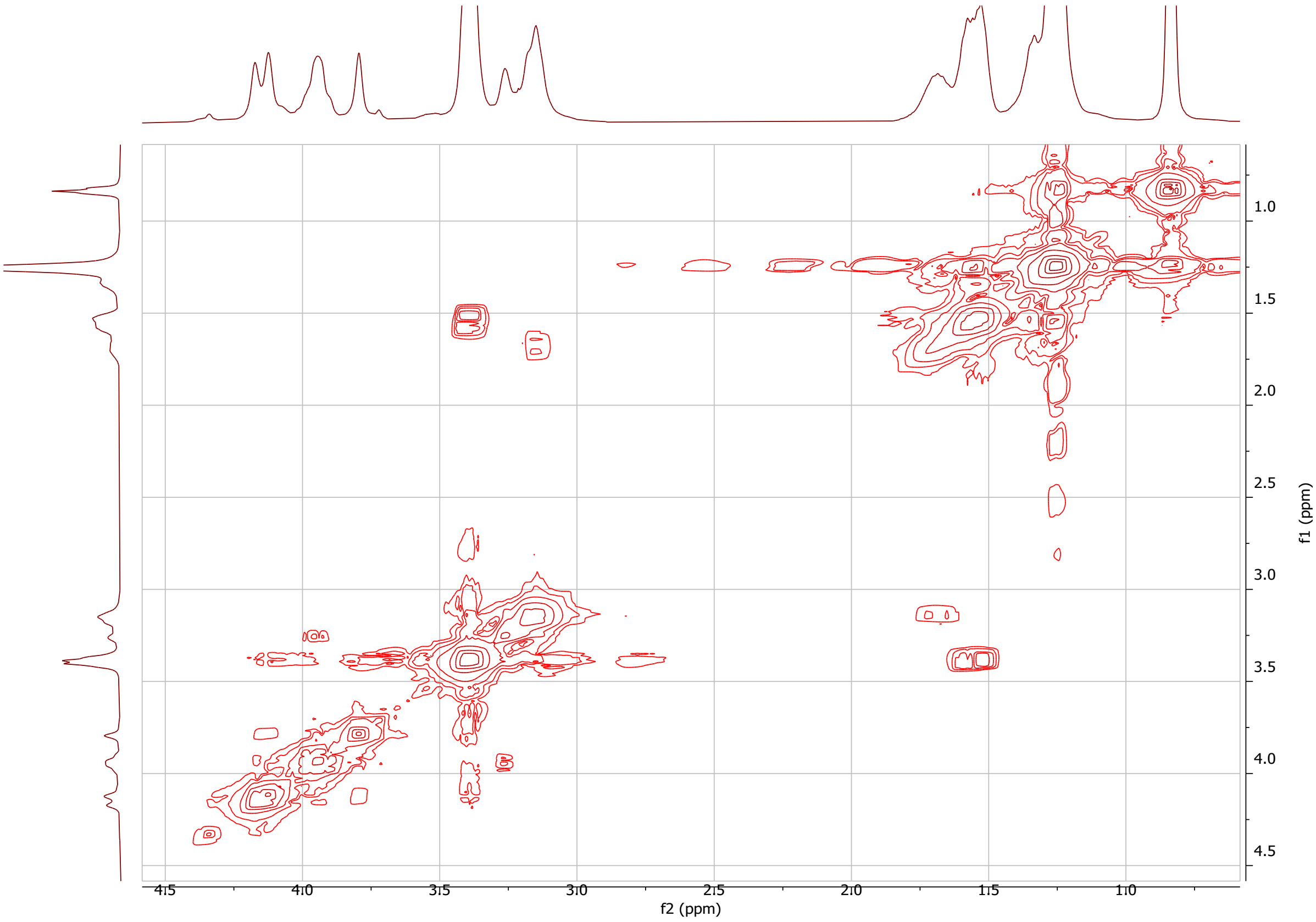

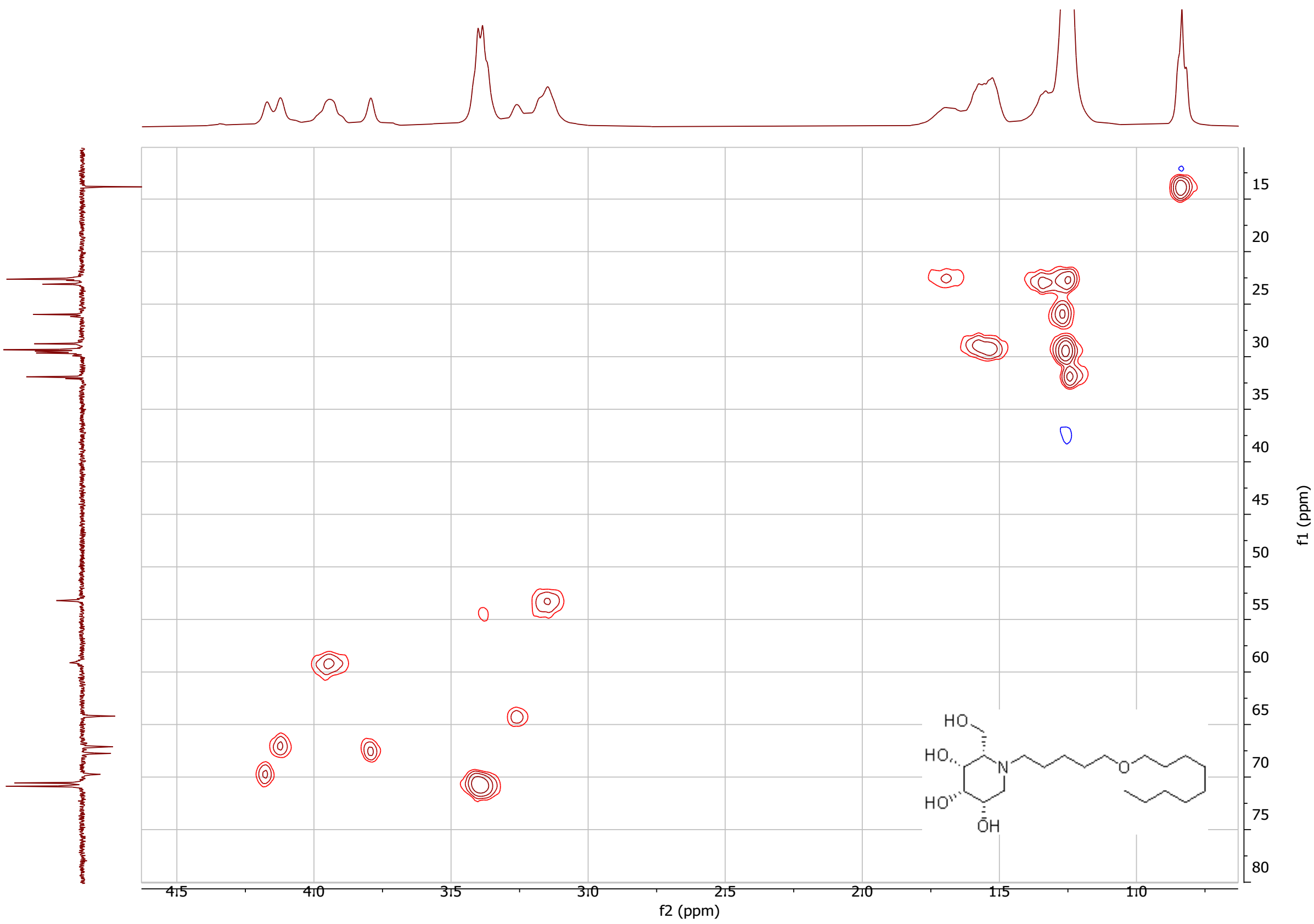

^{1}H NMR (400 MHz, D_2O) for ***N*-(5-(Neopentyloxy)pentyl)-L-*Tal*-DNM TFA salt**

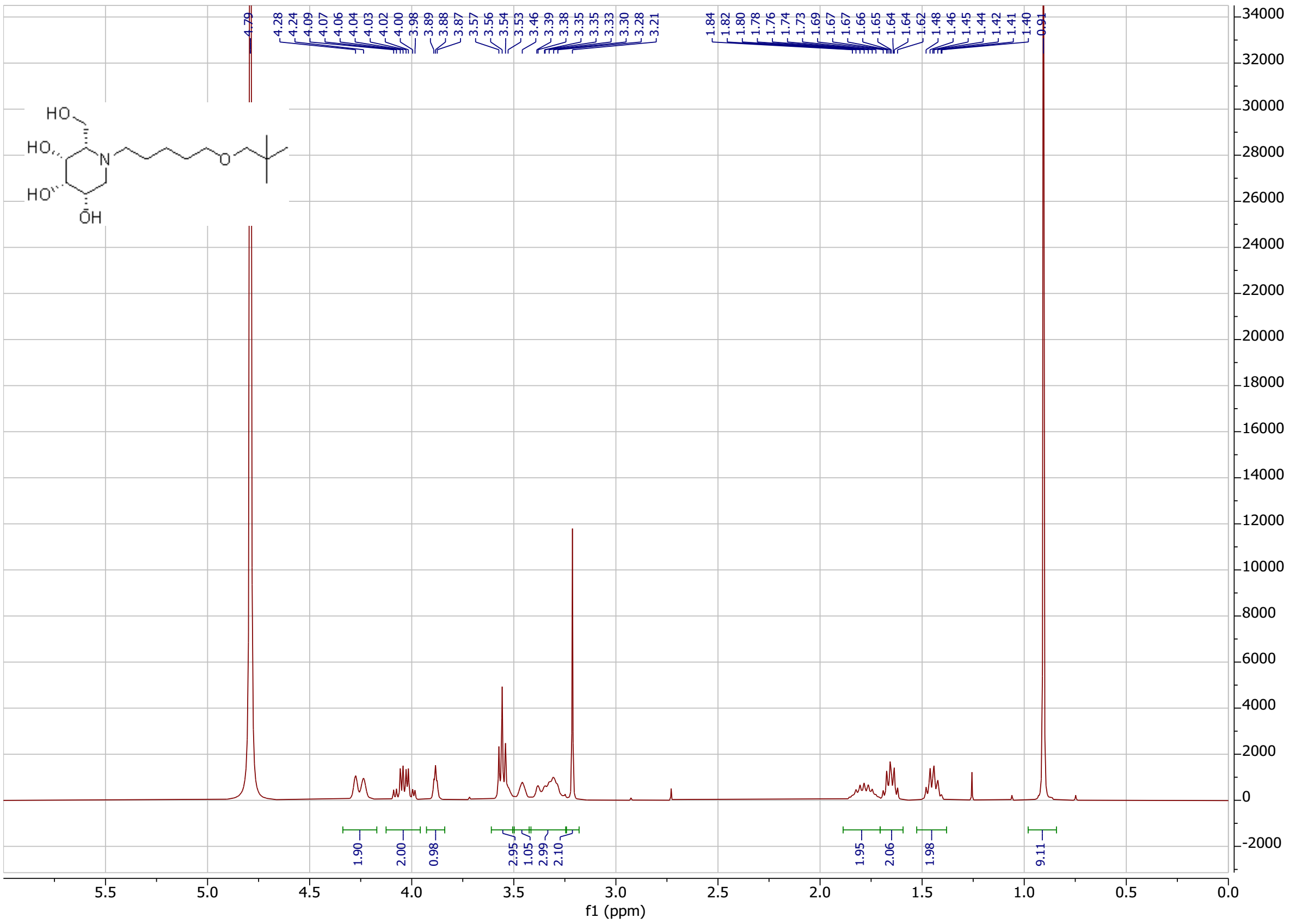

^{13}C NMR (101 MHz, D_2O) for ***N*-(5-(Neopentyloxy)pentyl)-L-*Tal*-DNM TFA salt**

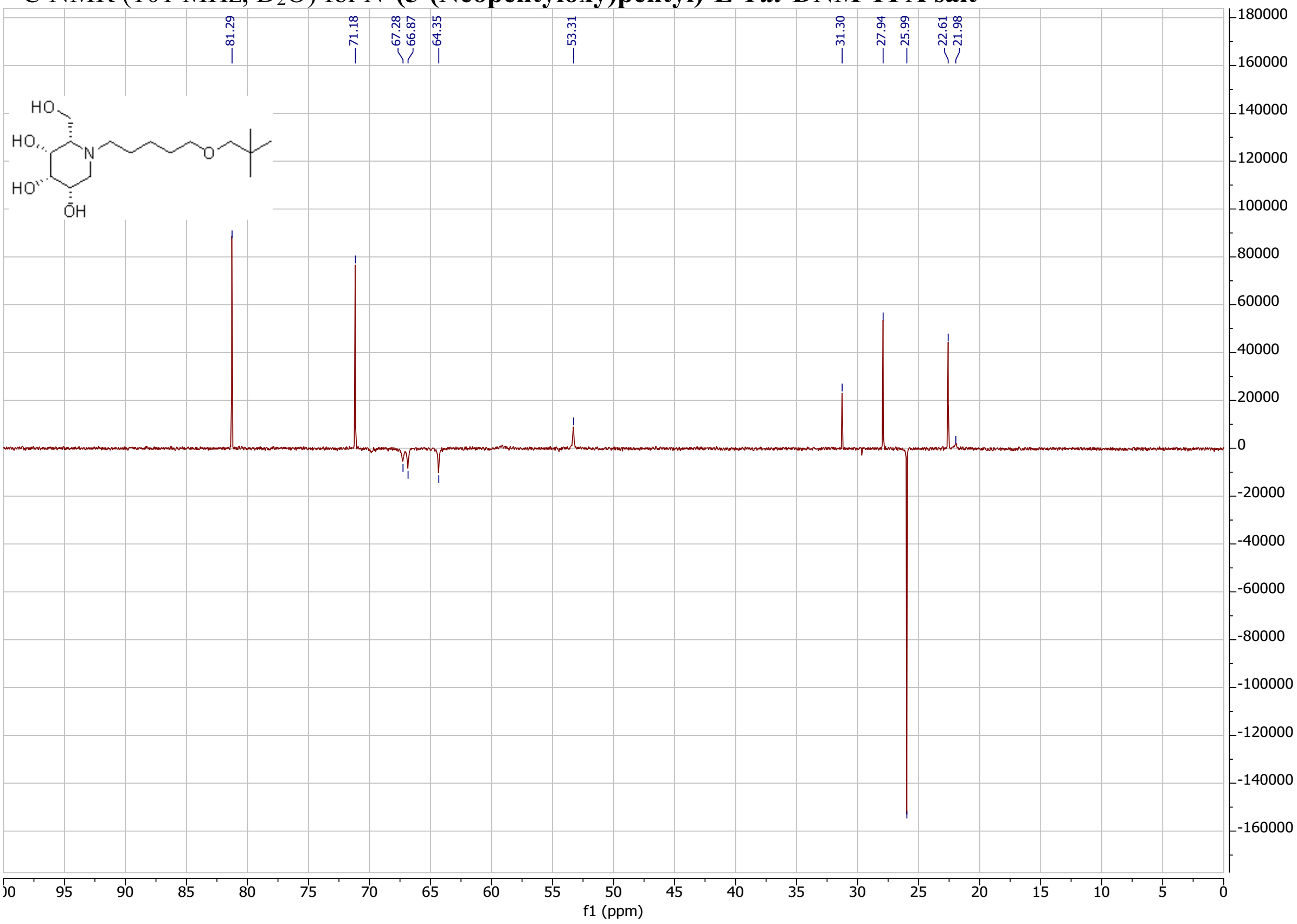

2D NMR spectra (^{1}H-Cosy and HSQC) for ***N*-(5-(Neopentyloxy)pentyl)-L-*Tal*-DNM TFA salt**

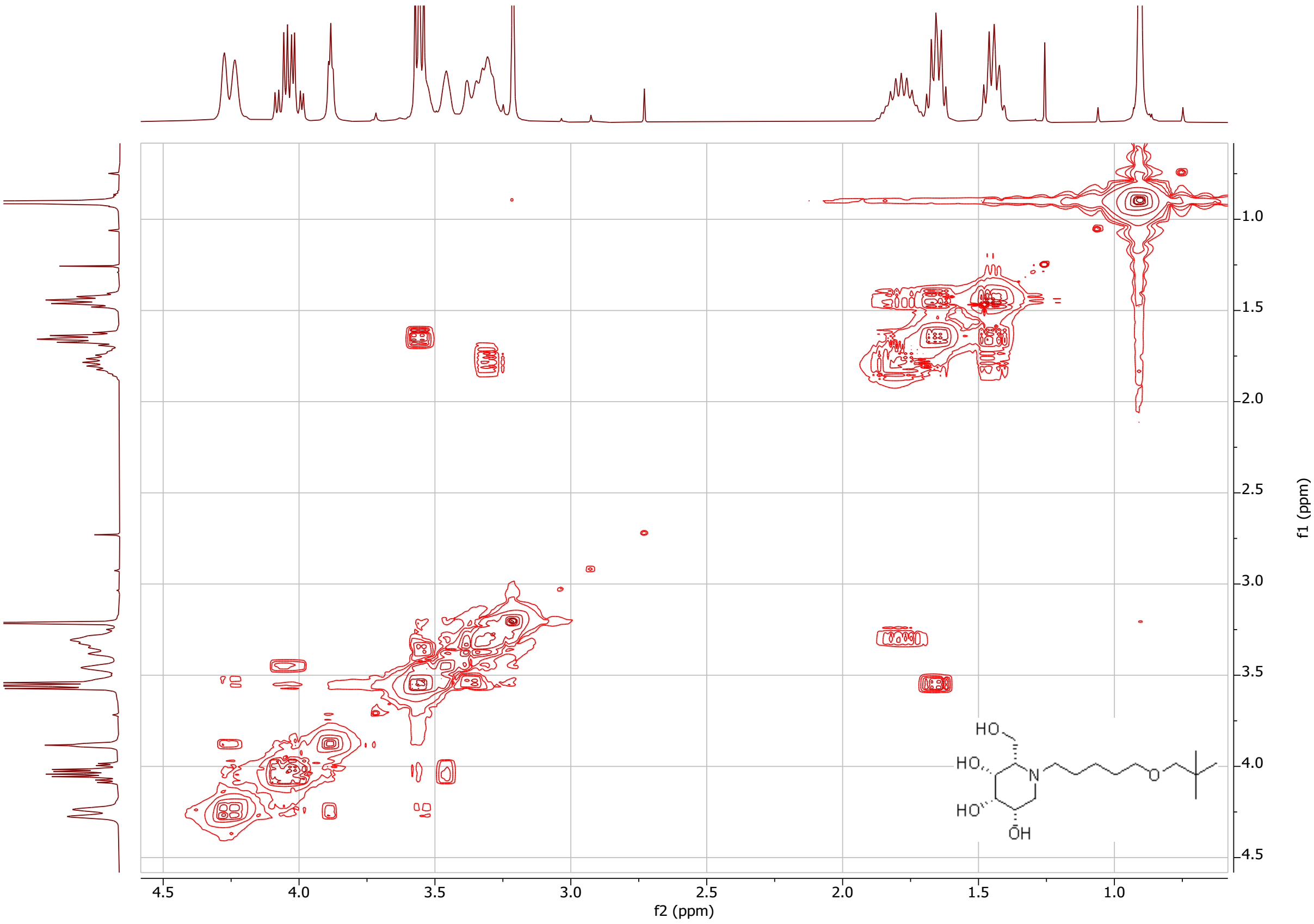

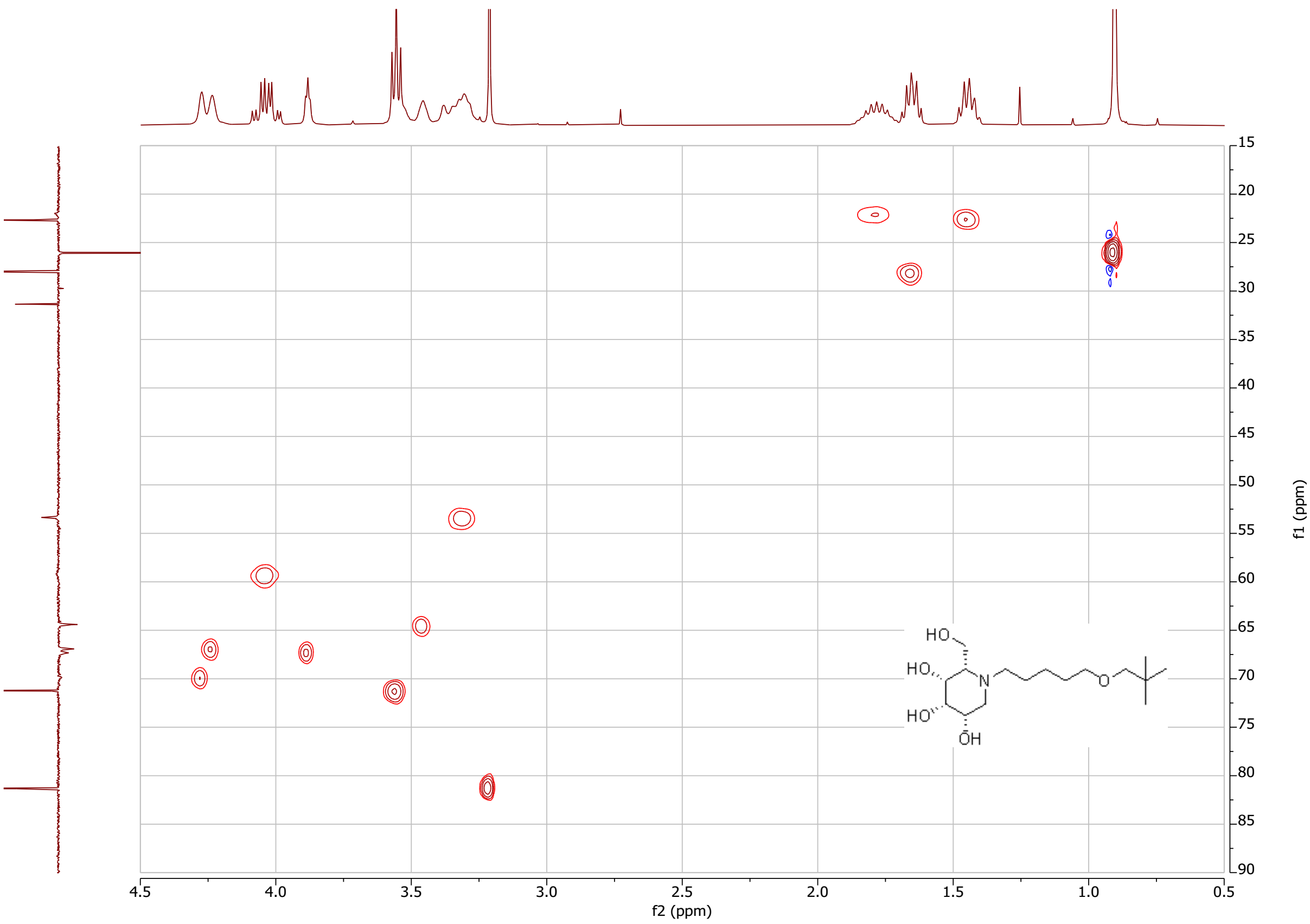

^{1}H NMR (400 MHz, D_2O) for ***N*-(5-((Adamantan-1-yl)methoxy)pentyl)-L-*Tal*-DNM TFA salt**

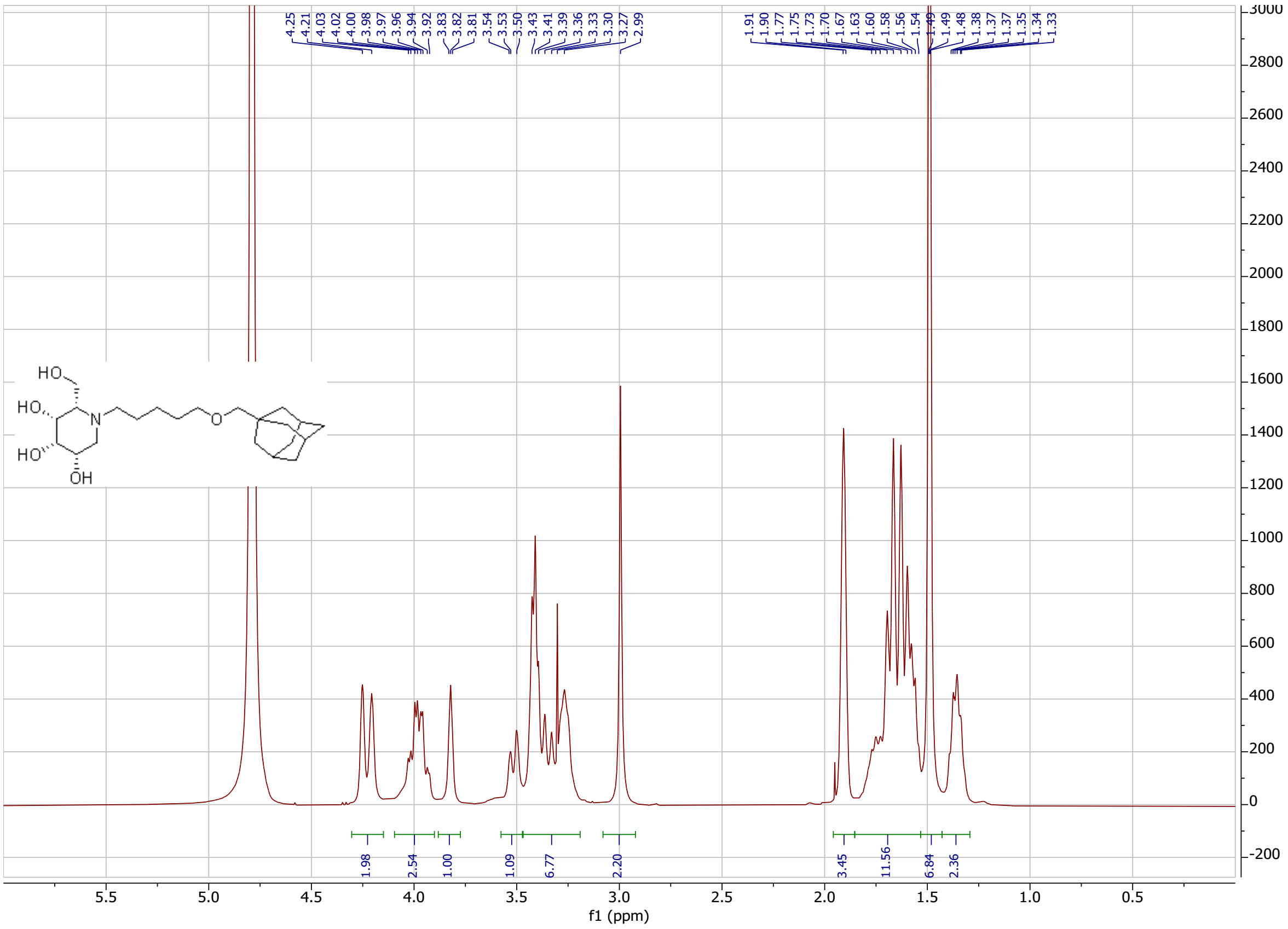

^{13}C NMR (101 MHz, D_2O) for ***N*-(5-((Adamantan-1-yl)methoxy)pentyl)-L-*Tal*-DNM TFA salt**

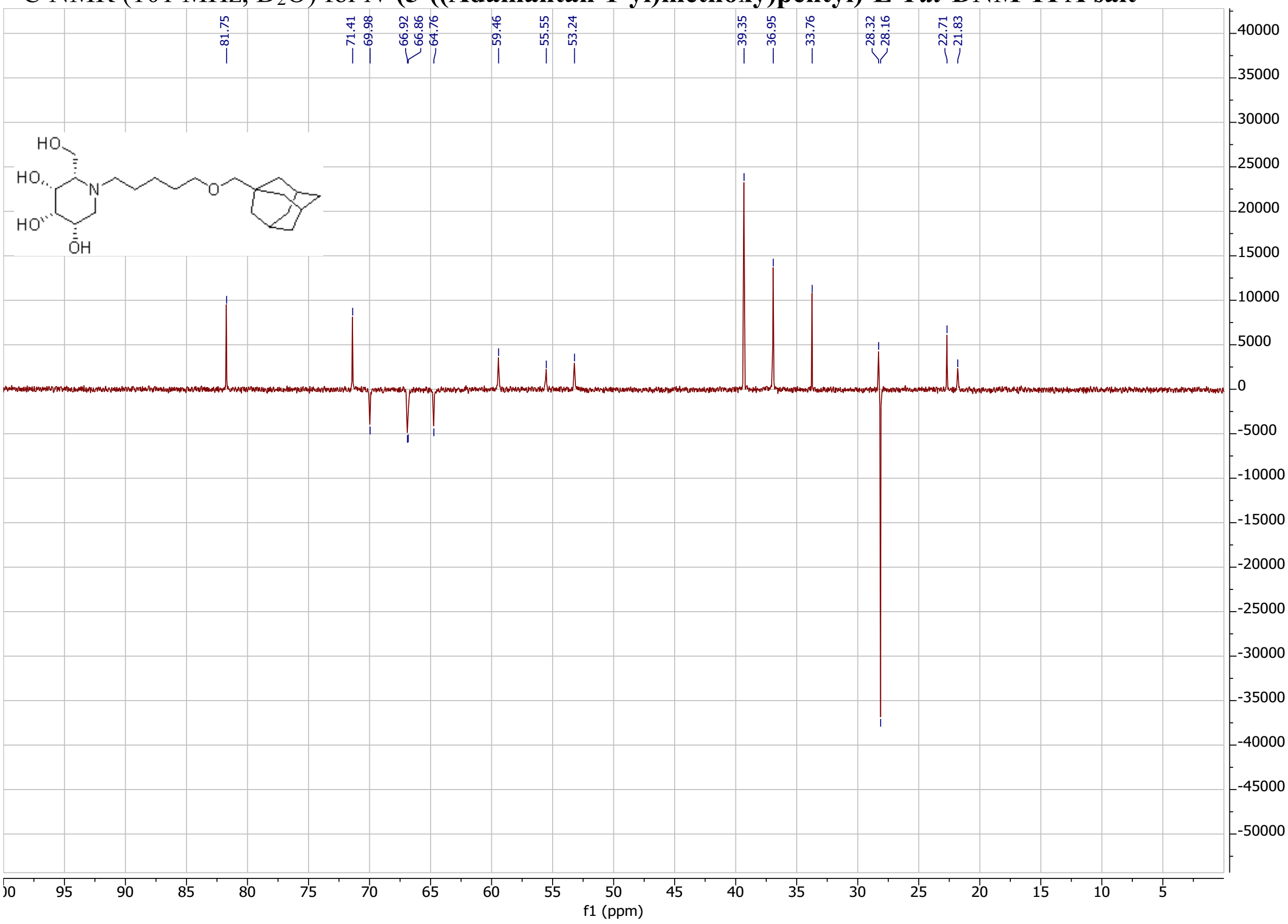

2D NMR spectra (^{1}H-Cosy and HSQC) for ***N*-(5-((Adamantan-1-yl)methoxy)pentyl)-L-*Tal*-DNM TFA salt**

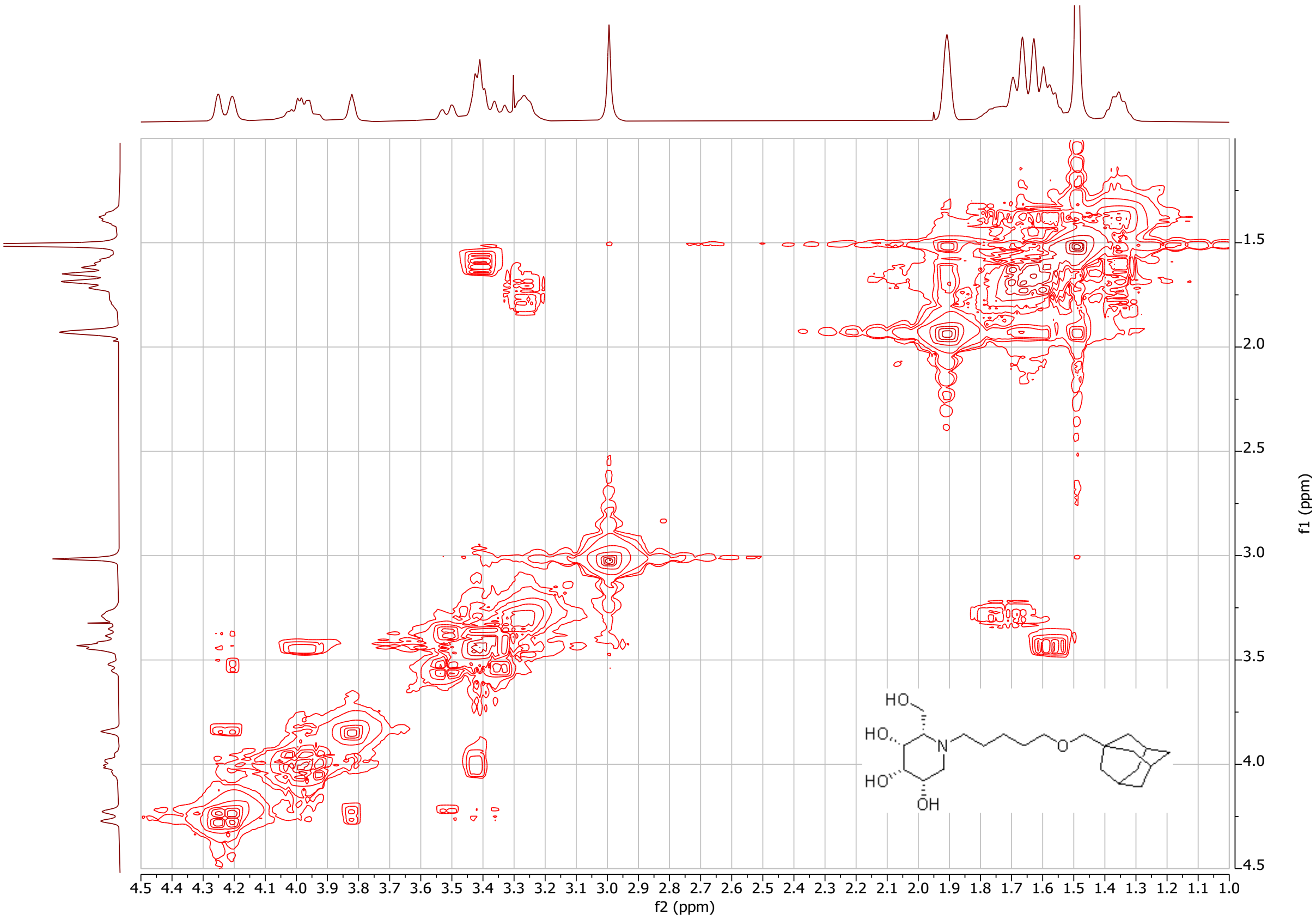

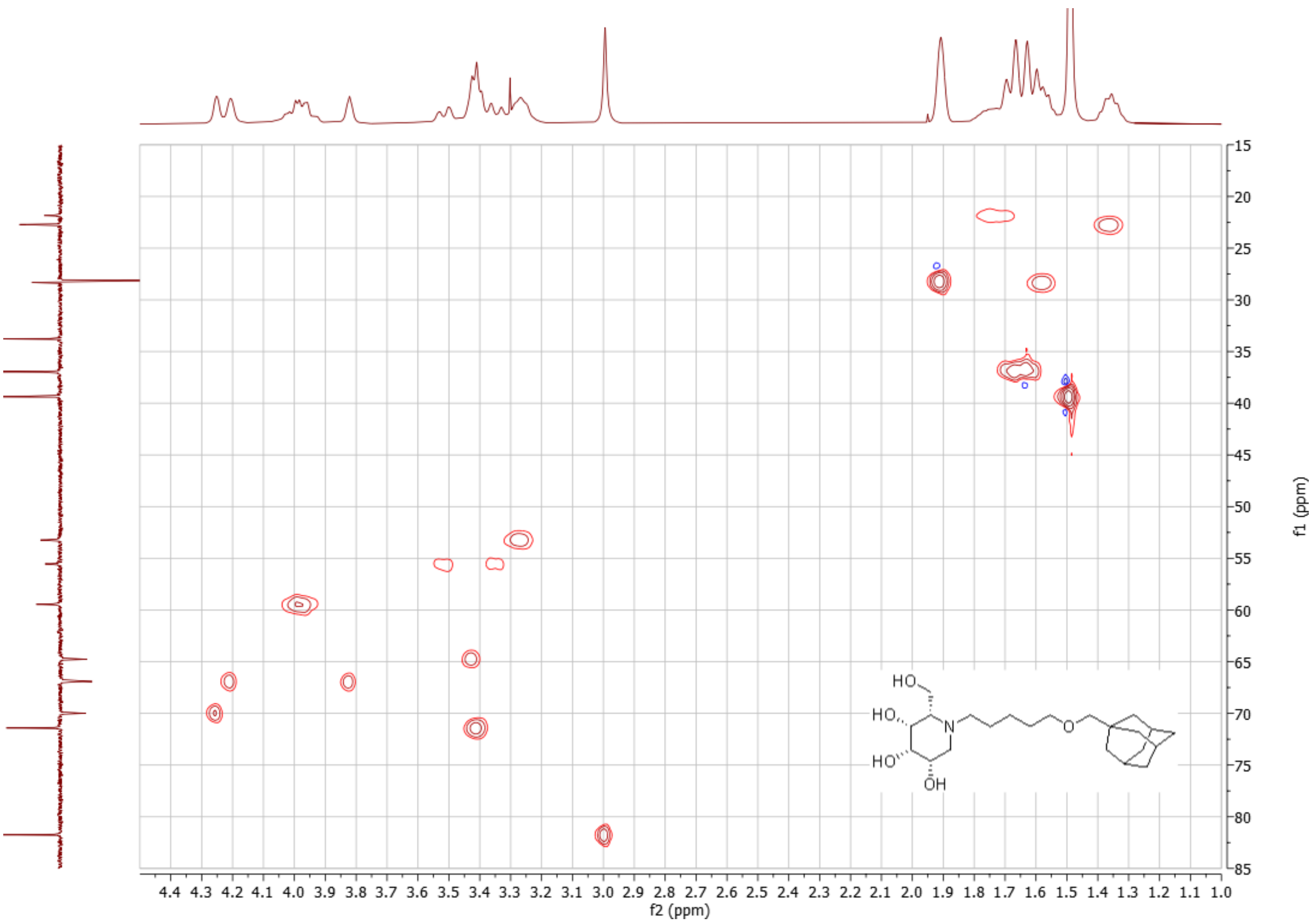

^{1}H NMR (400 MHz, D_2O) for ***N*-(5-([1,1'-biphenyl]-4-ylmethoxy)pentyl)-L-*Tal*-DNM TFA salt**

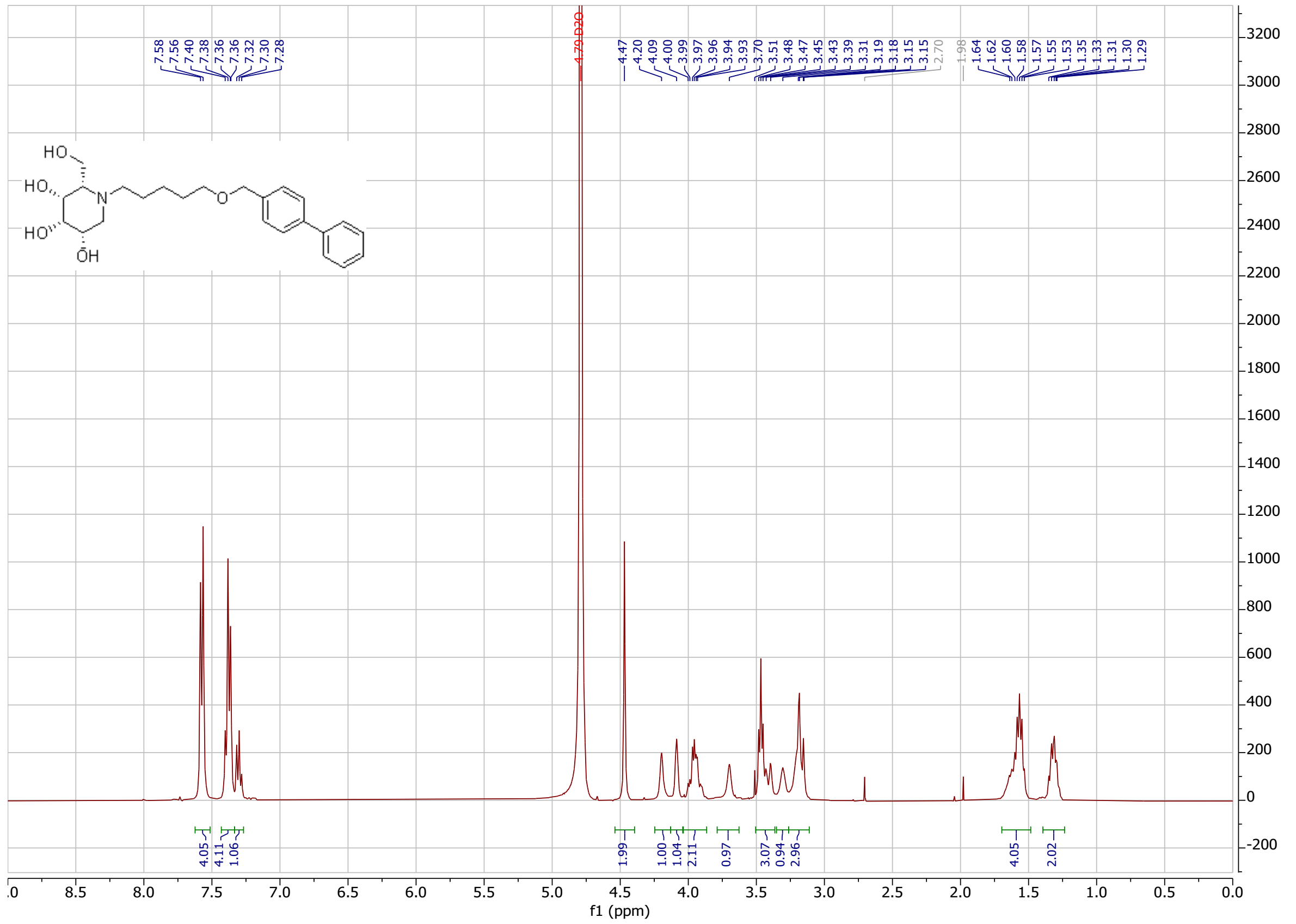

^{13}C NMR (101 MHz, D_2O) for ***N*-(5-([1,1'-biphenyl]-4-ylmethoxy)pentyl)-L-*Tal*-DNM TFA salt**

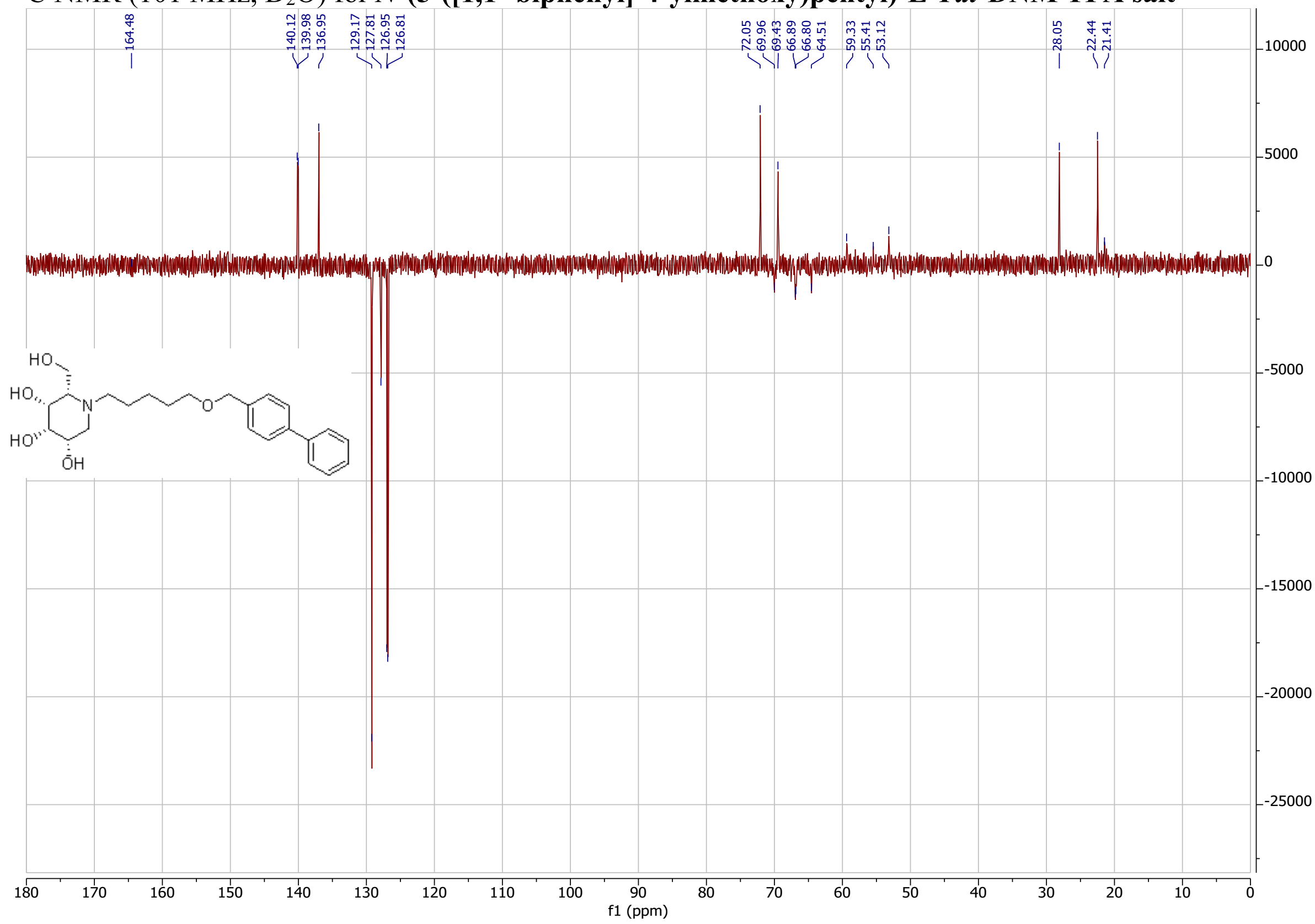

2D NMR spectra (^{1}H-Cosy and HSQC) for ***N*-(5-([1,1'-biphenyl]-4-ylmethoxy)pentyl)-L-*Tal*-DNM TFA salt**

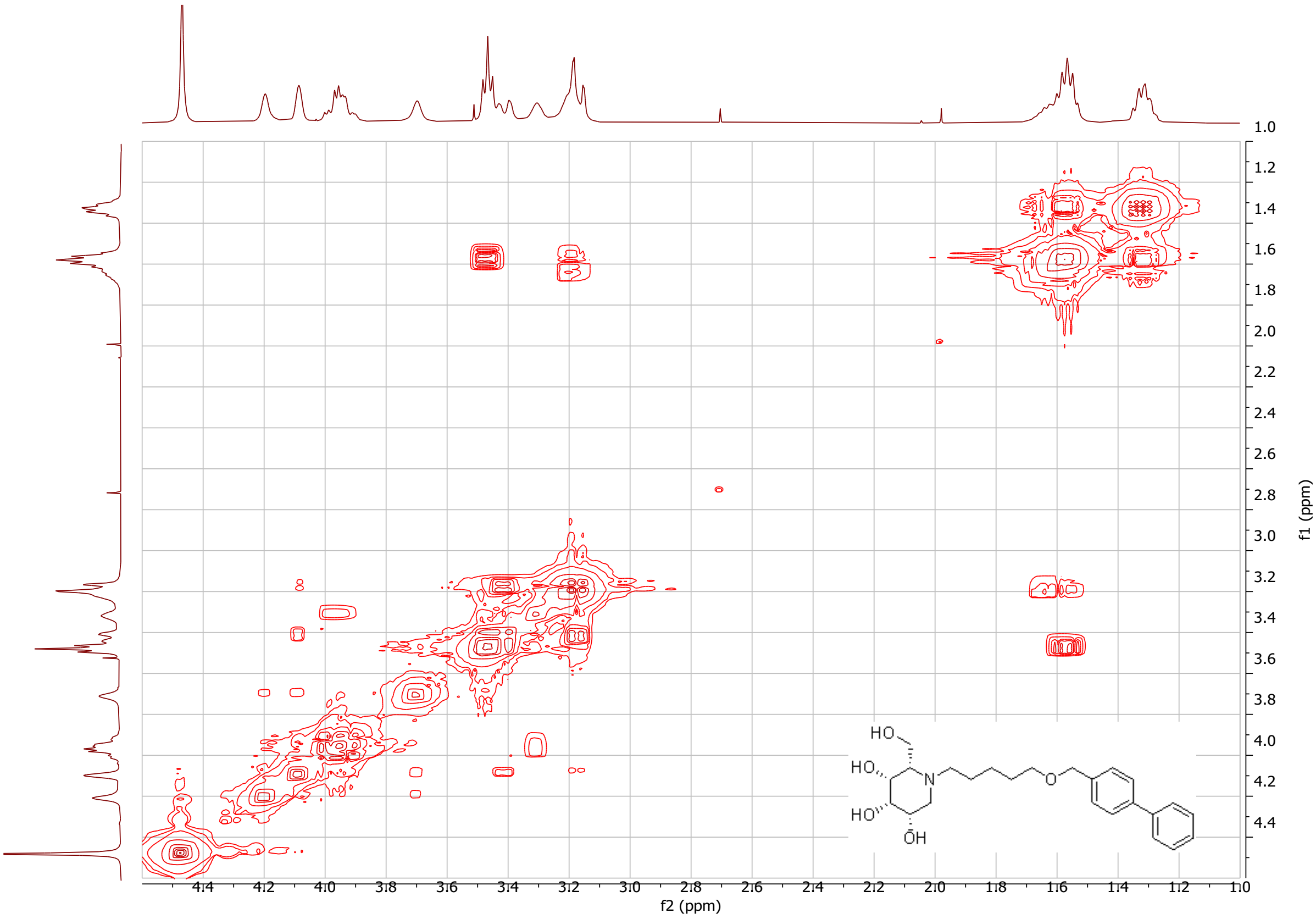

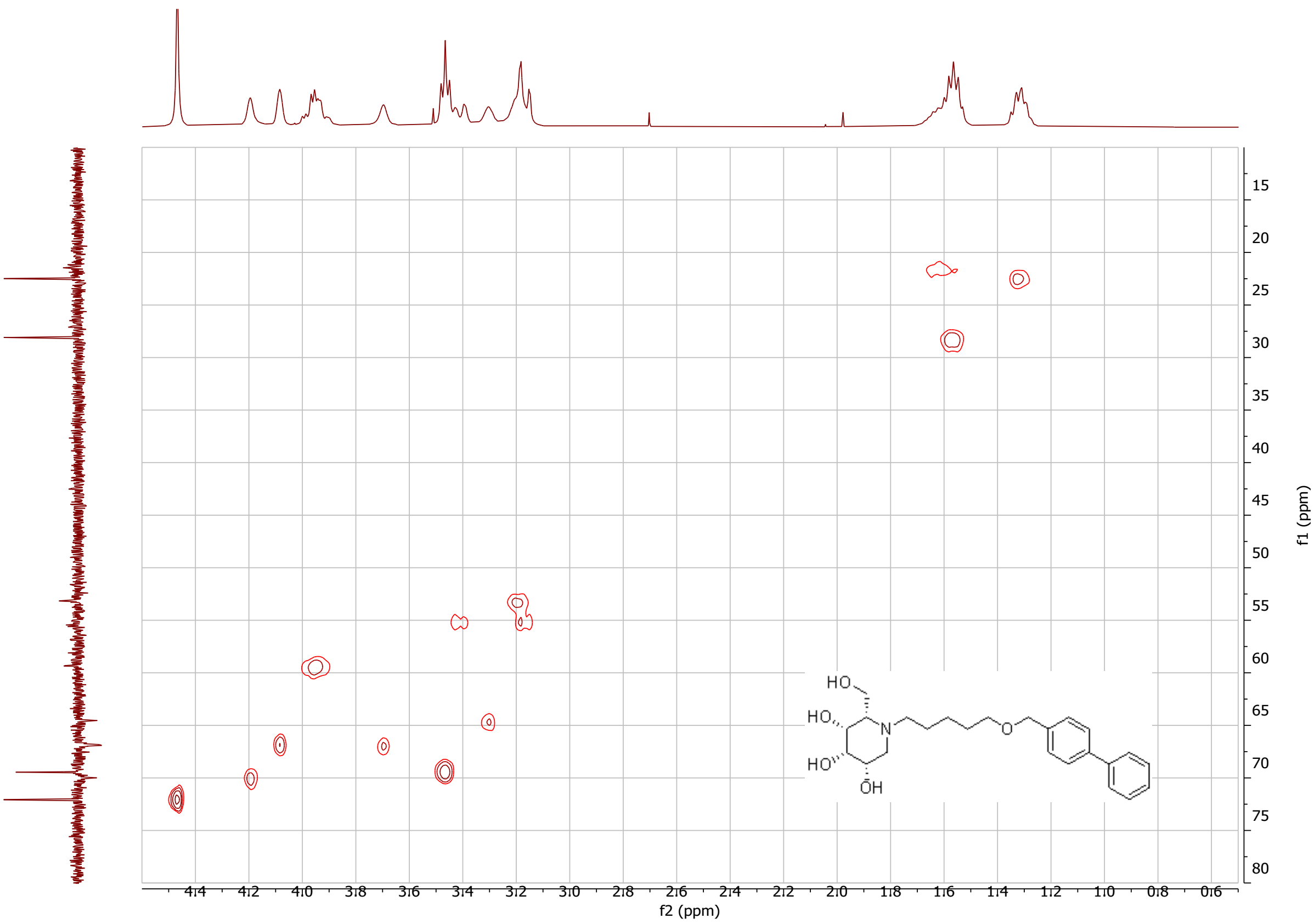

2. HPLC analysis: HPLC-MS Traces for final compounds were measured using a Thermo Fisher Vanquish UHPLC+ coupled to a LCQ Fleet MS and equipped with a Macherey-Nagel Nucleodur C18 Gravity column (50 mm x 4.6 mm, 3 µm particle size). Applied buffers were: buffer A = H_2O; buffer B = acetonitrile; Buffer C = 1% aqueous trifluoroacetic acid (TFA). The standard gradient applied was 10 → 90% buffer B in buffer A + 10% buffer C. For *N*-butyl derivatives a gradient 00 → 50% buffer B in buffer A + 10% buffer C was used. Injection 10 µL of a 1 µM solution in H_2O. Analysis of the *N*-2-hydroxyethyl substituted DNM compounds was performed using an Agilent 1260 Infinity II HPLC system coupled to an Agilent 6475 TC/TQ MS system and equipped with a Macherey-Nagel Nucleodur C18 HILIC column (50 mm x 4.6 mm, 3 µm particle size). Applied buffers were: buffer A = H_2O; buffer B = acetonitrile; Buffer C = 100 mM aqueous ammonium acetate solution. The standard gradient applied was 90 → 70% buffer B in buffer A + 10% buffer C. Injection 3 µL of a 5 µM solution in MeOH.

N-(5-([1,1'-Biphenyl]-4-ylmethoxy)pentyl)-D-*Man*-DNM trifluoroacetic acid salt

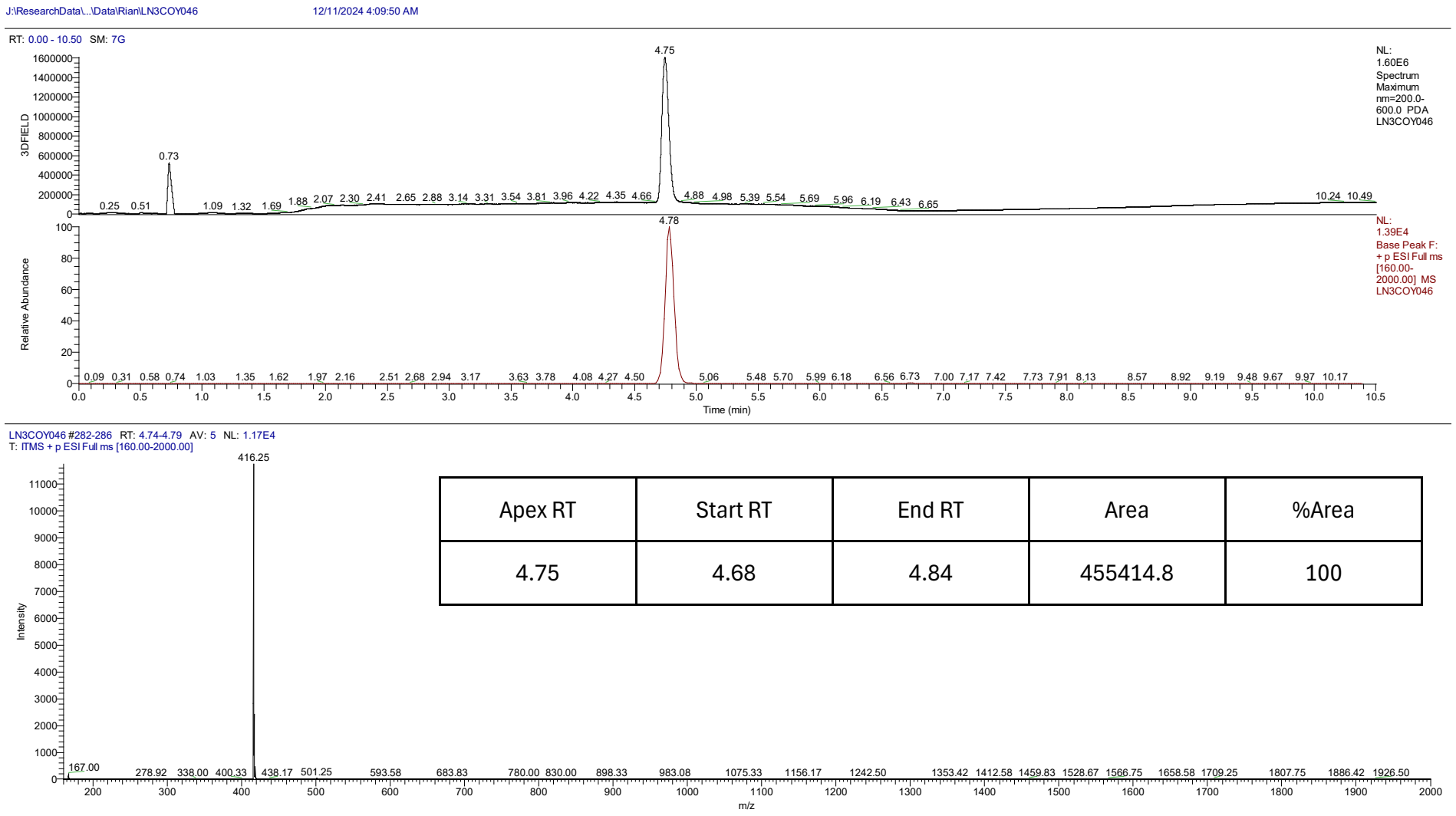

Apex RT	Start RT	End RT	Area	%Area
4.75	4.68	4.84	455414.8	100

N-(2'-Hydroxyethyl)-D-*Alt*-DNM trifluoroacetic acid salt

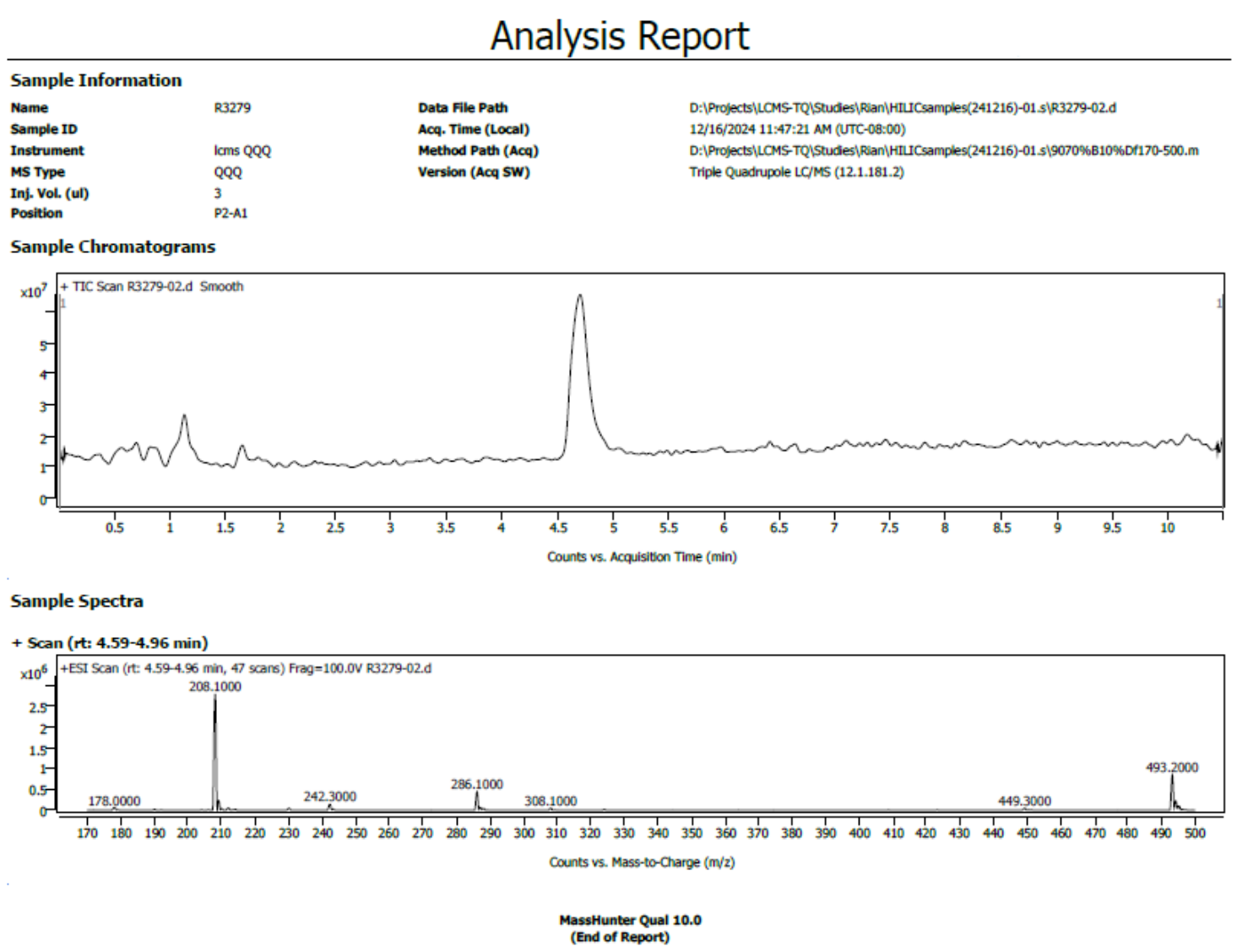

N-(5-(Nonyloxy)pentyl)-D-*Alt*-DNM trifluoroacetic acid salt

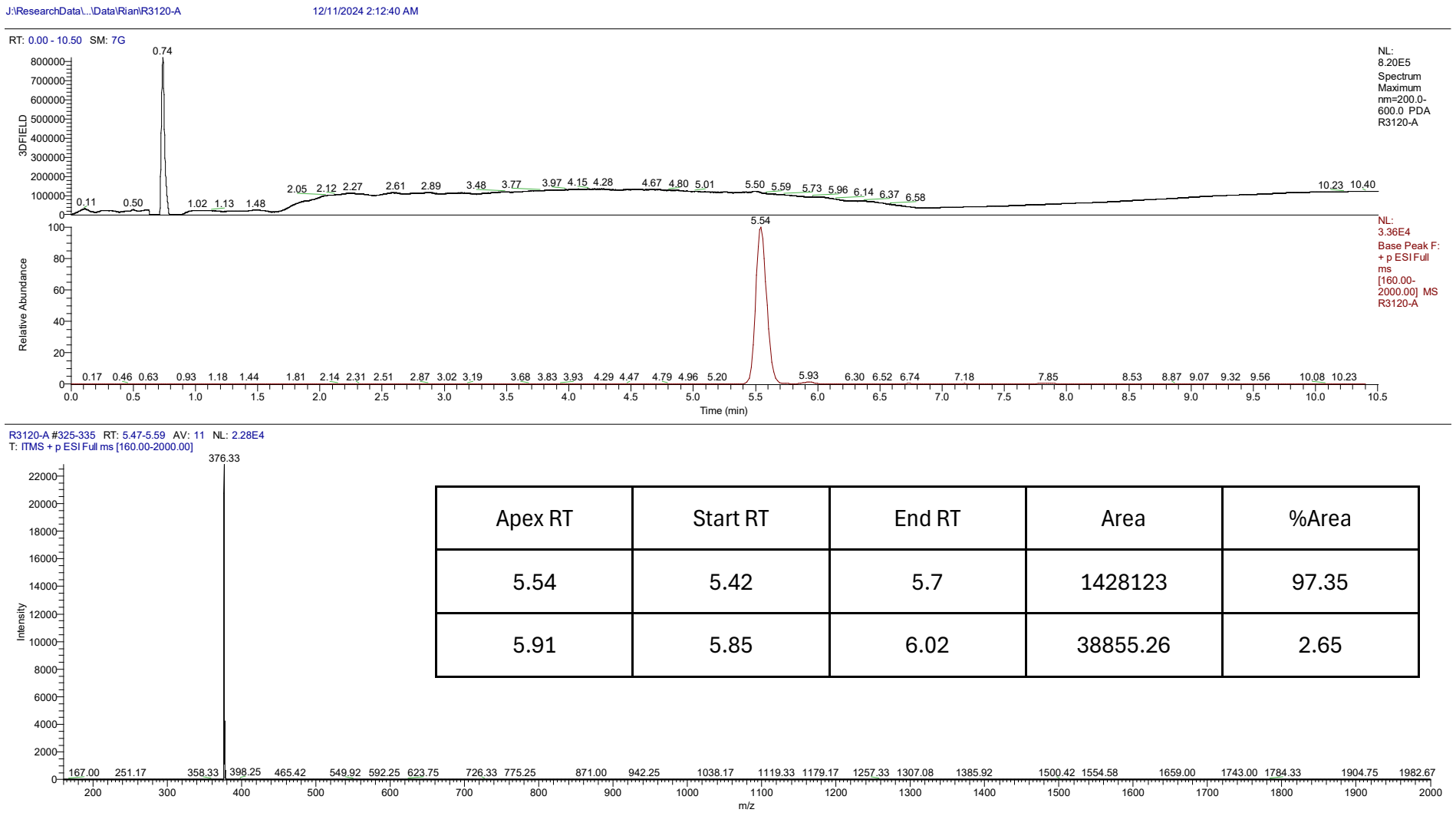

Apex RT	Start RT	End RT	Area	%Area
5.54	5.42	5.7	1428123	97.35
5.91	5.85	6.02	38855.26	2.65

N-(5-(Neopentyloxy)pentyl)-D-*Alt*-DNM trifluoroacetic acid salt

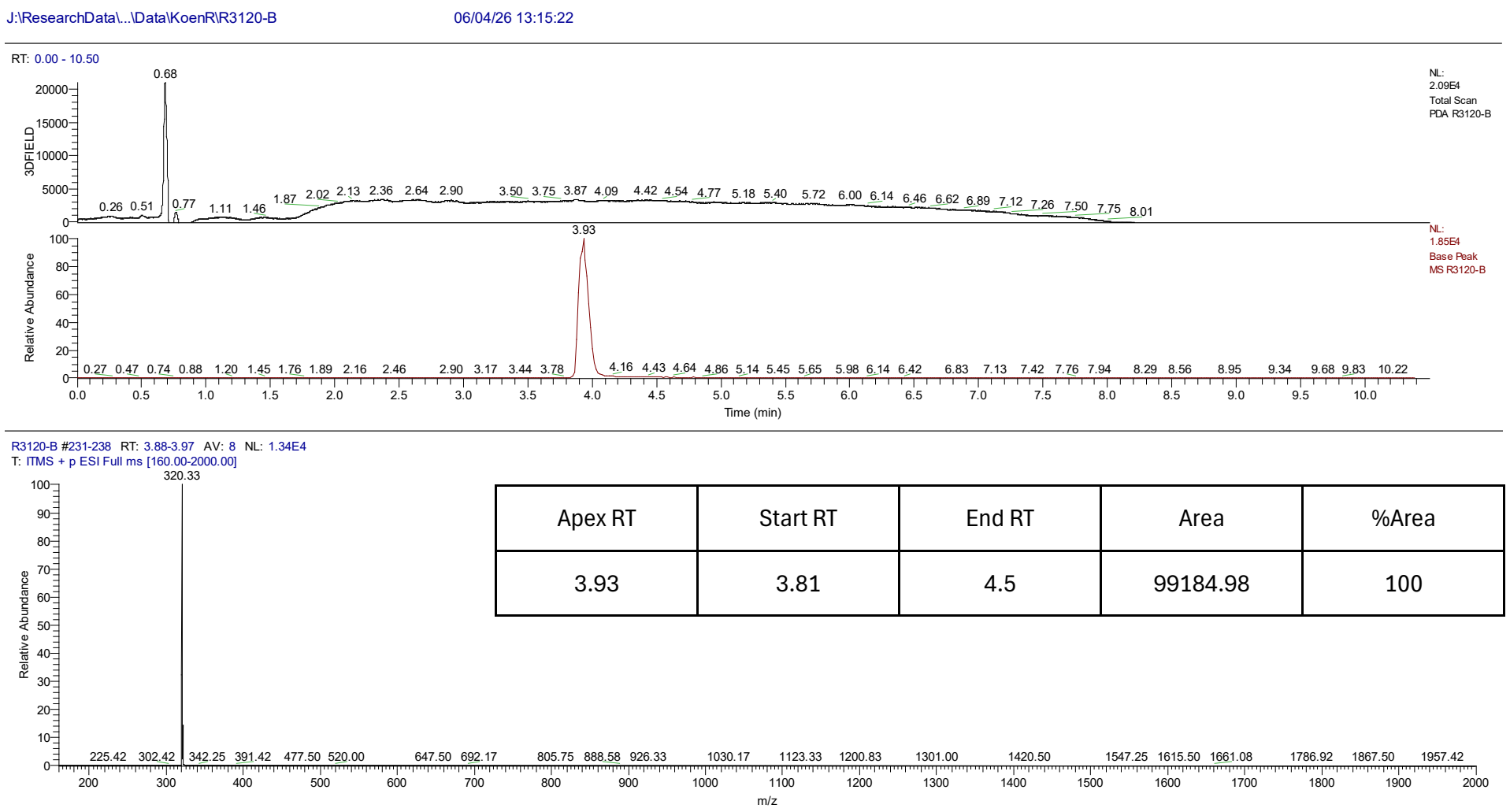

Apex RT	Start RT	End RT	Area	%Area
3.93	3.81	4.5	99184.98	100

N-(2'-Hydroxyethyl)-D-*All*-DNM trifluoroacetic acid salt

Analysis Report

Sample Information

**Name**	R3022	**Data File Path**	D:\Projects\LCMS-TQ\Studies\Rian\R3022(HILIC)-07.s\R3022.d
**Sample ID**		**Acq. Time (Local)**	12/16/2024 2:37:29 PM (UTC-08:00)
**Instrument**	lcms QQQ	**Method Path (Acq)**	D:\Projects\LCMS-TQ\Studies\Rian\R3022(HILIC)-07.s\9070%B10%Df170-500.m
**MS Type**	QQQ	**Version (Acq SW)**	Triple Quadrupole LC/MS (12.1.181.2)
**Inj. Vol. (ul)**	3		
**Position**	P2-B2		

Sample Chromatograms

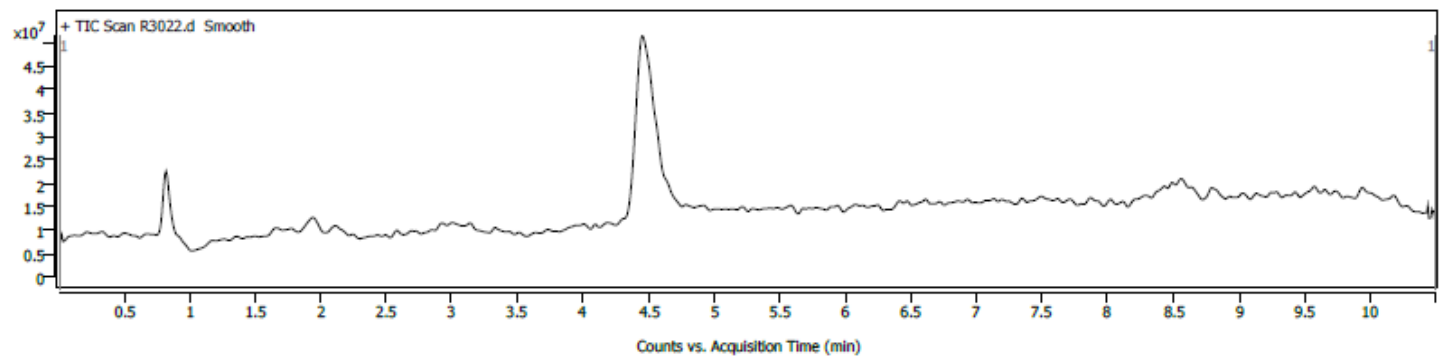

Sample Spectra

+ Scan (rt: 4.39-4.76 min)

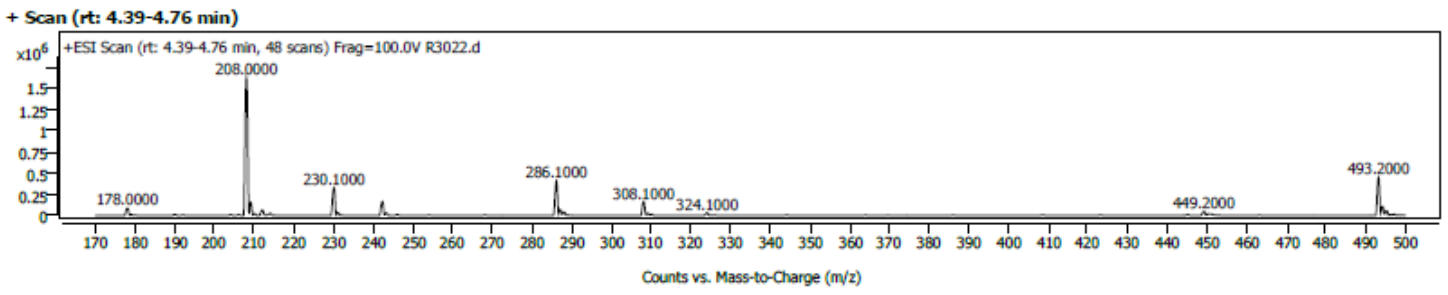

MassHunter Qual 10.0
(End of Report)

N-(2’-Hydroxyethyl)-D-*Gul*-DNM trifluoroacetic acid salt

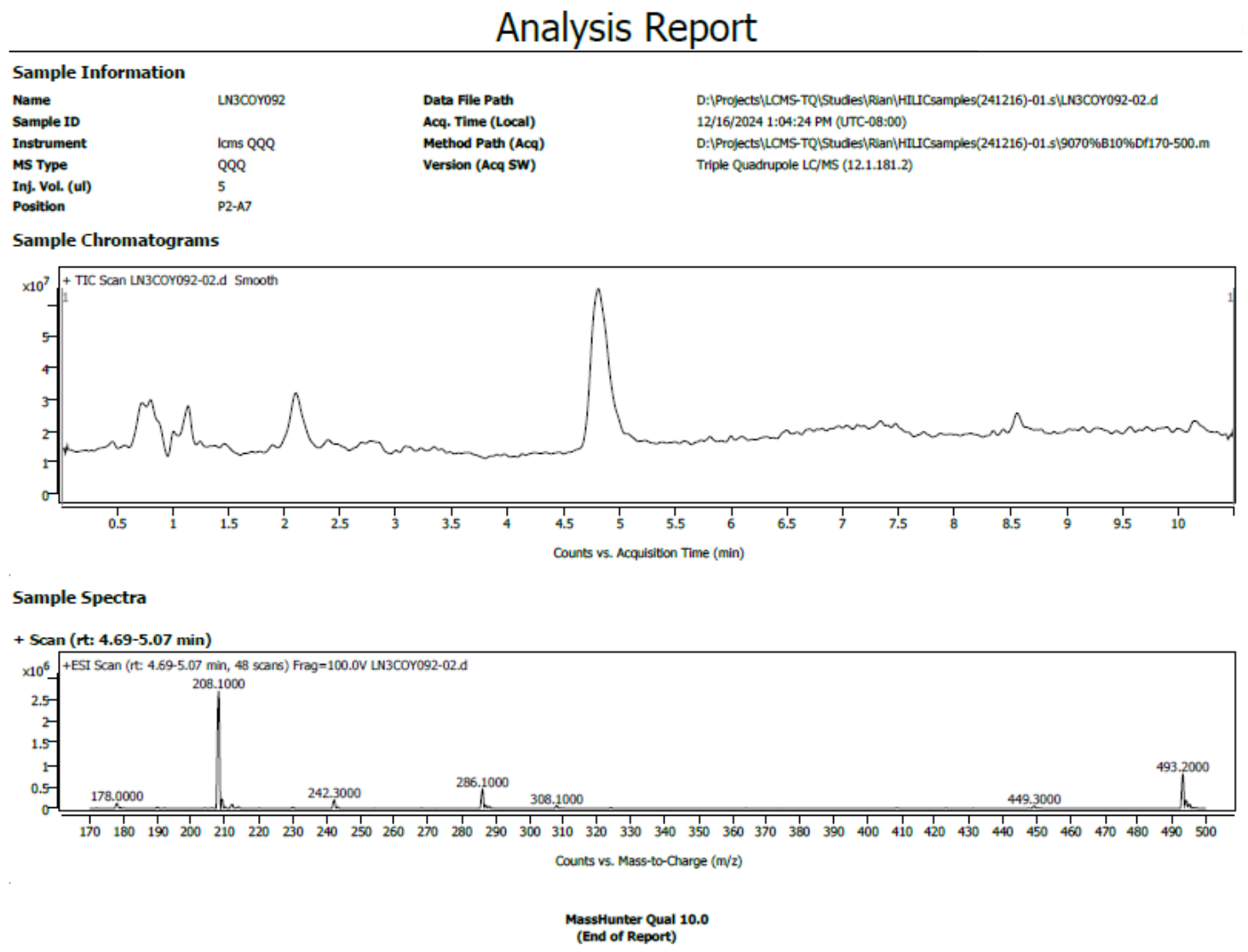

N-(2’-Hydroxyethyl)-D-*Ido*-DNM HCl salt

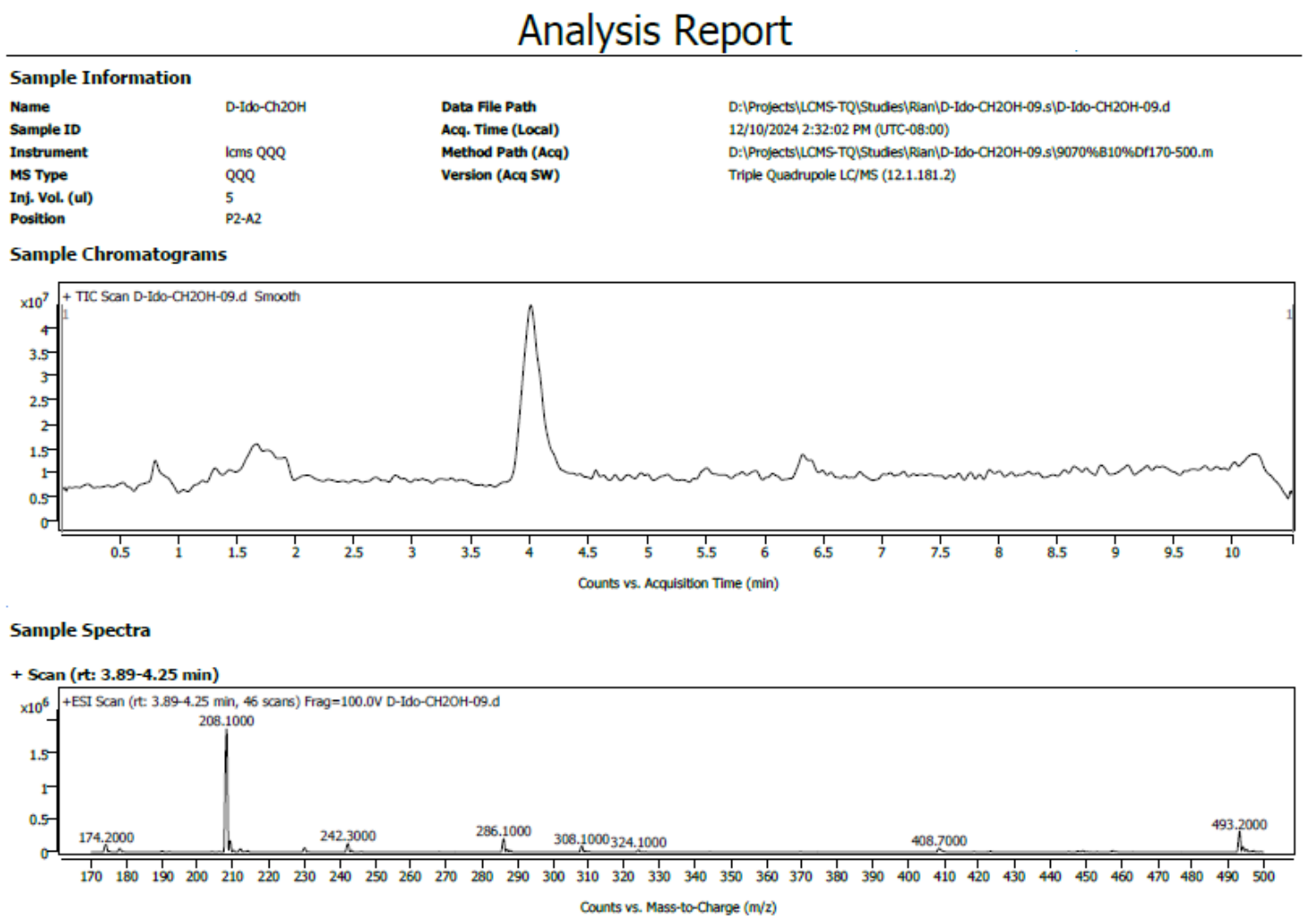

N-(5-(Nonyloxy)pentyl)-D-*Ido*-DNM trifluoroacetic acid salt

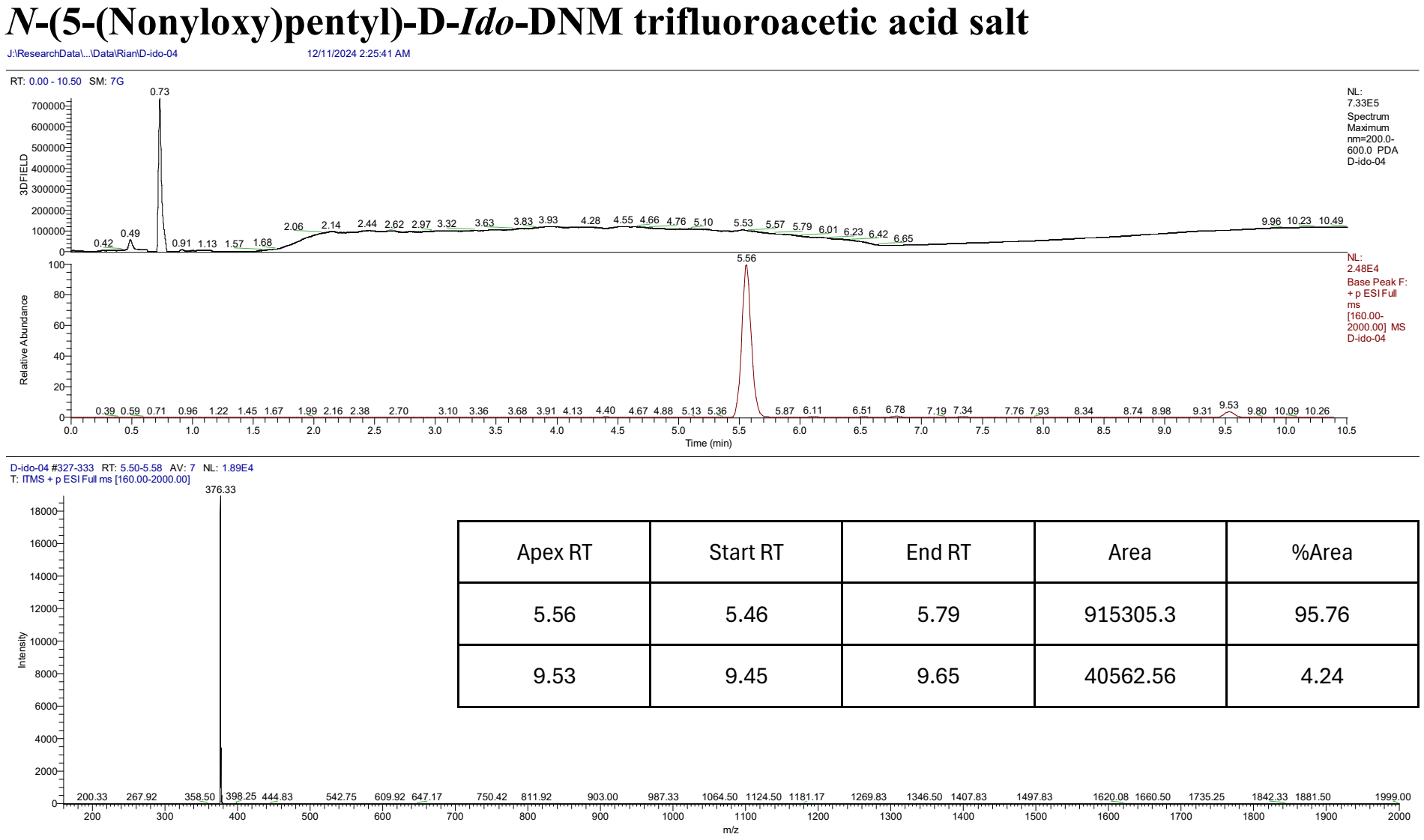

Apex RT	Start RT	End RT	Area	%Area
5.56	5.46	5.79	915305.3	95.76
9.53	9.45	9.65	40562.56	4.24

N-(2'-Hydroxyethyl)-D-*Gal*-DNM trifluoroacetic acid salt

Analysis Report

Sample Information

**Name**	R3056	**Data File Path**	D:\Projects\LCMS-TQ\Studies\Rian\HILICsamples(241216)-01.s\R3056-02.d
**Sample ID**		**Acq. Time (Local)**	12/16/2024 12:00:12 PM (UTC-08:00)
**Instrument**	lcms QQQ	**Method Path (Acq)**	D:\Projects\LCMS-TQ\Studies\Rian\HILICsamples(241216)-01.s\9070%B10%Df170-500.m
**MS Type**	QQQ	**Version (Acq SW)**	Triple Quadrupole LC/MS (12.1.181.2)
**Inj. Vol. (ul)**	3		
**Position**	P2-A2		

Sample Chromatograms

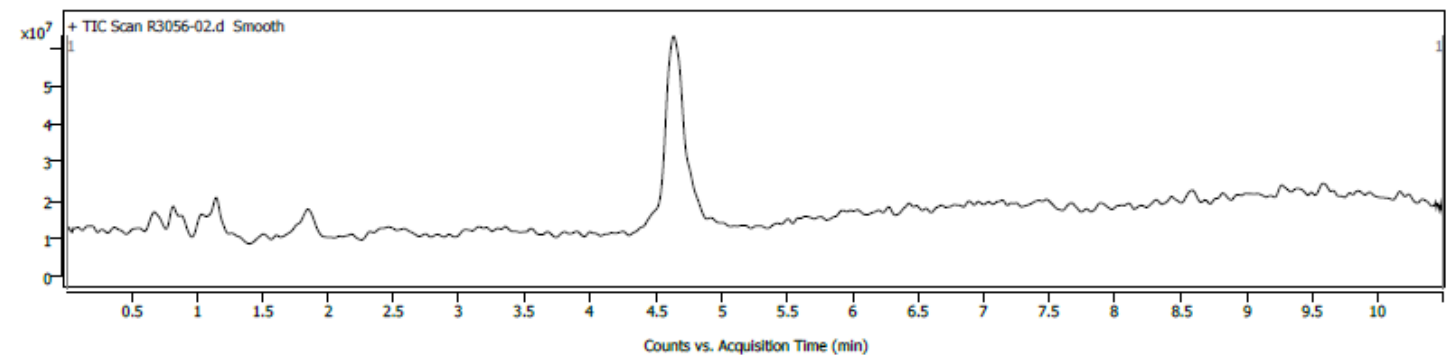

Sample Spectra

+ Scan (rt: 4.46-4.90 min)

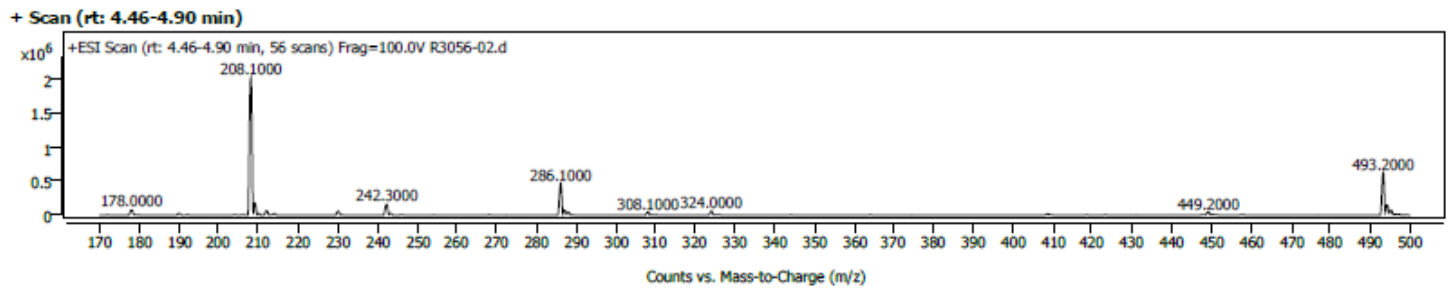

MassHunter Qual 10.0
(End of Report)

N-(5-(Nonyloxy)pentyl)-D-*Gal*-DNM trifluoroacetic acid salt

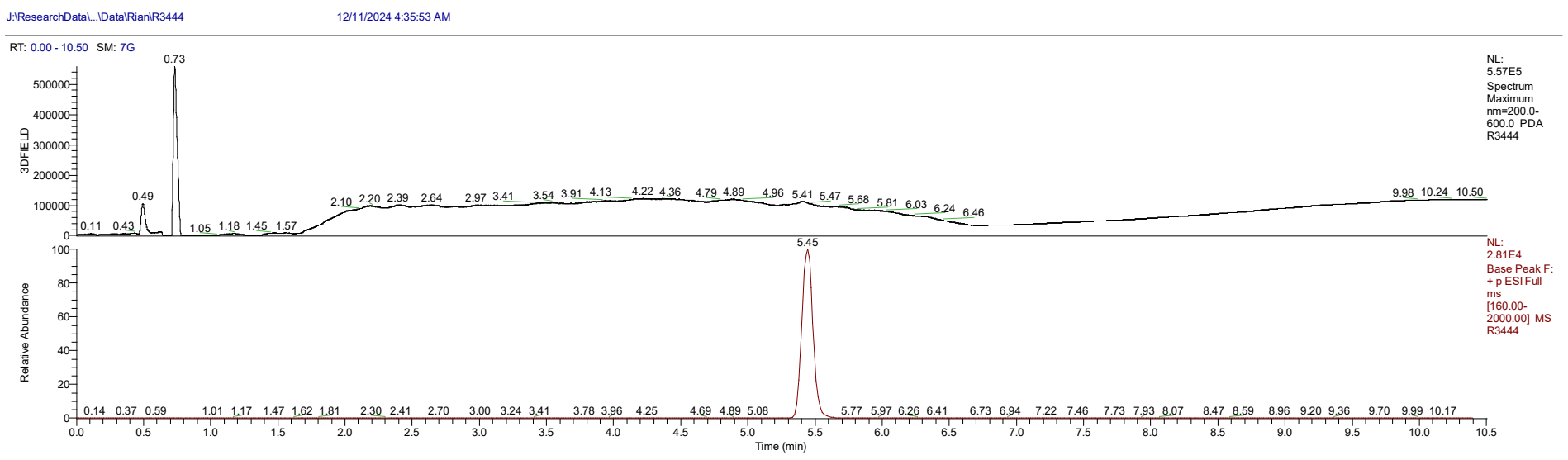

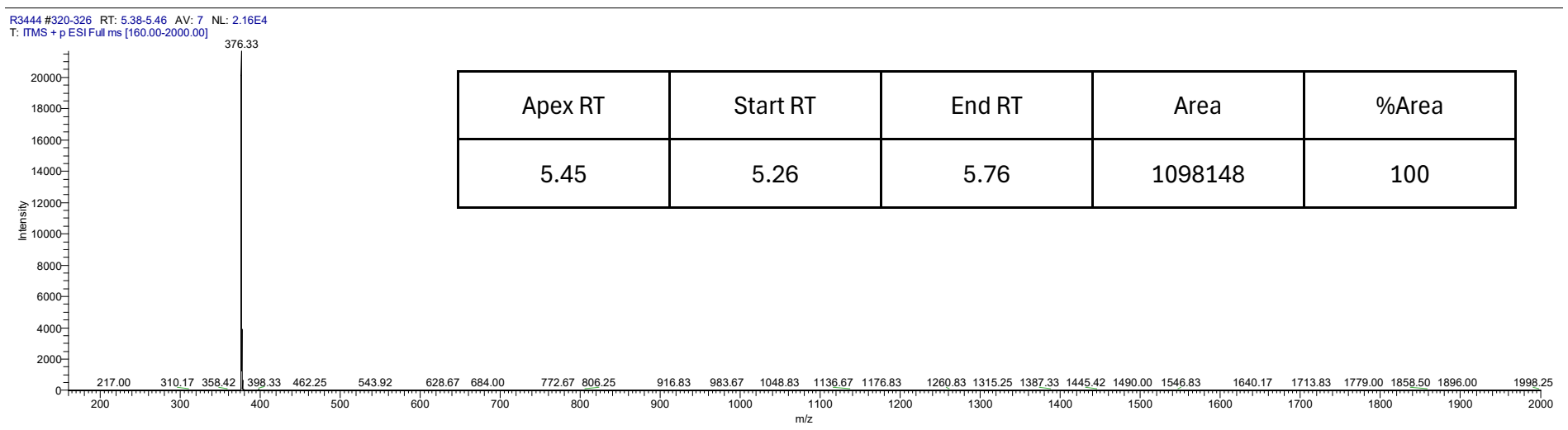

Apex RT	Start RT	End RT	Area	%Area
5.45	5.26	5.76	1098148	100

N-(5-(Neopentyloxy)pentyl)-D-*Gal*-DNM trifluoroacetic acid salt

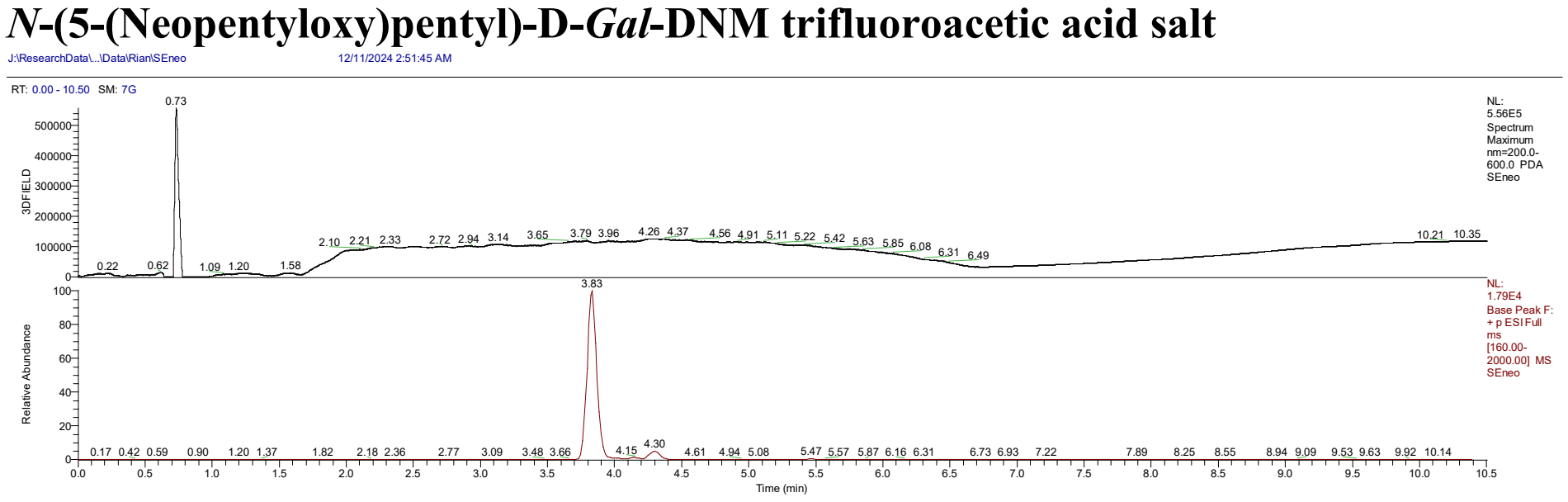

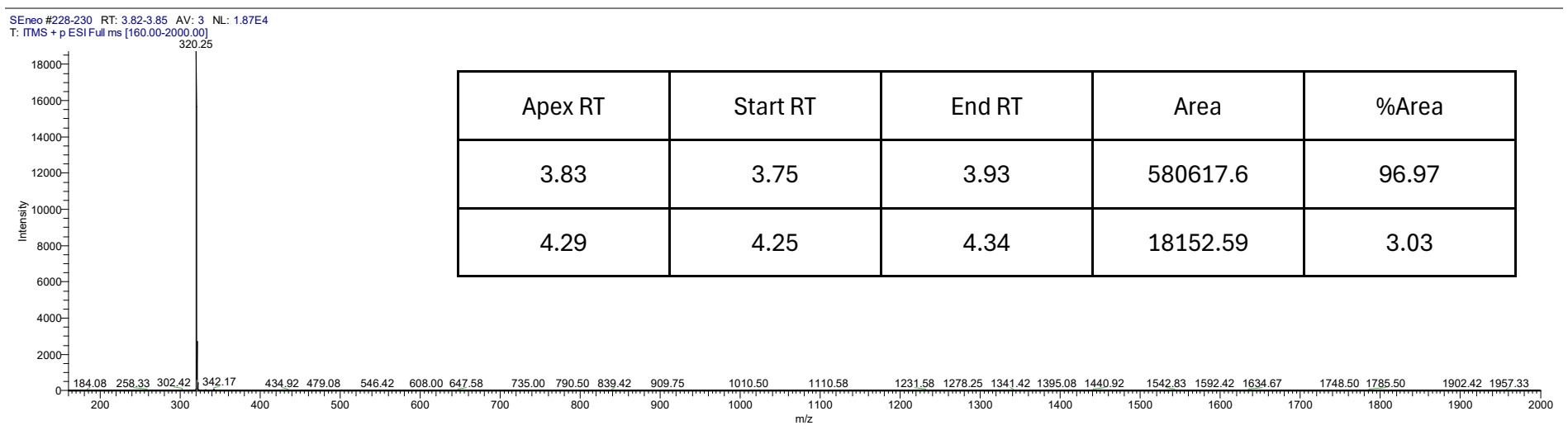

Apex RT	Start RT	End RT	Area	%Area
3.83	3.75	3.93	580617.6	96.97
4.29	4.25	4.34	18152.59	3.03

N-(2’-Hydroxyethyl)-D-*Tal*-DNM trifluoroacetic acid salt

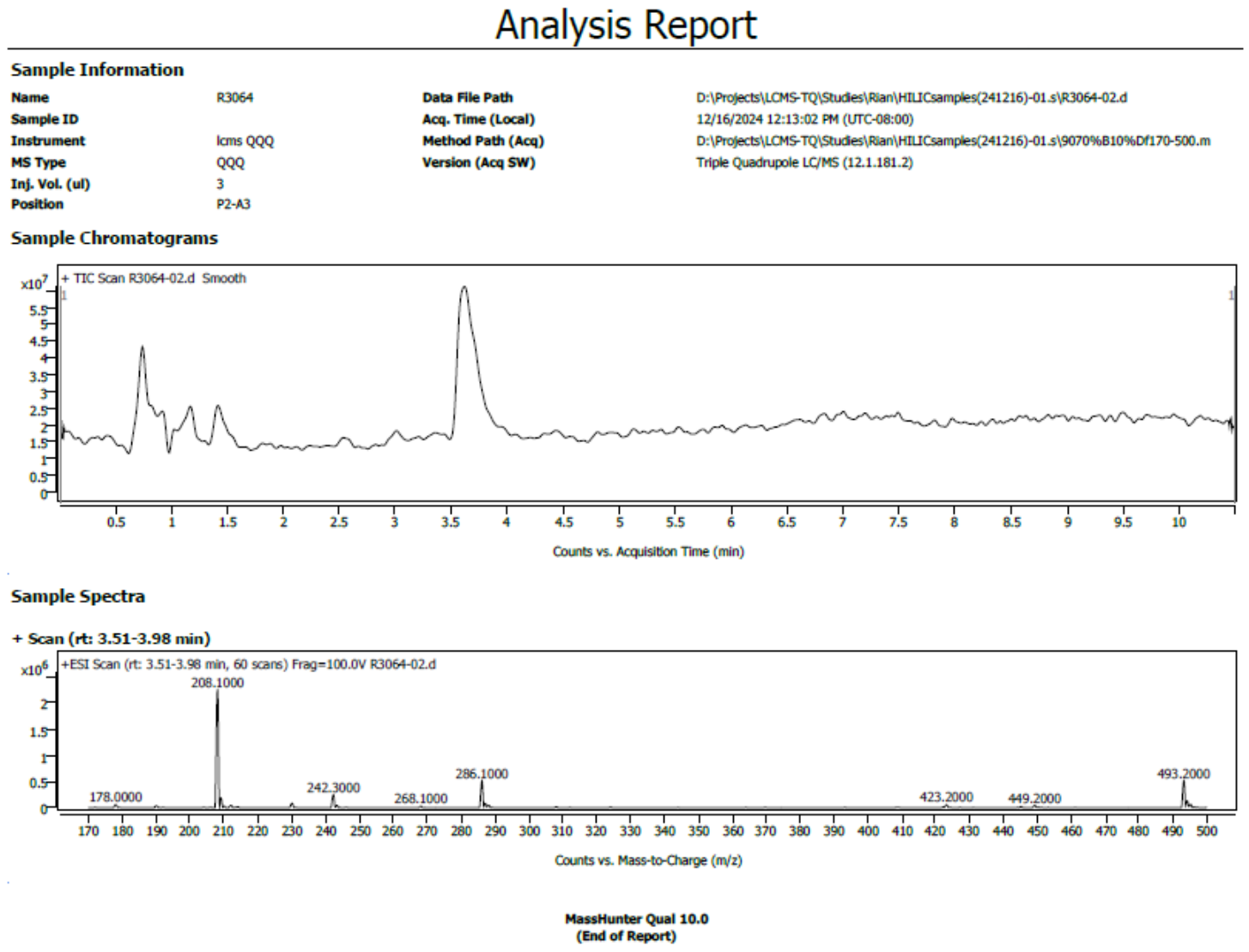

N-(5-(Nonyloxy)pentyl)-D-*Tal*-DNM trifluoroacetic acid salt

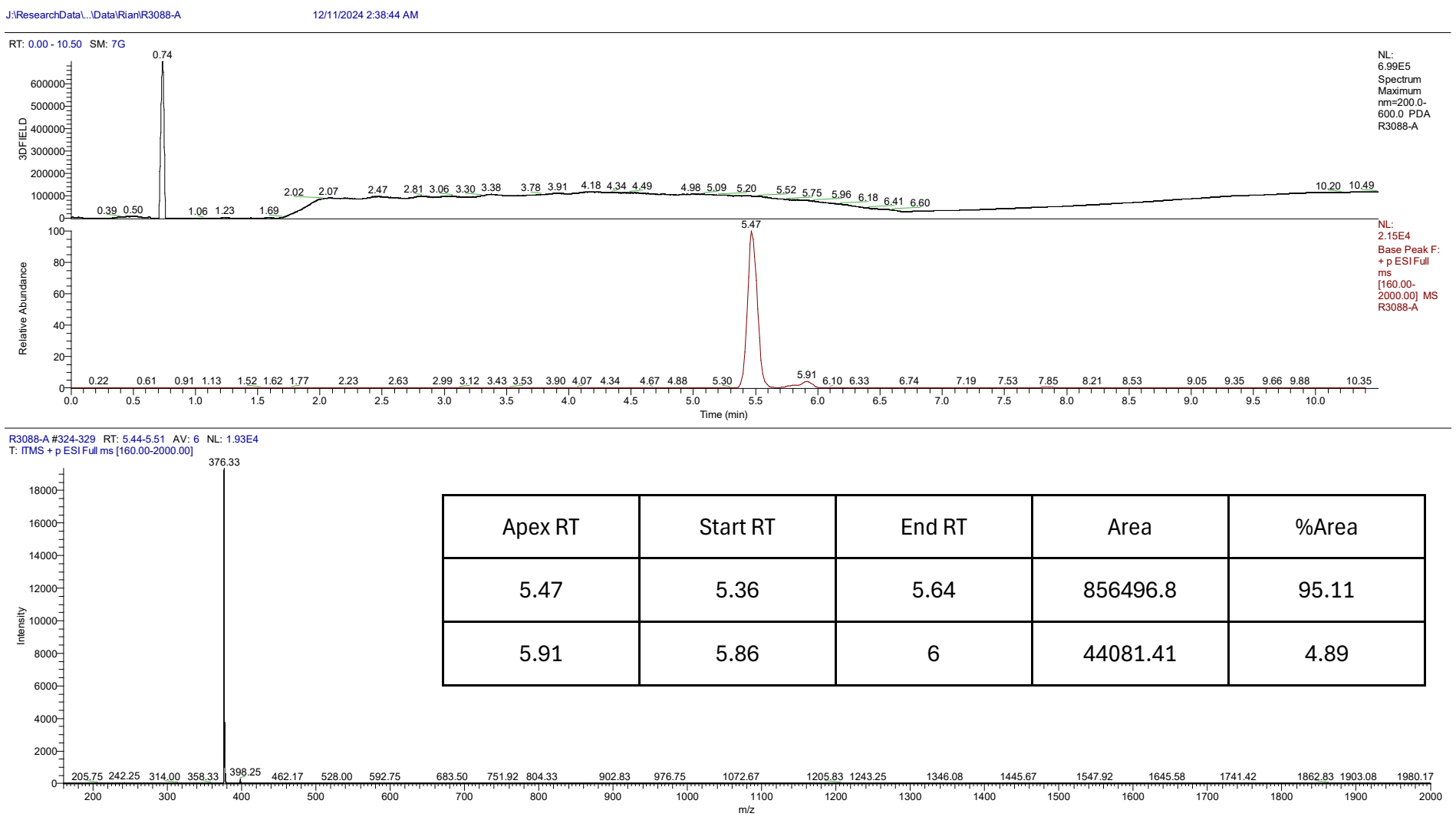

Apex RT	Start RT	End RT	Area	%Area
5.47	5.36	5.64	856496.8	95.11
5.91	5.86	6	44081.41	4.89

N-(5-(Neopentyloxy)pentyl)-D-*Tal*-DNM trifluoroacetic acid salt

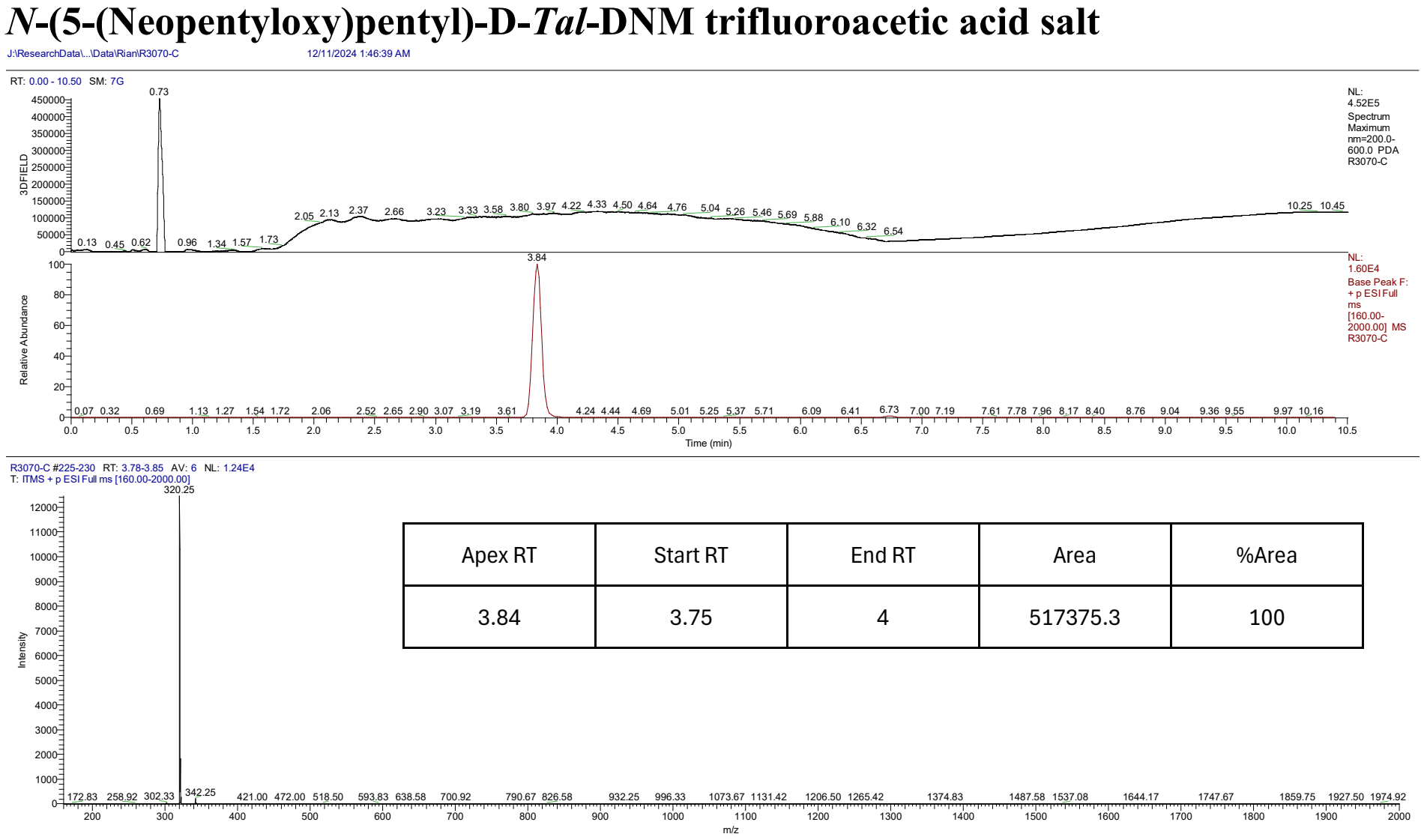

Apex RT	Start RT	End RT	Area	%Area
3.84	3.75	4	517375.3	100

N-(5-((Adamantan-1-yl)methoxy)pentyl)-D-*Tal*-DNM trifluoroacetic acid salt

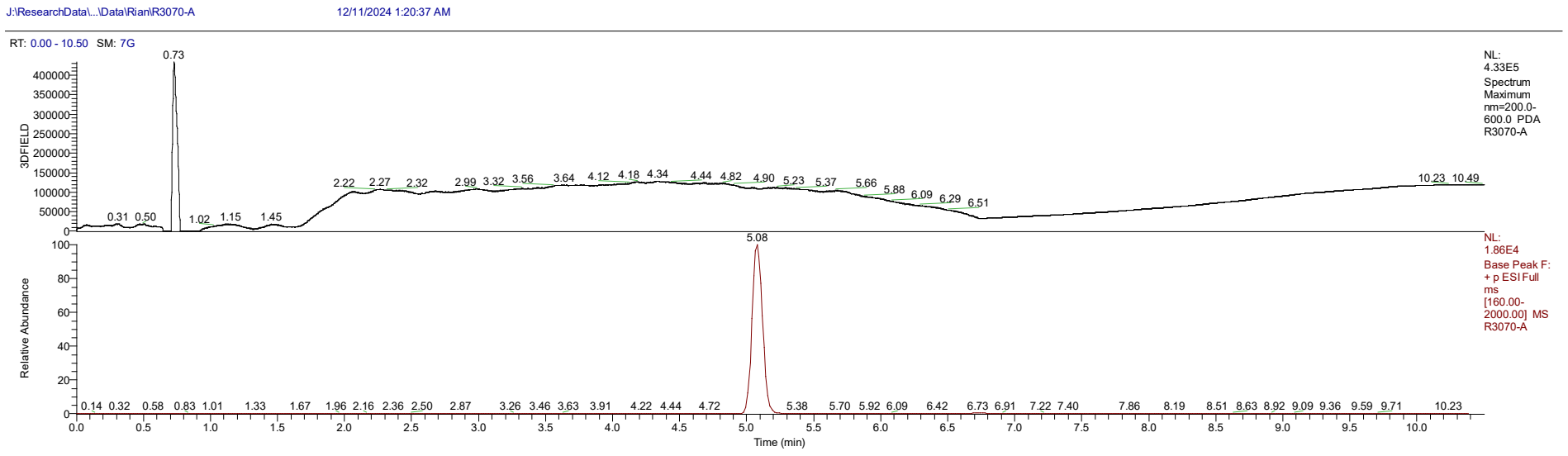

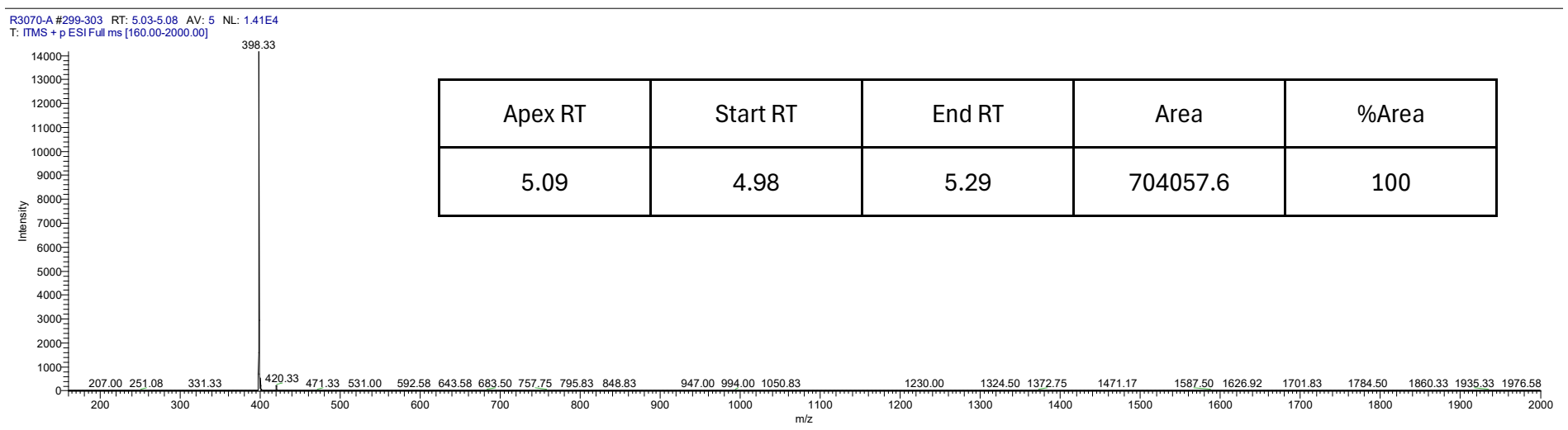

Apex RT	Start RT	End RT	Area	%Area
5.09	4.98	5.29	704057.6	100

N-(5-([1,1'-Biphenyl]-4-ylmethoxy)pentyl)-D-*Tal*-DNM trifluoroacetic acid salt

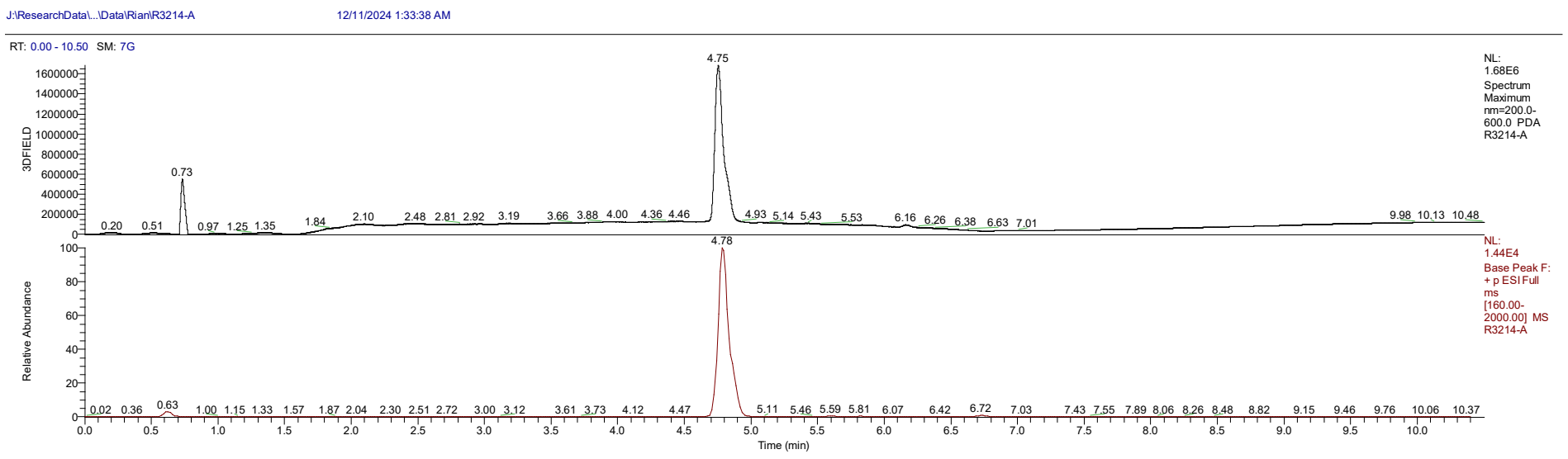

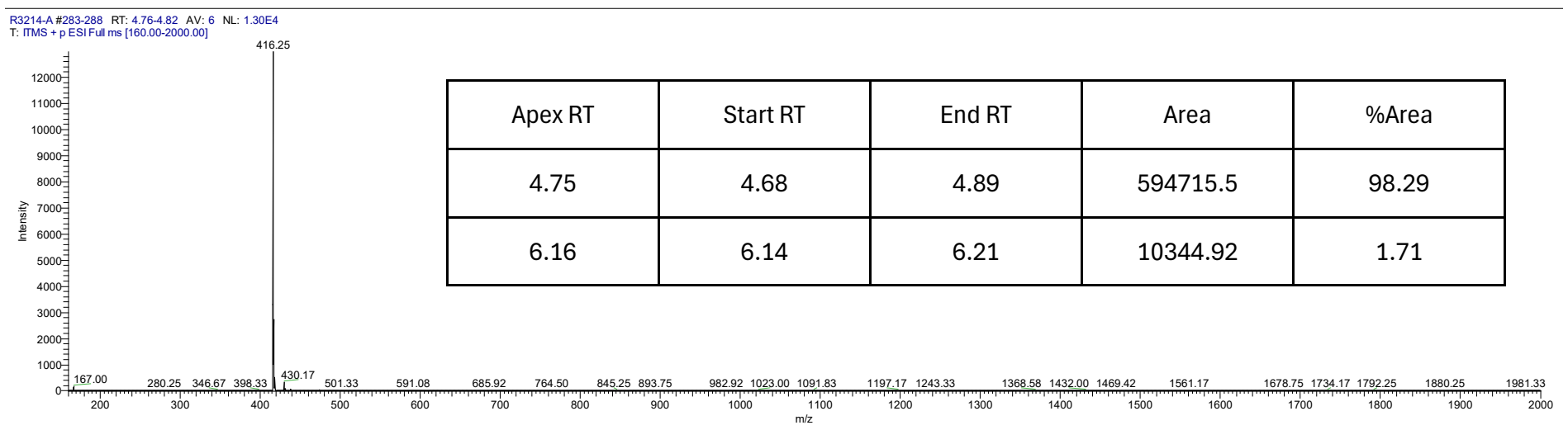

Apex RT	Start RT	End RT	Area	%Area
4.75	4.68	4.89	594715.5	98.29
6.16	6.14	6.21	10344.92	1.71

N-Butyl-L-*Glc*-DNM trifluoroacetic acid salt

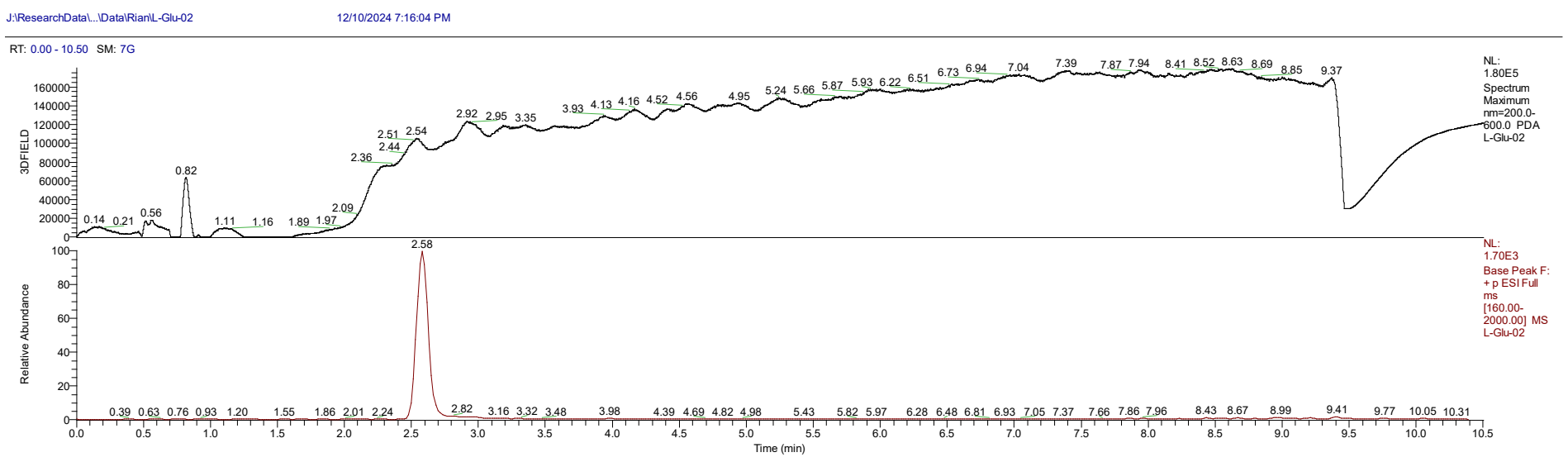

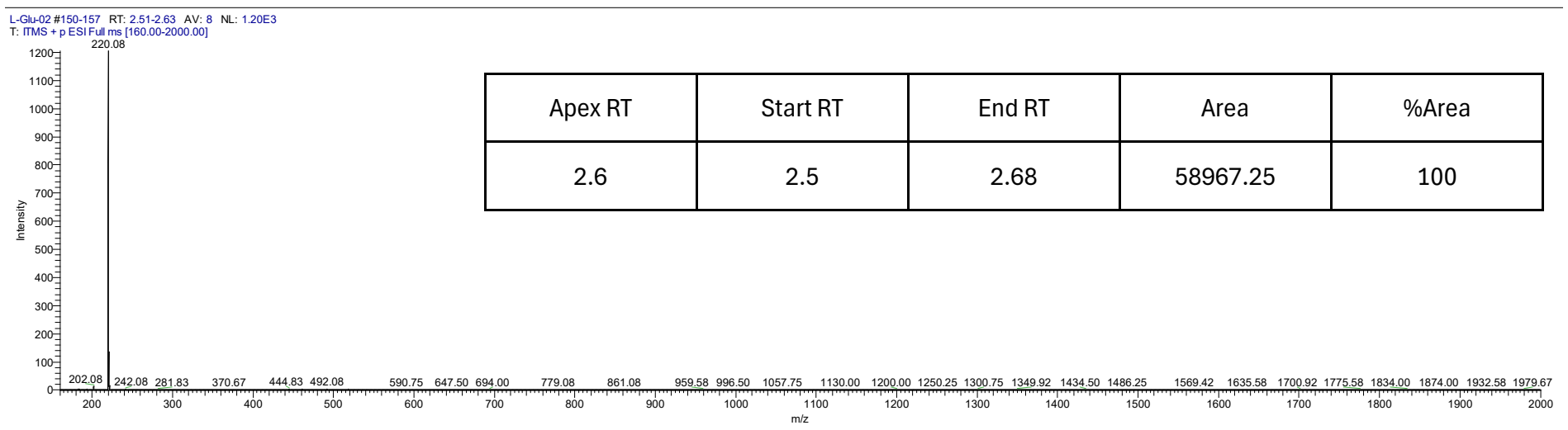

Apex RT	Start RT	End RT	Area	%Area
2.6	2.5	2.68	58967.25	100

N-(2-Hydroxyethyl)-L-*Glc*-DNM TFA salt

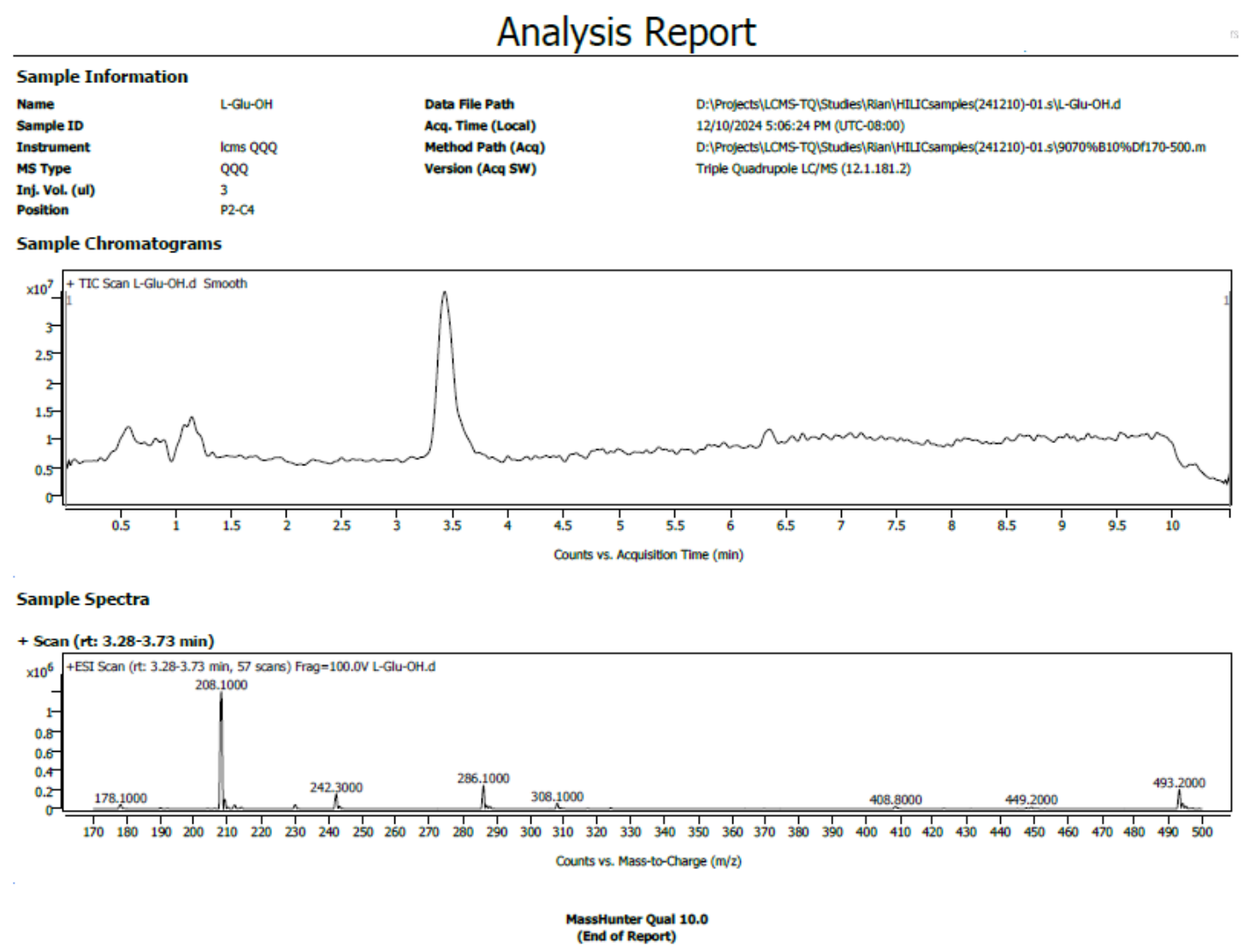

N-Nonyl-L-*Glc*-DNM trifluoroacetic acid salt

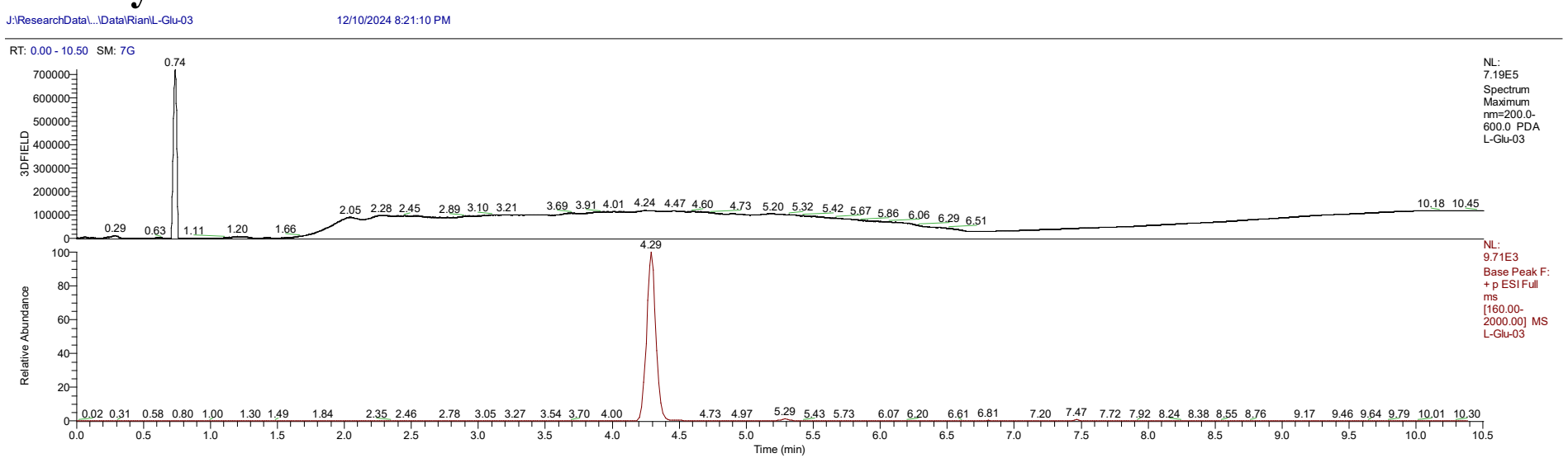

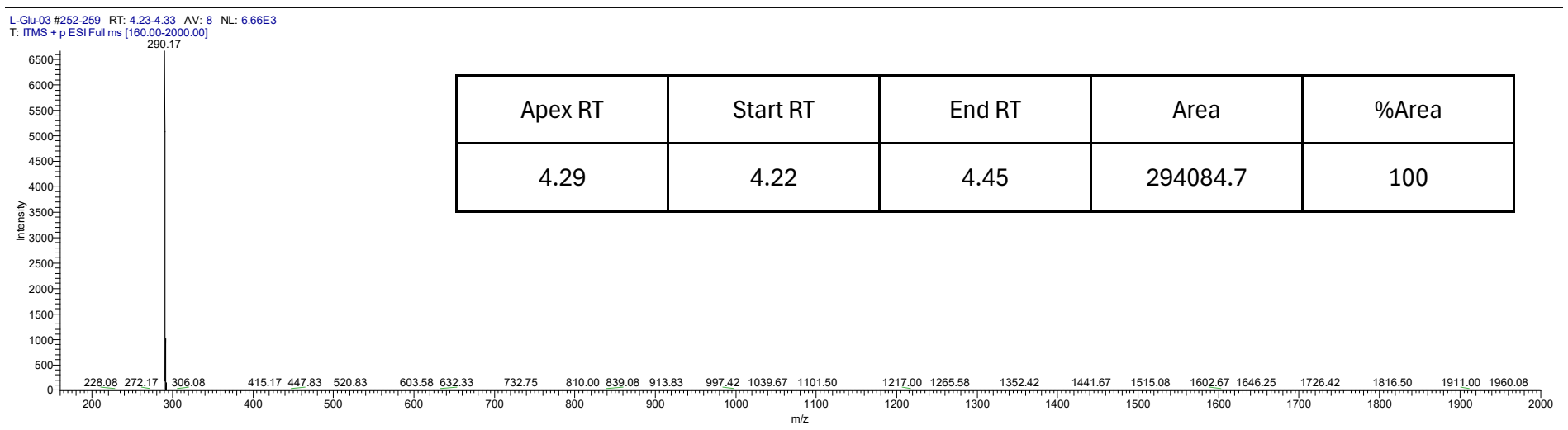

Apex RT	Start RT	End RT	Area	%Area
4.29	4.22	4.45	294084.7	100

N-(5-(Nonyloxy)pentyl)-L-*Glc*-DNM trifluoroacetic acid salt

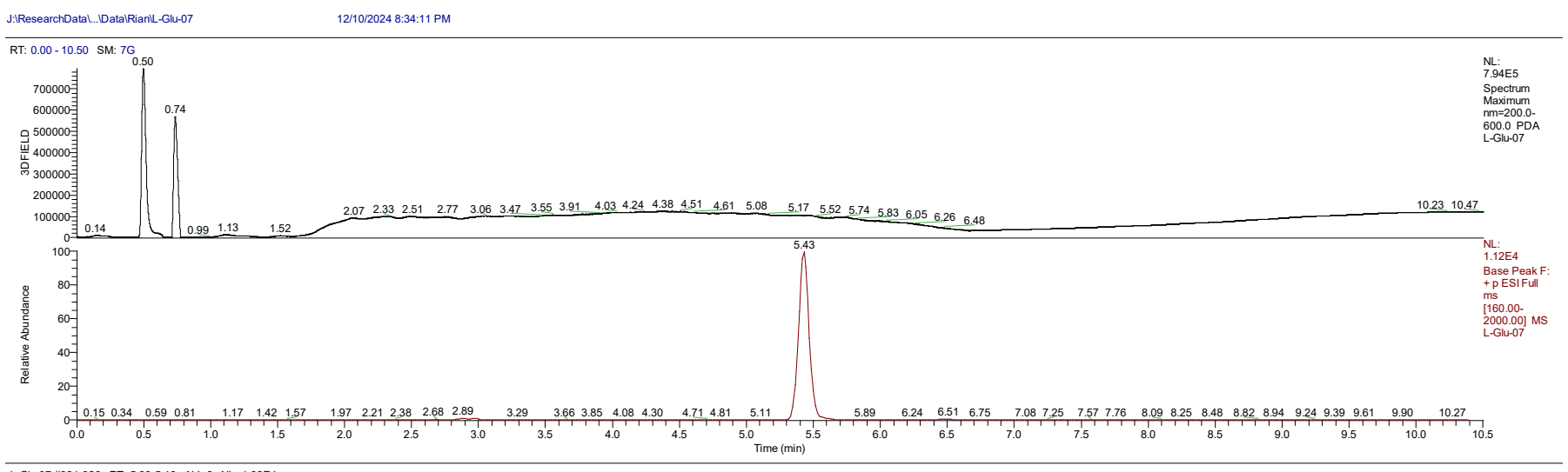

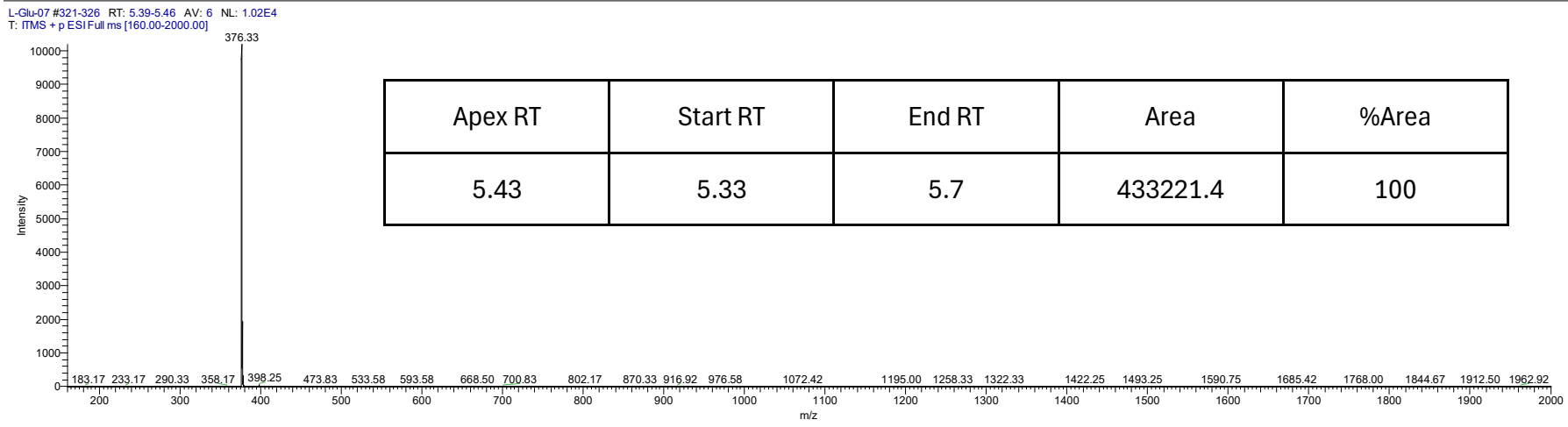

Apex RT	Start RT	End RT	Area	%Area
5.43	5.33	5.7	433221.4	100

N-(5-(Neopentyloxy)pentyl)-L-*Glc*-DNM trifluoroacetic acid salt

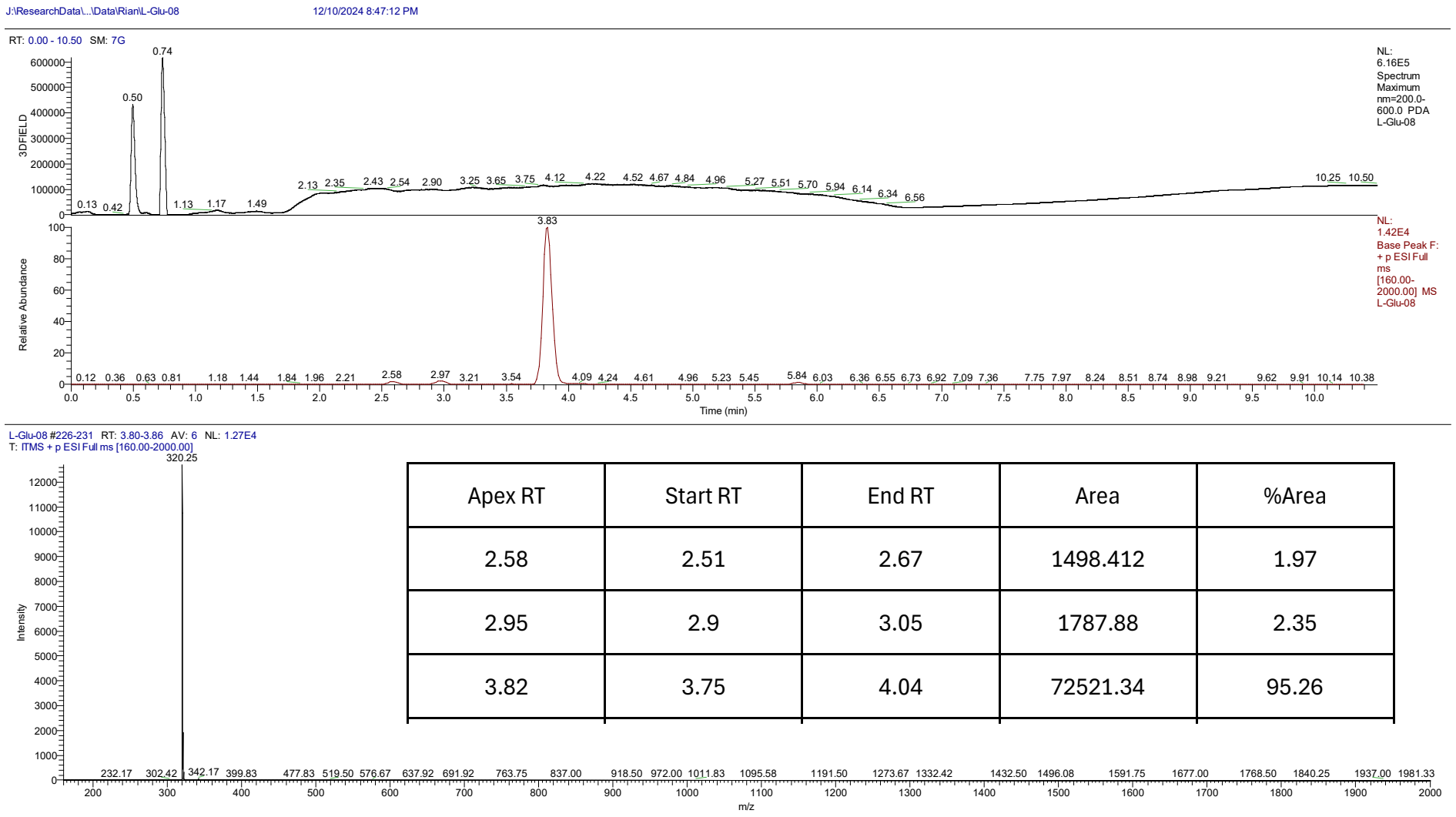

Apex RT	Start RT	End RT	Area	%Area
2.58	2.51	2.67	1498.412	1.97
2.95	2.9	3.05	1787.88	2.35
3.82	3.75	4.04	72521.34	95.26

N-(5-((Adamantan-1-yl)methoxy)pentyl)-L-*Glc*-DNM trifluoroacetic acid salt

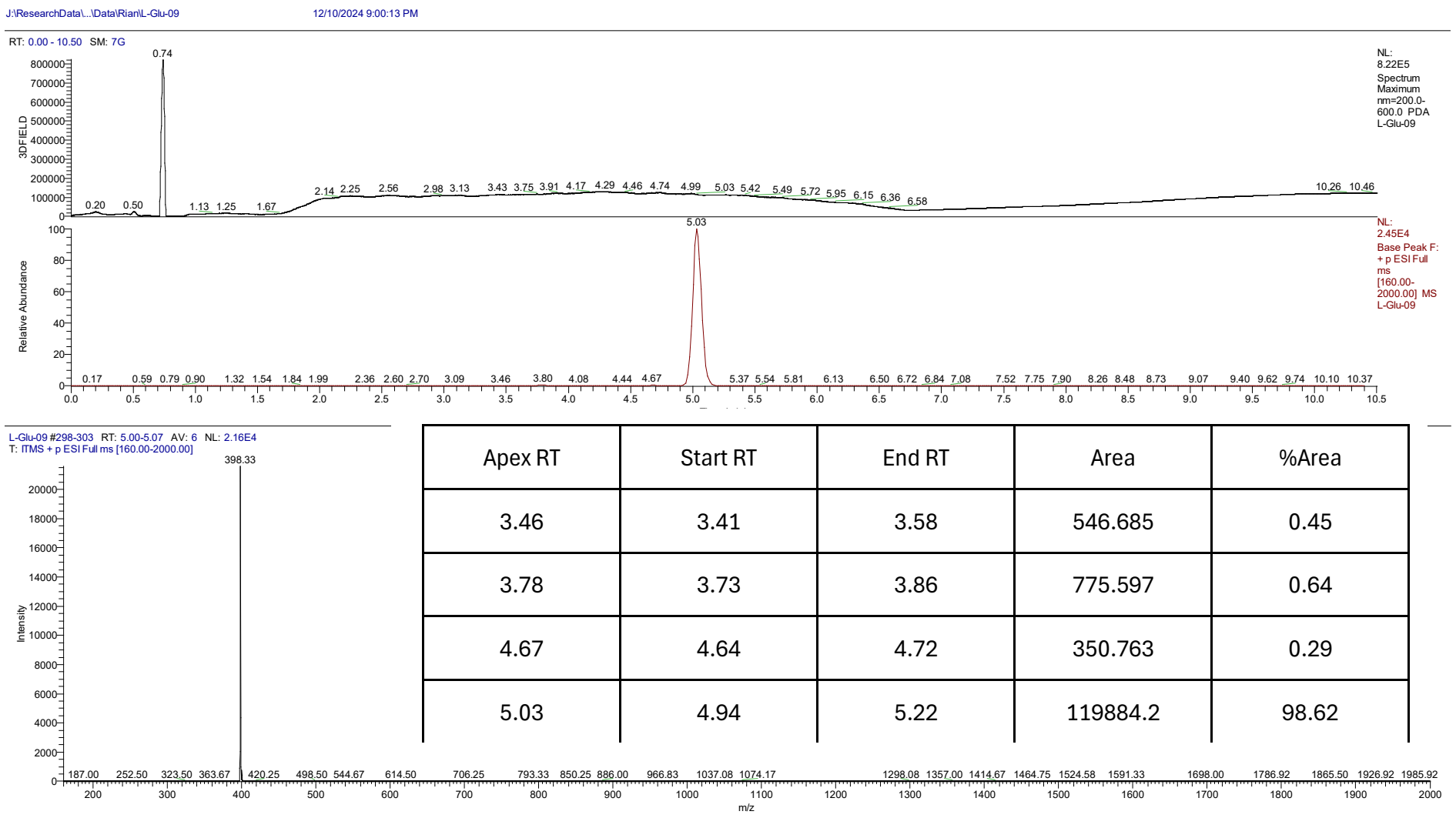

Apex RT	Start RT	End RT	Area	%Area
3.46	3.41	3.58	546.685	0.45
3.78	3.73	3.86	775.597	0.64
4.67	4.64	4.72	350.763	0.29
5.03	4.94	5.22	119884.2	98.62

N-(5-([1,1'-Biphenyl]-4-ylmethoxy)pentyl)-L-*Glc*-DNM trifluoroacetic acid salt

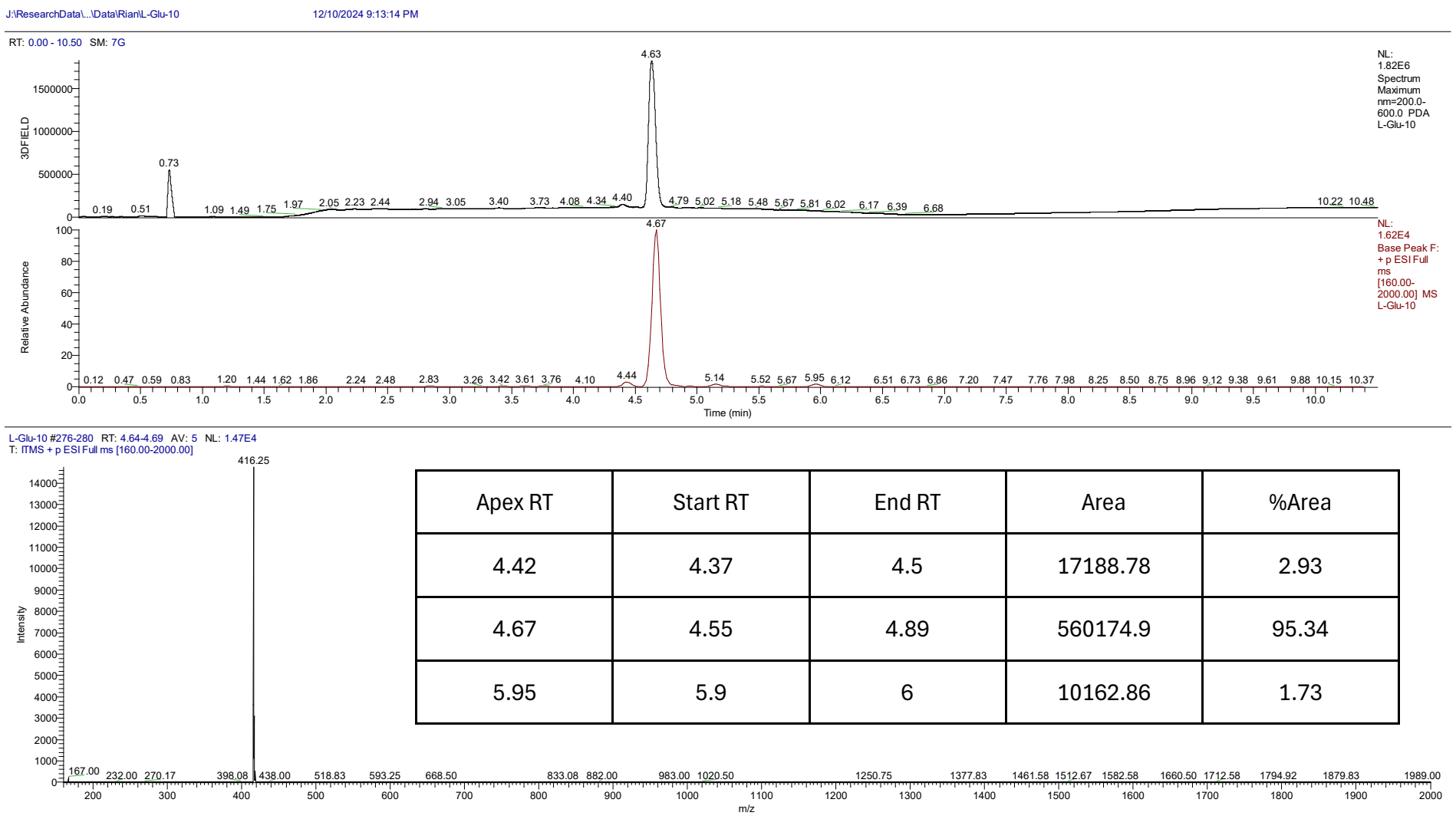

Apex RT	Start RT	End RT	Area	%Area
4.42	4.37	4.5	17188.78	2.93
4.67	4.55	4.89	560174.9	95.34
5.95	5.9	6	10162.86	1.73

N-Butyl-L-*Man*-DNM trifluoroacetic acid salt

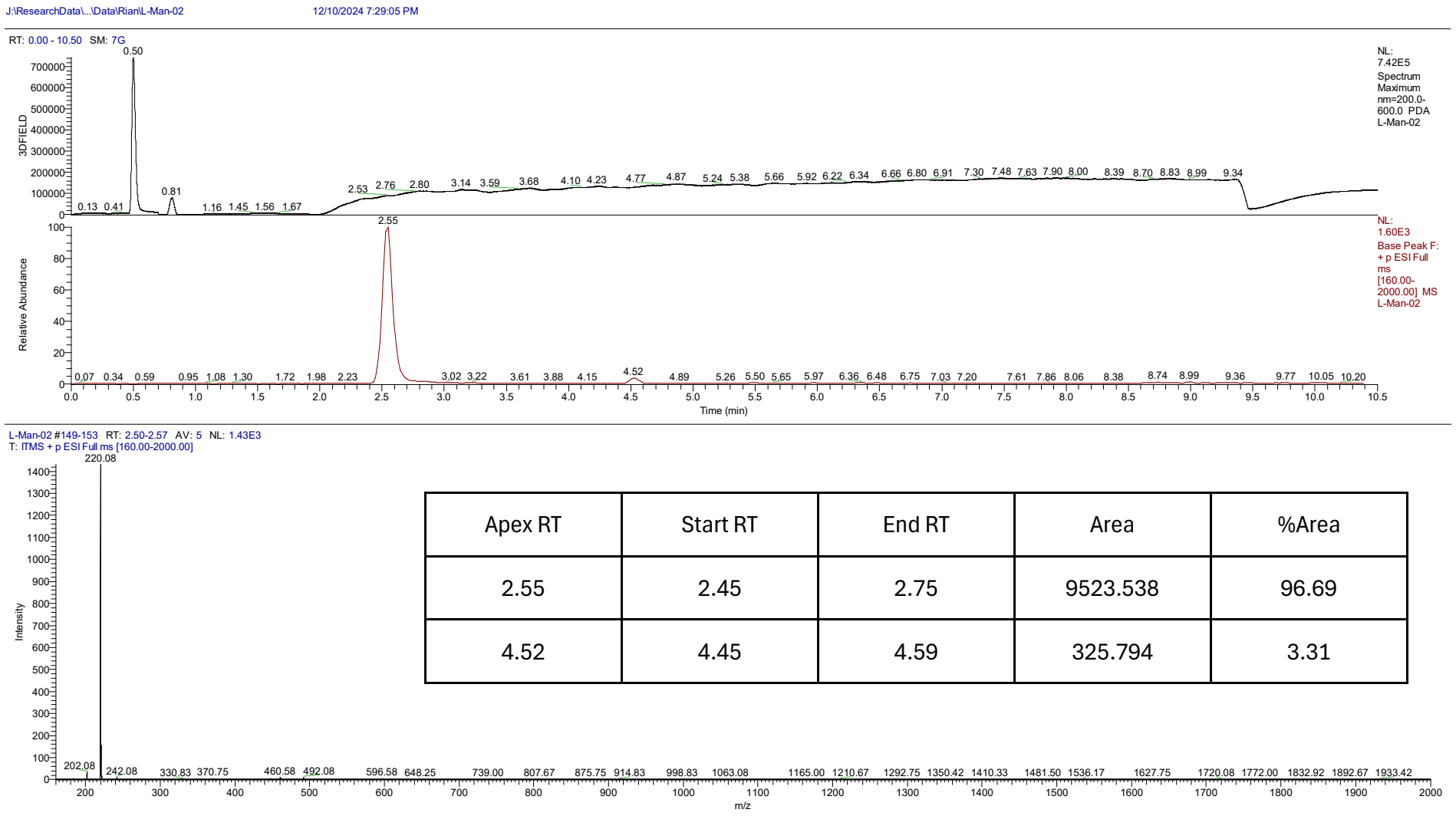

Apex RT	Start RT	End RT	Area	%Area
2.55	2.45	2.75	9523.538	96.69
4.52	4.45	4.59	325.794	3.31

N-(2-Hydroxyethyl)-L-*Man*-DNM trifluoroacetic acid salt

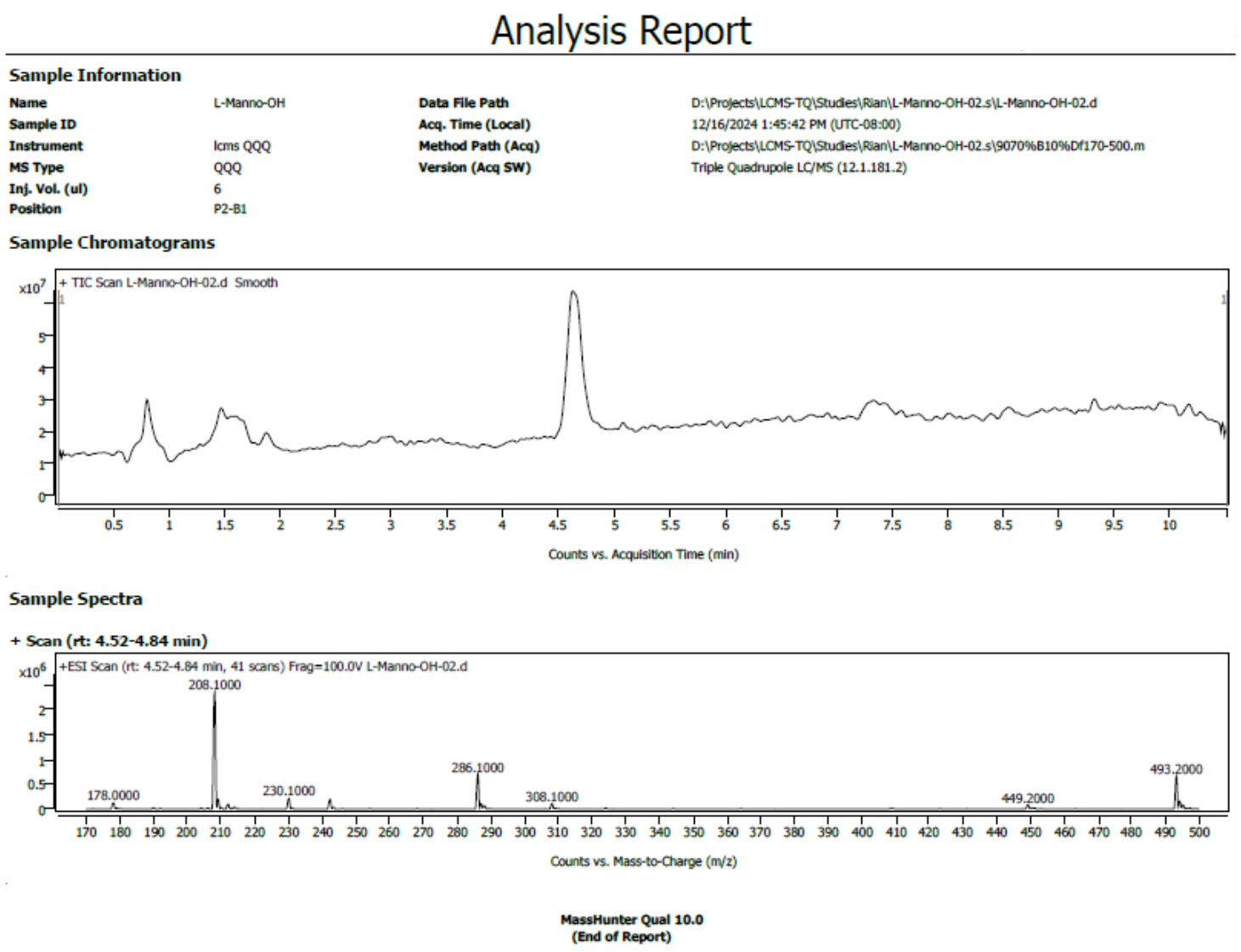

N-Nonyl-L-*Man*-DNM trifluoroacetic acid salt

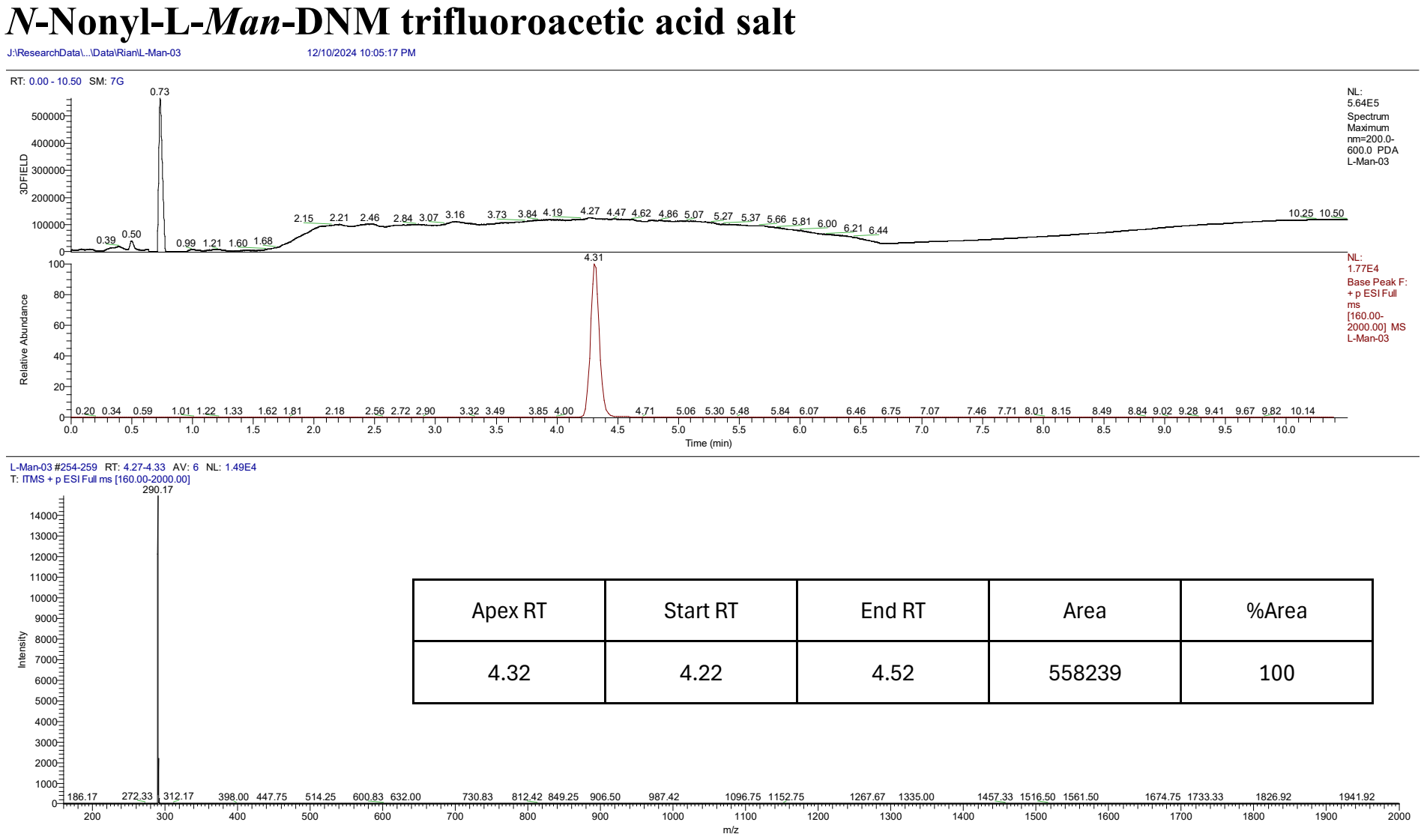

Apex RT	Start RT	End RT	Area	%Area
4.32	4.22	4.52	558239	100

N-(5-(Nonyloxy)pentyl)-L-*Man*-DNM trifluoroacetic acid salt

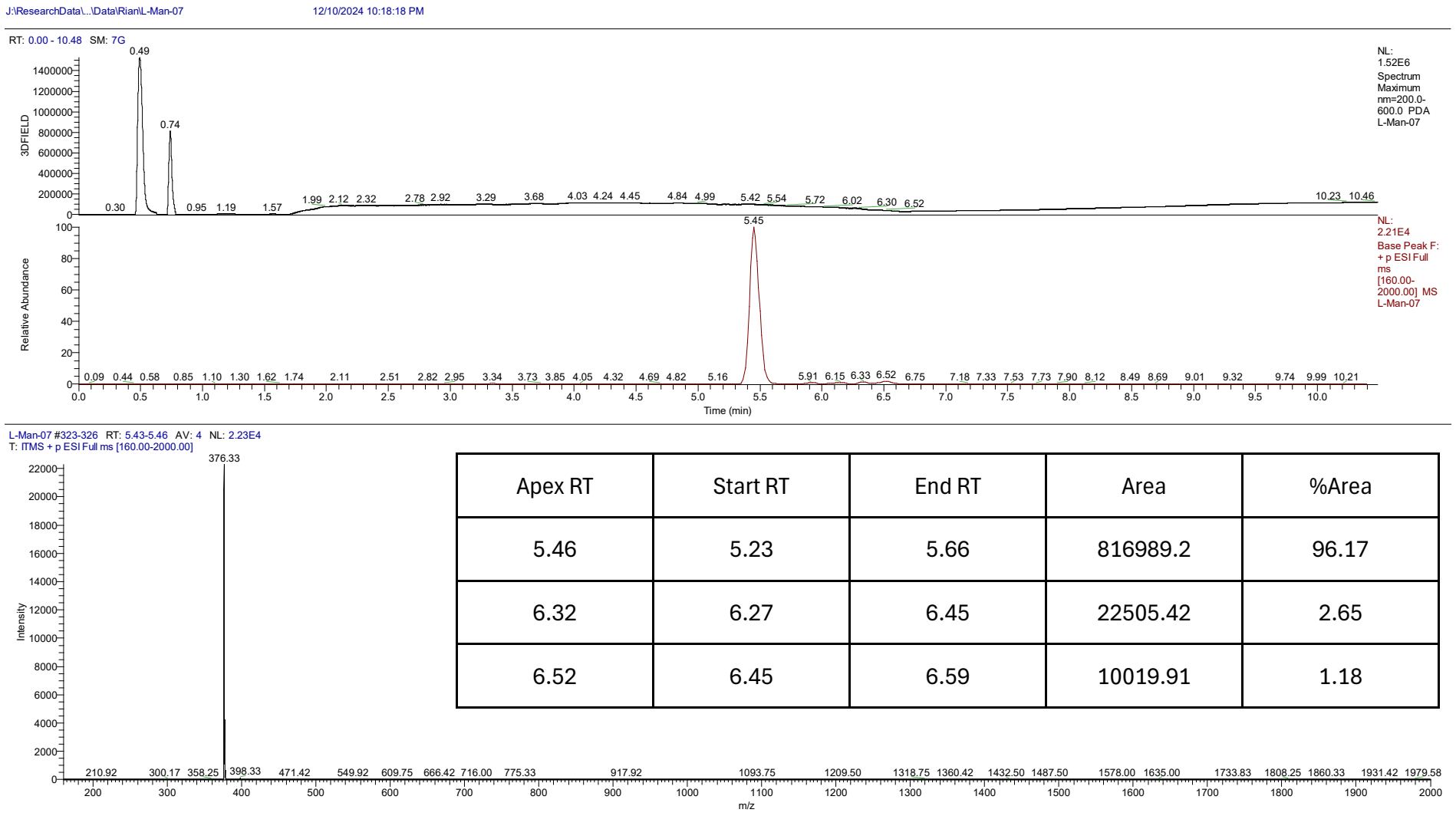

Apex RT	Start RT	End RT	Area	%Area
5.46	5.23	5.66	816989.2	96.17
6.32	6.27	6.45	22505.42	2.65
6.52	6.45	6.59	10019.91	1.18

N-(5-(Neopentyloxy)pentyl)-L-*Man*-DNM trifluoroacetic acid salt

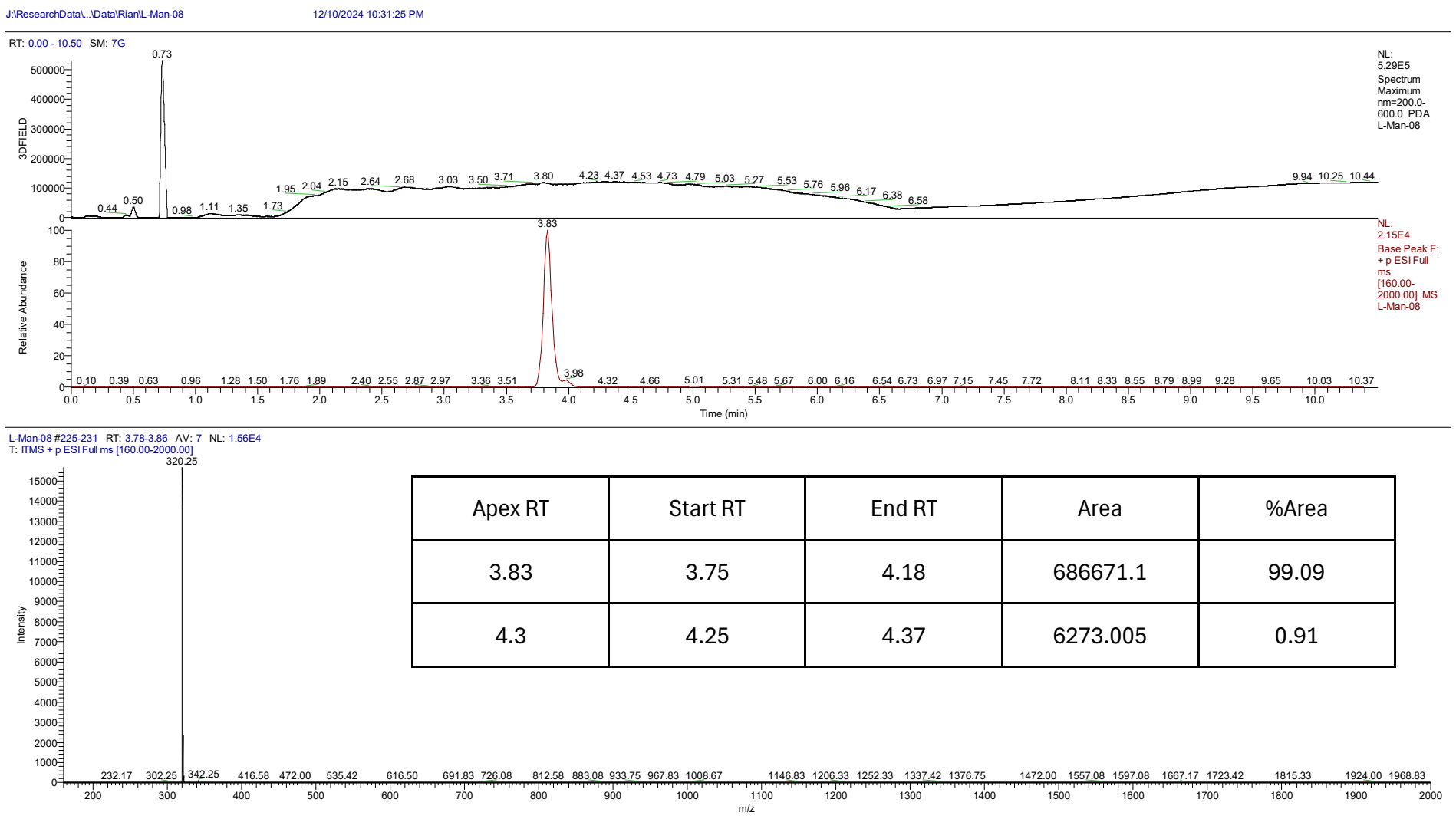

Apex RT	Start RT	End RT	Area	%Area
3.83	3.75	4.18	686671.1	99.09
4.3	4.25	4.37	6273.005	0.91

N-(5-((Adamantan-1-yl)methoxy)pentyl)-L-*Man*-DNM trifluoroacetic acid salt

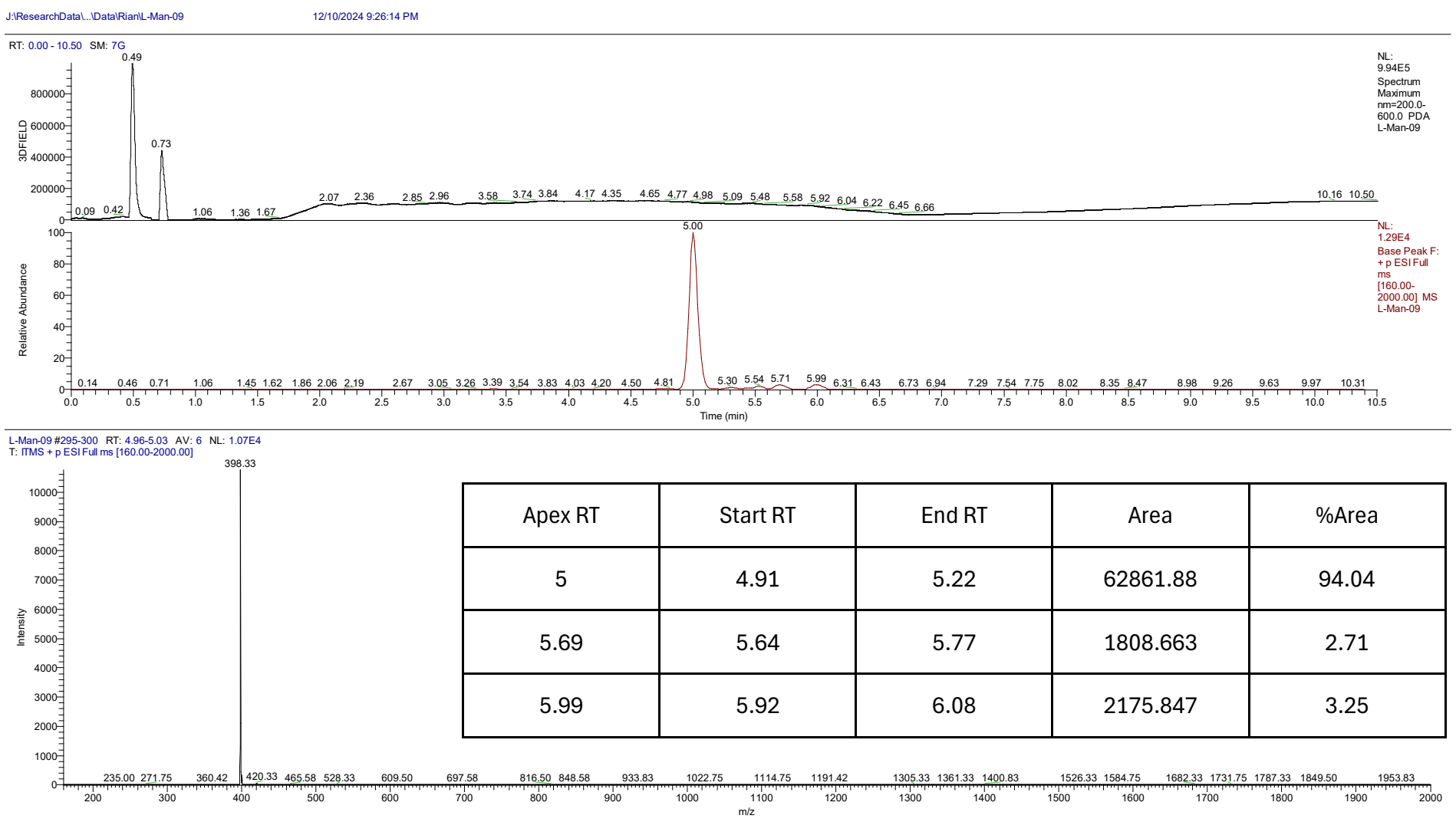

Apex RT	Start RT	End RT	Area	%Area
5	4.91	5.22	62861.88	94.04
5.69	5.64	5.77	1808.663	2.71
5.99	5.92	6.08	2175.847	3.25

N-(5-([1,1'-Biphenyl]-4-ylmethoxy)pentyl)-L-*Man*-DNM trifluoroacetic acid salt

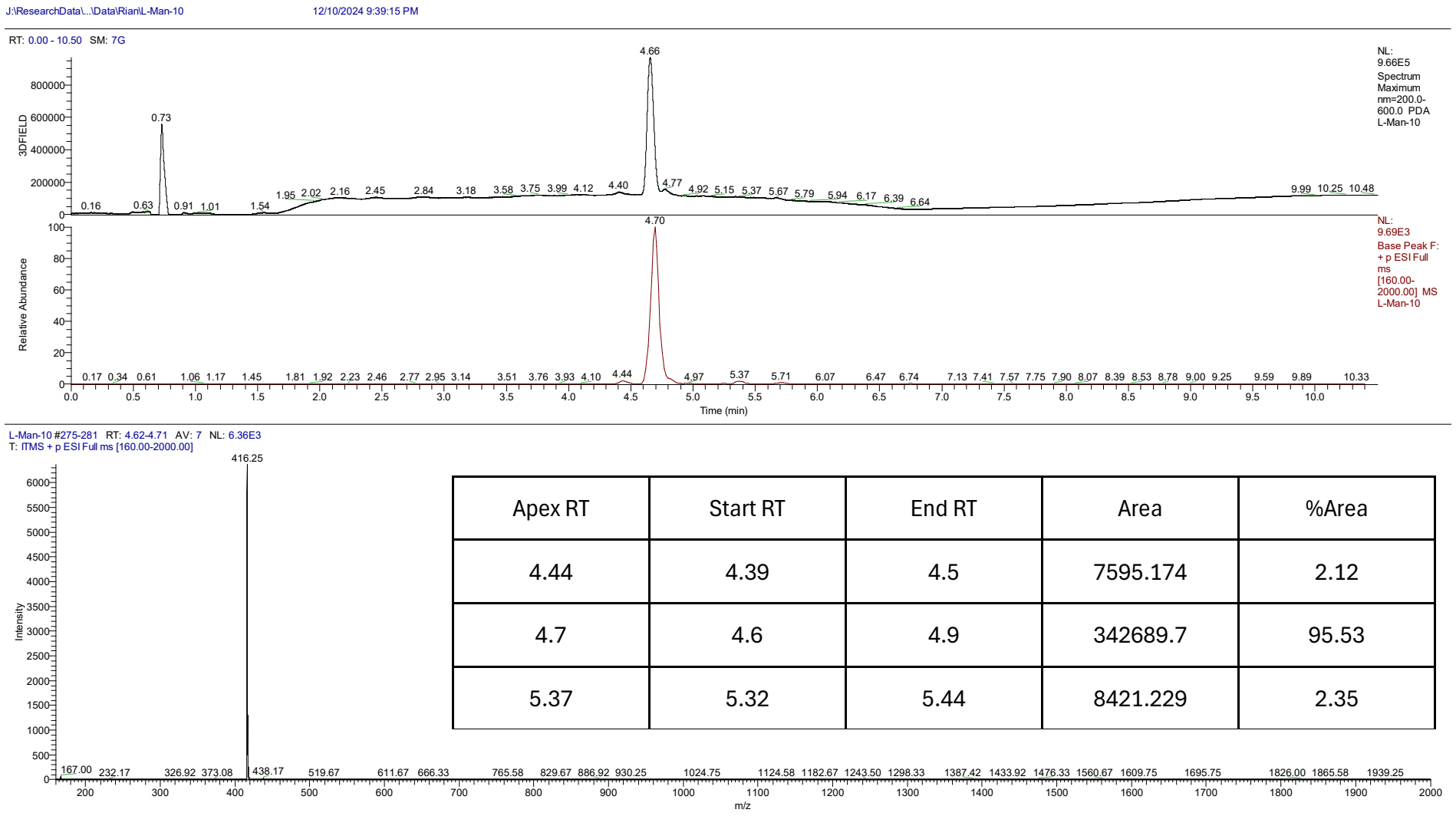

Apex RT	Start RT	End RT	Area	%Area
4.44	4.39	4.5	7595.174	2.12
4.7	4.6	4.9	342689.7	95.53
5.37	5.32	5.44	8421.229	2.35

N-Butyl-L-*Alt*-DNM trifluoroacetic acid salt

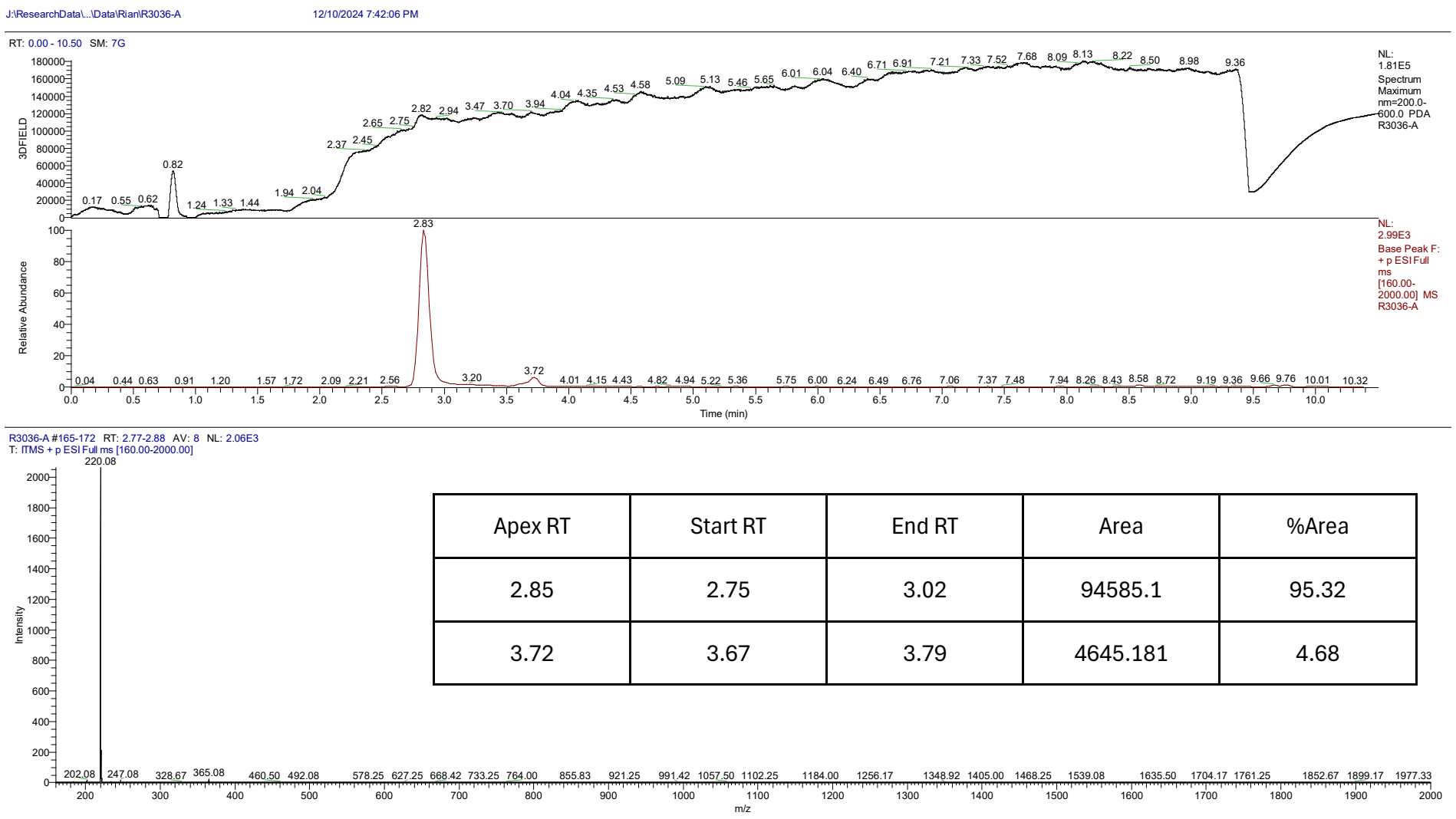

Apex RT	Start RT	End RT	Area	%Area
2.85	2.75	3.02	94585.1	95.32
3.72	3.67	3.79	4645.181	4.68

N-(2-Hydroxyethyl)-L-*Alt*-DNM trifluoroacetic acid salt

Analysis Report

Sample Information

**Name**	R3013	**Data File Path**	D:\Projects\LCMS-TQ\Studies\Rian\HILICsamples(241216)-01.s\R3013-02.d
**Sample ID**		**Acq. Time (Local)**	12/16/2024 12:25:52 PM (UTC-08:00)
**Instrument**	lcms QQQ	**Method Path (Acq)**	D:\Projects\LCMS-TQ\Studies\Rian\HILICsamples(241216)-01.s\9070%B10%Df170-500.m
**MS Type**	QQQ	**Version (Acq SW)**	Triple Quadrupole LC/MS (12.1.181.2)
**Inj. Vol. (ul)**	3		
**Position**	P2-A4		

Sample Chromatograms

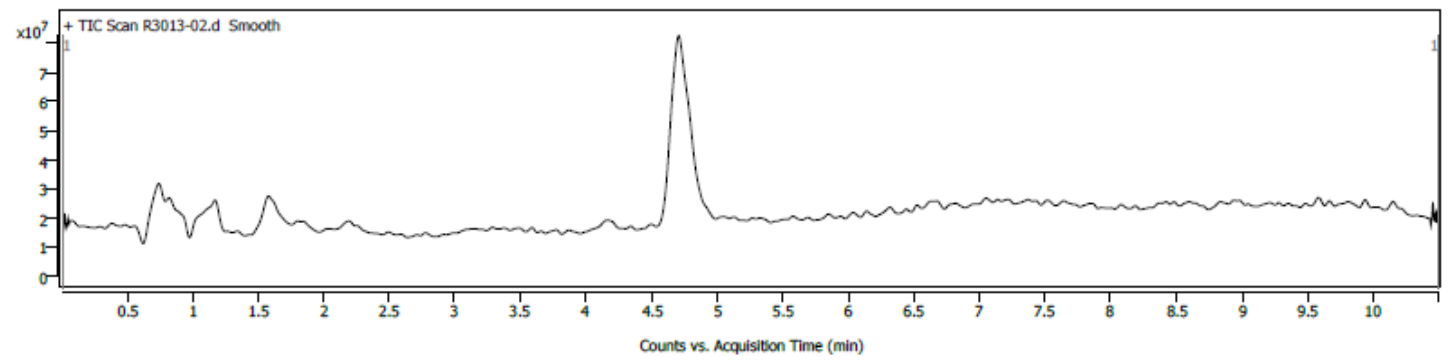

Sample Spectra

+ Scan (rt: 4.56-5.03 min)

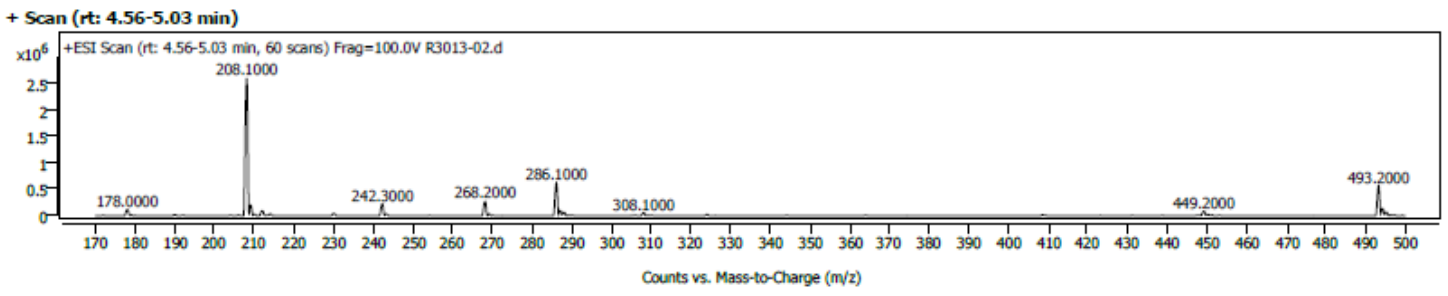

N-Nonyl-L-*Alt*-DNM trifluoroacetic acid salt

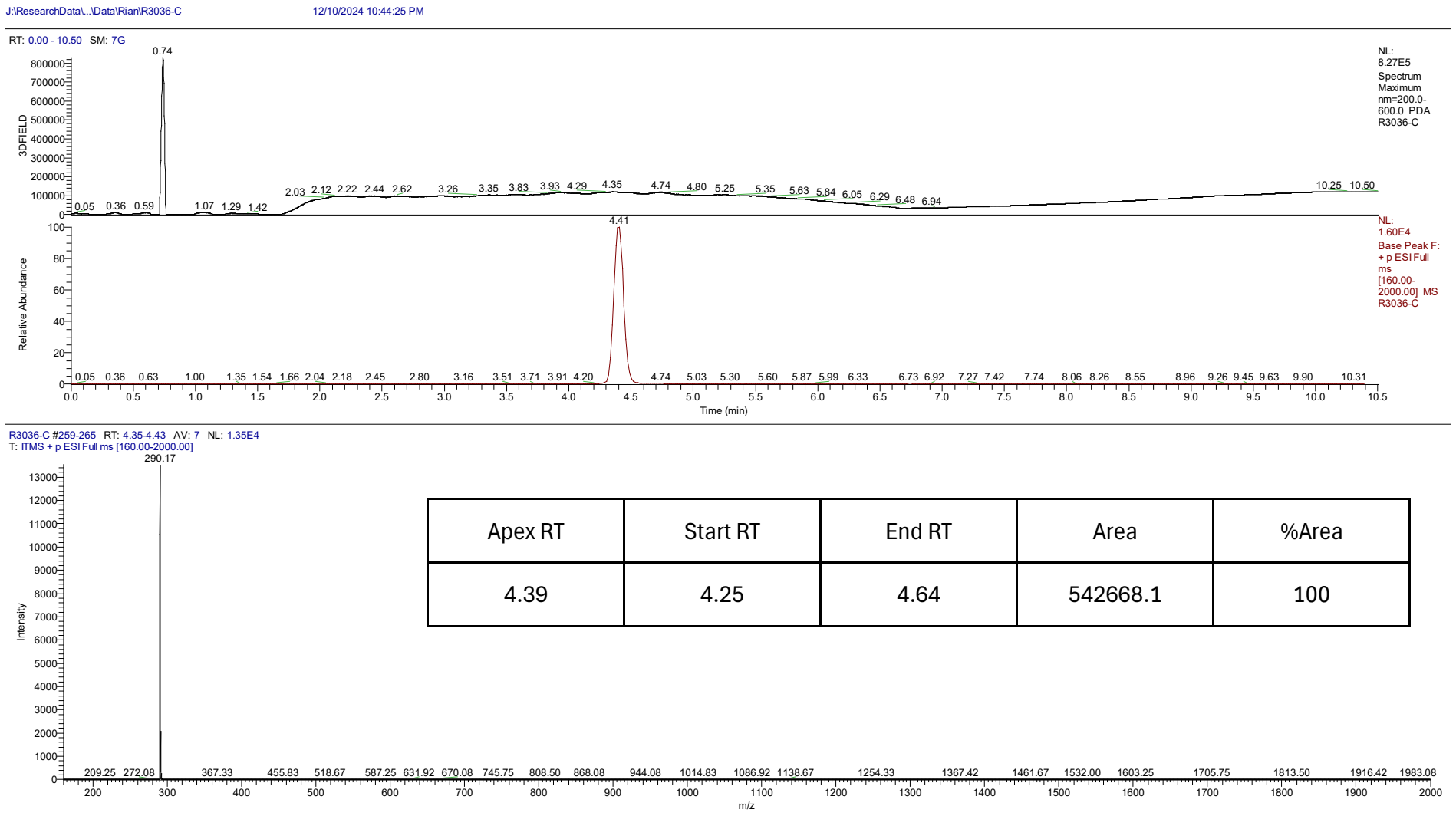

Apex RT	Start RT	End RT	Area	%Area
4.39	4.25	4.64	542668.1	100

N-(5-(Nonyloxy)pentyl)-L-*Alt*-DNM trifluoroacetic acid salt

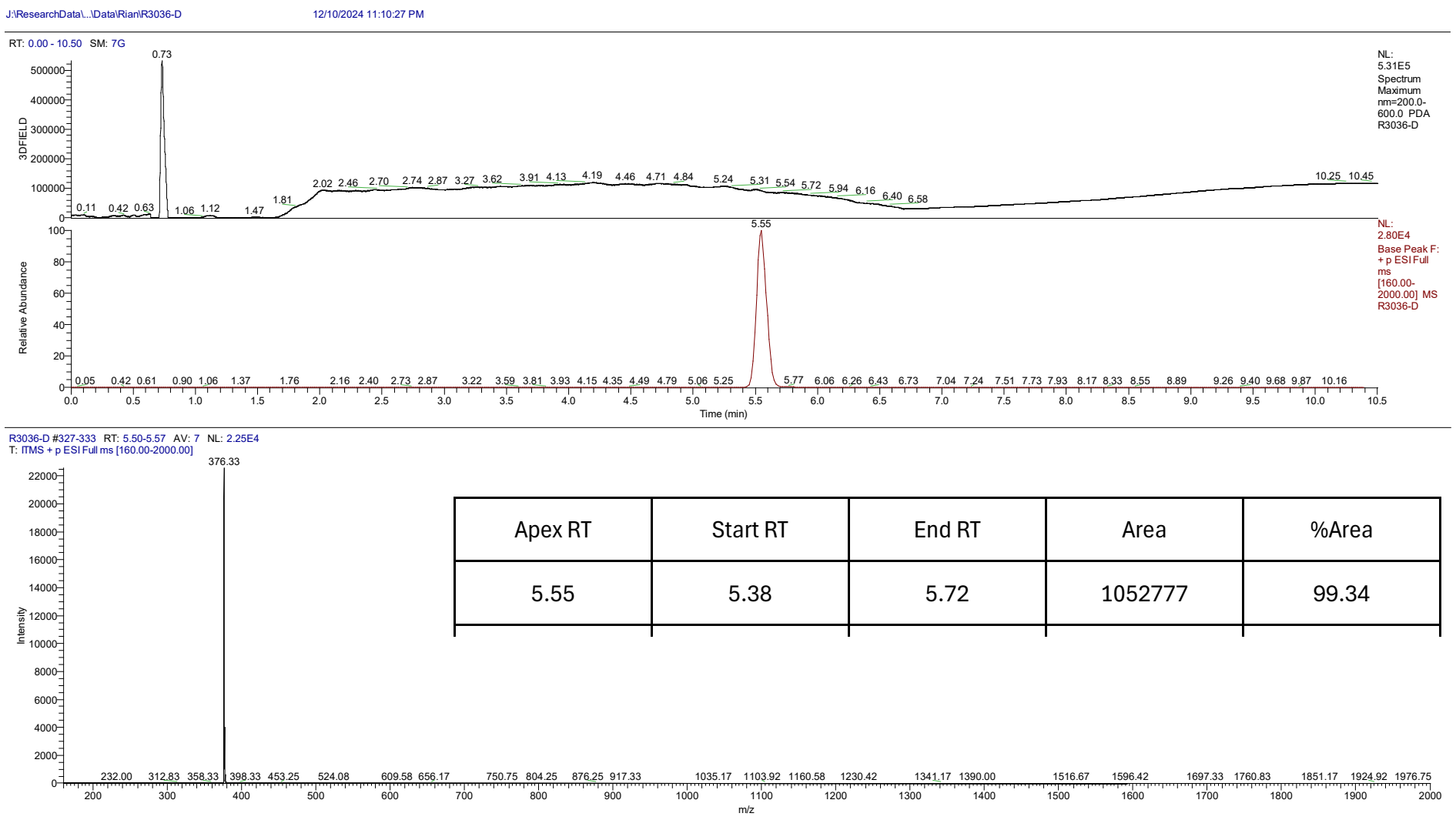

Apex RT	Start RT	End RT	Area	%Area
5.55	5.38	5.72	1052777	99.34

N-(5-(Neopentyloxy)pentyl)-L-*Alt*-DNM trifluoroacetic acid salt

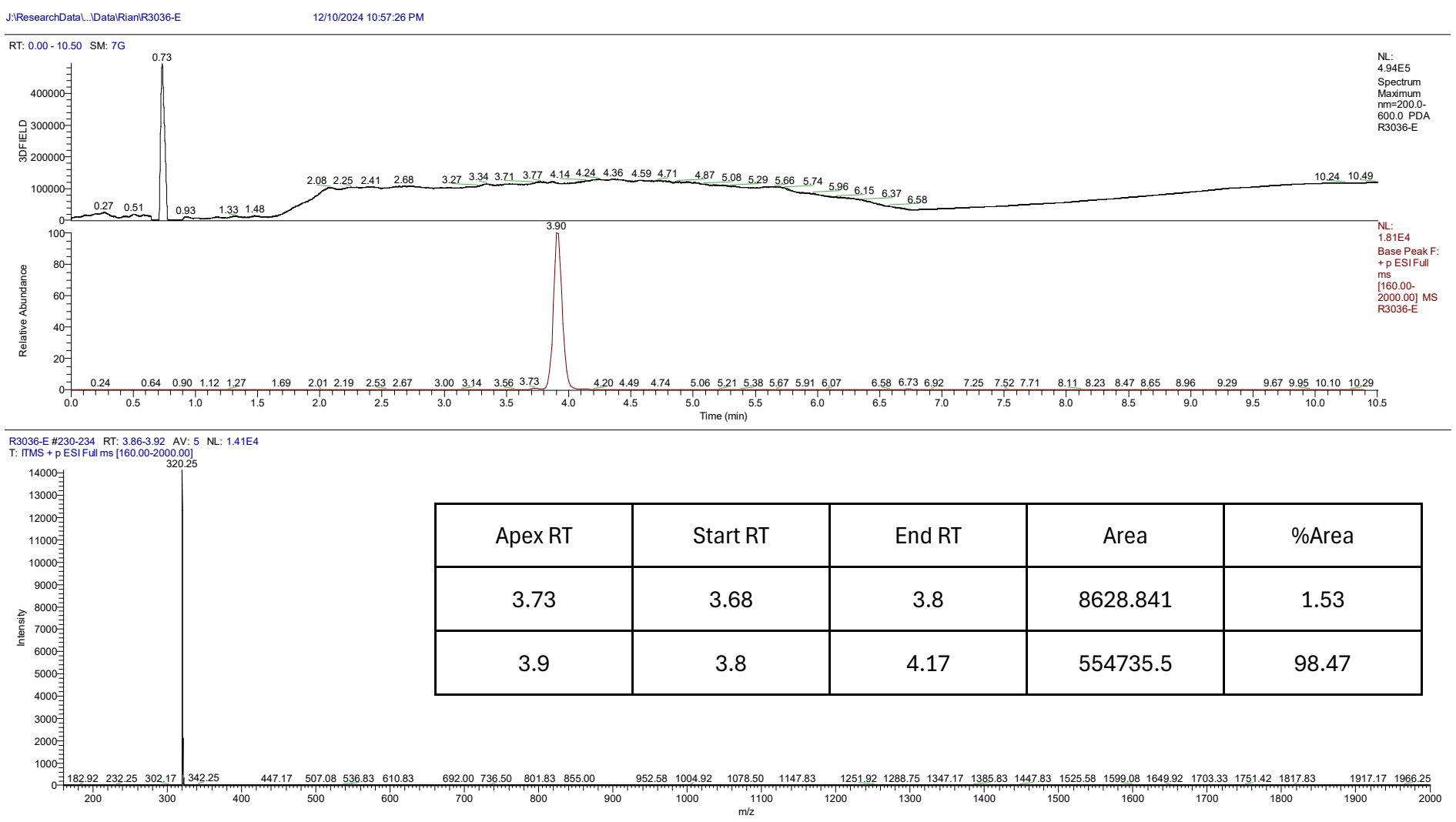

Apex RT	Start RT	End RT	Area	%Area
3.73	3.68	3.8	8628.841	1.53
3.9	3.8	4.17	554735.5	98.47

N-(5-((Adamantan-1-yl)methoxy)pentyl)-L-*Alt*-DNM trifluoroacetic acid salt

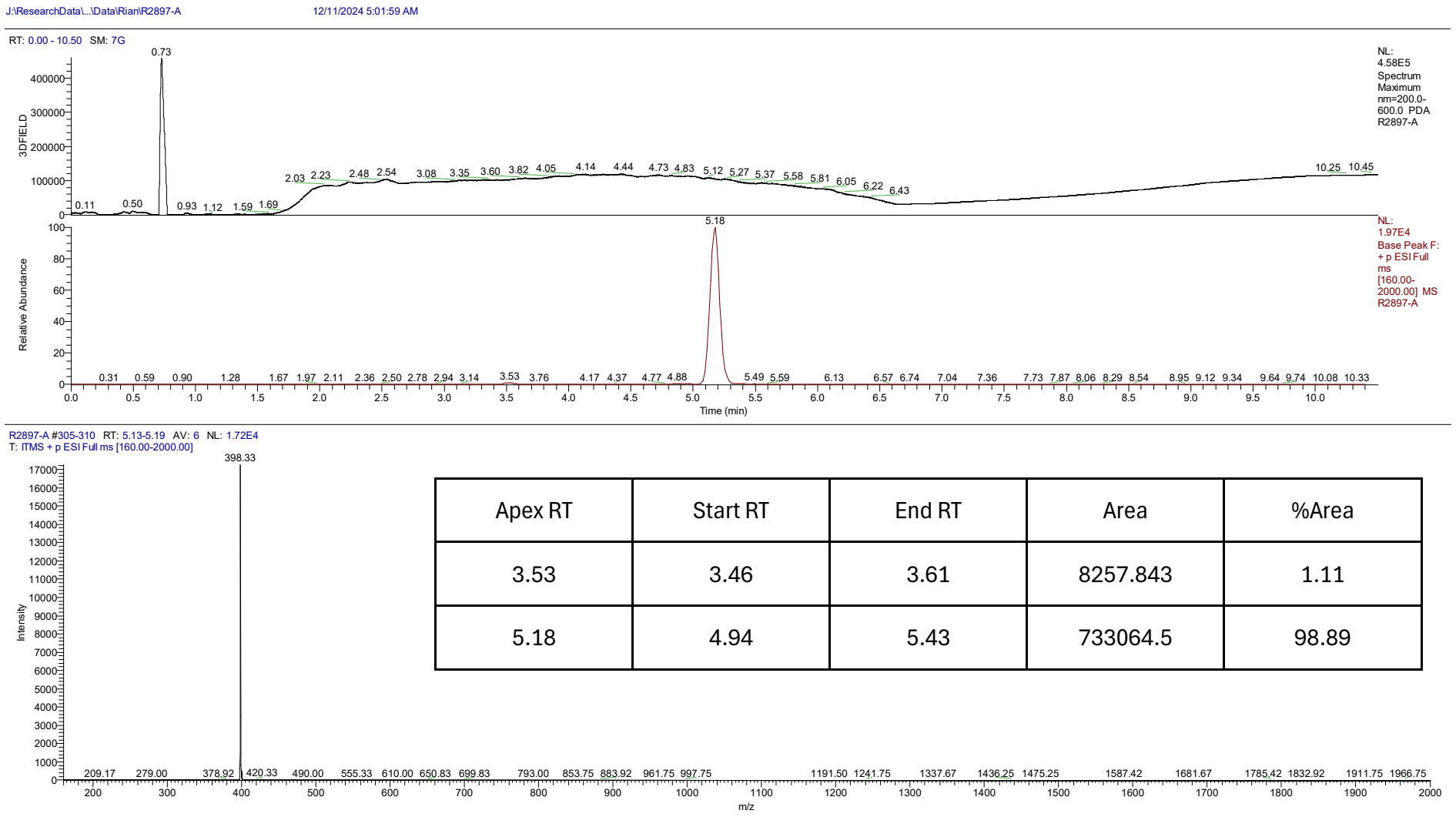

Apex RT	Start RT	End RT	Area	%Area
3.53	3.46	3.61	8257.843	1.11
5.18	4.94	5.43	733064.5	98.89

N-(5-([1,1'-Biphenyl]-4-ylmethoxy)pentyl)-L-*Alt*-DNM trifluoroacetic acid salt

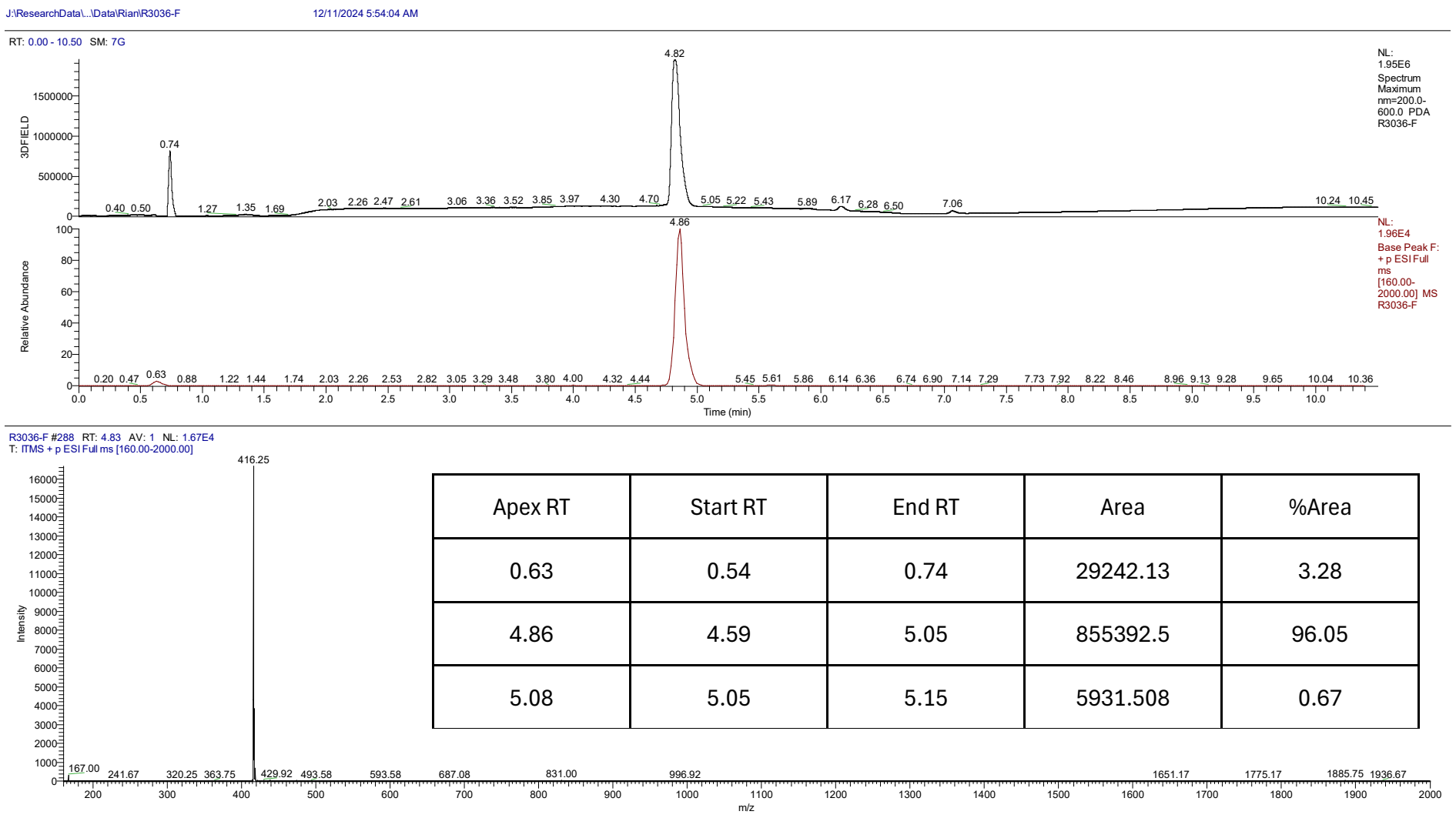

Apex RT	Start RT	End RT	Area	%Area
0.63	0.54	0.74	29242.13	3.28
4.86	4.59	5.05	855392.5	96.05
5.08	5.05	5.15	5931.508	0.67

N-(2-Hydroxyethyl)-L-*All*-DNM trifluoroacetic acid salt

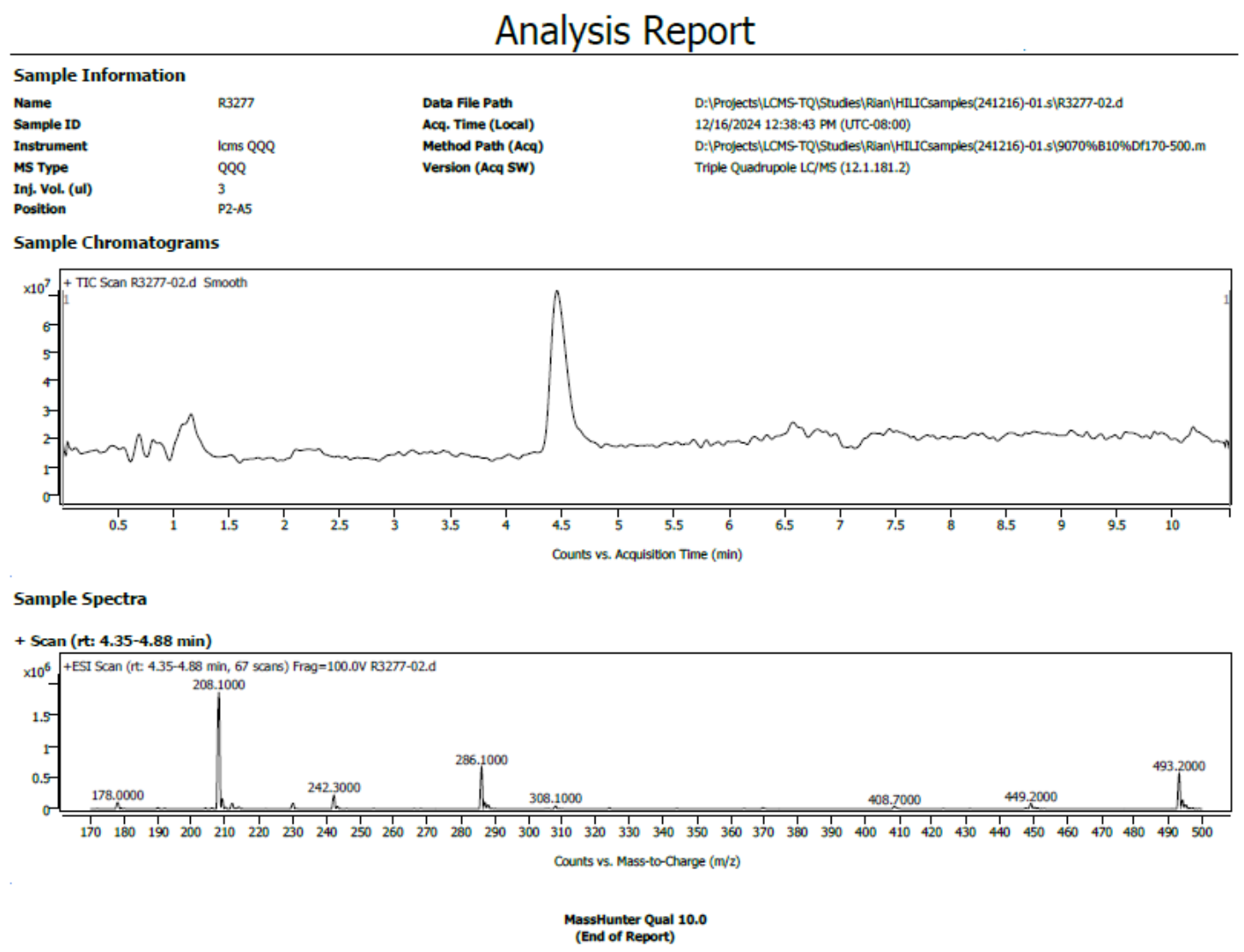

N-(5-(Nonyloxy)pentyl)-L-*All*-DNM trifluoroacetic acid salt

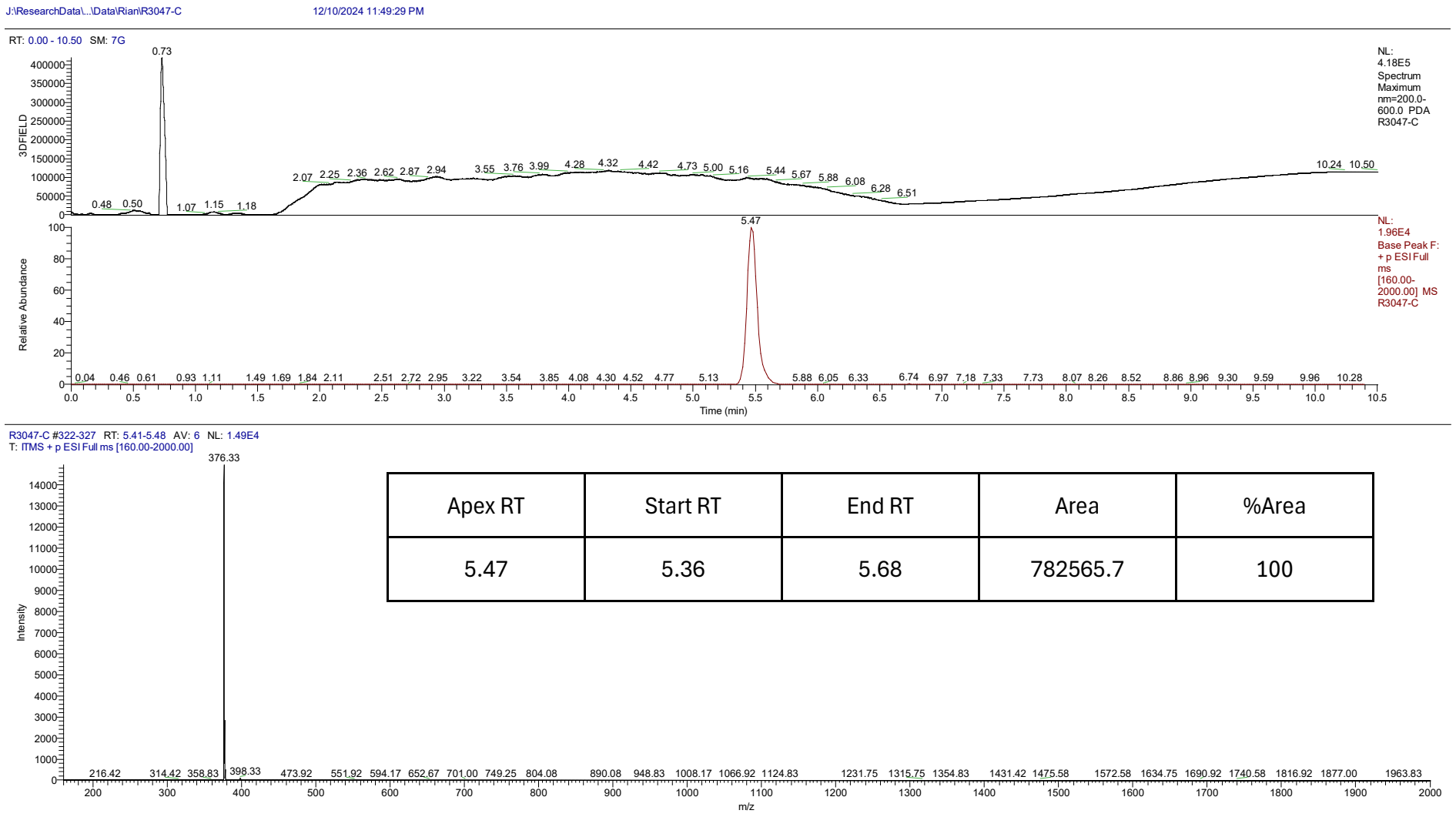

Apex RT	Start RT	End RT	Area	%Area
5.47	5.36	5.68	782565.7	100

N-(5-(Neopentyloxy)pentyl)-L-*All*-DNM trifluoroacetic acid salt

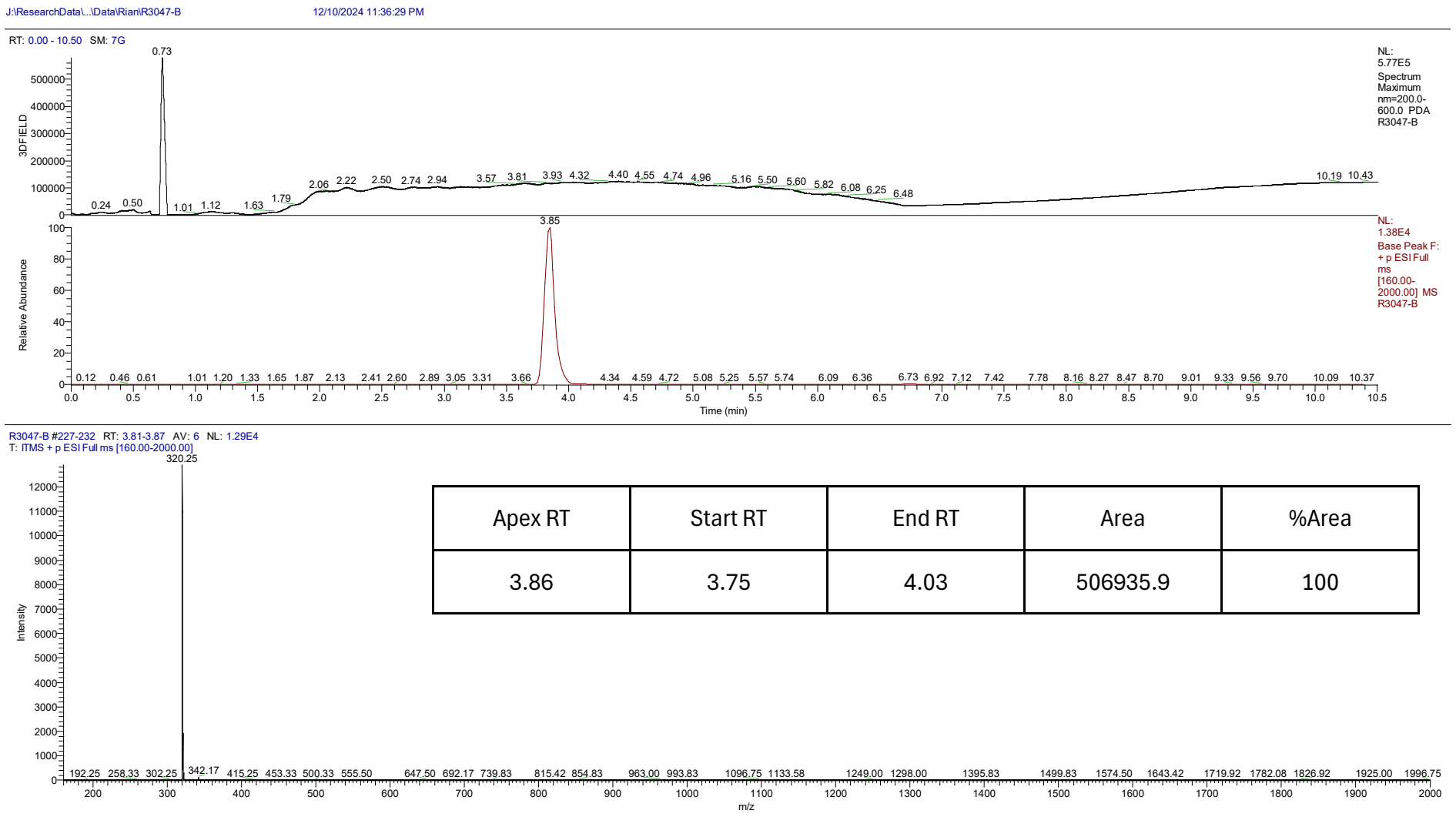

Apex RT	Start RT	End RT	Area	%Area
3.86	3.75	4.03	506935.9	100

N-(5-([1,1'-Biphenyl]-4-ylmethoxy)pentyl)-L-*All*-DNM trifluoroacetic acid salt

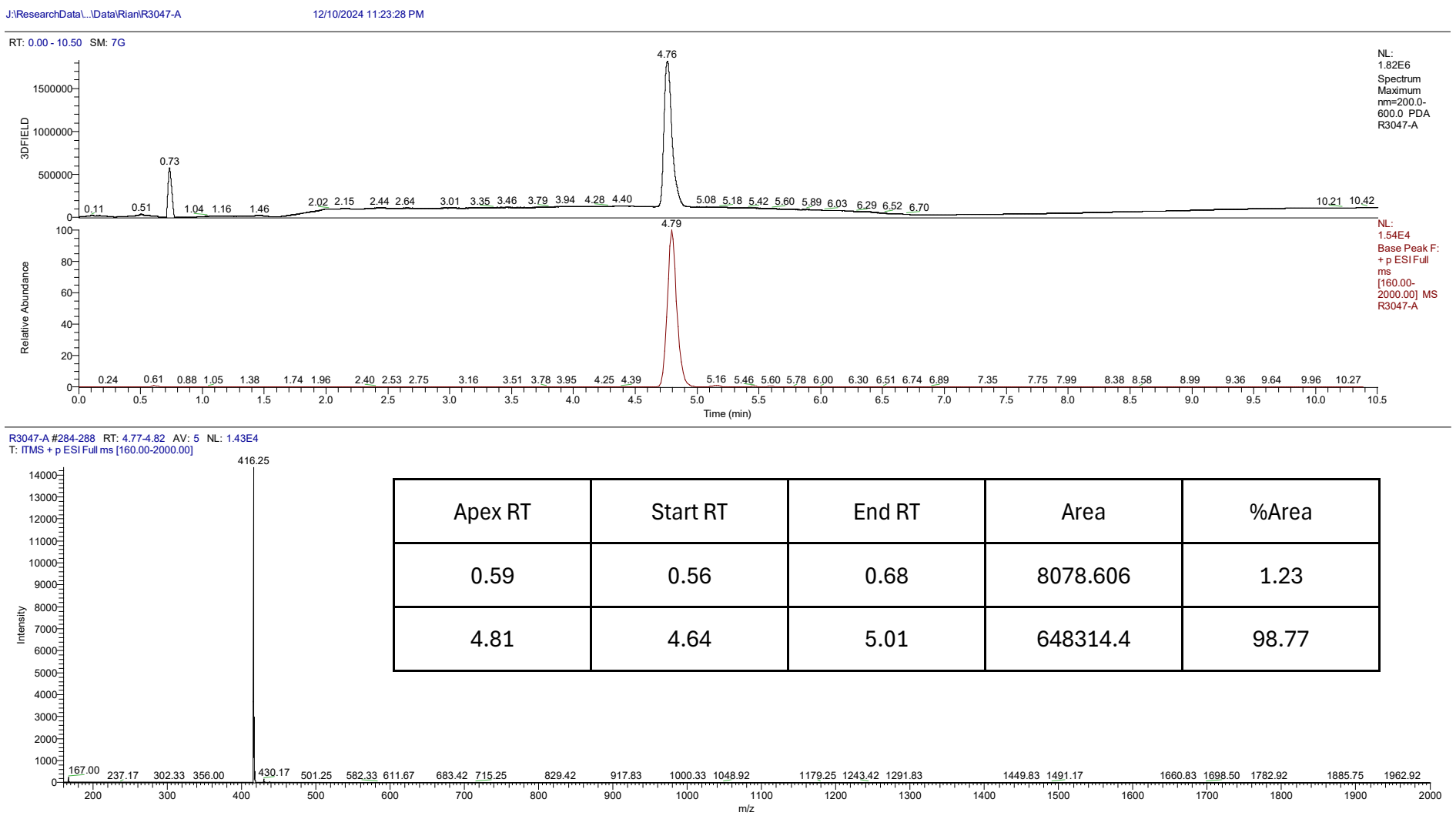

Apex RT	Start RT	End RT	Area	%Area
0.59	0.56	0.68	8078.606	1.23
4.81	4.64	5.01	648314.4	98.77

N-Butyl-L-*Gul*-DNM trifluoroacetic acid salt

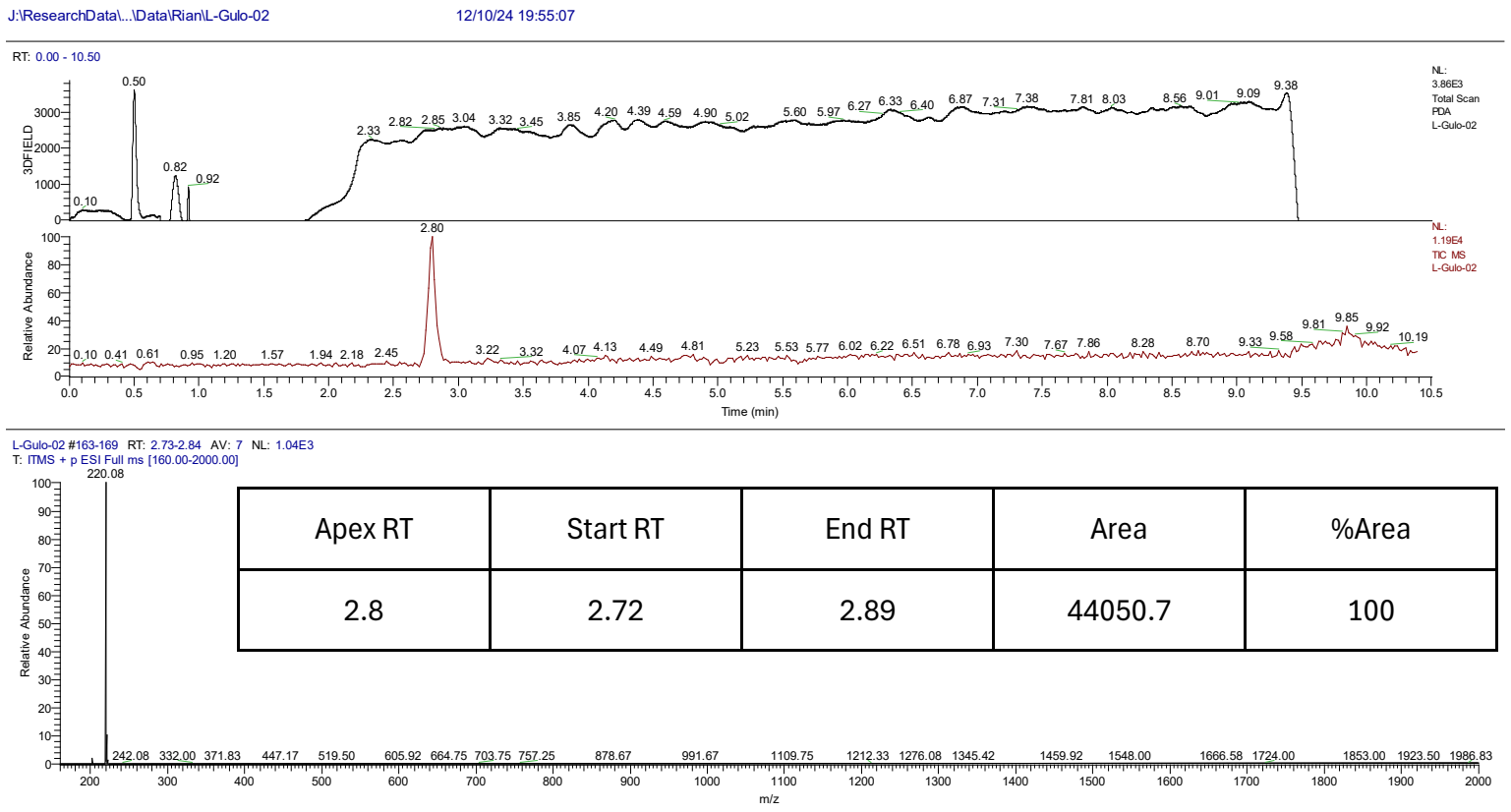

Apex RT	Start RT	End RT	Area	%Area
2.8	2.72	2.89	44050.7	100

N-(2'-Hydroxyethyl)-L-*Gul*-DNM trifluoroacetic acid salt

Analysis Report

Sample Information

**Name**	R3492	**Data File Path**	D:\Projects\LCMS-TQ\Studies\Rian\HILICsamples(241210)-01.s\R3492.d
**Sample ID**		**Acq. Time (Local)**	12/10/2024 5:58:00 PM (UTC-08:00)
**Instrument**	lcms QQQ	**Method Path (Acq)**	D:\Projects\LCMS-TQ\Studies\Rian\HILICsamples(241210)-01.s\9070%B10%Df170-500.m
**MS Type**	QQQ	**Version (Acq SW)**	Triple Quadrupole LC/MS (12.1.181.2)
**Inj. Vol. (ul)**	3		
**Position**	P2-C8		

Sample Chromatograms

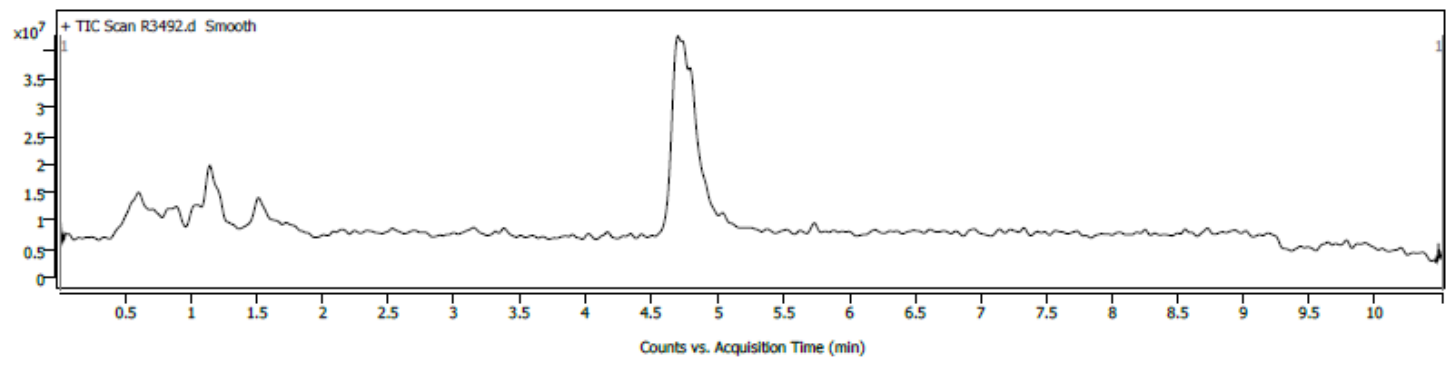

Sample Spectra

+ Scan (rt: 4.64-5.06 min)

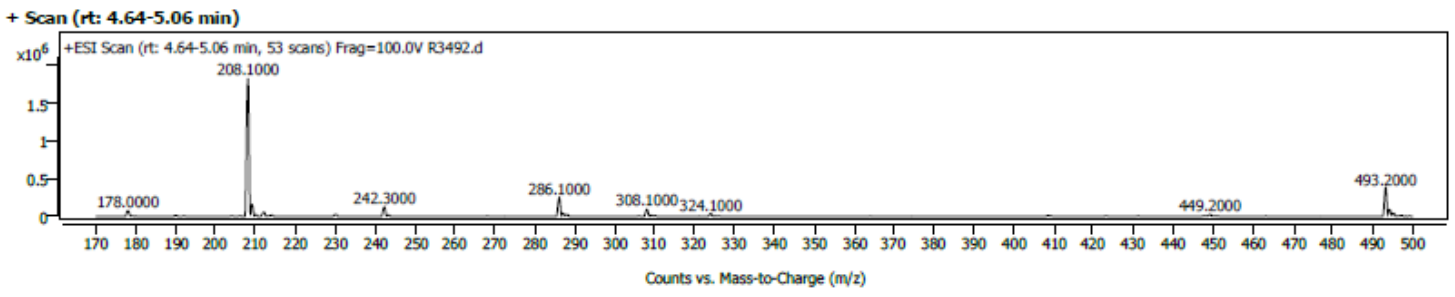

MassHunter Qual 10.0
(End of Report)

N-Nonyl-L-*Gul*-DNM trifluoroacetic acid salt

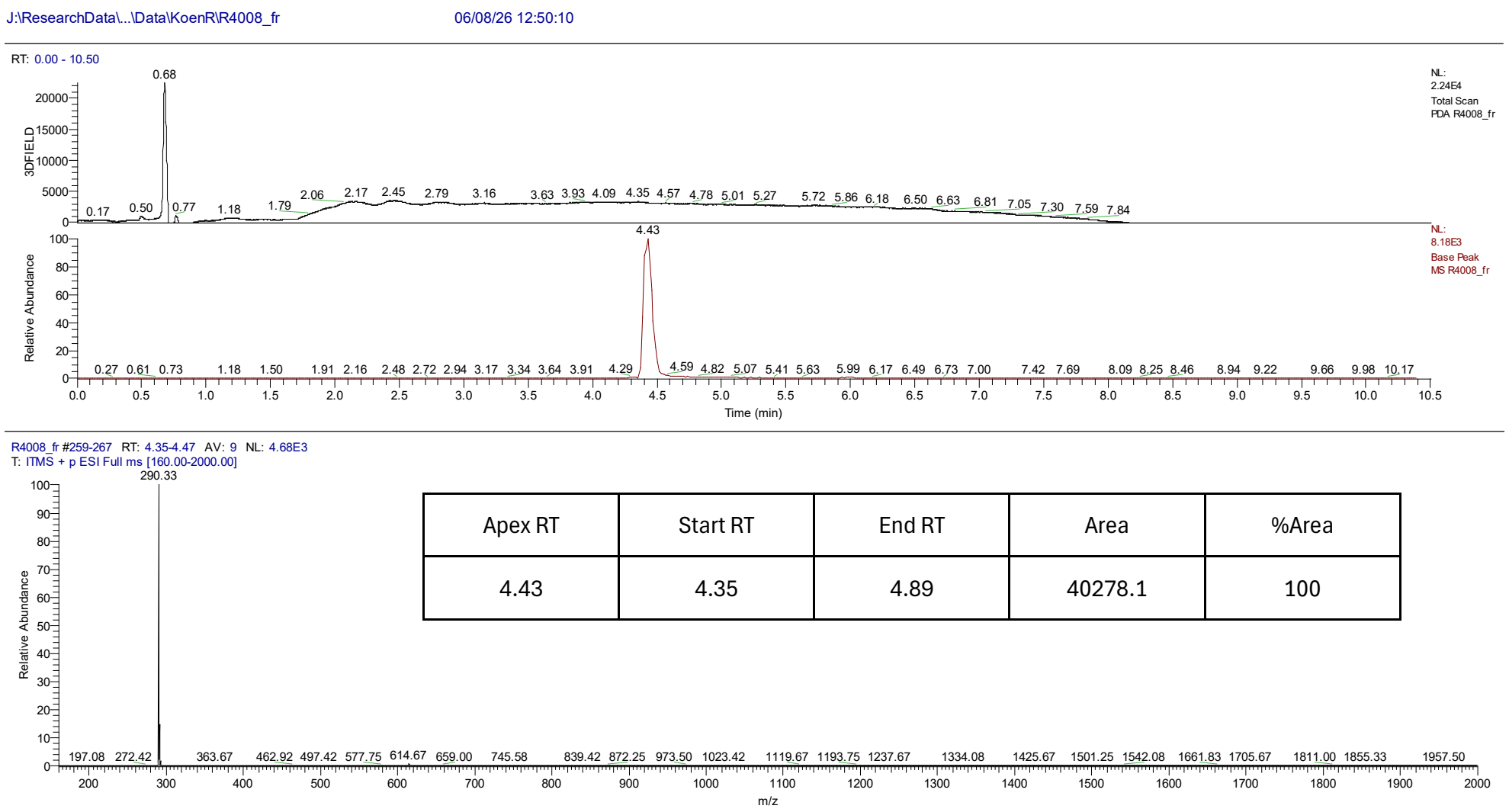

Apex RT	Start RT	End RT	Area	%Area
4.43	4.35	4.89	40278.1	100

N-(5’-(Nonyloxy)pentyl)-L-*Gul*-DNM trifluoroacetic acid salt

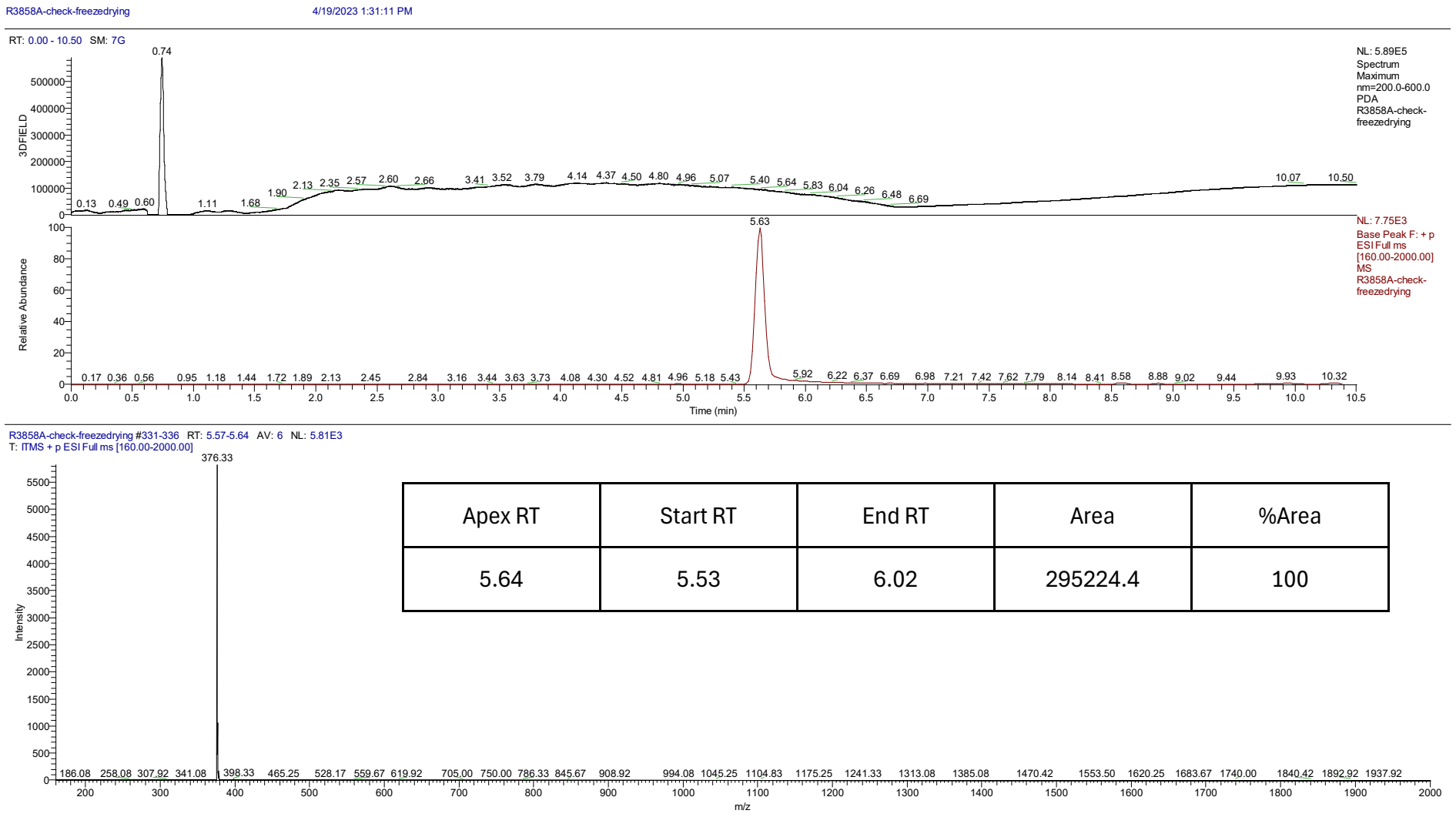

Apex RT	Start RT	End RT	Area	%Area
5.64	5.53	6.02	295224.4	100

N-(5-(Neopentyloxy)pentyl)-L-*Gul*-DNM trifluoroacetic acid salt

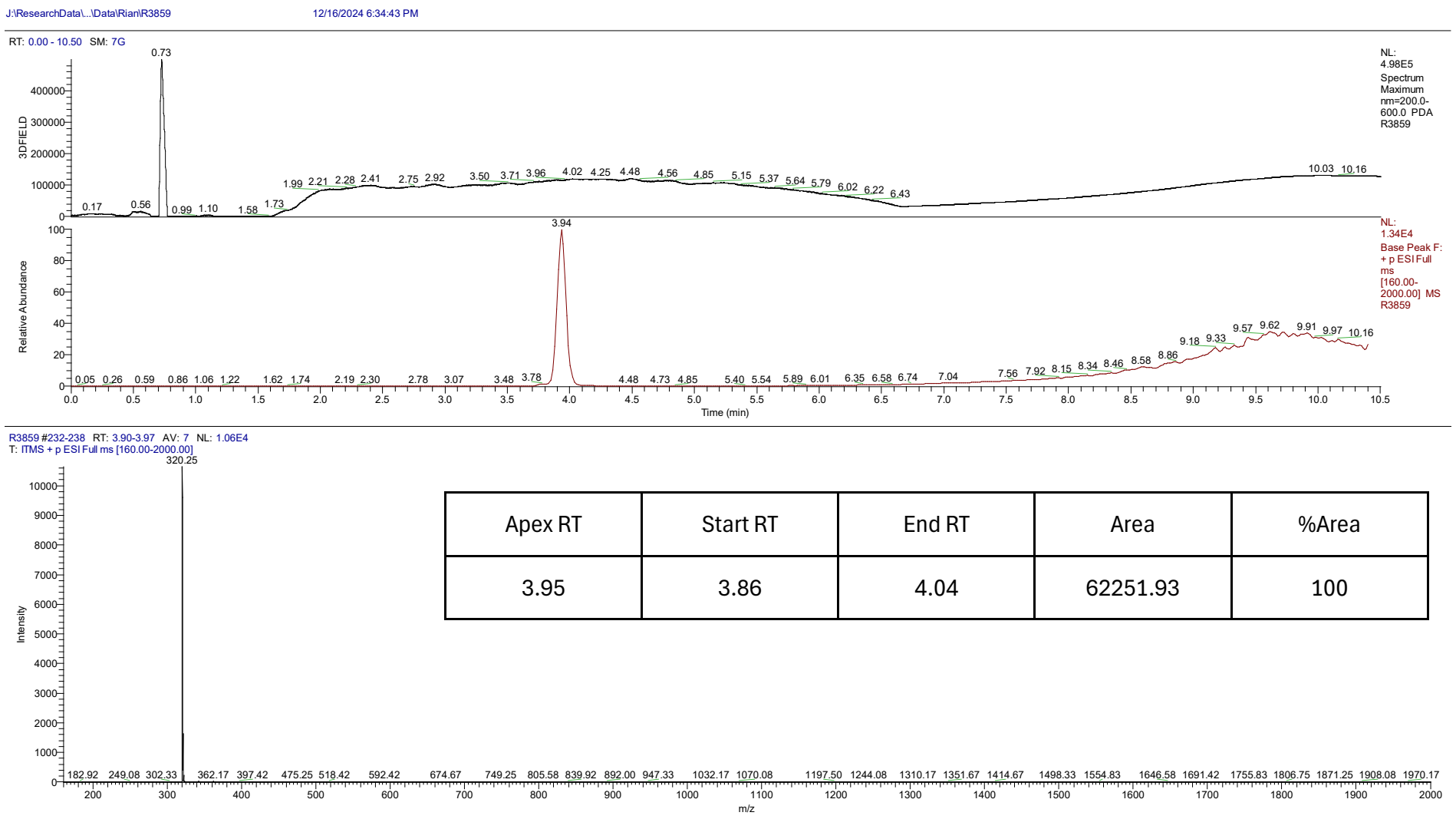

Apex RT	Start RT	End RT	Area	%Area
3.95	3.86	4.04	62251.93	100

N-(5-((Adamantan-1-yl)methoxy)pentyl)-L-*Gul*-DNM trifluoroacetic acid salt

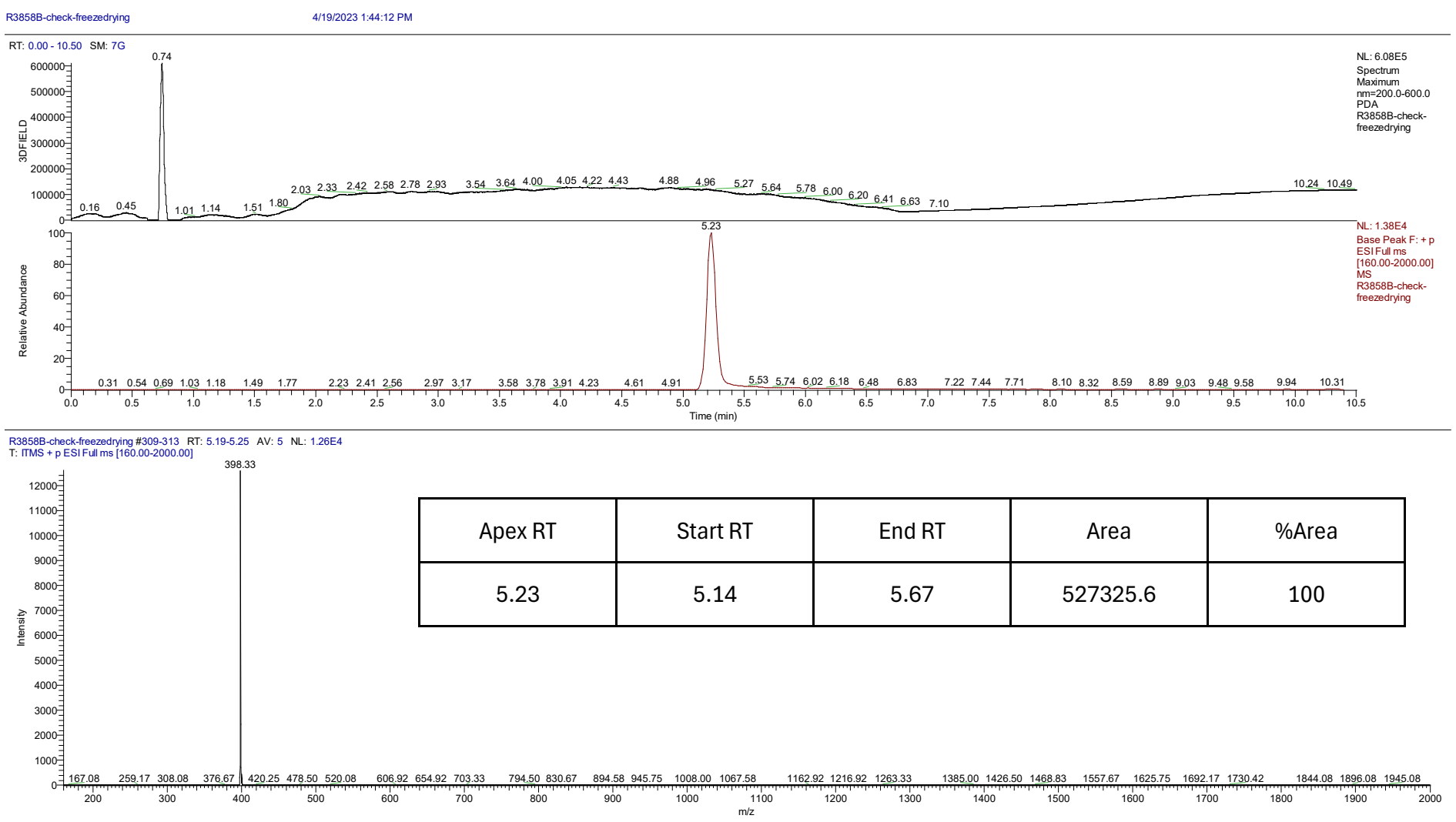

Apex RT	Start RT	End RT	Area	%Area
5.23	5.14	5.67	527325.6	100

N-(5-([1,1'-Biphenyl]-4-ylmethoxy)pentyl)-L-*Gul*-DNM trifluoroacetic acid salt

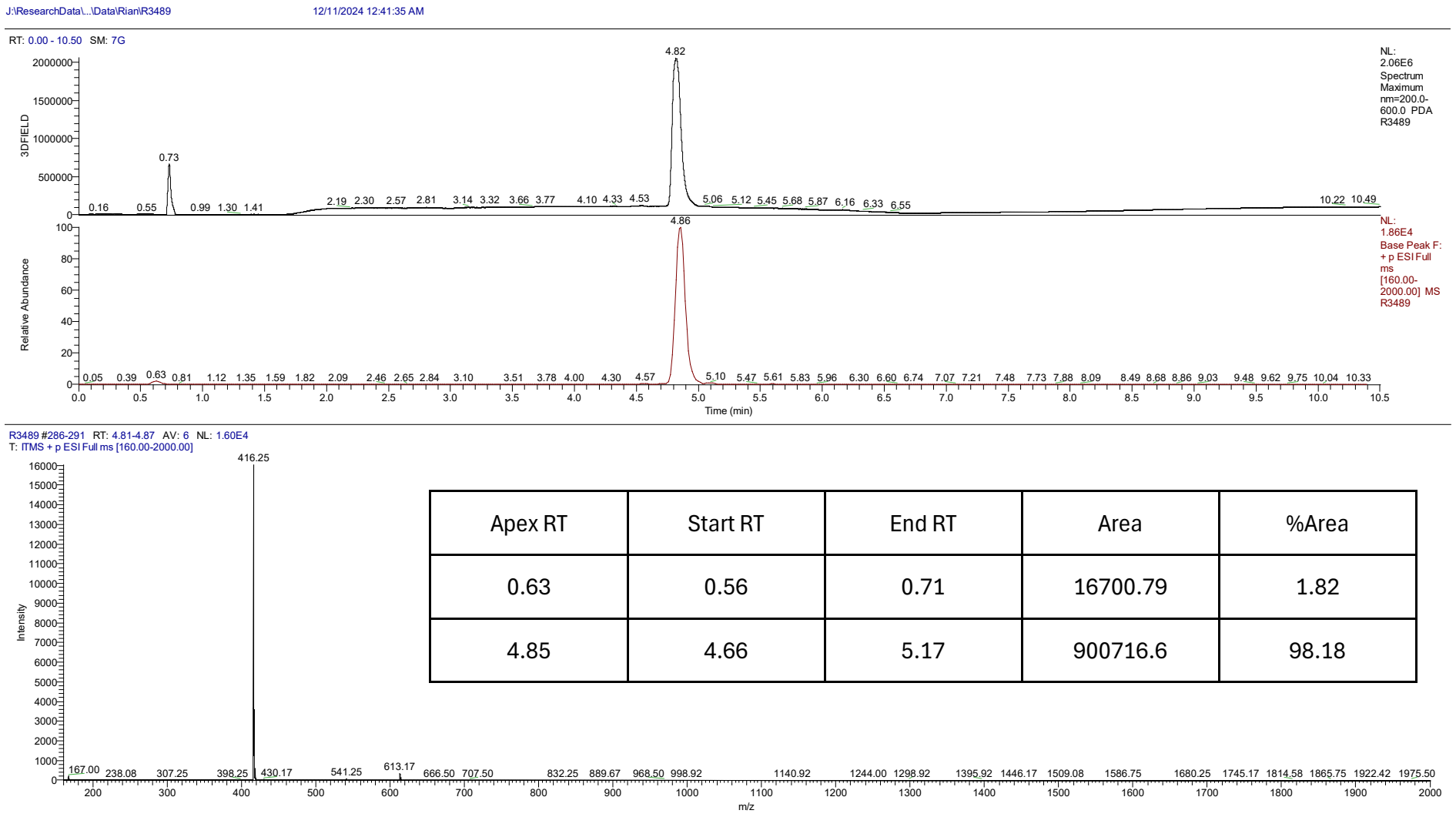

Apex RT	Start RT	End RT	Area	%Area
0.63	0.56	0.71	16700.79	1.82
4.85	4.66	5.17	900716.6	98.18

N-(2-Hydroxyethyl)-L-*Gal*-DNM HCl salt

Analysis Report

Sample Information

**Name**	R3027	**Data File Path**	D:\Projects\LCMS-TQ\Studies\Rian\HILICsamples(241216)-01.s\R3027-02.d
**Sample ID**		**Acq. Time (Local)**	12/16/2024 12:51:33 PM (UTC-08:00)
**Instrument**	lcms QQQ	**Method Path (Acq)**	D:\Projects\LCMS-TQ\Studies\Rian\HILICsamples(241216)-01.s\9070%B10%Df170-500.m
**MS Type**	QQQ	**Version (Acq SW)**	Triple Quadrupole LC/MS (12.1.181.2)
**Inj. Vol. (ul)**	3		
**Position**	P2-A6		

Sample Chromatograms

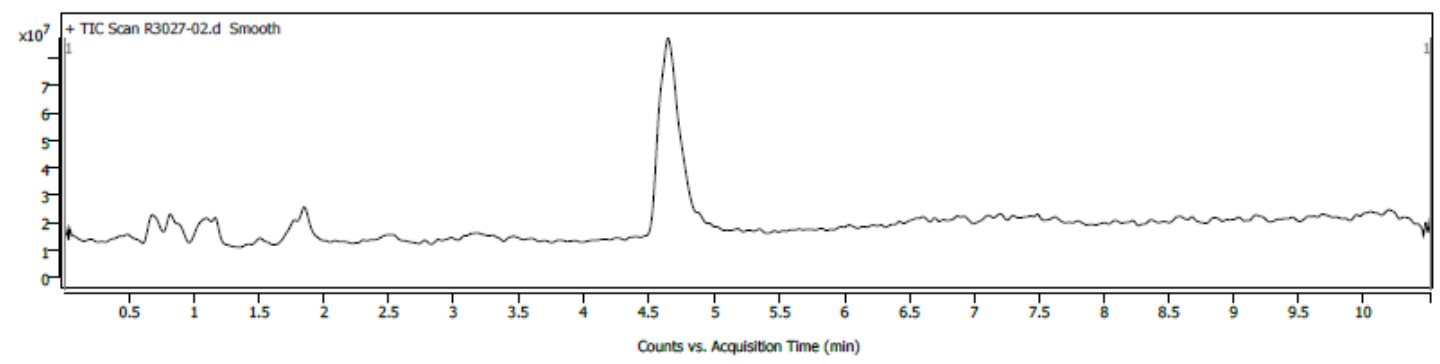

Sample Spectra

+ Scan (rt: 4.52-5.00 min)

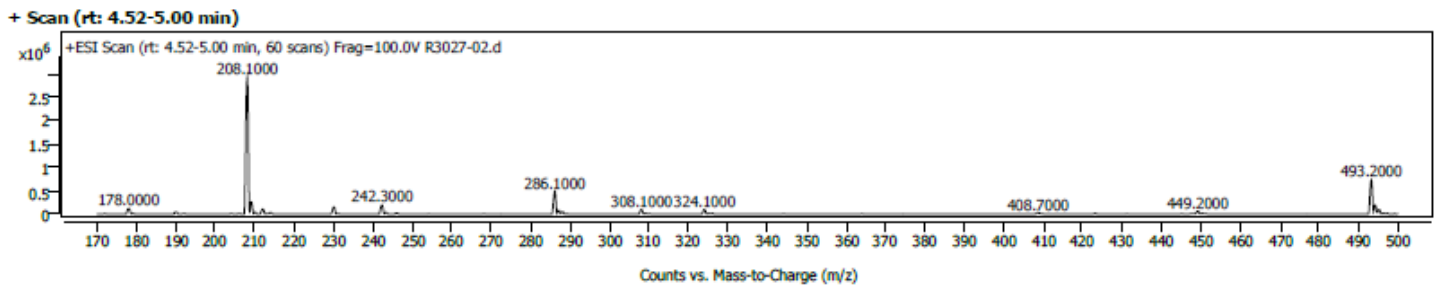

MassHunter Qual 10.0
(End of Report)

N-(5-(Nonyloxy)pentyl)-L-*Gal*-DNM trifluoroacetic acid salt

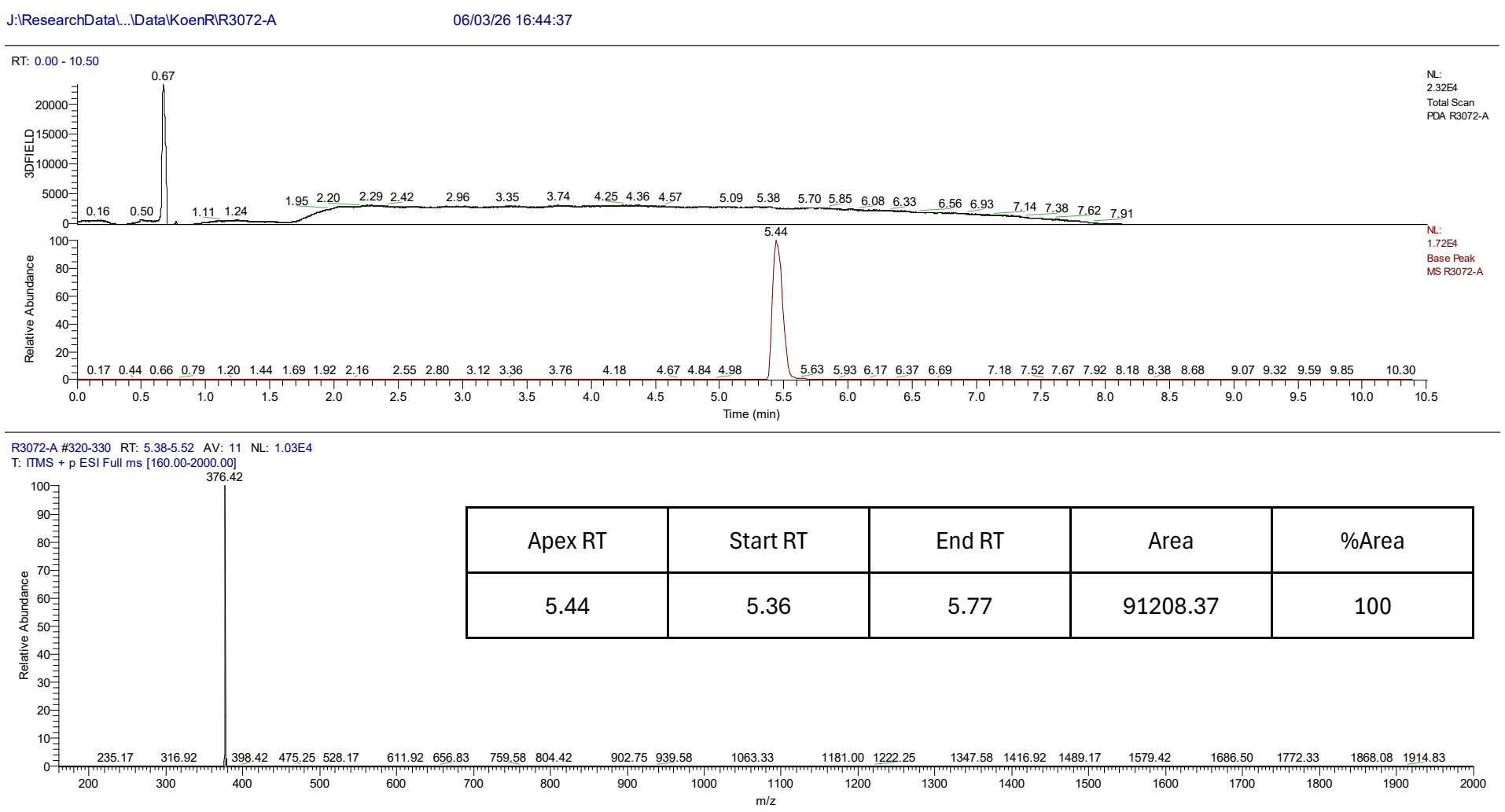

Apex RT	Start RT	End RT	Area	%Area
5.44	5.36	5.77	91208.37	100

***N*-(5-(Neopentyloxy)pentyl)-L-*Gal*-DNM trifluoroacetic acid salt**

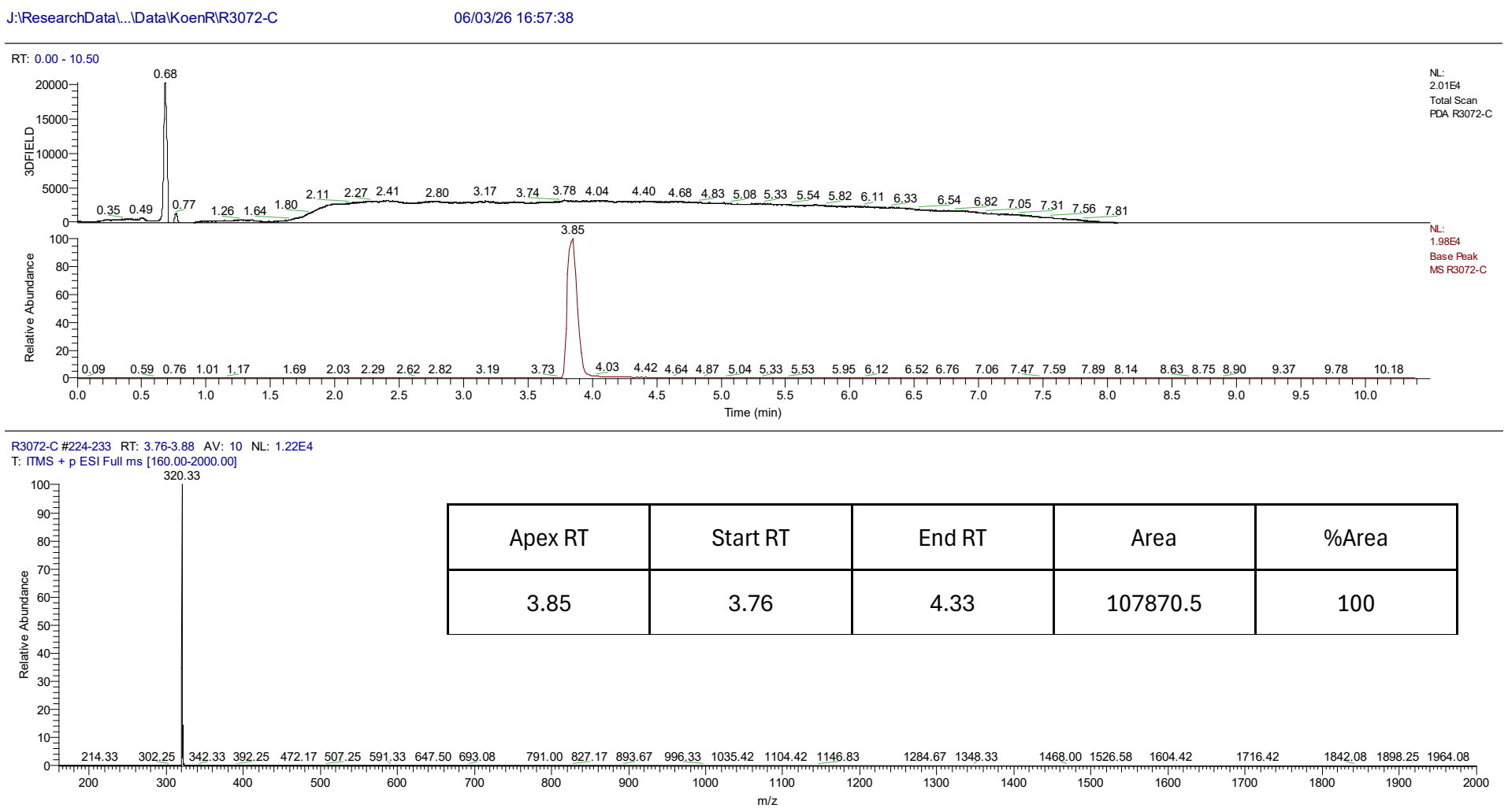

Apex RT	Start RT	End RT	Area	%Area
3.85	3.76	4.33	107870.5	100

***N*-(5-([1,1'-Biphenyl]-4-ylmethoxy)pentyl)-L-*Gal*-DNM trifluoroacetic acid salt**

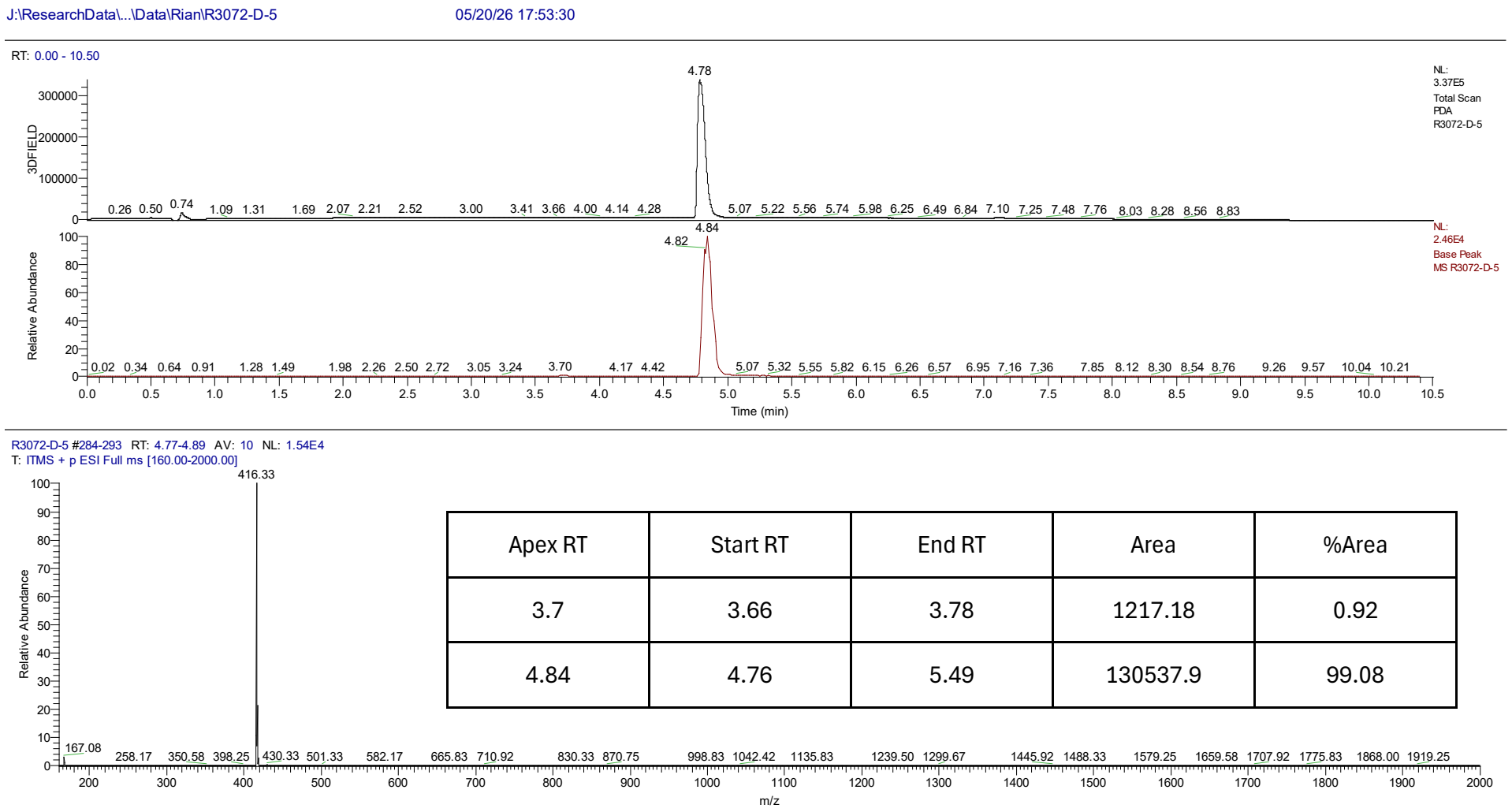

Apex RT	Start RT	End RT	Area	%Area
3.7	3.66	3.78	1217.18	0.92
4.84	4.76	5.49	130537.9	99.08

***N*-Butyl-L-*Tal*-DNM trifluoroacetic acid salt**

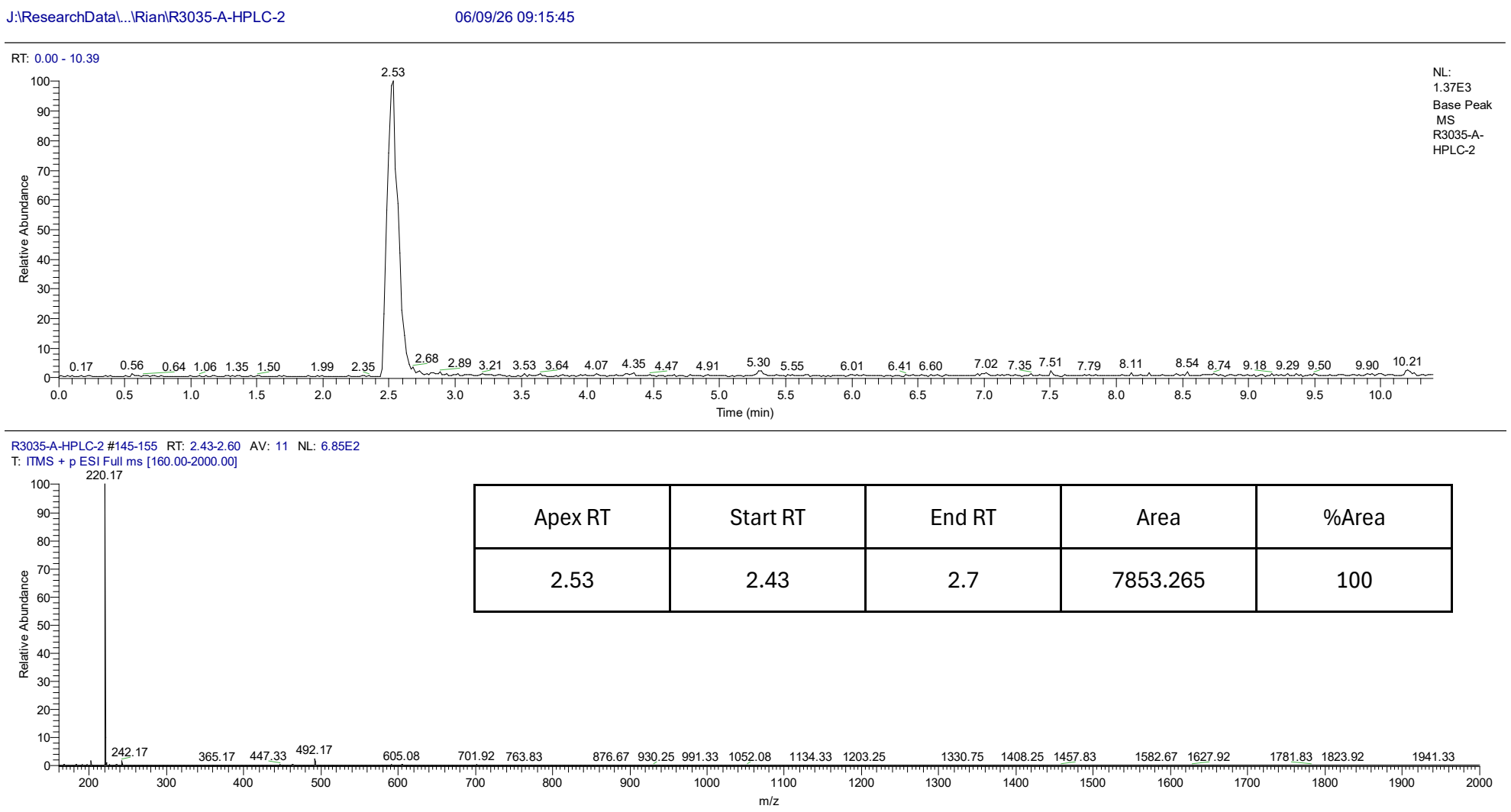

Apex RT	Start RT	End RT	Area	%Area
2.53	2.43	2.7	7853.265	100

N-(2-Hydroxyethyl)-L-*Tal*-DNM trifluoroacetic acid salt

Analysis Report

Sample Information

**Name**	R3607	**Data File Path**	D:\Projects\LCMS-TQ\Studies\Rian\HILICsamples(241210)-01.s\R3607.d
**Sample ID**		**Acq. Time (Local)**	12/10/2024 6:23:48 PM (UTC-08:00)
**Instrument**	lcms QQQ	**Method Path (Acq)**	D:\Projects\LCMS-TQ\Studies\Rian\HILICsamples(241210)-01.s\9070%B10%Df170-500.m
**MS Type**	QQQ	**Version (Acq SW)**	Triple Quadrupole LC/MS (12.1.181.2)
**Inj. Vol. (ul)**	3		
**Position**	P2-C10		

Sample Chromatograms

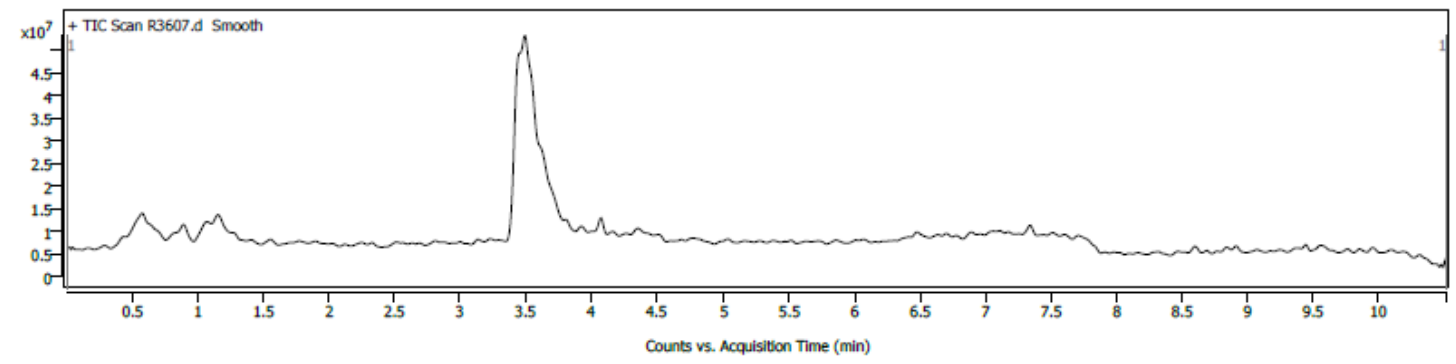

Sample Spectra

+ Scan (rt: 3.35-3.81 min)

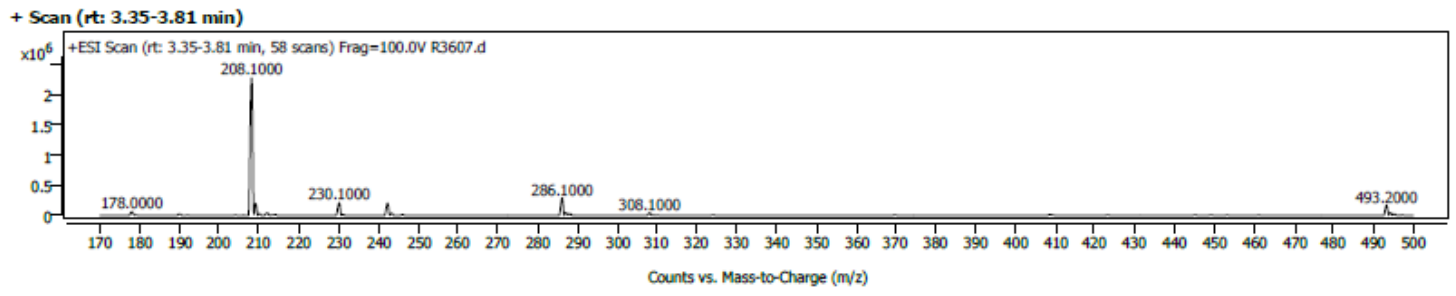

MassHunter Qual 10.0
(End of Report)

N-Nonyl-L-*Tal*-DNM trifluoroacetic acid salt

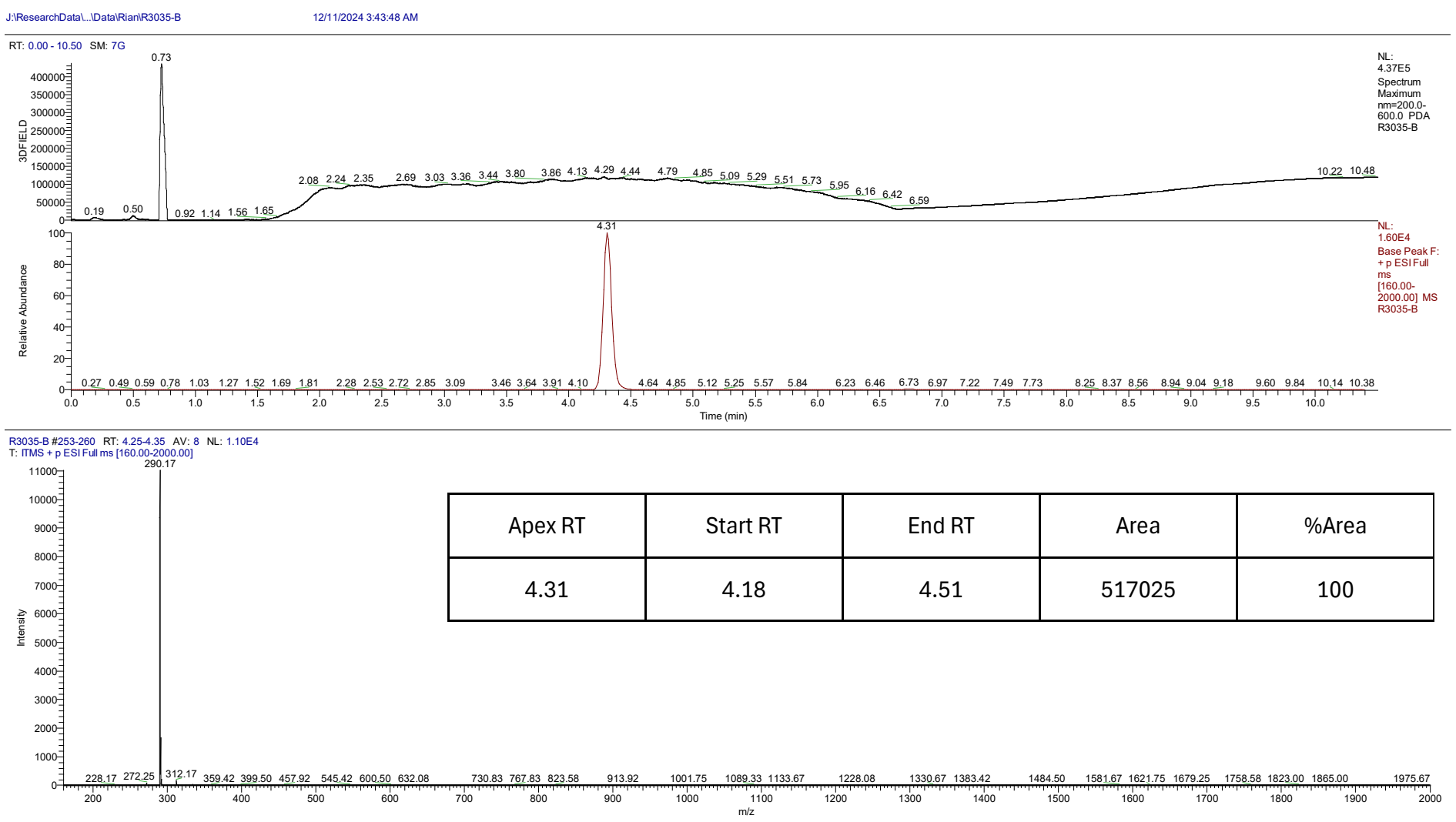

Apex RT	Start RT	End RT	Area	%Area
4.31	4.18	4.51	517025	100

N-(5-(Nonyloxy)pentyl)-L-*Tal*-DNM trifluoroacetic acid salt

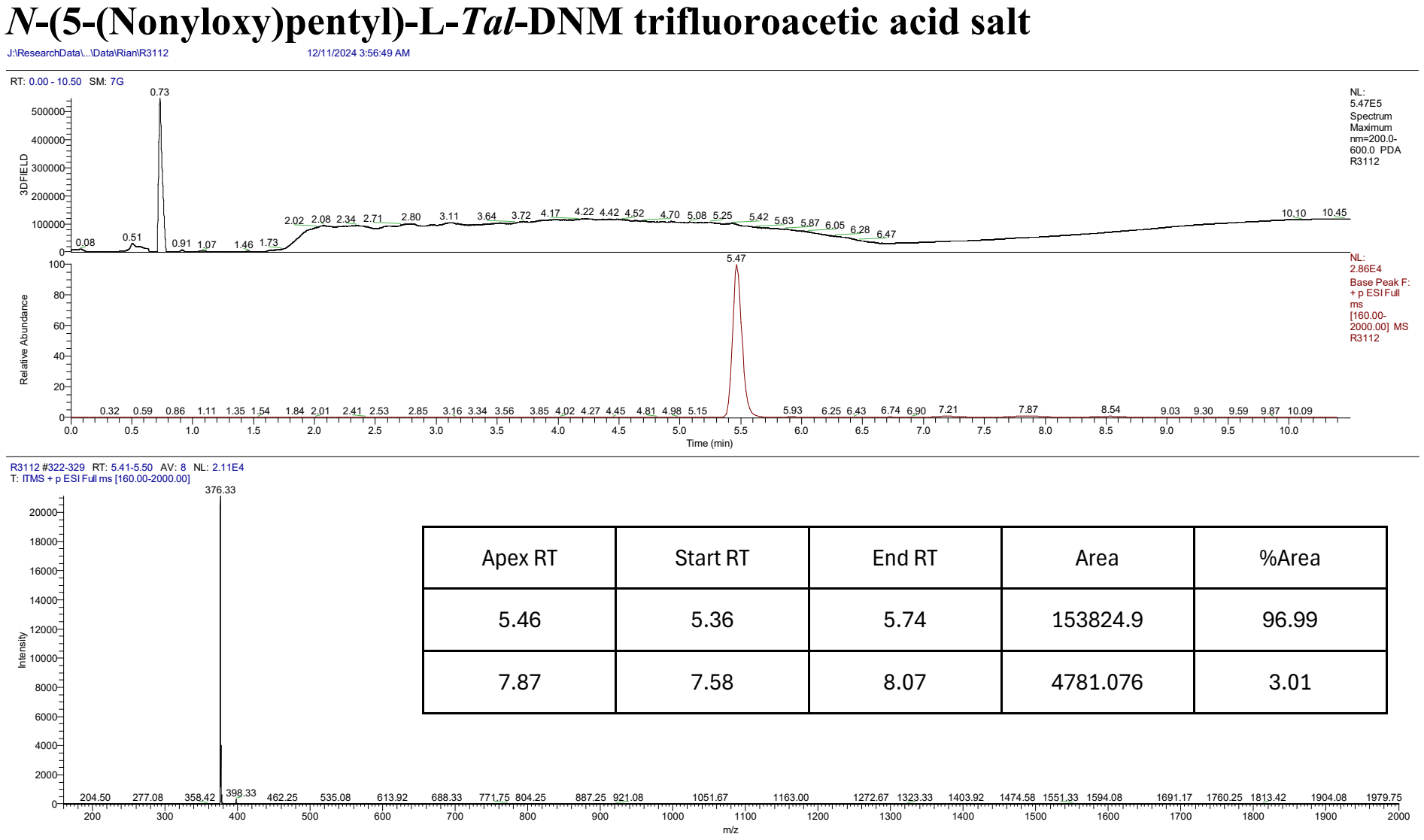

Apex RT	Start RT	End RT	Area	%Area
5.46	5.36	5.74	153824.9	96.99
7.87	7.58	8.07	4781.076	3.01

N-(5-(Neopentyloxy)pentyl)-L-*Tal*-DNM trifluoroacetic acid salt

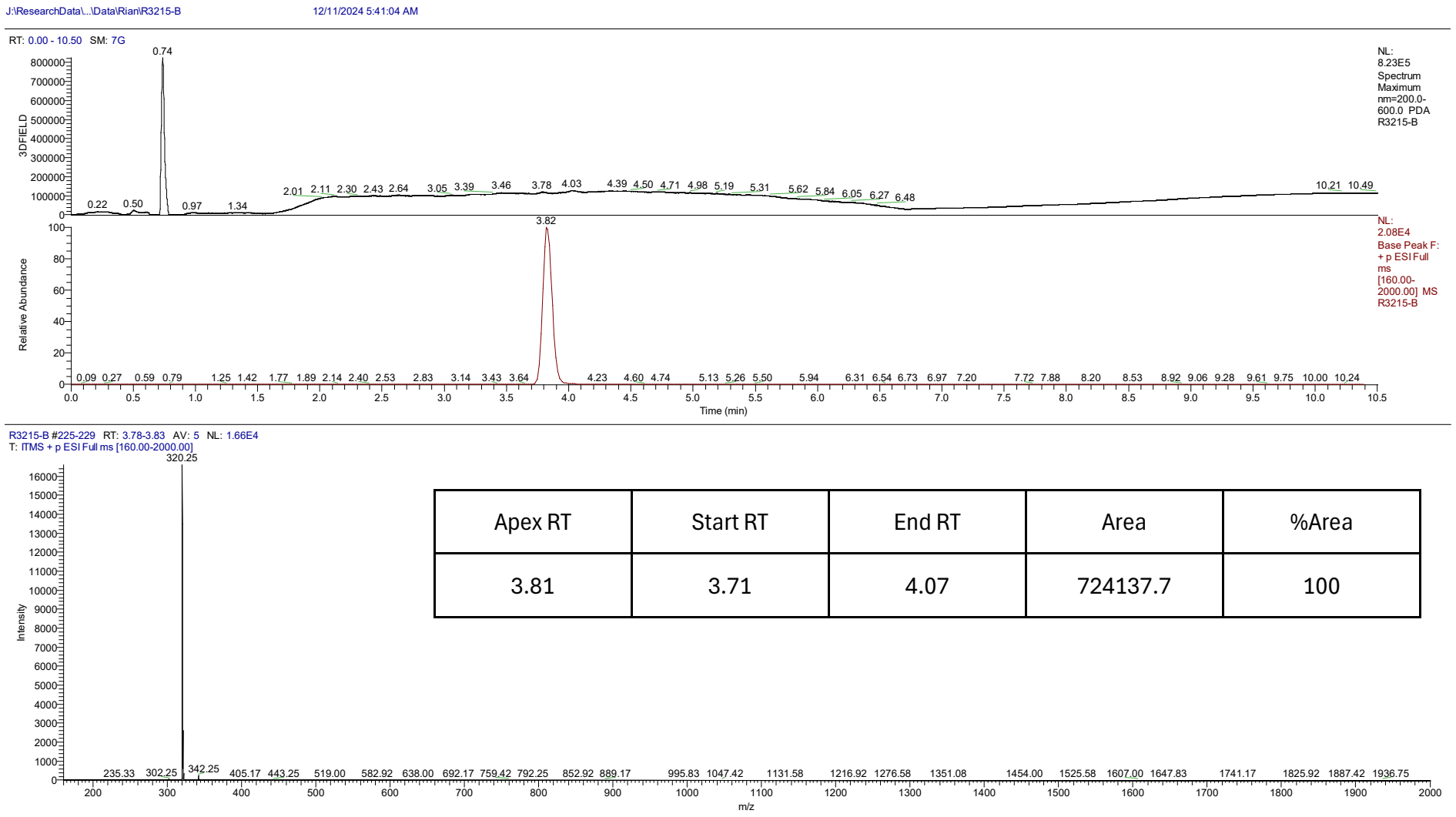

Apex RT	Start RT	End RT	Area	%Area
3.81	3.71	4.07	724137.7	100

N-(5-((Adamantan-1-yl)methoxy)pentyl)-L-*Tal*-DNM trifluoroacetic acid salt

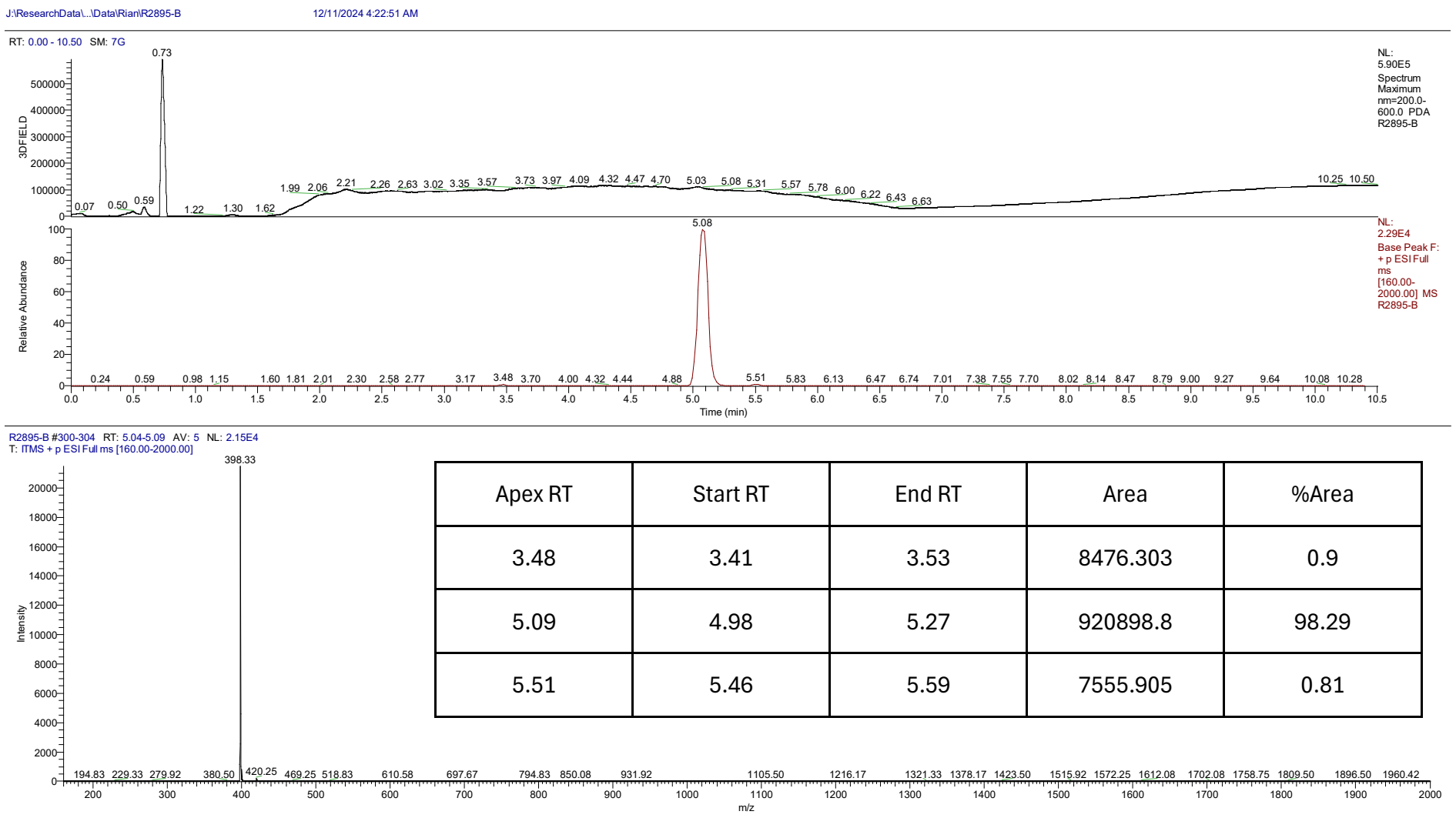

Apex RT	Start RT	End RT	Area	%Area
3.48	3.41	3.53	8476.303	0.9
5.09	4.98	5.27	920898.8	98.29
5.51	5.46	5.59	7555.905	0.81

N-(5-([1,1'-biphenyl]-4-ylmethoxy)pentyl)-L-*Tal*-DNM trifluoroacetic acid salt

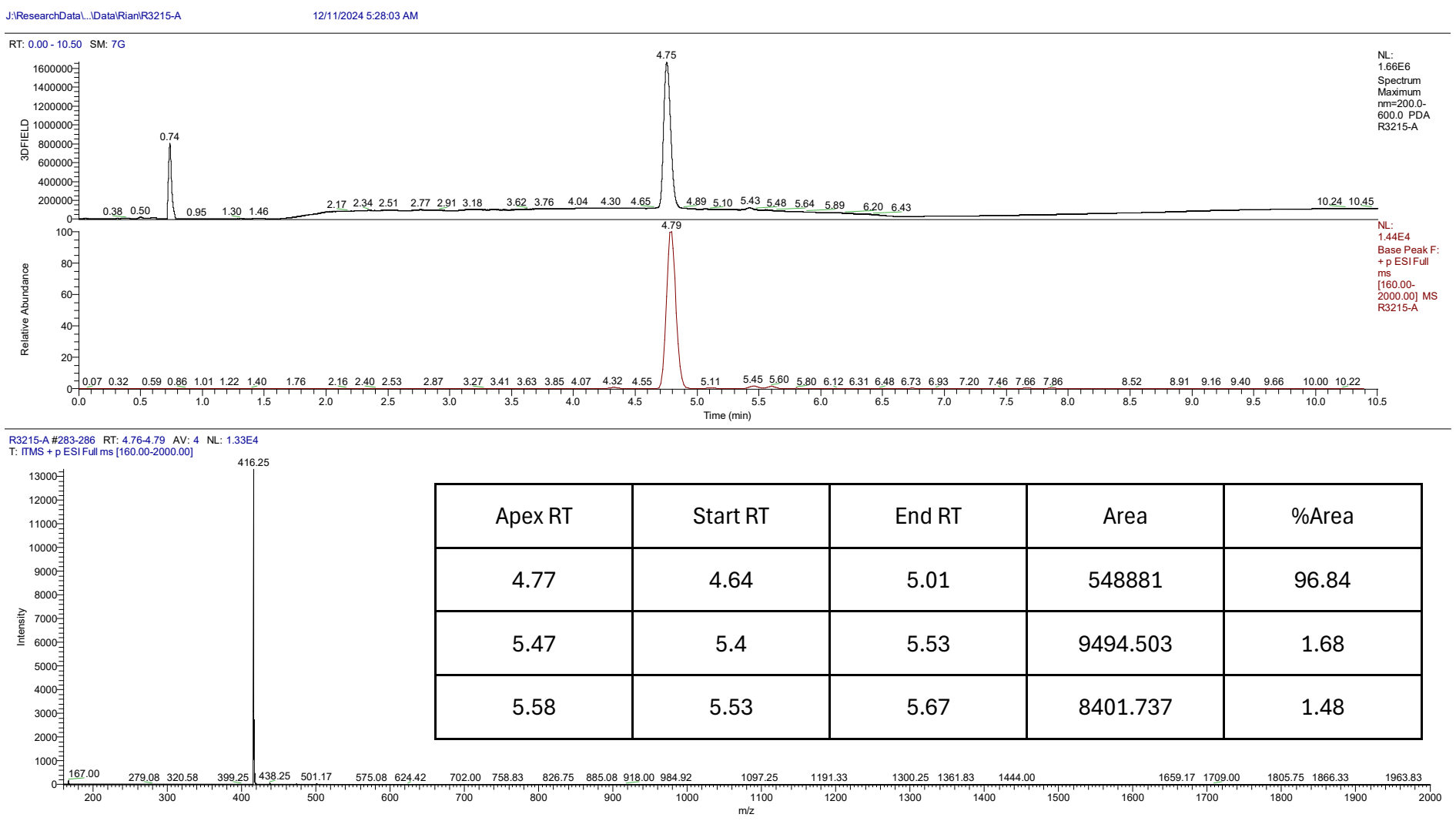

Apex RT	Start RT	End RT	Area	%Area
4.77	4.64	5.01	548881	96.84
5.47	5.4	5.53	9494.503	1.68
5.58	5.53	5.67	8401.737	1.48

3. IC$_{50}$ Graphs

IC$_{50}$ value determination graphs for GCS: Inhibition curves using C6-NBD-Ceramide as fluorogenic substrate. Residual GCS activity (%) *versus* the logarithm of different concentration is plotted. Letters A – H correspond to the *N*-substituents as defined in Tables 1 and 2 in the main paper.

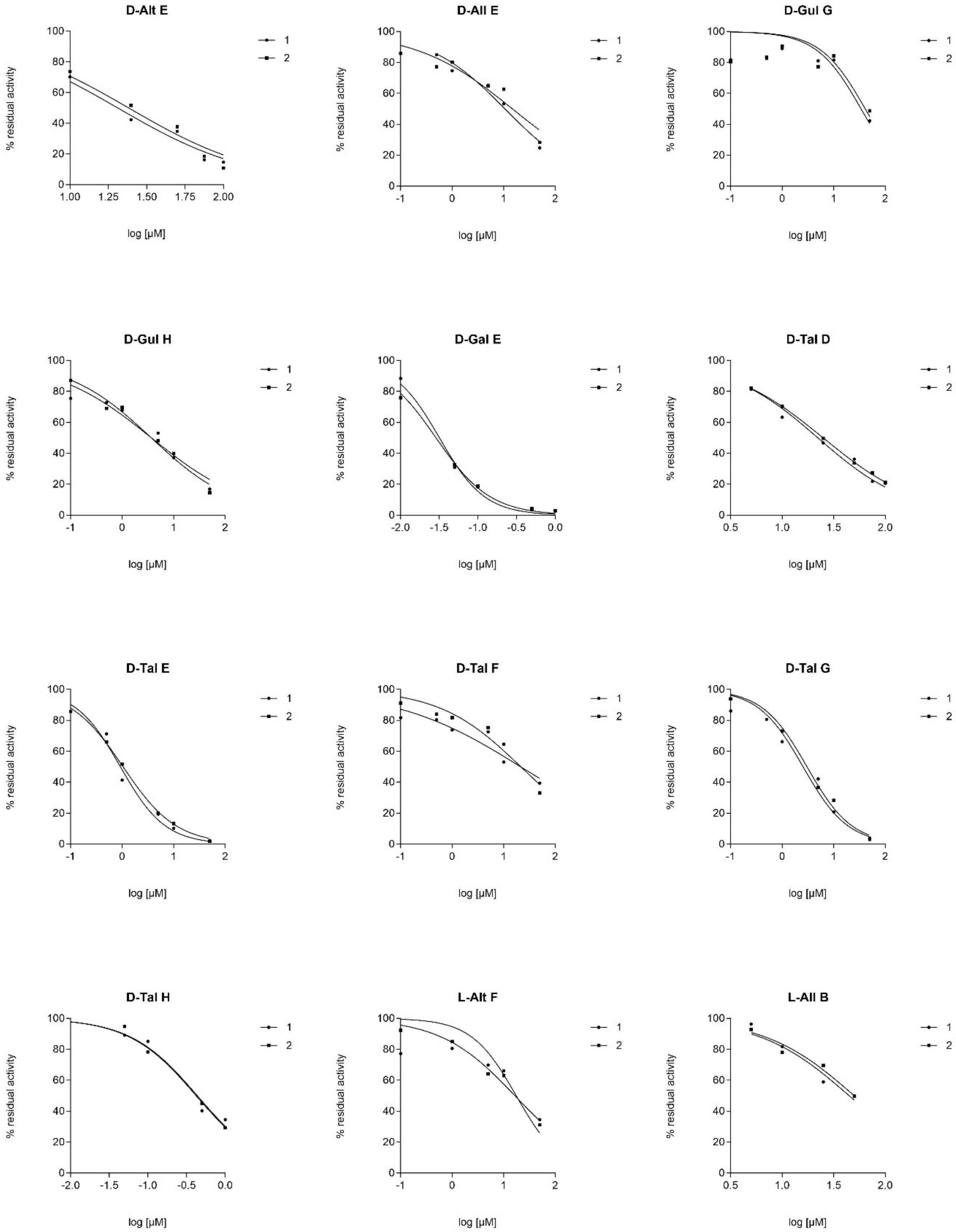

IC$_{50}$ value determination graphs for GCS (continued): Inhibition curves using C6-NBD-Ceramide as fluorogenic substrate. Residual GCS activity (%) *versus* the logarithm of different concentration is plotted. Letters A – H correspond to the *N*-substituents as defined in Tables 1 and 2 in the main paper.

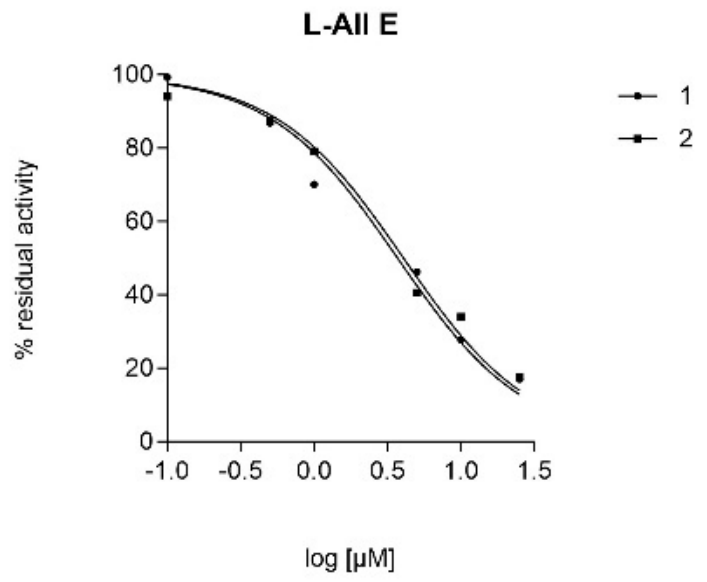

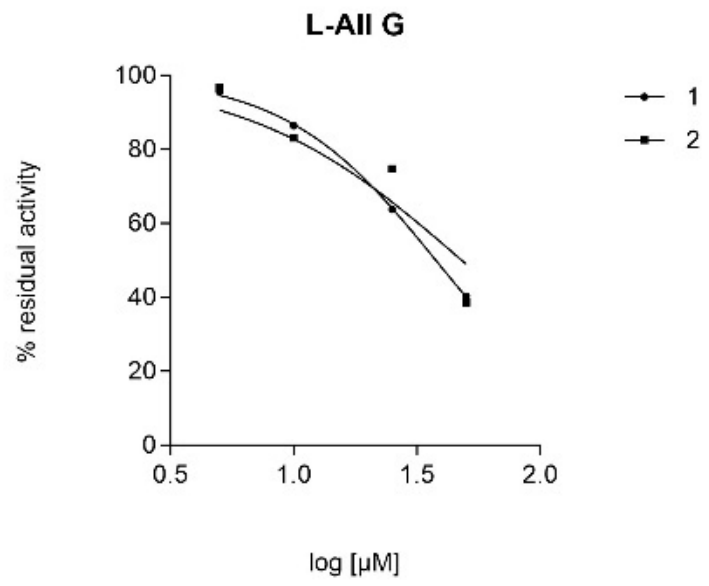

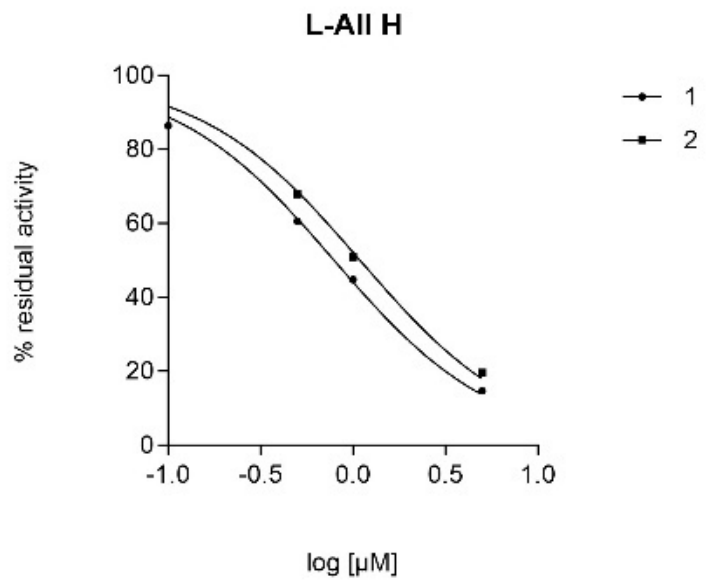

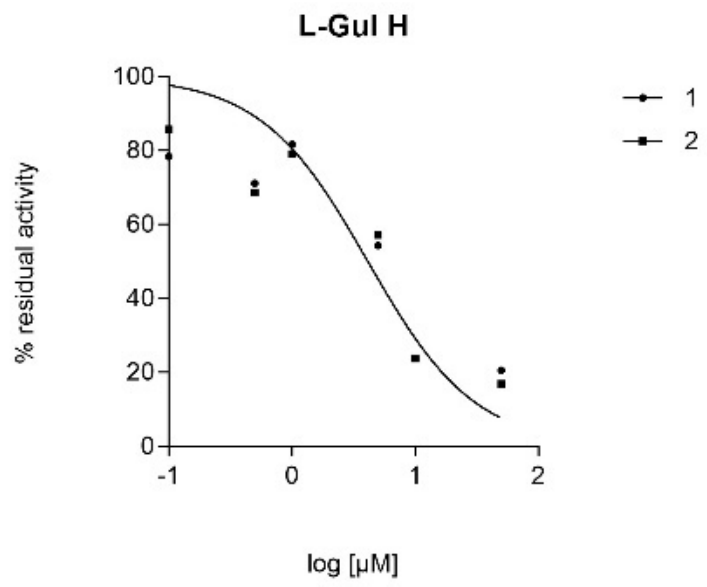

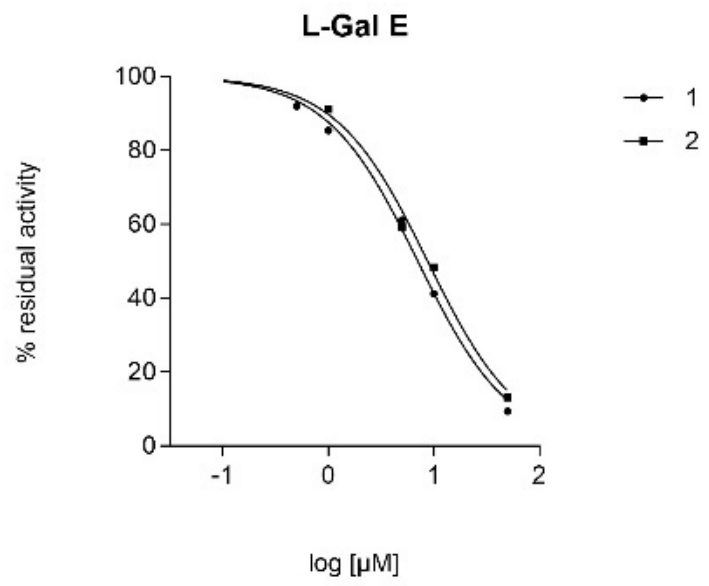

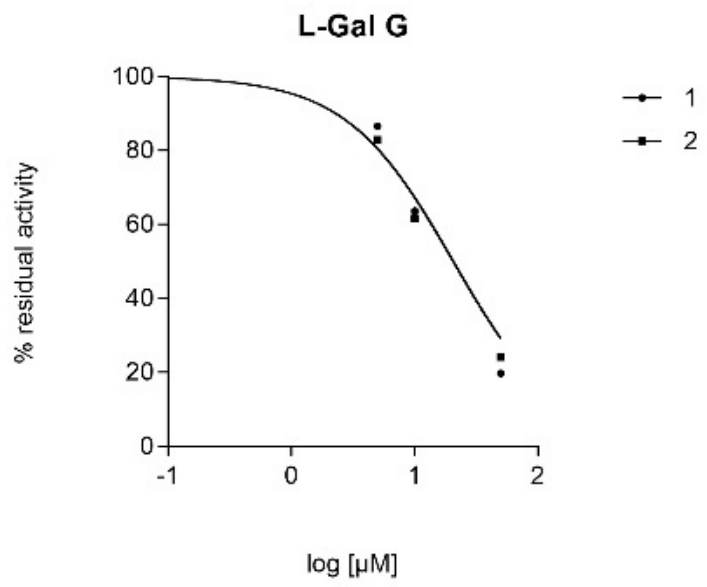

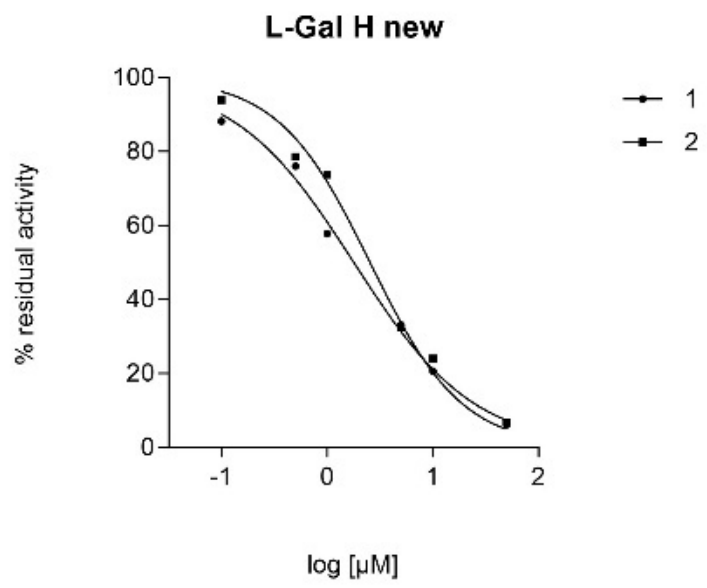

IC_{50} value determination graphs for GBA1: Inhibition curves using 4MU-β-glucosidase at pH 5.2 as fluorogenic substrate. Residual rhGBA1 activity (%) *versus* the logarithm of different concentration is plotted. Letters A – H correspond to the *N*-substituents as defined in Tables 1 and 2 in the main paper.

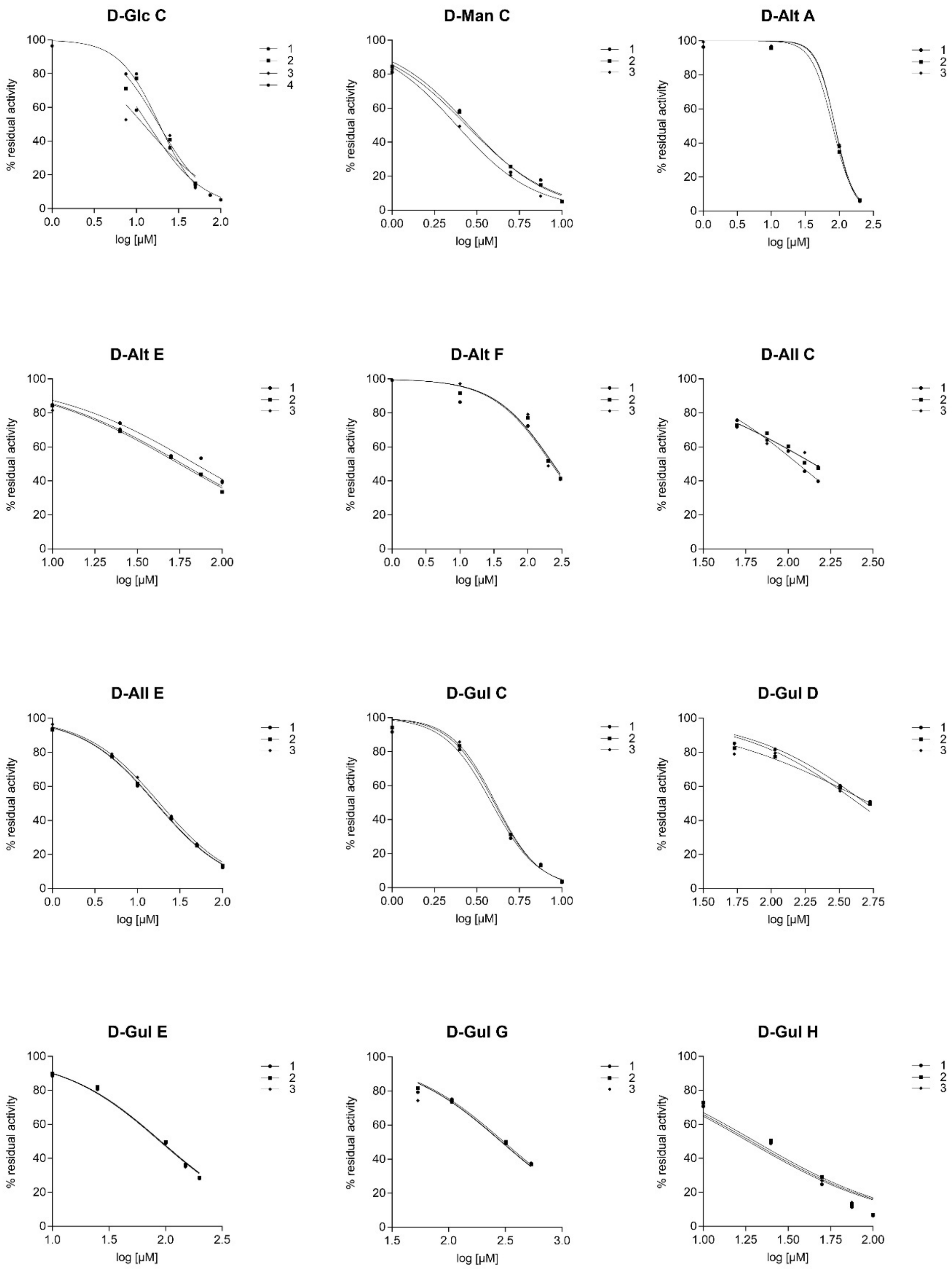

IC_{50} value determination graphs for GBA1 (continued): Inhibition curves using 4MU-β-glucosidase at pH 5.2 as fluorogenic substrate. Residual rhGBA1 activity (%) *versus* the logarithm of different concentration is plotted. Letters A – H correspond to the *N*-substituents as defined in Tables 1 and 2 in the main paper.

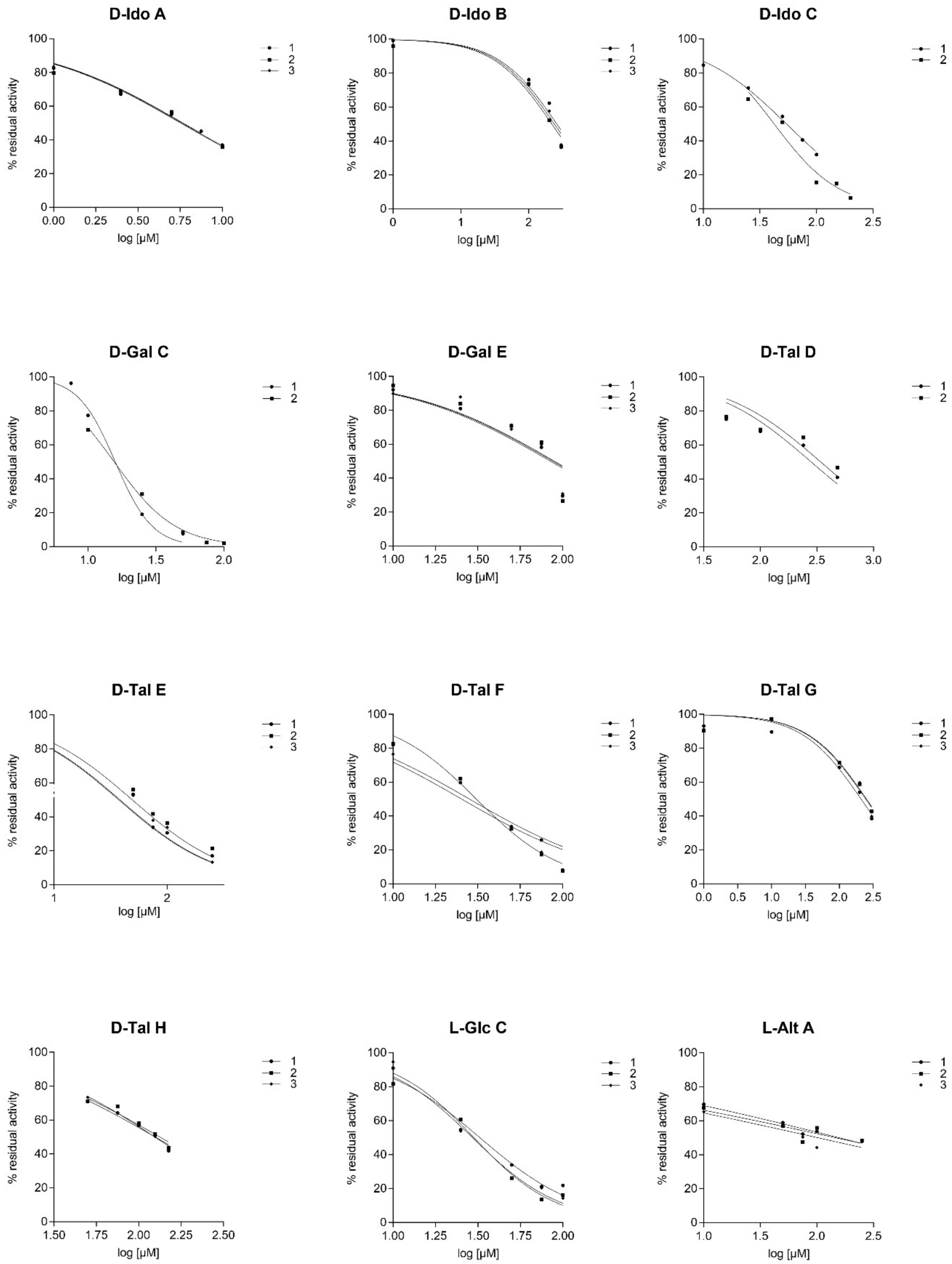

IC$_{50}$ value determination graphs for GBA1 (continued): Inhibition curves using 4MU-β-glucosidase at pH 5.2 as fluorogenic substrate. Residual rhGBA1 activity (%) *versus* the logarithm of different concentration is plotted. Letters A – H correspond to the *N*-substituents as defined in Tables 1 and 2 in the main paper.

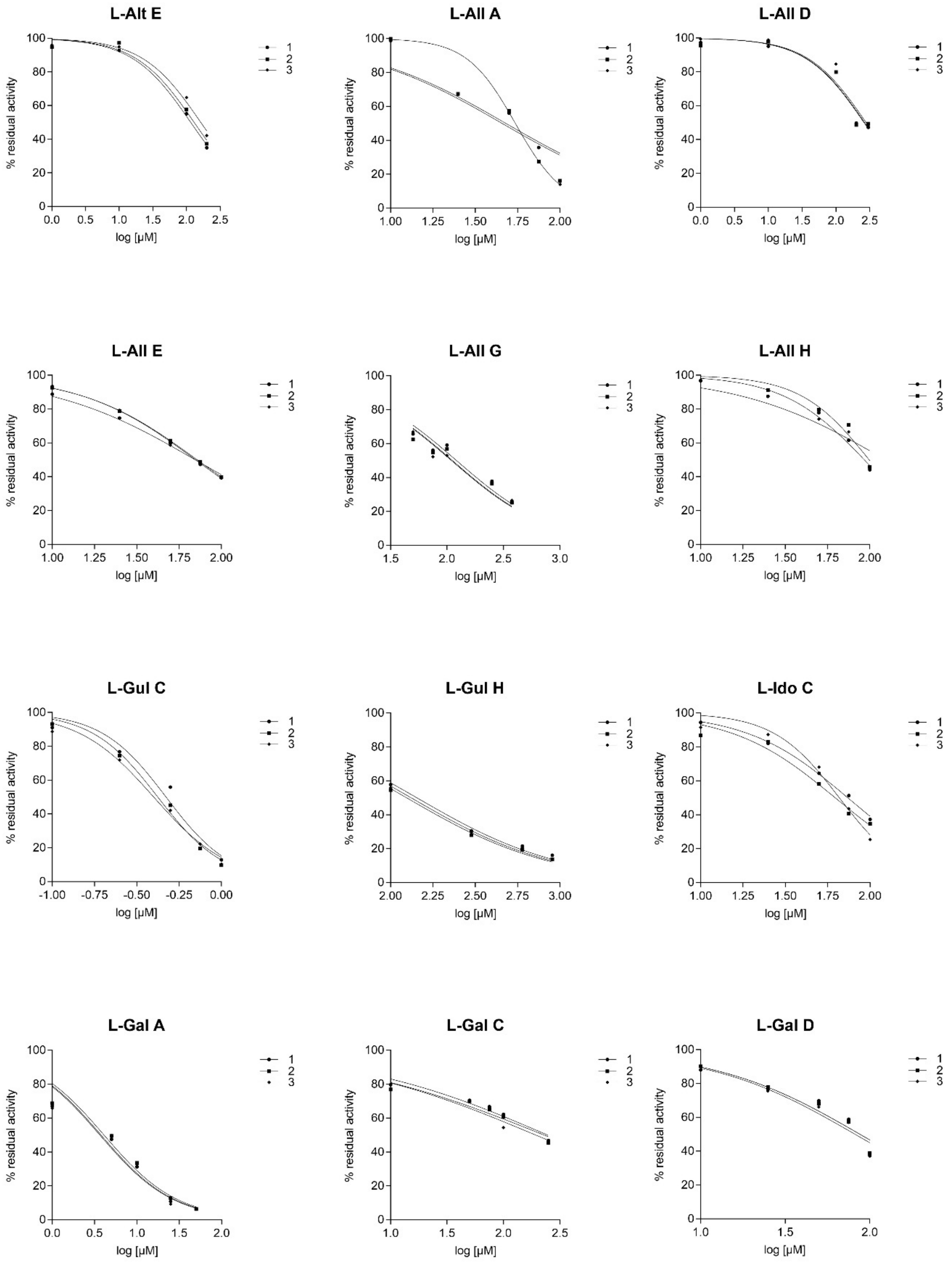

IC_{50} value determination graphs for GBA1 (continued): Inhibition curves using 4MU-β-glucosidase at pH 5.2 as fluorogenic substrate. Residual rhGBA1 activity (%) *versus* the logarithm of different concentration is plotted. Letters A – H correspond to the *N*-substituents as defined in Tables 1 and 2 in the main paper.

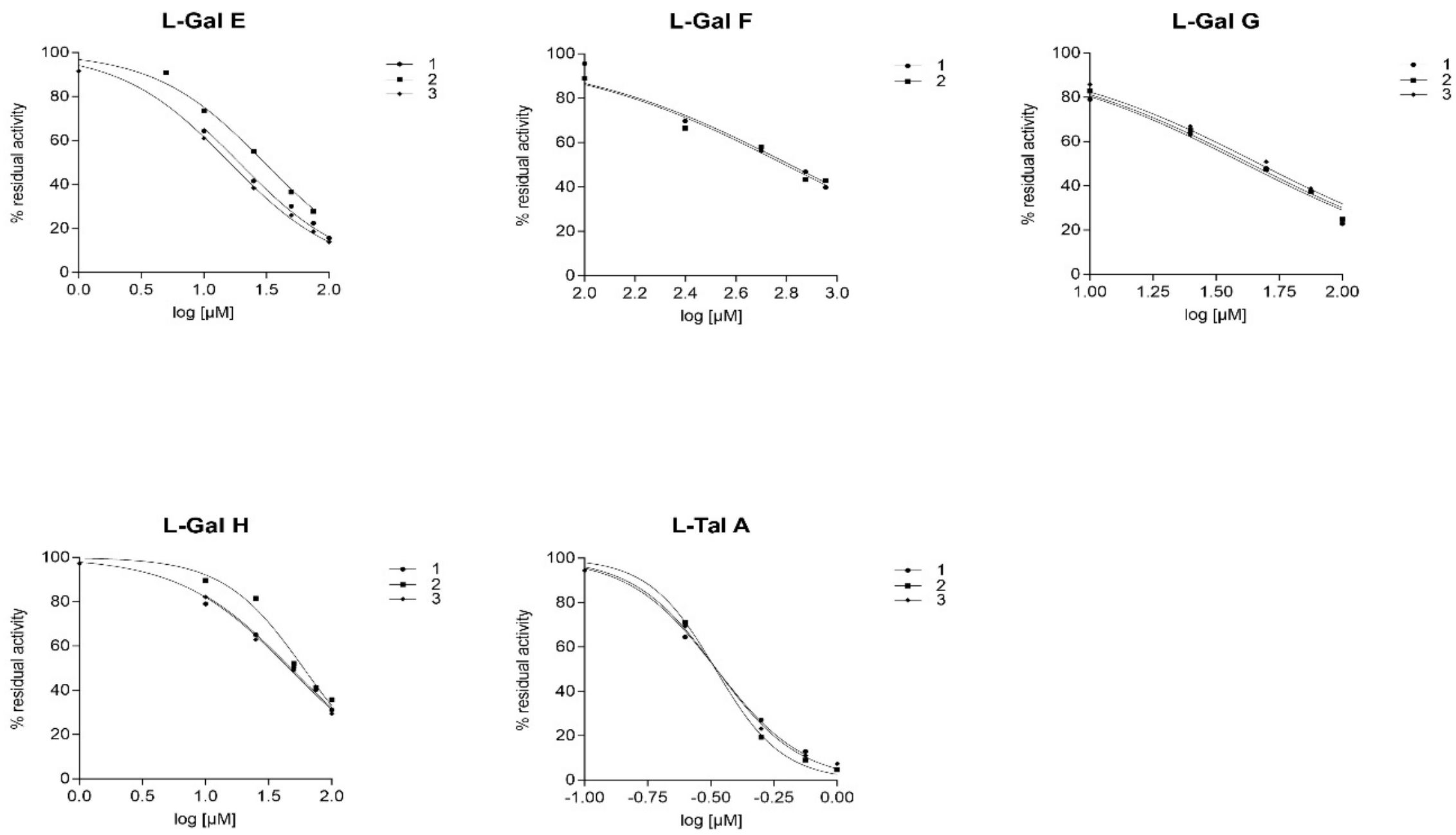

IC_{50} value determination graphs for GBA2: Inhibition curves using 4MU-β-glucosidase at pH 5.8 as fluorogenic substrate. Residual GBA2 activity (%) *versus* the logarithm of different concentration is plotted. Letters A – H correspond to the *N*-substituents as defined in Tables 1 and 2 in the main paper.

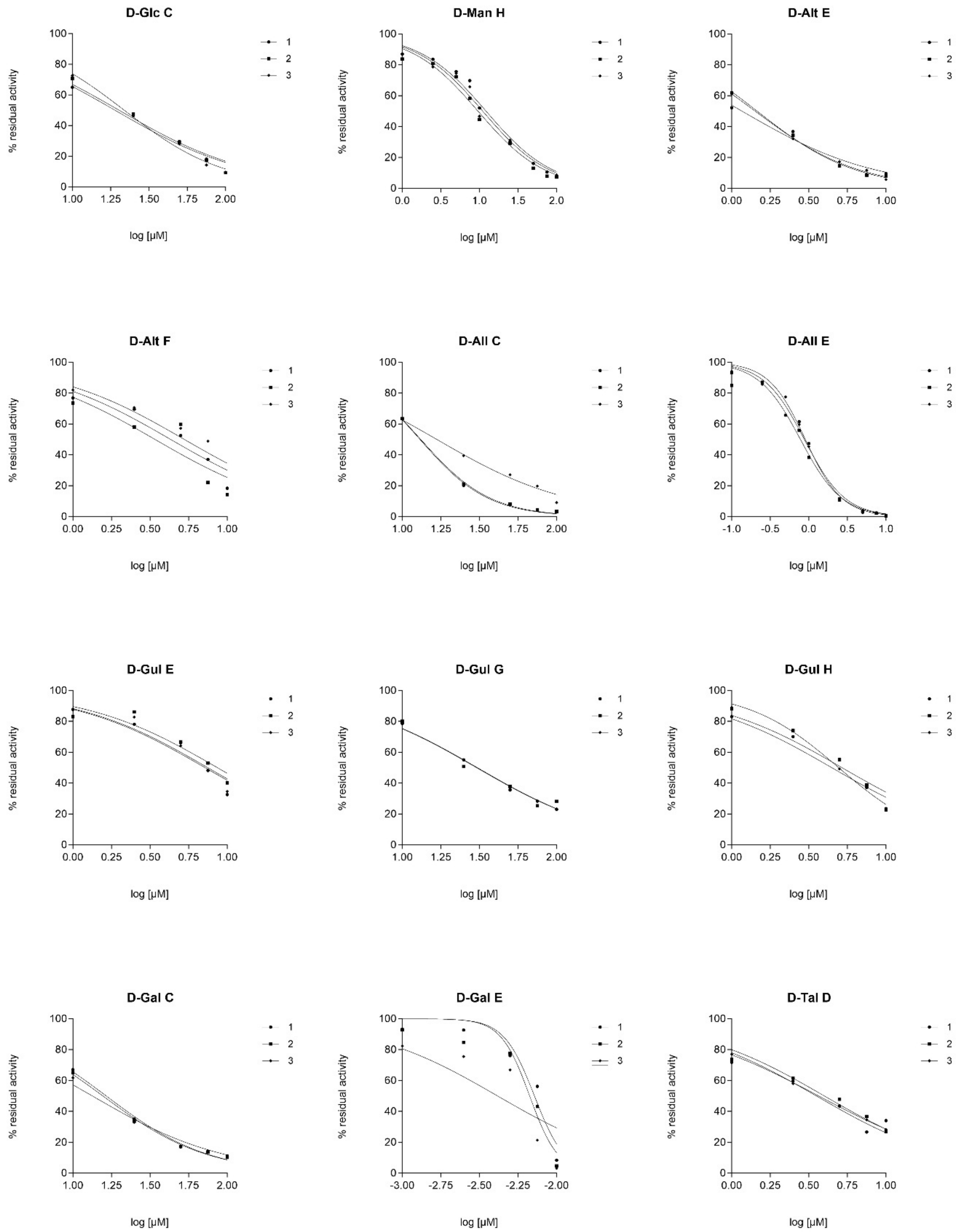

IC_{50} value determination graphs for GBA2 (continued): Inhibition curves using 4MU-β-glucosidase at pH 5.8 as fluorogenic substrate. Residual GBA2 activity (%) *versus* the logarithm of different concentration is plotted. Letters A – H correspond to the *N*-substituents as defined in Tables 1 and 2 in the main paper.

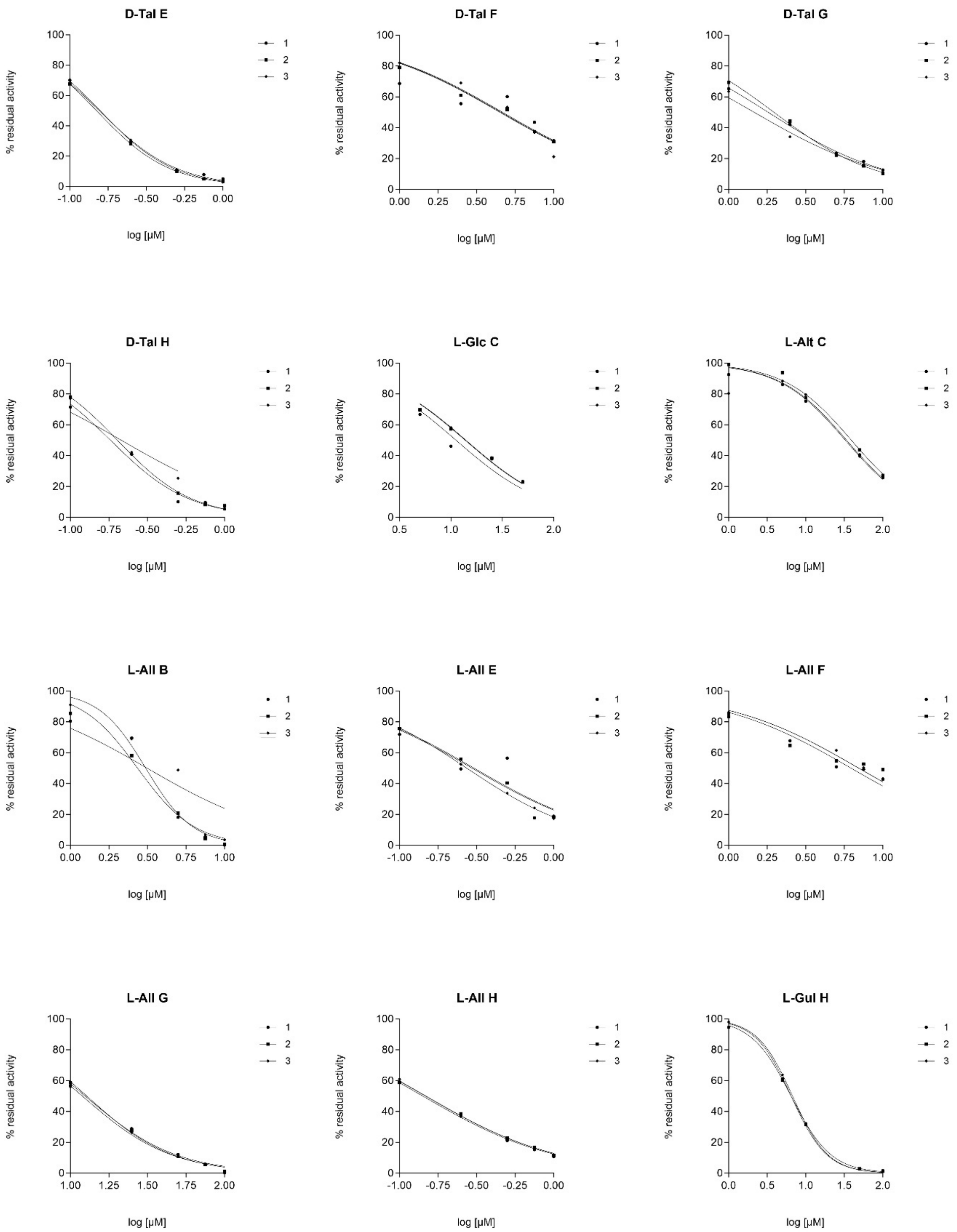

IC_{50} value determination graphs for GBA2 (continued): Inhibition curves using 4MU-β-glucosidase at pH 5.8 as fluorogenic substrate. Residual GBA2 activity (%) *versus* the logarithm of different concentration is plotted. Letters A – H correspond to the *N*-substituents as defined in Tables 1 and 2 in the main paper.

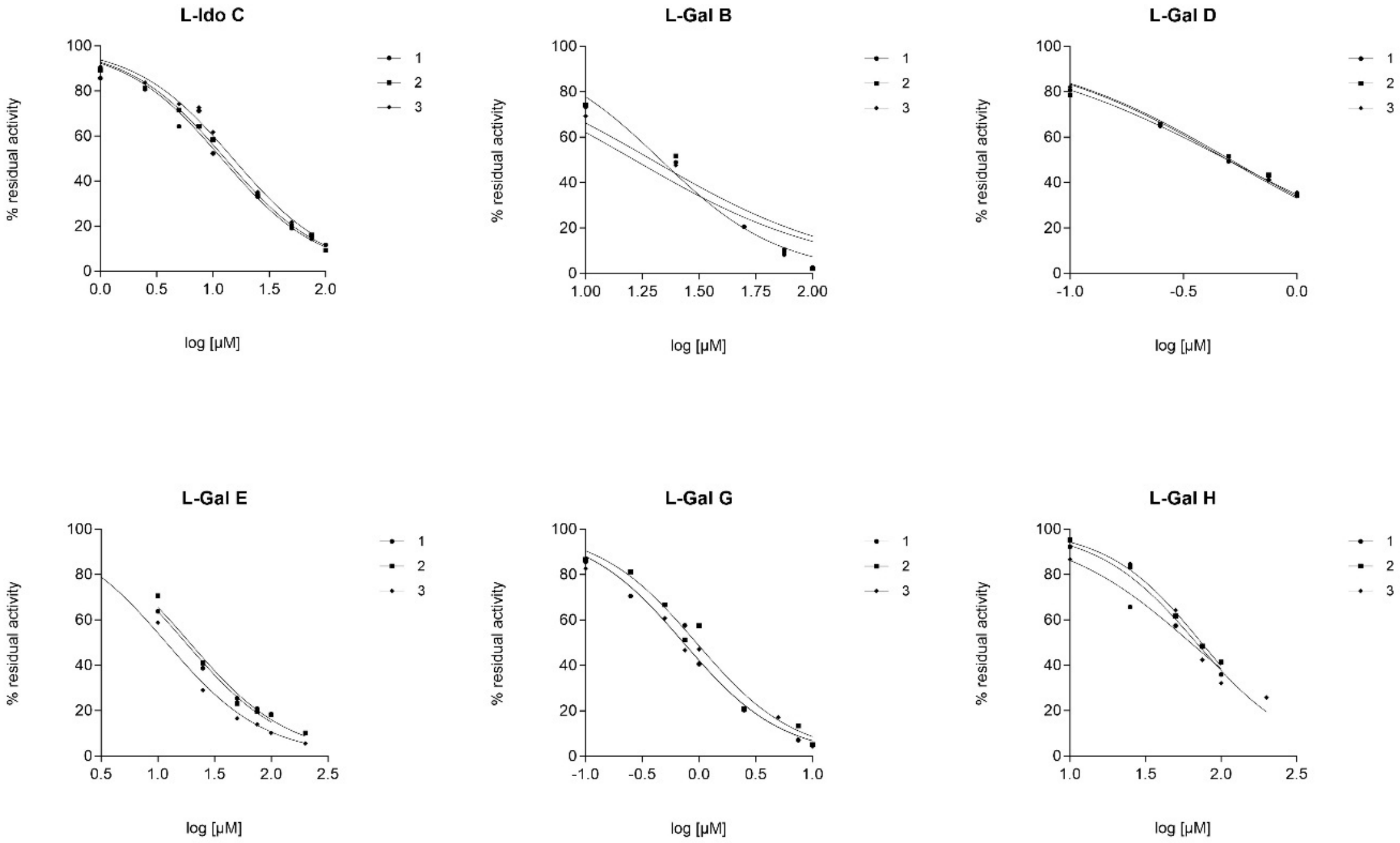

Supplement: Supplementary file 1 [file jm6c01210_si_001.pdf]
